# The PH Domain and C-Terminal polyD Motif of Phafin2 Exhibit a Unique Concurrence in Animals

**DOI:** 10.3390/membranes12070696

**Published:** 2022-07-07

**Authors:** Mahmudul Hasan, Daniel G. S. Capelluto

**Affiliations:** 1 Protein Signaling Domains Laboratory, Department of Biological Sciences, Fralin Life Sciences Institute and Center for Soft Matter and Biological Physics, Virginia Tech, Blacksburg, VA 24061, USA; 2 Department of Pharmaceuticals and Industrial Biotechnology, Sylhet Agricultural University, Sylhet 3100, Bangladesh

**Keywords:** Phafin2, PH domain, polyD motif, FYVE domain, bioinformatics

## Abstract

Phafin2, a member of the Phafin family of proteins, contributes to a plethora of cellular activities including autophagy, endosomal cargo transportation, and macropinocytosis. The PH and FYVE domains of Phafin2 play key roles in membrane binding, whereas the C-terminal poly aspartic acid (polyD) motif specifically autoinhibits the PH domain binding to the membrane phosphatidylinositol 3-phosphate (PtdIns3P). Since the Phafin2 FYVE domain also binds PtdIns3P, the role of the polyD motif remains unclear. In this study, bioinformatics tools and resources were employed to determine the concurrence of the PH-FYVE module with the polyD motif among Phafin2 and PH-, FYVE-, or polyD-containing proteins from bacteria to humans. FYVE was found to be an ancient domain of Phafin2 and is related to proteins that are present in both prokaryotes and eukaryotes. Interestingly, the polyD motif only evolved in Phafin2 and PH- or both PH-FYVE-containing proteins in animals. PolyD motifs are absent in PH domain-free FYVE-containing proteins, which usually display cellular trafficking or autophagic functions. Moreover, the prediction of the Phafin2-interacting network indicates that Phafin2 primarily cross-talks with proteins involved in autophagy, protein trafficking, and neuronal function. Taken together, the concurrence of the polyD motif with the PH domain may be associated with complex cellular functions that evolved specifically in animals.

## 1. Introduction

Phafin2, also known as PLEKHF2 or EAPF, is a member of the Phafin family of proteins, which contains an N-terminal PH domain, a central FYVE domain, and a poly aspartic acid (polyD) motif at the C-terminus [1]. Structurally, Phafin2 is a moderately elongated monomer primarily composed of both α-helical and β-strand elements, but it is also estimated to have a large contribution of random coil regions [2]. Quantitative transcriptomics analysis shows that the human *Phafin2* gene, localized on chromosome 8q22, is broadly expressed in bone marrow and lymph nodes, among other tissues [3]. Amplification of the *Phafin2* gene is associated with reduced survival in patients with prostate cancer [4].

Phafin2 is involved in multiple cellular functions, including endosomal cargo trafficking, apoptosis, macropinocytosis, and autophagy [5,6,7,8,9]. Similarly, the Phafin2 homolog Phafin1 targets lysosomes to promote autophagosome formation [10]. The multiple functions of Phafin2 (and Phafin1) are associated with its ability to bind the phosphoinositide phosphatidylinositol 3-phosphate (PtdIns3P) through both the PH and FYVE domains [1,8]. These domains are thermodynamically coupled [2], suggesting interdomain contacts. PtdIns3P is primarily found on both early endosomes and lysosomal surfaces and serves as a point of recruitment for PtdIns3P-binding effectors. For example, Phafin2 has been reported to control endosomal structure and function, primarily via its FYVE domain, in a Rab5-dependent manner [5]. Further studies established that Phafin2 associates with early endosomes [11], where it co-localizes with PtdIns3P-and Rab5 [5] and interacts with the endosomal protein early endosome autoantigen 1 (EEA1), regulating endosomal fusion and protein trafficking [11]. Likewise, the *Drosophila* Phafin2 homolog Rush controls endosomal and lysosomal cargo trafficking by interacting with a Rab GDP dissociation inhibitor and PtdIns3P [12].

Both the Phafin2 PH and FYVE domains are required for the induction of apoptosis [6] and autophagy [8]. To trigger apoptosis, Phafin2 is recruited to the endoplasmic reticulum, where it suppresses the protein unfolding response in this compartment and, simultaneously, it stimulates an increase in Ca^2+^ levels in the cytosol [6]. In the case of autophagy, both the PH and FYVE domains of Phafin2 associate with lysosomal PtdIns3P when coupled with the serine/threonine kinase AKT [8], which phosphorylates Phafin2 [13], and possibly depends on the serine/threonine kinase activity of the vaccinia-related kinase-2 [14].

Recently, it has been reported that, during macropinocytosis, Phafin2 binds newly formed macropinosomes in a process that requires the presence of two distinct pools of PtdIns3P and PtdIns4P [9]. The FYVE domain is required for the localization of Phafin2 to both the early and late steps of macropinosomal maturation, whereas the PH domain is only essential during the early steps [7]. To participate in macropinosomal maturation, the Phafin2 FYVE domain specifically associates with PtdIns3P, whereas the PH domain binds transient pools of PtdIns3P and PtdIns4P at different stages of this process [9]. Phafin2 also facilitates the transition of nascent macropinosomes through the subcortical actin network by an interaction with F-actin [9]. The maturation of macropinosomes requires not only the actin network but also regulation of the actin filament cross-linking protein Filamin A, which, upon interaction with Phafin2, sheds from the macropinosome membrane [7]. Additionally, the Phafin2 PH domain is necessary for the recruitment of the coiled-coil protein JIP-4 at macropinosomes, promoting membrane tubulation [15]. More recently, attention has been focused on the C-terminal polyD motif of Phafin2. Removal of the polyD motif does not alter Phafin2 function during macropinosome formation [7] but is required to prevent nonspecific association of the protein to the plasma membrane [9]. The polyD motif is required to downregulate the Phafin2 PH domain binding to PtdIns3P [1], suggesting that the polyD motif prevents Phafin2 binding to other phosphoinositides at the plasma membrane; however, it becomes dispensable when the Phafin2 PH domain engages with nascent vesicles generated from macropinocytosis. Here, we employed a bioinformatics approach, focusing on the correlation between the simultaneous presence of a PH domain and a polyD motif in Phafin2 homologs from bacteria to mammals. Although PH domains are found in all organisms studied, only animal Phafin2 and other related PH-, FYVE-, and polyD-containing proteins simultaneously bear both a PH domain and a polyD motif, suggesting that the PH domain–polyD motif pair appeared more recently in evolution.

## 2. Material and Methods

### 2.1. Prediction of the Three-Dimensional Structure of Phafin2

The three-dimensional coordinates of all human Phafin2 heavy atoms were predicted by Alphafold (https://alphafold.ebi.ac.uk; accessed on 21 June 2022). To predict the structure of Phafin2, Alphafold employed the amino acid sequence of protein homologs as input [16].

### 2.2. Sequence Retrieval and Acquisition

PH-, FYVE-, and polyD-containing proteins were retrieved from NCBI (https://www.ncbi.nlm.nih.gov), Ensembl genome browser (https://www.ensembl.org), and UniProt (https://www.uniprot.org) (all platforms accessed on 1 May 2021) using the human Phafin2 amino acid sequence (Accession No: NP_078889) as the query sequence. We employed blastp (protein–protein BLAST) and PSI-BLAST (Position-Specific Iterated BLAST) with default parameters and a nonredundant database. No differences in sequence outcomes were found between these two search engines. For the input of the human Phafin2 query sequence, we employed the general parameters of the nonredundant database, with an expected threshold of 0.05, matrix BLOSUM62, gap costs of 11, extension of 1, and the conditional score matrix adjustment was used as compositional adjustment. The output blastp searches were setup without fixing the limits of their statistical parameters (such as E-value, max score, total score, query cover, and percent identity) to retrieve as many of the human Phafin2 homologous sequences. Homologous sequences of human Phafin2 retrieved from the Ensembl genome browser database were setup with the target and query identities ranging between 100 to 44% and 100 to 53%, respectively.

### 2.3. Functional Protein Domain and Motif Search

Homolog sequences of human Phafin2 were screened using the MEME [17] database for identifying different motifs. The web-based domains search toolkits Pfam [18], Hmmscan [19], and InterPro [20] were employed to search for the presence of domains and motifs in PH-, FYVE-, or polyD-containing proteins from bacteria, archaea, fungi, plants, nonhuman mammals, humans, and model organisms. In the case of Hmmscan, the cut off for the identification of protein domains and motifs was carried out using the gathering method, which defines significance threshold.

### 2.4. Multiple Sequence Alignment and Phylogenetic Analysis

Retrieved PH-, FYVE-, or polyD-containing protein sequences were aligned using the multiple sequence alignment tool Clustal Omega v2.0.12 (EMBL-EBI, Wellcome Genome Campus, Cambridgeshire, UK, https://www.ebi.ac.uk/Tools/msa/clustalo/ [21] (accessed on 27 December 2021) with default parameters (MBED-like clustering guide-tree: yes; MBED-like clustering iteration: yes). After the assessment by multiple sequence alignment (MSA), phylogeny analysis was performed to understand the diversity and evolutionary trends of homolog sequences of human Phafin2 proteins. The analyses were achieved using the Molecular Evolutionary Genetics Analysis X (MEGA X) (University of Pennsylvania, Philadelphia, PA, USA) [22] (accessed on 15 December 2021)). An unrooted phylogenetic tree was generated with 203 retrieved Phafin2 homolog sequences using the maximum likelihood method of MEGA X platforms. Bootstrapping with 1000 replicates was performed to ensure the maximum reliability of individual branches of the tree. The MEGA parameters were as follows: model/method: Jones–Taylor–Thornton (JTT) model; rates among sites: uniform rates; gaps/missing data treatment: use all sites; ML heuristic method: nearest-neighbor interchange; initial tree for ML: NJ/BioNJ; branch swap filter: none; and statistical method: maximum likelihood method. A Newick file generated from MEGA X was employed in the iTOL website for the display, annotation, and management of our phylogenetic tree. The PH, FYVE, and polyD sequence patterns were displayed in the constructed tree by using the advanced dataset settings of iTOL [23]. Amino acid conservation and sequence logo alignment were generated using Jalview.

### 2.5. Protein–Protein Interactions Analyses

The protein–protein interactions network of human Phafin2 and *Drosophila melanogaster* Rush was predicted using the STRING database. Human Phafin2 was used as the query search in the STRING database, and molecular interactions with a confidence score ≥90% were considered to avoid false-positive results [24].

## 3. Results and Discussion

### 3.1. Genomic Features of the polyD Motif in Phafin2′s Human Homologs

**Figure 1:**
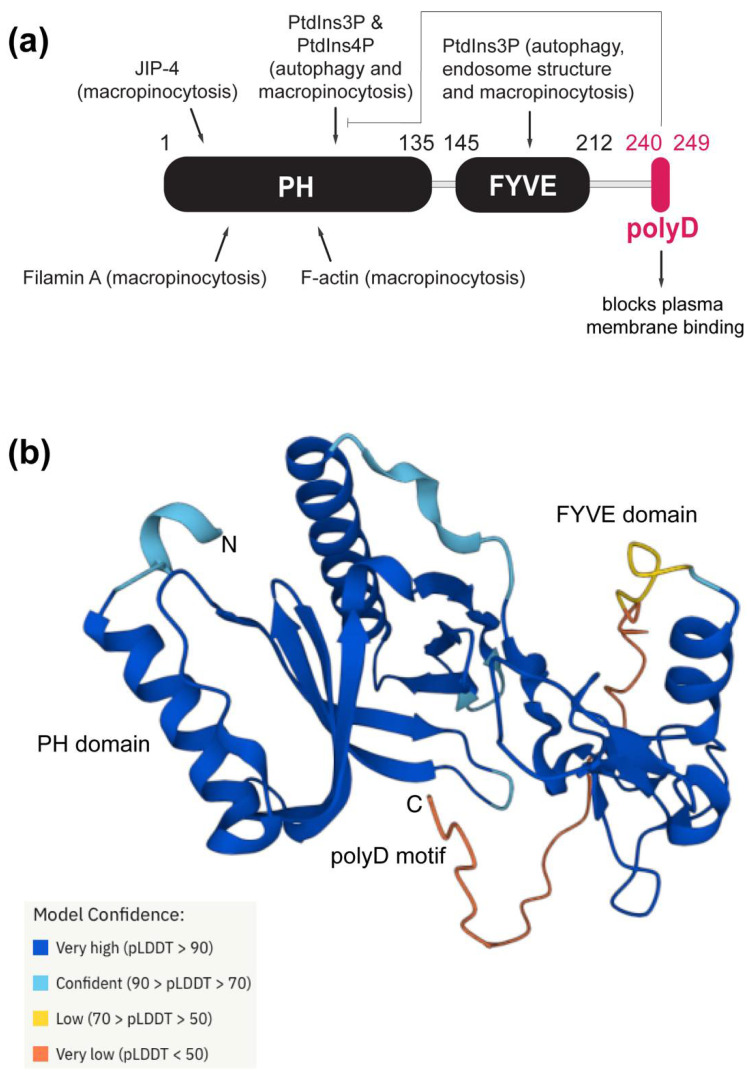
Structural features, ligands, and functions of the Phafin2 domains and motif. (**a**) Domain and motif architecture of human Phafin2. Ligand interactions are shown for each conserved unit of the protein with the associated function in parenthesis. (**b**) Estimated three-dimensional structure of human Phafin2 retrieved from AlphaFold. The model of confidence of the structure is color coded.

**Figure 2:**
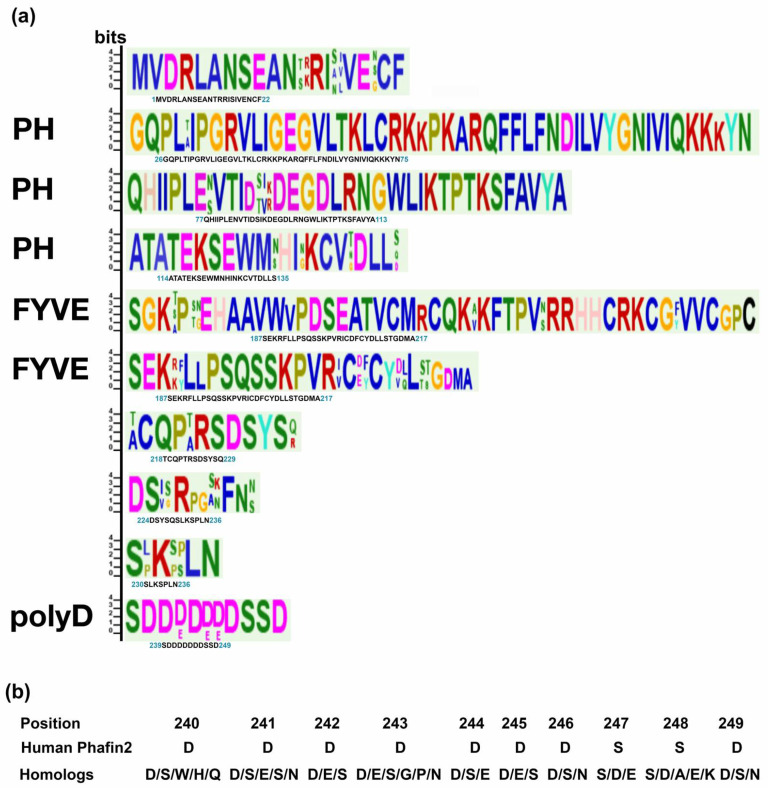
Consensus sequences found in mammalian Phafin2 and PH-, FYVE-, and polyD-containing proteins. (**a**) Sequences associated with the PH and FYVE domains as well as with the polyD motif are labeled. Logo sequences were obtained from protein sequences of human origin. (**b**) Substitutions in the polyD motif identified in Phafin2 homologs.

Homolog proteins are usually the preliminary platforms used for evolutionary studies and the functional annotation of proteins [25]. Phafin2 is composed of an N-terminal PH domain, a central FYVE domain, and a C-terminal polyD motif (Figure 1). To obtain structural insights, we retrieved the estimated three-dimensional structure of human Phafin2 from AlphaFold. Whereas the PH and FYVE domains are displayed as globular domains, the polyD motif-containing C-terminal region is predicted to be disordered, thus, providing conformational flexibility for protein interactions, including the intramolecular association with the PH domain (Figure 1). MEME identified ten highly conserved regions in Phafin2, including the C-terminal polyD motif. Most of the conserved amino acids are found within the PH and FYVE domains (Figure 2). The polyD motif of human Phafin2 is ten-amino acids long (240-DDDDDDDSSD-249), but variable amino acid positioning was identified in protein homologs (Figure 2). Although highly conserved, serine residues can also be present in other positions within the motif (Figure 2). Quantitative phosphoproteomic studies indicated that human Phafin2 serine residues at positions 239, 247, and 248 are phosphorylated [26], emphasizing the role of these residues within the polyD region. In addition, aspartic acid was often found to be replaced by glutamic acid within the polyD motif (Figure 2), indicating that the conserved acidic nature of the motif is relevant for the function of Phafin2.

**Figure 3:**
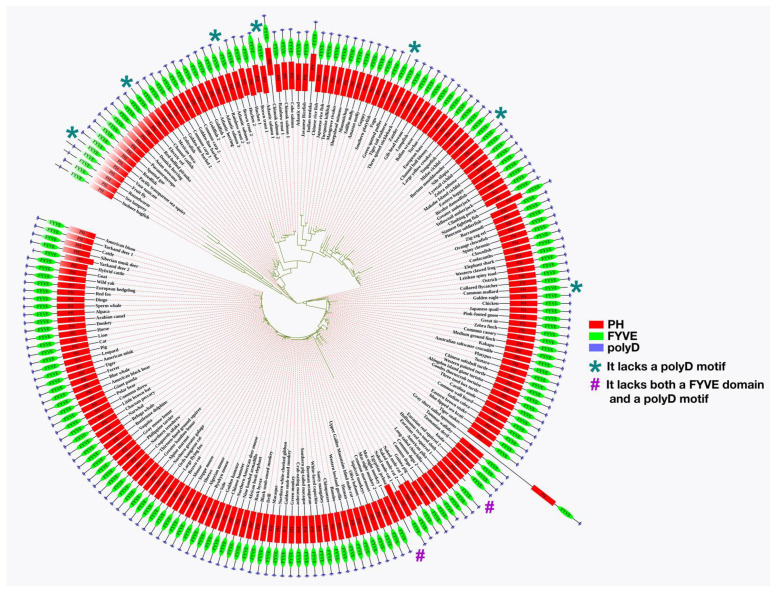
Phylogenetic analysis of Phafin2 homologs retrieved using the Ensembl genome browser. The analysis of Phafin2 ancestral sequences was generated using MEGA X software, and domain structure was constructed using iTOL software.

Features of the polyD motif in human Phafin2 has been described comparatively to its homolog proteins. A total of 203 homologs of human Phafin2 (Supplementary File S1) were employed in the phylogenetic study, and the results indicated that Phafin2 and other related PH-, FYVE-, or polyD-containing proteins of *Gorilla gorilla* (gorilla), *Pan troglodytes* (chimpanzee), and *Pan paniscus* (bonobo) were evolutionarily closer to human Phafin2 than other organism proteins (Figure 3). Most homologs were found to have PH, FYVE, or polyD units, but vase tunicate, channel catfish, Atlantic salmon, green spotted puffer, burtons mouthbrooder, and common mallard have no polyD motif encoded with their PH-FYVE domains (Figure 3). Moreover, Hoffmann’s two-toed sloth and Egyptian zebra contain a PH domain, but they lack both FYVE and polyD units (Figure 3). The retrieved homolog sequences from the *human genome browser 105* includes 26 species of primates, 32 species of rodents, 43 species of carnivores, ungulates, and insectivores, 106 species of mammals, 69 species of reptiles and birds, and 86 species of fishes. Among 203 sequences, 78 homologs were from mammals, which indicates a relevant pool of Phafin2 proteins available in mammalian species (Table S2). Most importantly, MSA analysis shows that all human Phafin2 homologs in other organisms usually carry the C-terminal acidic polyD region alongside the PH and FYVE domains (Supplementary File S2).

### 3.2. Concurrence of the polyD Motif with the PH and FYVE Domains in Phafin2 and Other Related PH-, FYVE-, or polyD-Containing Proteins

As indicated in the following sections, the presence of the PH and FYVE domains as well as a polyD motif are signatures of Phafin2. However, other unrelated proteins having only PH or FYVE domains are expressed in both prokaryotes [27] (this article) and eukaryotes [28,29]. The presence of the polyD motif in human Phafin2 raises the question as to whether it is exclusively found in humans and other mammals, or whether it evolved or is evolutionarily encoded in Phafin2 and other related PH-, FYVE-, or polyD-containing proteins of other kingdoms. Database searches sometimes provide sequences from the whole-genome sequencing (WGS) that might be contaminated from environmental sources. However, filtering WGS sequences did not affect our data analysis. Analysis of the evolution and concurrence of the polyD motif with the PH and FYVE domains in Phafin2 and other related PH-, FYVE-, or polyD-containing proteins in different organisms is provided in the following sections.

#### 3.2.1. Bacteria

**Figure 4:**
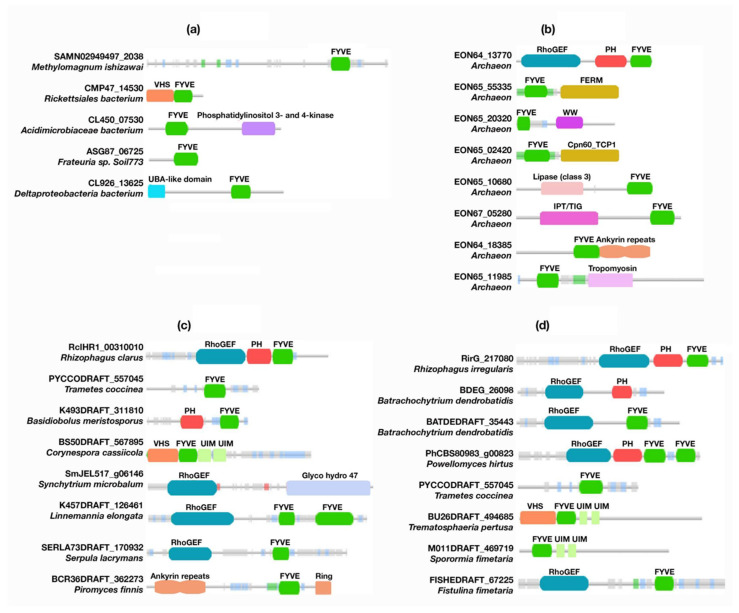
Schematic representation of PH- and FYVE-containing domains found in proteins from bacteria (**a**), archaea (**b**), fungi (**c**), and plants (**d**). Other unique domains are also shown.

Unlike canonical PH domains and polyD motifs, FYVE domains, of unknown function, were found in about 40 bacterial proteins. Other protein domains, such as Vps27, Hrs, STAM (VHS), phosphatidylinositol 3- and 4-kinases, and ubiquitin-associated (UBA)-like domains, were found to be encoded together with the FYVE domains (Table S1, Figure 4 and Figure S1, and Supplementary Files S3 and S4). Whereas eukaryotic VHS domains are involved in vesicular trafficking by either ubiquitinated cargo recognition or through phosphorylated receptor binding [30], bacterial VHS domains facilitate the subcellular localization of protein effectors [31]. Likewise, UBA-like domains are associated with the ubiquitin/proteasome pathway in eukaryotes, but are uncommon domains of unknown function in bacteria [32,33]. The same applies for phosphatidylinositol 3- and 4-kinases, which phosphorylate phosphoinositides in eukaryotes and whose activities are associated with cell cycle regulation, DNA recombination, and the DNA damage checkpoint, among other functions [34,35,36]. A PH-like domain (PH*b*) was reported in bacterial species; however, our database search did not recognize it as there were genomic dissimilarities with canonical PH domains [27]. Genome-wide studies of *Bacillus subtilis* reported three paralogs of PH*b*, which indicates common functional features of PH*b* proteins [37,38,39,40]. PH*b* was also found in the Min1 phage from the nematode pathogen *Microbacterium nematophilum* (ORF77) [41] and *Lactococcus bacteriophage* ul36 (ORF124) [42]. PH*b* homologs were also identified in other bacteria, such as *Oceanobacillus iheyensis48* (Swiss-Prot: Q8ELK9) and *Streptomyces coelicolor47* (SCO3793, Swiss-Prot: Q9F325). The availability of PH-like domains in bacterial species highlights that eukaryotic PH domains do not represent a new feature in these organisms; rather, they may be evolved or diverged from prokaryotes [27]. Despite having two categories of *PHb*, known as *PHb1* and *PHb2,* that are based on their size and ring structure symmetry, they are assumed to have the same function in bacterial cells. A genome-wide study found that three paralogs of PH*b*, YjqA, YozO, and YvbH, are expressed in *B. subtilis.* YozO is associated with cell stress responses, whereas YvbH is a membranal peripheral protein [39,40,43]. A YozO homolog, involved in cell envelope stress response activities, was detected in *Bacillus licheniformis* [44]. However, *PHb1* was confirmed in *Lactococcus bacteriophage ul36* (ORF124)45 and Min1 phage, indicating that *PHb* might be involved in the phage life cycle [41]. Phosphoinositides are considered absent in *E.coli* and are only found in small bacterial and archaeal groups [45]. The unavailability of a canonical PH domain in bacteria, such as the one found in Phafin2, indicates that eukaryotic PH domains might evolve in response to a natural selective pressure, and some gained the ability to bind phosphoinositides as a mechanism for organelle localization.

#### 3.2.2. Archaea

Interestingly, while PH domains were found in tandem with FYVE domains, the polyD motif was not encoded in 68 archaeal proteins retrieved from the database (Table S1, Figure 4 and Figure S2, and Supplementary Files S3 and S4). The classic phylogeny study by Woese and co-workers, and a subsequent evolutionary study of universal genes, suggested that archaea are evolutionarily more convergent with eukaryotic organisms than with bacteria, and it was assumed that eukaryotic cells emerged from an ancient archaeon cellular system [46,47]. The presence of PH domains in archaea clearly indicates that these domains emerged among archaea species as an ancestor of all PH domains that were further evolved or emerged in other eukaryotic organisms. A Rho guanine nucleotide-exchange factor (GEF)-FYVE-PH tandem domain was identified as predominant among archaea proteins (Figure 4). In eukaryotes, the RhoGEF domain regulates G protein signaling, where it stimulates GDP release and, consequently, GTP binding for the activation of specific Rho family proteins [48].

Interestingly, FERM N-terminal, WW, lipase (class 3), Ig-like, plexins, transcription factors (IPT), and tropomyosin domains as well as ankyrin repeats were also found in tandem with archaea FYVE domains. In eukaryotes, the FERM domain is associated with protein localization to the plasma membrane, accelerating signaling pathways [49,50]. Curiously, the FERM domain of human Kindlin-2 contains an inserted PH domain that specifically binds PtdIns3P and phosphatidylinositol 3,4,5-trisphosphate [PtdIns(3,4,5)P_3_]. This insertion may allow Kindlin-2 to target the plasma membrane, facilitating the regulation of integrin receptors [51]. On the other hand, lipase (class 3), IPT, and tropomyosin domains as well as ankyrin repeats, are universally distributed in prokaryotes, plants, and animals and are involved in a wide range of cellular functions. For example, lipase class 3 is associated with lipid degradation, esterification, and transesterification, whereas the tropomyosin domain is required for the actin–myosin interactions [52,53]. Ankyrin repeats are repetitive short sequences involved in protein–protein interactions, mediating a plethora of functions [54]. Thus, it is possible that the membrane binding of archaea PH- and FYVE-containing proteins are required for lipid and protein interactions.

#### 3.2.3. Protozoans

To investigate protozoan PH-, FYVE-, or polyD-containing proteins, we employed a literature-based approach as there is no BLAST option for protozoans available in the NCBI and UniProt databases. Several reports highlighted the presence of PH and FYVE domain-containing proteins in protozoans. For example, eleven FYVE- and two PX domain-containing proteins were found as potential PtdIns3P-binding proteins, and one single PH domain-containing protein was detected as a PtdIns4P-binding protein in the *Entamoeba histolytica* genome [55]. Among the PtdIns3P-binding proteins, a predominant conserved modular organization is represented by RhoGEF-PH/FYVE or by just the FYVE or PX domains only, but all lack a polyD motif. The protozoan parasite *Leishmania major* contains five putative FYVE domain proteins displaying functional PtdIns3P-binding sites [56]. The FYVE domain has also been identified and characterized in *Giardia lamblia* [29]. For the identification of a polyD motif in protozoans, an NCBI pBLAST was employed using human Phafin2 as a query sequence with the FYVE domain-containing sequences of *E. histolytica, L. major,* and *G. lamblia*. No sequences from *G. lamblia* were found to be aligned with human Phafin2 in the BLAST search. Interestingly, protozoan sequences aligned with human Phafin2 have no polyD motif, suggesting that their membrane binding properties through the PH domain may be modulated by an alternative route. The most aligned protozoans’ protein sequence was from *E. histolytica* with 71% query cover and 43% identity among all retrieved sequences of *E. histolytica and L. major* together. The BLAST and MSA results of protozoan proteins are shown in Supplementary File S5.

#### 3.2.4. Fungi

Approximately forty Phafin2-related proteins from fungi were retrieved from the database. All these proteins lacked a polyD motif. RhoGEF-PH-FYVE and VHS-FYVE-ubiquitin-interaction motif (UIM) tandem domains were identified in fungal proteins (Table 1, Figure 4 and Figure S3, and Supplementary Files S3 and S4). In addition, glycosyl hydrolase family 47, ankyrin repeats, and ring finger domains were detected in these organisms. Both UIM and ring finger domains play significant roles in ubiquitin moiety interactions present in cargo [57]. Thus, the presence of a FYVE domain may contribute to membrane recruitment and prelocalization of fungal proteins. The presence of a PH domain has been reported in the fungal Target of Rapamycin (TOR) complex. Fungal species present two isoforms, TORC1 and TORC2, which are also found in mammals. The physiological fate of mTOR signaling lies in the regulation of cell growth in response to nutrient uptake or growth factors [58,59]. Fungal TORC2 is composed of six subunits (Tor2, Avo1, Avo2, Avo3, Lst8, and Bit61) [60], with a PH domain found only in the Avo1 subunit. The PH domain of Avo1 binds phosphatidylinositol 4,5-bisphosphate [PtdIns(4,5)P_2_], allowing the recruitment of TORC2 to the plasma membrane [61]. Although no direct downregulation of Avo1 PtdIns(4,5)P_2_ has yet been reported, it is speculated that calcineurin modulates mTORC2 function by an uncharacterized molecular mechanism [62]. As fungal TORC2 plays major roles in sensing, homeostasis, and modulation of the lipid/protein composition of the plasma membrane as well as for the actin cytoskeleton and actin-driven endocytosis [61,63,64], the presence of a PH domain would serve as a membrane anchor for carrying out these functions.

The phosphoinositide-specific PH domain-containing phospholipase C (PLC) has been studied in different fungal species [65]. PLC catalyzes the hydrolysis of PtdIns(4,5)P_2_, which leads to the production of the secondary messengers inositol 1, 4, 5-trisphosphate and diacylglycerol [66]. The PH domain is localized at the N-terminus of PLCs and is responsible for membrane PtdIns(4,5)P_2_ binding [67]. PLCs, such as those from PLC1 from *Alternaria alternata* [68] and *Magnaporthe oryzae* [69], encode a PH domain, which is in tandem with the EF-hand Ca^2+^-binding, the catalytic X and Y, and the phospholipid-binding C2 domains. Regulation of phospholipid-binding of PLC is mediated by association of PtdIns4P 5-kinase and Ca^2+^ to the PH and C2 domains, respectively [70]. One of the molecular mechanisms suggested for the regulation of the mammalian PLC PH domain binding to phosphoinositides is that Ca^2+^ controls phosphoinositide headgroup conformation and recognition [71]. Not all fungal PLCs have a PH domain. For example, *Botrytis cinerea* have two PLC-encoding genes, *bcplc1* and *bcplc2*, where bcplc1 only contains a PH domain [72], suggesting that membrane binding by bcplc2 is driven by its C2 domain. Similarly, *Coprinopsis cinerea*, a multicellular basidiomycete mushroom employed as a model organism in eukaryotes [73], has three putative PLC genes, *CcPLC1*, *CcPLC2*, and *CcPLC3*, with the first two encoding for a PH domain [74], whereas *Cryphonectria parasitica* PLC does not have a PH domain [75]. In summary, few fungal PH domains have been reported to bind PtdIns(4,5)P_2_, and the absence of a polyD motif in these proteins suggests the presence of an alternative regulatory mechanism for phosphoinositide binding.

#### 3.2.5. Plants

Plants possess a vast number of functional activities related to PH- and FYVE-containing proteins. Our study found ~250 sequences from database searches, but it is possible that some of these sequences correspond to contaminants from fungal species. Moreover, no polyD motif was identified in plant proteins. Instead, the PH and FYVE domains were present in the form of RhoGEF-PH-FYVE and VHS-FYVE-UIM-UIM tandem domains. In addition, RING finger and ankyrin repeats were encoded with FYVE domains (Table 1, Figure 4 and Figure S4, and Supplementary Files S3 and S4). The PH domain is the predominant lipid-binding domain of plants. About sixty PH domain-containing proteins have been detected in the *Arabidopsis* genome [76]. Plant PH domains preferentially exhibit an association with phosphoinositides and phosphatidic acid, leading to widely diverse functions. For example, the *Medicago truncatula* ZR1 protein regulates root and nodule development, which might be mediated by interactions of its PH and FYVE domains with phosphoinositides and phosphatidic acid [77]. However, the regulation of ZR1 interactions with phospholipids remains to be investigated. Other studies involve the role of the PH domains of proteins that lack FYVE domains. *Arabidopsis thaliana* AtPH1 is a late endosomal, vacuolar, and multivesicular body protein that regulates vacuolar metal transporter localization through the interaction of its PH domain with PtdIns3P [78]. *A. thaliana* SEC3 is a PH domain protein that binds membrane PtdIns(4,5)P_2_, which is required for pollen tube growth and provides the site of pollen germination [79]. Indeed, SEC3 function might be restricted by subcellular localization since the protein can be regulated by both PtdIns(4,5)P_2_-dependent and -independent mechanisms [79]. Moreover, plant enzymes bear PH domains as they occur with the phosphatidylinositol-4-kinase, which produces PtdIns4P, the most abundant phosphoinositide required for the control of cell membrane identity [80].

#### 3.2.6. Animals

**Figure 5:**
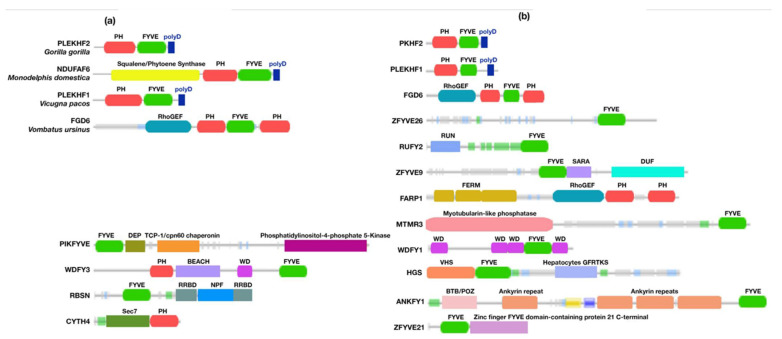
Schematic representation of PH-, FYVE-, and polyD-containing modules found in proteins from nonhuman mammals (**a**) and humans (**b**). Other unique domains are also shown.

In animals, the polyD motif is encoded in Phafin2 and other related PH-, FYVE-, or polyD-containing proteins (Supplementary Files S1 and S2). Our analysis focused on the presence of Phafin2 and other related PH-, FYVE-, or polyD-containing proteins in nonhuman mammals since they are the closest relatives to humans. In nonhuman mammals, the polyD motif is consistently found at the C-terminus, downstream of both the PH and FYVE domains. However, PH domain-containing proteins that lack both FYVE and polyD units were also identified (Figure 5). In addition, the squalene synthase catalytic activity and the presence of a RhoGEF domain are found in some proteins with PH and FYVE domains as well as a polyD motif (Table 2, Figure 5 and Figure S5, and Supplementary Files S3 and S4). In mammals, membrane-bound squalene synthases are associated with the cholesterol biosynthetic pathway [81]. RhoGEF domains, which regulate the function of GTPases, are predominantly found in tandem with the Dbl-homology (DH), PH, and FYVE domains leading to a wide range of cellular activities [82,83].

About 170 homologous Phafin2 sequences are associated with humans. Unlike PH-, FYVE-, or polyD-containing proteins, the polyD motif is encoded together with the PH and FYVE domains in Phafin1 and 2 (Supplementary Figure S6 and File S4). Phafin1 (PLEKHF1; Figure 5) shares a high sequence similarity with Phafin2, but it presents an additional tail of 27 amino acids downstream of the polyD motif [10]. Phafin1 is not only involved in autophagosome formation [10], but is also linked to apoptosis [84], caveolae-associated endocytosis, bacteria removal in macrophages, and the induction of innate immunity [85].

Human FYVE domains are often associated with other domains than PH domains (Table 2, Figure 5, and Supplementary File S3). Other polyD-free PH- and FYVE-containing proteins contain alternative tandem domains, such as RhoGEF-PH-FYVE-PH, FERM N-terminal-FERM-central-FERM C-terminal-PH-like-FERM adjacent, RhoGEF-PH-PH, RUN-FYVE, myotubularin-like phosphatase-FYVE, WD-G-β repeat-WD-G-β repeat-WD-G-β repeat-FYVE-WD-G β repeat, VHS-FYVE-hepatocyte growth factor-regulated tyrosine kinase substrate (GFRTKS), ankyrin repeats-FYVE, FYVE-Zinc finger FYVE domain-containing protein 21 C-terminus, FYVE-DEP-TCP-1/cpn60 chaperonin family-PtdIns4P 5-kinase, PH-BEACH-WD-G-β repeat-FYVE, FYVE-Rabenosyn Rab binding domain (RRBD)-Rabosyn-5 repeating NPF sequence-motif-RRBD, and Sec7-PH domain domains. PH domains are predominantly found in human proteins that lack both FYVE domains and polyD motifs and alternative mechanisms of regulation of PH domains are proposed [86]. Phosphoinositide synthesis and turnover as well as post-translational modifications are the most common mechanisms for the modulation of PH domain function, as occurs with mTOR proteins [87]. Interestingly, an alternative mechanism has been reported for the human PH-containing protein Cytohesin-4, which is involved in vesicular trafficking pathways [88]. Cytohesin-4 contains a tandem of Sec7-PH domains, and instead of a polyD motif, presents an N-terminal coiled-coil domain that autoinhibits PH domain binding to membrane phosphoinositides [89]. Likewise, the PH domain-containing oxysterol-binding proteins (OSBPs) are cholesterol transfer proteins that modulate the Golgi apparatus organization and function [90]. It has been proposed that, in the absence of sterols, the OSBP sterol-binding domain intramolecularly interacts with the PH domain, inhibiting PtdIns4P binding and restricting the protein at the endoplasmic reticulum [91]. Sterol binding promotes an open conformational state in OSBP, which relieves the autoinhibition of its PH domain, consequently facilitating binding to Golgi apparatus membranes via PtdIns4P interactions [90]. A recent study estimates that about 50% of human PH domain-containing proteins do not bind phosphoinositides [92]. For example, the human FERM, ARH/RhoGEF, and pleckstrin domain protein 1 (FARP1) protein displays a modular FERM N-terminal-FERM central-FERM C-terminal PH-like-FERM adjacent-RhoGEF-PH-PH modular unit. Membrane lipid-binding of FARP1 appears to be FERM domain-dependent but PH domain-independent [93]. The second PH domain of FARP1 has been suggested to intramolecularly modulate the binding of the first PH domain and the DH domain to Rho GTPases [94].

The presence of a polyD motif in Phafin2 and its homologs facilitates a unique regulatory mechanism of PH domains. Thus, the Phafin2 C-terminal polyD motif downregulates the binding of the PH domain to PtdIns3P [1], impairing spurious plasma membrane targeting [9]. Phafin2 forms a complex with lysosomal PtdIns3P and AKT, inducing autophagy [8]. AKT presents an N-terminal PH domain that binds PtdIns(3,4,5)P_3_ with high affinity [95]. Similar to that found in Phafin2, the C-terminal tail of AKT is enriched in acidic residues, which can be phosphorylated at Ser473, Ser477, and Thr479. Ser473 phosphorylation is required to displace the PH domain from the kinase domain, leading to an increase in its catalytic activity [96]. On the other hand, phosphorylation of the AKT C-terminal acidic tail at Ser477 and Thr479 is required to reduce the affinity of the PH domain for PtdIns(3,4,5)P_3_ [96], by targeting the PH domain C-terminal α-helix [97]. Interestingly, the soy bean-derived peptide lunasin, which contains a C-terminal polyD motif, inhibits the anti-autophagic phosphorylation of AKT at Thr308 and Ser473 [98], a cellular process that is required for the binding of AKT to PtdIns(3,4,5)P_3_. Other PH domains can be modulated by poly acidic regions. For example, the cell cycle transcription factor DP1 contains an acidic region that binds to the PH domain of the p62 subunit of the transcription factor IIH, serving as a mechanism of transcriptional activation [99]. However, it is not known whether this association controls p62 PH domain membrane binding through PtdIns3P and PtdIns5P [81]. Thus, it is possible that poly acidic regions regulate the activity of PH domains, independent of the presence of a FYVE domain.

### 3.3. The Presence of Phafin2 or Related Proteins in Model Organisms and Their Structural Features

The study also extended to different nonmammalian model organisms, such as *Arabidopsis thaliana*, *Saccharomyces cerevisiae* (yeast), *Danio rerio* (zebrafish), *D. melanogaster* (fruit fly), *Xenopus tropicalis* (western clawed frog), and *Caenorhabditis elegans* (nematode worm). Unlike *A. thaliana*, *S. cerevisiae*, *D. melanogaster*, *X. tropicalis*, and *C. elegans*, our search indicates that only *D. rerio* contains a protein encoding a polyD motif together with the PH-FYVE domains.

Data analysis indicates that in *Arabidopsis* species, we found a similar pattern of PH or FYVE domains to those described before for other plant and fungal species. Both PH and FYVE domains were found in *Arabidopsis* but never encoded together. Consistent with that found in plants, the polyD motif is absent in *Arabidopsis*. The most abundant domains were FYVE-, PH-, Las17-binding protein actin regulator-FYVE, phosphatidylinositol-4-phosphate-5-kinase-TCP-1/cpn60 chaperonin family-FYVE, BAR domain of APPL family, putative GTPase-activating protein for Arf, and ankyrin repeats. The autophagy-linked FYVE (Alfy) protein contains a tandem of FYVE, BEACH, and WD40 domains, allowing association to the autophagy receptor p62 and PtdIns3P [100,101]. Interestingly, *Arabidopsis* Alfy-like proteins contain both PH and BEACH domains but lack a FYVE domain [102,103]. In *A. thaliana*, the BEACH domain-containing proteins (BDCPs) are expressed by the gene *SPIRRIG* (SPI) [104,105]. Plant BDCPs are known to initiate membrane-dependent cellular processes [105]. SPI contains a tandem of PH-BEACH domains followed by five WD40 repeats. Unexpectedly, the SPI PH domains do not bind phospholipids [106], suggesting that the binding of SPI to endosomal membranes is phospholipid-independent. About fifteen genes encoding FYVE domain-containing proteins are reported in the *Arabidopsis* genome [107], where thirteen of them do not have homologous sequences in yeast and mammals [108]. Two *Arabidopsis* FYVE domain-containing PtdIns(3,5)-kinases, FAB1A and FAB1B, play a significant role in the plant cellular environment, and depletion of their expression leads to pollen abortion, delayed endocytosis, acidification, or abnormal vacuole formation [108,109,110]. The plant FYVE domain protein FREE1 has been reported for cargo membrane protein sorting, vacuole formation, multivesicular body biogenesis, and autophagic-mediated degradation [111,112,113]. Similarly, the cell death-related endosomal FYVE/SYLF protein 1 (CFS1) was found to be associated with endosomal trafficking and autophagy [114].

**Figure 6:**
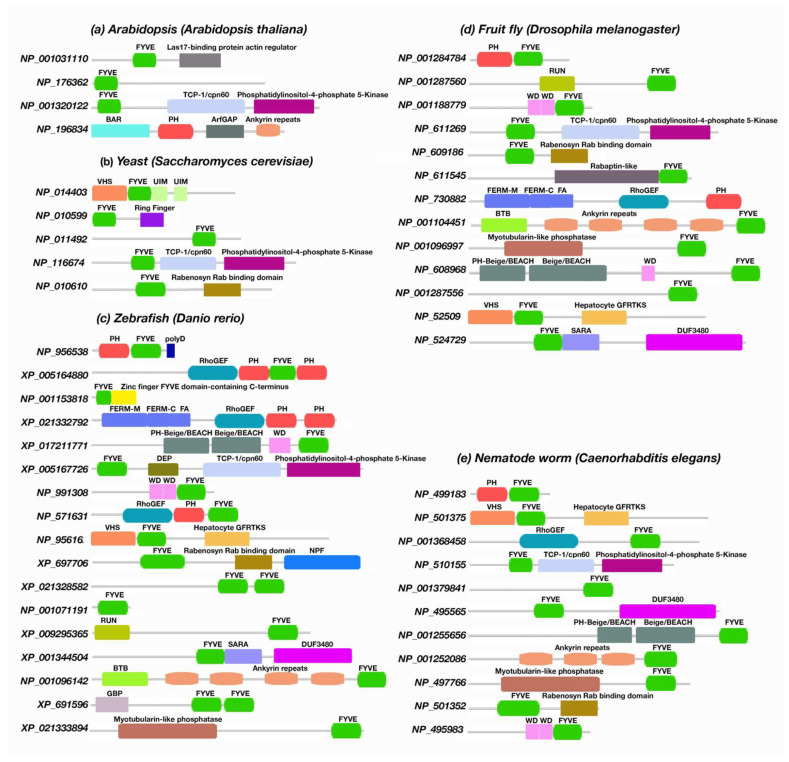
Availability of PH-, FYVE-, and polyD-containing modules in model organisms. (**a**) *Arabidopsis thaliana*, (**b**) *Saccharomyces cerevisiae*, (**c**) *Danio rerio*, (**d**) *Drosophila melanogaster*, and (**e**) *Caenorhabditis elegans*.

Both PH and FYVE domains were found to be encoded together without a polyD motif in *D. melanogaster*. The FYVE domain encoded with the RUN, WD, phosphatidylinositol-4-phosphate 5-kinase, and Rabenosyn Rab binding domains, and VHS were frequently found in *Drosophila.* These multidomain proteins are mainly associated with membrane trafficking, endocytic cargo transport, and autophagy [115,116,117,118,119]. No PH domains nor polyD motifs were identified in *S. cerevisiae*. The FYVE domain was found to be encoded in proteins with phosphatidylinositol-4-phosphate 5-kinase, VHS, UIM, and Rabenosyn Rab binding domains, in agreement with our findings in other fungi species. *D. rerio* is the only model organism that we identified as bearing a protein (NP_956538) with a polyD motif at the C-terminus, downstream of the PH and FYVE domains. The sequence of NP_956538 was stored in the UniProt database as the part of the NIH-Zebrafish Gene Collection (ZGC) project. Despite this, we did not find any experimental study associated with Phafin2 expressed in *D. rerio*. The *Gene Ontology annotation of EMBL-EBI* suggested its function as human Phafin2; that is, as a phosphatidylinositol binding protein that is associated with early endosomes [120]. In zebrafish, other functional domains, such as RUN, RhoGEF, WD, phosphatidylinositol-4-phosphate 5-kinase, Rabenosyn Rab binding, myotubularin-like phosphatase, and Beige/BEACH domains were identified to be encoded with PH, FYVE, or PH-FYVE domains (Figure 6).

In *C. elegans*, we found a protein (NP_499183) that encodes PH and FYVE domains together, but it lacks the polyD motif. Despite this, the protein was stored on the NCBI database as an uncharacterized protein [121]; the presence of both PH and FYVE domains suggests a similar Phafin2 function. Other proteins are PH-free but with a FYVE domain encoded together with VHS, RhoGEF, phosphatidylinositol-4-phosphate 5-kinase, myotubularin-like phosphatase, Rabenosyn Rab binding, WD, and Beige/BEACH domains (Figure 6 and Supplementary File S6). The *protein–protein NCBI BLAST* search did not show any PH-, FYVE-, or polyD-containing proteins in *X. tropicalis*. However, homologs of ALFY were reported in *X. tropicalis*, with a role in assisting autophagic progression by selectively degrading protein aggregates [122]. The FYVE domain-containing protein EEA1 was also reported in *X. tropicalis* [123]. A homolog of human ANKFY1 protein was found in *X. tropicalis*, a FYVE domain-containing protein involved in a variety of cellular functions, including Rab5 regulation, motor function, and early endosomal maturation [124,125].

### 3.4. Protein–Protein Network and Functional Lineage of Human Phafin2

**Figure 7:**
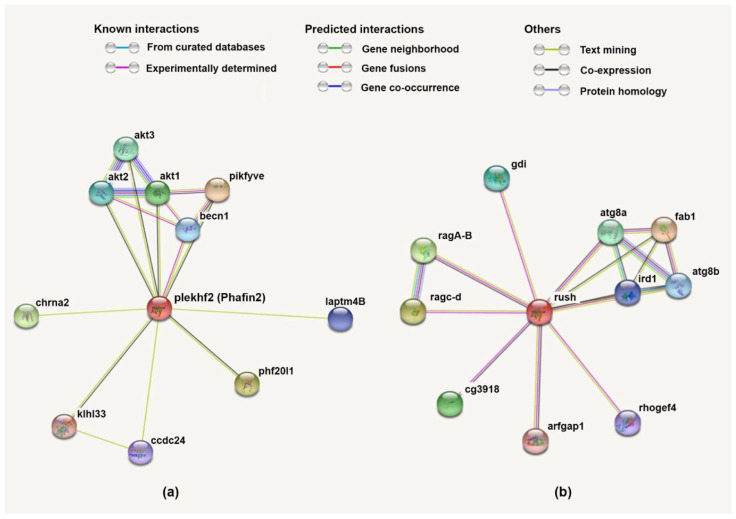
Protein–protein interaction network of human Phafin2 (**a**) and *D. melanogaster* Rush (**b**) generated using the STRING database. The color of the lines connecting the proteins indicates the typology of the protein–protein interaction. (**a**) BECN1, Beclin-1; PIKFYVE, PtdIns3P 5-kinase; PHF20L1, PHD finger protein 20-like protein 1; CHRNA2, neuronal acetylcholine receptor subunit α-2; LAPTM4B, lysosomal-associated transmembrane protein 4B; CCDC24, coiled-coil domain-containing protein 24; KLHL33, Kelch-like family member 33. (**b**) Atg8a, autophagy-related 8a; Atg8b, autophagy-related 8b; ird1, immune response-deficient 1; Gdi, guanine dissociation inhibitor; fab1, formation of aploid and binucleate cells 1; ArfGAP1, ADP-ribosylation factor GTPase-activating protein-1; Rho GEF4, Rho guanine exchange factor 4.

The protein–protein interaction network of human Phafin2 was retrieved using the STRING database, and proteins involved in this network were analyzed for their reported functions. The proteins AKT1, AKT2, AKT3, Beclin-1 (BECN1), PtdIns3P 5-kinase (PIKFYVE), PHD finger protein 20-like protein 1 (PHF20L1), neuronal acetylcholine receptor subunit α-2 (CHRNA2), lysosomal-associated transmembrane protein 4B (LAPTM4B), coiled-coil domain-containing protein 24 (CCDC24), and Kelch-like family member 33 (KLHL33) were found in the protein–protein network of human Phafin2 (Figure 7). Interestingly, several of these proteins participate in autophagy. Both AKT1 and AKT2 are found to interact with Phafin2, and the interaction of AKT2 with Phafin2 plays a critical role in the induction of lysosomal autophagy [8]. BECN1 is also considered a key player in autophagy [51,52], facilitating the PI3K complex formation, leading to multiple membrane trafficking pathways and the initiation of autophagosome formation [126]. BECN1 stimulates the aggregation of cofactors to generate the BECN1–PIK3C3–PIK3R4 complex, which induces the autophagy protein cascade [127]. It is not currently known whether Phafin2 participates in this process. In addition, BECN1 was found to be in complex with VPS34 and AMBRA1 for autophagy induction and that downregulation of AMBRA1 limits the function of BECN1 in the induction complex [128]. LAPTM4B, another member of the Phafin2 protein network, plays a significant role in lysosomal function in autophagy. Lower levels of LAPTM4B cause impairment of the autophagosome–lysosome fusion, decreasing autophagy [129,130].

Other predicted Phafin2-associated proteins are involved in functions outside of autophagy. For example, PIKFYVE participates in endosomal cargo transport, endomembrane homeostasis, and lysosomal trafficking [131,132]. This prediction is in line with early work on Phafin2’s function, in which the protein was associated with cargo trafficking and endosomal and lysosomal function [5,11]. Unrelated protein networks include the prediction of Phafin2’s association to PHF20L1, which interacts with proteins that are mono-methylated in their lysine residues and is crucial for transcriptional repression [133]. However, methylations have not yet been reported on Phafin2. Another predicted Phafin2-interacting protein is CHRNA2, which is linked with hippocampus-dependent learning and memory processing [134,135]. Interestingly, CHRNA2 and Phafin2 are common targets of the androgen receptor upon binding with androgen; however, the receptor represses CHRNA2 expression but upregulates Phafin2 transcription [136]. CCDC24, which is linked to hyperactivity disorder [137], and Kelch-like protein 33, which is associated with neuronal proteins and linked with Mendelian diseases [138], are proteins of unknown function. Nonetheless, except for AKTs, none of the predicted Phafin2-interacting proteins have yet been reported to physically contact Phafin2. Thus, this protein network remains to be confirmed experimentally.

To better understand the presence of the polyD motif in Phafin2, we investigated the protein interaction network of *D. melanogaster* Rush, a protein that contains both PH and FYVE domains but lacks a polyD motif (Figure 7). In common with what was observed for Phafin2, Rush also interacted with autophagic proteins, including autophagic-related 8a (Atg8a; a mammalian LC3 homologous) and Atg8b [139], the PIKYVE human homolog and PtdIns3P-5-kinase formation of aploid and binucleate cells 1 (fab1) [140], and the immune response-deficient 1 (ird1) protein, a serine/threonine kinase [141]. However, unlike Phafin2, Rush displayed a protein interaction network involving GTPases such as the lysosomal RagA-B and RagC-D [142] and GTPase regulators, including a guanine dissociation inhibitor (Gdi) [12], ADP-ribosylation factor GTPase-activating protein 1 (Arf1GAP) [143], and Rho guanine exchange factor 4 (GEF4) [144]. Additionally Rush interacted with CG3918, a poorly characterized nucleic acid binding protein [145]. These Rush interactions suggest that they are independent of the presence of a polyD motif.

## 4. Conclusions

**Figure 8:**
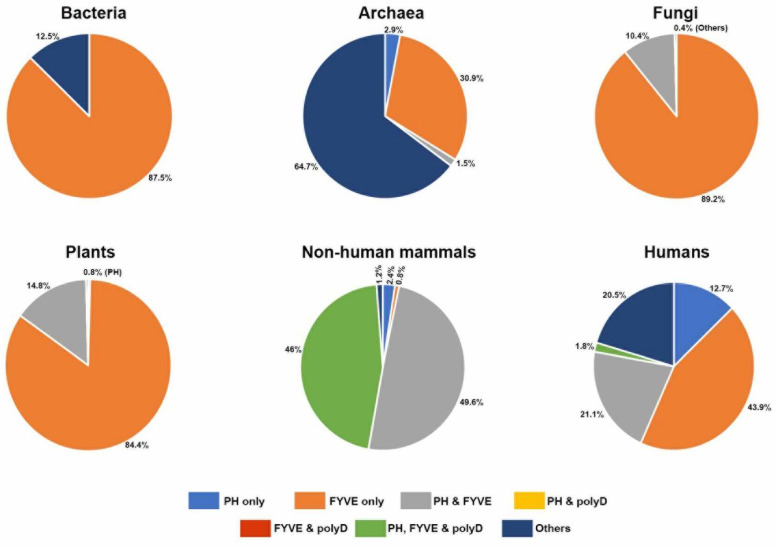
Distribution of the Phafin2 domains and motif in different organisms. Pie representations showing the distribution of individual PH, FYVE, and polyD and combined domains identified from the blastp data search.

Whereas many functions have been characterized for the PH and FYVE domains of Phafin2, there is limited information about the role of the C-terminal polyD motif in the protein. In this bioinformatics analysis, we primarily focused on the evolutionary history and functional lineage of the polyD motif in Phafin2 and other related PH-, FYVE-, or polyD-containing proteins. Interestingly, the polyD motif was absent in the PH-, FYVE-, or both PH-FYVE-containing proteins of bacteria, archaea, fungi, and plants. Moreover, PH-like domains were not found in tandem with FYVE domain-containing proteins in bacteria. Thus, the FYVE domain, which is predominant in bacteria (Figure 8), might be the ancient functional domain of PH-, FYVE-, and polyD-containing proteins. The canonical, and dominant, PH domain emerged for the first time in archaea species and, later, was distributed among different life forms with other protein domains accompanied with a higher frequency of the PH-FYVE pair (Figure 8). The polyD motif is exclusively found in animals and is located at the C-terminus of some PH- and FYVE-containing proteins (Figure 8). However, the functional diversity of Phafin2 and other related PH-, FYVE-, or polyD-containing proteins is more consistent in eukaryotic organisms including in cellular trafficking, autophagy, membrane remodeling, apoptosis, signal transduction, and transcription regulation. Interestingly, human homologs of Phafin2 possess a few additional functional modules (WD, BEACH, Rabosyn-5 repeating NPF sequence-motif, and Sec7), which are only found in eukaryotes. Phafin2 was predicted to interact with AKT1, AKT2, and AKT3, which are known as Phafin2 partners, BECN1, and LAPTM4B, and that all these proteins are linked to autophagy. Other putative Phafin2 interactors, such as PIKFYVE, contribute to endosomal cargo transport, endomembrane homeostasis, and lysosomal trafficking, whereas others, such as CHRNA2, CCDC24, and KLHL33, are associated with neuronal functioning. The Phafin2 homologous protein in *D. melanogaster*, Rush, which lacks a polyD motif, also displays autophagic functions but, unlike Phafin2, cross-talks with GTPases and their regulators. In closing, the polyD motif likely displays a functional association with both the PH and FYVE domains in animal Phafin2 proteins. Although the C-terminal polyD motif of Phafin2 regulates binding of the PH domain to PtdIns3P and provides membrane specificity, it remains unknown, and intriguing, whether this acidic region controls the function of other PH domain-containing proteins.

## Figures and Tables

**Table 1:** Different modules found in fungi and plant PH- and FYVE domain-containing proteins.

Life Forms	UniProt ID	Gene	Organism	Proteins	Modules
Fungi	A0A2Z6RA14_9GLOM	RclHR1_00310010	*Rhizophagus clarus*	Uncharacterized protein	RhoGEF; PH; FYVE
	A0A1Y2IJD5_PYCCO	PYCCODRAFT_557045	*Trametes coccinea*	FYVE domain-containing protein	FYVE
	A0A1Y1YYR8_9FUNG	K493DRAFT_311810	*Basidiobolusmeristosporus*	Uncharacterized protein	PH; FYVE
	A0A2T2PC27_CORCC	BS50DRAFT_567895	*Corynespora cassiicola*	*Vacuolar protein sorting-associated protein 27*	VHS; FYVE; UIM
	A0A507BWX7_9FUNG	SmJEL517_g06146	*Synchytrium microbalum*	*alpha-1,2-Mannosidase*	RhoGEF; Glycosyl hydrolase family 47
	A0A197JU28_9FUNG	K457DRAFT_126461	*Linnemannia elongate*	*Uncharacterized protein*	RhoGEF; FYVE
	F8Q904_SERL3	SERLA73DRAFT_170932	*Serpula lacrymans*	*Uncharacterized protein*	RhoGEF; FYVE
	A0A1Y1UY75_9FUNG	BCR36DRAFT_362273	*Piromyces finnis*	*Ankyrin*	Ankyrin repeats; FYVE; Ring finger
Plants	A0A015IGQ3_RHIIW	RirG_217080	*Rhizophagus irregularis*	*Rom2p*	RhoGEF; PH; FYVE
	A0A177WRZ8_BATDL	BDEG_26098	*Batrachochytrium dendrobatidis*	*Uncharacterized protein*	RhoGEF; PH
	F4P650_BATDJ	BATDEDRAFT_35443	*Batrachochytrium dendrobatidis*	*Uncharacterized protein*	RhoGEF; FYVE
	A0A507EFA4_9FUNG	PhCBS80983_g00823	*Powellomyces hirtus*	*Uncharacterized protein*	RhoGEF; PH; FYVE
	A0A1Y2IJD5_PYCCO	PYCCODRAFT_557045	*Trametes coccinea*	*FYVE domain-containing protein*	FYVE
	A0A6A6HW15_9PLEO	BU26DRAFT_494685	*Trematosphaeria pertusa*	*Vacuolar protein sorting-associated protein 27*	VHS; FYVE; UIM
	A0A6A6V864_9PLEO	M011DRAFT_469719	*Sporormia fimetaria*	*Vacuolar protein sorting-associated protein 27*	FYVE; UIM
	tr|A0A0D7A3M6|A0A0D7A3M6_9AGAR	FISHEDRAFT_67225	*Fistulina fimetaria*	*Uncharacterized protein*	RhoGEF; FYVE
	tr|A0A1Y1UY75|A0A1Y1UY75_9FUNG	BCR36DRAFT_362273	*Piromyces finnis*	*Ankyrin*	Ankyrin repeats; FYVE; Ring finger

**Table 2:** Different modules found in mammalian Phafin2 and PH-, FYVE-, and polyD-containing proteins.

UniProt ID	Gene	Organism	Protein	Modules
G3RXJ3_GORGO	*PLEKHF2*	*Gorilla gorilla*	Pleckstrin homology and FYVE domain-containing 2	PH; FYVE; polyD
F6Q9Z2_MONDO	*NDUFAF6*	*Monodelphis domestica*	Uncharacterized protein	Squalene/phytoene synthase; PH; FYVE, polyD
A0A6J0AU76_VICPA	*PLEKHF1*	*Vicugna pacos*	Pleckstrin homology domain-containing family F member 1	PH; FYVE; polyD
A0A4X2MDN9_VOMUR	*FGD6*	*Vombatus ursinus*	FYVE, RhoGEF and PH domain-containing 6	RhoGEF; PH; FYVE
PKHF2_HUMAN	*PKHF2*	*Homo sapiens*	Pleckstrin homology domain-containing family F member 2	PH; FYVE; polyD
K7ELB8_HUMAN	*PLEKHF1*	*Homo sapiens*	Pleckstrin homology domain-containing family F member 1	PH; FYVE; polyD
A4FVC4_HUMAN	*FGD6*	*Homo sapiens*	FGD6 protein	RhoGEF; PH; FYVE
G3V2D8_HUMAN	*ZFYVE26*	*Homo sapiens*	Zinc finger FYVE domain-containing protein 26	FYVE
RUFY2_HUMAN	*RUFY2*	*Homo sapiens*	RUN and FYVE domain-containing protein 2	RUN; FYVE
ZFYV9_HUMAN	*ZFYVE9*	*Homo sapiens*	Zinc finger FYVE domain-containing protein 9	FYVE; SARA; DUF
FARP1_HUMAN	*FARP1*	*Homo sapiens*	FERM, ARHGEF, and pleckstrin domain-containing protein 1	FERM N-terminal; FERM central; FERM C-terminal PH-like; FERM adjacent (FA); RhoGEF; PH
G5E953_HUMAN	*MTMR3*	*Homo sapiens*	Phosphatidylinositol-3-phosphate phosphatase	Myotubularin-like phosphatase; FYVE
WDFY1_HUMAN	*WDFY1*	*Homo sapiens*	WD repeat and FYVE domain-containing protein 1	WD, G-b repeat; FYVE
A0A7I2YQD1_HUMAN	*HGS*	*Homo sapiens*	Hepatocyte growth factor-regulated tyrosine kinase substrate	VHS; FYVE; hepatocyte growth factor-regulated tyrosine kinase substrate
ANFY1_HUMAN	*ANKFY1*	*Homo sapiens*	Rabankyrin-5	Ankyrin repeats; FYVE
ZFY21_HUMAN	*ZFYVE21*	*Homo sapiens*	Zinc finger FYVE domain-containing protein 21	Zinc finger FYVE-containing protein 21 C-terminus
FYV1_HUMAN	*PIKFYVE*	*Homo sapiens*	1-phosphatidylinositol 3-phosphate 5-kinase	FYVE; DEP; TCP-1/cpn60 chaperonin family; PtdIns4P 5-kinase
A7E293_HUMAN	*WDFY3*	*Homo sapiens*	WDFY3 protein	PH domain associated with BEACH; BEACH; WD; G-b repeat; FYVE
RBNS5_HUMAN	*RBSN*	*Homo sapiens*	Rabenosyn-5	FYVE; Rabenosyn Rab binding; Rabenosyn-5 repeating NPF sequence-motif
CYH4_HUMAN	*CYTH4*	*Homo sapiens*	Cytohesin-4	Sec7; PH

## Data Availability

The data presented in this study are available in the main manuscript and Supplementary Material.

## Supplementary Materials

The following supporting information can be downloaded at: https://www.mdpi.com/article/10.3390/membranes12070696/s1, Table S1. Different modules found in bacterial and archaea PH- and FYVE domain-containing proteins. Table S2: Summary of homologs of human Phafin2. Figures S1–S6: Phafin2-related protein sequence alignments using Jalview; Files S1–S6.
