# Supplementary material for: The PH Domain and C-Terminal polyD Motif of Phafin2 Exhibit a Unique Concurrence in Animals
Source: Membranes (Basel). 2022 Jul 7;12(7):696. doi: 10.3390/membranes12070696 (PMC9324892; doi:10.3390/membranes12070696)
Supplement: Supplementary file 1 [file membranes-12-00696-s001.zip › Supplementary Figures and Tables.pdf]

Supplementary Material

The PH Domain and C-Terminal polyD Motif of Phafin2 Exhibit a Unique Concurrence in Animals

Mahmudul Hasan [1,2,*] and Daniel G. S. Capelluto [1,*]

[1] Protein Signaling Domains Laboratory, Department of Biological Sciences, Fralin Life Sciences Institute and Center for Soft Matter and Biological Physics, Virginia Tech, Blacksburg, VA 24061, USA
[2] Department of Pharmaceuticals and Industrial Biotechnology, Sylhet Agricultural University, Sylhet 3100, Bangladesh
* Correspondence: mahmudulh20@vt.edu (M.H.); capellut@vt.edu (D.G.S.C.)

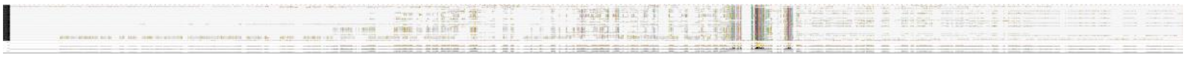

Supplementary Figure S1. JalView illustration of aligned retrieved bacterial protein sequences constructed by Clustal Omega.

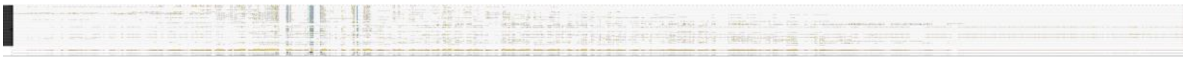

Supplementary Figure S2. JalView illustration of aligned retrieved archaeal protein sequences constructed by Clustal Omega.

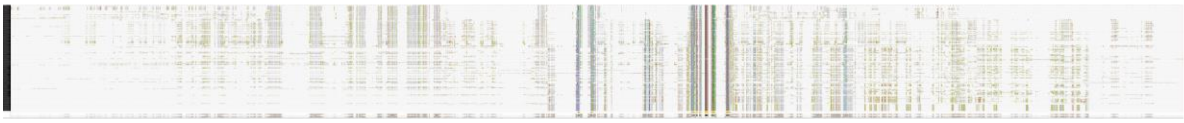

Supplementary Figure S3. JalView illustration of aligned retrieved fungal protein sequences constructed by Clustal Omega.

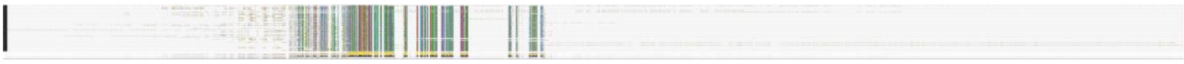

Supplementary Figure S4. JalView illustration of aligned retrieved plant's protein sequences constructed by Clustal Omega.

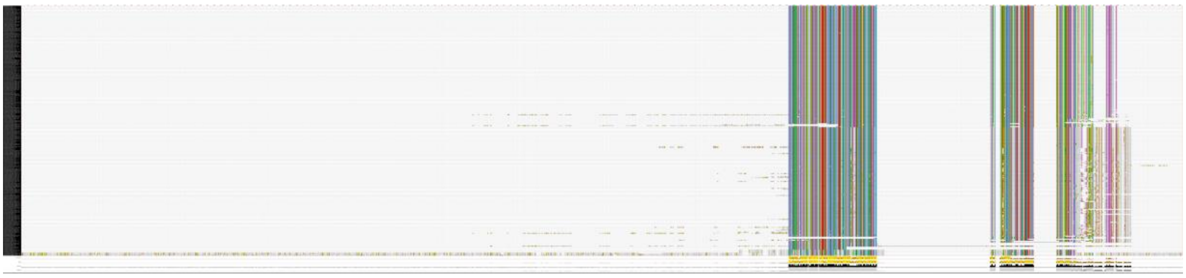

Supplementary Figure S5. JalView illustration of aligned retrieved non-human mammalian protein sequences constructed by Clustal Omega.

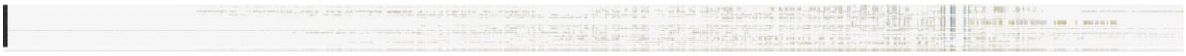

Supplementary Figure S6. JalView illustration of aligned retrieved human's protein sequences constructed by Clustal Omega.

Supplementary Table S1. Different modules found in bacterial and archaea PH- and FYVE domain containing proteins.

Life forms	***UniProt ID***	***Gene***	***Organism***	***Protein***	***Modules***
**Bacteria**	A0A1Y6CWR2_9GAMM	*SAMN02949497_2038*	*Methylomagnum ishizawai*	FYVE zinc finger	FYVE
	A0A2E9XWQ4_9RICK	*CMP47_14530*	*Rickettsiales bacterium*	Uncharacterized protein	VHS; FYVE
	A0A2E4CAT1_9ACTN	*CL450_07530*	*Acidimicrobiaceae bacterium*	Uncharacterized protein	FYVE; Phosphatidylinositol 3- and 4-kinase
	A0A0Q9PCH9_9GAMM	*ASG87_06725*	*Frateuria sp. Soil773*	FYVE-type domain-containing protein	FYVE
	A0A2E8CGN4_9DELT	*CL926_13625*	*Deltaproteobacteria bacterium*	Uncharacterized protein	UBA-like; FYVE
**Archaea**	A0A482RXE8_9ARCH	*EON64_13770*	*Archaeon*	Uncharacterized protein	RhoGEF; FYVE; PH
	A0A482S4E7_9ARCH	*EON65_55335*	*Archaeon*	FYVE-type domain-containing protein	FYVE; FERM
	A0A482SL25_9ARCH	*EON65_20320*	*Archaeon*	Uncharacterized protein	FYVE; WW
	A0A482SVF7_9ARCH	*EON65_02420*	*Archaeon*	FYVE-type domain-containing protein	FYVE
	A0A482SZF2_9ARCH	*EON65_10680*	*Archaeon*	FYVE-type domain-containing protein	Lipase (class 3); FYVE
	A0A482RGE2_9ARCH	*EON67_05280*	*Archaeon*	FYVE-type domain-containing protein	IPT/TIG; FYVE
	A0A482RTU5_9ARCH	*EON64_18385*	*Archaeon*	FYVE-type domain-containing protein	FYVE; Ankyrin repeat
	A0A482SVG8_9ARCH	*EON65_11985*	*Archaeon*	FYVE-type domain-containing protein	FYVE; Tropomyosin

Supplementary Table S2. Summary of orthologues of human Phafin2**.**

Species set	***With 1:1 orthologues***	***With 1:many orthologues***	***With many:many orthologues***	***Without orthologues***
Primates (*26 species*)<br>Humans and other primates	22	1	0	3
Rodents and related species (*32 species*)<br>Rodents, lagomorphs and tree shrews	18	3	0	11
Laurasiatheria (*43 species*)<br>Carnivores, ungulates, and insectivores	31	1	0	11
Placental mammals (*106 species*)	75	5	0	26
Sauropsida (*69 species*)<br>Birds and reptiles	24	0	0	45
Fish (*86 species*)<br>Ray-finned fishes	55	8	0	23
All (*278 species*)<br>All species, including invertebrates	169	13	0	96
