# Supplementary material for: The PH Domain and C-Terminal polyD Motif of Phafin2 Exhibit a Unique Concurrence in Animals
Source: Membranes (Basel). 2022 Jul 7;12(7):696. doi: 10.3390/membranes12070696 (PMC9324892; doi:10.3390/membranes12070696)
Supplement: Supplementary file 1 [file membranes-12-00696-s001.zip › Supplementary File S1.pdf]

Supplementary File S1: Human Phafin2 orthologues retrieved from Ensembl genome browser.

>*Homo_sapiens*(Human)

MVDRLANSEANTRRISIVENCFGAAGQPLTIPGRVLIGEGVLTKLCRKKPKARQFFLFND

ILVYGNIVIQKKKYNKQHIIPLENVTIDSIKDEGDLRNGWLIKTPTKSFAVYAATATEKS

EWMNHINKCVTDLLSKSGKTPSNEHAAVWVPDSEATVCMRCQKAKFTPVNRRHHCRKCGF

VVCGPCSEKRFLLPSQSSKPVRICDFCYDLLSAGDMATCQPARSDSYSQSLKSPLNDMSD

DDDDDDSSD

>*Ficedula_albicollis*(Collared_flycatcher)

MVDRLANSEANTRRISIVENCFGAAGQPLTIPGRVLIGEGVLTKLCRKKPKARQFFLFND

ILVYGNIVIQKKKYNKQHIIPLENVTIDSIQDEGDLRNGWLIKTPTKSFAVYAATATEKS

EWMNHINKCVSDLLSKSGKTPSNEHAAVWVPDSEATVCMRCQKAKFTPVNRRHHCRKCGF

VVCGPCSEKRFLLPSQSSKPVRICDFCYDLLSTGEMTACQSTRSDSYSQSPKSPLNDVSD

DDDDEDSSD

>*Ciona_savignyi*(Pacific_transparent_sea_squirt)

MVERLANTEINARRISHVEACFGSSGKPLFHPTRVLVGEGVLTKLCRKKPKQRQFFLFND

VLVYGNIVISKKRYNKQNVLSLRNVRVEEIPDSDTNGGAEGTEVLKNGWLIISTSKSFAV

YAQTAMEKQEWLTHMNRCIEEEKKKSGPSIDVTEQFAPAWVPDSEAEECMRCKKTRFTAL

QRRHHCRRCGFVVCQGCSSKKVVIKNQSAKPLRVCDTCLTSLANKKDNSPPQNNKLAFGD

TSISDSESSDDTRT

>*Latimeria_chalumnae*(Coelacanths)

MVDRLANSEANSRRIAMVESCFGAAGQPLAIPGRVLIGEGVLTKLCRKKPKARQFFLFND

ILVYGNIVIQKKKYNKQHIIPLENVTIDSIQDEGDLRNGWLIKTPTKSFAVYAATATEKS

EWMNHINKCVADLLMKSGKTPNSEHAAVWVPDSEASVCMRCQRVKFTPVNRRHHCRKCGF

VVCGPCSEKRFLLPSQSSKPVRVCNFCYDLLFSAGPMSASLPSKSDSYRRSPKSSIDNVS

DEDEDEDSSD

>*Petromyzon_marinus*(Sea_lamprey)

MAEPLASSEENCRRIARIESCFGAAGRPLMAPGRVLVGEGTLTKLCRKGPKPRQFFLFDD

MVVYGAAVLGNRRYANQRVLALEAVTICTLPDEGELSNGWLMQTPAKSFKVFAATESERA
EWIGHIERCVRELRAKRGLPPHGLEPAAWWVPDSSASACMRRQRSRFNALNRRHHCRKCG
IVVCGACSDRRFLLPGQSSRPLRVCIDCFDSLAAAAAAAAVTAPAGPLSSSSSDDLDSDE
DDDDDDDGGD

>*Lepisosteus_oculatus*(Spotted_gar)
MVDRLANSEANSKRIALVEGCFGTAGQPLAIPGRVLIGEGVLTKLCRKKPKARQFFLFND
ILVYGNIVIQKKKYNKQHIIPLENVTIDSIEDEGDLRNGWLIKTPTKSFAVYAATATEKS
EWMNHINKCVSDLLEKSGKSPSSEHAAVWVPDSEATVCMRCQKIKFTPVSRRHHCRKCGF
VVCGPCSEKKFLLPSQSSKPVRVCEFCYEQLAGGQAASLPARSDSYSRSSKFPAPSVSDD
DDEDDSSD

>*Myotis_lucifugus*(Little_brown_bat)
MVDRLANSEANTRRINIVENCFGAAGQPLTIPGRVLIGEGVLTKLCRKKPKARQFFLFND
ILVYGNIVIQKKKYNKQHIIPLENVTIDSIKDEGDLRNGWLIKTPTKSFAVYAATATEKS
EWMNHINKCVTDLLSKSGKTPSNEHAAVWVPDSEATVCMRCQKAKFTPVNRRHHCRKCGF
VVCGPCSEKRFLLPSQSSKPVRICDFCYDLLSTGDMASCQPTRSDSYSQSLKSPLNDVSD
DDDDDDSSD

>*Pongo_pygmaeus*(Bornean_orangutan)
MVDRLANSEANTRRISIVENCFGAAGQPLTIPGRVLIGEGVLTKLCRKKPKARQFFLFND
ILVYGNIVIQKKKYNKQHIIPLENVTIDSIKDEGDLRNGWLIKTPTKSFAVYAATATEKS
EWMNHINKCVTDLLSKSGKTPSNEHAAVWVPDSEATVCMRCQKAKFTPVNRRHHCRKCGF
VVCGPCSEKRFLLPSQSSKPVRICDFCYDLLSTGDMATCQPARSDSYSQSLKSPLNDMSD
DDDDDDSSD

>*Dichotomyctere_nigroviridis*(Green_spotted_puffer)
MVDRLANSEANSKRIAFVESCFGAAGQPLAIPGRVLIGEGILTKLCRKKPKTRQFFLFND
ILVYGNILIQKKKYNKQHIIPLEGVTIDTVPDEGELRNGWLIKTPTKSFAVYAATATEKS
EWMNHIGKCVNDLLQKSGKAPTGEHAAVWVPDSEATVCMRCQKVKFTPVSRRHHCRKCGL
VVCGPCSEKKFLLPSQSSKPVRVCEFCYVQLMAGGVAPRSDSMSRPAATFSSSNLCWDEG
SSSSSD

>*Pteropus_vampyrus*(Large_flying_fox)
MVDRLANSEANTRRINIVENCFGAAGQPLTIPGRVLIGEGVLTKLCRKKPKARQFFLFND

ILVYGNIVIQKKKYNKQHIIPLENVTIDSIKDEGDLRNGWLIKTPTKSFAVYAATATEKS
EWMNHINKCVTDLLSKSGKTPSNEHAAVWVPDSEATVCMRCQKAKFTPVNRRHHCRKCGF
VVCGPCSEKRFLLPSQSSKPVRICDFCYDLLSTGDLATCQPTRSDSYSQSLKSPLNDVSD
DDDDDDSSD

>*Loxodonta_africana*(African_bush_elephant)

MVDRLANSEANTRRISIVENCFGAAGQPLTIPGRVLIGEGVLTKLCRKKPKARQFFLFND
ILVYGNIVIQKKKYNKQHIIPLENVTIDSIKDEGDLRNGWLIKTPTKSFAVYAATATEKS
EWMNHINKCVTDLLSKSGKTPSNEHAAVWVPDSEATVCMRCQKAKFTPVNRRHHCRKCGF
VVCGPCSEKRFLLPSQSSKPVRICDFCYDLLSTGDMDRCQPTRSDSYSQSLKSPLNDVSD
DDDDDDSSD

>*Procavia_capensis*(Rock_hyrax)

MVDRLANSEANTRRISIVENCFGAAGQPLTIPGRVLIGEGVLTKLCRKKPKARQFFLFND
ILVYGNIVIQKKKYNKQHIIPLENVTIDSIKDEGDLRNGWLIKTPTKSFAVYAATATEKS
EWMNHINKCVTDLLSKSGKTPSNEHAAVWVPDSEATVCMRCQKAKFTPVNRRHHCRKCGF
VVCGPCSEKRFLLPSQSSKPVRICDFCYDLLSTGDMASRSDSYSQSLKSPLNDVSDDDDD
DDSSD

>*Ciona_intestinalis*(Vase_tunicate)

MVERLANTEINARRISHVESCFGSSGKPLFHPSRVLVGEGVLTKLCRKKPKARQFFLFND
VLVYGNIVINKKRYNKQSVLSLRNVRVEEIPDNEETAAADGSDTLKNGWLIISPSKSFAV
YAQTAMEKEEWLTHMTRCIDEEKKKSGPSISVTDQFAPTWVPDSEAVVCMRCKKTRFTTL
QRKHHCRKCGLVVCNACSTKKCIIQHQSAKPLRVCDVCYQSLQNGSNTPEVTASNVPNNK
LAFGDTSYSSDSESSNEA

>*Gasterosteus_aculeatus*(Three_spined_stickleback)

MVDRLANSEANSKRIAVVEGCFGAAGQPLAIPGRVLIGEGVLTKLCRKKPKARQFFLFND
ILVYGNIMIQKKKYNKQHIIPLESVTIDKVPDEGDLRNGWLIKTPTKSFAVYAATATEKS
EWMNHIGKCVEDLLLKSGKAPTGEHAAVWVPDSEATVCMRCQKVKFTPVSRRHHCRKCGF
VVCGPCSERKHLLPSQSSKPVRVCEFCFVQLTAEGAAARSDSLGRLGSKFHNMSDDDDDD
DSSD

>*Tursiops_truncatus*(Bottlenose_dolphins)

MVDRLANSEANTRRINIVENCFGAAGQPLTIPGRVLIGEGVLTKLCRKKPKARQFFLFND

ILVYGNIVIQKKKYNKQHIIPLENVTIDSIKDEGDLRNGWLIKTPTKSFAVYAATATEKS
EWMNHINKCVTDLLSKSGKTPSNEHAAVWVPDSEATVCMRCQKAKFTPVNRRHHCRKCGF
VVCGPCSEKRFLLPSQSSKPVRICDFCYDLLSTGDMATCQPTRSDSYSQSLKSTLNDVSD
DDDDDDSSD

>*Poecilia_formosa*(Amazon_molly)
MVDRLANSEANSKRIGLVESCFGAAGQPLAIPGRVLIGEGVLTKLCRKKPKARQFFLFND
ILVYGNIVIQKKKYNKQHIIPLEGVTIDTVPDEGELRNGWLIKTPTKSFAVYAATATEKS
EWMNHIGKCVRELLQKSGKSPTGEHAAVWVPDSEATVCMRCKKVKFTPVSRRHHCRKCGY
VVCGPCSEKKFLLPSQSSKPVRVCEHCYGMLSGSLGPRSESMSRPGANFNNNTLSDDDDD
DDSSD

>*Mustela_putorius*(Ferret)
MVDRLANSEANTRRINIVENCFGAAGQPLTIPGRVLIGEGVLTKLCRKKPKARQFFLFND
ILVYGNIVIQKKKYNKQHIIPLENVTIDSIKDEGDLRNGWLIKTPTKSFAVYAATATEKS
EWMNHINKCVTDLLSKSGKTPSNEHAAVWVPDSEATVCMRCQKAKFTPVNRRHHCRKCGF
VVCGPCSEKRFLLPSQSSKPVRICDFCYDLLSTGDMATCQPTRSDSYSQSLKSPLNDVSD
DDDDDDSSD

>*Otolemur_garnettii*(Northern_greater_galago)
MVDRLANSEANTRRISIVENCFGAAGQPLTIPGRVLIGEGVLTKLCRKKPKARQFFLFND
ILVYGNIVIQKKKYNKQHIIPLENVTIDSIKDEGDLRNGWLIKTPTKSFAVYAATATEKS
EWMNHINKCVTDLLSKSGKTPSNEHAAVWVPDSEATVCMRCQKAKFTPVNRRHHCRKCGF
VVCGPCSEKRFLLPSQSSKPVRICDFCYDLLSTGDLATCQPTRSDSYGQSLKSSLNDVSD
DDDDDDSSD

>*Chlorocebus_sabaeus(*Green_monkey)
MVDRLANSEANTRRISIVENCFGAAGQPLTIPGRVLIGEGVLTKLCRKKPKARQFFLFND
ILVYGNIVIQKKNYNKQHIIPLENVTIDSIKDEGDLRNGWLIKTPTKSFAVYAATATEKS
EWMNHINKCVTDLLSKSGKTPSNEHAAVWVPDSEATVCMRCQKAKFTPVNRRHHCRKCGF
VVCGPCSEKRFLLPSQSSKPVRICDFCYDLLSTGDMATCQPARSDSYSQSLKSPLNDMSD
DDDDDDSSD

>*Dasypus_novemcinctus*(Nine_banded_armadillo)
MVDRLANSEANTRRISIVENCFGAAGQPLTIPGRVLIGEGVLTKLCRKKPKARQFFLFND

ILVYGNIVIQKKKYNKQHIIPLENVTIDSIKDEGDLRNGWLIKTPTKSFAVYAATATEKS
EWMNHINKCVTDLLSKSGKTPSNEHAAVWVPDSEATVCMRCQKAKFTPVNRRHHCRKCGF
VVCGPCSEKRFLLPSQSSKPVRICDFCYDLLSTGDMAACQPNRSDSYSHSLKSPLNDVSD
DDDDDDSSD
>*Tupaia_belangeri*(Northern_treeshrew)
MVDRLANSEANTRRISIVENCFGAAGQPLTIPGRVLIGEGVLTKLCRKKPKARQFFLFND
ILVYGNIVIQKKKYNKQHIIPLENVTIDSIKDEGDLRNGWLIKTPTKSFAVYAATATEKS
EWMNHINKCVTDLLSKSGKTPSNEHAAVWVPDSEATVCMRCQKAKFTPVNRRHHCRKCGF
VVCGPCSEKRFLLPSQSSKPVRICDFCYDLLSTGDMATCQQPTRSDSYSQSLKSPLNDVS
DDDDDDDSSD
>*Anolis_carolinensis*(Carolina_anole)
MVDRLANSEANTRRISIVENCFGAAGQPLTIPGRVLIGEGVLTKLCRKKPKARQFFLFND
ILVYGNIVIQKKKYNKQHIIPLENVTIESIQDEGDLRNGWLIKTPTKSFAVYAATATEKS
EWMNHINKCVSDLLSKSGKTPSNEHAAVWVPDSEATICMRCQKAKFTPVNRRHHCRKCGF
VVCGPCSEKRFLIPSQSSKPVRICDFCYDLLSTGEMATCQSGRSDSYSPSLKPPLNDISD
DDDDDDSSD
>*Pelodiscus_sinensis*(Chinese_softshell_turtle)
MVDRLANSEANTRRISIVENCFGAAGQPLTIPGRVLIGEGVLTKLCRKKPKARQFFLFND
ILVYGNIVIQKKKYNKQHIIPLENVTIDSIQDEGDLRNGWLIKTPTKSFAVYAATATEKS
EWMSHINKCVSDLLSKSGKTPSNEHAAVWVPDSEATVCMRCQKAKFTPVNRRHHCRKCGF
VVCGPCSEKRFLLPNQSSKPVRICDFCYDLLSTGEMTACQPTRSDSYSQSPKSPINDVSD
DDDDDDSSD
>*Ochotona_princeps*(American_pika)
MVDRLANSEANTRRISIVENCFGAAGQPLTIPGRVLIGEGVLTKLCRKKPKARQFFLFND
ILVYGNIVIQKKKYNKQHIIPLENVTIDSIKDEGDLRNGWLIKTPTKSFAVYAATATEKS
EWMSHINKCVTDLLSKSGKTPSNEHAAVWVPDSEATVCMRCQKAKFTPVNRRHHCRKCGF
VVCGPCSEKRFLLPSQSSKPVRICDFCYDLLSTGDMATCQPARSDSYSQSLKSPLNDVSD
DDDDDDSSD
>*Erinaceus_europaeus*(European_hedgehog)
MVDRLANSEANTRRINIVENCFGAAGQPLTIPGRVLIGEGVLTKLCRKKPKARQFFLFND

ILVYGNIVIQKKKYNKQHIIPLENVTIDSIKDEGDLRNGWLIKTPTKSFAVYAATATEKS
EWMNHINKCVTDLLSKSGKTPSNEHAAVWVPDSEATVCMRCQKAKFTPVNRRHHCRKCGF
VVCGPCSEKRFLLPSQSSKPVRICDFCYDLLSTGDMATCQPARSDSYSQSLKSPLNDVSD
DDDDDDSSD

>*Vicugna_pacos*(Alpaca)

MVDRLANSEANTRRINIVENCFGAAGQPLTIPGRVLIGEGVLTKLCRKKPKARQFFLFND
ILVYGNIVIQKKKYNKQHIIPLENVTIDSIKDEGDLRNGWLIKTPTKSFAVYAATATEKS
EWMNHINKCVTDLLSKSGKTPSNEHAAVWVPDSEATVCMRCQKAKFTPVNRRHHCRKCGF
VVCGPCSEKRFLLPSQSSKPVRICDFCYDLLSAGDMATCQPARSDSYSQSLKSPLNDVSD
DDDDDDSSD

>*Sorex_araneus*(Common_shrew)

MVDRLANSEANTRRINIVENCFGAAGQPLTIPGRVLIGEGVLTKLCRKKPKARQFFLFND
ILVYGNIVIQKKKYNKQHIIPLENVTIDSIKDEGDLRNGWLIKTPTKSFAVYAATATEKS
EWMNHINKCVTDLLSKSGKTPSNEHAAVWVPDSEATVCMRCQKAKFTPVSRRHHCRKCGF
VVCGSCSEKRFLLPSQSSKPVRICDFCYDLLSTGDSGLGQPTRSDSYSQSLKSPLNDASD
DDDDDDSSD

>*Choloepus_hoffmanni*(Hoffmann's_two-toed_sloth)

MVDRLANSEANTRRINIVENCFGAAGQPLTIPGRVLIGEGVLTKLCRKKPKARQFFLFND
ILVYGNIVIQKKKYNKQHIIPLENVTIDSIKDEGDLRNGWLIKTPTKSFAVYAATATEKS
EWMNHINKCVTDLLSKSGKTPSNEHA

>*Macropus_eugenii*(Tammar_wallaby)

MVDRLANSEANTRRISIVENCFGAAGQPLTIPGRVLIGEGVLTKLCRKKPKARQFFLFND
ILVYGNIVIQKKKYNKQHIIPLENVTIDSIKDEGDLRNGWLIKTPTKSFAVYAATATEKS
EWMNHINKCVTDLLSKSGKTPSNEHAAVWVPDSEATVCMRCQKAKFTPVNRRHHCRKCGF
VVCGPCSEKRFLLPSQSSKPVRICDFCFDLLSSGDLATCQPSRSDSYSQSPKSPLNNVSD
DEDDEDSSD

>*Heterocephalus_glaber_1_*(Naked_mole-rat)

MVDRLANSEANTRRIGIVENCFGAAGQPLTIPGRVLIGEGVLTKLCRKKPKARQFFLFND
ILVYGNIVIQKKKYNKQHIIPLENVTIDSIKDEGDLRNGWLIKTPTKSFAVYAATATEKS
EWMNHINKCVTDLLSKSGKTPVNEHAAVWVPDSEATVCMRCQKAKFTPVNRRHHCRKCGY

VVCGPCSEKRFLLPSQSSKPVRICDFCYDLLSTGDMATCQPGRSDSYSQSLKSSLNNVSD
DEDDDSSD
>*Heterocephalus_glaber_2_*(Naked_mole-rat)
MDRLTNSEANTRRIGIVESCFGAANQPLTIPGRVLIGEGPKARQFFLFNDILVNGNIVVQ
EKKYNKQHIIPLQNVTIDSVKDEGDLRNGWLIKTPTKLFAVYAATATEKSEWMNHINKCV
TDLLSKSGKTPINEHTAVWVPDSEATVCMRCQKAKFTPVNPLHHCRKCGHVVCGPCSEKR
FLLPSQSSKPVRICDFCYDLLSTGDMATCQPARSDSVYSQSLKSSLNNVSDDEDDDSSA
>*Chinchilla_lanigera*(Long-tailed_chinchilla)
MVDRLANSEANTRRIGIVENCFGAAGQPLTIPGRVLIGEGVLTKLCRKKPKARQFFLFND
ILVYGNIVIQKKKYNKQHIIPLENVTIDSIKDEGDLRNGWLIKTPTKSFAVYAATATEKS
EWMNHINKCVTDLLSKSGKTPINEHAAVWVPDSEATVCMRCQKAKFTPVNRRHHCRKCGF
VVCGPCSEKRFLLPSQSSKPVRICDFCYDLLSTGDMAACQPARSDSYSQSLKSPLNDVSD
DDDDDSSD
>*Cavia_porcellus*(Guinea_pig)
MVDRLANSEANTRRIGIVENCFGAAGQPLTIPGRVLIGEGVLTKLCRKKPKARQFFLFND
ILVYGNIVIQKKKYNKQHIIPLENVTIDSIKDEGDLRNGWLIKTPTKSFAVYAATATEKS
EWMNHINKCVTDLLSKSGKTPINEHAAVWVPDSEATVCMRCQKAKFTPVNRRHHCRKCGF
VVCGPCSEKRFLLPSQSSKPVRICDFCYDLLSTGDMATCQPARSDSYSQSLKSPLNNVSD
DDDDDSSD
>*Ictidomys_tridecemlineatus*(Thirteen-lined_ground_squirre)
MVDRLANSEANTRRISIVENCFGAAGQPLTIPGRVLIGEGVLTKLCRKKPKARQFFLFND
ILVYGNIVIQKKKYNKQHIIPLENVTIDSIKDEGDLRNGWLIKTPTKSFAVYAATATEKS
EWMNHINKCVTDLLSKSGKTPSNEHAAVWVPDSEATVCMRCQKAKFTPVNRRHHCRKCGF
VVCGPCSEKRFLLPSQSSKPVRICDFCYDLLSTGDMATCQPTRSDSYSQSLKSPLNDVSD
DDDDDDSSD
>*Heterocephalus_glaber*(Naked_mole-rat)
MVDRLANSEANTRRIGIVENFVIGEGVLTKLCRKKPKARQFFLFNDIFVYGNIVIQKKKY
NKQHIIPLENVTIESIKDERDLRNGWLIKTPTKSFAVYAATATEKSEWMNRINKCVTDLL
SKSGKTPINERAAVWVPDSEAPVCMRCQKAKFTPVSRRHHCRKCGYVVCGPCSEKRFLLP
RQSSKPVRICDFCYDLLSTGDMAACQPARSDSIYSQSLKSSLNNVSDDEDDDSSD

>*Rattus_norvegicus*(Brown_rat)

MVDRLANSEANTRRISIVESCFGAAGQPLTIPGRVLIGEGVLTKLCRKKPKARQFFLFND
ILVYGNIVIQKKKYNKQHIIPLENVTIDSIKDEGELRNGWLIKTPTKSFAVYAATATEKS
EWMNHINKCVSDLLSKSGKTPSNEHAAVWVPDSEATVCMRCQKAKFTPVNRRHHCRKCGF
VVCGPCSEKRFLLPSQSSKPVRICDFCYDLLSTGDMAACQPTRSDSYSQSLKSPLNDASD
DDDDDDSSD

>*Clethrionomys_glareolus*(Vole)

MVDRLANSEANSRRISIVESCFGAAGQPLTIPGRVLIGEGVLTKLCRKKPKARQFFLFND
ILVYGNIVIQKKKYNKQHIIPLENVTIDSIKDEGELRNGWLIKTPTKSFAVYAATATEKS
EWMSHINKCVTDLLSKSGKTPSSEHAAVWVPDSEATVCMRCQKAKFTPVNRRHHCRKCGF
VVCGPCSEKRFLLPSQSSKPVRICDFCYELLSTGDMAACQPARSDSYSQSLKPPLNDVSD
DDDDDDSSD

>*Cricetulus_griseus*(Chinese_hamster)

MVDRLANSEANTRRISIVENCFGAAGQPLTIPGRVLIGEGVLTKLCRKKPKARQFFLFND
ILVYGNIVIQKKKYNKQHIIPLENVTIDSIKDEGELRNGWLIKTPTKSFAVYAATATEKS
EWMNHINKCVTDLLSKSGKTPSSEHAAVWVPDSEATVCMRCQKAKFTPVNRRHHCRKCGF
VVCGPCSEKRFLLPNQSSKPVRICDFCYDLLSTGDMSSGQPTRSDSYSQSLKSPLNDVSD
DDDDDDSSD

>*Cercocebus_atys*(Sooty_mangabey)

MVDRLANSEANTRRISIVENCFGAAGQPLTIPGRVLIGEGVLTKLCRKKPKARQFFLFND
ILVYGNIVIQKKKYNKQHIIPLENVTIDSIKDEGDLRNGWLIKTPTKSFAVYAATATEKS
EWMNHINKCVTDLLSKSGKTPSNEHAAVWVPDSEATVCMRCQKAKFTPVNRRHHCRKCGF
VVCGPCSEKRFLLPSQSSKPVRICDFCYDLLSTGDMATCQPARSDSYSQSLKSPLNDMSD
DDDDDDSSD

>*Cebus_imitator(*White-faced_capuchin)

MVDRLANSEANTRRISIVENCFGAAGQPLTIPGRVLIGEGVLTKLCRKKPKARQFFLFND
ILVYGNIVIQKKKYNKQHIIPLENVTIDSIKDEGDLRNGWLIKTPTKSFAVYAATATEKS
EWMNHINKCVTDLLSKSGKTPSNEHAAVWVPDSEATVCMRCQKAKFTPVNRRHHCRKCGF
VVCGPCSEKRFLLPSQSSKPVRICDFCYDLLSTGDMATCQPARSDSYSQSLKSPLNDMSD
DDDDDDSSD

>*Macaca_nemestrina*(Southern_pig-tailed_macaque)

MVDRLANSEANTRRISIVENCFGAAGQPLTIPGRVLIGEGVLTKLCRKKPKARQFFLFND
ILVYGNIVIQKKKYNKQHIIPLENVTIDSIKDEGDLRNGWLIKTPTKSFAVYAATATEKS
EWMNHINKCVTDLLSKSGKTPSNEHAAVWVPDSEATVCMRCQKAKFTPVNRRHHCRKCGF
VVCGPCSEKRFLLPSQSSKPVRICDFCYDLLSTGDMATCQPARSDSYSQSLKSPLNDMSD
DDDDDDSSD

>*Nomascus_leucogenys*(Northern_white-cheeked_gibbon)

MVDRLANSEANTRRISIVENCFGAAGQPLTIPGRVLIGEGVLTKLCRKKPKARQFFLFND
ILVYGNIVIQKKKYNKQHIIPLENVTIDSIKDEGDLRNGWLIKTPTKSFAVYAATATEKS
EWMNHINKCVTDLLSKSGKTPSNEHAAVWVPDSEATVCMRCQKAKFTPVNRRHHCRKCGF
VVCGPCSEKRFLLPSQSSKPVRICDFCYDLLSTGDMATCQPARSDSYSQSLKSPLNDMSD
DDDDDDSSD

>*Capra_aegagrus*(Goat)

MVDRLANSEANTRRINIVENCFGAAGQPLTIPGRVLIGEGVLTKLCRKKPKARQFFLFND
ILVYGNIVIQKKKYNKQHIIPLENVTIDSIKDEGDLRNGWLIKTPTKSFAVYAATATEKS
EWMNHINKCVTDLLSKSGKTPSNEHAAVWVPDSEATVCMRCQKAKFTPVNRRHHCRKCGF
VVCGPCSEKRFLLPSQSSKPVRICDFCYDLLSTGDVAACQPARSDSYSQSLQPPLNDVSD
DDDDDDSSD

>*Sorex_araneus*(Shrews)

MVDRLANSEANTRRISIVENCFGAAGQPLTIPGRVLIGEGVLTKLCRKKPKARQFFLFND
ILVYGNIVIQKKKYNKQHIIPLENVTIDSIKDEGELRNGWLIKTPTKSFAVYAATATEKS
EWMNHINKCVTDLLSKSGKTPSNEHAAVWVPDSEATVCMRCQKAKFTPVNRRHHCRKCGF
VVCGPCSEKRFLLPSQSSKPVRICDFCYDLLSTGDMAACQPTRSDSYSQSLKSPLNDASD
DDDDDDSSD

>*Mus_spretus*(Algerian_mouse)

MVDRLANSEANTRRISIVENCFGAAGQPLTIPGRVLIGEGVLTKLCRKKPKARQFFLFND
ILVYGNIVIQKKKYNKQHIIPLENVTIDSIKDEGELRNGWLIKTPTKSFAVYAATATEKS
EWMNHINKCVTDLLSKSGKTPSNEHAAVWVPDSEATVCMRCQKAKFTPVNRRHHCRKCGF
VVCGPCSEKRFLLPSQSSKPVRICDFCYDLLSTGDMAACQPTRSDSYSQSLKSPLNDASD
DDDDDDSSD

>*Mus_caroli(*Ryukyu_mouse*)*

MVDRLANSEANTRRISIVENCFGAAGQPLTIPGRVLIGEGVLTKLCRKKPKARQFFLFND

ILVYGNIVIQKKKYNKQHIIPLENVTIDSIKDEGELRNGWLIKTPTKSFAVYAATATEKS

EWMNHINKCVTDLLSKSGKTPSNEHAAVWVPDSEATVCMRCQKAKFTPVNRRHHCRKCGF

VVCGPCSEKRFLLPSQSSKPVRICDFCYDLLSTGDMAACQPTRSDSYSQSLKSPLNDASD

DDDDDDSSD

>*Eptatretus_burgeri*(Inshore_hagfish)

MVDRLVNSEGNSRRIAAVESCFGAAGQPLAAPGRVLVGEGTLTKQCRKGPKPRQFFLFND

LLVYGAVVLSRRRYAGQRIIPLEQVSIRAVPDEGDLHNGWLIQTPAKSFKVFAATALERT

EWMSHIERCVRDQLDRKRRDKSTVDEPAAVWVPDASASTCMRCHRTRFTALNRRHHCRKC

GFVVCAACSEHRHLIPQQSSRPLRVCQPCYEDLSANSHNSSHNTSRRGSDGPVPCPGGLD

SSDEDSSDSGEEIGSSLPGYTTDFYS

>*Panthera_tigris*(Tiger)

MVDRLANSEANTRRINIVENCFGAAGQPLTIPGRVLIGEGVLTKLCRKKPKARQFFLFND

ILVYGNIVIQKKKYNKQHIIPLENVTIDSIKDEGDLRNGWLIKTPTKSFAVYAATATEKS

EWMNHINKCVTDLLSKSGKTPSNEHAAVWVPDSEATVCMRCQKAKFTPVNRRHHCRKCGF

VVCGPCSEKRFLLPSQSSKPVRICDFCYDLLSTGDMATCQPTRSDSYSQSLKSPLNDVSD

DDDDDDSSD

>*Danio_rerio*(Zebrafish)

MVDRLANSEANSKRIGVVEACFGTAGQPLAIPGRVLIGEGVLTKLCRKRPKARQFFLFND

ILVYGNIVIQKKKYNKQHIIPLESVTIDTVEDEGELRNGWLIKTPTKSFAVYAATATEKS

EWMSHINKCVSDLLEKSGKSPTGEHAAVWVPDSEATVCMRCQKMKFTPVNRRHHCRKCGF

VVCGPCSEKKFLLPSQSSKPVRVCEFCYKQLSTGATLPPRSDSYSRQGSDFGSNNISDDD

DDDDSSD

>*Cyprinodon_variegatus*(Sheepshead_minnow)

MVDRLANSEANSKRIGLVESCFGAAGQPLAIPGRVLIGEGVLTKLCRKKPKARQFFLFND

ILVYGNIVIQKKKYNKQHIIPLESVTIDTVPDEGELRNGWLIKTPTKSFAVYAATATEKS

EWMNHIGKCVRELLQKSGKSPTGEHAAVWVPDSEATVCMRCKKVKFTAVSRRHHCRKCGY

VVCGPCSDKKFLLPSQSSKPVRVCEHCYAMLSGSLGPRSDSITRPGANFNNNTLSDDEDE

DDSSD

>*Amphilophus_citrinellus*(Midas_cichlid)

MVDRLANSEANSKRIALVENCFGAAGQPLAIPGRVLIGEGVLTKLCRKKPKARQFFLFND
ILVYGNIVIQKKKYNKQHIIPLESVTIDTVPDEGDLRNGWLIKTPTKSFAVYAATATEKS
EWMNHIGKCVGDLLQKSGKSPTGEHAAVWVPDSEASVCMRCKKVKFTPVSRRHHCRKCGF
VVCGPCSEKKYLLPSQSSKPVRVCEYCYEQLTSVNLQQHSDTITRPGSKFNSSNLSDDDD
DDDSSD

>*Oryzias_dancena*(Indian_medaka)

MQPDDLDTLQSVIFEYEREIYLSNIKKEPEQCEVKMVDRLANSEANSKRIGMVESCFGAA
GQPLAIPGRVLIGEGVLTKLCRKKPKARQFFLFNDILVYGNIVIQKKKYNKQHIIPLESV
TIDTVPDEGDLRNGWLIKTPTKSFAVYAATGTEKCEWMNHIGKCVRDLLQKSGKSPTGEH
AAVWVPDSEAPICMRCEKVKFTPVIRRHHCRKCGYVVCGPCSEKKYLIPSQSSKPVRVCE
YCYSQLTSGSHAPRSDSNNWGESRYNNNHLSDDEDEDDSSD

>*Stegastes_partitus*(Bicolor_damselfish)

MVDRLANSEANSKRIAVVEGCFGAAGQPLAIPGRVLIGEGVLTKLCRKKPKARQFFLFND
ILVYGNIVIQKKKYNKQHIIPLESVTIDTVPDEGDLRNGWLIKTPTKSFAVYAATATEKS
EWMNHIGKCVGDLLQKSGKSPTGEHAAVWVPDSEASVCMRCQKVKFTPVSRRHHCRKCGF
VVCGPCSEKKYLLPSQSSKPVRVCEYCYVQLTSGSLAPRSDSISRSKFNSNNLSDDEDED
DSSD

>*Amphiprion_percula*(Orange_clownfish)

MVDRLANSEANSKRIAVVESCFGAAGQPLAIPGRVLIGEGVLTKLCRKKPKARQFFLFND
ILVYGNIVIQKKKYNKQHIIPLESVTIDTVPDEGDLRNGWLIKTPTKSFAVYAATATEKS
EWMNHIGKCVGDLLQKSGKSPTGEHAAVWVPDSEASVCMRCQKVKFTPVSRRHHCRKCGF
VVCGPCSEKKYLLPSQSSKPVRVCEYCYLQLTSGSLGPRSDSISRPGSKFNSNNLSDDED
EDDSSD

>*Fundulus_heteroclitus*(Mummichog)

MVDRLANSEANSKRIGLVESCFGAAGQPLAIPGRVLIGEGVLTKLCRKKPKARQFFLFND
ILVYGNIVIQKKKYNKQHIIPLESVTIDTVQDEGELRNGWLIKTPTKSFAVYAATATEKS
EWMNHIGKCVRELLQKSGKSPTGEHAAVWVPDSEATVCMRCKKVKFTPVSRRHHCRKCGY
VVCGPCSEKKFLLPSQSSKPVRVCEHCYGVLSGSLGPNADSLSRPGLNFNNTLSDDDDDD
DSSD

>*Maylandia_zebra*(Zebra_mbuna)

MVDRLANSEANSKRIAVVESCFGAAGQPLAIPGRVLIGEGVLTKLCRKKPKARQFFLFND

ILVYGNIVIQKKKYNKQHIIPLESVTIDTVPDEGDLRNGWLIKTPTKSFAVYAATATEKS

EWMNHIGKCVGDLLQKSGKSPTGEHAAVWVPDSEASVCMRCKKVKFTPVSRRHHCRKCGF

VVCGPCSEKKYLLPSQSSKPVRVCEHCYEQLTSVNQARSDSITRPGSKFHSNNLSDDDDD

DDSSD

>*Astatotilapia_calliptera*(Eastern_happy)

MVDRLANSEANSKRIAVVESCFGAAGQPLAIPGRVLIGEGVLTKLCRKKPKARQFFLFND

ILVYGNIVIQKKKYNKQHIIPLESVTIDTVPDEGDLRNGWLIKTPTKSFAVYAATATEKS

EWMNHIGKCVGDLLQKSGKSPTGEHAAVWVPDSEASVCMRCKKVKFTPVSRRHHCRKCGF

VVCGPCSEKKYLLPSQSSKPVRVCEHCYEQLTSVNQARSDSITRPGSKFHSNNLSDDDDD

DDSSD

>*Oryzias_latipes*(Japanese_rice_fish)

MVDRLANSEANSKRIGMVESCFGAAGQPLAIPGRVLIGEGVLTKLCRKKPKARQFFLFND

ILVYGNIVIQKKKYNKQHIIPLESVTIDTVPDEGDLRNGWLIKTPTKSFAVYAATATEKC

EWMNHIGKCVKDLLQKSGKSPTGEHAAVWVPDSEASVCMRCLKVKFTAVNRRHHCRKCGY

VVCNPCSEKKYLLPSQSSKPVRVCEFCYAQLTSGSLAPCSDSNSWSESRYNNNHLSDDED

EDDSSD

>*Amphiprion_ocellaris*(Clownfish)

MVDRLANSEANSKRIAVVESCFGAAGQPLAIPGRVLIGEGVLTKLCRKKPKARQFFLFND

ILVYGNIVIQKKKYNKQHIIPLESVTIDTVPDEGDLRNGWLIKTPTKSFAVYAATATEKS

EWMNHIGKCVGDLLQKSGKSPTGEHAAVWVPDSEASVCMRCQKVKFTPVSRRHHCRKCGF

VVCGPCSEKKYLLPSQSSKPVRVCEYCYLQLTSGSLGPRSDSISRPGSKFNSNNLSDDED

EDDSSD

>*Xiphophorus_maculatus*(Southern_platyfish)

MVDRLANSEANSKRIGLVESCFGAAGQPLAIPGRVLIGEGVLTKLCRKKPKARQFFLFND

ILVYGNIVIQKKKYNKQHIIPLEGVTIDTVPDDGELRNGWLIKTPTKSFAVYAATATEKS

EWMNHIGKCVRELLQKSGKSPTGEHAAVWVPDSEATVCMRCKKVKFTPVSRRHHCRKCGY

VVCGPCSEKKFLLPSQSSKPVRVCEHCYGMLSGSLGRQSESMSRSGANFNNTLSDDDDDD

DSSD

>*Neolamprologus_brichardi(*Lyretail_cichlid)

MVDRLANSEANSKRIAVVESCFGAAGQPLAIPGRVLIGEGVLTKLCRKKPKARQFFLFND

ILVYGNIVIQKKKYNKQHIIPLESVTIDTVPDEGDLRNGWLIKTPTKSFAVYAATATEKS

EWMNHIGKCVGDLLQKSGKSPTGEHAAVWVPDSEASVCMRCKKVKFTPVSRRHHCRKCGY

VVCGPCSEKKYLLPSQSSKPVRVCEHCYDQLTSVNQARSDSITRPGSKFHSNNLSDDDDD

DDSSD

>*Scophthalmus_maximus*(Turbot)

MVDQLAKSEANSKRIAVVEGCFGAAGQPLAIPGRVLIGEGVLTKLCRKKPKARQFFLFND

ILVYGNIVIQKKKYNKQHIIPLESVTIDTVPDEGDLRNGWLIKTPTKSFAVYAATATEKS

EWMNHIGKCVGDLLQKSGKAPTGEHAAVWVPDSEATVCMRCQKVKFTPVSRRHHCRKCGY

VVCGPCSEKKYLLPSQSSKPVRVCEYCFVQLTSGGLAVRSDSFGRMGSKFNNLSDDDDED

DSSD

>*Pundamilia_nyererei*(Makobe_Island_cichlid)

MVDRLANSEANSKRIAVVESCFGAAGQPLAIPGRVLIGEGVLTKLCRKKPKARQFFLFND

ILVYGNIVIQKKKYNKQHIIPLESVTIDTVPDEGDLRNGWLIKTPTKSFAVYAATATEKS

EWMNHIGKCVGDLLQKSGKSPTGEHAAVWVPDSEASVCMRCKKVKFTPVSRRHHCRKCGF

VVCGPCSEKKYLLPSQSSKPVRVCEHCYEQLTSVNQARSDSITRPGSKFHSNNLSDDDDD

DDSSD

>*Vulpes_vulpes*(Red_fox)

MVDRLANSEANTRRINIVENCFGAAGQPLTIPGRVLIGEGVLTKLCRKKPKARQFFLFND

ILVYGNIVIQKKKYNKQHIIPLENVTIDSIKDEGDLRNGWLIKTPTKSFAVYAATATEKS

EWMNHINKCVTDLLSKSGKTPSNEHAAVWVPDSEATVCMRCQKAKFTPVNRRHHCRKCGF

VVCGPCSEKRFLLPSQSSKPVRICDFCYDLLSTGDMATCQPARSDSYSQSLKSPLNDVSD

DDDDDDSSD

>*Gallus_gallus*(Chicken)

MVDRLANSEANTRRISIVENCFGAAGQPLTIPGRVLIGEGVLTKLCRKKPKARQFFLFND

ILVYGNIVIQKKKYNKQHIIPLENVTIDSIQDEGDLRNGWLIKTPTKSFAVYAATATEKS

EWMNHINKCVSDLLSKSGKTPSNEHAAVWVPDSEATVCMRCQKAKFTPVNRRHHCRKCGF

VVCGPCSEKRFLLPSQSSKPVRICDFCYDLLSTGEMTACQSTRSDSYSQSPKSSLNDASD

DDDDEDSSD

>*Sphenodon_punctatus*(Tuatara)

MVDRLANSEANTRRISIVENCFGAAGQPLTIPGRVLIGEGVLTKLCRKKPKARQFFLFND

ILVYGNIVIQKKKYNKQHIIPLENVTIESIQDEGDLRNGWLIKTPTKSFAVYAATATEKS

EWMNHINKCVSDLLSKSGKTPSNEHAAVWVPDSEASVCMRCQKAKFTPVNRRHHCRKCGF

VVCGPCSEKRFLLPSQASKPVRICDFCYDLLSSGEMTTCLPTRSDSYSQSPKSPLNNVSD

DDDDDDSSD

>*Equus_asinus*(Donkey)

MVDRLANSEANTRRINIVEHCFGAAGQPLTIPGRVLIGEGVLTKLCRKKPKARQFFLFND

ILVYGNIVIQKKKYNKQHIIPLENVTIDSIKDEGDLRNGWLIKTPTKSFAVYAATATEKS

EWMNHINKCVTDLLSKSGKTPSNEHAAVWVPDSEATVCMRCQKAKFTPVNRRHHCRKCGF

VVCGPCSEKRFLLPSQSSKPVRICDFCYDLLSTGDMATCQPPRSDTYSQSLKSPLNDVSD

DDDDDDSSD

>*Notechis_scutatus*(Tiger_snakes)

MVDRLANSEANTRRISVVENCFGAAGQPLTIPGRVLIGEGVLTKLCRKKPKARQFFLFND

ILVYGNIVIQKKKYNKQHIIPLENVTIESIQDEGDLRNGWLIKTPTKSFAVYAATATEKS

EWMNHINKCVSDLLSKSGKTPSNEHAAVWVPDSEATVCMRCQKAKFTPVNRRHHCRKCGF

VVCGPCSEKRFLIPSQSSKPVRICDFCYEFLSMGEMSTCQPTRSDSFSQSPKPPLNDISD

DDDDDDSSD

>*Phascolarctos_cinereus*(koala)

MVDRLANSEANTRRISIVENCFGAAGQPLTIPGRVLIGEGVLTKLCRKKPKARQFFLFND

ILVYGNIVIQKKKYNKQHIIPLENVTIDSIKDEGDLRNGWLIKTPTKSFAVYAATATEKS

EWMNHINKCVTDLLSKSGKTPSNEHAAVWVPDSEATVCMRCQKAKFTPVNRRHHCRKCGF

VVCGPCSEKRFLLPSQSSKPVRICDFCFDLLSSGDLATCQPTRSDSYSQSPKSPLNNVSD

DEDDEDSSD

>*Bos_mutus*(Wild_yak)

MVDRLANSEANTRRINIVENCFGAAGQPLTIPGRVLIGEGVLTKLCRKKPKARQFFLFND

ILVYGNIVIQKKKYNKQHIIPLENVTIDSIKDEGDLRNGWLIKTPTKSFAVYAATATEKS

EWMNHINKCVTDLLSKSGKTPSNEHAAVWVPDSEATVCMRCQKAKFTPVNRRHHCRKCGF

VVCGPCSEKRFLLPSQSSKPVRICDFCYDLLSTGDMATCQPARSDSYSQSLKPPLNDVSD

DDDDDDSSD

>*Anser_brachyrhynchus(*Pink-footed_goose)

MVDRLANSEANTRRISIVENCFGAAGQPLTIPGRVLIGEGVLTKLCRKKPKARQFFLFND

ILVYGNIVIQKKKYNKQHIIPLENVTIDSIQDEGDLRNGWLIKTPTKSFAVYAATATEKS

EWMNHINKCVSDLLSKSGKTPSNEHAAVWVPDSEATVCMRCQKAKFTPVNRRHHCRKCGF

VVCGPCSEKRFLLPSQSSKPVRICDFCYDLLSTGEMTACQSTRSDSYSQSPKSSLNDASD

DDDDEDSSD

>*Mus_spicilegus*(Steppe_mouse)

MVDRLANSEANTRRISIVENCFGAAGQPLTIPGRVLIGEGVLTKLCRKKPKARQFFLFND

ILVYGNIVIQKKKYNKQHIIPLENVTIDSIKDEGELRNGWLIKTPTKSFAVYAATATEKS

EWMNHINKCVTDLLSKSGKTPSNEHAAVWVPDSEATVCMRCQKAKFTPVNRRHHCRKCGF

VVCGPCSEKRFLLPSQSSKPVRICDFCYDLLSTGDMAACQPARSDSYSQSLKSPLNDASD

DDDDDDSSD

>*Neovison_vison(American_mink)*

MVDRLANSEANTRRINIVENCFGAAGQPLTIPGRVLIGEGVLTKLCRKKPKARQFFLFND

ILVYGNIVIQKKKYNKQHIIPLENVTIDSIKDEGDLRNGWLIKTPTKSFAVYAATATEKS

EWMNHINKCVTDLLSKSGKTPSNEHAAVWVPDSEATVCMRCQKAKFTPVNRRHHCRKCGF

VVCGPCSEKRFLLPSQSSKPVRICDFCYDLLSTGDMATCQPTRSDSYSQSLKSPLNDVSD

DDDDDDSSD

>*Chelonoidis_abingdonii*(Abingdon_island_giant_tortoise)

MVDRLANSEANTRRISIVENCFGAAGQPLTIPGRVLIGEGVLTKLCRKKPKARQFFLFND

ILVYGNIVIQKKKYNKQHIIPLENVTIDSIQDEGDLRNGWLIKTPTKSFAVYAATATEKS

EWMSHINKCVSDLLSKSGKTPSNEHAAVWVPDSEATVCMRCQKAKFTPVNRRHHCRKCGF

VVCGPCSEKRFLLPSQSSKPVRICDFCYDLLSTGEMTTCQPTRSDSYSQSPKSPLNDVSD

DDDDDDSSD

>*Peromyscus_maniculatus*(Northern_American_deer_mouse)

MVDRLANSEANTRRISIVENCFGAAGQPLTIPGRVLIGEGVLTKLCRKKPKARQFFLFND

ILVYGNIVIQKKKYNKQHIIPLENVTIDSIKDEGELRNGWLIKTPTKSFAVYAATATEKS

EWMNHINKCVTDLLSKSGKTPSSEHAAVWVPDSEATVCMRCQKAKFTPVNRRHHCRKCGF

VVCGPCSEKRFLLPSQSSKPVRICDFCYDLLSTGDMAACQPTRSDSYSQSLKSPLNDVSD

DDDDDDSSD

>*Parus_major*(Great_tit)

MVDRLANSEANTRRISIVENCFGAAGQPLTIPGRVLIGEGVLTKLCRKKPKARQFFLFND

ILVYGNIVIQKKKYNKQHIIPLENVTIDSIQDEGDLRNGWLIKTPTKSFAVYAATATEKS

EWMNHINKCVSDLLSKSGKTPSNEHAAVWVPDSEATVCMRCQKAKFTPVNRRHHCRKCGF

VVCGPCSEKRFLLPSQSSKPVRICDFCYDLLSTGEMTACQSSRSDSYSQSPKSSLNDVSD

DDDDEDSSD

>*Crocodylus_porosus*(Australian_saltwater_crocodile)

MVDRLANSEANTRRISIVENCFGAAGQPLTIPGRVLIGEGVLTKLCRKKPKARQFFLFND

ILVYGNIVIQKKKYNKQHIIPLENVTIDSIQDEGDLRNGWLIKTPTKSFAVYAATATEKS

EWMNHINKCVSDLLSKSGKTPSNEHAAVWVPDSEATVCMRCQKAKFTPVNRRHHCRKCGF

VVCGPCSEKRFLLPSQSSKPVRICDFCYDLLSSGEMTTCQPTRSDSYSQSPKSSLNDISD

DDDDDDDSSD

>*Coturnix_japonica*(Japanese_quail)

MVDRLANSEANTRRISIVENCFGAAGQPLTIPGRVLIGEGVLTKLCRKKPKARQFFLFND

ILVYGNIVIQKKKYNKQHIIPLENVTIDSIQDEGDLRNGWLIKTPTKSFAVYAATATEKS

EWMNHINKCVSDLLSKSGKTPSNEHAAVWVPDSEATVCMRCQKAKFTPVNRRHHCRKCGF

VVCGPCSEKRFLLPSQSSKPVRICDFCYELLSTGEMTACQSTRSDSYSQSPKCSLNDASD

DDDDEDSSD

>*Bos_taurus*(Cattle)

MVDRLANSEANTRRINIVENCFGAAGQPLTIPGRVLIGEGVLTKLCRKKPKARQFFLFND

ILVYGNIVIQKKKYNKQHIIPLENVTIDSIKDEGDLRNGWLIKTPTKSFAVYAATATEKS

EWMNHINKCVTDLLSKSGKTPSNEHAAVWVPDSEATVCMRCQKAKFTPVNRRHHCRKCGF

VVCGPCSEKRFLLPSQSSKPVRICDFCYDLLSTGDMATCQPARSDSYSQSLKPPLNDVSD

DDDDDDSSD

>*Electrophorus_electricus*(Electric_eel)

MVDRLANSEANSKRIAVVESCFGTAGQPLAIPGRVLIGEGVLTKLCRKKPKARQFFLFND

ILVYGNIVIQKKKYNKQHIIPLESVTIDTVADEGDLRNGWLIKTPTKSFAVYAATATEKS

EWMSHINKCVSDLLEKSGKAPSGEHAAVWVPDSEASVCMRCQKVKFTPVSRRHHCRKCGF

VVCGPCSEKKFLLPSQSSKPVRVCEFCYEQLSTGATLPPRSNSYSRQPCSTNVSDDDDED

DSSD

>*Callorhinchus_milii*(Elephant_shark)

MVDRLANSEANARRIGMVESCFGAAGQPLTVTGRVLIGEGVLTKLCRKKAKARQFFLFND

ILVYGNIVIQKKKYNKQHIIPLENVTIDTIEDDGELRNGWLIKTPTKSFAVYAATATEKS

EWMNHITKCVADLLSKSGKIPSNEHAAVWVPDSEATICMRCQKVKFTPVSRRHHCRKCGF

VVCGPCSEKRFLLPSQSSKPVRVCSFCYDLLSSGQLNATLSNRSEFYHGALQSPGNLSDE

DEDDSSSD

>*Caenorhabditis_elegans*(Roundworm)

MVDRLVNSEVNSRRMANVEQCFGKMGEQLSVFGRVLVGEGVLVKMCRKKPKQRQFFLFND

ILVYGNIVISKKRYNKQRILRLEGVQVEDLEDDGIEKHGWIIKTPAKSFAVYAATETEKR

EWMLHIERCVTDLLERGNKQAATAHAAVWVPDGEAVKCMVCGKTQFNLVQRRHHCRNCGR

VVCGACSSRTFRIDNVHKKPVRVCDHCFDSLSSATPGQEESEPKTGNRLHHEDSSSDSED

EVNGSGRSSNESRPTFYREDVQQPAT

>*Bos_taurus*(Hybrid_cattle)

MVDRLANSEANTRRINIVENCFGAAGQPLTIPGRVLIGEGVLTKLCRKKPKARQFFLFND

ILVYGNIVIQKKKYNKQHIIPLENVTIDSIKDEGDLRNGWLIKTPTKSFAVYAATATEKS

EWMNHINKCVTDLLSKSGKTPSNEHAAVWVPDSEATVCMRCQKAKFTPVNRRHHCRKCGF

VVCGPCSEKRFLLPSQSSKPVRICDFCYDLLSTGDMATCQPARSDSYSQSLKPPLNDVSD

DDDDDDSSD

>*Aquila_chrysaetos*(Golden_eagle)

MVDRLANSEANTRRISIVENCFGAAGQPLTIPGRVLIGEGVLTKLCRKKPKARQFFLFND

ILVYGNIVIQKKKYNKQHIIPLENVTIDSIQDEGDLRNGWLIKTPTKSFAVYAATATEKS

EWMNHINKCVSDLLSKSGKTPSNEHAAVWVPDSEATVCMRCQKAKFTPVNRRHHCRKCGF

VVCGPCSEKRFLLPSQSSKPVRICDFCYDLLSTGEMSACQSTRSDSYSQSPKSSLNDVSD

DDDDEDSSD

>*Taeniopygia_guttata*(Zebra_finch)

MVDRLANSEANTRRISIVENCFGAAGQPLTIPGRVLIGEGVLTKLCRKKPKARQFFLFND

ILVYGNIVIQKKKYNKQHIIPLENVTIDSIQDEGDLRNGWLIKTPTKSFAVYAATATEKS

EWMNHINKCVSDLLSKSGKTPSNEHAAVWVPDSEAAVCMRCQKAKFTPVNRRHHCRKCGF

VVCGPCSEKRFLLPSQSSKPVRICDFCYDLLSSGEMTACQSTRSDSYSQSPKSSLNDVSD

DDDDEDSSD

>*Strigops_habroptilus*(Kakapo)

MVDRLANSEANTRRISIVENCFGAAGQPLTIPGRVLIGEGVLTKLCRKKPKARQFFLFND

ILVYGNIVIQKKKYNKQHIIPLENVTIDSIQDEGDLRNGWLIKTPTKSFAVYAATATEKS

EWMNHINKCVSDLLSKSGKTPSNEHAAVWVPDSEATVCMRCQKAKFTPVNRRHHCRKCGF

VVCGPCSEKRFLLPSQSSKPVRICDFCYDLLSTGEMTACQSTRSDSYSQSPKSSLNDVSD

DDDDEDSSD

>*Jaculus_orientalis*(Egyptian_jerboa)

VENCFGAAGQPLTIPGRVLIGEGALTKLCRSLRQGSFSYLMTVLNTKKKYNKQIIPLENV

TIGSIKDEGELRNGWLTKTPTKSFAGCAKSERMNPISKSAADFLSQSGKTASDDEICTCS

SSSPGQKCGFVVRGPCSEKRFLLPSQSSKPVRICDFCDDPLSTGAMATCQPTRSHSLSLK

SPL

>*Prolemur_simus*(Greater_bamboo_lemur)

MVDRLANSEANTRRISIVENCFGAAGQPLTIPGRVLIGEGVLTKLCRKKPKARQFFLFND

ILVYGNIVIQKKKYNKQHIIPLENVTIDSIKDEGDLRNGWLIKTPTKSFAVYAATATEKS

EWMNHINKCVTDLLSKSGKTPSNEHAAVWVPDSEATVCMRCQKAKFTPVNRRHHCRKCGF

VVCGPCSEKRFLLPSQSSKPVRICDFCYDLLSTGDMATCQPTRSDSYSQSLKSPLNDVSD

DDDDDDDSSD

>*Serinus_canaria*(Common_canary)

MVDRLANSEANTRRISIVENCFGAAGQPLTIPGRVLIGEGVLTKLCRKKPKARQFFLFND

ILVYGNIVIQKKKYNKQHIIPLENVTIDSIQDEGDLRNGWLIKTPTKSFAVYAATATEKS

EWMNHINKCVSDLLSKSGKTPSNEHAAVWVPDSEATVCMRCQKAKFTPVNRRHHCRKCGF

VVCGPCSEKRFLLPSQSSKPVRICDFCYDLLSTGEMTACQSTRSDSYSQSPKSSLNDVSD

DDDDEDSSD

>*Gorilla_gorilla*(Western_lowland_gorilla)

MVDRLANSEANTRRISIVENCFGAAGQPLTIPGRVLIGEGVLTKLCRKKPKARQFFLFND

ILVYGNIVIQKKKYNKQHIIPLENVTIDSIKDEGDLRNGWLIKTPTKSFAVYAATATEKS

EWMNHINKCVTDLLSKSGKTPSNEHAAVWVPDSEATVCMRCQKAKFTPVNRRHHCRKCGF

VVCGPCSEKRFLLPSQSSKPVRICDFCYDLLSAGDMATCQPARSDSYSQSLKSPLNDMSD

DDDDDDSSD

>*Hippocampus_comes*(Tiger_tail_seahorse)

MVDRLANSEANSKRISVVEGCFGAAGQPLAIPGRVLIGEGVLTKLCRKKPKARQFFLFND
ILVYGNIVLQKKKYNKQHIIPLESVTIDTVPDEGELRNGWLIKTPTKSFAVYAATATEKS
EWMNHIGKCVQELLQKSGKAPAGEHAAVWVPDSEAAVCMRCQKVKFTPVSRRHHCRKCGF
VVCGPCSEKKYLLPSQSSKPVRVCQFCYGQLVAGGRSDTVCGRAGDFRDVSDDDDDDDDS
SD
>*Propithecus_coquereli*(Coquerel's_sifaka)
MVDRLANSEANTRRISIVENCFGAAGQPLTIPGRVLIGEGVLTKLCRKKPKARQFFLFND
ILVYGNIVIQKKKYNKQHIIPLENVTIDSIKDEGDLRNGWLIKTPTKSFAVYAATATEKS
EWMNHINKCVTDLLSKSGKTPSNEHAAVWVPDSEATVCMRCQKAKFTPVNRRHHCRKCGF
VVCGPCSEKRFLLPSQSSKPVRICDFCYDLLSTGDMATCQPTRSDSYSQSLKSPLNDVSD
DDDDDDSSD
>*Poecilia_latipinna*(Sailfin_molly)
MVDRLANSEANSKRIGLVESCFGAAGQPLAIPGRVLIGEGVLTKLCRKKPKARQFFLFND
ILVYGNIVIQKKKYNKQHIIPLEGVTIDTVPDEGELRNGWLIKTPTKSFAVYAATATEKS
EWMNHIGKCVRELLQKSGKSPTGEHAAVWVPDSEATVCMRCKKVKFTPVSRRHHCRKCGY
VVCGPCSEKKFLLPSQSSKPVRVCEHCYGMLSGSLGPRSESMSRPGANFNNNTLSDDDDD
DDSSD
>*Astyanax_mexicanus*(Mexican_tetra)
MVDRLANSEANSKRIAVVEGCFGTAGQPLAIPGRVLIGEGVLTKLCRKKPKARQFFLFND
ILVYGNIVIQKKKYNKQHIIPLESVTIDTVADEGDLRNGWLIKTPTKSFAVYAATATEKS
EWMNHISKCVSDLLEKSGKSPTGEHAAVWVPDSEASVCMRCQKVKFTPVSRRHHCRKCGF
VVCGPCSEKKFLLPSQSSKPVRVCEFCYEQLSTEASLPVRSNSYSRTPTSKFLTNNVSDD
DDEDDSSD
>*Aotus_nancymaae_1_*(Ma's_night_monkey)
MVDRLANSEANTRRISIVENCFGAAGQPLTIPGRVLIGEGVLTKLCRKKPKARQFFLFND
ILVYGNIVIQKKKYNKQHIIPLENVTIDSIKDEGDLRNGWLIKTPTKSFAVYAATATEKS
EWMNHINKCVTDLLSKSGKTPSNEHAAVWVPDSEATVCMRCQKAKFTPVNRRHHCRKCGF
VVCGPCSEKRFLLPSQSSKPVRICDFCYDLLSTGDMATCQPTRSDSYSQSLKSPLNDMSD
DDDDDDSSD
>*Poecilia_reticulata*(Guppy)

MVDRLANSEANSKRIGLVESCFGAAGQPLAIPGRVLIGEGVLTKLCRKKPKARQFFLFND
IMVYGNIVIQKKKYNKQHIIPLEGVTIDTVPDEGELRNGWLIKTPTKSFAVYAATATEKS
EWMNHIGKCVRELLQKSGKSPTGEHAAVWVPDSEATVCMRCKKVKFTPVSRRHHCRKCGY
VVCGPCSEKKFLLPSQSSKPVRVCEHCYGMLSGSLGPRSESMSRPGANFNNNTLSDDDDD
DDSSD

>*Pygocentrus_nattereri*(Red-bellied_piranha)

MVDRLANSEANSKRIAVVESCFGTAGQPLAIPGRVLIGEGVLTKLCRKKPKARQFFLFND
ILVYGNIVIQKKKYNKQHIIPLESVTIDTVADEGDLRNGWLIKTPTKSFAVYAATATEKS
EWMSHISKCVSDLLEKSGKSPTGEHAAVWVPDSEASVCMRCQKVKFTPVSRRHHCRKCGF
VVCGPCSEKKFLLPSQSSKPVRVCEFCYDQLSTGATLPVRSNSYSLLPPSCKFGSNVSDD
EDEDDSSD

>*Anas_platyrhynchos*(Common_mallard)

MVDRLANSEANTRRISIVENCFGAAGQPLTIPGRVLIGEGVLTKLCRKKPKARQFFLFND
ILVYGNIVIQKKKYNKQHIIPLENVTIDSIQDEGDLRNGWLIKTPTKSFAVYAATATEKS
EWMNHINKCVSDLLSKSGKTPSNEHAAVWVPDSEATVCMRCQKAKFTPVNRRHHCRKCGF
VVCGPCSEKRFLLPSQSSKPVRICDFCYDLLSTGEMTACQSARPSGSSNRGIWRVEM

>*Labris_bergylta*(Ballan_wrasse)

MISRPWLYFRTVQQVRERVSALRCHIVVSVSPQLQQLVGVCLSSSCSVSCLRTLRSLRRA
APASTDSIWTNTELQVAAKVSRGREEPSGEVKMVDRLANSEANSKRIAVVEGCFGAAGQP
LAIPGRVLIGEGVLTKLCRKKPKARQFFLFNDILVYGNIVIQKKKYNKQHIIPLESVTID
TVPDEGDLHNGWLIKTPTKSFAVYAATATEKSEWMNHIGKCVGDLLQKSGKSPTGEHAAV
WVPDSEATVCMRCQKVKFTPVSRRHHCRKCGYVVCGPCSDKKYLLPSQSSKPVRVCEFCF
VQLTSGGLSIRSDSISRPGSKFNSSNMSDDDDEDDSSD

>*Aotus_nancymaae_2_*(Ma's_night_monkey)

MVDRLANSEANTRRISIVENCFGAAGQPLTIPGRVPIGEGVLTKLCRKKPKARQFFLFND
ILVYGNIVIQKKKYNKQHIIPLENVTIDSIKDEGDLRNGWLIKTPTKSFAVYAATATEKS
EWMNHINKCVTDLLSKSGKTPSNEHAAVWDPDSEATVCMRCQKAKFTPVNRRHHCRKCGF
VVCGPCSEKRFLLPSQSSKPVRICDFCYDLLSTGNMATCQPTRSDSYSQSLKSPLNDMSD
DDDDDDSSD

>*Spalax_galili*(Upper_Galilee_Mountains_blind_mole-rat)

MVDRLANSEANTRRISIVENCFGAAGQPLTIPGRVLIGEGVLTKLCRKKPKARQFFLFND
ILVYGNIVIQKKKYNKQHIIPLENVTIDSIKDEGELRNGWLIKTPTKSFAVYAATATEKS
EWMNHINKCVTDLLSKSGKTPSNEHAAVWVPDSEATVCMRCQKAKFTPVNRRHHCRKCGF
VVCGPCSEKRFLLPSQSSKPVRICDFCYELLSAGDMATCQPARSDSYSQSLKSPLNDVSD
DDDDDDSSD

>*Chrysemys_picta*(Western_painted_turtle)
MVDRLANSEANTRRISIVENCFGAAGQPLTIPGRVLIGEGVLTKLCRKKPKARQFFLFND
ILVYGNIVIQKKKYNKQHIIPLENVTIDSIQDEGDLRNGWLIKTPTKSFAVYAATATEKS
EWMSHINKCVSDLLSKSGKTPSNEHAAVWVPDSEATVCMRCQKAKFTPVNRRHHCRKCGF
VVCGPCSEKRFLLPSQSSKPVRICDFCYDLLSTGEMTTCQPTRSDSYSQSPKSPLNDVSD
DDDDDDSSD

>*Astatotilapia_burtoni*(Burton's_mouthbrooder)
MVDRLANSEANSKRIAVVESCFGAAGQPLAIPGRVLIGEGVLTKLCRKKPKARQFFLFND
ILVYGNIVIQKKKYNKQHIIPLESVTIDTVPDEGDLRNGWLIKTPTKSFAVYAATATEKS
EWMNHIGKCVGDLLQKSGKSPTGEHAAVWVPDSEASVCMRCKKVKFTPVSRRHHCRKCGF
VVCGPCSEKKYLLPSQSSKPVRVCEHCYEQLTSSLLPPQDCLYSVILLQSEP

>*Pan_troglodytes*(Chimpanzee)
MVDRLANSEANTRRISIVENCFGAAGQPLTIPGRVLIGEGVLTKLCRKKPKARQFFLFND
ILVYGNIVIQKKKYNKQHIIPLENVTIDSIKDEGDLRNGWLIKTPTKSFAVYAATATEKS
EWMNHINKCVTDLLSKSGKTPSNEHAAVWVPDSEATVCMRCQKAKFTPVNRRHHCRKCGF
VVCGPCSEKRFLLPSQSSKPVRICDFCYDLLSAGDMATCQPARSDSYSQSLKSPLNDMSD
DDDDDDSSD

>*Bison_bison*(American_bison)
MVDRLANSEANTRRINIVENCFGAAGQPLTIPGRVLIGEGVLTKLCRKKPKARQFFLFND
ILVYGNIVIQKKKYNKQHIIPLENVTIDSIKDEGDLRNGWLIKTPTKSFAVYAATATEKS
EWMNHINKCVTDLLSKSGKTPSNEHAAVWVPDSEATVCMRCQKAKFTPVNRRHHCRKCGF
VVCGPCSEKRFLLPSQSSKPVRICDFCYDLLSTGDMATCQPARSDSYSQSLKPPLNDVSD
DDDDDDSSD

>*Saimiri_sciureus*(Squirrel_monkeys)
MVDRLANSEANTRRISIVENCFGAAGQPLTIPGRVLIGEGVLTKLCRKKPKARQFFLFND

ILVYGNIVIQKKKYNKQHIIPLENVTIDSIKDEGDLRNGWLIKTPTKSFAVYAATATEKS
EWMNHINKCVTDLLSKSGKTPSNDHAAVWVPDSEATVCMRCQKAKFTPVNRRHHCRKCGF
VVCGPCSEKRFLLPSQSSKPVRICDFCYDLLSTGEMATCQPARSDSYSQSLKSPLNDMSD
DDDDDDSSD

>*Ictalurus_punctatus*(Channel_catfish)
MVDRLANSEANSKRIAVVEGCFGTAGQPLAIPGRVLIGEGVLTKLCRKKPKARQFFLFND
ILVYGNIVIQKKKYNKQHIIPLESVTIDTVADEGELRNGWLIKTPTKSFAVYAATATEKS
EWMNHINKCVSDLLEKSGKSPTGEHAAVWVPDSEASVCMRCQKVKFTPVSRRHHCRKCGF
VVCGPCSEKKFLLPSQSSKPVRVCEFCYEQLSTGATLPPRSNSYSRTNNISEDEDEDDSS
D

>*Papio_anubis*(Olive_baboon)
MVDRLANSEANTRRISIVENCFGAAGQPLTIPGRVLIGEGVLTKLCRKKPKARQFFLFND
ILVYGNIVIQKKKYNKQHIIPLENVTIDSIKDEGDLRNGWLIKTPTKSFAVYAATATEKS
EWMNHINKCVTDLLSKSGKTPSNEHAAVWVPDSEATVCMRCQKAKFTPVNRRHHCRKCGF
VVCGPCSEKRFLLPSQSSKPVRICDFCYDLLSAGDMATCQPARSDSYSQSLKSSLNDMSD
DDDDDDSSD

>*Seriola_lalandi*(Yellowtail_amberjack)
MVDRLANSEANSKRIAVVEGCFGAAGQPLAIPGRVLIGEGVLTKLCRKKPKARQFFLFND
ILVYGNIVIQKKKYNKQHIIPLESVTIDTVPDEGDLRNGWLIKTPTKSFAVYAATATEKS
EWMNHIGKCVGDLLQKSGKAPTGEHAAVWVPDSEAAVCMRCQKVKFTPVSRRHHCRKCGF
VVCGPCSEKKYLLPSQSSKPVRVCEYCYVQLTSGSLAPRSDSISRPGSKFNNLSDDEDED
DSSD

>*Mesocricetus_auratus*(Golden_hamster)
MVDRLANSEANTRRISIVENCFGAAGQPLTIPGRVLIGEGVLTKLCRKKPKARQFFLFND
ILVYGNIVIQKKKYNKQHIIPLENVTIDSIKDEGELRNGWLIKTPTKSFAVYAATATEKS
EWMNHINKCVTDLLSKSGKTPSSEHAAVWVPDSEATVCMRCQKAKFTPVNRRHHCRKCGF
VVCGPCSEKRFLLPSQSSKPVRICDFCYDLLSTGDMAACQPARSDSYSQSLKSPLNDVSD
DDDDDDSSD

>*Ursus_maritimus*(Polar_bear)
MVDRLANSEANTRRINIVENCFGAAGQPLTIPGRVLIGEGVLTKLCRKKPKARQFFLFND

ILVYGNIVIQKKKYNKQHIIPLENVTIDSIKDEGDLRNGWLIKTPTKSFAVYAATATEKS
EWMNHINKCVTDLLSKSGKTPSNEHAAVWVPDSEATVCMRCQKAKFTPVNRRHHCRKCGF
VVCGPCSEKRFLLPSQSSKPVRICDFCYDLLSTGDMSACQPTRSDSYSQSLKSPLNDVSD
DDDDDDSSD
>*Seriola_dumerili*(Greater_amberjack)
MVDRLANSEANSKRIAVVEGCFGAAGQPLAIPGRVLIGEGVLTKLCRKKPKARQFFLFND
ILVYGNIVIQKKKYNKQHIIPLESVTIDTVPDEGDLRNGWLIKTPTKSFAVYAATATEKS
EWMNHIGKCVGDLLQKSGKAPTGEHAAVWVPDSEAAVCMRCQKVKFTPVSRRHHCRKCGF
VVCGPCSEKKYLLPSQSSKPVRVCEYCYVQLTSGSLAPRSDSISRPGSKFNNLSDDEDED
DSSD
>*Marmota_marmota*(Alpine_marmot)
MVDRLANSEANTRRISIVENCFGAAGQPLTIPGRVLIGEGVLTKLCRKKPKARQFFLFND
ILVYGNIVIQKKKYNKQHIIPLENVTIDSIKDEGDLRNGWLIKTPTKSFAVYAATATEKS
EWMNHINKCVTDLLSKSGKTPSNEHAAVWVPDSEATVCMRCQKAKFTPVNRRHHCRKCGF
VVCGPCSEKRFLLPSQSSKPVRICDFCYDLLSTGDMATCQPTRSDSYSQSLKSPLNDVSD
DDDDDDSSD
>*Rhinopithecus_roxellana*(Golden_snub-nosed_monkey
MVDRLANSEANTRRISIVENCFGAAGQPLTIPGRVLIGEGVLTKLCRKKPKARQFFLFND
ILVYGNIVIQKKKYNKQHIIPLENVTIDSIKDEGDLRNGWLIKTPTKSFAVYAATATEKS
EWMNHINKCVTDLLSKSGKTPSNEHAAVWVPDSEATVCMRCQKAKFTPVNRRHHCRKCGF
VVCGPCSEKRFLLPSQSSKPVRICDFCYDLLSTGDMATCQPARSDSYSQSLKSPLNDMSD
DDDDDDSSD
>*Paramormyrops_batesii*(Paramormyrops)
MVDRLANSEANSKRIAVVEGCFGAAGQPLAIPGRVLIGEGVLTKLCRKKPKARQFFLFND
ILVYGNIVIQKKKYNKQHIIPLESVTIDTVEDEGDLRNGWLIKTPTKSFAVYAATATEKS
EWMNHINKCVSDLLEKSGKLPSSEHAAVWVPDSEASVCMRCQKIKFTPVSRRHHCRKCGF
VVCGQCSEKKFLLPSQSSKPVRVCEFCYDQLSVGQPGVPGGLSARSDSYSLSSNKFCGNN
MSDDEDEDDSSD
>*Rhinopithecus_bieti*(Black_snub-nosed_monkey)
MVDRLANSEANTRRISIVENCFGAAGQPLTIPGRVLIGEGVLTKLCRKKPKARQFFLFND

ILVYGNIVIQKKKYNKQHIIPLENVTIDSIKDEGDLRNGWLIKTPTKSFAVYAATATEKS
EWMNHINKCVTDLLSKSGKTPSNEHAAVWVPDSEATVCMRCQKAKFTPVNRRHHCRKCGF
VVCGPCSEKRFLLPSQSSKPVRICDFCYDLLSTGDMATCQPARSDSYSQSLKSPLNDMSD
DDDDDDSSD

>*Microcebus_murinus*(Gray_mouse_lemur)
MVDRLANSEANTRRISIVENCFGAAGQPLTIPGRVLIGEGVLTKLCRKKPKARQFFLFND
ILVYGNIVIQKKKYNKQHIIPLENVTIDSIKDEGDLRNGWLIKTPTKSFAVYAATATEKS
EWMNHINKCVTDLLSKSGKTPSNEHAAVWVPDSEATVCMRCQKAKFTPVNRRHHCRKCGF
VVCGPCSEKRFLLPSQSSKPVRICDFCYDLLSTGDMATCQPTRSDSYSQSLKSPLNDVSD
DDDDDDSSD

>*Panthera_pardus*(Leopard)
MVDRLANSEANTRRINIVENCFGAAGQPLTIPGRVLIGEGVLTKLCRKKPKARQFFLFND
ILVYGNIVIQKKKYNKQHIIPLENVTIDSIKDEGDLRNGWLIKTPTKSFAVYAATATEKS
EWMNHINKCVTDLLSKSGKTPSNEHAAVWVPDSEATVCMRCQKAKFTPVNRRHHCRKCGF
VVCGPCSEKRFLLPSQSSKPVRICDFCYDLLSTGDMATCQPTRSDSYSQSLKSPLNDVSD
DDDDDDSSD

>*Hucho_hucho_1_*(Huchen)
MVDRLANSEANSKRIQVVEGCFGTAGQPLVIPGRVLIGEGVLTKLCRKKPKARQFFLFND
ILVYGNIVIQKKKYNKQHIIPLESVTIDTVADEGDLRNGWLIKTPTKSFAVYAATATEKS
EWMSHIGKCVGDLLEKSGKAPTGEHAAVWIPDSEATLCMRCQKIKFTPLSRRHHCRKCGY
VVCGPCSDKKFLLPSQSSKPVRVCEFCYLQLASTGGGMGLGPRSDSYSRSVSKFNNVSDD
EDEEDSSD

>*Geospiza_fortis*(Medium_ground_finch)
MVDRLANSEANTRRISIVENCFGAAGQPLTIPGRVLIGEGVLTKLCRKKPKARQFFLFND
ILVYGNIVIQKKKYNKQHIIPLENVTIDSIQDEGDLRNGWLIKTPTKSFAVYAATATEKS
EWMNHINKCVSDLLSKSGKTPSNEHAAVWVPDSEATVCMRCQKAKFTPVNRRHHCRKCGF
VVCGPCSEKRFLLPSQSSKPVRICDFCYDLLSTGEMTACQSTRSDSYSQSPKSSLNDVSD
DDDDEDSSD

>Hucho_hucho_2_(Huchen)
MSEAKMVDRLANSEANSKRIQVVEGCFGAAGQPLVIPGRVLIGEGVLTKLCRKKPKARQF

FLFNDILVYGSIVIQKKKYNKQHIIPLESITIDTVADEGDLRNGWLIKTPTKSFAVYAAT
ATEKSEWMSHIGKCVGDLLEKSGKAPTGEHAAVWIPDSEATLCMRCQKIKFTPLSRRHHC
RKCGYVVCGPCSDKKFLLPSQSSKPVRVCEFCYLQLASAGGGTGLGPRSDSYSRSGSKFN
NVSDDEDEEDSSD
>*Ursus_americanus*(American_black_bear)
MVDRLANSEANTRRINIVENCFGAAGQPLTIPGRVLIGEGVLTKLCRKKPKARQFFLFND
ILVYGNIVIQKKKYNKQHIIPLENVTIDSIKDEGDLRNGWLIKTPTKSFAVYAATATEKS
EWMNHINKCVTDLLSKSGKTPSNEHAAVWVPDSEATVCMRCQKAKFTPVNRRHHCRKCGF
VVCGPCSEKRFLLPSQSSKPVRICDFCYDLLSTGDMAACQPTRSDSYSQSLKSPLNDVSD
DDDDDDSSD
>*Symphurus_thermophilus*(Tonguefish)
MVDRLANSEANSKRIAVVEGCFGAAGQPLAIPGRVLIGEGVLTKLCRKKPKARQFFLFND
ILVYGNIVIQKKKYNKQHIIPLESVTIDTVPDEGDLRNGWLIKTPTKSFAVYAATATEKS
EWMNHIGKCVGDLLQKSGKSPSGEHAAVWVPDSEATVCMRCQKVKFTPVSRRHHCRKCGY
VVCGPCSEKKYLLPSQSSKPVRVCEYCYIQLTSGPRSDSIGRPGLKFGNNLTDDDDDDDD
SSD
>Octodon_degus_1_(Common_degu)
MLDRLANSEANTRRIGIVENCVGAAGQPLTIPGRVLIGEGVLTKLCRKKPKARQFFLFND
ILVYGNIVIQKKKYNKQHIIPLENVTIDSIKDEGDLRNGWLIKTPTKSFAVYAATATEKS
EWMNHINKCVTDLLSKSGKTPVNEHAAVWVPDSEATVCMRCQKVKFTPVTRRHHCRKCGF
VVCGPCSEKGFLQPSQSSKPVRICDFCYDLISTGDMAACQPAWSDSYSQSVKSPLNDVSD
DDEEDSSD
>*Dipodomys_ordii*(Ord's_kangaroo_rat)
MVDRLANSEANTRRISIVENCFGAAGQPLTIPGRVLIGEGVLTKLCRKKPKARQFFLFND
ILVYGNIVIQKKKYNKQHIIPLENVTIDSIKDEGELRNGWLIKTPTKSFAVYAATATEKS
EWMNHINKCVTDLLSKSGKTPNNEHAAVWVPDSEATVCMRCQKAKFTPVNRRHHCRKCGF
VVCGPCSEKRFLLPSQSSKPVRICDFCYDLLSTGDLATCQPTRSDSYSQSLKSPLNDGSD
DDDDDDSSD
>*Mandrillus_leucophaeus*(Drill)
MVDRLANSEANTRRISIVENCFGAAGQPLTIPGRVLIGEGVLTKLCRKKPKARQFFLFND

ILVYGNIVIQKKKYNKQHIIPLENVTIDSIKDEGDLRNGWLIKTPTKSFAVYAATATEKS
EWMNHINKCVTDLLSKSGKTPSNEHAAVWVPDSEATVCMRCQKAKFTPVNRRHHCRKCGF
VVCGPCSEKRFLLPSQSSKPVRICDFCYDLLSTGDMATCQPARSDSYSQSLKSPLNDMSD
DDDDDDSSD

>Octodon_degus_2_(Common_degu)
MVDQLANSEANTRRIGIVENCFGAAGQPFTIPGRVLIGEGVLTKLCRKKPKARQFFLFND
ILVYGNTVIQKKKYNKQHIIPLENVTIDSIKDETPTKSFAVYAAAATEKSEWMNHINKCV
TDLLSKSGKTPVNEHAAVWVPDSEATKVKFTPVNRHHHCHKCGFVVCGPCSEKRFLLPSQ
FSKPVQICEFCYDLLSTVDMAACQPARSDSYSQSLKSPLNDVSDDDEEDNEKND

>*Kryptolebias_marmoratus*(Mangrove_rivulus)
MVDRLANSEANSKRIALVESCFGAAGQPLAIPGRVLIGEGVLTKLCRKKPKARQFFLFND
ILVYGNIVIQKKKYNKQHIIPLENVTIDTVPDEGELRNGWLIKTPTKSFAVYAATATEKS
EWMNHIGKCVRELLQKSGKSPTGEHAAVWVPDSEATVCMRCHKVKFTPVSRRHHCRKCGF
VVCGPCSEKKFLLPSQSSKPVRVCGHCYSLLMSGSLGPRSDSITLGPNFNNNTLSDDEDD
DDSSD

>*Acanthochromis_polyacanthus*(Spiny_chromis)
MVDRLANSEANSKRIAVVESCFGAAGQPLAIPGRVLIGEGVLTKLCRKKPKARQFFLFND
ILVYGNIVIQKKKYNKQHIIPLESVTIDTVPDEGDLRNGWLIKTPTKSFAVYAATATEKS
EWMNHIGKCVGDLLQKSGKSPTGEHAAVWVPDSEASVCMRCQKVKFTPVSRRHHCRKCGF
VVCGPCSEKKYLLPSQSSKPVRVCEYCYLQLTSGSLGPRSDSISRPGSKFNNLSDDEDED
DSSD

>*Pan_paniscus*(Bonobo)
MVDRLANSEANTRRISIVENCFGAAGQPLTIPGRVLIGEGVLTKLCRKKPKARQFFLFND
ILVYGNIVIQKKKYNKQHIIPLENVTIDSIKDEGDLRNGWLIKTPTKSFAVYAATATEKS
EWMNHINKCVTDLLSKSGKTPSNEHAAVWVPDSEATVCMRCQKAKFTPVNRRHHCRKCGF
VVCGPCSEKRFLLPSQSSKPVRICDFCYDLLSAGDMATCQPARSDSYSQSLKSPLNDMSD
DDDDDDSSD

>*Sinocyclocheilus_grahami_1_*(Golden-line_barbel)
MVDRLANSEANSKRIGVVEVCFGTAGQPLVIPGRVLIGEGVLTKLCRKRPKARQFFLFND
ILVYGNIIIQKKKYNKQHIIPLESVTIDTVEDEGDLRNGWLIKTPTKSFAVYAATATEKS

EWMSHISKCVSDLLEKSGKSPTGDHAAVWVPDSEATVCMRCQKTKFTPVSRRHHCRKCGF
VVCGPCSEKKFLLPSQSSKPVRVCEFCYKQLFSGATLPSHSDSYSHPEISSSNISDDDDD
DDSSD
>*Lates_calcarifer*(Barramundi)
MVDRLANSEANSKRIAVVESCFGAAGQPLAIPGRVLIGEGVLTKLCRKKPKARQFFLFND
ILVYGNIVIQKKKYNKQHIIPLESVTIDTVPDEGDLRNGWLIKTPTKSFAVYAATATEKS
EWMNHIGKCVGDLLQKSGKAPTGEHAAVWVPDSEATVCMRCQKVKFTPVSRRHHCRKCGF
VVCGPCSEKKYLLPSQSSKPVRVCEYCYVQLTSGSLPPRSDSMSRLGSKFNSNNLSDDED
EDDSSD
>*Sinocyclocheilus_grahami_2_*(Golden-line_barbel)
MVDRLANSEANSKRIGVVEACFGTAGQPLAIPGRVLIGEGVLTKLCRKRPKARQFFLFND
ILVYGNIVIQKKKYNKQHIIPLESVTIDTVEDEGDLRNGWLIKTPTKSFAVYAATATEKS
EWMSHISKCVSDLLEKSGKSPTGEHAAVWVPDSEATVCMRCQKMKFTPMNRRHHCRKCGF
VVCGPCSEKKFLLPSQSSKPVRVCEFCYKQLSTGGTLPSHSDSYSRSEFSSNNISDDDDD
DDSSD
>*Carlito_syrichta*(Philippine_tarsier)
MVDRLANSEANTRRISIVENCFGAAGQPLTIPGRVLIGEGVLTKLCRKKPKARQFFLFND
ILVYGNIVIQKKKYNKQHIIPLENVTIDSIKDEGDLRNGWLIKTPTKSFAVYAATATEKS
EWMNHINKCVTDLLSKSGKTPSNEHAAVWVPDSEATVCMRCQKAKFTPVNRRHHCRKCGF
VVCGPCSEKRFLLPSQSSKPVRICDFCYDLLSTGDMATCQPTRSDSYSQSLKSPLNDVSD
DDDDDDSSD
>*Canis_lupus*(Dingo)
MVDRLANSEANTRRINIVENCFGAAGQPLTIPGRVLIGEGVLTKLCRKKPKARQFFLFND
ILVYGNIVIQKKKYNKQHIIPLENVTIDSIKDEGDLRNGWLIKTPTKSFAVYAATATEKS
EWMNHINKCVTDLLSKSGKTPSNEHAAVWVPDSEATVCMRCQKAKFTPVNRRHHCRKCGF
VVCGPCSEKRFLLPSQSSKPVRICDFCYDLLSTGDMATCQPARSDSYSQSLKSPLNDVSD
DDDDDDSSD
>*Cyclopterus_lumpus*(Lumpfish)
MVDRLANSEANSKRIAVVEGCFGAAGQPLVIPGRVLIGEGVLTKLCRKKPKARQFFLFND
ILVYGNIVIQKKKYNKQHIIPLEAVTIDTVPDEGDLRNGWLIKTPTKSFAVYAATATEKS

EWMNHIGKCVEDLLQKSGKAPAGEHAAVWVPDSEATVCMRCQKVKFTPVSRRHHCRKCGF
VVCGPCSEKKHLLPSQSSKPVRVCEYCFLQLTAGGLAPRSDSLSRLGSKFNNLSDDDDED
DSSD
>*Sander_lucioperca*(Zander)
MVDRLANSEANSKRIGVVEGCFGAAGQPLAIPGRVLIGEGVLTKLCRKKPKARQFFLFND
ILVYGNIVIQKKKYNKQHIIPLESVTIDTVPDEGDLHNGWLIKTPTKSFAVYAATATEKS
EWMNHIGKCVGDLLQKSGKAPTGEHAAVWVPDSEATVCMRCQKVKFTPVSRRHHCRKCGY
VVCGPCSEKKHLLPSQSSKPVRVCEYCYVQLTSGGLAQRSDSISRLGSKFNNLSDDDDDD
DSSD
>*Nothobranchius_furzeri*(Turquoise_killifish)
MVDRLANSEANSKRIGLVESCFGAAGQPLAIPGRVLIGEGVLTKLCRKKPKARQFFLFND
ILVYGNIVIQKKKYNKQHIIPLESVTIDTVPDEGELRNGWLIKTPTKSFAVYAATATEKS
EWMNHIGKCVRELLQKSGKSPTGEHAAVWVPDSEAPLCMCCHKVKFTPVSRRHHCRKCGF
VVCGPCSEKRFLLPSQSSKPVRVCEHCYTVLMSGGLGPHDSISRPTSNFNNNTLSDDEDD
DNSSD
>*Monodon_monoceros*(Narwhal)
MVDRLANSEANTRRINIVENCFGAAGQPLTIPGRVLIGEGVLTKLCRKKPKARQFFLFND
ILVYGNIVIQKKKYNKQHIIPLENVTIDSIKDEGDLRNGWLIKTPTKSFAVYAATATEKS
EWMNHINKCVTDLLSKSGKTPSNEHAAVWVPDSEATVCMRCQKAKFTPVNRRHHCRKCGF
VVCGPCSEKRFLLPSQSSKPVRICDFCYDLLSAGDMATCQPTRSDSYSQSLKSTLNDVSD
DDDDDDSSD
>*Cervus_elaphus_1_*(Yarkand_deer)
MVDRLANSEANTRRINIVENCFGAAGQPLTIPGRVLIGEGVLTKLCRKKPKARQFFLFND
ILVYGNIVIQKKKYNKQHIIPLENVTIDSIKDEGDLRNGWLIKTPTKSFAVYAATATEKS
EWMNHINKCVTDLLSKSGKTPSNEHAAVWVPDSEATVCMRCQKAKFTPVNRRHHCRKCGF
VVCGPCSEKRFLLPSQSSKPVRICDFCYDLLSTGDMATCQPARSDSYSQSLKPPLNDVSD
DDDDDDSSD
>*Cervus_elaphus_2_*(Yarkand_deer)
MPTVDPLAKSEANTRRINIVENGFGAAGGLTELCRKKPKVRQFFLLNDILVYGNIVIQKK
KYIKQHIIPLENVTIDSIKDEGDLRNGWLIKTPTKSLAVYAATATEKSEWMNPINKCVTD

LLSKSGKTPSNEHAAVWVPDSEATVCMRCQKAKFTPVNRRHHCRKCGFVVCGPCSEKRFL
LPSQSSKPVRICDFCYDLLSTGDMATCQPARSDSYSQSLKPPLNDVSDDDSSD

>*Balaenoptera_musculus*(Blue_whale)

MVDRLANSEANTRRINIVENCFGAAGQPLTIPGRVLIGEGVLTKLCRKKPKARQFFLFND
ILVYGNIVIQKKKYNKQHIIPLENVTIDSIKDEGDLRNGWLIKTPTKSFAVYAATATEKS
EWMNHINKCVTDLLSKSGKTPSNEHAAVWVPDSEATVCMRCQRAKFTPVNRRHHCRKCGF
VVCGPCSEKRFLLPSQSSKPVRICDFCYDLLSTGDMATCQPTRSDSYSQSLKSPLNDVSD
DDDDDDSSD

>*Leptobrachium_leishanense*(Leishan_spiny_toad)

MVDRLANSEANARRINLVESCFGTAGQPLAIHGRVLIGEGVLTKLCRKKPKARQFFLFND
ILVYGNIVIQKKKYNKQHIIPLENVTIDSIEDEGDLRNGWLIKTPTKSFAVYAATATEKS
EWMNHINKCVYDLISKSGKTPNSEHAAVWIPDSEATVCMRCSKVKFTPVSRRHHCRKCGF
VICGPCSEKKFLLPNQSSKPVRVCDFCYDVLASGDLQCQAGRSDSLSSSPKFGHNLSDND
DDDDSSD

>*Naja_naja*(Indian_cobra)

MVDRLANSEANTRRISVVENCFGAAGQPLTIPGRVLIGEGVLTKLCRKKPKARQFFLFND
ILVYGNIVIQKKKYNKQHIIPLENVTIESIQDEGDLRNGWLIKTPTKSFAVYAATATEKS
EWMNHINKCVSDLLSKSGKTPSNEHAAVWVPDSEATACMRCQKAKFTPVNRRHHCRKCGF
VVCGPCSEKRFLIPSQSSKPVRICDFCYEFLSMGEMPACQPTRSDSFSQSPKPPLNDISD
DDDDDDSSD

>*Phocoena_sinus*(Vaquita)

MVDRLANSEANTRRINIVENCFGAAGQPLTIPGRVLIGEGVLTKLCRKKPKARQFFLFND
ILVYGNIVIQKKKYNKQHIIPLENVTIDSIKDEGDLRNGWLIKTPTKSFAVYAATATEKS
EWMNHINKCVTDLLSKSGKTPSNEHAAVWVPDSEATVCMRCQKAKFTPVNRRHHCRKCGF
VVCGPCSEKRFLLPSQSSKPVRICDFCYDLLSTGDMATCQPTRSDSYSQSLKSTLNDVSD
DDDDDDSSD

>*Sciurus_vulgaris_1_*(Eurasian_red_squirrel)

MVDRLANSEANTRRISIVENCFGAAGQPLTIPGRVLIGEGVLTKLCRKKPKARQFFLFND
ILVYGNIVIQKKKYNKQHIIPLENVTIDSIKDEGDLRNGWLIKTPTKSFAVYAATATEKS
EWMNHINKCVTDLLSKSGKTPSNEHAAVWVPDSEATVCMRCQKAKFTPVNRRHHCRKCGF

VVCGPCSEKRFLLPSQSSKPVRICDFCYDLLSTGDMATCQPTRSDSYSQSSKSPLNDVSD
DDDDDDSSD
>*Oncorhynchus_kisutch*(Coho_salmon)
MGGEAKMVDRLANSEANSKRIQVVEGCFGAAGQPLVIPGRVLIGEGVLTKLCRKKPKARQ
FFLFNDILVYGNIVIQKKKYNKQHIIPLESVTIDTVADEGDLRNGWLIKTPTKSFAVYAA
TATEKSEWMSHIGKCVGDLLEKSGKAPTGEHAAVWIPDSEATLCMRCQKIKFTPLSRRHH
CRKCGYVVCGPCSDKKFLLPSQSSKPVRVCEFCYLQLASAGGGMGLGPHSDSYSRSGSKF
NNVSDDEDEEDSSD
>*Sciurus_vulgaris_2_*(Eurasian_red_squirrel)
MVDRLANSEANTRRLSIVENCFGAAGQPLTIPGRVLIGEGVLTKLCRKKPKARQFFLFND
ILVYGNIVIQKKKYNKQHIIPLENVTIDSIKDEGDLRNGWLIKTPTKSFAVYAATATEKS
EWMNHINKCVTDLLSKSGKTPSNEHTAIWVPDSEATVCMHCQKAKFTPVNRRHHCRKCGF
VVCGPCSEKRFLLPNQSSKPVRICDFCYDLLSTGDMATCQPTRSDSYSQSSKSPLNNVSD
DDDDDSSD
>*Terrapene_carolina*(Three-toed_box_turtle)
MVDRLANSEANTRRISIVENCFGAAGQPLTIPGRVLIGEGVLTKLCRKKPKARQFFLFND
ILVYGNIVIQKKKYNKQHIIPLENVTIDSIQDEGDLRNGWLIKTPTKSFAVYAATATEKS
EWMSHINKCVSDLLSKSGKTPSNEHAAVWVPDSEATVCMRCQKAKFTPVNRRHHCRKCGF
VVCGPCSEKRFLLPSQSSKPVRICDFCYDLLSTGEMTTCQPTRSDSYSQSPKSPLNDVSD
DDDDDDSSD
>*Salmo_salar_1_*(Atlantic_salmon)
MVDRLANSEANSKRIQVVEGCFGTAGQPLVIPGRVLIGEGVLTKLCRKKPKARQFFLFND
ILVYGNIVIQKKKYNKQHIIPLESVTIDTVADEGDLRNGWLIKTPTKSFAVYAATATEKS
EWMSHIGKCVGDLLEKSGKAPTGEHAAVWIPDSEATLCMRCQKIKFTPLSRRHHCRKCGY
VVCGPCSDKKFLLPSQSSKPVRVCEFCYLQLASAGGGMGLGPRSDSYSRSGSKFNNVSDD
EDEEDSSD
>*Catagonus_wagneri*(Chacoan_peccary)
MVDRLANSEANTRRINIVENCFGAAGQPLTIPGRVLIGEGVLTKLCRKKPKARQFFLFND
ILVYGNIVIQKKKYNKQHIIPLENVTIDSIKDEGDLRNGWLIKTPTKSFAVYAATATEKS
EWMNHINKCVTDLLSKSGKTPSNEHAAVWVPDSEATVCMRCQKAKFTPVNRRHHCRKCGF

VVCGPCSEKRFLLPSQSSKPVRICDFCYDLLSTGDMATCQPTRSDSYSQSLKAPLNDVSD
DDDDDDSSD
>*Pseudonaja_textilis*(Eastern_brown_snake)
MVDRLANSEANTRRISVVENCFGAAGQPLTIPGRVLIGEGVLTKLCRKKPKARQFFLFND
ILVYGNIVIQKKKYNKQHIIPLENVTIESIQDEGDLRNGWLIKTPTKSFAVYAATATEKS
EWMNHINKCVSDLLSKSGKTPSNEHAAVWVPDSEATVCMRCQKAKFTPVNRRHHCRKCGF
VVCGPCSEKRFLIPSQSSKPVRICDFCYEFLSMGEMSTCQPTRSDSFSQSPKPPLNDISD
DDDDDDSSD
>*Tetraodontidae*(Fugu)
MVDRLANSEANSKRIAIVEGCFGTAGQPLAIPGRVLIGEGILTKVCRKKPKTRQFFLFND
ILVYGNILIQKKKYNKQHIIPLESVTIDTVPDDGDLRNGWLIKTPTKSFAVYAATATEKS
EWMNHIGKCVNDLVQKSGKAPTGEHAAVWVPDSEATVCMRCQKVKFTPVSRRHHCRKCGL
VVCGPCSEKKYLLPSQSSKPVRVCEFCYMQLTSGGLAPRSDSLSRPGSKFNTSNLSDDDD
DDDSSD
>*Moschus_moschiferus*(Siberian_musk_deer)
MVDRLANSEANTRRINIVENCFGAAGQPLTIPGRVLIGEGVLTKLCRKKPKARQFFLFND
ILVYGNIVIQKKKYNKQHIIPLENVTIDSIKDEGDLRNGWLIKTPTKSFAVYAATATEKS
EWMNHINKCVTDLLSKSGKTPSNEHAAVWVPDSEATVCMRCQKAKFTPVNRRHHCRKCGF
VVCGPCSEKRFLLPSQSSKPVRICDFCYDLLSTGDMATCQPARSDSYSQSLKPPLNDVSD
DDDDDDSSD
>*Equus_caballus*(Horse)
MVDRLANSEANTRRINIVEHCFGAAGQPLTIPGRVLIGEGVLTKLCRKKPKARQFFLFND
ILVYGNIVIQKKKYNKQHIIPLENVTIDSIKDEGDLRNGWLIKTPTKSFAVYAATATEKS
EWMNHINKCVTDLLSKSGKTPSNEHAAVWVPDSEATVCMRCQKAKFTPVNRRHHCRKCGF
VVCGPCSEKRFLLPSQSSKPVRICDFCYDLLSTGDMAACQPPRSDTYSQSLKSPLNDVSD
DDDDDDSSD
>*Salmo_salar_2_*(Atlantic_salmon)
MVDRLANSEANSKRIQVVEGCFGAAGQPLVIPGRVLIGEGVLTKLCRKKPKARQFFLFND
ILVYGNIVIQKKKYNKQHIIPLESVTIDTVADEGDLRNGWLIKTPTKSFAVYAATATEKS
EWMSHIGKCVGDLLEKSGKAPTGEHAAVWIPDSEATLCMRCQKIKFTPLSRRHHCRKCGY

VVCGPCSDKKFLLPSQSSKPVRVCEFCYLQLASAGGGTGLGPRSDSYSRSGSKFNNVSDD
EDEEDSSD
>*Larimichthys_crocea*(Large_yellow_croaker)
MVDRLANSEANSKRIAVVEGCFGTAGQPLAIPGRVLIGEGVLTKLCRKKPKARQFFLFND
ILVYGNIVIQKKKYNKQHIIPLESVTIDTVPDEGDLRNGWLIKTPTKSFAVYAATATEKS
EWMNHIGKCVGDLLQKSGKAPTGEHAAVWVPDSEATVCMRCQKVKFTPVSRRHHCRKCGY
VVCGPCSEKKYLLPSQSSKPVRVCEYCFVQLTSGSLGPRSDSISRPGSKFNNLSDDDDED
DSSD
>*Cottoperca_gobio*(Channel_bull_blenny)
MVDRLANSEANSKRIAVVEGCFGAAGQPLAIPGRVLIGEGVLTKLCRKKPKARQFFLFND
ILVYGNIVIQKKKYNKQHIIPLESVTIDTVPDEGDLRNGWLIKTPTKSFAVYAATATEKS
EWMNHIGKCVGDLLQKSGKAPTGEHAAVWIPDSEATVCMRCQKVKFTPVSRRHHCRKCGY
VVCGPCSEKKYLLPSQSSKPVRVCEYCFVQLTSGGPRSDSITRLGSKFNNLSDDDDEDDS
SD
>*Xenopus_tropicalis*(Western_clawed_frog)
MVDRLANSEANSKRINLVESCFGTAGQPLAIPGRVLIGEGVLTKLCRKKPKARQFFLFND
ILVYGNIVIQKKKYNKQHIIPLENVTIDTIEDEGELRNGWLIKTPTKSFAVYAATATEKS
EWMNHINKCVCDLISKSGKTPNSEHAAVWIPDSEATICMRCKKVKFTPVNRRHHCRKCGY
VICGPCSEKRYLLPSQSSKPVRVCDFCYDLLSNGDFSSCLPSRSDSFSHPSKSKNNLSDD
DDDEDSSD
>*Sus_scrofa*(Pig)
MVDRLANSEANTRRINIVENCFGAAGQPLTIPGRVLIGEGVLTKLCRKKPKARQFFLFND
ILVYGNIVIQKKKYNKQHIIPLENVTIDSIKDEGDLRNGWLIKTPTKSFAVYAATATEKS
EWMNHINKCVTDLLSKSGKTPSNEHAAVWVPDSEATVCMRCQKAKFTPVNRRHHCRKCGF
VVCGPCSEKRFLLPSQSSKPVRICDFCYDLLSTGDMATCQPTRSDSYSQSLKSPLNDVSD
DDDDDDSSD
>*Erpetoichthys_calabaricus*(Reedfish)
MVDRLANSEANSKRIAVVEGCFGAAGQPLAIPGRVLIGEGVLTKLCRKKPKARQFFLFND
ILVYGNIVIQKKKYNKQHIIPLENVTIDSIKDEGDLRNGWLIKTPTKSFAVYAATATEKS
EWMNHISKCVTDLLEKSGKAPNNEHAAVWVPDSEASVCMRCQKIKFTPVNRRHHCRKCGF

VVCGPCSEKKFLLPSQSSKPVRVCDFCYHQLSSGQLSASLPTRSDSYSRASKFSTNNMSD
DDDDDDSSD
>*Sparus_aurata*(Gilt-head_bream)
MVDRLANSEANSKRIAVVEGCFGAAGQPLAIPGRVLIGEGVLTKLCRKKPKARQFFLFND
ILVYGNIVIQKKKYNKQHIIPLESVTIDTVPDEGDLRNGWLIKTPTKSFAVYAATATEKS
EWMNHIGKCVGDLLQKSGKAPTGEHAAVWVPDSEATVCMRCQKVKFTPVSRRHHCRKCGF
VVCGPCSEKKYLLPSQSSKPVRVCEYCFVQLTSGGLAPRSDSISRPGSKFNSNNLSDDDD
EDDSSD
>*Struthio_camelus*(Ostrich)
MVDRLANSEANTRRISIVENCFGAAGQPLTIPGRVLIGEGVLTKLCRKKPKARQFFLFND
ILVYGNIVIQKKKYNKQHIIPLENVTIDSIQDEGDLRNGWLIKTPTKSFAVYAATATEKS
EWMNHINKCVSDLLSKSGKTPSNEHAAVWVPDSEATVCMRCQKAKFTPVNRRHHCRKCGF
VVCGPCSEKRFLLPSQSSKPVRICDFCYDLLSTGEMTTCQSTRSDSYSQSPKSPLNDVSD
DDEDEDSSD
>*Salmo_trutta_1_*(Brown_trout)
MSAGEAKMVDRLANSEANSKRIQVVEGCFGAAGQPLVIPGRVLIGEGVLTKLCRKKPKAR
QFFLFNDILVYGNIVIQKKKYNKQHIIPLESVTIDTVADEGDLRNGWLIKTPTKSFAVYA
ATATEKSEWMSHIGKCVGDLLEKSGKAPTGEHAAVWIPDSEATLCMRCQKIKFTPLSRRH
HCRKCGYVVCGPCSDKKFLLPSQSSKPVRVCEFCYLQLASAGGGTGLGPRSDSYSRSGSK
FNNVSDDEDEEDSSD
>*Panthera_leo*(Lion)
MVDRLANSEANTRRINIVENCFGAAGQPLTIPGRVLIGEGVLTKLCRKKPKARQFFLFND
ILVYGNIVIQKKKYNKQHIIPLENVTIDSIKDEGDLRNGWLIKTPTKSFAVYAATATEKS
EWMNHINKCVTDLLSKSGKTPSNEHAAVWVPDSEATVCMRCQKAKFTPVNRRHHCRKCGF
VVCGPCSEKRFLLPSQSSKPVRICDFCYDLLSTGDMATCQPTRSDSYSQSLKSPLNDVSD
DDDDDDSSD
>*Camelus_dromedarius*(Arabian_camel)
MVDRLANSEANTRRINIVENCFGAAGQPLTIPGRVLIGEGVLTKLCRKKPKARQFFLFND
ILVYGNIVIQKKKYNKQHIIPLENVTIDSIKDEGDLRNGWLIKTPTKSFAVYAATATEKS
EWMNHINKCVTDLLSKSGKTPSNEHAAVWVPDSEATVCMRCQKAKFTPVNRRHHCRKCGF

VVCGPCSEKRFLLPSQSSKPVRICDFCYDLLSAGDMATCQPARSDSYSQSLKSPLNDVSD
DDDDDDSSD

>*Denticeps_clupeoides*(Denticle_herring)
MVDRLANSEANSKRIAVVEGCFGTAGQPLAIPGRVLIGEGVLTKLCRKKPKARQFFLFND
ILVYGNIVIQKKKYNKQHIIPLESVTIDTVADEGDLRNGWLIKTPTKSFAVYAATATEKS
EWMSHINKCVSDLLEKSGKSPSGEHAAVWVPDSEATVCMRCQKVRFTPVNRRHHCRKCGF
VVCGPCSEKKFLLPSQSSKPVRVCEFCHEQLSSASGLPPRSDSYSRPRSNFGSNNVSEDD
DDDDSSD

>*Ornithorhynchus_anatinus*(Platypus)
MVDRLANSEANTRRISIVESCFGAAGQPLTIPGRVLIGEGVLTKLCRKKPKARQFFLFND
ILVYGNIVIQKKKYNKQHIIPLENVTIDSIQDEGELRNGWLIKTPTKSFAVYAATATEKS
EWMNHINKCVSDLLSKSGKTPSNEHAAVWIPDSEATVCMRCQKAKFTPVNRRHHCRKCGF
VVCGPCSEKRFLLPSQSSKPVRICDFCHELLSSGEMTACQPTRSDPYSQSPKSPLNNTSD
DDDDDSSSD

>*Macaca_mulatta*(Macaque)
MVDRLANSEANTRRISIVENCFGAAGQPLTIPGRVLIGEGVLTKLCRKKPKARQFFLFND
ILVYGNIVIQKKKYNKQHIIPLENVTIDSIKDEGDLRNGWLIKTPTKSFAVYAATATEKS
EWMNHINKCVTDLLSKSGKTPSNEHAAVWVPDSEATVCMRCQKAKFTPVNRRHHCRKCGF
VVCGPCSEKRFLLPSQSSKPVRICDFCYDLLSTGDMATCQPARSDSYSQSLKSPLNDMSD
DDDDDDSSD

>*Oreochromis_niloticus*(Nile_tilapia)
MVDRLANSEANSKRIAVVESCFGAAGQPLAIPGRVLIGEGVLTKLCRKKPKARQFFLFND
ILVYGNIVIQKKKYNKQHIIPLESVTIDTVPDEGDLRNGWLIKTPTKSFAVYAATATEKS
EWMNHIGKCVGDLLQKSGKSPTGEHAAVWVPDSEASVCMRCKKVKFTPVSRRHHCRKCGF
VVCGPCSEKKYLLPSQSSKPVRVCEHCYEQLTSVNLQARSDSITRGSKFHSNNLSDDDDD
DDSSD

>*Monodelphis_domestica*(Gray_short-tailed_opossums)
MAAPTLALAWAPILRGGHNCCLRGHAPLGLSAPRKGFPAVLGRRATASSRTEVPGNDRYC
VELLRKRDYEGYLCCLLLPTEARSSAFALRAFNAELAQVKDSISQKTIGQMRMQFWRKAV
EDMYSDNPPHQPVAIELWKAVKRHNLTKRWLVKIIDEREKNLDDKAYRNIQELEAYAENT

QSSLLYLTLEVLGVRNVHADHAASHIGKAQGIITCLRATPYHSSRRKVFLPMDICMLHGV
SQEDFLRKNQDKNVRDVIYDIASQGHLHLIHARSFQKSVPVKAFPAFLQTATKQKMVDRL
ANSEANTRRISIVENCFGAAGQPLTIPGRVLIGEGVLTKLCRKKPKARQFFLFNDILVYG
NIVIQKKKYNKQHIIPLENVTIDSIKDEGDLRNGWLIKTPTKSFAVYAATATEKSEWMNH
INKCVTDLLSKSGKTPSNEHAAVWVPDSEATVCMRCQKAKFTPVNRRHHCRKCGFVVCGP
CSEKRFLLPSQSSKPVRICDFCFDLLSTGDLATCQPTRSDSYSQSPKSLNNVSDDEDDED
SSD

>*Cyprinus_carpio_1_*(Common_carp)

MVDRLANSEANSKRIGVVEACFGTAGQPLAIPGRVLIGEGVLTKLCRKRPKARQFFLFND
ILVYGNIVIQKKKYNKQHIIPLESVTIDTVEDEGDLRNGWLIKTPTKSFAVYAATATEKS
EWMSHISKCVSDLLEKSGKSPTGEHAAVWVPDSEATVCMRCQKVKFTPVNRRHHCRKCGF
VVCGPCSEKKFLLPSQSSKPVRVCEFCYKQLSSDSYSHSEISGNNISDDDDDDDSSD

>*Callithrix_jacchus*(Common_marmoset)

MVDRLANSEANTRRISIVENCFGAAGQPLTIPGRVLIGEGVLTKLCRKKPKARQFFLFND
ILVYGNIVIQKKKYNKQHIIPLENVTIDSIKDEGDLRNGWLIKTPTKSFAVYAATATEKS
EWMNHINKCVTDLLSKSGKTPSNEHAAVWVPDSEATVCMRCQKAKFTPVNRRHHCRKCGF
VVCGPCSEKRFLLPSQSSKPVRICDFCYDLLSTGDMATCQPARSDSYSQSLKSPLNDMSD
DDEDDDSSD

>*Cyprinus_carpio_2_*(Common_carp)

MVDRLANSEANSKRIGVVEACFGTAGQPLAIPGRVLIGEGVLTKLCRKRPKARQFFLFND
ILVYGNIVIQKKKYNKQHIIPLESVTIDTVEDEGDLRNGWLIKTPTKSFAVYAATATEKS
EWMSHISKCVSDLLEKSGKSPTGEHAAVWVPDSEATVCMRCQKIKFNPVNRRHHCRKCGF
VVCGPCSEKKFLLPSQSSKPVRVCEFCYKQLSTGGTLPARSDSYSRSEFSSNNISDDDDD
DDSSD

>*Scleropages_formosus*(Asian_arowana)

MVDRLANSEANSKRIAVVEGCFGAAGQPLAIPGRVLIGEGVLTKLCRKKPKARQFFLFND
ILVYGNIVIQKKKYNKQHIIPLESVTIDTVEDEGELRNGWLIKTPTKSFAVYAATATEKS
EWMNHINKCVSDLLLKSGKSPSNEHAAVWVPDSEASVCMRCQKVKFTPVSRRHHCRKCGF
VVCGPCSEKKFLLPSQSSKPVRVCEFCYEQLSAGQMSGGLPPRSDSYSRSSSRFPGSNVS
EDEEEEDSSD

>*Oryzias_sinensis*(Chinese_rice_fish)

MVDRLANSEANSKRIGMVESCFGAAGQPLAIPGRVLIGEGVLTKLCRKKPKARQFFLFND

ILVYGNIVIQKKKYNKQHIIPLESVTIDTVPDEGDLRNGWLIKTPTKSFAVYAATATEKC

EWMNHIGKCVKDLLQKSGKSPTGEHAAVWVPDSEASVCMRCLKVKFTAVNRRHHCRKCGY

VVCNPCSEKKYLLPSQSSKPVRVCEFCYAQLTSGSLAPCSDSNNWSESRYNNNHLSDDED

EDDSSD

>*Clupea_harengus*(Atlantic_herring)

MVDRLANSEANNKRIGVVEGCFGTAGQPLAIPGRVLIGEGVLTKLCRKKPKARQFFLFND

ILVYGNIVIQKKKYNKQHIIPLESVTIDTVADEGDLRNGWLIKTPTKSFAVYAATATEKS

EWMSHIGKCVTDLLSKSGKAPSGEHAAVWVPDSEATICMRCLKVKFTPVSRRHHCRKCGF

VVCGPCSDKKFLLPSQSSKPVRVCEFCYEQLSLGTGLHTGRPDPYGRSPGNNVSDDDDED

DSSD

>*Gopherus_evgoodei*(Goode's_thornscrub_tortoise)

MVDRLANSEANTRRISIVENCFGAAGQPLTIPGRVLIGEGVLTKLCRKKPKARQFFLFND

ILVYGNIVIQKKKYNKQHIIPLENVTIDSIQDEGDLRNGWLIKTPTKSFAVYAATATEKS

EWMSHINKCVSDLLSKSGKTPSNEHAAVWVPDSEATVCMRCQKAKFTPVNRRHHCRKCGF

VVCGPCSEKRFLLPSQSSKPVRICDFCYDLLSTGEMTTCQPTRSDSYSQSPKSPLNDVSD

DDDDDDSSD

>*Physeter_macrocephalus*(Sperm_whale)

MVDRLANSEANTRRINIVENCFGAAGQPLTIPGRVLIGEGVLTKLCRKKPKARQFFLFND

ILVYGNIVIQKKKYNKQHIIPLENVTIDSIKDEGDLRNGWLIKTPTKSFAVYAATATEKS

EWMNHINKCVTDLLSKSGKTPSNEHAAVWVPDSEATVCMRCQKAKFTPVNRRHHCRKCGF

VVCGPCSEKRFLLPSQSSKPVRICDFCYDLLSTGDMATCQPARSDSYSRSLKSPLNDVSD

DDDDDDSSD

>*Laticauda_laticaudata*(blue-lipped_sea_krait)

MVDRLANSEANTRRISVVENCFGAAGQPLTIPGRVLIGEGVLTKLCRKKPKARQFFLFND

ILVYGNIVIQKKKYNKQHIIPLENVTIESIQDEGDLRNGWLIKTPTKSFAVYAATATEKS

EWMNHINKCVSDLLSKSGKTPSNEHAAVWVPDSEATVCMRCQKAKFTPVNRRHHCRKCGF

VVCGPCSEKRFLIPSQSSKPVRICDFCYEFLSMGEMSTCQPTRSDSFSQSPKPPLNDISD

DDDDDDSSD

>*Podarcis_muralis*(Common_wall_lizard)

MVDRLANSEANTRRISIVENCFGAAGQPLTIPGRVLIGEGVLTKLCRKKPKARQFFLFND

ILVYGNIVIQKKKYNKQHIIPLENVTIESIQDEGDLRNGWLIKTPTKSFAVYAATATEKS

EWMNHINKCVSDLLSKSGKTPSNEHAAVWVPDSEATVCMRCQKAKFTPVNRRHHCRKCGF

VVCGPCSEKRFLIPSQSSKPVRICDFCYDLLSTGEMPTCQPTRSDSYSHPPKLPLNDMSD

DDDDDDSSD

>*Delphinapterus_leucas*(Beluga_whale)

MVDRLANSEANTRRINIVENCFGAAGQPLTIPGRVLIGEGVLTKLCRKKPKARQFFLFND

ILVYGNIVIQKKKYNKQHIIPLENVTIDSIKDEGDLRNGWLIKTPTKSFAVYAATATEKS

EWMNHINKCVTDLLSKSGKTPSNEHAAVWVPDSEATVCMRCQKAKFTPVNRRHHCRKCGF

VVCGPCSEKRFLLPSQSSKPVRICDFCYDLLSAGDMATCQPTRSDSYSQSLKSTLNDVSD

DDDDDDSSD

>*Oncorhynchus_tshawytscha_1_*(Chinook_salmon)

MSPAASICLLHEDKGFAMVTHLGKPWVIFRCTVSQFETERRLFCIGCLFCSTLQEGEAKM

VDRLANSEANSKRIQVVEGCFGAAGQPLVIPGRVLIGEGVLTKLCRKKPKARQFFLFNDI

LVYGNIVIQKKKYNKQHIIPLESVTIDTVADEGDLRNGWLIKTPTKSFAVYAATATEKSE

WMSHIGKCVGDLLEKSGKAPTGEHAAVWIPDSEATLCMRCQKIKFTPLSRRHHCRKCGYV

VCGPCSDKKFLLPSQSSKPVRVCEFCYLQLASAGGGMGLGPRSDSYSRSGSKFNNVSDDE

DEEDSSD

>*Betta_splendens*(Siamese_fighting_fish)

MKETHRFFRLIWTTTQIRATKSYHRTPGEVKMVDRLANSEANSKRIAVVESCFGAAGQPL

AIPGRVLIGEGVLTKLCRKKPKSRQFFLFNDILVYGNIVIQKKKYNKQHIIPLESVTIDT

VPDEGDLRNGWLIKTPTKSFAVYAATATEKSEWMNHIGRCVEELLQKSGKAPIGEHAAVW

VPDSEASVCMRCQKVKFTPVSRRHHCRKCGFVVCGPCSDKRHLLPSQSSKPVRVCEYCYL

QLTSGNHTPRSECISRPGSKFNNDNLTDEEEEDDSSD

>*Oncorhynchus_mykiss_1_*(Rainbow_trout)

MVDRLANSEANSKRIQVVEGCFGAAGQPLVIPGRVLIGEGVLTKLCRKKPKARQFFLFND

ILVYGNIVIQKKKYNKQHIIPLESVTIDTVADEGDLRNGWLIKTPTKSFAVYAATATEKS

EWMSHIGKCVGDLLEKSGKAPTGEHAAVWIPDSEATLCMRCQKIKFTPLSRRHHCRKCGY

VVCGPCSDKKFLLPSQSSKPMNLGVHASMLIFLQGEAISIQSHVTWSTYSFQYFR

>*Oncorhynchus_mykiss_2_*(Rainbow_trout)

MVDRLANSEANSKRIQVVEGCFGAAGQPLVIPGRVLIGEGVLTKLCRKKPKARQFFLFND

ILVYGNIVIQKKKYNKQHIIPLESVTIDTVADEGDLRNGWLIKTPTKSFAVYAATATEKS

EWMSHIGKCVGDLLEKSGKAPTGEHAAVWIPDSEATLCMRCQKIKFTPLSRRHHCRKCGY

VVCGPCSDKKFLLPSQSSKPVRVCEFCYLQLASAGGGMGLGPRSDSYSRSGSKFNNVSDD

EDEEDSSD

>*Salmo_trutta_2_*(Brown_trout)

MVDRLANSEANSKRIQVVEGCFGTAGQPLVIPGRVLIGEGVLTKLCRKKPKARQFFLFND

ILVYGNIVIQKKKYNKQHIIPLESVTIDTVADEGDLRNGWLIKTPTKSFAVYAATATEKS

EWMSHIGKCVGDLLEKSGKAPTGEHAAVWIPDSEATLCMRCQKIKFTPLSRRHHCRKCGY

VVCGPCSDKKFLLPSQSSKPVRVCEFCYLQLASAGGGMGLGPRSDSYSRSGSKFNNVSDD

EDEEDSSD

>*Dicentrarchus_labrax*(European_bass)

MVDRLANSEANSKRIAVVEGCFGAAGQPLAIPGRVLIGEGVLTKLCRKKPKARQFFLFND

ILVYGNIVIQKKKYNKQHIIPLESVTIDTVPDEGDLRNGWLIKTPTKSFAVYAATATEKS

EWMSHIGKCVGDLLQKSGKAPTGEHAAVWVPDSEATVCMRCQKVKFTPVSRRHHCRKCGY

VVCGPCSEKKYLLPSQSSKPVRVCEYCFVQLTSGGLGPRSDSIGRPVSKFNSNNLSDDDD

EDDSSD

>*Oryzias_javanicus*(Javanese_Ricefish)

MVDRLANSEANSKRIAMVESCFGAAGQPLAIPGRVLIGEGVLTKLCRKKPKARQFFLFND

ILVYGNIVIQKKKYNKQHIIPLESVTIDTVPDEGDLRNGWLIKTPTKSFAVYAATGTEKC

EWMNHIGKCVRDLLQKSGKSPTGEHAAVWVPDSEAPICMRCEKVKFTPVIRRHHCRKCGY

VVCGPCSEKKYLIPSQSSKPVRVCEYCYSQLTSGSHAPRSDSNNWAESRYNNNHLSDDED

EDDSSD

>*Salmo_salar_3_*(Atlantic_salmon)

MVDRLANSEANSKRIQVVEGCFGAAGQPLVIPGRVLIGEGVLTKLCRKKPKARQFFLFND

ILVYGNIVIQKKKYNKQHIIPLESVTIDTVADEGDLRNGWLIKTPTKSFAVYAATATEKS

EWMSHIGKCVGDLLEKSGKAPTGEHAAVWIPDSEATLCMRCQKIKFTPLSRRHHCRKCGY

VVCGPCSDKKFLLPSQSSKPVRVCEFCYLQLASIHSLHSSVLGKSNFFF

>*Myripristis_murdjan*(Pinecone_soldierfish)

MVDRLANSEANSKRIAVVESCFGAAGQPLAIPGRVLIGEGVLTKLCRKKPKARQFFLFND
ILVYGNIVIQKKKYNKQHIIPLESVTIDTVPDEGDLRNGWLIKTPTKSFAVYAATATEKS
EWMNHIGKCVGDLLQKSGKTPTGEHAAVWVPDSEATVCMRCQKVKFTPVSRRHHCRKCGF
VVCGPCSEKKYLLPSQSSKPVRVCEYCYVQLTSSGGGLAPRFDSISRTGSKFNSNNVSDD
EDEDDSSD
>*Sarcophilus_harrisii*(Tasmanian_devil)
MVDRLANSEANTRRISIVENCFGAAGQPLTIPGRVLIGEGVLTKLCRKKPKARQFFLFND
ILVYGNIVIQKKKYNKQHIIPLENVTIDSIKDEGDLRNGWLIKTPTKSFAVYAATATEKS
EWMNHINKCVTDLLSKSGKTPSNEHAAVWVPDSEATVCMRCQKAKFTPVNRRHHCRKCGF
VVCGPCSEKRFLLPSQSSKPVRICDFCFDLLSSGDLATCQPTRSDSYSQSTKSPLNNVSD
DEDDEDSSD
>*Carassius_auratus_1_*(Goldfish)
MVDRLANSEANSKRIGVVETCFGTAGQPLAIPGRVLIGEGVLTKLCRKRPKARQFFLFND
ILVYGNIVIQKKKYNKQHIIPLESVTIDTVEDEGELRNGWLIKTPTKSFAVYAATATEKS
EWMSHISKCVSDLLEKSGKSPTGEHAAVWVPDSEATVCMRCQKIKFNPVNRRHHCRKCGF
VVCGPCSEKKFLLPSQSTKPVRVCEFCYKQLSTGGTLPSRSDSLLSSNNISDDDDDDDSS
D
>*Oncorhynchus_tshawytscha_2_*(Chinook_salmon)
MSEAKMVDRLANSEANSKRIQVVEGCFGAAGQPLVIPGRVLIGEGVLTKLCRKKPKARQF
FLFNDILVYGNIVIQKKKYNKQHIIPLESVTIDTVAEEGDLRKGWLIKTPTKSFAVYAAT
ATEKSEWMSHIGKCVGDLLEKSGKAPTGEHAAVWIPDSEATLCMRCQKIKFTPLSRRHHC
RKCGYVVCGPCSDKKFLLPSQSSKPVRVCEFCYLQLASAGGGMGLGPRSDSYSRSGSKFN
NVSDDEDEEDSSD
>*Carassius_auratus_2_*(Goldfish)
MVDRLANSEANSKRIGVVEACFGTAGQPLAIPGRVLIGEGVLTKLCRKRPKARQFFLFND
ILVYGNIVIQKKKYNKQHIIPLESVTIDTVEDEGDLRNGWLIKTPTKSFAVYAATASEKS
EWMSHISKCVSDLLEKSGKSPTGEHAAVWVPDSEATVCMRCQKIKFNPVNRRHHCRKCGF
VVCGPCSEKKFLLPSQSTKPVRVCEFCYKQLSSGSYTHLEISGNNISDDDDDDDSSD
>*Mus_musculus*(Mouse)
MVDRLANSEANTRRISIVESCFGAAGQPLTIPGRVLIGEGVLTKLCRKKPKARQFFLFND

ILVYGNIVIQKKKYNKQHIIPLENVTIDSIKDEGELRNGWLIKTPTKSFAVYAATATEKS

EWMNHINKCVTDLLSKSGKTPSNEHAAVWVPDSEATVCMRCQKAKFTPVNRRHHCRKCGF

VVCGPCSEKRFLLPSQSSKPVRICDFCYDLLSTGDMAACQPTRSDSYSQSLKSPLNDASD

DDDDDDSSD

>*Gadus_morhua*(Atlantic_cod)

MVDRLANSEANSKRIGVVEGCFGAAGQPLVIPGRVLIGEGVLTKLCRKRPKARQFFLFND

ILVYGNIVIQKKKYNKQHIIPLESVTIDTVPDEGDLRNGWLIKTPTKSFAVYAATATEKS

EWMNHIAKCVADLLSKSGKSPTGEHAAVWVPDSEATVCMRCLKVKFTPVSRRHHCRKCGF

VVCGPCSDKKYLLPSQSSKPVRVCEFCYVHLTSAVGGYAQRADSFGRQGFNLSDDEDEDD

SSD

>*Drosophila_melanogaster*(Fruit_fly)

MVDRLVNSEANTRRIASVENCFGSSGVPLAMQGRVLVGEGVLTKMCRKRPKSRQFFLFND

ILVYGNIVIGKKKYNKQHIMPLEEVSLESIADNQTYRNGWYIRTTTKSFVVFAATSTEKQ

EWMAHINKCVEDLLRKSGKKPVENHAAVWVPDTDASVCMHCKKTQFTFIQRRHHCRNCGA

VVCAGCSAKKFLLPQQSTKALRVCDACYERLKHVPSSLGSGEDSAAATGAASGNKLNTTA

GDSSNDEDSDEETASPGGESHDEPRFYGDNSVLSAVEDSSTITSPSSATTGSLEAPQVTP

SVQSSPAAVATTGSHC

>*Ailuropoda_melanoleuca*(Giant_panda)

MVDRLANSEANTRRINIVENCFGAAGQPLTIPGRVLIGEGVLTKLCRKKPKARQFFLFND

ILVYGNIVIQKKKYNKQHIIPLENVTIDSIKDEGDLRNGWLIKTPTKSFAVYAATATEKS

EWMNHINKCVTDLLSKSGKTPSNEHAAVWVPDSEATVCMRCQKAKFTPVNRRHHCRKCGF

VVCGPCSEKRFLLPSQSSKPVRICDFCYDLLSTGDMAACQPTRSDSHSQSLKSPLNDVSD

DDDDDDSSD

>*Mastacembelus_armatus*(Zig-zag_eel)

MVDRLANSEANSKRIAVVESCFGTAGQPLAIPGRVLIGEGVLTKLCRKKPKARQFFLFND

ILVYGNIVIQKKKYNKQHIIPLESVTIDTVPDEGDLRNGWLIKTPTKSFAVYAATATEKS

EWMNHIGKCVEDLLQKSGKTPTGEHAAVWVPDSEASVCMRCQKVKFTPVSRRHHCRKCGF

VVCGPCSEKKYLLPSQSSKPVRVCEYCFLQLTAGSLAPRSDSISRPGSKFHSNNLSDDED

EDDSSD

>*Macaca_fascicularis*(Crab-eating_macaque)

MVDRLANSEANTRRISIVENCFGAAGQPLTIPGRVLIGEGVLTKLCRKKPKARQFFLFND
ILVYGNIVIQKKKYNKQHIIPLENVTIDSIKDEGDLRNGWLIKTPTKSFAVYAATATEKS
EWMNHINKCVTDLLSKSGKTPSNEHAAVWVPDSEATVCMRCQKAKFTPVNRRHHCRKCGF
VVCGPCSEKRFLLPSQSSKPVRICDFCYDLLSTGDMATCQPARSDSYSQSLKSPLNDMSD
DDDDDDSSD

>*Anabas_testudineus*(Climbing_perch)
MVDRLANSEANSKRIAVVEGCFGAAGQPLAIPGRVLIGEGVLTKLCRKKPKARQFFLFND
ILVYGNIVIQKKKYNKQHIIPLESVTIDTVPDEGDLRNGWLIKTPTKSFAVYAATATEKS
EWMNHIGKCVEDLLQKSGKAPTGEHAAVWVPDSEASVCMRCQKVKFTPVSRRHHCRKCGF
VVCGPCSEKKHLLPSQSSKPVRVCEYCYVQLTSGNHAPRSDCISRSKFNSNNLSDDEDED
DSSD

>*Felis_catus*(Cat)
MVDRLANSEANTRRINIVENCFGAAGQPLTIPGRVLIGEGVLTKLCRKKPKARQFFLFND
ILVYGNIVIQKKKYNKQHIIPLENVTIDSIKDEGDLRNGWLIKTPTKSFAVYAATATEKS
EWMNHINKCVTDLLSKSGKTPSNEHAAVWVPDSEATVCMRCQKAKFTPVNRRHHCRKCGF
VVCGPCSEKRFLLPSQSSKPVRICDFCYDLLSTGDMATCQPTRSDSYSQSLKSPLNDVSD
DDDDDDSSD
