# Supplementary material for: The PH Domain and C-Terminal polyD Motif of Phafin2 Exhibit a Unique Concurrence in Animals
Source: Membranes (Basel). 2022 Jul 7;12(7):696. doi: 10.3390/membranes12070696 (PMC9324892; doi:10.3390/membranes12070696)
Supplement: Supplementary file 1 [file membranes-12-00696-s001.zip › Supplementary File S2.pdf]

Supplementary File S2: Multiple sequence alignment of the polyD region of human Phafin2 orthologues

```
Homo_sapiens(Human)                                          PARSDSYSQS-----LKSPLNDMSDDDDDDDDSSD------------------------- 249
Ficedula_albicollis(Collared_flycatcher)                     STRSDSYSQS-----PKSPLNDVSDDDDDEDSSD------------------------- 249
Ciona_savignyi(Pacific_transparent_sea_squirt)               PPQN---------NKLAFGDTSISDSESSDD---TRT----------------------- 254
Latimeria_chalumnae(Coelacanths)                             SKSDS-YRRS---PKSSI--DNVSDEDEDEDSSD-------------------------- 250
Petromyzon_marinus(Sea_lamprey)                              AGPL-----------SSSSSDDLDSDEDDDDDDDGGD----------------------- 250
Lepisosteus_oculatus(Spotted_gar)                            ARSDS-YSRS-----SKFPAPSVSDDDDEDDSSD------------------------- 248
Myotis_lucifugus(Little_brown_bat)                           PTRSDSYSQS-----LKSPLNDVSDDDDDDDDSSD------------------------- 249
Pongo_pygmaeus(Bornean_orangutan)                            PARSDSYSQS-----LKSPLNDMSDDDDDDDDSSD------------------------- 249
Dichotomyctere_nigroviridis(Green_spotted_puffer)            PRSDS-MSR----PAATFSSSNLCWDEGSSSSSD-------------------------- 246
Pteropus_vampyrus(Large_flying_fox)                          PTRSDSYSQS-----LKSPLNDVSDDDDDDDDSSD------------------------- 249
Loxodonta_africana(African_bush_elephant)                    PTRSDSYSQS-----LKSPLNDVSDDDDDDDDSSD------------------------- 249
Procavia_capensis(Rock_hyrax)                                -SRSDSYSQS-----LKSPLNDVSDDDDDDDDSSD------------------------- 245
Ciona_intestinalis(Vase_tunicate)                            NVPN---------NKLAFGDTSYSSDSESSN---EA------------------------ 258
Gasterosteus_aculeatus(Three_spined_stickleback)             ARSDS-LGR----LGSKFHN--MSDDDDDDDDSSD------------------------- 244
Tursiops_truncatus(Bottlenose_dolphins)                      PTRSDSYSQS-----LKSTLNDVSDDDDDDDDSSD------------------------- 249
Poecilia_formosa(Amazon_molly)                               PRSES-MSR----PGANFNNNTLSDDDDDDDDSSD------------------------- 245
Mustela_putorius(Ferret)                                     PTRSDSYSQS-----LKSPLNDVSDDDDDDDDSSD------------------------- 249
Otolemur_garnettii(Northern_greater_galago)                  PTRSDSYGQS-----LKSSLNDVSDDDDDDDDSSD------------------------- 249
Chlorocebus_sabaeus(Green_monkey)                            PARSDSYSQS-----LKSPLNDMSDDDDDDDDSSD------------------------- 249
Dasypus_novemcinctus(Nine_banded_armadillo)                  PNRSDSYSHS-----LKSPLNDVSDDDDDDDDSSD------------------------- 249
Tupaia_belangeri(Northern_treeshrew)                         PTRSDSYSQS-----LKSPLNDVSDDDDDDDDSSD------------------------- 250
Anolis_carolinensis(Carolina_anole)                          SGRSDSYSPS-----LKPPLNDISDDDDDDDDSSD------------------------- 249
Pelodiscus_sinensis(Chinese_softshell_turtle)                PTRSDSYSQS-----PKSPINDVSDDDDDDDDSSD------------------------- 249
Ochotona_princeps(American_pika)                             PARSDSYSQS-----LKSPLNDVSDDDDDDDDSSD------------------------- 249
Erinaceus_europaeus(European_hedgehog)                       PARSDSYSQS-----LKSPLNDVSDDDDDDDDSSD------------------------- 249
Vicugna_pacos(Alpaca)                                        PARSDSYSQS-----LKSPLNDVSDDDDDDDDSSD------------------------- 249
Sorex_araneus(Common_shrew)                                  PTRSDSYSQS-----LKSPLNDASDDDDDDDDSSD------------------------- 249
Choloepus_hoffmanni(Hoffmann's_two-toed_sloth)               ------------------------------------------------------------ 146
Macropus_eugenii(Tammar_wallaby)                             PSRSDSYSQS-----PKSPLNNVSDDEDDEDSSD------------------------- 249
Heterocephalus_glaber_1_(Naked_mole-rat)                     PGRSDSYSQS-----LKSSLNNVSDDEDDDSSD--------------------------- 248
Heterocephalus_glaber_2_(Naked_mole-rat)                     ARSDSVYSQS-----LKSSLNNVSDDEDDDSSA--------------------------- 239
Chinchilla_lanigera(Long-tailed_chinchilla)                  PARSDSYSQS-----LKSPLNDVSDDDDDDDSSD-------------------------- 248
Cavia_porcellus(Guinea_pig)                                  PARSDSYSQS-----LKSPLNNVSDDDDDDDSSD-------------------------- 248
Ictidomys_tridecemlineatus(Thirteen-lined_ground_squirre)    PTRSDSYSQS-----LKSPLNDVSDDDDDDDDSSD------------------------- 249
Heterocephalus_glaber(Naked_mole-rat)                        ARSDSIYSQS-----LKSSLNNVSDDEDDDSSD--------------------------- 235
Rattus_norvegicus(Brown_rat)                                 PTRSDSYSQS-----LKSPLNDASDDDDDDDDSSD------------------------- 249
Clethrionomys_glareolus(Vole)                                PARSDSYSQS-----LKPPLNDVSDDDDDDDDSSD------------------------- 249
Cricetulus_griseus(Chinese_hamster)                          PTRSDSYSQS-----LKSPLNDVSDDDDDDDDSSD------------------------- 249
Cercocebus_atys(Sooty_mangabey)                              PARSDSYSQS-----LKSPLNDMSDDDDDDDDSSD------------------------- 249
Cebus_imitator(White-faced_capuchin)                         PARSDSYSQS-----LKSPLNDMSDDDDDDDDSSD------------------------- 249
Macaca_nemestrina(Southern_pig-tailed_macaque)               PARSDSYSQS-----LKSPLNDMSDDDDDDDDSSD------------------------- 249
Nomascus_leucogenys(Northern_white-cheeked_gibbon)           PARSDSYSQS-----LKSPLNDMSDDDDDDDDSSD------------------------- 249
Capra_aegagrus(Goat)                                         PARSDSYSQS-----LQPPLNDVSDDDDDDDDSSD------------------------- 249
Sorex_araneus(Shrews)                                        PTRSDSYSQS-----LKSPLNDASDDDDDDDDSSD------------------------- 249
Mus_spretus(Algerian_mouse)                                  PTRSDSYSQS-----LKSPLNDASDDDDDDDDSSD------------------------- 249
Mus_caroli(Ryukyu_mouse)                                     PTRSDSYSQS-----LKSPLNDASDDDDDDDDSSD------------------------- 249
Eptatretus_burgeri(Inshore_hagfish)                          ------TSRRGSDGPVPCPGGL---DSSDEDSSDSGEEIGSSLPGYTTDFYS-------- 266
```

```
Panthera_tigris(Tiger)                                      PTRSDSYSQS-----LKSPLNDVSDDDDDDDSSD-------------------------- 249
Danio_rerio(Zebrafish)                                      PRSDS-YSRQ----GSDFGSNNISDDDDDDDSSD-------------------------- 247
Cyprinodon_variegatus(Sheepshead_minnow)                    PRSDS-ITR----PGANFNNNTLSDDEDEDDSSD-------------------------- 245
Amphilophus_citrinellus(Midas_cichlid)                      QHSDT-ITR----PGSKFNSSNLSDDDDDDDSSD-------------------------- 246
Oryzias_dancena(Indian_medaka)                              PRSDS-NNW----GESRYNNNHLSDDEDEDDSSD-------------------------- 281
Stegastes_partitus(Bicolor_damselfish)                      PRSDS-ISR------SKFNSNNLSDDEDEDDSSD-------------------------- 244
Amphiprion_percula(Orange_clownfish)                        PRSDS-ISR----PGSKFNSNNLSDDEDEDDSSD-------------------------- 246
Fundulus_heteroclitus(Mummichog)                            PNADS-LSR----PGLNFNNTL-SDDDDDDDSSD-------------------------- 244
Maylandia_zebra(Zebra_mbuna)                                RSDSI-TRP-----GSKFHSNNLSDDDDDDDSSD-------------------------- 245
Astatotilapia_calliptera(Eastern_happy)                     RSDSI-TRP-----GSKFHSNNLSDDDDDDDSSD-------------------------- 245
Oryzias_latipes(Japanese_rice_fish)                         PCSDS-NSW----SESRYNNNHLSDDEDEDDSSD-------------------------- 246
Amphiprion_ocellaris(Clownfish)                             PRSDS-ISR----PGSKFNSNNLSDDEDEDDSSD-------------------------- 246
Xiphophorus_maculatus(Southern_platyfish)                   RQSES-MSR----SGANFNNTL-SDDDDDDDSSD-------------------------- 244
Neolamprologus_brichardi(Lyretail_cichlid)                  RSDSI-TRP-----GSKFHSNNLSDDDDDDDSSD-------------------------- 245
Scophthalmus_maximus(Turbot)                                VRSDS-FGR----MGSKFNN--LSDDDDEDDSSD-------------------------- 244
Pundamilia_nyererei(Makobe_Island_cichlid)                  RSDSI-TRP-----GSKFHSNNLSDDDDDDDSSD-------------------------- 245
Vulpes_vulpes(Red_fox)                                      PARSDSYSQS-----LKSPLNDVSDDDDDDDSSD-------------------------- 249
Gallus_gallus(Chicken)                                      STRSDSYSQS-----PKSSLNDASDDDDDEDSSD-------------------------- 249
Sphenodon_punctatus(Tuatara)                                PTRSDSYSQS-----PKSPLNNVSDDDDDDDSSD-------------------------- 249
Equus_asinus(Donkey)                                        PPRSDTYSQS-----LKSPLNDVSDDDDDDDSSD-------------------------- 249
Notechis_scutatus(Tiger_snakes)                             PTRSDSFSQS-----PKPPLNDISDDDDDDDSSD-------------------------- 249
Phascolarctos_cinereus(koala)                               PTRSDSYSQS-----PKSPLNNVSDDEDDEDSSD-------------------------- 249
Bos_mutus(Wild_yak)                                         PARSDSYSQS-----LKPPLNDVSDDDDDDDSSD-------------------------- 249
Anser_brachyrhynchus(Pink-footed_goose)                     STRSDSYSQS-----PKSSLNDASDDDDDEDSSD-------------------------- 249
Mus_spicilegus(Steppe_mouse)                                PARSDSYSQS-----LKSPLNDASDDDDDDDSSD-------------------------- 249
Neovison_vison(American_mink)                               PTRSDSYSQS-----LKSPLNDVSDDDDDDDSSD-------------------------- 249
Chelonoidis_abingdonii(Abingdon_island_giant_tortoise)      PTRSDSYSQS-----PKSPLNDVSDDDDDDDSSD-------------------------- 249
Peromyscus_maniculatus(Northern_American_deer_mouse)        PTRSDSYSQS-----LKSPLNDVSDDDDDDDSSD-------------------------- 249
Parus_major(Great_tit)                                      SSRSDSYSQS-----PKSSLNDVSDDDDDEDSSD-------------------------- 249
Crocodylus_porosus(Australian_saltwater_crocodile)          PTRSDSYSQS-----PKSSLNDISDDDDDDDDSSD------------------------- 250
Coturnix_japonica(Japanese_quail)                           STRSDSYSQS-----PKCSLNDASDDDDDEDSSD-------------------------- 249
Bos_taurus(Cattle)                                          PARSDSYSQS-----LKPPLNDVSDDDDDDDSSD-------------------------- 249
Electrophorus_electricus(Electric_eel)                      PRSNS-YSRQ---PCS----TNVSDDDDEDDSSD-------------------------- 244
Callorhinchus_milii(Elephant_shark)                         NRSEF-YHGA------LQSPGNLSDEDEDDSSSD-------------------------- 248
Caenorhabditis_elegans(Roundworm)                           -----------------KTGNRLHHEDSSSDSEDEVNGSGRSSNESRPTFYREDVQQPAT 266
Bos_taurus(Hybrid_cattle)                                   PARSDSYSQS-----LKPPLNDVSDDDDDDDSSD-------------------------- 249
Aquila_chrysaetos(Golden_eagle)                             STRSDSYSQS-----PKSSLNDVSDDDDDEDSSD-------------------------- 249
Taeniopygia_guttata(Zebra_finch)                            STRSDSYSQS-----PKSSLNDVSDDDDDEDSSD-------------------------- 249
Strigops_habroptilus(Kakapo)                                STRSDSYSQS-----PKSSLNDVSDDDDDEDSSD-------------------------- 249
Jaculus_orientalis(Egyptian_jerboa)                         TRSHS-LSLKSPL----------------------------------------------- 183
Prolemur_simus(Greater_bamboo_lemur)                        PTRSDSYSQS-----LKSPLNDVSDDDDDDDDSSD------------------------- 250
Serinus_canaria(Common_canary)                              STRSDSYSQS-----PKSSLNDVSDDDDDEDSSD-------------------------- 249
Gorilla_gorilla(Western_lowland_gorilla)                    PARSDSYSQS-----LKSPLNDMSDDDDDDDSSD-------------------------- 249
Hippocampus_comes(Tiger_tail_seahorse)                      ---DT-VCG----RAGDFRDVSD-DDDDDDDSSD-------------------------- 242
Propithecus_coquereli(Coquerel's_sifaka)                    PTRSDSYSQS-----LKSPLNDVSDDDDDDDSSD-------------------------- 249
Poecilia_latipinna(Sailfin_molly)                           PRSES-MSR----PGANFNNNTLSDDDDDDDSSD-------------------------- 245
Astyanax_mexicanus(Mexican_tetra)                           VRSNS-YSRT---PTSKFLTNNVSDDDDEDDSSD-------------------------- 248
Aotus_nancymaae_1_(Ma's_night_monkey)                       PTRSDSYSQS-----LKSPLNDMSDDDDDDDSSD-------------------------- 249
Poecilia_reticulata(Guppy)                                  PRSES-MSR----PGANFNNNTLSDDDDDDDSSD-------------------------- 245
Pygocentrus_nattereri(Red-bellied_piranha)                  VRSNS-YSLL---PPSCKFGSNVSDDEDEDDSSD-------------------------- 248
Anas_platyrhynchos(Common_mallard)                          SARPSGSSNR-----GIWRVEM------------------------------------ 237
```

```
Labris_bergylta(Ballan_wrasse)                              IRSDS-ISR----PGSKFNSSNMSDDDDEDDSSD-------------------------- 338
Aotus_nancymaae_2_(Ma's_night_monkey)                       PTRSDSYSQS-----LKSPLNDMSDDDDDDDSSD-------------------------- 249
Spalax_galili(Upper_Galilee_Mountains_blind_mole-rat)       PARSDSYSQS-----LKSPLNDVSDDDDDDDSSD-------------------------- 249
Chrysemys_picta(Western_painted_turtle)                     PTRSDSYSQS-----PKSPLNDVSDDDDDDDSSD-------------------------- 249
Astatotilapia_burtoni(Burton's_mouthbrooder)                PQDCL-YS------------VILIQSEP-------------------------------- 232
Pan_troglodytes(Chimpanzee)                                 PARSDSYSQS-----LKSPLNDMSDDDDDDDSSD-------------------------- 249
Bison_bison(American_bison)                                 PARSDSYSQS-----LKPPLNDVSDDDDDDDSSD-------------------------- 249
Saimiri_sciureus(Squirrel_monkeys)                          PARSDSYSQS-----LKSPLNDMSDDDDDDDSSD-------------------------- 249
Ictalurus_punctatus(Channel_catfish)                        PRSNS-YSRT----------NNISEDEDEDDSSD-------------------------- 241
Papio_anubis(Olive_baboon)                                  PARSDSYSQS-----LKSSLNDMSDDDDDDDSSD-------------------------- 249
Seriola_lalandi(Yellowtail_amberjack)                       PRSDS-ISR----PGSKFN--NLSDDEDEDDSSD-------------------------- 244
Mesocricetus_auratus(Golden_hamster)                        PARSDSYSQS-----LKSPLNDVSDDDDDDDSSD-------------------------- 249
Ursus_maritimus(Polar_bear)                                 PTRSDSYSQS-----LKSPLNDVSDDDDDDDSSD-------------------------- 249
Seriola_dumerili(Greater_amberjack)                         PRSDS-ISR----PGSKFN--NLSDDEDEDDSSD-------------------------- 244
Marmota_marmota(Alpine_marmot)                              PTRSDSYSQS-----LKSPLNDVSDDDDDDDSSD-------------------------- 249
Rhinopithecus_roxellana(Golden_snub-nosed_monkey            PARSDSYSQS-----LKSPLNDMSDDDDDDDSSD-------------------------- 249
Paramormyrops_batesii(Paramormyrops)                        ARSDS-YSLS---S-NKFCGNNMSDDEDEDDSSD-------------------------- 252
Rhinopithecus_bieti(Black_snub-nosed_monkey)                PARSDSYSQS-----LKSPLNDMSDDDDDDDSSD-------------------------- 249
Microcebus_murinus(Gray_mouse_lemur)                        PTRSDSYSQS-----LKSPLNDVSDDDDDDDSSD-------------------------- 249
Panthera_pardus(Leopard)                                    PTRSDSYSQS-----LKSPLNDVSDDDDDDDSSD-------------------------- 249
Hucho_hucho_1_(Huchen)                                      PRSDS-YSRS------VSKFNNVSDDEDEEDSSD-------------------------- 248
Geospiza_fortis(Medium_ground_finch)                        STRSDSYSQS-----PKSSLNDVSDDDDDEDSSD-------------------------- 249
Hucho_hucho_2_(Huchen)                                      PRSDS-YSRS------GSKFNNVSDDEDEEDSSD-------------------------- 253
Ursus_americanus(American_black_bear)                       PTRSDSYSQS-----LKSPLNDVSDDDDDDDSSD-------------------------- 249
Symphurus_thermophilus(Tonguefish)                          PRSDS-IGR----PGLKFGNNLTDDDDDDDDSSD-------------------------- 243
Octodon_degus_1_(Common_degu)                               PAWSDSYSQS-----VKSPLNDVSDDDEEDSSD--------------------------- 248
Dipodomys_ordii(Ord's_kangaroo_rat)                         PTRSDSYSQS-----LKSPLNDGSDDDDDDDSSD-------------------------- 249
Mandrillus_leucophaeus(Drill)                               PARSDSYSQS-----LKSPLNDMSDDDDDDDSSD-------------------------- 249
Octodon_degus_2_(Common_degu)                               ARSDS-YSQS-----LKSPLNDVSDDDEEDNEKND------------------------- 234
Kryptolebias_marmoratus(Mangrove_rivulus)                   PRSDS-IT-----LGPNFNNNTLSDDEDDDDSSD-------------------------- 245
Acanthochromis_polyacanthus(Spiny_chromis)                  PRSDS-ISR----PGSKFN--NLSDDEDEDDSSD-------------------------- 244
Pan_paniscus(Bonobo)                                        PARSDSYSQS-----LKSPLNDMSDDDDDDDSSD-------------------------- 249
Sinocyclocheilus_grahami_1_(Golden-line_barbel)             SHSDS-YSHP------EISSSNISDDDDDDDSSD-------------------------- 245
Lates_calcarifer(Barramundi)                                PRSDS-MSR----LGSKFNSNNLSDDEDEDDSSD-------------------------- 246
Sinocyclocheilus_grahami_2_(Golden-line_barbel)             SHSDS-YSR------SEFSSNNISDDDDDDDSSD-------------------------- 245
Carlito_syrichta(Philippine_tarsier)                        PTRSDSYSQS-----LKSPLNDVSDDDDDDDSSD-------------------------- 249
Canis_lupus(Dingo)                                          PARSDSYSQS-----LKSPLNDVSDDDDDDDSSD-------------------------- 249
Cyclopterus_lumpus(Lumpfish)                                PRSDS-LSR----LGSKFNN--LSDDDDEDDSSD-------------------------- 244
Sander_lucioperca(Zander)                                   QRSDS-ISR----LGSKFNN--LSDDDDDDDSSD-------------------------- 244
Nothobranchius_furzeri(Turquoise_killifish)                 PH-DS-ISR----PTSNFNNNTLSDDEDDDNSSD-------------------------- 245
Monodon_monoceros(Narwhal)                                  PTRSDSYSQS-----LKSTLNDVSDDDDDDDSSD-------------------------- 249
Cervus_elaphus_1_(Yarkand_deer)                             PARSDSYSQS-----LKPPLNDVSDDDDDDDSSD-------------------------- 249
Cervus_elaphus_2_(Yarkand_deer)                             ARSDS-YSQS-----LKPPLNDVSDDDSSD------------------------------ 233
Balaenoptera_musculus(Blue_whale)                           PTRSDSYSQS-----LKSPLNDVSDDDDDDDSSD-------------------------- 249
Leptobrachium_leishanense(Leishan_spiny_toad)               GRSDS-LSSS---PK---FGHNLSDNDDDDDSSD-------------------------- 247
Naja_naja(Indian_cobra)                                     PTRSDSFSQS-----PKPPLNDISDDDDDDDSSD-------------------------- 249
Phocoena_sinus(Vaquita)                                     PTRSDSYSQS-----LKSTLNDVSDDDDDDDSSD-------------------------- 249
Sciurus_vulgaris_1_(Eurasian_red_squirrel)                  PTRSDSYSQS-----SKSPLNDVSDDDDDDDSSD-------------------------- 249
Oncorhynchus_kisutch(Coho_salmon)                           PHSDS-YSRS------GSKFNNVSDDEDEEDSSD-------------------------- 254
Sciurus_vulgaris_2_(Eurasian_red_squirrel)                  PTRSDSYSQS-----SKSPLNNVSDDDDDDSSD--------------------------- 248
Terrapene_carolina(Three-toed_box_turtle)                   PTRSDSYSQS-----PKSPLNDVSDDDDDDDSSD-------------------------- 249
```

```
Salmo_salar_1_(Atlantic_salmon)                       PRSDS-YSRS------GSKFNNVSDDEDEEDSSD-------------------------- 248
Catagonus_wagneri(Chacoan_peccary)                    PTRSDSYSQS-----LKAPLNDVSDDDDDDDSSD-------------------------- 249
Pseudonaja_textilis(Eastern_brown_snake)              PTRSDSFSQS-----PKPPLNDISDDDDDDDSSD-------------------------- 249
Tetraodontidae(Fugu)                                  PRSDS-LSR----PGSKFNTSNLSDDDDDDDSSD-------------------------- 246
Moschus_moschiferus(Siberian_musk_deer)               PARSDSYSQS-----LKPPLNDVSDDDDDDDSSD-------------------------- 249
Equus_caballus(Horse)                                 PPRSDTYSQS-----LKSPLNDVSDDDDDDDSSD-------------------------- 249
Salmo_salar_2_(Atlantic_salmon)                       PRSDS-YSRS------GSKFNNVSDDEDEEDSSD-------------------------- 248
Larimichthys_crocea(Large_yellow_croaker)             PRSDS-ISR----PGSKFN--NLSDDDDEDDSSD-------------------------- 244
Cottoperca_gobio(Channel_bull_blenny)                 PRSDS-ITR----LGSKFN--NLSDDDDEDDSSD-------------------------- 242
Xenopus_tropicalis(Western_clawed_frog)               SRSDS-FSHP---SK---SKNNLSDDDDDEDSSD-------------------------- 248
Sus_scrofa(Pig)                                       PTRSDSYSQS-----LKSPLNDVSDDDDDDDSSD-------------------------- 249
Erpetoichthys_calabaricus(Reedfish)                   TRSDS-YSRA-----SKFSTNNMSDDDDDDDSSD-------------------------- 249
Sparus_aurata(Gilt-head_bream)                        PRSDS-ISR----PGSKFNSNNLSDDDDEDDSSD-------------------------- 246
Struthio_camelus(Ostrich)                             STRSDSYSQS-----PKSPLNDVSDDDEDEDSSD-------------------------- 249
Salmo_trutta_1_(Brown_trout)                          PRSDS-YSRS------GSKFNNVSDDEDEEDSSD-------------------------- 255
Panthera_leo(Lion)                                    PTRSDSYSQS-----LKSPLNDVSDDDDDDDSSD-------------------------- 249
Camelus_dromedarius(Arabian_camel)                    PARSDSYSQS-----LKSPLNDVSDDDDDDDSSD-------------------------- 249
Denticeps_clupeoides(Denticle_herring)                PRSDS-YSRP----RSNFGSNNVSEDDDDDDSSD-------------------------- 247
Ornithorhynchus_anatinus(Platypus)                    PTRSDPYSQS-----PKSPLNNTSDDDDDDSSSD-------------------------- 249
Macaca_mulatta(Macaque)                               PARSDSYSQS-----LKSPLNDMSDDDDDDDSSD-------------------------- 249
Oreochromis_niloticus(Nile_tilapia)                   ARSDS-ITR-----GSKFHSNNLSDDDDDDDSSD-------------------------- 245
Monodelphis_domestica(Gray_short-tailed_opossums)     PTRSDSYSQS-----PKS-LNNVSDDEDDEDSSD-------------------------- 543
Cyprinus_carpio_1_(Common_carp)                       --SDS-YSH------SEISGNNISDDDDDDDSSD-------------------------- 237
Callithrix_jacchus(Common_marmoset)                   PARSDSYSQS-----LKSPLNDMSDDDEDDDSSD-------------------------- 249
Cyprinus_carpio_2_(Common_carp)                       ARSDS-YSR------SEFSSNNISDDDDDDDSSD-------------------------- 245
Scleropages_formosus(Asian_arowana)                   PRSDS-YSRS---S-SRFPGSNVSEDEEEEDSSD-------------------------- 250
Oryzias_sinensis(Chinese_rice_fish)                   PCSDS-NNW----SESRYNNNHLSDDEDEDDSSD-------------------------- 246
Clupea_harengus(Atlantic_herring)                     GRPDP-YGRS--------PGNNVSDDDDEDDSSD-------------------------- 244
Gopherus_evgoodei(Goode's_thornscrub_tortoise)        PTRSDSYSQS-----PKSPLNDVSDDDDDDDSSD-------------------------- 249
Physeter_macrocephalus(Sperm_whale)                   PARSDSYSRS-----LKSPLNDVSDDDDDDDSSD-------------------------- 249
Laticauda_laticaudata(blue-lipped_sea_krait)          PTRSDSFSQS-----PKPPLNDISDDDDDDDSSD-------------------------- 249
Podarcis_muralis(Common_wall_lizard)                  PTRSDSYSHP-----PKLPLNDMSDDDDDDDSSD-------------------------- 249
Delphinapterus_leucas(Beluga_whale)                   PTRSDSYSQS-----LKSTLNDVSDDDDDDDSSD-------------------------- 249
Oncorhynchus_tshawytscha_1_(Chinook_salmon)           PRSDS-YSRS------GSKFNNVSDDEDEEDSSD-------------------------- 307
Betta_splendens(Siamese_fighting_fish)                PRSEC-ISR----PGSKFNNDNLTDEEEEDDSSD-------------------------- 277
Oncorhynchus_mykiss_1_(Rainbow_trout)                 S-HVT-WSTY------SFQYFR-------------------------------------- 235
Oncorhynchus_mykiss_2_(Rainbow_trout)                 PRSDS-YSRS------GSKFNNVSDDEDEEDSSD-------------------------- 248
Salmo_trutta_2_(Brown_trout)                          PRSDS-YSRS------GSKFNNVSDDEDEEDSSD-------------------------- 248
Dicentrarchus_labrax(European_bass)                   PRSDS-IGR----PVSKFNSNNLSDDDDEDDSSD-------------------------- 246
Oryzias_javanicus(Javanese_Ricefish)                  PRSDS-NNW----AESRYNNNHLSDDEDEDDSSD-------------------------- 246
Salmo_salar_3_(Atlantic_salmon)                       L------GKS------NFFF---------------------------------------- 229
Myripristis_murdjan(Pinecone_soldierfish)             PRFDS-ISR----TGSKFNSNNVSDDEDEDDSSD-------------------------- 248
Sarcophilus_harrisii(Tasmanian_devil)                 PTRSDSYSQS-----TKSPLNNVSDDEDDEDSSD-------------------------- 249
Carassius_auratus_1_(Goldfish)                        SRSDS-L----------LSSNNISDDDDDDDSSD-------------------------- 241
Oncorhynchus_tshawytscha_2_(Chinook_salmon)           PRSDS-YSRS------GSKFNNVSDDEDEEDSSD-------------------------- 253
Carassius_auratus_2_(Goldfish)                        --SGS-YTH------LEISGNNISDDDDDDDSSD-------------------------- 237
Mus_musculus(Mouse)                                   PTRSDSYSQS-----LKSPLNDASDDDDDDDSSD-------------------------- 249
Gadus_morhua(Atlantic_cod)                            QRADS-FGR----QGFN-----LSDDEDEDDSSD-------------------------- 243
Drosophila_melanogaster(Fruit_fly)                    -GEDS-AAATGAASGNKLNT-TAGDSSNDEDSDEETASPGGE-SHDEPRFYGDNSVLSAV 276
Ailuropoda_melanoleuca(Giant_panda)                   PTRSDSHSQS-----LKSPLNDVSDDDDDDDSSD-------------------------- 249
Mastacembelus_armatus(Zig-zag_eel)                    PRSDS-ISR----PGSKFHSNNLSDDEDEDDSSD-------------------------- 246
```

```
Macaca_fascicularis(Crab-eating_macaque)        PARSDSYSQS-----LKSPLNDMSDDDDDDDSSD-------------------------- 249
Anabas_testudineus(Climbing_perch)              PRSDC-ISR------SKFNSNNLSDDEDEDDSSD-------------------------- 244
Felis_catus(Cat)                                PTRSDSYSQS-----LKSPLNDVSDDDDDDDSSD-------------------------- 249
```
