# Supplementary material for: The PH Domain and C-Terminal polyD Motif of Phafin2 Exhibit a Unique Concurrence in Animals
Source: Membranes (Basel). 2022 Jul 7;12(7):696. doi: 10.3390/membranes12070696 (PMC9324892; doi:10.3390/membranes12070696)
Supplement: Supplementary file 1 [file membranes-12-00696-s001.zip › Supplementary File S3.pdf]

**Supplementary File S3:** Details of different modules found in bacteria, archaea, fungi, plants, nonhuman mammals, and humans.

## Bacteria

| *No* | *Accession No* | *Modules* |
|---|---|---|
| 1 | tr\|A0A1Y6CWR2\|A0A1Y6CWR2_9GAMM | FYVE |
| 2 | tr\|A0A4Q5XUS2\|A0A4Q5XUS2_9DELT | NO |
| 3 | tr\|A0A0A8E4G1\|A0A0A8E4G1_9GAMM | FYVE |
| 4 | tr\|A0A2E9XWQ4\|A0A2E9XWQ4_9RICK | VHS, FYVE |
| 5 | tr\|A0A0G2ZPZ8\|A0A0G2ZPZ8_9DELT | FYVE |
| 6 | tr\|A0A5C6VDZ7\|A0A5C6VDZ7_9BURK | FYVE |
| 7 | tr\|A0A2E9LIA3\|A0A2E9LIA3_9CHLR | FYVE, |
| 8 | tr\|A0A2E4CAT1\|A0A2E4CAT1_9ACTN | FYVE, PI3_PI4_Kinase |
| 9 | tr\|A0A4Q6X9R2\|A0A4Q6X9R2_9BURK | FYVE |
| 10 | tr\|A0A158KI31\|A0A158KI31_9BURK | FYVE |
| 11 | tr\|A0A2W4SQ66\|A0A2W4SQ66_9GAMM | FYVE |
| 12 | tr\|A0A5J6QPA5\|A0A5J6QPA5_9PSED | FYVE |
| 13 | tr\|A0A561P8H2\|A0A561P8H2_XANCT | FYVE |
| 14 | tr\|A0A2E8LZD9\|A0A2E8LZD9_9BACT | FYVE, PI3_PI4_kinase |
| 15 | tr\|A0A4R7ALI0\|A0A4R7ALI0_9GAMM | FYVE |
| 16 | tr\|A0A3L7RST6\|A0A3L7RST6_9BACT | FYVE |
| 17 | tr\|A0A2E0WHK0\|A0A2E0WHK0_9CHLR | FYVE, PI3_PI4_kinase |
| 18 | tr\|A0A1Z9KZ67\|A0A1Z9KZ67_9SYNE | FYVE |
| 19 | tr\|A0A2E9LIE3\|A0A2E9LIE3_9CHLR | FYVE, PI3_PI4_kinase |
| 20 | tr\|A0A3S3EH81\|A0A3S3EH81_9PSED | FYVE |
| 21 | tr\|A0A0F4XPH7\|A0A0F4XPH7_9PSED | FYVE |
| 22 | tr\|A0A657PDX6\|A0A657PDX6_9PSED | FYVE |
| 23 | tr\|G8Q597\|G8Q597_PSEF3 | FYVE |
| 24 | tr\|A0A2E0E560\|A0A2E0E560_9RHOB | PI3_PI4_kinase |
| 25 | tr\|A0A0C2EFB1\|A0A0C2EFB1_9PSED | FYVE |
| 26 | tr\|A0A2E6VXV8\|A0A2E6VXV8_9PROT | FYVE |
| 27 | tr\|A0A2D7RET3\|A0A2D7RET3_9GAMM | PI3_PI4_kinase |
| 28 | tr\|A0A4R7L8D8\|A0A4R7L8D8_9GAMM | FYVE |

| | | |
|---|---|---|
| 29 | tr\|A0A4R3YS80\|A0A4R3YS80_9GAMM | FYVE |
| 30 | tr\|A0A4Q3E3P0\|A0A4Q3E3P0_9RICK | FYVE |
| 31 | tr\|A0A0Q9PCH9\|A0A0Q9PCH9_9GAMM | FYVE |
| 32 | tr\|A0A2D5QHX0\|A0A2D5QHX0_9GAMM | FYVE |
| 33 | tr\|A0A239KKI8\|A0A239KKI8_9PSED | FYVE, FYVE |
| 34 | tr\|A0A2E8CGN4\|A0A2E8CGN4_9DELT | FYVE |
| 35 | tr\|A0A6I1NH92\|A0A6I1NH92_9PSED | FYVE, FYVE |
| 36 | tr\|A0A1E3GCQ5\|A0A1E3GCQ5_9GAMM | FYVE |
| 37 | tr\|A0A2A8BFD9\|A0A2A8BFD9_9BACI | NO |
| 38 | tr\|A0A484ZT69\|A0A484ZT69_9GAMM | DsbB |
| 39 | tr\|A0A2M7G853\|A0A2M7G853_9BACT | Peptidase_S9 |
| 40 | tr\|A0A2C5DIR4\|A0A2C5DIR4_9BACI | NO |

**Archaea**

| *No* | *Accession No* | *Modules* |
|---|---|---|
| 1 | tr\|A0A482RXE8\|A0A482RXE8_9ARCH | RhoGEF, PH, FYVE |
| 2 | tr\|A0A482S4E7\|A0A482S4E7_9ARCH | FYVE |
| 3 | tr\|A0A482RTS6\|A0A482RTS6_9ARCH | FYVE |
| 4 | tr\|A0A482UR88\|A0A482UR88_9ARCH | FYVE |
| 5 | tr\|A0A482R0G1\|A0A482R0G1_9EURY | FYVE |
| 6 | tr\|A0A482RYJ3\|A0A482RYJ3_9ARCH | FYVE |
| 7 | tr\|A0A482SL25\|A0A482SL25_9ARCH | WW, FYVE |
| 8 | tr\|A0A482SVI4\|A0A482SVI4_9ARCH | FYVE |
| 9 | tr\|A0A482ST03\|A0A482ST03_9ARCH | FYVE |
| 10 | tr\|A0A482R4B7\|A0A482R4B7_9EURY | FYVE |
| 11 | tr\|A0A482SVF7\|A0A482SVF7_9ARCH | FYVE, Cpn60_TCP1 |
| 12 | tr\|A0A482SZF2\|A0A482SZF2_9ARCH | Lipase_3, FYVE |
| 13 | tr\|A0A482RGE2\|A0A482RGE2_9ARCH | FYVE |
| 14 | tr\|A0A482RTU5\|A0A482RTU5_9ARCH | FYVE, Ank_2 |
| 15 | tr\|A0A482SG35\|A0A482SG35_9ARCH | FYVE |
| 16 | tr\|A0A482RW94\|A0A482RW94_9ARCH | FYVE |
| 17 | tr\|A0A482RV05\|A0A482RV05_9ARCH | FYVE |
| 18 | tr\|A0A482RVR9\|A0A482RVR9_9ARCH | FYVE |
| 19 | tr\|A0A482SAN9\|A0A482SAN9_9ARCH | FYVE |
| 20 | tr\|A0A482RAR6\|A0A482RAR6_9ARCH | FYVE, Ank_4 |
| 21 | tr\|A0A482UQL2\|A0A482UQL2_9ARCH | FYVE |
| 22 | tr\|A0A482V091\|A0A482V091_9ARCH | FYVE |
| 23 | tr\|A0A482S307\|A0A482S307_9ARCH | FYVE |

| | | |
|---|---|---|
| 24 | tr\|A0A482SRA1\|A0A482SRA1_9ARCH | PH, Ank_2, Ank_2 |
| 25 | tr\|A0A482RXE5\|A0A482RXE5_9ARCH | FYVE |
| 26 | tr\|A0A482SVG8\|A0A482SVG8_9ARCH | FYVE |
| 27 | tr\|A0A482UVH9\|A0A482UVH9_9ARCH | FYVE |
| 28 | tr\|A0A482RQW7\|A0A482RQW7_9ARCH | FYVE |
| 29 | tr\|A0A3A5UT62\|A0A3A5UT62_9EURY | CARDB |
| 30 | tr\|A0A482UPW1\|A0A482UPW1_9ARCH | Sec7 |
| 31 | tr\|A0A7C5RN86\|A0A7C5RN86_9ARCH | HD, mCpol |
| 32 | tr\|A0A7C4ZF49\|A0A7C4ZF49_9ARCH | HD, mCpol |
| 33 | tr\|A0A524F3J3\|A0A524F3J3_9ARCH | dNK |
| 34 | tr\|A0A7J2X8Y9\|A0A7J2X8Y9_9EURY | zf-HYPE, zf-HYPE,Sua5_yciO_yrdC, HYPE_C, TsaD |
| 35 | tr\|A0A482RV58\|A0A482RV58_9ARCH | PH |
| 36 | tr\|A0A2E8V4G5\|A0A2E8V4G5_9EURY | FG-GAP, TSP_3 |
| 37 | tr\|A0A7K4DTG4\|A0A7K4DTG4_9ARCH | Diphthami_syn_2 |
| 38 | tr\|H6Q7G6\|H6Q7G6_PYROT | zf-HIT |
| 39 | tr\|A0A7J2XB36\|A0A7J2XB36_9EURY | Prok-RING_4 |
| 40 | tr\|A0A662LIL0\|A0A662LIL0_9EURY | Prok-RING_4 |
| 41 | tr\|A0A7L4PAM3\|A0A7L4PAM3_9CREN | zf-HIT |
| 42 | tr\|A4WL18\|A4WL18_PYRAR | zf-HIT |
| 43 | tr\|A0A2D6X683\|A0A2D6X683_9ARCH | NO |
| 44 | tr\|A0A151E9R7\|A0A151E9R7_9EURY | Sua5_yciO_yrdC |
| 45 | tr\|A0A497EKV5\|A0A497EKV5_9ARCH | zinc_ribbon_2 |
| 46 | tr\|A0A482SQW3\|A0A482SQW3_9ARCH | FYVE |
| 47 | tr\|A0A2E5RC13\|A0A2E5RC13_9EURY | Ribosomal_L37ae |
| 48 | tr\|A0A2E9DYS3\|A0A2E9DYS3_9EURY | VCBS |
| 49 | tr\|A0A524DJL4\|A0A524DJL4_9ARCH | dNK |
| 50 | tr\|A0A497HLC9\|A0A497HLC9_9EURY | HATPase_c |
| 51 | tr\|A0A7C4S6U6\|A0A7C4S6U6_9ARCH | Ribosomal_L34e |
| 52 | tr\|A0A7J4LRK9\|A0A7J4LRK9_9ARCH | DZR |
| 53 | tr\|A0A7K4IJU2\|A0A7K4IJU2_9ARCH | FYVE |
| 54 | tr\|A0A2D9NJM7\|A0A2D9NJM7_9EURY | FG-GAP |
| 55 | tr\|A0A5A7S0K7\|A0A5A7S0K7_9EURY | PIN_6 |
| 56 | tr\|A0A202E9J8\|A0A202E9J8_9EURY | AAT |
| 57 | tr\|A0A7J4TTH8\|A0A7J4TTH8_9ARCH | DZR |
| 58 | tr\|A0A2E6BWJ1\|A0A2E6BWJ1_9EURY | DUF4428 |
| 59 | tr\|A0A7J4I1H7\|A0A7J4I1H7_9ARCH | DZR |
| 60 | tr\|A0A7C6DZH7\|A0A7C6DZH7_9ARCH | mCpol |
| 61 | tr\|A0A662JAF1\|A0A662JAF1_9CREN | Prok-RING_4 |
| 62 | tr\|A0A151ENE1\|A0A151ENE1_9EURY | Sua5_yciO_yrdC |
| 63 | tr\|A0A2U3CK81\|A0A2U3CK81_HEIAB | Topoisom_bac |
| 64 | tr\|A0A2U9ILS6\|A0A2U9ILS6_9CREN | RNA_POL_M_15KD |
| 65 | tr\|A0A1V4YZZ9\|A0A1V4YZZ9_9EURY | NO |
| 66 | tr\|A0A0X8UZZ7\|A0A0X8UZZ7_9EURY | HypA |

| | | |
|---|---|---|
| 67 | tr\|A0A1H6TIC6\|A0A1H6TIC6_9EURY | Ribosomal_L40e |
| 68 | tr\|A0A7C0UA16\|A0A7C0UA16_9ARCH | zf-B_box |

**Fungi**

| *No* | *Accession No* | *Modules* |
|---|---|---|
| 1 | tr\|A0A2Z6RA14\|A0A2Z6RA14_9GLOM | RhoGEF, PH, FYVE |
| 2 | tr\|A0A397VBS0\|A0A397VBS0_9GLOM | RhoGEF, PH, FYVE |
| 3 | tr\|A0A015IGQ3\|A0A015IGQ3_RHIIW | RhoGEF, PH, FYVE |
| 4 | tr\|A0A397TNA4\|A0A397TNA4_9GLOM | RhoGEF, PH, FYVE |
| 5 | tr\|A0A015KA47\|A0A015KA47_RHIIW | RhoGEF, PH, FYVE |
| 6 | tr\|A0A015IQ23\|A0A015IQ23_RHIIW | RhoGEF, PH, FYVE |
| 7 | tr\|A0A2I1G6N8\|A0A2I1G6N8_9GLOM | RhoGEF, PH, FYVE |
| 8 | tr\|A0A2I1F0G8\|A0A2I1F0G8_9GLOM | RhoGEF, PH, FYVE |
| 9 | tr\|U9SXZ4\|U9SXZ4_RHIID | RhoGEF, PH, FYVE |
| 10 | tr\|A0A372RFQ0\|A0A372RFQ0_9GLOM | RhoGEF, PH, FYVE |
| 11 | tr\|A0A4P9YZL8\|A0A4P9YZL8_9FUNG | RhoGEF, PH, FYVE |
| 12 | tr\|A0A4V1IX88\|A0A4V1IX88_9FUNG | RhoGEF, PH, FYVE |
| 13 | tr\|A0A4P9ZP44\|A0A4P9ZP44_9FUNG | RhoGEF, PH, FYVE |
| 14 | tr\|A0A0L0HGC7\|A0A0L0HGC7_SPIPD | RhoGEF, PH, FYVE |
| 15 | tr\|A0A177WRZ8\|A0A177WRZ8_BATDL | RhoGEF |
| 16 | tr\|F4P650\|F4P650_BATDJ | RhoGEF |
| 17 | tr\|A0A1Y1X2T5\|A0A1Y1X2T5_9FUNG | RhoGEF, PH, FYVE |
| 18 | tr\|A0A507FBG2\|A0A507FBG2_9FUNG | RhoGEF, FYVE |
| 19 | tr\|A0A1Y1X1V7\|A0A1Y1X1V7_9FUNG | RhoGEF, PH, FYVE |
| 20 | tr\|A0A4V1IY93\|A0A4V1IY93_9FUNG | RhoGEF, FYVE |
| 21 | tr\|A0A1Y1X677\|A0A1Y1X677_9FUNG | RhoGEF, PH, FYVE |
| 22 | tr\|A0A137P479\|A0A137P479_CONC2 | RhoGEF, PH, FYVE |
| 23 | tr\|A0A507EFA4\|A0A507EFA4_9FUNG | RhoGEF, PH, FYVE, FYVE |
| 24 | tr\|A0A507CXB0\|A0A507CXB0_9FUNG | RhoGEF, FYV |
| 25 | tr\|A0A1S8W822\|A0A1S8W822_9FUNG | RhoGEF, FYVE |
| 26 | tr\|A0A397IVX3\|A0A397IVX3_9GLOM | RhoGEF, PH, FYVE |
| 27 | tr\|A0A139AK54\|A0A139AK54_GONPJ | NO |
| 28 | tr\|A0A4P9WLC0\|A0A4P9WLC0_9FUNG | RhoGEF, FYVE |
| 29 | tr\|A0A1Y1WA63\|A0A1Y1WA63_9FUNG | RhoGEF, FYVE |
| 30 | tr\|A0A0E9ND18\|A0A0E9ND18_SAICN | RhoGEF, FYVE |
| 31 | tr\|A0A2G5BFQ4\|A0A2G5BFQ4_COERN | RhoGEF, FYVE |
| 32 | tr\|A0A0W4ZGN5\|A0A0W4ZGN5_PNEJ7 | RhoGEF, FYVE |

| | | |
|---|---|---|
| 33 | tr\|A0A167MKJ0\|A0A167MKJ0_CALVF | RhoGEF, FYVE |
| 34 | tr\|A0A165DP15\|A0A165DP15_9BASI | RhoGEF, FYVE |
| 35 | tr\|M5FY58\|M5FY58_DACPD | RhoGEF, FYVE |
| 36 | tr\|A0A1Y2IJD5\|A0A1Y2IJD5_PYCCO | FYVE |
| 37 | tr\|A0A1Y2IIH2\|A0A1Y2IIH2_PYCCO | RhoGEF, FYVE |
| 38 | tr\|A0A1Y2CRY6\|A0A1Y2CRY6_9FUNG | FYVE |
| 39 | tr\|M9MDT8\|M9MDT8_PSEA3 | RhoGEF, FYVE |
| 40 | tr\|A0A0L0SM81\|A0A0L0SM81_ALLM3 | RhoGEF, FYVE |
| 41 | tr\|A0A1M2VA80\|A0A1M2VA80_TRAPU | RhoGEF, FYVE |
| 42 | tr\|A0A2H3IXF5\|A0A2H3IXF5_WOLCO | RhoGEF, FYVE |
| 43 | tr\|A0A4Q9MH65\|A0A4Q9MH65_9APHY | RhoGEF, FYVE |
| 44 | tr\|A0A5E3XNR7\|A0A5E3XNR7_9AGAM | RhoGEF, FYVE |
| 45 | tr\|A0A5C3FP20\|A0A5C3FP20_PSEA2 | RhoGEF, FYVE |
| 46 | tr\|A0A081CEI2\|A0A081CEI2_PSEA2 | RhoGEF, FYVE |
| 47 | tr\|K5WQA6\|K5WQA6_PHACS | RhoGEF, FYVE |
| 48 | tr\|A0A0H2S726\|A0A0H2S726_9AGAM | RhoGEF, FYVE |
| 49 | tr\|A0A165Z002\|A0A165Z002_9AGAM | RhoGEF, FYVE |
| 50 | tr\|A0A481SFP1\|A0A481SFP1_9BASI | RhoGEF, PH, FYVE |
| 51 | tr\|A0A1Y1YYR8\|A0A1Y1YYR8_9FUNG | PH, FYVE |
| 52 | tr\|A0A4Q9NS61\|A0A4Q9NS61_9APHY | RhoGEF, FYVE |
| 53 | tr\|A0A4Q9Q5Q4\|A0A4Q9Q5Q4_9APHY | RhoGEF, FYVE |
| 54 | tr\|A0A5C3NHQ1\|A0A5C3NHQ1_9AGAM | RhoGEF, FYVE |
| 55 | tr\|A0A481SI77\|A0A481SI77_9BASI | RhoGEF, PH, FYVE |
| 56 | tr\|S8FM81\|S8FM81_FOMPI | RhoGEF, FYVE |
| 57 | tr\|A0A2G8SBX2\|A0A2G8SBX2_9APHY | RhoGEF, FYVE |
| 58 | tr\|A0A137NR99\|A0A137NR99_CONC2 | FYVE |
| 59 | tr\|A0A1Y1Z786\|A0A1Y1Z786_9FUNG | FYVE |
| 60 | tr\|A0A5C3ERJ4\|A0A5C3ERJ4_9BASI | RhoGEF, PH, FYVE |
| 61 | tr\|A0A165BJR9\|A0A165BJR9_9APHY | RhoGEF, FYVE |
| 62 | tr\|A0A164UM97\|A0A164UM97_9AGAM | RhoGEF, FYVE |
| 63 | tr\|A0A0D7AXV4\|A0A0D7AXV4_9AGAR | RhoGEF, FYVE |
| 64 | tr\|A0A6A6HW15\|A0A6A6HW15_9PLEO | VHS, FYVE |
| 65 | tr\|A0A165L4G7\|A0A165L4G7_9APHY | RhoGEF, FYVE |
| 66 | tr\|A0A2N8UMQ8\|A0A2N8UMQ8_9BASI | RhoGEF, PH, FYVE |
| 67 | tr\|V5GIZ1\|V5GIZ1_KALBG | RhoGEF, PH, FYVE |
| 68 | tr\|A0A2T2PC27\|A0A2T2PC27_CORCC | VHS, FYVE |
| 69 | tr\|A0A0L0HIX4\|A0A0L0HIX4_SPIPD | FYVE |
| 70 | tr\|A0A5C2U016\|A0A5C2U016_9APHY | RhoGEF, FYVE |
| 71 | tr\|A0A5C2SLU6\|A0A5C2SLU6_9APHY | RhoGEF, FYVE |

| | | |
|---|---|---|
| 72 | tr\|A0A2R6PJ56\|A0A2R6PJ56_9APHY | RhoGEF, FYVE |
| 73 | tr\|A0A074RVC0\|A0A074RVC0_9AGAM | RhoGEF, FYVE |
| 74 | tr\|X8IWG9\|X8IWG9_9AGAM | RhoGEF, FYVE |
| 75 | tr\|A0A067Q7F2\|A0A067Q7F2_9AGAM | RhoGEF, FYVE |
| 76 | tr\|E6ZVA4\|E6ZVA4_SPORE | RhoGEF, PH, FYVE |
| 77 | tr\|A0A3N4I9X6\|A0A3N4I9X6_ASCIM | VHS, FYVE |
| 78 | tr\|A0A0C3NH87\|A0A0C3NH87_PHLGI | RhoGEF, FYVE |
| 79 | tr\|A0A166A0Y0\|A0A166A0Y0_9AGAM | RhoGEF, FYVE |
| 80 | tr\|A0A0F7RZL7\|A0A0F7RZL7_9BASI | RhoGEF, PH, FYVE |
| 81 | tr\|A0A6A5VUJ0\|A0A6A5VUJ0_9PLEO | VHS, FYVE |
| 82 | tr\|A0A6G6FQD5\|A0A6G6FQD5_9APHY | RhoGEF, FYVE |
| 83 | tr\|A0A0B7F915\|A0A0B7F915_THACB | RhoGEF, FYVE |
| 84 | tr\|Q8NJX4\|Q8NJX4_USTMD | RhoGEF, PH, FYVE |
| 85 | tr\|R9PB35\|R9PB35_PSEHS | RhoGEF, FYVE |
| 86 | tr\|A0A077R837\|A0A077R837_9BASI | RhoGEF, PH, FYVE |
| 87 | tr\|A0A0D1E146\|A0A0D1E146_USTMA | RhoGEF, PH, FYVE |
| 88 | tr\|A0A1K0FWK6\|A0A1K0FWK6_9BASI | RhoGEF, FYVE |
| 89 | tr\|A0A4U7KNT8\|A0A4U7KNT8_9BASI | RhoGEF, PH, FYVE |
| 90 | tr\|A0A4T0IBV4\|A0A4T0IBV4_WALIC | RhoGEF, FYVE |
| 91 | tr\|R9AV38\|R9AV38_WALI9 | RhoGEF, FYVE |
| 92 | tr\|A0A4T0J9U8\|A0A4T0J9U8_WALIC | RhoGEF, FYVE |
| 93 | tr\|I2FM96\|I2FM96_USTH4 | RhoGEF, FYVE |
| 94 | tr\|A0A177BYI1\|A0A177BYI1_9PLEO | VHS, FYVE |
| 95 | tr\|A0A5C3M448\|A0A5C3M448_9AGAR | RhoGEF, FYVE |
| 96 | tr\|A0A0W0G1I7\|A0A0W0G1I7_9AGAR | RhoGEF, FYVE |
| 97 | tr\|V2X232\|V2X232_MONRO | RhoGEF, FYVE |
| 98 | tr\|A0A6A5UD68\|A0A6A5UD68_9PLEO | VHS, FYVE |
| 99 | tr\|A0A507ES66\|A0A507ES66_9FUNG | RhoGEF, PH |
| 100 | tr\|A0A0K6FN13\|A0A0K6FN13_9AGAM | RhoGEF, FYVE |
| 101 | tr\|A0A5M9KV80\|A0A5M9KV80_9PLEO | VHS, FYVE |
| 102 | tr\|A0A2W1CTG6\|A0A2W1CTG6_9PLEO | VHS, FYVE |
| 103 | tr\|A0A6S6V7N3\|A0A6S6V7N3_9PLEO | VHS, FYVE |
| 104 | tr\|B2W9Q9\|B2W9Q9_PYRTR | VHS, FYVE |
| 105 | tr\|A0A316ZS75\|A0A316ZS75_9PLEO | VHS, FYVE |
| 106 | tr\|A0A6A6RWR3\|A0A6A6RWR3_9PLEO | VHS, FYVE |
| 107 | tr\|A0A1Y2AFM9\|A0A1Y2AFM9_9FUNG | VHS, FYVE |
| 108 | tr\|A0A1Y2GJ54\|A0A1Y2GJ54_9FUNG | VHS, FYVE |
| 109 | tr\|A0A316VEC8\|A0A316VEC8_9BASI | RhoGEF, FYVE |
| 110 | tr\|A0A6A5QBT9\|A0A6A5QBT9_AMPQU | VHS, FYVE |

| | | |
|---|---|---|
| 111 | tr\|A0A6A6ZW62\|A0A6A6ZW62_9PLEO | VHS, FYVE |
| 112 | tr\|A0A317XKP1\|A0A317XKP1_9BASI | RhoGEF, PH, FYVE |
| 113 | tr\|A0A5C3F116\|A0A5C3F116_9BASI | RhoGEF, PH, FYVE |
| 114 | tr\|A0A177WUX8\|A0A177WUX8_BATDL | FYVE |
| 115 | tr\|F4P7P0\|F4P7P0_BATDJ | FYVE |
| 116 | tr\|A0A6A6V864\|A0A6A6V864_9PLEO | FYVE |
| 117 | tr\|N1QHA6\|N1QHA6_SPHMS | VHS, FYVE |
| 118 | tr\|A0A2V1DU97\|A0A2V1DU97_9PLEO | VHS, FYVE |
| 119 | tr\|A0A6A6TFR8\|A0A6A6TFR8_9PLEO | VHS, FYVE |
| 120 | tr\|A0A550CL33\|A0A550CL33_9AGAR | RhoGEF, FYVE |
| 121 | tr\|A0A284S9X6\|A0A284S9X6_ARMOS | RhoGEF, FYVE |
| 122 | tr\|A0A0C3Q7C3\|A0A0C3Q7C3_9AGAM | RhoGEF, FYVE |
| 123 | tr\|A0A367KQZ9\|A0A367KQZ9_RHIST | VHS, FYVE |
| 124 | tr\|A0A074VKM0\|A0A074VKM0_9PEZI | VHS, FYVE |
| 125 | tr\|R0ITW9\|R0ITW9_SETT2 | VHS, FYVE |
| 126 | tr\|A0A1Y2EN19\|A0A1Y2EN19_9BASI | RhoGEF, FYVE |
| 127 | tr\|A0A507BWX7\|A0A507BWX7_9FUNG | RhoGEF, Glyco_hydro_47 |
| 128 | tr\|A0A6A6JKH9\|A0A6A6JKH9_9PLEO | VHS, FYVE |
| 129 | tr\|A0A3N4M7C5\|A0A3N4M7C5_9PEZI | VHS, FYVE |
| 130 | tr\|A0A6A5JXY0\|A0A6A5JXY0_9PLEO | VHS, FYVE |
| 131 | tr\|A0A6G1IPA9\|A0A6G1IPA9_9PLEO | VHS, FYVE |
| 132 | tr\|A0A0D7A3M6\|A0A0D7A3M6_9AGAR | RhoGEF, FYVE |
| 133 | tr\|A0A0C3B4B2\|A0A0C3B4B2_9AGAM | RhoGEF, FYVE |
| 134 | tr\|A0A067MUV1\|A0A067MUV1_9AGAM | RhoGEF, FYVE |
| 135 | tr\|A0A0P1BAI6\|A0A0P1BAI6_9BASI | RhoGEF, FYVE |
| 136 | tr\|A0A0D0C6Q8\|A0A0D0C6Q8_9AGAR | RhoGEF, FYVE |
| 137 | tr\|A0A197JU28\|A0A197JU28_9FUNG | RhoGEF, FYVE, FYVE |
| 138 | tr\|A0A178AI31\|A0A178AI31_9PLEO | VHS, FYVE |
| 139 | tr\|A0A316W3E2\|A0A316W3E2_9BASI | RhoGEF, FYVE |
| 140 | tr\|A0A316YEW3\|A0A316YEW3_9BASI | RhoGEF, FYVE |
| 141 | tr\|A0A1Y2GHB3\|A0A1Y2GHB3_9FUNG | RhoGEF, FYVE, FYVE |
| 142 | tr\|A0A6A4GV68\|A0A6A4GV68_9AGAR | FYVE |
| 143 | sp\|O13821\|VPS27_SCHPO | VHS, FYVE |
| 144 | tr\|A0A317T2J4\|A0A317T2J4_9PEZI | VHS, FYVE |
| 145 | tr\|A0A6S6WPJ0\|A0A6S6WPJ0_9PLEO | VHS, FYVE |
| 146 | tr\|E3RQL4\|E3RQL4_PYRTT | VHS, FYVE |
| 147 | tr\|A0A0C9WXY8\|A0A0C9WXY8_9AGAR | VHS, FYVE |
| 148 | tr\|F8P7Z0\|F8P7Z0_SERL9 | VHS, FYVE |
| 149 | tr\|F8Q904\|F8Q904_SERL3 | VHS, FYVE |

| | | |
|---|---|---|
| 150 | tr\|A0A292PL22\|A0A292PL22_9PEZI | VHS, FYVE |
| 151 | tr\|A0A1Y1XKQ0\|A0A1Y1XKQ0_9FUNG | VHS, FYVE |
| 152 | sp\|Q0U4Z8\|VPS27_PHANO | VHS, FYVE |
| 153 | tr\|A0A6A6D0R8\|A0A6A6D0R8_9PEZI | VHS, FYVE |
| 154 | tr\|A0A1Y1UY75\|A0A1Y1UY75_9FUNG | Ank_2, Ank_2, FYVE |
| 155 | tr\|A0A6A6QQF5\|A0A6A6QQF5_9PEZI | VHS, FYVE |
| 156 | tr\|A0A168QCU4\|A0A168QCU4_MUCCL | FYVE |
| 157 | tr\|A0A0D2PBI9\|A0A0D2PBI9_HYPSF | RhoGEF, FYVE |
| 158 | tr\|U4LJ46\|U4LJ46_PYROM | VHS, FYVE |
| 159 | tr\|A0A2T6ZAZ1\|A0A2T6ZAZ1_TUBBO | VHS, FYVE |
| 160 | tr\|A0A4U0WTC3\|A0A4U0WTC3_9PEZI | VHS, FYVE |
| 161 | tr\|A0A4S8LNE8\|A0A4S8LNE8_DENBC | RhoGEF, FYVE |
| 162 | tr\|A0A4V1IUA8\|A0A4V1IUA8_9FUNG | VHS, FYVE |
| 163 | tr\|L0P8I6\|L0P8I6_PNEJ8 | FYVE |
| 164 | tr\|R7Z380\|R7Z380_CONA1 | VHS, FYVE |
| 165 | tr\|E4ZXS6\|E4ZXS6_LEPMJ | VHS, FYVE |
| 166 | tr\|A0A168Q840\|A0A168Q840_MUCCL | VHS, FYVE |
| 167 | tr\|A0A0C2S3H8\|A0A0C2S3H8_AMAMU | RhoGEF, FYVE |
| 168 | tr\|A0A0C9N4H5\|A0A0C9N4H5_9FUNG | VHS, FYVE |
| 169 | tr\|R4XHH8\|R4XHH8_TAPDE | VHS, FYVE |
| 170 | tr\|A0A0B7NCZ8\|A0A0B7NCZ8_9FUNG | FYVE |
| 171 | tr\|A0A163F5K2\|A0A163F5K2_DIDRA | VHS, FYVE |
| 172 | tr\|A0A6A6YA67\|A0A6A6YA67_9PEZI | VHS, FYVE |
| 173 | tr\|A0A5C3KRI4\|A0A5C3KRI4_9AGAR | RhoGEF, FYVE |
| 174 | tr\|A0A0C2YUU7\|A0A0C2YUU7_HEBCY | RhoGEF, FYVE |
| 175 | tr\|G4T9B0\|G4T9B0_SERID | RhoGEF, FYVE |
| 176 | tr\|A0A367JD28\|A0A367JD28_RHIAZ | VHS, FYVE |
| 177 | tr\|A0A367JX58\|A0A367JX58_RHIAZ | VHS, FYVE |
| 178 | tr\|A0A0M8MVG5\|A0A0M8MVG5_9BASI | FYVE |
| 179 | tr\|S2JXE4\|S2JXE4_MUCC1 | VHS, FYVE |
| 180 | tr\|A0A286USL7\|A0A286USL7_9AGAM | RhoGEF, FYVE |
| 181 | tr\|A0A067NSG1\|A0A067NSG1_PLEOS | RhoGEF, FYVE |
| 182 | tr\|A0A316ZLR8\|A0A316ZLR8_9BASI | RhoGEF, FYVE |
| 183 | tr\|A0A4T0G0A2\|A0A4T0G0A2_9BASI | RhoGEF, FYVE |
| 184 | tr\|A0A075ARC6\|A0A075ARC6_ROZAC | FYVE |
| 185 | tr\|A0A4V1IZA8\|A0A4V1IZA8_ROZAC | FYVE |
| 186 | tr\|A0A3M7M1P8\|A0A3M7M1P8_9PLEO | VHS, FYVE |
| 187 | tr\|D5GD30\|D5GD30_TUBMM | VHS, FYVE |
| 188 | tr\|A0A1X7RQY2\|A0A1X7RQY2_ZYMTR | VHS, FYVE |

| | | |
|---|---|---|
| 189 | tr\|A0A1Y6LJF9\|A0A1Y6LJF9_ZYMTR | VHS, FYVE |
| 190 | tr\|A0A2H1GBB8\|A0A2H1GBB8_ZYMTR | VHS, FYVE |
| 191 | tr\|F9X9I8\|F9X9I8_ZYMTI | VHS, FYVE |
| 192 | tr\|A0A6A6A070\|A0A6A6A070_9PLEO | VHS, FYVE |
| 193 | tr\|B0DJQ4\|B0DJQ4_LACBS | RhoGEF, FYVE |
| 194 | tr\|A0A369JTG7\|A0A369JTG7_HYPMA | VHS, FYVE |
| 195 | tr\|I4YHW0\|I4YHW0_WALMC | VHS, FYVE |
| 196 | tr\|A0A507CTR3\|A0A507CTR3_9FUNG | VHS, FYVE |
| 197 | tr\|F4RZR0\|F4RZR0_MELLP | RhoGEF, FYVE |
| 198 | tr\|A0A4T0WVM5\|A0A4T0WVM5_9ASCO | VHS, FYVE |
| 199 | tr\|A0A150VI72\|A0A150VI72_9PEZI | VHS, FYVE |
| 200 | tr\|A0A3M6ZNS9\|A0A3M6ZNS9_HORWE | VHS, FYVE |
| 201 | tr\|A0A1Y1XRY0\|A0A1Y1XRY0_9FUNG | PH, FYVE |
| 202 | tr\|A0A074WRI8\|A0A074WRI8_9PEZI | VHS, FYVE |
| 203 | tr\|A0A6A7BU37\|A0A6A7BU37_9PEZI | VHS, FYVE |
| 204 | tr\|K1WTE0\|K1WTE0_TRIAC | VHS, FYVE |
| 205 | tr\|J6EPC0\|J6EPC0_TRIAS | VHS, FYVE |
| 206 | tr\|A0A3N4JEN9\|A0A3N4JEN9_9PEZI | VHS, FYVE |
| 207 | tr\|N1QCW3\|N1QCW3_PSEFD | VHS, FYVE |
| 208 | tr\|M2STQ7\|M2STQ7_COCH5 | VHS, FYVE |
| 209 | tr\|N4X1G1\|N4X1G1_COCH4 | VHS, FYVE |
| 210 | tr\|A0A6A5SBR1\|A0A6A5SBR1_9PLEO | VHS, FYVE |
| 211 | tr\|A0A1Y2LUT7\|A0A1Y2LUT7_EPING | VHS, FYVE |
| 212 | tr\|E2LZL4\|E2LZL4_MONPE | FYVE |
| 213 | tr\|A0A067S9R7\|A0A067S9R7_GALM3 | RhoGEF, FYVE |
| 214 | tr\|L0PFS6\|L0PFS6_PNEJ8 | FYVE |
| 215 | tr\|F4NZK1\|F4NZK1_BATDJ | VHS, FYVE |
| 216 | tr\|A0A3M7BVL5\|A0A3M7BVL5_HORWE | VHS, FYVE |
| 217 | tr\|A0A5J5F2H1\|A0A5J5F2H1_9PEZI | VHS, FYVE |
| 218 | tr\|A0A3M7GF14\|A0A3M7GF14_HORWE | VHS, FYVE |
| 219 | tr\|A0A3M7EI93\|A0A3M7EI93_HORWE | VHS, FYVE |
| 220 | tr\|A0A3M7A400\|A0A3M7A400_HORWE | VHS, FYVE |
| 221 | tr\|A0A162Y6D2\|A0A162Y6D2_MUCCL | FYVE |
| 222 | tr\|A0A4U0TZE6\|A0A4U0TZE6_9PEZI | VHS, FYVE |
| 223 | tr\|A0A3M7DFE3\|A0A3M7DFE3_HORWE | VHS, FYVE |
| 224 | tr\|A0A3M7C5L4\|A0A3M7C5L4_HORWE | VHS, FYVE |
| 225 | tr\|A0A1Z5SW51\|A0A1Z5SW51_HORWE | VHS, FYVE |
| 226 | tr\|A0A3M7IAA8\|A0A3M7IAA8_HORWE | VHS, FYVE |
| 227 | tr\|A0A1Y1ZMB2\|A0A1Y1ZMB2_9PLEO | VHS, FYVE |

| | | |
|---|---|---|
| 228 | tr\|A0A3M7EIE8\|A0A3M7EIE8_HORWE | VHS, FYVE |
| 229 | tr\|A0A3M7FRT0\|A0A3M7FRT0_HORWE | VHS, FYVE |
| 230 | tr\|A0A3M6WMK4\|A0A3M6WMK4_HORWE | VHS, FYVE |
| 231 | tr\|A0A0W4ZU85\|A0A0W4ZU85_PNEJ7 | FYVE |
| 232 | tr\|A0A409Y3I3\|A0A409Y3I3_9AGAR | RhoGEF, FYVE |
| 233 | tr\|A0A1X0QT00\|A0A1X0QT00_RHIZD | VHS, FYVE |
| 234 | tr\|A0A2G4SSF8\|A0A2G4SSF8_RHIZD | VHS, FYVE |
| 235 | tr\|A0A4V4JT84\|A0A4V4JT84_AURPU | VHS, FYVE |
| 236 | tr\|A0A4S9NID1\|A0A4S9NID1_AURPU | VHS, FYVE |
| 237 | tr\|A0A4V4JPE1\|A0A4V4JPE1_AURPU | VHS, FYVE |
| 238 | tr\|A0A4S8U3P8\|A0A4S8U3P8_AURPU | VHS, FYVE |
| 239 | tr\|A0A4T0CLC8\|A0A4T0CLC8_AURPU | VHS, FYVE |
| 240 | tr\|A0A4S8XK24\|A0A4S8XK24_AURPU | VHS, FYVE |
| 241 | tr\|A0A4S9PXJ6\|A0A4S9PXJ6_AURPU | VHS, FYVE |
| 242 | tr\|A0A4S9S1G3\|A0A4S9S1G3_AURPU | VHS, FYVE |
| 243 | tr\|A0A4S9X121\|A0A4S9X121_AURPU | VHS, FYVE |
| 244 | tr\|A0A4S9MIC5\|A0A4S9MIC5_AURPU | VHS, FYVE |
| 245 | tr\|A0A4T0AAT4\|A0A4T0AAT4_AURPU | VHS, FYVE |
| 246 | tr\|A0A4V4LMX5\|A0A4V4LMX5_AURPU | VHS, FYVE |
| 247 | tr\|A0A4S9MD28\|A0A4S9MD28_AURPU | VHS, FYVE |
| 248 | tr\|A0A4S9SZX6\|A0A4S9SZX6_AURPU | VHS, FYVE |
| 249 | tr\|A0A4S8TGC0\|A0A4S8TGC0_AURPU | VHS, FYVE |
| 250 | tr\|A0A4S9BBL0\|A0A4S9BBL0_AURPU | VHS, FYVE |

**Plants**

| ***No*** | ***Accession No*** | ***Modules*** |
|---|---|---|
| 1 | tr\|A0A2Z6RA14\|A0A2Z6RA14_9GLOM | RhoGEF, PH, FYVE |
| 2 | tr\|A0A397VBS0\|A0A397VBS0_9GLOM | RhoGEF, PH, FYVE |
| 3 | tr\|A0A015IGQ3\|A0A015IGQ3_RHIIW | RhoGEF, PH, FYVE |
| 4 | tr\|A0A397TNA4\|A0A397TNA4_9GLOM | RhoGEF, PH, FYVE |
| 5 | tr\|A0A015KA47\|A0A015KA47_RHIIW | RhoGEF, PH, FYVE |
| 6 | tr\|A0A015IQ23\|A0A015IQ23_RHIIW | RhoGEF, PH, FYVE |
| 7 | tr\|A0A2I1G6N8\|A0A2I1G6N8_9GLOM | RhoGEF, PH, FYVE |
| 8 | tr\|A0A2I1F0G8\|A0A2I1F0G8_9GLOM | RhoGEF, PH, FYVE |

| | | |
|---|---|---|
| 9 | tr\|U9SXZ4\|U9SXZ4_RHIID | RhoGEF, PH, FYVE |
| 10 | tr\|A0A372RFQ0\|A0A372RFQ0_9GLOM | RhoGEF, PH, FYVE |
| 11 | tr\|A0A4P9YZL8\|A0A4P9YZL8_9FUNG | RhoGEF, PH, FYVE |
| 12 | tr\|A0A4V1IX88\|A0A4V1IX88_9FUNG | RhoGEF, PH, FYVE |
| 13 | tr\|A0A4P9ZP44\|A0A4P9ZP44_9FUNG | RhoGEF, PH, FYVE |
| 14 | tr\|A0A0L0HGC7\|A0A0L0HGC7_SPIPD | RhoGEF, PH, FYVE |
| 15 | tr\|A0A177WRZ8\|A0A177WRZ8_BATDL | RhoGEF, RhoGEF, FYVE |
| 16 | tr\|F4P650\|F4P650_BATDJ | RhoGEF, FYVE |
| 17 | tr\|A0A1Y1X2T5\|A0A1Y1X2T5_9FUNG | RhoGEF, PH, FYVE |
| 18 | tr\|A0A507FBG2\|A0A507FBG2_9FUNG | RhoGEF, FYVE |
| 19 | tr\|A0A1Y1X1V7\|A0A1Y1X1V7_9FUNG | RhoGEF, FYVE, PH |
| 20 | tr\|A0A4V1IY93\|A0A4V1IY93_9FUNG | RhoGEF, FYVE |
| 21 | tr\|A0A1Y1X677\|A0A1Y1X677_9FUNG | RhoGEF, PH, FYVE |
| 22 | tr\|A0A137P479\|A0A137P479_CONC2 | RhoGEF, PH, FYVE |
| 23 | tr\|A0A507EFA4\|A0A507EFA4_9FUNG | RhoGEF, PH, FYVE, PH |
| 24 | tr\|A0A507CXB0\|A0A507CXB0_9FUNG | RhoGEF, FYVE |
| 25 | tr\|A0A1S8W822\|A0A1S8W822_9FUNG | RhoGEF, FYVE |
| 26 | tr\|A0A397IVX3\|A0A397IVX3_9GLOM | RhoGEF, PH, FYVE |
| 27 | tr\|A0A139AK54\|A0A139AK54_GONPJ | NO |
| 28 | tr\|A0A4P9WLC0\|A0A4P9WLC0_9FUNG | RhoGEF, PH, FYVE |
| 29 | tr\|A0A1Y1WA63\|A0A1Y1WA63_9FUNG | RhoGEF, FYVE |
| 30 | tr\|A0A0E9ND18\|A0A0E9ND18_SAICN | RhoGEF, FYVE, Pex14_N |
| 31 | tr\|A0A2G5BFQ4\|A0A2G5BFQ4_COERN | RhoGEF, PH, FYVE |
| 32 | tr\|A0A0W4ZGN5\|A0A0W4ZGN5_PNEJ7 | RhoGEF, FYVE |
| 33 | tr\|A0A167MKJ0\|A0A167MKJ0_CALVF | RhoGEF, FYVE |
| 34 | tr\|A0A165DP15\|A0A165DP15_9BASI | RhoGEF, FYVE |
| 35 | tr\|M5FY58\|M5FY58_DACPD | RhoGEF, FYVE |
| 36 | tr\|A0A1Y2IJD5\|A0A1Y2IJD5_PYCCO | FYVE |
| 37 | tr\|A0A1Y2IIH2\|A0A1Y2IIH2_PYCCO | RhoGEF, FYVE |
| 38 | tr\|A0A1Y2CRY6\|A0A1Y2CRY6_9FUNG | FYVE |
| 39 | tr\|M9MDT8\|M9MDT8_PSEA3 | RhoGEF, FYVE |
| 40 | tr\|A0A0L0SM81\|A0A0L0SM81_ALLM3 | RhoGEF, FYVE |
| 41 | tr\|A0A1M2VA80\|A0A1M2VA80_TRAPU | RhoGEF, FYVE |
| 42 | tr\|A0A2H3IXF5\|A0A2H3IXF5_WOLCO | RhoGEF, FYVE |
| 43 | tr\|A0A4Q9MH65\|A0A4Q9MH65_9APHY | RhoGEF, FYVE |
| 44 | tr\|A0A5E3XNR7\|A0A5E3XNR7_9AGAM | RhoGEF, FYVE |
| 45 | tr\|A0A5C3FP20\|A0A5C3FP20_PSEA2 | RhoGEF, FYVE |

| | | |
|---|---|---|
| 46 | tr\|A0A081CEI2\|A0A081CEI2_PSEA2 | RhoGEF, FYVE |
| 47 | tr\|K5WQA6\|K5WQA6_PHACS | RhoGEF, FYVE |
| 48 | tr\|A0A0H2S726\|A0A0H2S726_9AGAM | RhoGEF, FYVE |
| 49 | tr\|A0A165Z002\|A0A165Z002_9AGAM | RhoGEF, FYVE |
| 50 | tr\|A0A481SFP1\|A0A481SFP1_9BASI | RhoGEF, PH, FYVE |
| 51 | tr\|A0A1Y1YYR8\|A0A1Y1YYR8_9FUNG | PH, FYVE |
| 52 | tr\|A0A4Q9NS61\|A0A4Q9NS61_9APHY | RhoGEF, FYVE |
| 53 | tr\|A0A4Q9Q5Q4\|A0A4Q9Q5Q4_9APHY | RhoGEF, FYVE |
| 54 | tr\|A0A5C3NHQ1\|A0A5C3NHQ1_9AGAM | RhoGEF, FYVE |
| 55 | tr\|A0A481SI77\|A0A481SI77_9BASI | RhoGEF, PH, FYVE |
| 56 | tr\|S8FM81\|S8FM81_FOMPI | RhoGEF, FYVE |
| 57 | tr\|A0A2G8SBX2\|A0A2G8SBX2_9APHY | RhoGEF, FYVE |
| 58 | tr\|A0A137NR99\|A0A137NR99_CONC2 | FYVE |
| 59 | tr\|A0A1Y1Z786\|A0A1Y1Z786_9FUNG | FYVE |
| 60 | tr\|A0A5C3ERJ4\|A0A5C3ERJ4_9BASI | RhoGEF, PH, FYVE |
| 61 | tr\|A0A165BJR9\|A0A165BJR9_9APHY | RhoGEF, FYVE |
| 62 | tr\|A0A164UM97\|A0A164UM97_9AGAM | RhoGEF, FYVE |
| 63 | tr\|A0A0D7AXV4\|A0A0D7AXV4_9AGAR | RhoGEF, FYVE |
| 64 | tr\|A0A6A6HW15\|A0A6A6HW15_9PLEO | VHS, FYVE, UIM, UIM |
| 65 | tr\|A0A165L4G7\|A0A165L4G7_9APHY | RhoGEF, FYVE |
| 66 | tr\|A0A2N8UMQ8\|A0A2N8UMQ8_9BASI | RhoGEF, PH, FYVE |
| 67 | tr\|V5GIZ1\|V5GIZ1_KALBG | RhoGEF, PH, FYVE |
| 68 | tr\|A0A2T2PC27\|A0A2T2PC27_CORCC | VHS, FYVE, UIM, UIM |
| 69 | tr\|A0A0L0HIX4\|A0A0L0HIX4_SPIPD | FYVE |

| | | |
|---|---|---|
| 70 | tr\|A0A5C2U016\|A0A5C2U016_9APHY | RhoGEF, FYVE |
| 71 | tr\|A0A5C2SLU6\|A0A5C2SLU6_9APHY | RhoGEF, FYVE |
| 72 | tr\|A0A2R6PJ56\|A0A2R6PJ56_9APHY | RhoGEF, FYVE |
| 73 | tr\|A0A074RVC0\|A0A074RVC0_9AGAM | RhoGEF, FYVE |
| 74 | tr\|X8IWG9\|X8IWG9_9AGAM | RhoGEF, FYVE |
| 75 | tr\|A0A067Q7F2\|A0A067Q7F2_9AGAM | RhoGEF, FYVE |
| 76 | tr\|E6ZVA4\|E6ZVA4_SPORE | RhoGEF, PH, FYVE |
| 77 | tr\|A0A3N4I9X6\|A0A3N4I9X6_ASCIM | VHS, FYVE, UIM, UIM |
| 78 | tr\|A0A0C3NH87\|A0A0C3NH87_PHLGI | RhoGEF, FYVE |
| 79 | tr\|A0A166A0Y0\|A0A166A0Y0_9AGAM | RhoGEF, FYVE |
| 80 | tr\|A0A0F7RZL7\|A0A0F7RZL7_9BASI | RhoGEF, PH, FYVE |
| 81 | tr\|A0A6A5VUJ0\|A0A6A5VUJ0_9PLEO | VHS, FYVE, UIM, UIM |
| 82 | tr\|A0A6G6FQD5\|A0A6G6FQD5_9APHY | RhoGEF, FYVE |
| 83 | tr\|A0A0B7F915\|A0A0B7F915_THACB | RhoGEF, FYVE |
| 84 | tr\|Q8NJX4\|Q8NJX4_USTMD | RhoGEF, PH, FYVE |
| 85 | tr\|R9PB35\|R9PB35_PSEHS | RhoGEF, FYVE |
| 86 | tr\|A0A077R837\|A0A077R837_9BASI | RhoGEF, PH, FYVE |

| | | |
|---|---|---|
| 87 | tr\|A0A0D1E146\|A0A0D1E146_USTMA | RhoGEF, PH, FYVE |
| 88 | tr\|A0A1K0FWK6\|A0A1K0FWK6_9BASI | RhoGEF, FYVE |
| 89 | tr\|A0A4U7KNT8\|A0A4U7KNT8_9BASI | RhoGEF, PH, FYVE |
| 90 | tr\|C\|A0A4T0IBV4_WALIC | RhoGEF, PH, FYVE |
| 91 | tr\|R9AV38\|R9AV38_WALI9 | RhoGEF, FYVE |
| 92 | tr\|A0A4T0J9U8\|A0A4T0J9U8_WALIC | RhoGEF, FYVE |
| 93 | tr\|I2FM96\|I2FM96_USTH4 | RhoGEF, FYVE |
| 94 | tr\|A0A177BYI1\|A0A177BYI1_9PLEO | VHS, FYVE, UIM, UIM |
| 95 | tr\|A0A5C3M448\|A0A5C3M448_9AGAR | RhoGEF, FYVE |
| 96 | tr\|A0A0W0G1I7\|A0A0W0G1I7_9AGAR | RhoGEF, FYVE |
| 97 | tr\|V2X232\|V2X232_MONRO | RhoGEF, FYVE |
| 98 | tr\|A0A6A5UD68\|A0A6A5UD68_9PLEO | VHS, FYVE |
| 99 | tr\|A0A507ES66\|A0A507ES66_9FUNG | RhoGEF, PH |
| 100 | tr\|A0A0K6FN13\|A0A0K6FN13_9AGAM | RhoGEF, FYVE |
| 101 | tr\|A0A5M9KV80\|A0A5M9KV80_9PLEO | VHS, FYVE, UIM, UIM |
| 102 | tr\|A0A2W1CTG6\|A0A2W1CTG6_9PLEO | VHS, FYVE, UIM, UIM |
| 103 | tr\|A0A6S6V7N3\|A0A6S6V7N3_9PLEO | VHS, FYVE, UIM, UIM |

| | | |
|---|---|---|
| 104 | tr\|B2W9Q9\|B2W9Q9_PYRTR | VHS, FYVE, UIM, UIM |
| 105 | tr\|A0A316ZS75\|A0A316ZS75_9PLEO | VHS, FYVE, UIM, UIM |
| 106 | tr\|A0A6A6RWR3\|A0A6A6RWR3_9PLEO | VHS, FYVE, UIM, UIM |
| 107 | tr\|A0A1Y2AFM9\|A0A1Y2AFM9_9FUNG | VHS, FYVE, UIM, UIM |
| 108 | tr\|A0A1Y2GJ54\|A0A1Y2GJ54_9FUNG | VHS, FYVE, UIM |
| 109 | tr\|A0A316VEC8\|A0A316VEC8_9BASI | RhoGEF, FYVE |
| 110 | tr\|A0A6A5QBT9\|A0A6A5QBT9_AMPQU | VHS, FYVE, UIM, UIM |
| 111 | tr\|A0A6A6ZW62\|A0A6A6ZW62_9PLEO | VHS, FYVE |
| 112 | tr\|A0A317XKP1\|A0A317XKP1_9BASI | RhoGEF, PH, FYVE |
| 113 | tr\|A0A5C3F116\|A0A5C3F116_9BASI | RhoGEF, PH, FYVE |
| 114 | tr\|A0A177WUX8\|A0A177WUX8_BATDL | FYVE |
| 115 | tr\|F4P7P0\|F4P7P0_BATDJ | FYVE |
| 116 | tr\|A0A6A6V864\|A0A6A6V864_9PLEO | FYVE, UIM, UIM |
| 117 | tr\|N1QHA6\|N1QHA6_SPHMS | VHS, FYVE, UIM, UIM |
| 118 | tr\|A0A2V1DU97\|A0A2V1DU97_9PLEO | VHS, FYVE, UIM, UIM |
| 119 | tr\|A0A6A6TFR8\|A0A6A6TFR8_9PLEO | VHS, FYVE, UIM, UIM |
| 120 | tr\|A0A550CL33\|A0A550CL33_9AGAR | RhoGEF, FYVE |

| | | |
|---|---|---|
| 121 | tr\|A0A284S9X6\|A0A284S9X6_ARMOS | RhoGEF, FYVE |
| 122 | tr\|A0A0C3Q7C3\|A0A0C3Q7C3_9AGAM | RhoGEF, FYVE |
| 123 | tr\|A0A367KQZ9\|A0A367KQZ9_RHIST | VHS, FYVE, UIM, Hrs_helical |
| 124 | tr\|A0A074VKM0\|A0A074VKM0_9PEZI | VHS, FYVE, UIM, UIM |
| 125 | tr\|R0ITW9\|R0ITW9_SETT2 | VHS, FYVE, UIM, UIM |
| 126 | tr\|A0A1Y2EN19\|A0A1Y2EN19_9BASI | RhoGEF, FYVE |
| 127 | tr\|A0A507BWX7\|A0A507BWX7_9FUNG | RhoGEF, Glyco_hydro_47 |
| 128 | tr\|A0A6A6JKH9\|A0A6A6JKH9_9PLEO | VHS, FYVE, UIM, UIM |
| 129 | tr\|A0A3N4M7C5\|A0A3N4M7C5_9PEZI | VHS, FYVE, UIM, UIM |
| 130 | tr\|A0A6A5JXY0\|A0A6A5JXY0_9PLEO | VHS, FYVE, UIM, UIM |
| 131 | tr\|A0A6G1IPA9\|A0A6G1IPA9_9PLEO | VHS, FYVE, UIM, UIM |
| 132 | tr\|A0A0D7A3M6\|A0A0D7A3M6_9AGAR | RhoGEF, FYVE |
| 133 | tr\|A0A0C3B4B2\|A0A0C3B4B2_9AGAM | RhoGEF, FYVE |
| 134 | tr\|A0A067MUV1\|A0A067MUV1_9AGAM | RhoGEF, FYVE |
| 135 | tr\|A0A0P1BAI6\|A0A0P1BAI6_9BASI | RhoGEF, FYVE |
| 136 | tr\|A0A0D0C6Q8\|A0A0D0C6Q8_9AGAR | RhoGEF, FYVE |
| 137 | tr\|A0A197JU28\|A0A197JU28_9FUNG | RhoGEF, FYVE, FYVE |

| | | |
|---|---|---|
| 138 | tr\|A0A178AI31\|A0A178AI31_9PLEO | VHS, FYVE, UIM, UIM |
| 139 | tr\|A0A316W3E2\|A0A316W3E2_9BASI | RhoGEF, FYVE |
| 140 | tr\|A0A316YEW3\|A0A316YEW3_9BASI | RhoGEF, FYVE |
| 141 | tr\|A0A1Y2GHB3\|A0A1Y2GHB3_9FUNG | RhoGEF, FYVE, FYVE |
| 142 | tr\|A0A6A4GV68\|A0A6A4GV68_9AGAR | FYVE |
| 143 | sp\|O13821\|VPS27_SCHPO | VHS, FYVE, UIM, UIM |
| 144 | tr\|A0A317T2J4\|A0A317T2J4_9PEZI | VHS, FYVE, UIM, UIM |
| 145 | tr\|A0A6S6WPJ0\|A0A6S6WPJ0_9PLEO | VHS, FYVE, UIM, UIM |
| 146 | tr\|E3RQL4\|E3RQL4_PYRTT | VHS, FYVE, UIM, UIM |
| 147 | tr\|A0A0C9WXY8\|A0A0C9WXY8_9AGAR | RhoGEF, FYVE |
| 148 | tr\|F8P7Z0\|F8P7Z0_SERL9 | RhoGEF, FYVE |
| 149 | tr\|F8Q904\|F8Q904_SERL3 | RhoGEF, FYVE |
| 150 | tr\|A0A292PL22\|A0A292PL22_9PEZI | VHS, FYVE, UIM, UIM |
| 151 | tr\|A0A1Y1XKQ0\|A0A1Y1XKQ0_9FUNG | VHS, FYVE, UIM, UIM |
| 152 | sp\|Q0U4Z8\|VPS27_PHANO | VHS, FYVE, UIM |
| 153 | tr\|A0A6A6D0R8\|A0A6A6D0R8_9PEZI | VHS, FYVE, UIM, UIM |
| 154 | tr\|A0A1Y1UY75\|A0A1Y1UY75_9FUNG | Ank_2, Ank_2, FYVE, zf-RING_2 |

| | | |
|---|---|---|
| 155 | tr\|A0A6A6QQF5\|A0A6A6QQF5_9PEZI | VHS, FYVE, UIM, UIM |
| 156 | tr\|A0A168QCU4\|A0A168QCU4_MUCCL | FYVE |
| 157 | tr\|A0A0D2PBI9\|A0A0D2PBI9_HYPSF | RhoGEF, FYVE |
| 158 | tr\|U4LJ46\|U4LJ46_PYROM | VHS, FYVE, UIM, UIM |
| 159 | tr\|A0A2T6ZAZ1\|A0A2T6ZAZ1_TUBBO | VHS, FYVE, UIM, UIM |
| 160 | tr\|A0A4U0WTC3\|A0A4U0WTC3_9PEZI | VHS, FYVE, UIM, UIM |
| 161 | tr\|A0A4S8LNE8\|A0A4S8LNE8_DENBC | RhoGEF, FYVE |
| 162 | tr\|A0A4V1IUA8\|A0A4V1IUA8_9FUNG | VHS, FYVE |
| 163 | tr\|L0P8I6\|L0P8I6_PNEJ8 | FYVE |
| 164 | tr\|R7Z380\|R7Z380_CONA1 | VHS, FYVE, UIM, UIM |
| 165 | tr\|E4ZXS6\|E4ZXS6_LEPMJ | VHS, FYVE, UIM, UIM |
| 166 | tr\|A0A168Q840\|A0A168Q840_MUCCL | VHS, FYVE, UIM, UIM |
| 167 | tr\|A0A0C2S3H8\|A0A0C2S3H8_AMAMU | RhoGEF, FYVE |
| 168 | tr\|A0A0C9N4H5\|A0A0C9N4H5_9FUNG | VHS, FYVE, UIM, UIM |
| 169 | tr\|R4XHH8\|R4XHH8_TAPDE | VHS, FYVE, UIM, UIM |
| 170 | tr\|A0A0B7NCZ8\|A0A0B7NCZ8_9FUNG | FYVE |
| 171 | tr\|A0A163F5K2\|A0A163F5K2_DIDRA | VHS, FYVE, UIM, UIM |

| | | |
|---|---|---|
| 172 | tr\|A0A6A6YA67\|A0A6A6YA67_9PEZI | VHS, FYVE, UIM, UIM |
| 173 | tr\|A0A5C3KRI4\|A0A5C3KRI4_9AGAR | RhoGEF, FYVE |
| 174 | tr\|A0A0C2YUU7\|A0A0C2YUU7_HEBCY | RhoGEF, FYVE |
| 175 | tr\|G4T9B0\|G4T9B0_SERID | RhoGEF, FYVE |
| 176 | tr\|A0A367JD28\|A0A367JD28_RHIAZ | VHS, FYVE, UIM, UIM |
| 177 | tr\|A0A367JX58\|A0A367JX58_RHIAZ | VHS, FYVE, UIM, UIM |
| 178 | tr\|A0A0M8MVG5\|A0A0M8MVG5_9BASI | FYVE, zf-RING 2 |
| 179 | tr\|S2JXE4\|S2JXE4_MUCC1 | VHS, FYVE, UIM, UIM |
| 180 | tr\|A0A286USL7\|A0A286USL7_9AGAM | RhoGEF, FYVE |
| 181 | tr\|A0A067NSG1\|A0A067NSG1_PLEOS | RhoGEF, FYVE |
| 182 | tr\|A0A316ZLR8\|A0A316ZLR8_9BASI | RhoGEF, FYVE |
| 183 | tr\|A0A4T0G0A2\|A0A4T0G0A2_9BASI | RhoGEF, FYVE |
| 184 | tr\|A0A075ARC6\|A0A075ARC6_ROZAC | FYVE |
| 185 | tr\|A0A4V1IZA8\|A0A4V1IZA8_ROZAC | FYVE |
| 186 | tr\|A0A3M7M1P8\|A0A3M7M1P8_9PLEO | VHS, FYVE, UIM, UIM |
| 187 | tr\|D5GD30\|D5GD30_TUBMM | VHS, FYVE, UIM, UIM |
| 188 | tr\|A0A1X7RQY2\|A0A1X7RQY2_ZYMTR | VHS, FYVE, UIM, UIM |

| | | |
|---|---|---|
| 189 | tr\|A0A1Y6LJF9\|A0A1Y6LJF9_ZYMTR | VHS, FYVE, UIM, UIM |
| 190 | tr\|A0A2H1GBB8\|A0A2H1GBB8_ZYMTR | VHS, FYVE, UIM, UIM |
| 191 | tr\|F9X9I8\|F9X9I8_ZYMTI | VHS, FYVE, UIM, UIM |
| 192 | tr\|A0A6A6A070\|A0A6A6A070_9PLEO | VHS, FYVE, UIM, UIM |
| 193 | tr\|B0DJQ4\|B0DJQ4_LACBS | RhoGEF, FYVE |
| 194 | tr\|A0A369JTG7\|A0A369JTG7_HYPMA | RhoGEF, FYVE |
| 195 | tr\|I4YHW0\|I4YHW0_WALMC | RhoGEF, FYVE |
| 196 | tr\|A0A507CTR3\|A0A507CTR3_9FUNG | VHS, FYVE, UIM, UIM |
| 197 | tr\|F4RZR0\|F4RZR0_MELLP | RhoGEF, FYVE |
| 198 | tr\|A0A4T0WVM5\|A0A4T0WVM5_9ASCO | VHS, FYVE, UIM, UIM |
| 199 | tr\|A0A150VI72\|A0A150VI72_9PEZI | VHS, FYVE, UIM, UIM |
| 200 | tr\|A0A3M6ZNS9\|A0A3M6ZNS9_HORWE | VHS, FYVE, UIM, UIM |
| 201 | tr\|A0A1Y1XRY0\|A0A1Y1XRY0_9FUNG | PH, FYVE |
| 202 | tr\|A0A074WRI8\|A0A074WRI8_9PEZI | VHS, FYVE, UIM, UIM |
| 203 | tr\|A0A6A7BU37\|A0A6A7BU37_9PEZI | VHS, FYVE |
| 204 | tr\|C\|K1WTE0_TRIAC | VHS, FYVE |
| 205 | tr\|J6EPC0\|J6EPC0_TRIAS | VHS, FYVE, UIM, UIM |

| | | |
|---|---|---|
| 206 | tr\|A0A3N4JEN9\|A0A3N4JEN9_9PEZI | VHS, FYVE, UIM, UIM |
| 207 | tr\|N1QCW3\|N1QCW3_PSEFD | VHS, FYVE, UIM, UIM |
| 208 | tr\|M2STQ7\|M2STQ7_COCH5 | VHS, FYVE, UIM, UIM |
| 209 | tr\|N4X1G1\|N4X1G1_COCH4 | VHS, FYVE, UIM, UIM |
| 210 | tr\|A0A6A5SBR1\|A0A6A5SBR1_9PLEO | VHS, FYVE, UIM, UIM |
| 211 | tr\|A0A1Y2LUT7\|A0A1Y2LUT7_EPING | VHS, FYVE |
| 212 | tr\|E2LZL4\|E2LZL4_MONPE | FYVE |
| 213 | tr\|A0A067S9R7\|A0A067S9R7_GALM3 | RhoGEF, FYVE |
| 214 | tr\|L0PFS6\|L0PFS6_PNEJ8 | FYVE |
| 215 | tr\|F4NZK1\|F4NZK1_BATDJ | VHS, FYVE, UIM, UIM |
| 216 | tr\|A0A3M7BVL5\|A0A3M7BVL5_HORWE | VHS, FYVE, UIM, UIM |
| 217 | tr\|A0A5J5F2H1\|A0A5J5F2H1_9PEZI | VHS, FYVE, UIM, UIM |
| 218 | tr\|A0A3M7GF14\|A0A3M7GF14_HORWE | VHS, FYVE, UIM, UIM |
| 219 | tr\|A0A3M7EI93\|A0A3M7EI93_HORWE | VHS, FYVE, UIM, UIM |
| 220 | tr\|A0A3M7A400\|A0A3M7A400_HORWE | VHS, FYVE, UIM, UIM |
| 221 | tr\|A0A162Y6D2\|A0A162Y6D2_MUCCL | FYVE |
| 222 | tr\|A0A4U0TZE6\|A0A4U0TZE6_9PEZI | VHS, FYVE, UIM, UIM |

| | | |
|---|---|---|
| 223 | tr\|A0A3M7DFE3\|A0A3M7DFE3_HORWE | VHS, FYVE, UIM, UIM |
| 224 | tr\|A0A3M7C5L4\|A0A3M7C5L4_HORWE | VHS, FYVE, UIM, UIM |
| 225 | tr\|A0A1Z5SW51\|A0A1Z5SW51_HORWE | VHS, FYVE, UIM, UIM |
| 226 | tr\|A0A3M7IAA8\|A0A3M7IAA8_HORWE | VHS, FYVE, UIM, UIM |
| 227 | tr\|A0A1Y1ZMB2\|A0A1Y1ZMB2_9PLEO | VHS, FYVE, UIM, UIM |
| 228 | tr\|A0A3M7EIE8\|A0A3M7EIE8_HORWE | VHS, FYVE, UIM, UIM |
| 229 | tr\|A0A3M7FRT0\|A0A3M7FRT0_HORWE | VHS, FYVE, UIM, UIM |
| 230 | tr\|A0A3M6WMK4\|A0A3M6WMK4_HORWE | VHS, FYVE, UIM, UIM |
| 231 | tr\|A0A0W4ZU85\|A0A0W4ZU85_PNEJ7 | FYVE |
| 232 | tr\|A0A409Y3I3\|A0A409Y3I3_9AGAR | RhoGEF, FYVE |
| 233 | tr\|A0A1X0QT00\|A0A1X0QT00_RHIZD | VHS, FYVE, UIM, UIM |
| 234 | tr\|A0A2G4SSF8\|A0A2G4SSF8_RHIZD | VHS, FYVE, UIM, UIM |
| 235 | tr\|A0A4V4JT84\|A0A4V4JT84_AURPU | VHS, FYVE, UIM, UIM |
| 236 | tr\|A0A4S9NID1\|A0A4S9NID1_AURPU | VHS, FYVE, UIM, UIM |
| 237 | tr\|A0A4V4JPE1\|A0A4V4JPE1_AURPU | VHS, FYVE, UIM, UIM |
| 238 | tr\|A0A4S8U3P8\|A0A4S8U3P8_AURPU | VHS, FYVE, UIM, UIM |
| 239 | tr\|A0A4T0CLC8\|A0A4T0CLC8_AURPU | VHS, FYVE, UIM, UIM |

| | | |
|---|---|---|
| 240 | tr\|A0A4S8XK24\|A0A4S8XK24_AURPU | VHS, FYVE, UIM, UIM |
| 241 | tr\|A0A4S9PXJ6\|A0A4S9PXJ6_AURPU | VHS, FYVE, UIM, UIM |
| 242 | tr\|A0A4S9S1G3\|A0A4S9S1G3_AURPU | VHS, FYVE, UIM, UIM |
| 243 | tr\|A0A4S9X121\|A0A4S9X121_AURPU | VHS, FYVE, UIM, UIM |
| 244 | tr\|A0A4S9MIC5\|A0A4S9MIC5_AURPU | VHS, FYVE, UIM, UIM |
| 245 | tr\|A0A4T0AAT4\|A0A4T0AAT4_AURPU | VHS, FYVE, UIM, UIM |
| 246 | tr\|A0A4V4LMX5\|A0A4V4LMX5_AURPU | VHS, FYVE, UIM, UIM |
| 247 | tr\|A0A4S9MD28\|A0A4S9MD28_AURPU | VHS, FYVE, UIM, UIM |
| 248 | tr\|A0A4S9SZX6\|A0A4S9SZX6_AURPU | VHS, UIM, UIM |
| 249 | tr\|A0A4S8TGC0\|A0A4S8TGC0_AURPU | VHS, FYVE, UIM, UIM |
| 250 | tr\|A0A4S9BBL0\|A0A4S9BBL0_AURPU | VHS, FYVE, UIM, UIM |

**Nonhuman mammals**

| ***No*** | ***Accession No*** | ***Modules*** |
|---|---|---|
| 1 | sp\|Q9H8W4\|PKHF2_HUMAN | PH, FYVE |
| 2 | tr\|G3RXJ3\|G3RXJ3_GORGO | PH, FYVE |
| 3 | tr\|A0A2R8ZRE3\|A0A2R8ZRE3_PANPA | PH, FYVE |
| 4 | tr\|A0A6D2WS13\|A0A6D2WS13_PANTR | PH, FYVE |
| 5 | tr\|H2QWG2\|H2QWG2_PANTR | PH, FYVE |
| 6 | tr\|A0A2K6B0W2\|A0A2K6B0W2_MACNE | PH, FYVE |
| 7 | tr\|A0A2K6JSQ3\|A0A2K6JSQ3_RHIBE | PH, FYVE |
| 8 | tr\|A0A2K5UMP8\|A0A2K5UMP8_MACFA | PH, FYVE |
| 9 | tr\|G7PC92\|G7PC92_MACFA | PH, FYVE |
| 10 | tr\|A0A2I3GPW4\|A0A2I3GPW4_NOMLE | PH, FYVE |
| 11 | tr\|A0A2K5XAN3\|A0A2K5XAN3_MANLE | PH, FYVE |
| 12 | tr\|A0A2K5KIW2\|A0A2K5KIW2_CERAT | PH, FYVE |
| 13 | tr\|A0A2J8UGL7\|A0A2J8UGL7_PONAB | PH, FYVE |
| 14 | tr\|G7MZT0\|G7MZT0_MACMU | PH, FYVE |
| 15 | tr\|A0A2K5PKC3\|A0A2K5PKC3_CEBIM | PH, FYVE |

| | | |
|---|---|---|
| 16 | tr\|A0A2K6NLA2\|A0A2K6NLA2_RHIRO | PH, FYVE |
| 17 | tr\|F7ASZ1\|F7ASZ1_MACMU | PH, FYVE |
| 18 | tr\|A0A6J3FGX3\|A0A6J3FGX3_SAPAP | PH, FYVE |
| 19 | tr\|A0A6D2X014\|A0A6D2X014_PONAB | PH, FYVE |
| 20 | tr\|A0A6I9ICZ8\|A0A6I9ICZ8_VICPA | PH, FYVE |
| 21 | tr\|S9WGC9\|S9WGC9_CAMFR | PH, FYVE |
| 22 | tr\|A0A5N4CHZ2\|A0A5N4CHZ2_CAMDR | PH, FYVE |
| 23 | tr\|A0A2K5HBE8\|A0A2K5HBE8_COLAP | PH, FYVE |
| 24 | tr\|A0A2K5C657\|A0A2K5C657_AOTNA | PH, FYVE |
| 25 | tr\|A0A096MLM1\|A0A096MLM1_PAPAN | PH, FYVE |
| 26 | tr\|F7FEC3\|F7FEC3_CALJA | PH, FYVE |
| 27 | tr\|A0A0D9SB87\|A0A0D9SB87_CHLSB | PH, FYVE |
| 28 | tr\|A0A2K6S793\|A0A2K6S793_SAIBB | PH, FYVE |
| 29 | tr\|A0A3Q7TWS2\|A0A3Q7TWS2_VULVU | PH, FYVE |
| 30 | tr\|A0A1S3AB18\|A0A1S3AB18_ERIEU | PH, FYVE |
| 31 | tr\|A0A5E4BFX5\|A0A5E4BFX5_MARMO | PH, FYVE |
| 32 | tr\|I3MAC4\|I3MAC4_ICTTR | PH, FYVE |
| 33 | tr\|A0A1U7T0A4\|A0A1U7T0A4_CARSF | PH, FYVE |
| 34 | tr\|A0A2K6ERG1\|A0A2K6ERG1_PROCO | PH, FYVE |
| 35 | tr\|A0A6I9JBJ2\|A0A6I9JBJ2_CHRAS | PH, FYVE |
| 36 | tr\|A0A3Q7P8U5\|A0A3Q7P8U5_CALUR | PH, FYVE |
| 37 | tr\|A0A3P4LK23\|A0A3P4LK23_GULGU | PH, FYVE |
| 38 | tr\|M3Z4K3\|M3Z4K3_MUSPF | PH, FYVE |
| 39 | tr\|A0A485MJJ6\|A0A485MJJ6_LYNPA | PH, FYVE |
| 40 | tr\|A0A667IBC1\|A0A667IBC1_LYNCA | PH, FYVE |
| 41 | tr\|A0A4X1U327\|A0A4X1U327_PIG | PH, FYVE |
| 42 | tr\|U6CRZ6\|U6CRZ6_NEOVI | PH, FYVE |
| 43 | tr\|A0A2Y9EYL3\|A0A2Y9EYL3_PHYMC | PH, FYVE |
| 44 | tr\|M3WVQ3\|M3WVQ3_FELCA | PH, FYVE |
| 45 | tr\|A0A7J8DUD9\|A0A7J8DUD9_MOLMO | PH, FYVE |
| 46 | tr\|A0A6P6HRR1\|A0A6P6HRR1_PUMCO | PH, FYVE |
| 47 | tr\|A0A287ASA9\|A0A287ASA9_PIG | PH, FYVE |
| 48 | tr\|A0A6J1ZRF1\|A0A6J1ZRF1_ACIJB | PH, FYVE |
| 49 | tr\|A0A6P4WXG5\|A0A6P4WXG5_PANPR | PH, FYVE |
| 50 | tr\|A0A5N3XBU7\|A0A5N3XBU7_MUNRE | PH, FYVE |
| 51 | tr\|A0A5N3WEU8\|A0A5N3WEU8_MUNMU | PH, FYVE |
| 52 | tr\|L8ILX8\|L8ILX8_9CETA | PH, FYVE |
| 53 | tr\|A4IFN8\|A4IFN8_BOVIN | PH, FYVE |
| 54 | tr\|A0A4W2EB56\|A0A4W2EB56_BOBOX | PH, FYVE |

| | | |
|---|---|---|
| 55 | tr\|A0A6P5CTL6\|A0A6P5CTL6_BOSIN | PH, FYVE |
| 56 | tr\|A0A6J0WJ70\|A0A6J0WJ70_ODOVR | PH, FYVE |
| 57 | tr\|A0A6P3GQJ4\|A0A6P3GQJ4_BISBI | PH, FYVE |
| 58 | tr\|A0A2U3WPC7\|A0A2U3WPC7_ODORO | PH, FYVE |
| 59 | tr\|A0A7J7VF05\|A0A7J7VF05_RHIFE | PH, FYVE |
| 60 | tr\|L5KAU6\|L5KAU6_PTEAL | PH, FYVE |
| 61 | tr\|A0A6P3QVM8\|A0A6P3QVM8_PTEVA | PH, FYVE |
| 62 | tr\|A0A2Y9PAZ8\|A0A2Y9PAZ8_DELLE | PH, FYVE |
| 63 | tr\|G1PK15\|G1PK15_MYOLU | PH, FYVE |
| 64 | tr\|A0A7J7TK50\|A0A7J7TK50_MYOMY | PH, FYVE |
| 65 | tr\|A0A2Y9GX76\|A0A2Y9GX76_NEOSC | PH, FYVE |
| 66 | tr\|L5MHS6\|L5MHS6_MYODS | PH, FYVE |
| 67 | tr\|A0A2U3XES2\|A0A2U3XES2_LEPWE | PH, FYVE |
| 68 | tr\|A0A7J7VN67\|A0A7J7VN67_PIPKU | PH, FYVE |
| 69 | tr\|A0A452QFV9\|A0A452QFV9_URSAM | PH, FYVE |
| 70 | tr\|A0A6G1APQ5\|A0A6G1APQ5_CROCR | PH, FYVE |
| 71 | tr\|A0A340WWB4\|A0A340WWB4_LIPVE | PH, FYVE |
| 72 | tr\|A0A3Q7WAC4\|A0A3Q7WAC4_URSAR | PH, FYVE |
| 73 | tr\|A0A250XXA1\|A0A250XXA1_CASCN | PH, FYVE |
| 74 | tr\|G9KH59\|G9KH59_MUSPF | PH, FYVE |
| 75 | tr\|A0A1U7QHL5\|A0A1U7QHL5_MESAU | PH, FYVE |
| 76 | tr\|A0A452CBQ1\|A0A452CBQ1_BALAS | PH, FYVE |
| 77 | tr\|A0A2Y9QET1\|A0A2Y9QET1_TRIMA | PH, FYVE |
| 78 | tr\|A0A6P5PKF8\|A0A6P5PKF8_MUSCR | PH, FYVE |
| 79 | tr\|A0A341CEL5\|A0A341CEL5_NEOAA | PH, FYVE |
| 80 | tr\|A0A484GRA3\|A0A484GRA3_SOUCH | PH, FYVE |
| 81 | tr\|A0A7J8C3Q9\|A0A7J8C3Q9_ROUAE | PH, FYVE |
| 82 | tr\|A0A384BXS8\|A0A384BXS8_URSMA | PH, FYVE |
| 83 | tr\|A0A2U4AI60\|A0A2U4AI60_TURTR | PH, FYVE |
| 84 | tr\|A0A2Y9J9Y4\|A0A2Y9J9Y4_ENHLU | PH, FYVE |
| 85 | tr\|L8YGB7\|L8YGB7_TUPCH | PH, FYVE |
| 86 | tr\|G3UCQ4\|G3UCQ4_LOXAF | PH, FYVE |
| 87 | tr\|D2HVB5\|D2HVB5_AILME | PH, FYVE |
| 88 | tr\|A0A6I9L8U8\|A0A6I9L8U8_PERMB | PH, FYVE |
| 89 | tr\|A0A1S3EVN9\|A0A1S3EVN9_DIPOR | PH, FYVE |
| 90 | tr\|A0A2K5C233\|A0A2K5C233_AOTNA | PH, FYVE |
| 91 | tr\|H0Y0B3\|H0Y0B3_OTOGA | PH, FYVE |
| 92 | tr\|A0A6J2NCI4\|A0A6J2NCI4_9CHIR | PH, FYVE |
| 93 | sp\|Q91WB4\|PKHF2_MOUSE | PH, FYVE |

| | | |
|---|---|---|
| 94 | tr|A0A7J7EHJ9|A0A7J7EHJ9_DICBM | PH, FYVE |
| 95 | tr|K9IHW6|K9IHW6_DESRO | PH, FYVE |
| 96 | tr|A0A452DYL3|A0A452DYL3_CAPHI | PH, FYVE |
| 97 | tr|A0A3Q2HWW6|A0A3Q2HWW6_HORSE | PH, FYVE |
| 98 | tr|W5PZ70|W5PZ70_SHEEP | PH, FYVE |
| 99 | tr|A0A6P7ECY0|A0A6P7ECY0_SHEEP | PH, FYVE |
| 100 | tr|B1WBV4|B1WBV4_RAT | PH, FYVE |
| 101 | tr|A0A6J2F8G3|A0A6J2F8G3_ZALCA | PH, FYVE |
| 102 | tr|A0A7N4NQ24|A0A7N4NQ24_SARHA | PH, FYVE |
| 103 | tr|A0A6P5IQU3|A0A6P5IQU3_PHACI | PH, FYVE |
| 104 | tr|A0A286XYJ1|A0A286XYJ1_CAVPO | PH, FYVE |
| 105 | tr|A0A5E4BEY9|A0A5E4BEY9_MARMO | PH, FYVE |
| 106 | tr|A0A091DW22|A0A091DW22_FUKDA | PH, FYVE |
| 107 | tr|A0A0P6K1S8|A0A0P6K1S8_HETGA | PH, FYVE |
| 108 | tr|F7A285|F7A285_ORNAN | PH, FYVE |
| 109 | tr|F6Q9Z2|F6Q9Z2_MONDO | SQS_PSY, PH, FYVE |
| 110 | tr|A0A6P3FWN8|A0A6P3FWN8_OCTDE | PH, FYVE |
| 111 | tr|A0A6A1QJM0|A0A6A1QJM0_BALPH | PH, FYVE |
| 112 | tr|A0A673V5I4|A0A673V5I4_SURSU | PH, FYVE |
| 113 | tr|A0A6J0YZI7|A0A6J0YZI7_ODOVR | PH, FYVE |
| 114 | tr|F7F262|F7F262_MONDO | PH, FYVE |
| 115 | tr|A0A5F8H7A3|A0A5F8H7A3_MONDO | PH, FYVE |
| 116 | tr|A0A5F8GYD7|A0A5F8GYD7_MONDO | PH, FYVE |
| 117 | tr|A8Y5N8|A8Y5N8_MOUSE | PH, FYVE |
| 118 | tr|A0A5N3XKB2|A0A5N3XKB2_MUNRE | PH, FYVE |
| 119 | tr|A0A1A6FXD9|A0A1A6FXD9_NEOLE | PH, FYVE |
| 120 | tr|A0A6B0RRU8|A0A6B0RRU8_9CETA | PH, FYVE |
| 121 | tr|A0A5N3UY12|A0A5N3UY12_MUNMU | PH, FYVE |
| 122 | tr|A0A0D9SB08|A0A0D9SB08_CHLSB | PH, FYVE |
| 123 | tr|A0A2J8KSM2|A0A2J8KSM2_PANTR | PH, FYVE |
| 124 | sp|Q96S99|PKHF1_HUMAN | PH, FYVE |
| 125 | tr|A0A2K5JGW8|A0A2K5JGW8_COLAP | PH, FYVE |
| 126 | tr|A0A2K6CYZ9|A0A2K6CYZ9_MACNE | PH, FYVE |
| 127 | tr|A0A2K6JWD4|A0A2K6JWD4_RHIBE | PH, FYVE |
| 128 | tr|A0A2K5UDM0|A0A2K5UDM0_MACFA | PH, FYVE |
| 129 | tr|G3QH50|G3QH50_GORGO | PH, FYVE |
| 130 | tr|A0A2I3G625|A0A2I3G625_NOMLE | PH, FYVE |
| 131 | tr|A0A2K5XLS1|A0A2K5XLS1_MANLE | PH, FYVE |
| 132 | tr|A0A0A0MVG7|A0A0A0MVG7_PAPAN | PH, FYVE |

| | | |
|---|---|---|
| 133 | tr\|A0A2K5KX81\|A0A2K5KX81_CERAT | PH, FYVE |
| 134 | tr\|G7NLE7\|G7NLE7_MACMU | PH, FYVE |
| 135 | tr\|A0A2K6NPM4\|A0A2K6NPM4_RHIRO | PH, FYVE |
| 136 | tr\|F6WEV7\|F6WEV7_MACMU | PH, FYVE |
| 137 | tr\|H2QFY5\|H2QFY5_PANTR | PH, FYVE |
| 138 | tr\|A0A2J8SAC5\|A0A2J8SAC5_PONAB | PH, FYVE |
| 139 | tr\|A0A6D2Y8X4\|A0A6D2Y8X4_PONAB | PH, FYVE |
| 140 | tr\|G7PX49\|G7PX49_MACFA | PH, FYVE |
| 141 | tr\|A0A2R9AA53\|A0A2R9AA53_PANPA | PH, FYVE |
| 142 | tr\|B4DWN9\|B4DWN9_HUMAN | PH, FYVE |
| 143 | tr\|L8IB21\|L8IB21_9CETA | PH, FYVE |
| 144 | tr\|A2VE19\|A2VE19_BOVIN | PH, FYVE |
| 145 | tr\|A0A4W2H1W7\|A0A4W2H1W7_BOBOX | PH, FYVE |
| 146 | tr\|A0A6P5DDG5\|A0A6P5DDG5_BOSIN | PH, FYVE |
| 147 | tr\|A0A091D210\|A0A091D210_FUKDA | PH, FYVE |
| 148 | tr\|A0A6B0R7Q5\|A0A6B0R7Q5_9CETA | PH, FYVE |
| 149 | tr\|A0A2K5PK00\|A0A2K5PK00_CEBIM | PH, FYVE |
| 150 | tr\|A0A1U7R682\|A0A1U7R682_MESAU | PH, FYVE |
| 151 | tr\|A0A2K6SFK7\|A0A2K6SFK7_SAIBB | PH, FYVE |
| 152 | tr\|A0A2K5E2Y5\|A0A2K5E2Y5_AOTNA | PH, FYVE |
| 153 | tr\|U3D2H9\|U3D2H9_CALJA | PH, FYVE |
| 154 | tr\|A0A6J3FHK1\|A0A6J3FHK1_SAPAP | PH, FYVE |
| 155 | tr\|H0XH62\|H0XH62_OTOGA | PH, FYVE |
| 156 | tr\|A0A6J1Y5U6\|A0A6J1Y5U6_ACIJB | PH, FYVE |
| 157 | tr\|A0A7N4PT60\|A0A7N4PT60_SARHA | PH, FYVE |
| 158 | tr\|A0A6P3YLF4\|A0A6P3YLF4_SHEEP | PH, FYVE |
| 159 | tr\|G5BL44\|G5BL44_HETGA | PH, FYVE |
| 160 | tr\|A0A5K1U7A2\|A0A5K1U7A2_CALJA | PH, FYVE |
| 161 | tr\|A0A7J8CA60\|A0A7J8CA60_MOLMO | PH, FYVE |
| 162 | sp\|Q68FU1\|PKHF1_RAT | PH, FYVE |
| 163 | tr\|A0A5J5MWL9\|A0A5J5MWL9_MUNRE | PH, FYVE |
| 164 | tr\|A0A5N3VFQ3\|A0A5N3VFQ3_MUNMU | PH, FYVE |
| 165 | tr\|A0A452DRQ5\|A0A452DRQ5_CAPHI | PH, FYVE |
| 166 | tr\|A0A6J0XTU7\|A0A6J0XTU7_ODOVR | PH, FYVE |
| 167 | tr\|A0A1S3AR32\|A0A1S3AR32_ERIEU | PH, FYVE |
| 168 | tr\|A0A7N4NQK3\|A0A7N4NQK3_SARHA | PH, FYVE |
| 169 | tr\|A0A6P5K5Z3\|A0A6P5K5Z3_PHACI | PH, FYVE |
| 170 | tr\|A0A286XEI1\|A0A286XEI1_CAVPO | PH, FYVE |
| 171 | tr\|A0A6P5K5R9\|A0A6P5K5R9_PHACI | PH, FYVE |

| | | |
|---|---|---|
| 172 | tr\|A0A6J0XTS7\|A0A6J0XTS7_ODOVR | PH, FYVE |
| 173 | tr\|A0A485NDJ2\|A0A485NDJ2_LYNPA | PH, FYVE |
| 174 | tr\|A0A667J1S0\|A0A667J1S0_LYNCA | PH, FYVE |
| 175 | tr\|A0A4X2L5D8\|A0A4X2L5D8_VOMUR | PH, FYVE |
| 176 | tr\|A0A6P5K760\|A0A6P5K760_PHACI | PH, FYVE |
| 177 | tr\|M3WT21\|M3WT21_FELCA | PH, FYVE |
| 178 | tr\|A0A6P6H4E9\|A0A6P6H4E9_PUMCO | PH, FYVE |
| 179 | tr\|A0A6P4TXR5\|A0A6P4TXR5_PANPR | PH, FYVE |
| 180 | tr\|A0A2U3X5H7\|A0A2U3X5H7_ODORO | PH, FYVE |
| 181 | tr\|A0A6G1B9W0\|A0A6G1B9W0_CROCR | PH, FYVE |
| 182 | tr\|A0A6J0E1B0\|A0A6J0E1B0_PERMB | PH, FYVE |
| 183 | tr\|F7EZS4\|F7EZS4_MONDO | PH, FYVE |
| 184 | tr\|A0A6J0DUG8\|A0A6J0DUG8_PERMB | PH, FYVE |
| 185 | tr\|A0A2Y9HYM5\|A0A2Y9HYM5_NEOSC | PH, FYVE |
| 186 | tr\|A0A2U3Y6L0\|A0A2U3Y6L0_LEPWE | PH, FYVE |
| 187 | tr\|A0A5E4BQ75\|A0A5E4BQ75_MARMO | PH, FYVE |
| 188 | tr\|I3N3T1\|I3N3T1_ICTTR | PH, FYVE |
| 189 | tr\|F7AL15\|F7AL15_ORNAN | PH, FYVE |
| 190 | tr\|A0A6J0E1A5\|A0A6J0E1A5_PERMB | PH, FYVE |
| 191 | tr\|A0A3Q7MSJ7\|A0A3Q7MSJ7_CALUR | PH, FYVE |
| 192 | tr\|A0A6J2F2G5\|A0A6J2F2G5_ZALCA | PH, FYVE |
| 193 | tr\|A0A6P6EYH5\|A0A6P6EYH5_OCTDE | PH, FYVE |
| 194 | sp\|Q3TB82\|PKHF1_MOUSE | PH, FYVE |
| 195 | tr\|A0A061HTX9\|A0A061HTX9_CRIGR | PH, FYVE |
| 196 | tr\|A0A671FB93\|A0A671FB93_RHIFE | PH, FYVE |
| 197 | tr\|A0A341AK17\|A0A341AK17_NEOAA | PH, FYVE |
| 198 | tr\|A0A340YG10\|A0A340YG10_LIPVE | PH, FYVE |
| 199 | tr\|A0A5N4DR82\|A0A5N4DR82_CAMDR | PH, FYVE |
| 200 | tr\|A0A4U1EUS3\|A0A4U1EUS3_MONMO | PH, FYVE |
| 201 | tr\|A0A2Y9P694\|A0A2Y9P694_DELLE | PH, FYVE |
| 202 | tr\|A0A2U3V2U0\|A0A2U3V2U0_TURTR | PH, FYVE |
| 203 | tr\|A0A3Q7TL14\|A0A3Q7TL14_VULVU | PH, FYVE |
| 204 | tr\|G1ML40\|G1ML40_AILME | PH, FYVE |
| 205 | tr\|A0A6P7R571\|A0A6P7R571_MUSCR | PH, FYVE |
| 206 | tr\|A0A3P4S7D1\|A0A3P4S7D1_GULGU | PH, FYVE |
| 207 | tr\|A0A6P3V9N9\|A0A6P3V9N9_OCTDE | PH, FYVE |
| 208 | tr\|M3Z5G8\|M3Z5G8_MUSPF | PH, FYVE |
| 209 | tr\|D2H395\|D2H395_AILME | PH, FYVE |
| 210 | tr\|A0A383YQU7\|A0A383YQU7_BALAS | PH, FYVE |

| | | |
|---|---|---|
| 211 | tr\|A0A2Y9F4I2\|A0A2Y9F4I2_PHYMC | PH, FYVE |
| 212 | tr\|A0A2Y9LBN9\|A0A2Y9LBN9_ENHLU | PH, FYVE |
| 213 | tr\|A0A3Q7UUY9\|A0A3Q7UUY9_URSAR | PH, FYVE |
| 214 | tr\|A0A2F0BD53\|A0A2F0BD53_ESCRO | PH, FYVE |
| 215 | tr\|G3UCB5\|G3UCB5_LOXAF | PH, FYVE |
| 216 | tr\|A0A2Y9QH78\|A0A2Y9QH78_TRIMA | PH, FYVE |
| 217 | tr\|A0A250YE73\|A0A250YE73_CASCN | PH, FYVE |
| 218 | tr\|A0A6I9K0J7\|A0A6I9K0J7_CHRAS | PH, FYVE |
| 219 | tr\|A0A452QT72\|A0A452QT72_URSAM | PH, FYVE |
| 220 | tr\|A0A4X1TW95\|A0A4X1TW95_PIG | PH, FYVE |
| 221 | tr\|F1RNZ0\|F1RNZ0_PIG | PH, FYVE |
| 222 | tr\|A0A484GVY7\|A0A484GVY7_SOUCH | PH, FYVE |
| 223 | tr\|A0A7J8CKE8\|A0A7J8CKE8_ROUAE | PH, FYVE |
| 224 | tr\|A0A5G2RAS5\|A0A5G2RAS5_PIG | PH, FYVE |
| 225 | tr\|A0A1A6G1C6\|A0A1A6G1C6_NEOLE | PH, FYVE |
| 226 | tr\|A0A6J2L2L8\|A0A6J2L2L8_9CHIR | PH, FYVE |
| 227 | tr\|A0A3Q2LSN2\|A0A3Q2LSN2_HORSE | PH, FYVE |
| 228 | tr\|E2R1B7\|E2R1B7_CANLF | PH, FYVE |
| 229 | tr\|K9IX34\|K9IX34_DESRO | PH, FYVE |
| 230 | tr\|A0A7J7SDK0\|A0A7J7SDK0_MYOMY | PH, FYVE |
| 231 | tr\|A0A6P3IET2\|A0A6P3IET2_BISBI | PH, FYVE |
| 232 | tr\|G3HI22\|G3HI22_CRIGR | FYVE |
| 233 | tr\|G5C4B3\|G5C4B3_HETGA | FYVE |
| 234 | tr\|W5P1M9\|W5P1M9_SHEEP | PH |
| 235 | tr\|S9WTG5\|S9WTG5_CAMFR | PH, FYVE |
| 236 | tr\|A0A6J0AU76\|A0A6J0AU76_VICPA | PH |
| 237 | tr\|L5KVX2\|L5KVX2_PTEAL | PH |
| 238 | tr\|A0A2J8KSN2\|A0A2J8KSN2_PANTR | PH |
| 239 | tr\|A0A2J8SAE9\|A0A2J8SAE9_PONAB | PH |
| 240 | tr\|K7ELB8\|K7ELB8_HUMAN | PH |
| 241 | tr\|L8Y3Z0\|L8Y3Z0_TUPCH | PH, FYVE |
| 242 | tr\|A0A2J8KSM7\|A0A2J8KSM7_PANTR | NO |
| 243 | tr\|A0A2J8SAE1\|A0A2J8SAE1_PONAB | NO |
| 244 | tr\|K7EIX0\|K7EIX0_HUMAN | NO |
| 245 | tr\|A0A2J8KVM1\|A0A2J8KVM1_PANTR | PH, FYVE |
| 246 | tr\|A0A2J8XN15\|A0A2J8XN15_PONAB | PH, FYVE |
| 247 | tr\|F8VQX5\|F8VQX5_HUMAN | PH, FYVE |
| 248 | tr\|A0A4X2MDN9\|A0A4X2MDN9_VOMUR | RhoGEF, PH, FYVE, PH |
| 249 | tr\|A0A4X2M634\|A0A4X2M634_VOMUR | RhoGEF, PH, FYVE, PH |

| | | |
|---|---|---|
| 250 | tr\|F6W6U6\|F6W6U6_MONDO | RhoGEF, PH, FYVE, PH |

**Humans**

| *No* | *Accession No* | *Modules* |
|---|---|---|
| 1 | sp\|Q9H8W4\|PKHF2_HUMAN | PH, FYVE |
| 2 | sp\|Q96S99\|PKHF1_HUMAN | PH, FYVE |
| 3 | tr\|B4DWN9\|B4DWN9_HUMAN | PH, FYVE |
| 4 | tr\|K7ELB8\|K7ELB8_HUMAN | PH |
| 5 | tr\|K7EIX0\|K7EIX0_HUMAN | NO |
| 6 | tr\|F8VQX5\|F8VQX5_HUMAN | PH, FYVE |
| 7 | tr\|A4FVC4\|A4FVC4_HUMAN | RhoGEF, PH, FYVE, PH |
| 8 | sp\|Q6ZV73\|FGD6_HUMAN | R RhoGEF, PH, FYVE, PH |
| 9 | tr\|A0A2X0SFJ2\|A0A2X0SFJ2_HUMAN | RhoGEF, PH, FYVE, PH |
| 10 | tr\|Q49A55\|Q49A55_HUMAN | RhoGEF, PH, FYVE |
| 11 | tr\|F8W1R0\|F8W1R0_HUMAN | RhoGEF, PH, FYVE, PH |
| 12 | sp\|Q96M96\|FGD4_HUMAN | RhoGEF, PH, FYVE, PH |
| 13 | tr\|A0A2X0U2J9\|A0A2X0U2J9_HUMAN | RhoGEF, PH, FYVE, PH |
| 14 | tr\|B7Z493\|B7Z493_HUMAN | RhoGEF, PH, FYVE, PH |

| | | |
|---|---|---|
| 15 | tr\|B7Z8F9\|B7Z8F9_HUMAN | RhoGEF, PH, FYVE, PH |
| 16 | tr\|E9PJX4\|E9PJX4_HUMAN | RhoGEF, PH, FYVE, PH |
| 17 | tr\|F8VWL3\|F8VWL3_HUMAN | RhoGEF, PH, FYVE, PH |
| 18 | sp\|Q7Z6J4\|FGD2_HUMAN | RhoGEF, PH, FYVE, PH |
| 19 | tr\|A8K304\|A8K304_HUMAN | RhoGEF, PH, FYVE, PH |
| 20 | tr\|A0A384NQ05\|A0A384NQ05_HUMAN | RhoGEF, PH, FYVE, PH |
| 21 | tr\|A0A2X0SYZ7\|A0A2X0SYZ7_HUMAN | RhoGEF, PH, FYVE, PH |
| 22 | sp\|P98174\|FGD1_HUMAN | RhoGEF, PH, FYVE, PH |
| 23 | tr\|A0A024R9Y5\|A0A024R9Y5_HUMAN | RhoGEF, PH, FYVE, PH |
| 24 | tr\|A0A2X0SFE1\|A0A2X0SFE1_HUMAN | RhoGEF, PH, FYVE, PH |
| 25 | tr\|G3V2D8\|G3V2D8_HUMAN | FYVE |
| 26 | tr\|A0A7I2YQV0\|A0A7I2YQV0_HUMAN | FYVE |
| 27 | sp\|Q68DK2\|ZFY26_HUMAN | FYVE |
| 28 | tr\|A0A7I2YQU3\|A0A7I2YQU3_HUMAN | FYVE |
| 29 | tr\|A0A7I2V403\|A0A7I2V403_HUMAN | FYVE |
| 30 | tr\|B7ZM68\|B7ZM68_HUMAN | RhoGEF, PH, FYVE |
| 31 | tr\|A3KMQ0\|A3KMQ0_HUMAN | RhoGEF, PH, FYVE, PH |
| 32 | sp\|Q6ZNL6\|FGD5_HUMAN | RhoGEF, PH, FYVE, PH |
| 33 | tr\|A0A2X0SFF2\|A0A2X0SFF2_HUMAN | RhoGEF, PH, FYVE, PH |
| 34 | sp\|Q8WXA3\|RUFY2_HUMAN | RUN, FYVE |

| | | |
|---|---|---|
| 35 | tr\|H0YD93\|H0YD93_HUMAN | RUN, FYVE |
| 36 | tr\|B3KSL1\|B3KSL1_HUMAN | FYVE |
| 37 | tr\|A8K7B1\|A8K7B1_HUMAN | RUN, FYVE |
| 38 | sp\|Q96T51\|RUFY1_HUMAN | RUN, FYVE |
| 39 | tr\|Q59GI4\|Q59GI4_HUMAN | FYVE, DUF3480 |
| 40 | sp\|O95405\|ZFYV9_HUMAN | FYVE, DUF3480 |
| 41 | sp\|Q7Z3T8\|ZFY16_HUMAN | FYVE, DUF3480 |
| 42 | tr\|A0A024RAL3\|A0A024RAL3_HUMAN | FYVE, DUF3480 |
| 43 | tr\|B4DVN4\|B4DVN4_HUMAN | FYVE, PH |
| 44 | sp\|Q15075\|EEA1_HUMAN | FYVE |
| 45 | tr\|Q49AA1\|Q49AA1_HUMAN | FYVE |
| 46 | tr\|F8VY01\|F8VY01_HUMAN | RhoGEF, FYVE |
| 47 | sp\|Q9HCC9\|LST2_HUMAN | FYVE |
| 48 | sp\|Q9Y4F1\|FARP1_HUMAN | FERM_M, FERM_C, FA, RhoGEF, PH, PH |
| 49 | tr\|A0A2X0TVY0\|A0A2X0TVY0_HUMAN | FERM_M, FERM_C, FA, RhoGEF, PH, PH |
| 50 | tr\|B4DS99\|B4DS99_HUMAN | RhoGEF, PH |
| 51 | tr\|G5E953\|G5E953_HUMAN | Myotub-related, FYVE |

| | | |
|---|---|---|
| 52 | sp\|Q13615\|MTMR3_HUMAN | Myotub-related, FYVE |
| 53 | tr\|A8K0L6\|A8K0L6_HUMAN | Myotub-related, FYVE |
| 54 | tr\|A7E2W2\|A7E2W2_HUMAN | Myotub-related, FYVE |
| 55 | sp\|Q5JSP0\|FGD3_HUMAN | RhoGEF, PH, FYVE |
| 56 | tr\|A0A024R252\|A0A024R252_HUMAN | RhoGEF, PH, FYVE |
| 57 | tr\|A0A2X0U4Q9\|A0A2X0U4Q9_HUMAN | RhoGEF, PH, FYVE |
| 58 | tr\|Q9H8N9\|Q9H8N9_HUMAN | WD40 |
| 59 | sp\|Q8IWB7\|WDFY1_HUMAN | WD40 |
| 60 | tr\|A0A024R488\|A0A024R488_HUMAN | WD40 |
| 61 | tr\|A0A7I2YQD1\|A0A7I2YQD1_HUMAN | VHS, FYVE, Hrs_helical |
| 62 | tr\|I3L1P5\|I3L1P5_HUMAN | VHS, FYVE, Hrs_helical |
| 63 | tr\|B4E1E2\|B4E1E2_HUMAN | VHS, FYVE, Hrs_helical |
| 64 | tr\|A0A7I2V3Z1\|A0A7I2V3Z1_HUMAN | VHS, FYVE, Hrs_helical |
| 65 | tr\|Q9NXY1\|Q9NXY1_HUMAN | RhoGEF, PH, FYVE |
| 66 | tr\|A0A0S2Z4Q4\|A0A0S2Z4Q4_HUMAN | VHS, FYVE |
| 67 | tr\|A0A7I2V6E0\|A0A7I2V6E0_HUMAN | VHS, FYVE, Hrs_helical |
| 68 | tr\|A8K9G1\|A8K9G1_HUMAN | RhoGEF, PH, FYVE |

| | | |
|---|---|---|
| 69 | tr\|A0A7I2V2K2\|A0A7I2V2K2_HUMAN | VHS, FYVE, Hrs_helical |
| 70 | tr\|A0A7I2V5A2\|A0A7I2V5A2_HUMAN | VHS, FYVE, Hrs_helical |
| 71 | tr\|A0A7I2V3I8\|A0A7I2V3I8_HUMAN | VHS, FYVE, Hrs_helical |
| 72 | tr\|A0A7I2V3F9\|A0A7I2V3F9_HUMAN | VHS, FYVE, Hrs_helical |
| 73 | tr\|A0A7I2V2D8\|A0A7I2V2D8_HUMAN | VHS, FYVE, Hrs_helical |
| 74 | tr\|A0A7I2V2W5\|A0A7I2V2W5_HUMAN | VHS, FYVE, Hrs_helical |
| 75 | tr\|A0A7I2V2R9\|A0A7I2V2R9_HUMAN | VHS, FYVE, Hrs_helical |
| 76 | sp\|O14964\|HGS_HUMAN | VHS, FYVE, Hrs_helical |
| 77 | tr\|A0A7I2V3J3\|A0A7I2V3J3_HUMAN | VHS, FYVE, Hrs_helical |
| 78 | tr\|A0A0S2Z4R4\|A0A0S2Z4R4_HUMAN | VHS, FYVE, Hrs_helical |
| 79 | tr\|A0A7I2V637\|A0A7I2V637_HUMAN | VHS, VHS, FYVE, Hrs_helical |
| 80 | tr\|A0A7I2V5A3\|A0A7I2V5A3_HUMAN | VHS, FYVE, Hrs_helical |
| 81 | tr\|B4DFP5\|B4DFP5_HUMAN | VHS, FYVE |
| 82 | sp\|O94887\|FARP2_HUMAN | FERM_M, FERM_C, FA, RhoGEF, PH, PH |
| 83 | tr\|A0A2X0SSL3\|A0A2X0SSL3_HUMAN | FERM_M, FERM_C, FA, RhoGEF, PH, PH |
| 84 | tr\|J3QR65\|J3QR65_HUMAN | Myotub-related, FYVE |
| 85 | sp\|Q9NYA4\|MTMR4_HUMAN | Myotub-related, FYVE |
| 86 | tr\|Q5H9P4\|Q5H9P4_HUMAN | Ank_2, Ank2, Ank2, Ank2, FYVE |

| | | |
|---|---|---|
| 87 | sp\|Q9P2R3\|ANFY1_HUMAN | Ank_2, Ank2, Ank2, Ank2, FYVE |
| 88 | tr\|A0A7I2V307\|A0A7I2V307_HUMAN | VHS, FYVE |
| 89 | tr\|A0A024R1I2\|A0A024R1I2_HUMAN | Myotub-related, FYVE |
| 90 | sp\|Q9HBF4\|ZFYV1_HUMAN | FYVE, FYVE |
| 91 | tr\|G3V5N8\|G3V5N8_HUMAN | FYVE, FYVE |
| 92 | tr\|C9JME2\|C9JME2_HUMAN | FERM_M, FERM_C, FA, RhoGEF, PH, PH |
| 93 | tr\|C9JL08\|C9JL08_HUMAN | FYVE |
| 94 | sp\|Q9BQ24\|ZFY21_HUMAN | FYVE, ZFYVE21_C |
| 95 | tr\|E9PDH4\|E9PDH4_HUMAN | FYVE, DEP, Cpn60_TCP1 |
| 96 | sp\|Q9Y2I7\|FYV1_HUMAN | FYVE, DEP, Cpn60_TCP1 |
| 97 | tr\|V9HWG4\|V9HWG4_HUMAN | FYVE, DEP, Cpn60_TCP1 |
| 98 | tr\|A0A7I2V5X2\|A0A7I2V5X2_HUMAN | FYVE |
| 99 | tr\|A7E1Z6\|A7E1Z6_HUMAN | FYVE |
| 100 | tr\|Q96LK4\|Q96LK4_HUMAN | WD40, FYVE |
| 101 | sp\|Q9BQS8\|FYCO1_HUMAN | RUN, FYVE |
| 102 | sp\|Q96P53\|WDFY2_HUMAN | WD40, FYVE |
| 103 | tr\|A7E293\|A7E293_HUMAN | Beach, FYVE |

| | | |
|---|---|---|
| 104 | sp\|Q8IZQ1\|WDFY3_HUMAN | Beach, FYVE |
| 105 | tr\|A0A024RDC2\|A0A024RDC2_HUMAN | Beach, FYVE |
| 106 | tr\|B4DXH4\|B4DXH4_HUMAN | PH, FYVE |
| 107 | tr\|A0A7I2V5N3\|A0A7I2V5N3_HUMAN | FYVE |
| 108 | tr\|A0A7I2V5W6\|A0A7I2V5W6_HUMAN | FYVE |
| 109 | tr\|Q96P52\|Q96P52_HUMAN | FYVE |
| 110 | tr\|B3KPJ0\|B3KPJ0_HUMAN | RhoGEF |
| 111 | tr\|H0Y696\|H0Y696_HUMAN | RhoGEF |
| 112 | tr\|A8K4R4\|A8K4R4_HUMAN | RCC1, RhoGEF |
| 113 | tr\|Q6IQ41\|Q6IQ41_HUMAN | RCC1, RhoGEF |
| 114 | sp\|Q96Q42\|ALS2_HUMAN | RCC1, RhoGEF, vps9 |
| 115 | tr\|A0A2X0SFA0\|A0A2X0SFA0_HUMAN | RCC1, RhoGEF, vps9 |
| 116 | sp\|Q9H1K0\|RBNS5_HUMAN | FYVE, NPF |
| 117 | tr\|A8K5H3\|A8K5H3_HUMAN | FYVE, NPF |
| 118 | tr\|H3BRF9\|H3BRF9_HUMAN | FYVE |
| 119 | sp\|Q8TCU6\|PREX1_HUMAN | RhoGEF, DEP, DEP |

| | | |
|---|---|---|
| 120 | tr\|A0A2X0SFH1\|A0A2X0SFH1_HUMAN | RhoGEF, DEP, DEP |
| 121 | tr\|M0QXT1\|M0QXT1_HUMAN | RhoGEF |
| 122 | tr\|B3KPZ0\|B3KPZ0_HUMAN | Ank_2 |
| 123 | tr\|H3BRM1\|H3BRM1_HUMAN | FYVE |
| 124 | tr\|E9PJM7\|E9PJM7_HUMAN | PH |
| 125 | sp\|Q96K21\|ANCHR_HUMAN | FYVE |
| 126 | tr\|B4E245\|B4E245_HUMAN | PH |
| 127 | tr\|E7EMB1\|E7EMB1_HUMAN | PH |
| 128 | tr\|B3KUB9\|B3KUB9_HUMAN | PH |
| 129 | sp\|Q9UH65\|SWP70_HUMAN | PH |
| 130 | sp\|Q70Z35\|PREX2_HUMAN | RhoGEF, PH, DEP, DEP |
| 131 | tr\|A0A2X0SZ31\|A0A2X0SZ31_HUMAN | RhoGEF, PH, DEP, DEP |
| 132 | tr\|H3BN64\|H3BN64_HUMAN | FYVE |
| 133 | tr\|H7C3C4\|H7C3C4_HUMAN | HCO3_cotransp |
| 134 | tr\|C9JRP1\|C9JRP1_HUMAN | HCO3_cotransp |
| 135 | tr\|A0A0A0MST8\|A0A0A0MST8_HUMAN | HCO3_cotransp |
| 136 | tr\|B5M450\|B5M450_HUMAN | HCO3_cotransp |
| 137 | tr\|E9PFN4\|E9PFN4_HUMAN | HCO3_cotransp |
| 138 | sp\|Q9Y6M7\|S4A7_HUMAN | HCO3_cotransp |
| 139 | tr\|Q5JVY0\|Q5JVY0_HUMAN | zf-RING_UBOX |

| | | |
|---|---|---|
| 140 | tr\|E7EW28\|E7EW28_HUMAN | HCO3_cotransp |
| 141 | tr\|C9J240\|C9J240_HUMAN | HCO3_cotransp |
| 142 | sp\|Q6U841\|S4A10_HUMAN | HCO3_cotransp |
| 143 | sp\|Q13049\|TRI32_HUMAN | NHL |
| 144 | tr\|A0A024R843\|A0A024R843_HUMAN | NHL |
| 145 | sp\|P52735\|VAV2_HUMAN | RhoGEF, PH,C1, SH2 |
| 146 | tr\|B4DFS9\|B4DFS9_HUMAN | RhoGEF, PH |
| 147 | tr\|H0Y724\|H0Y724_HUMAN | PH |
| 148 | tr\|B4E2V8\|B4E2V8_HUMAN | Sec7, PH |
| 149 | sp\|Q9UIA0\|CYH4_HUMAN | Sec7, PH |
| 150 | tr\|B2RCD2\|B2RCD2_HUMAN | Sec7, PH |
| 151 | tr\|A7E2A5\|A7E2A5_HUMAN | PH, PH, PH, RhoGAP, RA, PH |
| 152 | sp\|Q8WZ64\|ARAP2_HUMAN | PH, PH, PH, RhoGAP, RA, PH |
| 153 | tr\|A0A2X0SFS3\|A0A2X0SFS3_HUMAN | PH, PH, PH, RhoGAP, RA, PH |
| 154 | tr\|B4DZ04\|B4DZ04_HUMAN | RhoGEF, PH |
| 155 | tr\|B4E1Y1\|B4E1Y1_HUMAN | RhoGEF, PH |
| 156 | tr\|Q5SQI7\|Q5SQI7_HUMAN | RhoGEF, PH |
| 157 | tr\|A0A2X0SFV4\|A0A2X0SFV4_HUMAN | RhoGEF, PH |
| 158 | sp\|Q7Z628\|ARHG8_HUMAN | RhoGEF, PH |
| 159 | tr\|Q5SQI5\|Q5SQI5_HUMAN | RhoGEF, PH |

| | | |
|---|---|---|
| 160 | tr|B4DS29|B4DS29_HUMAN | HCO3_cotransp |
| 161 | sp|Q2Y0W8|S4A8_HUMAN | HCO3_cotransp |
| 162 | tr|Q9NUL6|Q9NUL6_HUMAN | Sec7, PH |
| 163 | tr|A0A0D9SFG6|A0A0D9SFG6_HUMAN | Sec7, PH |
| 164 | sp|Q6ZNE9|RUFY4_HUMAN | RUN |
| 165 | tr|B2RBP7|B2RBP7_HUMAN | PH |
| 166 | sp|Q8IVQ6|ZDH21_HUMAN | DHHC |
| 167 | tr|Q15795|Q15795_HUMAN | Sec7, PH |
| 168 | tr|C9JKV5|C9JKV5_HUMAN | FYVE_2 |
| 169 | sp|Q9P2F6|RHG20_HUMAN | RA, RhoGAP |
| 170 | tr|A0A2X0U2C5|A0A2X0U2C5_HUMAN | RA, RhoGAP |
| 171 | tr|E9PNM3|E9PNM3_HUMAN | I-set |
