# Supplementary material for: The PH Domain and C-Terminal polyD Motif of Phafin2 Exhibit a Unique Concurrence in Animals
Source: Membranes (Basel). 2022 Jul 7;12(7):696. doi: 10.3390/membranes12070696 (PMC9324892; doi:10.3390/membranes12070696)
Supplement: Supplementary file 1 [file membranes-12-00696-s001.zip › Supplementary File S4.pdf]

Supplementary File S4: Multiple sequence alignment of Phafin2 and Phafin2-like proteins from bacteria, archaea, fungi, plants, nonhuman mammals, and humans.

Bacteria

```
sp|Q9H8W4|PKHF2_HUMAN              ----ARSDSYSQSLKSPLNDMSDDDDDDDDSSD---------------------------      249
tr|A0A1Y6CWR2|A0A1Y6CWR2_9GAMM     LDP-AKAQLAQGLRNPVLGQGGHPQIQAKPS-----------------------------      1181
tr|A0A4Q5XUS2|A0A4Q5XUS2_9DELT     ------------------------------------------------------------      383
tr|A0A0A8E4G1|A0A0A8E4G1_9GAMM     -----VKN--------------------------IYKGFC--------------------      114
tr|A0A2E9XWQ4|A0A2E9XWQ4_9RICK     VSF-APKPSTMQPRSARVE-TSSFDQDLKRA---LEMSLE--------------------      271
tr|A0A0G2ZPZ8|A0A0G2ZPZ8_9DELT     ------------------------------------------------------------      347
tr|A0A5C6VDZ7|A0A5C6VDZ7_9BURK     ------------------------------------------------------------      269
tr|A0A2E9LIA3|A0A2E9LIA3_9CHLR     ------------------------------------------------------------      90
tr|A0A2E4CAT1|A0A2E4CAT1_9ACTN     TEP-LL----------VMGDGGWADIRDWRVLCCLNRGWK--------------------      267
tr|A0A4Q6X9R2|A0A4Q6X9R2_9BURK     ------------------------------------------------------------      334
tr|A0A158KI31|A0A158KI31_9BURK     ------------------------------------------------------------      332
tr|A0A2W4SQ66|A0A2W4SQ66_9GAMM     ------------------------------------------------------------      329
tr|A0A5J6QPA5|A0A5J6QPA5_9PSED     ------------------------------------------------------------      309
tr|A0A561P8H2|A0A561P8H2_XANCT     ------------------------------------------------------------      290
tr|A0A2E8LZD9|A0A2E8LZD9_9BACT     IRI-LS----------LVG----LDPQDMCRLACLSRSWK--------------------      111
tr|A0A4R7ALI0|A0A4R7ALI0_9GAMM     ------------------------------------------------------------      321
tr|A0A3L7RST6|A0A3L7RST6_9BACT     ---------------------------QITLALCLSARTA--------------------      340
tr|A0A2E0WHK0|A0A2E0WHK0_9CHLR     LMF-LP-----------------LPISELEVFLYVNKKWN--------------------      112
tr|A0A1Z9KZ67|A0A1Z9KZ67_9SYNE     LRP-APADAQIGTLSSPLA-GGHHPRHG--------------------------------      131
tr|A0A2E9LIE3|A0A2E9LIE3_9CHLR     LRF--------------------IPVKCWTSCFLVSRQFS--------------------      301
tr|A0A3S3EH81|A0A3S3EH81_9PSED     ------------------------------------------------------------      313
tr|A0A0F4XPH7|A0A0F4XPH7_9PSED     ------------------------------------------------------------      411
tr|A0A657PDX6|A0A657PDX6_9PSED     ------------------------------------------------------------      411
tr|G8Q597|G8Q597_PSEF3             ------------------------------------------------------------      411
tr|A0A2E0E560|A0A2E0E560_9RHOB     FAN-LP-----------------IVIVDFYNLRLVCKKWC--------------------      149
tr|A0A0C2EFB1|A0A0C2EFB1_9PSED     ------------------------------------------------------------      412
tr|A0A2E6VXV8|A0A2E6VXV8_9PROT     -----K-----------------------------NNFIF--------------------      128
tr|A0A2D7RET3|A0A2D7RET3_9GAMM     LCL-LP-----------------LTIMEKHVFLYVNKSWN--------------------      107
tr|A0A4R7L8D8|A0A4R7L8D8_9GAMM     ------------------------------------------------------------      310
tr|A0A4R3YS80|A0A4R3YS80_9GAMM     ------------------------------------------------------------      288
tr|A0A4Q3E3P0|A0A4Q3E3P0_9RICK     IRQ-LF----------ENL----VPYDEMTA---HN---L--------------------      127
tr|A0A0Q9PCH9|A0A0Q9PCH9_9GAMM     ------------------------------------------------------------      262
tr|A0A2D5QHX0|A0A2D5QHX0_9GAMM     SMG-YPSDLVNEQIERGAGYKGSTPDEKVKA----VVEWF--------------------      223
tr|A0A239KKI8|A0A239KKI8_9PSED     ------------------------------------------------------------      1020
tr|A0A2E8CGN4|A0A2E8CGN4_9DELT     IRD-LK-----QRRSEIEGELQKN------------------------------------      590
tr|A0A6I1NH92|A0A6I1NH92_9PSED     ------------------------------------------------------------      1019
tr|A0A1E3GCQ5|A0A1E3GCQ5_9GAMM     ------------------------------------------------------------      312
```

```
tr|A0A2A8BFD9|A0A2A8BFD9_9BACI      --M-DPADFVAMQLSGV-------------------------------------------      88
tr|A0A484ZT69|A0A484ZT69_9GAMM      ------------------------------------------------------------      146
tr|A0A2M7G853|A0A2M7G853_9BACT      NRIPIARDAVRT------G----RRRIEWREDQPASLFWVEAQDQGDPSHPSQIRDRLFD      371
tr|A0A2C5DIR4|A0A2C5DIR4_9BACI      --M-DPADFVAMQLSGV-------------------------------------------      88
```

Archaea

```
sp|Q9H8W4|PKHF2_HUMAN               --DDDSSD----------------------------------------------------      249
tr|A0A482RXE8|A0A482RXE8_9ARCH      ------------------------------------------------------------      468
tr|A0A482S4E7|A0A482S4E7_9ARCH      -VKDVSQELW---PYFPIALLLC---------T-----------------------QD-L      149
tr|A0A482RTS6|A0A482RTS6_9ARCH      ----LGLDPA---PWALRRLATL---------P-----------------------Y---      193
tr|A0A482UR88|A0A482UR88_9ARCH      ------------------------------------------------------------      110
tr|A0A482R0G1|A0A482R0G1_9EURY      ------------------------------------------------------------      93
tr|A0A482RYJ3|A0A482RYJ3_9ARCH      ------------------------------------------------------------      158
tr|A0A482SL25|A0A482SL25_9ARCH      ---GGFGAFW---ACI-----------------------------------IPNIAPQ--      280
tr|A0A482SVI4|A0A482SVI4_9ARCH      -----------SLGSAATTGSRSPVAGSIAA--------TTG------------------      127
tr|A0A482ST03|A0A482ST03_9ARCH      ------------------------------------------------------------      153
tr|A0A482R4B7|A0A482R4B7_9EURY      --GAGGSEAW---PGTLGNVTDV---------P-----------------------LA-P      290
tr|A0A482SVF7|A0A482SVF7_9ARCH      SEITSNHDEW---KQSIFNLVRE---------V------------------VSSVDPDVK      202
tr|A0A482SZF2|A0A482SZF2_9ARCH      ------------------------------------------------------------      465
tr|A0A482RGE2|A0A482RGE2_9ARCH      ------------------------------------------------------------      596
tr|A0A482RTU5|A0A482RTU5_9ARCH      SGGSLPLLQW---LLEVKRCSLM---------D---------------------RASG--      361
tr|A0A482SG35|A0A482SG35_9ARCH      V----------------DSVVTT-------------------------------------      227
tr|A0A482RW94|A0A482RW94_9ARCH      ------------------------------------------------------------      169
tr|A0A482RV05|A0A482RV05_9ARCH      ------------------------------------------------------------      129
tr|A0A482RVR9|A0A482RVR9_9ARCH      P----------------FFVIEA-------------------------------------      182
tr|A0A482SAN9|A0A482SAN9_9ARCH      -----------SLPVVLQEAVDSAVNNSIDS--------SEN------------------      207
tr|A0A482RAR6|A0A482RAR6_9ARCH      -AEVEARIAW---GQAVNVVSGC---------EGIT--PLHAA-VSYFNAYLQRVASG-D      316
tr|A0A482UQL2|A0A482UQL2_9ARCH      PTLTSPLPPW---IEGLVYVRHT-------------------------------------      251
tr|A0A482V091|A0A482V091_9ARCH      ------------------------------------------------------------      171
tr|A0A482S307|A0A482S307_9ARCH      ------------------------------------------------------------      158
tr|A0A482SRA1|A0A482SRA1_9ARCH      -----------NLPSSIQILVDCG------A--------DVN------------------      276
tr|A0A482RXE5|A0A482RXE5_9ARCH      ------------------------------------------------------------      80
tr|A0A482SVG8|A0A482SVG8_9ARCH      -NRAHSGI-------------EY---------MQSELDESEVK-LNTANEEA--------      228
tr|A0A482UVH9|A0A482UVH9_9ARCH      ------------------------------------------------------------      82
tr|A0A482RQW7|A0A482RQW7_9ARCH      --GECPSRSL---IETIRYCSIY---------C---------------------I-----      138
tr|A0A3A5UT62|A0A3A5UT62_9EURY      ------------------------------------------------------------      279
tr|A0A482UPW1|A0A482UPW1_9ARCH      -------------------------------------PGESQK-IDRITDAFS-------      15
tr|A0A7C5RN86|A0A7C5RN86_9ARCH      -DCWANRKPW---SEHVYYTYKF---------VRACL-GEELA-VYAMRHHVG-------      47
tr|A0A7C4ZF49|A0A7C4ZF49_9ARCH      -DCWANQRPL---PEHVYYTYKF---------VKDCL-GEELA-VHAMRHHVE-------      64
tr|A0A524F3J3|A0A524F3J3_9ARCH      PERYRKIPPF---PF-G----SK---------D---------------------------      63
tr|A0A7J2X8Y9|A0A7J2X8Y9_9EURY      -------RGYVPLPIKISIKNSKN-----IVAFGGEINTSICILSRN-------------      324
tr|A0A482RV58|A0A482RV58_9ARCH      ------------------------------------------------------------      130
tr|A0A2E8V4G5|A0A2E8V4G5_9EURY      SDGWGLLQS----PSSIT--Q--------GAFFGRSISVSGDINNDGFNDLVV-------      563
```

```
tr|A0A7K4DTG4|A0A7K4DTG4_9ARCH      NMKWTKLQSE---TMHIPQLTRT---------S---------------------------      67
tr|H6Q7G6|H6Q7G6_PYROT              ------------------------------------------------------------      82
tr|A0A7J2XB36|A0A7J2XB36_9EURY      ------------------------------------------------------------      53
tr|A0A662LIL0|A0A662LIL0_9EURY      ------------------------------------------------------------      53
tr|A0A7L4PAM3|A0A7L4PAM3_9CREN      ------------------------------------------------------------      83
tr|A4WL18|A4WL18_PYRAR              ------------------------------------------------------------      83
tr|A0A2D6X683|A0A2D6X683_9ARCH      ------------------------------------------------------------      86
tr|A0A151E9R7|A0A151E9R7_9EURY      -------RGYVPEPIEVNYNKK-------ILSVGAGENITGAVSHDK-------------      398
tr|A0A497EKV5|A0A497EKV5_9ARCH      ------------------------------------------------------------      0
tr|A0A482SQW3|A0A482SQW3_9ARCH      ------------------------------------------------------------      95
tr|A0A2E5RC13|A0A2E5RC13_9EURY      ------------------------------------------------------------      128
tr|A0A2E9DYS3|A0A2E9DYS3_9EURY      NDTWGDLQS----PSSIT--Q--------GAFFGRSISTSGDINNDGFNDLVV-------      563
tr|A0A524DJL4|A0A524DJL4_9ARCH      PERYRKIPPF---PF-G----SH---------D---------------------------      63
tr|A0A497HLC9|A0A497HLC9_9EURY      LKKYISIRES---PI---------------------------------------------      217
tr|A0A7C4S6U6|A0A7C4S6U6_9ARCH      ------------------------------------------------------------      89
tr|A0A7J4LRK9|A0A7J4LRK9_9ARCH      ------------------------------------------------------------      0
tr|A0A7K4IJU2|A0A7K4IJU2_9ARCH      ------------------------------------------------------------      109
tr|A0A2D9NJM7|A0A2D9NJM7_9EURY      SDGWGLLQS----PSSIT--Q--------GAFFGRSISVSGDINNDGFNDLVV-------      563
tr|A0A5A7S0K7|A0A5A7S0K7_9EURY      ------------------------------------------------------------      0
tr|A0A202E9J8|A0A202E9J8_9EURY      DDLWALARDHENGPGDDSICRHPE-PGLTKHSFGQLTTASAA------------------      348
tr|A0A7J4TTH8|A0A7J4TTH8_9ARCH      ------------------------------------------------------------      0
tr|A0A2E6BWJ1|A0A2E6BWJ1_9EURY      --CDEKKPTF---LYHG----SN---------S---------------------------      27
tr|A0A7J4I1H7|A0A7J4I1H7_9ARCH      -NTWIPTN---------YP--KT---------LEAHEPGSGY----------P-------      44
tr|A0A7C6DZH7|A0A7C6DZH7_9ARCH      -DCWANRKPW---SEHVYYTYKF---------VRACL-GEELA-VYAMRHHVG-------      58
tr|A0A662JAF1|A0A662JAF1_9CREN      ------------------------------------------------------------      53
tr|A0A151ENE1|A0A151ENE1_9EURY      -------RGYVPEPIAVNYDKK-------ILSVGAGENITGAVSHDK-------------      398
tr|A0A2U3CK81|A0A2U3CK81_HEIAB      -LDWHGNPSS---DEETIN--DI---------INKLEKEETVV-VSDFN-----------      298
tr|A0A2U9ILS6|A0A2U9ILS6_9CREN      ------------------------------------------------------------      0
tr|A0A1V4YZZ9|A0A1V4YZZ9_9EURY      ------------------------------------------------------------      46
tr|A0A0X8UZZ7|A0A0X8UZZ7_9EURY      -----------SLSRNSPICPTCGIPLNMSPTF--ALEGSVT------------------      272
tr|A0A1H6TIC6|A0A1H6TIC6_9EURY      ------------------------------------------------------------      0
tr|A0A7C0UA16|A0A7C0UA16_9ARCH      ------------------------------------------------------------      49
```

Fungi

```
sp|Q9H8W4|PKHF2_HUMAN               -----------------------QSLKS---PLND--------------MSDDDDDDDSS      248
tr|A0A2Z6RA14|A0A2Z6RA14_9GLOM      ---DL-------A--------------------------------PGIHVWDLV-----S      745
tr|A0A397VBS0|A0A397VBS0_9GLOM      ---DL-------A--------------------------------PGIHIWNSST----G      459
tr|A0A015IGQ3|A0A015IGQ3_RHIIW      ---DL-------A--------------------------------PGIHVWDLV-----G      785
```

```
tr|A0A397TNA4|A0A397TNA4_9GLOM      ---DL-------A--------------------------------PGIHVWDLV-----G    780
tr|A0A015KA47|A0A015KA47_RHIIW      ---DL-------A--------------------------------PGIHVWDLV-----G    785
tr|A0A015IQ23|A0A015IQ23_RHIIW      ---DL-------A--------------------------------PGIHVWDLV-----G    785
tr|A0A2I1G6N8|A0A2I1G6N8_9GLOM      ---DL-------A--------------------------------PGIHVWDLV-----G    785
tr|A0A2I1F0G8|A0A2I1F0G8_9GLOM      ---DL-------A--------------------------------PGIHVWDLV-----G    785
tr|U9SXZ4|U9SXZ4_RHIID              ---DL-------A--------------------------------PGIHVWDLV-----G    785
tr|A0A372RFQ0|A0A372RFQ0_9GLOM      ---DL-------A--------------------------------PGIHVWDLV-----G    786
tr|A0A4P9YZL8|A0A4P9YZL8_9FUNG      ----D-------M--------------------------------LRSRTMNFVQ----S    447
tr|A0A4V1IX88|A0A4V1IX88_9FUNG      ----Q-------A--------------------------------MRLRSLTFVA----A    446
tr|A0A4P9ZP44|A0A4P9ZP44_9FUNG      ---DN-------R--------------------------------MGMPLCS--------    456
tr|A0A0L0HGC7|A0A0L0HGC7_SPIPD      ------------E--------------------------------S--------------    673
tr|A0A177WRZ8|A0A177WRZ8_BATDL      ---------HPLS------------------------------------ID------D--    515
tr|F4P650|F4P650_BATDJ              ---------HPLS------------------------------------ID------D--    557
tr|A0A1Y1X2T5|A0A1Y1X2T5_9FUNG      ---------KNKR-----------------------------ILE----LANSSKDAE-N    374
tr|A0A507FBG2|A0A507FBG2_9FUNG      ----------LQALPSLPVEPIDDYCDD---SLTTKHYTGNAGVEMRRRASS--------    474
tr|A0A1Y1X1V7|A0A1Y1X1V7_9FUNG      ---------KPKR-----------------------------VLE----LSSS-QFSE-E    608
tr|A0A4V1IY93|A0A4V1IY93_9FUNG      ------------------------------------------------------------    524
tr|A0A1Y1X677|A0A1Y1X677_9FUNG      ---------KNKR-----------------------------IKE----LAIPDKNAE-N    701
tr|A0A137P479|A0A137P479_CONC2      ---TL-------A--------------------------------LGLRRYSLIN----Y    867
tr|A0A507EFA4|A0A507EFA4_9FUNG      ------------L--------------------------------LARRSFDIP------    652
tr|A0A507CXB0|A0A507CXB0_9FUNG      ----------SP-----------------------TLGSG-----------GSSGDEDES    526
tr|A0A1S8W822|A0A1S8W822_9FUNG      ---------HSLS------------------------------------IN------E--    537
tr|A0A397IVX3|A0A397IVX3_9GLOM      ---DE-------T--------------------------------PRIRVLNQTT----S    725
tr|A0A139AK54|A0A139AK54_GONPJ      ----------VP-----------------------TNPSG---------------VESIR    242
tr|A0A4P9WLC0|A0A4P9WLC0_9FUNG      ------------------------------------------------------------    705
tr|A0A1Y1WA63|A0A1Y1WA63_9FUNG      ---------HSLDPSALL--ST---LAA---LAGSASPP---RLEPRLEIT------EPP    486
tr|A0A0E9ND18|A0A0E9ND18_SAICN      ----------TLE--------DDLTN-G------------------------FQDIEIII    972
tr|A0A2G5BFQ4|A0A2G5BFQ4_COERN      ------------------------------------------------------------    386
tr|A0A0W4ZGN5|A0A0W4ZGN5_PNEJ7      ------------Q--------------------------------SNLNVSESIT----S    609
tr|A0A167MKJ0|A0A167MKJ0_CALVF      -------------------------N----------------------------------    641
tr|A0A165DP15|A0A165DP15_9BASI      -------------------------R----------------------------------    642
tr|M5FY58|M5FY58_DACPD              -------------------------H----------------------------------    640
tr|A0A1Y2IJD5|A0A1Y2IJD5_PYCCO      ------------------------------------------------------------    355
tr|A0A1Y2IIH2|A0A1Y2IIH2_PYCCO      ------------------------------------------------------------    526
tr|A0A1Y2CRY6|A0A1Y2CRY6_9FUNG      -------------------------------------P-I--------------------    154
tr|M9MDT8|M9MDT8_PSEA3              ---DSYTAPESAEMSSVPSTSTDDSVAN---ALGA----------YRLT-SDVDTDAE-G    1050
tr|A0A0L0SM81|A0A0L0SM81_ALLM3      -----------------------VDAHG------------------------HDTA--TQ    610
tr|A0A1M2VA80|A0A1M2VA80_TRAPU      ------------------------------------------------------------    656
tr|A0A2H3IXF5|A0A2H3IXF5_WOLCO      ------------------------------------------------------------    558
tr|A0A4Q9MH65|A0A4Q9MH65_9APHY      ------------------------------------------------------------    627
tr|A0A5E3XNR7|A0A5E3XNR7_9AGAM      -------------------E-----R----------------------------------    639
tr|A0A5C3FP20|A0A5C3FP20_PSEA2      ---DAYTAPESAEMSSVPSTSTDDSVAN---ALGA----------YRLT-SDVDTDAE-G    1003
tr|A0A081CEI2|A0A081CEI2_PSEA2      ---DAYTAPESAEMSSVPSTSTDDSVAN---ALGA----------YRLT-SDVDTDAE-G    1050
tr|K5WQA6|K5WQA6_PHACS              ------------------------------------------------------------    538
tr|A0A0H2S726|A0A0H2S726_9AGAM      ------------------------------------------------------------    617
```

```
tr|A0A165Z002|A0A165Z002_9AGAM      -------------------E-----R----------------------------------      642
tr|A0A481SFP1|A0A481SFP1_9BASI      ---EVVAPPASAEISSVPSTSTDESMAN---ALRA----------NRIA-SDVDTDAE-G      1114
tr|A0A1Y1YYR8|A0A1Y1YYR8_9FUNG      ----------------QVE-----------------TK----------------------      413
tr|A0A4Q9NS61|A0A4Q9NS61_9APHY      ------------------------------------------------------------      627
tr|A0A4Q9Q5Q4|A0A4Q9Q5Q4_9APHY      ------------------------------------------------------------      627
tr|A0A5C3NHQ1|A0A5C3NHQ1_9AGAM      -------------------A-----V----------------------------------      646
tr|A0A481SI77|A0A481SI77_9BASI      ---EAVAPPASAEISSVPSTSTDESMAN---ALGA----------IRIA-SDVDTDAE-G      1054
tr|S8FM81|S8FM81_FOMPI              ------------------------------------------------------------      534
tr|A0A2G8SBX2|A0A2G8SBX2_9APHY      ------------------------------------------------------------      645
tr|A0A137NR99|A0A137NR99_CONC2      ----------SDSNSSLPTL------------LQ-TTP----------------------      399
tr|A0A1Y1Z786|A0A1Y1Z786_9FUNG      ----------N-NERKYSDSDSDPETAG------------------------SSY----P      288
tr|A0A5C3ERJ4|A0A5C3ERJ4_9BASI      ---EAIAAPDSAEMSSVPSTSTDESVAQ---TLRA----------YRIA-SDFDTDAE-G      1049
tr|A0A165BJR9|A0A165BJR9_9APHY      ------------------------------------------------------------      640
tr|A0A164UM97|A0A164UM97_9AGAM      ------------------------------------------------------------      620
tr|A0A0D7AXV4|A0A0D7AXV4_9AGAR      -------------------S-----E----------------------------------      616
tr|A0A6A6HW15|A0A6A6HW15_9PLEO      TLYQ-----------------------------GSMEPRN-----ARVE---DSFD----      266
tr|A0A165L4G7|A0A165L4G7_9APHY      ------------------------------------------------------------      631
tr|A0A2N8UMQ8|A0A2N8UMQ8_9BASI      TAEAVVAAPDSAEMSSVPSSSTDDSVAQ---ALGA----------YQIGVSDGDTDAD-G      1082
tr|V5GIZ1|V5GIZ1_KALBG              AASDATTGADSVEMSSAPSTSTDDSVSQ---ALGG----------LRIGASDTDTDAE-G      719
tr|A0A2T2PC27|A0A2T2PC27_CORCC      TLYQ-----------------------------GSMEPRN-----ARVE---DSFD----      266
tr|A0A0L0HIX4|A0A0L0HIX4_SPIPD      ----------Q------P------------------------------------------      437
tr|A0A5C2U016|A0A5C2U016_9APHY      ------------------------------------------------------------      629
tr|A0A5C2SLU6|A0A5C2SLU6_9APHY      ------------------------------------------------------------      629
tr|A0A2R6PJ56|A0A2R6PJ56_9APHY      ------------------------------------------------------------      904
tr|A0A074RVC0|A0A074RVC0_9AGAM      -----------------------------------------------------TFM-SSR      966
tr|X8IWG9|X8IWG9_9AGAM              -----------------------------------------------------TFM-SSR      966
tr|A0A067Q7F2|A0A067Q7F2_9AGAM      -------------------I-----I----------------------------------      685
tr|E6ZVA4|E6ZVA4_SPORE              TAEAVAAAPDSAEMSSVPSSSTDDSVAQ---ALGA----------YQIGVSDGDTDAD-G      1080
tr|A0A3N4I9X6|A0A3N4I9X6_ASCIM      ------------------------H-----HHKHKPQPRN-----ARVE---DDDD----      255
tr|A0A0C3NH87|A0A0C3NH87_PHLGI      ------------------------------------------------------------      646
tr|A0A166A0Y0|A0A166A0Y0_9AGAM      ------------------------------------------------------------      655
tr|A0A0F7RZL7|A0A0F7RZL7_9BASI      AAEAMATAPDSAEMSSVPSSSTDESVAQ---TLGA----------YQIGALDGDTDAE-A      1077
tr|A0A6A5VUJ0|A0A6A5VUJ0_9PLEO      TLYQ-----------------------------GSMEPRN-----PRVE---DSFD----      266
tr|A0A6G6FQD5|A0A6G6FQD5_9APHY      ------------------------------------------------------------      651
tr|A0A0B7F915|A0A0B7F915_THACB      -----------------------------------------------------TIM-ALR      950
tr|Q8NJX4|Q8NJX4_USTMD              --AEASAAPDSADMSSVPTTGTDGSVAQ---AFGA----------YRIGASDADTDAE-G      1062
tr|R9PB35|R9PB35_PSEHS              TVVATSVAPDSADMSSVPSTGTDESVAQ---TLDA----------YRMGTSGTETDVE-S      1051
tr|A0A077R837|A0A077R837_9BASI      ---ETMIAPDSAEMFSVPSTSTDDSVTH---TLEA----------YRMI-SDIDTDAE-G      1055
tr|A0A0D1E146|A0A0D1E146_USTMA      --AEASAAPDSADMSSVPTTGTDGSVAQ---AFGA----------YRIGASDADTDAE-G      1062
tr|A0A1K0FWK6|A0A1K0FWK6_9BASI      -EDANAVGPDNAEMSLVPSTSTDDSVAQ---TLGA----------YRFA-SDLDTDVEAG      1085
tr|A0A4U7KNT8|A0A4U7KNT8_9BASI      TAEAMATAPDSAEMSSVPSSSTDESVTQ---ALSA----------YHIGASDGDTDAE-V      1081
tr|A0A4T0IBV4|A0A4T0IBV4_WALIC      ----------TT-------------------------Q-A-----------RCQDS-EV-      854
tr|R9AV38|R9AV38_WALI9              ----------TT-------------------------Q-A-----------RCQDS-EV-      854
tr|A0A4T0J9U8|A0A4T0J9U8_WALIC      ----------TT-------------------------Q-A-----------RCQDS-GV-      854
tr|I2FM96|I2FM96_USTH4              -EDANAVGPDIAEMPLVPSTSTDDSVAQ---TLGA----------YRFA-SDLDTDVEAG      1084
```

```
tr|A0A177BYI1|A0A177BYI1_9PLEO      TLYQ-----------------------------GSMEPRN-----PRVE---DSFD----      266
tr|A0A5C3M448|A0A5C3M448_9AGAR      -------------------E--R--S----------------------------------      692
tr|A0A0W0G1I7|A0A0W0G1I7_9AGAR      ---------------------IDMTH----------------------------------      881
tr|V2X232|V2X232_MONRO              ---------------------IDMTH----------------------------------      881
tr|A0A6A5UD68|A0A6A5UD68_9PLEO      TLYQ-----------------------------GSMEPPS-----ARVE---DSFD----      265
tr|A0A507ES66|A0A507ES66_9FUNG      ------------E--------------------------------S--------------      688
tr|A0A0K6FN13|A0A0K6FN13_9AGAM      -----------------------------------------------------TLM-SSR      960
tr|A0A5M9KV80|A0A5M9KV80_9PLEO      TLYQ---------G--------------------SMEPRN-----ARVE---DSFD----      266
tr|A0A2W1CTG6|A0A2W1CTG6_9PLEO      TLYQ---------G--------------------SMEPRN-----ARVE---DSFD----      266
tr|A0A6S6V7N3|A0A6S6V7N3_9PLEO      TFYQ---------G--------------------SMEPRN-----ARVE---DSFD----      266
tr|B2W9Q9|B2W9Q9_PYRTR              TLYQ---------G--------------------SMEPRN-----ARVE---DSFD----      266
tr|A0A316ZS75|A0A316ZS75_9PLEO      TLYQ---------G--------------------SMEPRN-----ARVE---DSFD----      266
tr|A0A6A6RWR3|A0A6A6RWR3_9PLEO      TMYQ-----------------------------GSMEPRS-----ARVE---DSFD----      265
tr|A0A1Y2AFM9|A0A1Y2AFM9_9FUNG      ----------YGN---IDFNDSDF--YGNDDYIDSFDTSN------RYGATSSKEDDDIQ      278
tr|A0A1Y2GJ54|A0A1Y2GJ54_9FUNG      ----------INS---LGGPAELVPATSKPAQSSSSKPSM------VQPPNPLSEDEDLK      397
tr|A0A316VEC8|A0A316VEC8_9BASI      --------------ASTPATN----VEQ---CIHA--------------SRALDSDASLP      935
tr|A0A6A5QBT9|A0A6A5QBT9_AMPQU      TLYQ---------G--------------------AMEPRS-----ARID---DSFD----      267
tr|A0A6A6ZW62|A0A6A6ZW62_9PLEO      TLYQ---------G--------------------SMEPRS-----ARVE---DSFD----      266
tr|A0A317XKP1|A0A317XKP1_9BASI      VSSDAEPELTSAEMTSAP--STDESIST---ALSN----------YRLD-SDGGDDADAD      1025
tr|A0A5C3F116|A0A5C3F116_9BASI      --------PAAAAAEPLPTSSTRQTVTG---VNMAAAPSNDGSVSGRSGSSSGLF--DSD      1044
tr|A0A177WUX8|A0A177WUX8_BATDL      ----------S------P------------------------------------------      518
tr|F4P7P0|F4P7P0_BATDJ              ----------S------P------------------------------------------      518
tr|A0A6A6V864|A0A6A6V864_9PLEO      TLYQ-----------------------------SSMEPRS-----ARVE--ADSFD----      176
tr|N1QHA6|N1QHA6_SPHMS              TLWQ---------TSGMSGTESALG-----GGTDRMQPRD-----ARVT--DDSFD----      286
tr|A0A2V1DU97|A0A2V1DU97_9PLEO      TLYQ-----------------------------GSMEPRS-----ARVE---DSFD----      265
tr|A0A6A6TFR8|A0A6A6TFR8_9PLEO      TLYQ-----------------------------RSMEPRS-----ARVE--TDSFD----      266
tr|A0A550CL33|A0A550CL33_9AGAR      -------------------SNDITGT----------------------------------      707
tr|A0A284S9X6|A0A284S9X6_ARMOS      -------------------H--VPPE----------------------------------      671
tr|A0A0C3Q7C3|A0A0C3Q7C3_9AGAM      ----------SPTSVSFSQI-----R----------------------------------      681
tr|A0A367KQZ9|A0A367KQZ9_RHIST      -------------------------------KIPESLPAQ-----PQPVADDNEFD----      259
tr|A0A074VKM0|A0A074VKM0_9PEZI      P--------------------------------GRMQARD-----ARVA--EDSFD----      259
tr|R0ITW9|R0ITW9_SETT2              TLYQ---------G--------------------SMEPRN-----ARVE---DSFD----      266
tr|A0A1Y2EN19|A0A1Y2EN19_9BASI      ----------N----------------------------------------------PIQ      985
tr|A0A507BWX7|A0A507BWX7_9FUNG      ----------SS-------------------------PGG-----------DDDDEAEQS      544
tr|A0A6A6JKH9|A0A6A6JKH9_9PLEO      TLYQ-----------------------------RSMEPRS-----ARVE--FDNFD----      264
tr|A0A3N4M7C5|A0A3N4M7C5_9PEZI      SRN---------------------K-----PPAPSMQPRN-----ARVE---DDDD----      270
tr|A0A6A5JXY0|A0A6A5JXY0_9PLEO      TLYQ---------G--------------------SMEPRN-----ARVE---DSFD----      266
tr|A0A6G1IPA9|A0A6G1IPA9_9PLEO      TLYQ-----------------------------GSMEPRS-----ARVE---DSFD----      266
tr|A0A0D7A3M6|A0A0D7A3M6_9AGAR      -------------------L-SPTMS----------------------------------      659
tr|A0A0C3B4B2|A0A0C3B4B2_9AGAM      -----------------------TTL----------------------------------      682
tr|A0A067MUV1|A0A067MUV1_9AGAM      ----------S-----------APSF----------------------------------      732
tr|A0A0P1BAI6|A0A0P1BAI6_9BASI      --VLP--EPSPAA-QSDTSRSTDFS------------------------TSD-------S      898
tr|A0A0D0C6Q8|A0A0D0C6Q8_9AGAR      -----------------------------------------------------GES-TVR      986
tr|A0A197JU28|A0A197JU28_9FUNG      ----------AE-----------------------ISP-I----------------ESPG      1074
tr|A0A178AI31|A0A178AI31_9PLEO      TLYQ---------G--------------------SMEPRS-----ARVD---DSFD----      266
```

```
tr|A0A316W3E2|A0A316W3E2_9BASI      --VLP--EPSPAA-QSDTSRSTDFS------------------------TSD-------S      898
tr|A0A316YEW3|A0A316YEW3_9BASI      ---EDEEEEEGEE----EEEHED-----------------EGVLSSSSNIHKVDS----D      961
tr|A0A1Y2GHB3|A0A1Y2GHB3_9FUNG      ----------IS-----------------------SEP-L----------------LADA      1116
tr|A0A6A4GV68|A0A6A4GV68_9AGAR      -----------------------------------------------------GDS-TIR      457
sp|O13821|VPS27_SCHPO               --------------------------------KTRKT-PS-----KPV---TNNED----      262
tr|A0A317T2J4|A0A317T2J4_9PEZI      VQK---------------------Q-----KPNSLMQPRN-----ARVE---DDDD----      263
tr|A0A6S6WPJ0|A0A6S6WPJ0_9PLEO      TLYQ---------G--------------------SMEPRN-----ARVE---DSFD----      266
tr|E3RQL4|E3RQL4_PYRTT              TLYQ---------G--------------------SMEPRN-----ARVE---DSFD----      266
tr|A0A0C9WXY8|A0A0C9WXY8_9AGAR      -------------------S--NLPE----------------------------------      664
tr|F8P7Z0|F8P7Z0_SERL9              ----------SEASSPFPRLTSPPTP----------------------------------      865
tr|F8Q904|F8Q904_SERL3              ----------SEASSPFPRLTSPPTP----------------------------------      762
tr|A0A292PL22|A0A292PL22_9PEZI      GQK---------------------Q-----KPNNLMQPRN-----ARVE---DDDD----      263
tr|A0A1Y1XKQ0|A0A1Y1XKQ0_9FUNG      ----------DAS---FDFNDSEF--YVN-DYMDNFDSSN------RHAAISSQEEDDIK      299
sp|Q0U4Z8|VPS27_PHANO               TLYQ---------G--------------------AMEPRS-----ARVD---DSFD----      266
tr|A0A6A6D0R8|A0A6A6D0R8_9PEZI      TLWQ---------GSSV--------------GQERMQPRG-----ARVT--EDSFD----      271
tr|A0A1Y1UY75|A0A1Y1UY75_9FUNG      ------------------------------------------------------------      641
tr|A0A6A6QQF5|A0A6A6QQF5_9PEZI      TLYQ-----------------------------GSMEPRN-----ARVE---DSFD----      265
tr|A0A168QCU4|A0A168QCU4_MUCCL      ------------------------------------------------------------      62
tr|A0A0D2PBI9|A0A0D2PBI9_HYPSF      ----------------------TSED----------------------------------      746
tr|U4LJ46|U4LJ46_PYROM              SRR---------------------V-----APSASMQPRN-----ARVE---QDDD----      266
tr|A0A2T6ZAZ1|A0A2T6ZAZ1_TUBBO      VQK---------------------Q-----KPNNLMQPRN-----ARVE---DDDD----      238
tr|A0A4U0WTC3|A0A4U0WTC3_9PEZI      TLYQ---------GY----------------TQSPMQPRS-----ARVE---DSFD----      269
tr|A0A4S8LNE8|A0A4S8LNE8_DENBC      ----------SSSV-SMERVDEDEEEKG------------------------NDED-TIR      1188
tr|A0A4V1IUA8|A0A4V1IUA8_9FUNG      -------------------------------AAA----AA-------------AEQ----      254
tr|L0P8I6|L0P8I6_PNEJ8              ------------------------------------------------------------      105
tr|R7Z380|R7Z380_CONA1              TLYQ---------GY----------------Q-GSMEPRS-----ARVE---DSFD----      265
tr|E4ZXS6|E4ZXS6_LEPMJ              TLYQ---------S--------------------SMQPRS-----ARVE---DSFD----      371
tr|A0A168Q840|A0A168Q840_MUCCL      -------------------------------PVA----AA-----APIDKKEDDYD----      261
tr|A0A0C2S3H8|A0A0C2S3H8_AMAMU      ----------S----SRSKEGSNSEN----------------------------------      801
tr|A0A0C9N4H5|A0A0C9N4H5_9FUNG      -------------------------------PVA-----A-----APIDKKEDDYD----      260
tr|R4XHH8|R4XHH8_TAPDE              PDYQ-------------------------------AQTPK-----RHVSRAGDTED----      263
tr|A0A0B7NCZ8|A0A0B7NCZ8_9FUNG      --------------RGLLELTEKDNEKD----LHNMVAYG-------------GVD-ALI      128
tr|A0A163F5K2|A0A163F5K2_DIDRA      TLYQ---------G--------------------AMEPRN-----PRVE---DSFD----      266
tr|A0A6A6YA67|A0A6A6YA67_9PEZI      TLYQ-----------------------------GSMEPRS-----ARVE---DSFD----      265
tr|A0A5C3KRI4|A0A5C3KRI4_9AGAR      ----------------------DKPK----------------------------------      701
tr|A0A0C2YUU7|A0A0C2YUU7_HEBCY      ----------------------DAER----------------------------------      758
tr|G4T9B0|G4T9B0_SERID              ---------------------GNGHL----------------------------------      911
tr|A0A367JD28|A0A367JD28_RHIAZ      -------------------------------KTT----NI-----PQP-QPENDFD----      260
tr|A0A367JX58|A0A367JX58_RHIAZ      -------------------------------KTP----NI-----PQP-QPENDFD----      260
tr|A0A0M8MVG5|A0A0M8MVG5_9BASI      ---------RLSRLYSMLRLSSSA-----------------------------TSP-TED      183
tr|S2JXE4|S2JXE4_MUCC1              -------------------------------ATA----AP-----APADKKEDDYD----      261
tr|A0A286USL7|A0A286USL7_9AGAM      ------------------------------------------------------------      633
tr|A0A067NSG1|A0A067NSG1_PLEOS      -------------------------S----------------------------------      656
tr|A0A316ZLR8|A0A316ZLR8_9BASI      --SQPLEPIEPAA-----------------------------------------------      990
tr|A0A4T0G0A2|A0A4T0G0A2_9BASI      ----------TT-------------------------P-G-----------KRRDE-DS-      833
```

```
tr|A0A075ARC6|A0A075ARC6_ROZAC      ------------------------------------------------------------      269
tr|A0A4V1IZA8|A0A4V1IZA8_ROZAC      ------------------------------------------------------------      269
tr|A0A3M7M1P8|A0A3M7M1P8_9PLEO      TLYQ---------G--------------------SMEPRN-----ARVE---DSFD----      278
tr|D5GD30|D5GD30_TUBMM              VQK---------------------Q-----KPNNLMQPRN-----ARVE---DDDD----      263
tr|A0A1X7RQY2|A0A1X7RQY2_ZYMTR      SLWQ---------GANA--------------STDRMQPRG-----ARVT--EDSFD----      271
tr|A0A1Y6LJF9|A0A1Y6LJF9_ZYMTR      SLWQ---------GANA--------------STDRMQPRG-----ARVT--EDSFD----      271
tr|A0A2H1GBB8|A0A2H1GBB8_ZYMTR      SLWQ---------GANA--------------STDRMQPRG-----ARVT--EDSFD----      271
tr|F9X9I8|F9X9I8_ZYMTI              SLWQ---------GANA--------------STDRMQPRG-----ARVT--EDSFD----      271
tr|A0A6A6A070|A0A6A6A070_9PLEO      TLYQ---------G--------------------SMEPRS-----ARVE---DSFD----      266
tr|B0DJQ4|B0DJQ4_LACBS              -------------------S--NLPE----------------------------------      664
tr|A0A369JTG7|A0A369JTG7_HYPMA      -------------------E--ISHK----------------------------------      721
tr|I4YHW0|I4YHW0_WALMC              ----------TT-------------------------H-G-----------KT-------      819
tr|A0A507CTR3|A0A507CTR3_9FUNG      ----------ING---------------------HSSDSR------NVEDVVRQEQEEFE      600
tr|F4RZR0|F4RZR0_MELLP              ----------LS-----------------------PTP-N-----------RPHDDLDLT      1033
tr|A0A4T0WVM5|A0A4T0WVM5_9ASCO      -----------------------------------ASTNK------KEIDIGEDDDDEIK      260
tr|A0A150VI72|A0A150VI72_9PEZI      TLWQ---------ESSL--VR---------DTSNRMQPRS-----ARA---EDSFD----      278
tr|A0A3M6ZNS9|A0A3M6ZNS9_HORWE      TLWQ---------DSGMGVSR---------AAEARMHPRS-----ARVDVDDSHFD----      285
tr|A0A1Y1XRY0|A0A1Y1XRY0_9FUNG      ----------------PPT-----------------VY----------------------      416
tr|A0A074WRI8|A0A074WRI8_9PEZI      S--------------------------------GRMQARD-----PRVA--EDSFD----      259
tr|A0A6A7BU37|A0A6A7BU37_9PEZI      TLWQ---------ESGIDRQP-----------EPQSQPQS-----AL---SDDRFD----      260
tr|K1WTE0|K1WTE0_TRIAC              ---------------------------------------G------RTPRSRRDFDADLQ      257
tr|J6EPC0|J6EPC0_TRIAS              ---------------------------------------G------RTPRSRRDFDADLQ      257
tr|A0A3N4JEN9|A0A3N4JEN9_9PEZI      VQK---------------------Q-----KPNNLMQPRN-----ARVE---DDDD----      263
tr|N1QCW3|N1QCW3_PSEFD              TLWQ---------DSGMGR-----------SGSERMQPRG-----ARVT--EDSFD----      280
tr|M2STQ7|M2STQ7_COCH5              TLYQ---------G--------------------SMEPRN-----ARIE---DSFD----      266
tr|N4X1G1|N4X1G1_COCH4              TLYQ---------G--------------------SMEPRN-----ARIE---DSFD----      266
tr|A0A6A5SBR1|A0A6A5SBR1_9PLEO      TLYQ---------G--------------------SMEPRN-----ARVE---DSFD----      266
tr|A0A1Y2LUT7|A0A1Y2LUT7_EPING      TLYQ---------G--------------------SMEPRN-----PRVE---DSFD----      269
tr|E2LZL4|E2LZL4_MONPE              ---------------------IDTTH----------------------------------      517
tr|A0A067S9R7|A0A067S9R7_GALM3      ----------SGSTSA--TAAADAEL----------------------------------      842
tr|L0PFS6|L0PFS6_PNEJ8              ---------------------------------------N-----------KSQSNLNVS      210
tr|F4NZK1|F4NZK1_BATDJ              ----------TTG---LGASSTT-----------STASSN------AHEDLAKKEDEDLA      280
tr|A0A3M7BVL5|A0A3M7BVL5_HORWE      TLWQ---------DSGMGVSR---------AAEARMHPRS-----ARVDVDDSHFD----      277
tr|A0A5J5F2H1|A0A5J5F2H1_9PEZI      SKR---------------------V-----T-TSSVEPRS-----ASVE---QDDD----      265
tr|A0A3M7GF14|A0A3M7GF14_HORWE      TLWQ---------DSGMGVSR---------AAEARMHPRS-----ARVDVDDSHFD----      285
tr|A0A3M7EI93|A0A3M7EI93_HORWE      TLWQ---------DSGMGVSR---------AAEARMHPRS-----ARVDVDDSHFD----      286
tr|A0A3M7A400|A0A3M7A400_HORWE      TLWQ---------DSGMGVSR---------AAEARMHPRS-----ARVDVDDSHFD----      285
tr|A0A162Y6D2|A0A162Y6D2_MUCCL      --------------RGLLELTEKDDEKD----LHNMVAYG-------------GVD-ALI      128
tr|A0A4U0TZE6|A0A4U0TZE6_9PEZI      TLWQ---------DSGMGLSS---------RAESKMHPRS-----ARLE--PESFD----      282
tr|A0A3M7DFE3|A0A3M7DFE3_HORWE      TLWQ---------DSGMGVSR---------AAEARMHPRS-----ARVDVDDSHFD----      285
tr|A0A3M7C5L4|A0A3M7C5L4_HORWE      TLWQ---------DSGMGVSR---------AAEARMHPRS-----ARVDVDDSHFD----      285
tr|A0A1Z5SW51|A0A1Z5SW51_HORWE      TLWQ---------DSGMGVSR---------AAEARMHPRS-----ARVDVDDSHFD----      285
tr|A0A3M7IAA8|A0A3M7IAA8_HORWE      TLWQ---------DSGMGVSR---------AAEARMHPRS-----ARVDVDDSHFD----      285
tr|A0A1Y1ZMB2|A0A1Y1ZMB2_9PLEO      TLYQ-----------------------------GSMEPRN-----PRVE---DSFD----      239
tr|A0A3M7EIE8|A0A3M7EIE8_HORWE      TLWQ---------DSGMGVSR---------AAEARMHPRS-----ARVDVDDSHFD----      285
```

```
tr|A0A3M7FRT0|A0A3M7FRT0_HORWE      TLWQ---------DSGTGVSR---------AAEARMHPRS-----ARVDVDDSHFD----     285
tr|A0A3M6WMK4|A0A3M6WMK4_HORWE      TLWQ---------DSGMGVSR---------AAEARMHPRS-----ARVDVDDSHFD----     285
tr|A0A0W4ZU85|A0A0W4ZU85_PNEJ7      ------------------------------------------------------------     104
tr|A0A409Y3I3|A0A409Y3I3_9AGAR      ----------------------ATYV----------------------------------     882
tr|A0A1X0QT00|A0A1X0QT00_RHIZD      -------------------------------KTP----NI-----PQP-QPENDFD----     260
tr|A0A2G4SSF8|A0A2G4SSF8_RHIZD      -------------------------------KTP----NI-----PQP-QPENDFD----     260
tr|A0A4V4JT84|A0A4V4JT84_AURPU      G--------------------------------ARMQARD-----PRVA--EDSFD----     338
tr|A0A4S9NID1|A0A4S9NID1_AURPU      G--------------------------------ARMQARD-----PRVA--EDSFD----     259
tr|A0A4V4JPE1|A0A4V4JPE1_AURPU      G--------------------------------ARMQARD-----PRVA--EDSFD----     259
tr|A0A4S8U3P8|A0A4S8U3P8_AURPU      G--------------------------------ARMQARD-----PRVA--EDSFD----     259
tr|A0A4T0CLC8|A0A4T0CLC8_AURPU      G--------------------------------ARMQARD-----PRVA--EDSFD----     259
tr|A0A4S8XK24|A0A4S8XK24_AURPU      G--------------------------------ARMQARD-----PRVA--EDSFD----     259
tr|A0A4S9PXJ6|A0A4S9PXJ6_AURPU      G--------------------------------ARMQARD-----PRVA--EDSFD----     259
tr|A0A4S9S1G3|A0A4S9S1G3_AURPU      G--------------------------------ARMQARD-----PRVA--EDSFD----     259
tr|A0A4S9X121|A0A4S9X121_AURPU      G--------------------------------ARMQARD-----PRVA--EDSFD----     259
tr|A0A4S9MIC5|A0A4S9MIC5_AURPU      G--------------------------------ARMQARD-----PRVA--EDSFD----     259
tr|A0A4T0AAT4|A0A4T0AAT4_AURPU      G--------------------------------ARMQARD-----PRVA--EDSFD----     259
tr|A0A4V4LMX5|A0A4V4LMX5_AURPU      G--------------------------------ARMQARD-----PRVA--EDSFD----     259
tr|A0A4S9MD28|A0A4S9MD28_AURPU      G--------------------------------ARMQARD-----PRVA--EDSFD----     259
tr|A0A4S9SZX6|A0A4S9SZX6_AURPU      G--------------------------------ARMQARD-----PRVA--EDSFD----     259
tr|A0A4S8TGC0|A0A4S8TGC0_AURPU      G--------------------------------VRMQARD-----PRVA--EDSFD----     259
tr|A0A4S9BBL0|A0A4S9BBL0_AURPU      G--------------------------------ARMQARD-----PRVA--EDSFD----     259
```

Plants

```
sp|Q9H8W4|PKHF2_HUMAN               PARSDSYSQSLKS---------PLNDMSDDDDDDDSSD----------------------     249
tr|A0A6T6RNY5|A0A6T6RNY5_9CHLO      -LPRMRARSVSDLAPNASALRRILDD--------EIS-----------------------     155
tr|A0A1Q3D812|A0A1Q3D812_CEPFO      QV-SHAAQSPINDLTDLSTLRSWVNFPWGQSMEYEIYKAANTIRGYDKI-----------     411
tr|A0A6J1KWW8|A0A6J1KWW8_CUCMA      GDKATCSAVSPSSSST---------SIGSTKSDYT----------CHSAISNVNSTPCSN     151
tr|A0A443PY34|A0A443PY34_9MAGN      SI-SNAVQVAKHDVMDWTCTRGWLNMPVGLSMEDEIYKSTNTLRG---------------     281
tr|A0A0K9PNH3|A0A0K9PNH3_ZOSMR      GRKSHAEKARSKLLPM--QEDENITRFLEEGGNYPSS-SSNSQADMFSDLDTVTTSTSRS     142
tr|A0A6J1EUT4|A0A6J1EUT4_CUCMO      GDKATCSAVSLSSSST---------SIGSSRSDYT----------CHSAISNVNSTPCSN     151
tr|A0A6J1EZX4|A0A6J1EZX4_CUCMO      GDKATCSAVSLSSSST---------SIGSSRSDYT----------CHSAISNVNSTPCSN     151
tr|A0A0E0E2T1|A0A0E0E2T1_9ORYZ      KI-SRACQIPTNDLTDLSTLRSWLNLPWARTMEYEIYKAVNSIYGYFK------------     350
tr|A0A0E0E2T0|A0A0E0E2T0_9ORYZ      KI-SRACQIPTNDLTDLSTLRSWLNLPWARTMEYEIYKAVNSIYGYFKLLVLSVILE--S     360
tr|I0YJ19|I0YJ19_COCSC              TV-SHAVQPAVHDVTDASALRSWFNPPMAASLAAELYKATNIVRSFA-------------     140
tr|A0A6A3A7M5|A0A6A3A7M5_HIBSY      TI-SNAAQVAKHDVMDWACTRFWLNLPVGLSMEHEIYKASNILKS---------------     150
tr|A0A7J0FIS1|A0A7J0FIS1_9ERIC      TI-SNAMQAAKHDVMDWTCSRSWMNLPVGLSMEYEIYKSSNTLRS---------------     260
tr|A0A6I9U8S6|A0A6I9U8S6_SESIN      TI-SNAAQAAKHDVMDWTCSRGWLNLPVGLSMEHEIYKSASTLRS---------------     281
tr|A0A0D3F2U0|A0A0D3F2U0_9ORYZ      RI-SRASQPPTNDVTDLSTLRSWLNFPYAHTMEYEIYKAANSLNSYCK------------     295
tr|I1NYS7|I1NYS7_ORYGL              RI-SRASQPPTNDVTDLSTLRSWLNFPYAHTMEYEIYKAANSLNSYCK------------     340
tr|A0A6P6VUP2|A0A6P6VUP2_COFAR      QV-SRAAQLPTHDLTDLSTLRSWVNFPWGQSMEYEIYKATNTIRGY--T-----------     496
tr|A0A6P6WBJ6|A0A6P6WBJ6_COFAR      QV-SRAAQLPTHDLTDLSTLRSWVNFPWGQSMEYEIYKATNTIRGY--T-----------     496
tr|A0A068TKZ1|A0A068TKZ1_COFCA      QV-SRAAQLPTHDLTDLSTLRSWVNFPWGQSMEYEIYKATNTIRGY--T-----------     496
```

```
tr|A0A5K0XTZ9|A0A5K0XTZ9_9MAGN      TI-SNAVQTAKHDVIDWTCMRGWLNMPVGLSMEQEIYKATNALIS---------------      297
tr|A0A392PZY2|A0A392PZY2_9FABA      TI-SNSVQAAKHDVMDWTCARGWLNLPIGVSMEHEIYKASNTLRN---------------      202
tr|A0A5P1EJS8|A0A5P1EJS8_ASPOF      SI-SNAMQSAKHDVMDWTCTRGWLNLPLGLSMEHEIYKAANTLRS---------------      275
tr|A0A6A3BAW0|A0A6A3BAW0_HIBSY      TI-SNAAQVAKHDVMDWTCTRGWLNLPVGLSMEHEIYKASNTLRS---------------      237
tr|A0A2Z7AUH1|A0A2Z7AUH1_9LAMI      TI-SNAVQVAKHDVMDWTCSRGWINFPLGLSMEHEIYKSANTLRS---------------      277
tr|A0A6P4A6D2|A0A6P4A6D2_ZIZJJ      SI-SNAVQVAKHDVMDWTCTRGWLNLPVGLSMEHEIYKATNTLRS---------------      285
tr|A0A7J6GHB9|A0A7J6GHB9_CANSA      QV-SHAAQLPTNDLTDLSTLRSWLNIPWGQSMEYEIYKAANTIRAY--N-----------      343
tr|A0A7J6H825|A0A7J6H825_CANSA      QV-SHAAQLPTNDLTDLSTLRSWLNIPWGQSMEYEIYKAANTIRAY--N-----------      352
tr|A0A0P0WXV7|A0A0P0WXV7_ORYSJ      KI-SRACQIPTNDLTDLSTLRSWLNLPWARTMEYEIYKAVNSIYGYFKVWPSYNSNT--C      360
tr|A0A6P6SN61|A0A6P6SN61_COFAR      TI-SNAVQVAKHDVMDWTSMRGWLNLPVGFSMEQEIYKSANTLRN---------------      278
tr|A0A426YNR2|A0A426YNR2_ENSVE      SN-SNAVQSAKHDVTDWTCMRGWLNLPVGLCMEYEIYKATNTLRS---------------      276
tr|M0TUZ8|M0TUZ8_MUSAM              SN-SNAVQSAKHDVTDWTCMRGWLNLPVGLSMEYEIYKATNTLRS---------------      276
tr|A0A2G9H231|A0A2G9H231_9LAMI      TI-SNAAQVAKHDVTDWTCTRGWLNLPVGLSMEHEIYKSANTLRT---------------      281
tr|A0A6P5XW21|A0A6P5XW21_DURZI      TI-SNAVQVAKHDVMDWTCTRGWLNLPVGLSMEHEIYKASNTLRS---------------      287
tr|A0A6A2YCQ0|A0A6A2YCQ0_HIBSY      SI-SNASQVAKHDVMDWTCTRGWLNLPVGLSMEHEIYKASNTLRS---------------      291
tr|A0A0E0LCH3|A0A0E0LCH3_ORYPU      KI-SRACQIPTNDLTDLSTLRSWLNLPWARTMEYEIYKAVNSIYGYFK------------      291
tr|A0A4D8ZKB4|A0A4D8ZKB4_SALSN      RV-SRAAQSPTHDLTDLSTLRSWLNFPWGLSMEHEIYKAANTLQGYTKCEKPVPLFV--G      290
tr|A0A4D8XQ43|A0A4D8XQ43_SALSN      RV-SRAAQSPTHDLTDLSTLRSWLNFPWGQSMEHEIYKAANTLQGYTKCEKPVPLFV--G      290
tr|A0A0E0J2X9|A0A0E0J2X9_ORYNI      SI-SNATQTAKHDVMDWTSTRGWLNLPIGLTMEHEIYKAANSVRS---------------      299
tr|A0A5N5KUW8|A0A5N5KUW8_9ROSI      QV-SNAAQLPTHDLTDLSTLRSWVNFPWGQTMEYEIYKAANTIQGY--N-----------      322
tr|I1Q300|I1Q300_ORYGL              KI-SRACQIPTNDLTDLSTLRSWLNLPWARTMEYEIYKAVNSIYGYFK------------      350
tr|A0A0D3GHQ6|A0A0D3GHQ6_9ORYZ      KI-SRACQIPTNDLTDLSTLRSWLNLPWARTMEYEIYKAVNSIYGYFK------------      350
tr|A0A0E0AAS1|A0A0E0AAS1_9ORYZ      KI-SRACQIPTNDLTDLSTLRSWLNLPWARTMEYEIYKAVNSIYGYFK------------      350
tr|Q5Z9E4|Q5Z9E4_ORYSJ              KI-SRACQIPTNDLTDLSTLRSWLNLPWARTMEYEIYKAVNSIYGYFK------------      350
tr|A0A0P0WY10|A0A0P0WY10_ORYSJ      KI-SRACQIPTNDLTDLSTLRSWLNLPWARTMEYEIYKAVNSIYGYFK------------      350
tr|A0A0E0PZ19|A0A0E0PZ19_ORYRU      KI-SRACQIPTNDLTDLSTLRSWLNLPWARTMEYEIYKAVNSIYGYFK------------      351
tr|A0A0E0PZ18|A0A0E0PZ18_ORYRU      KI-SRACQIPTNDLTDLSTLRSWLNLPWARTMEYEIYKAVNSIYGYFKAM----------      353
tr|A0A5J4Y9K4|A0A5J4Y9K4_9CHLO      TI-AQAVRPAIHDVTDGSVMRSWLNSPLSSGLESDIYKATNILNSFA-------------      257
tr|A0A0D3GHQ7|A0A0D3GHQ7_9ORYZ      KI-SRACQIPTNDLTDLSTLRSWLNLPWARTMEYEIYKAVNSIYGYFKLLVRSVILE--S      360
tr|A0A0E0AAS2|A0A0E0AAS2_9ORYZ      KI-SRACQIPTNDLTDLSTLRSWLNLPWARTMEYEIYKAVNSIYGYFKLLVRSVILE--S      360
tr|B9FTQ4|B9FTQ4_ORYSJ              KI-SRACQIPTNDLTDLSTLRSWLNLPWARTMEYEIYKAVNSIYGYFK------------      350
tr|A0A4S4EU20|A0A4S4EU20_CAMSI      QV-SRAAQLPTNDVTDLSTLRSWANFPWAQSMEYEIYKATNTIRGYN-D-----------      479
tr|A0A7J7GVA1|A0A7J7GVA1_CAMSI      QV-SRAAQLPTNDVTDLSTLRSWANFPWAQSMEYEIYKATNTIRGYN-D-----------      479
tr|A0A2R6Q5C5|A0A2R6Q5C5_ACTCC      TI-SNAMQAAKHDVMDWTCSRGWMNLPVGLSMEYEIYKSSNTLRS---------------      215
tr|A0A6J0ZNH9|A0A6J0ZNH9_9ROSI      TI-SNAVQVAKHDVMDWTCTRGWLNLPVGLSMEHEIYKASNTLRS---------------      254
tr|A0A1D1Y8J8|A0A1D1Y8J8_9ARAE      SI-SNSVQSAKHDVMDWTCSRGWLNLPLGLSMEHEIYKASNSLRS---------------      278
tr|A0A6I9RV21|A0A6I9RV21_ELAGV      SI-SNAMQSAKHDVMDWTSTRGWLNLPVGLSMEHEIYKAANTLRS---------------      277
tr|A0A6P4DQY5|A0A6P4DQY5_ARADU      TI-SNAMQVAKHDVMDWTCARGWLNLPVGLSMEHEIYKASNTLKN---------------      278
tr|A0A2R6QUJ3|A0A2R6QUJ3_ACTCC      TI-SNAMQAAKHDVMDWTCSRGWMNLPVGLSMEYEIYKSSNTLRS---------------      285
tr|A0A7J6GLE9|A0A7J6GLE9_CANSA      SV-SNAAQIAKHDVMDWTCTRGWLNLPVGLSMEHEIYKASNTLRS---------------      287
tr|A0A6G1FAM2|A0A6G1FAM2_9ORYZ      RI-SRASQPPTHDVTDLSTLRSWLNFPYAHTMEYEIYKAANSLNSYCK------------      336
tr|A0A6J0JU70|A0A6J0JU70_RAPSA      QV-SPAAQLPTHDLTDLSTLRSWVNFPWGQSMEYEIYKATNTIRGYI-S-----------      301
tr|A0A4U5Q811|A0A4U5Q811_POPAL      QV-SNAAQLPTHDLTDLSTLRSWVNFPWGQTMGYEIYKAANTIQGY--N-----------      321
tr|A0A6M2E6R0|A0A6M2E6R0_9ROSI      QV-SNAAQLPTHDLTDLSTLRSWVNFPWGQTMGYEIYKAANTIQGY--N-----------      321
tr|A0A2K1YGU9|A0A2K1YGU9_POPTR      QV-SNAAQLPTHDLTDLSTLRSWVNFPWGQTMGYEIYKAANTIQGY--N-----------      321
```

```
tr|A0A6G1FAM5|A0A6G1FAM5_9ORYZ      RI-SRASQPPTHDVTDLSTLRSWLNFPYAHTMEYEIYKAANSLNSYCK------------     336
tr|B8A231|B8A231_MAIZE              RF-SRACQLPTQDLTDLSTLRSWINTPWAVRMEYEIYKAANSIHGYCK------------     343
tr|B6SKK2|B6SKK2_MAIZE              RF-SRACQLPTQDLTDLSTLRSWINTPWAVRMEYEIYKAANSIHGYCK------------     343
tr|A0A5A7NW81|A0A5A7NW81_STRAF      RV-SRAAQVPTHDLTDLSTLRSWVNFPWGQSMEYEIYKATNTIRGYY-A-----------     349
tr|A0A0E0HS99|A0A0E0HS99_ORYNI      KI-SHACQIPTNDLTDLSTLRSWLNLPWARTMEYEIYKAVNSIYGYFK------------     350
tr|A0A5J9TB85|A0A5J9TB85_9POAL      QL-SRACQLPTQDLTDLSTLRSWLNFPWATTMEYEIYKAANSIYGYCK------------     391
tr|A0A6J0JVV9|A0A6J0JVV9_RAPSA      QV-SPAAQLPTHDLTDLSTLRSWVNFPWGQSMEYEIYKATNTIRGYI-S-----------     396
tr|A0A2G9HVA1|A0A2G9HVA1_9LAMI      QV-SRAAQLPTHDLTDLSTLRSWVNFPWGQSMEYEIYKATSTIRGY--A-----------     415
tr|A0A1D6P215|A0A1D6P215_MAIZE      RF-SRACQLPTQDLTDLSTLRSWINTPWAVRMEYEIYKAANSIHGYCK------------     503
tr|A0A3L6DHQ6|A0A3L6DHQ6_MAIZE      RF-SRACQLPTQDLTDLSTLRSWINTPWAVRMEYEIYKAANSIHGYCK------------     503
tr|A0A444D484|A0A444D484_ENSVE      AV-SKPAGLQSHE-----DLARRLQEELNK------------------------------     109
tr|A0A6N2L9V1|A0A6N2L9V1_SALVM      TI-SNAMQVAKHDVVDWTCTRGWLNLPVGFSMEHEVYKASNTLRS---------------     259
tr|A0A5N5MEC2|A0A5N5MEC2_9ROSI      TI-SNAMQVAKHDVVDWTCTRGWLNLPVGFSMEHEVYKASNTLRS---------------     259
tr|A0A6M2ESH9|A0A6M2ESH9_9ROSI      TI-SNAMQVAKHDVVDWTCTRGWLNLPLGFSMEQEVYKASNTLRS---------------     257
tr|A9PHU1|A9PHU1_POPTR              TI-SNAMQVAKHDVVDWTCTRGWLNLPVGFSMEHEVYKASNTLRS---------------     260
tr|A0A4V4H9B5|A0A4V4H9B5_MUSBA      SN-SNAVQSAKHDVTDWTCMRGWLNLPVGLSMEYEIYKATNTLRS---------------     276
tr|A0A5A7Q664|A0A5A7Q664_STRAF      TI-SNAVQVAKHDVMDWTCLRGWLNLPVGLSMEHEIYKASVTLRSYSRW-----------     285
tr|A0A3L6S5C4|A0A3L6S5C4_PANMI      AA-NRP-IVHSHE-----DLAKKLQEAMDI------------------------------     449
tr|A0A0E0JXR6|A0A0E0JXR6_ORYPU      RI-SRASQPPTHDVTDLSTLRSWLNFPYAHTMEYEIYKAANSLNSYCK------------     316
tr|A0A6U9R6X8|A0A6U9R6X8_9CHLO      -NQQTTTNKISHE-----ELTRKLQEDYDVKYAEAISKRETRKADFPRE-----------     413
tr|A0A0D9VE44|A0A0D9VE44_9ORYZ      RI-SRASQPPTHDVTDLSTLRSWLNFPYAHTMEYEIYKAANSLNSYCK------------     336
tr|A0A444ZAW7|A0A444ZAW7_ARAHY      TI-SNAVQVAKHDVMDWTCARGWLNLPVGLSMEHEIYKASNTLKN---------------     292
tr|A0A0E0CIE1|A0A0E0CIE1_9ORYZ      RI-SRASQPPTHDVTDLSTLRSWLNFPYAHTMEYEIYKAANSLNSYCK------------     339
tr|A0A445EPD8|A0A445EPD8_ARAHY      TI-SNAMQVAKHDVMDWTCARGWLNLPVGLSMEHEIYKASNTLKN---------------     292
tr|A0A0D9YPP9|A0A0D9YPP9_9ORYZ      RI-SRASQPPTHDVTDLSTLRSWLNFPYAHTMEYEIYKAANSLNSYCK------------     340
tr|B8AEK2|B8AEK2_ORYSI              RI-SRASQPPTHDVTDLSTLRSWLNFPYAHTMEYEIYKAANSLNSYCK------------     340
tr|A0A0E0G405|A0A0E0G405_ORYNI      RI-SRASQPPTHDVTDLSTLRSWLNFPYAHTMEYEIYKAANSLNSYCK------------     340
tr|A0A0E0NC57|A0A0E0NC57_ORYRU      RI-SRASQPPTHDVTDLSTLRSWLNFPYAHTMEYEIYKAANSLNSYCK------------     340
tr|A0A4U5QRQ4|A0A4U5QRQ4_POPAL      TI-SNAMQVAKHDVVDWTCTRGWLNLPLGFSMEHEVYKASNTLRS---------------     258
tr|A0A068U4E6|A0A068U4E6_COFCA      TI-SNAVQVAKHDVMDWTSMRGWLNLPVGFSMEQEIYKSANTLRN---------------     377
tr|A0A175YLZ2|A0A175YLZ2_DAUCS      QI-SRAAQLPTHDLTDLSTLRSWLNFPWGQSMEYEIYKATNTIRGY--N-----------     481
tr|D7TC91|D7TC91_VITVI              SI-SNAAQVAKHDVMDWTCTRGWLNLPVGLSMEHEIYKSANTLRS---------------     279
tr|A0A2P5CYF8|A0A2P5CYF8_PARAD      SV-SNAMQVAKHDVMDWTCTRGWLNLPVGLSMEHEIYKASNTLRS---------------     284
tr|A0A7J8V4X5|A0A7J8V4X5_9ROSI      TI-SNAVQVAKHDVMDWTCTRGWLNLPFGLSMEHEIYKASNTLRS---------------     287
tr|A0A7J8S9U3|A0A7J8S9U3_GOSDV      TI-SNAVQVAKHDVMDWTCTRGWLNLPFGLSMEHEIYKASNTLRS---------------     287
tr|A0A2K1WWT5|A0A2K1WWT5_POPTR      TI-SNAMQVAKHDVMDWTCMRGWLNLPVGLSMEHEIYKASNTLRS---------------     287
tr|A0A1U7ZHA9|A0A1U7ZHA9_NELNU      SV-SNAVQVAKHDVMDWTCTRGWLNLPVGLSMEHEIYKSSNTLRT---------------     285
tr|A0A4U5Q985|A0A4U5Q985_POPAL      TI-SNAMQVAKHDVMDWTCMRGWLNLPVGLSMEHEIYKASNTLRS---------------     289
tr|A0A6J1H961|A0A6J1H961_CUCMO      GDKATCSAISLSSSST---------SIGSTKSGYT----------CQSAISNIPSTPCSN     151
tr|A0A2J6JPZ5|A0A2J6JPZ5_LACSA      QV-SRAAQLPTYDVTDLSTLRSWLNFPWGQSMEYEIYKATNTIQGY--S-----------     345
tr|B9F4F8|B9F4F8_ORYSJ              RI-SRASQPPTHDVTDLSTLRSWLNFPYAHTMEYEIYKAANSLNSYCK------------     340
tr|Q6EUF2|Q6EUF2_ORYSJ              RI-SRASQPPTHDVTDLSTLRSWLNFPYAHTMEYEIYKAANSLNSYCK------------     340
tr|A0A0P0VGT7|A0A0P0VGT7_ORYSJ      RI-SRASQPPTHDVTDLSTLRSWLNFPYAHTMEYEIYKAANSLNSYCK------------     340
tr|A0A4D8Z2T2|A0A4D8Z2T2_SALSN      RV-SRAAQSPTHDLTDLSTLRSWLNFPWGQSMEHEIYKAANTLQGYT---KPVPLFV--S     344
tr|A0A2U1LN57|A0A2U1LN57_ARTAN      HV-SRASQLPTHDLTDLSTLRSWLNFPWGQSMEHEIYKATNIIQGY--S-----------     257
```

```
tr|A0A397XXT3|A0A397XXT3_BRACM      QV-SPAAQLPTHDLTDLSTLRSWVNFPWGQSMEYEIYKATNTIRGYI-S-----------      394
tr|A0A5D2S5L7|A0A5D2S5L7_GOSMU      SI-SNAAQVAKHDVMDWTCTRGWLNLPVGLSMEHEIYKASTTLRS---------------      289
tr|M4ERI0|M4ERI0_BRARP              GYKNRSAKGSSILRFN--QSHRL-TFGFGFK-----------------------------      114
tr|A0A5A7P127|A0A5A7P127_STRAF      -PPGRPAGLQSHE-----DLARKLQHILSHIVDLH------------EV-----------      609
tr|A0A444ES18|A0A444ES18_ENSVE      SN-SNAVQSAKHDVTDWTCMRGWLNLPVGLCMEYEIYKATNTLRS---------------      276
tr|A0A6P5ZB34|A0A6P5ZB34_DURZI      SI-SNAVQVAKHDVMDWTCTWGWLNLPVGLSMEHEIYKATNTLKS---------------      261
tr|A0A067KCV5|A0A067KCV5_JATCU      TI-SNAVQVAKHDVMDWTCSRGWLNLPVGLSMEHEIYKASNTLKS---------------      264
tr|A0A5N5KU68|A0A5N5KU68_9ROSI      GHRNRAAKGSSRMTSQ--NEDEILNKILDNDGEESSSSGQQFNTDLVSSIQRASSSASYS      144
tr|A0A2I4GWR6|A0A2I4GWR6_JUGRE      TI-SNAVQVAKHDVIDWTCTRGWLNLPLGWSMEHEIYKASNTLRS---------------      282
tr|A0A5J5B0G0|A0A5J5B0G0_9ASTE      TI-SNAVQVAKHDVMDWTCTRGWLNLPVGLLMEDEIYKSSNMLRS---------------      282
tr|A0A067GEB1|A0A067GEB1_CITSI      TI-SNAVQVAKHDVVDWTCTRGWLNLPVGLSMEYEIYKASNTLRS---------------      261
tr|A0A2P5FH90|A0A2P5FH90_TREOI      SV-SNAMQVAKHDVMDWTCTRGWLNLPVGLSMEHEIYKASNTLRS---------------      284
tr|A0A6P5ZC23|A0A6P5ZC23_DURZI      SI-SNAVQVAKHDVMDWTCTWGWLNLPVGLSMEHEIYKATNTLKS---------------      287
tr|A0A7J9HAJ7|A0A7J9HAJ7_9ROSI      TI-SNAVQVAKHDVMDWTCTRGWLNLPFGLSMEHEIYKASNTLRS---------------      287
tr|A0A5D2UUD2|A0A5D2UUD2_GOSMU      TI-SNAVQVAKHDVMDWTCTRGWLNLPFGLSMEHEIYKASNTLRS---------------      287
tr|A0A5D2Z4X4|A0A5D2Z4X4_GOSMU      TI-SNAVQVAKHDVMDWTCTRGWLNLPVGLSMEHEIYKASNTLTS---------------      287
tr|A0A7J9A5M7|A0A7J9A5M7_9ROSI      TI-SNAVQVAKHDVMDWTCTRGWLNLPFGLSMEHEIYKASNTLRS---------------      287
tr|A0A5D2CBR4|A0A5D2CBR4_GOSDA      TI-SNAVQVAKHDVMDWTCTRGWLNLPFGLSMEHEIYKASNTLRS---------------      287
tr|A0A7J8MIP5|A0A7J8MIP5_9ROSI      TI-SNAVQVAKHDVMDWTCTRGWLNLPFGLSMEHEIYKASNTLRS---------------      287
tr|A0A7J8XT08|A0A7J8XT08_GOSAI      TI-SNAVQVAKHDVMDWTCTRGWLNLPFGLSMEHEIYKASNTLRS---------------      287
tr|A0A7J9M837|A0A7J9M837_GOSSC      TI-SNAVQVAKHDVMDWTCTRGWLNLPFGLSMEHEIYKASNTLRS---------------      287
tr|A0A7J9EM10|A0A7J9EM10_9ROSI      TI-SNAVQVAKHDVMDWTCTRGWLNLPFGLSMEHEIYKASNTLRS---------------      287
tr|A0A0D2TD84|A0A0D2TD84_GOSRA      TI-SNAVQVAKHDVMDWTCTRGWLNLPFGLSMEHEIYKASNTLRS---------------      287
tr|A0A7J9JNL7|A0A7J9JNL7_9ROSI      TI-SNAVQVAKHDVMDWTCTRGWLNLPFGLSMEHEIYKASNTLRS---------------      287
tr|A0A5D2KQW7|A0A5D2KQW7_GOSTO      TI-SNAVQVAKHDVMDWTCTRGWLNLPFGLSMEHEIYKASNTLRS---------------      287
tr|A0A5D2QB35|A0A5D2QB35_GOSTO      TI-SNAVQVAKHDVMDWTCTRGWLNLPVGLSMEHEIYKASNTLRS---------------      287
tr|A0A2P5W217|A0A2P5W217_GOSBA      TI-SNAVQVAKHDVMDWTCTRGWLNLPFGLSMEHEIYKASNTLRS---------------      287
tr|A0A2P5WJU2|A0A2P5WJU2_GOSBA      TI-SNAVQVAKHDVMDWTCTRGWLNLPVGLSMEHEIYKASNTLRS---------------      287
tr|A0A0B0MVA9|A0A0B0MVA9_GOSAR      TI-SNAVQVAKHDVMDWTCTRGWLNLPVGLSMEHEIYKASNTLRS---------------      287
tr|A0A1R3IUY7|A0A1R3IUY7_9ROSI      TI-SNAVQVAKHDVMDWTCSRGWLNLPVGLSMEHEIYKASNTLRS---------------      288
tr|A0A1R3HA36|A0A1R3HA36_COCAP      TI-SNAVQVAKHDVMDWTCSRGWLNLPVGLSMEHEIYKASNTLRS---------------      288
tr|A0A151SSP4|A0A151SSP4_CAJCA      TI-SNAAQVAKHDVMDWTCARGWINLPIGLSMEHEIYKASNTLRN---------------      288
tr|I1LYB0|I1LYB0_SOYBN              TI-SNAVQGAKHDVTDWTCARGWINLPIGLSMEHEIYKASNTLRN---------------      288
tr|A0A5D2WLD7|A0A5D2WLD7_GOSMU      SI-SNAAQVAKHDVMDWTCTRGWLNLPVGLSMEHEIYKASTTLRS---------------      288
tr|A0A445HEB0|A0A445HEB0_GLYSO      TI-SNAVQGAKHDVTDWTCARGWINLPIGLSMEHEIYKASNTLRN---------------      288
tr|A0A5J5T6I5|A0A5J5T6I5_GOSBA      SI-SNAAQVAKHDVMDWTCTRGWLNLPVGLSMEHEIYKASTTLRS---------------      288
tr|A0A0B0NT31|A0A0B0NT31_GOSAR      SI-SNAAQVAKHDVMDWTCTRGWLNLPVGLSMEHEIYKASTTLRS---------------      288
tr|A0A5D2E253|A0A5D2E253_GOSDA      SI-SNAAQVAKHDVMDWTCTRGWLNLPVGLSMEHEIYKASTTLRS---------------      288
tr|A0A7J9I4D0|A0A7J9I4D0_9ROSI      SI-SNAAQVAKHDVMDWTCTRGWLNLPVGLSMEHEIYKASTTLRS---------------      289
tr|A0A7J8W2F5|A0A7J8W2F5_9ROSI      SI-SNAAQVAKHDVMDWTCTRGWLNLPVGLSMEHEIYKASTTLRS---------------      289
tr|A0A7J8T5I6|A0A7J8T5I6_GOSDV      SI-SNAAQVAKHDVMDWTCTRGWLNLPVGLSMEHEIYKASTTLRS---------------      289
tr|A0A7J9FFD9|A0A7J9FFD9_9ROSI      SI-SNAAQVAKHDVMDWTCTRGWLNLPVGLSMEHEIYKASTTLRS---------------      289
tr|A0A7J9KCR1|A0A7J9KCR1_9ROSI      SI-SNAAQVAKHDVMDWTCTRGWLNLPVGLSMEHEIYKASTTLRS---------------      289
tr|A0A7J9CVS4|A0A7J9CVS4_GOSGO      SI-SNAAQVAKHDVMDWTCTRGWLNLPVGLSMEHEIYKASTTLRS---------------      289
tr|A0A1U8I6I8|A0A1U8I6I8_GOSHI      SI-SNAAQVAKHDVMDWTCTRGWLNLPVGLSMEHEIYKASTTLRS---------------      289
```

```
tr|A0A5D2I0Z6|A0A5D2I0Z6_GOSTO      SI-SNAAQVAKHDVMDWTCTRGWLNLPVGLSMEHEIYKASTTLRS---------------      289
tr|A0A5D2S8Z8|A0A5D2S8Z8_GOSMU      SI-SNAAQVAKHDVMDWTCTRGWLNLPVGLSMEHEIYKASTTLRS---------------      289
tr|A0A0D2VIV8|A0A0D2VIV8_GOSRA      SI-SNAAQVAKHDVMDWTCTRGWLNLPVGLSMEHEIYKASTTLRS---------------      289
tr|A0A5J5NQE0|A0A5J5NQE0_GOSBA      SI-SNAAQVAKHDVMDWTCTRGWLNLPVGLSMEHEIYKASTTLRS---------------      289
tr|A0A5D2A1Z1|A0A5D2A1Z1_GOSDA      SI-SNAAQVAKHDVMDWTCTRGWLNLPVGLSMEHEIYKASTTLRS---------------      289
tr|J3LB56|J3LB56_ORYBR              RI-SRASQPPTRDVTDLSTLRSWLNFPYAHTMEYEIYKAANSLNSYCK------------      295
tr|A0A426YGZ0|A0A426YGZ0_ENSVE      SN-SNCIQSAKHDVTDWTCMRGWLNLPVGLSMEYEIYKATNILRS---------------      276
tr|A0A7J7C9A8|A0A7J7C9A8_TRIWF      QV-SRAAQLPTHDLTDLSTLRSWLNFPWGQSMEYEIYKAANIIQGY--N-----------      328
tr|A0A2P5YQD1|A0A2P5YQD1_GOSBA      SI-SNAAQVAKHDVMDWTCTRGWLNLPVGLSMEHEIYKASTTLRS---------------      288
tr|R0I6U2|R0I6U2_9BRAS              QV-SPAAQLPTHDLTDLSTLRSWVNFPWGQSMEYEIYKATNTIRGYI-T-----------      303
tr|M4EU55|M4EU55_BRARP              QV-SPAAQLPTHDLTDLSTLRSWVNFPWGQSMEYEIYKATNTIRGYI-S-----------      393
tr|A0A022QHF4|A0A022QHF4_ERYGU      QV-SRAAQLPTHDLTDLSTLRSWVNFPWGQTMEYEIYKAVNTIHGY--A-----------      409
tr|A0A6S7M578|A0A6S7M578_LACSI      QV-SRAAQLPTYDVTDLSTLRSWLNFPWGQSMEYEIYKATNTIQGY--S-----------      428
tr|A0A078GNT4|A0A078GNT4_BRANA      QV-SPAAQLPTHDLTDLSTLRSWVNFPWGQSMEYEIYKATNTIRGYI-S-----------      424
tr|A0A6N2AVW0|A0A6N2AVW0_SOLCI      QV-SRAAQLPTHDLTDLSTLRSWVNFPWGQSMEYEIYKAANTIRGY--E-----------      498
tr|A0A6G1CWD1|A0A6G1CWD1_9ORYZ      KT-SRACQLPTNDLTDLSTLRSWLNLPWAGTMEYEIYKAVNSIYGYFK------------      345
tr|A0A7J9CJB5|A0A7J9CJB5_GOSGO      GHRSRAGRGSSKPAAK--DEDDILNQILGADIKESSSLRVASNNDMTPSVARANSSSSSS      144
tr|A0A444G2C7|A0A444G2C7_ENSVE      SN-SNCIQSAKHDVTDWTCMRGWLNLPVGLSMEYEIYKATNILRS---------------      276
tr|A0A5N6QGY5|A0A5N6QGY5_9ROSI      TI-SNAMQVAKHDVMDWTCTRGWLNLPIGMSMEDEIYKASNTLKS---------------      283
tr|W9RVR7|W9RVR7_9ROSA              SV-SNAAQVAKHDVMDWTCTRGWLNLPVGLSMEHEIYKASNTLRT---------------      285
tr|A0A5B7AS41|A0A5B7AS41_DAVIN      TI-SNAMQVAKHDVTDWTCTRGWLNLPVGLLMEHEIYKSSNTLRS---------------      283
tr|A0A7J9C8P3|A0A7J9C8P3_GOSGO      TI-SNAVQVAKHDVMDWTCTRGWLNLPVGLSMEHEIYKASNTLRS---------------      287
tr|A0A1U7WIN6|A0A1U7WIN6_NICSY      TI-SNAVQAAKHDVMDWTCTRGWLNLPVGLSMEYEIYKSSNTLRT---------------      281
tr|A0A1J6IC17|A0A1J6IC17_NICAT      TI-SNAVQAAKHDVMDWTCTRGWLNLPVGLSMEYEIYKSSNTLRT---------------      281
tr|A0A1S4DM27|A0A1S4DM27_TOBAC      TI-SNAVQAAKHDVMDWTCTRGWLNLPVGLSMEYEIYKSSNTLRT---------------      281
tr|A0A6A3B545|A0A6A3B545_HIBSY      SI-SNASQVAKHDVMDWTCTRGWLNLPVGLSMEHEIYKASNTLRS---------------      290
tr|A0A7N2LTK1|A0A7N2LTK1_QUELO      SI-SNAVQVAKHDVIDWTCTRGWLNLPLGLSMEHEIYKASNTLRS---------------      292
tr|A0A2I0L5Z9|A0A2I0L5Z9_PUNGR      SI-SNAVQGAKHDVVDWTCTRGWLNLPVGLTMEHEIYKASNTLRS---------------      283
tr|A0A1R3JEB1|A0A1R3JEB1_COCAP      GYKSRAGRGSLKSAAK--DEDGMLNQILGADSKDSSSSGVASKNDKISSVQRAASSASGS      144
tr|A0A1S4AXM1|A0A1S4AXM1_TOBAC      TI-SNAVQAAKHDVMDWTCTRGWLNLPVGLSMEYEIYKSSNTLRT---------------      290
tr|A0A3L6PF97|A0A3L6PF97_PANMI      AA-NRP-IVHSHE-----DLAKKLQEAMDI------------------------------      449
tr|D7KDX7|D7KDX7_ARALL              QV-SPAAQLPTHDLTDLSTLRSWVNFPWGQSMEYEIYKATNTIRGYI-T-----------      302
tr|A0A218XH43|A0A218XH43_PUNGR      SI-SNAVQGAKHDVVDWTCTRGWLNLPVGLTMEHEIYKASNTLRS---------------      283
tr|A0A1S4AXX1|A0A1S4AXX1_TOBAC      TI-SNAVQAAKHDVMDWTCTRGWLNLPVGLSMEYEIYKSSNTLRT---------------      312
tr|A0A5N6P4Z3|A0A5N6P4Z3_9ASTR      AAASRSSGLQSHE-----DLAKKLQNLGLM------------------------------      220
tr|V7BPT2|V7BPT2_PHAVU              HV-SNAAQLPTHDLTDLSTLRSWVNFPWGQSMEYEIYKATNTIKAY--N-----------      344
tr|A0A6G1CV26|A0A6G1CV26_9ORYZ      KT-SRACQLPTNDLTDLSTLRSWLNLPWAGTMEYEIYKAVNSIYGYFK------------      345
tr|A0A2S3HIS8|A0A2S3HIS8_9POAL      RF-SRACQLPTQDLTDLSTLRSWINIPWAIKMEYEIYKAANSIYGYCK------------      342
tr|A0A2T7DXM5|A0A2T7DXM5_9POAL      RF-SRACQLPTQDLTDLSTLRSWINIPWAIKMEYEIYKAANSIYGYCK------------      342
tr|A0A3L6PEE3|A0A3L6PEE3_PANMI      RF-SRACQLPTQDLTDLSTLRSWINIPWAIKMEYEIYKAANSICGYCK------------      361
tr|A0A1S4AY23|A0A1S4AY23_TOBAC      TI-SNAVQAAKHDVMDWTCTRGWLNLPVGLSMEYEIYKSSNTLRT---------------      358
tr|A0A443PNH8|A0A443PNH8_9MAGN      SL-GKSAVQQSHE-----DLAKKLQEEMDR------------------------------      512
tr|A0A5E4E302|A0A5E4E302_PRUDU      NI-SNASQAAKHDVMDWTCTRGWLNLPVGFSMEHEIYKASNTLRS---------------      289
tr|A0A446PFI6|A0A446PFI6_TRITD      SV-SNATQTAKHDVMDWTSARGWLNLPIGLTLEHEIYKAAVSIRS---------------      300
tr|A0A438E5E5|A0A438E5E5_VITVI      QV-SRAAQSPTYDLTDLSTLRSWLNFPWGQSMEYEIYKAANTIRNSF-S-----------      157
```

```
tr|A0A7J9AYN2|A0A7J9AYN2_9ROSI      SI-SNAAQVAKHDVMDWTCTRGWLNLPVGLSMEHEIYKASTTLRS---------------     289
tr|A0A067G5B6|A0A067G5B6_CITSI      TI-SNAVQVAKHDVVDWTCTRGWLNLPVGLSMEYEIYKASNTLRS---------------     261
tr|A0A6V7P3V7|A0A6V7P3V7_ANACO      SI-SNATQLAKHDVIDWTCARGWLNLPVGLSMDHEIYKAANTLRS---------------     228
tr|A0A2K3L4T2|A0A2K3L4T2_TRIPR      TI-SNSVQAAKHDVMDWTCARGWLNLPIGVSMEHEIYKASNTLRN---------------     292
tr|A0A5E4E497|A0A5E4E497_PRUDU      NI-SNASQAAKHDVMDWTCTRGWLNLPVGFSMEHEIYKASNTLRS---------------     289
tr|A0A1W0VTG3|A0A1W0VTG3_SORBI      RF-SRACQLPTQDLTDLSTLRSWINIPWAVRMEYEIYKAANSIHGYCG------------     342
tr|A0A5K0Z6Z6|A0A5K0Z6Z6_9MAGN      KV-SHASQLATHDLTDLSTLRSWVNFPWGQSMEYEIYKATNTIRGYCK------------     301
tr|A0A0A0LWS3|A0A0A0LWS3_CUCSA      SI-SNAVQRAKHDVMDWTCSRGWLNLPIGLSMEHEIYKASQTLRG---------------     274
tr|A0A1D6HX11|A0A1D6HX11_MAIZE      SV-SNASQTAKHDVMDWTCARGWLNFPIGLTMEHEIYKAANTLTS---------------     300
tr|A0A2H3XIR1|A0A2H3XIR1_PHODC      SN-SNAMQSAKHDVMDWTSTRGWLNLPVGLSMEHEIYKAANTLRS---------------     277
tr|A0A6P5GTM1|A0A6P5GTM1_ANACO      SI-SNATQLAKHDVIDWTCARGWLNLPVGLSMDHEIYKAANTLRS---------------     278
tr|A0A199VRL4|A0A199VRL4_ANACO      SI-SNATQLAKHDVIDWTCARGWLNLPVGLSMDHEIYKAANTLRS---------------     278
tr|A0A022QAU6|A0A022QAU6_ERYGU      TI-SNAAQVAKHDVMDWTCSRGWLNLPVGLSLEHEIYKSCNTLRS---------------     281
tr|A0A371GJB6|A0A371GJB6_MUCPR      TI-SNAAQVAKHDVMDWTCARGWINLPIGLSMEHEIYKASNTLRN---------------     288
tr|A0A251IZI6|A0A251IZI6_MANES      TI-SNAVQAAKHDVMDWTCTRGWLNLPVGLSMEHEIYKASNTLRS---------------     285
tr|A0A7J7HWK5|A0A7J7HWK5_CAMSI      TI-SNAVQVAKHDVMDWTSMRGWLNLPVGLSMEYEIYKSSNTLRS---------------     285
tr|A0A5C7GVA1|A0A5C7GVA1_9ROSI      SI-SNAVQVAKHDVVDWTCTRGWLNLPVGMSMEYEIYKASNTLRS---------------     287
tr|A0A1S2YPT9|A0A1S2YPT9_CICAR      TI-SNAVQVAKHDVMDWTSARGWLNLPIGLSMEHEIYKASNTLQN---------------     288
tr|A0A5N5I234|A0A5N5I234_9ROSA      SI-SNAAQAAKHDVMDWTCTRGWLNLPVGFSMEQEIYKASNTLRS---------------     289
tr|A0A7J9AWW0|A0A7J9AWW0_9ROSI      SI-SNAAQVAKHDVMDWTCTRGWLNLPVGLSMEHEIYKASTTLRS---------------     289
tr|A0A7J8NBC7|A0A7J8NBC7_9ROSI      SI-SNAAQVAKHDVMDWTCTRGWLNLPVGLSMEHEIYKASTTLRS---------------     289
tr|A0A7J8YJZ0|A0A7J8YJZ0_GOSAI      SI-SNAAQVAKHDVMDWTCTRGWLNLPVGLSMEHEIYKASTTLRS---------------     289
tr|A0A7J9MU50|A0A7J9MU50_GOSSC      SI-SNAAQVAKHDVMDWTCTRGWLNLPVGLSMEHEIYKASTTLRS---------------     289
tr|A0A5E4E3G6|A0A5E4E3G6_PRUDU      NI-SNASQAAKHDVMDWTCTRGWLNLPVGFSMEHEIYKASNTLRS---------------     289
tr|A0A4U6V1J0|A0A4U6V1J0_SETVI      RF-SRACQFPTQDLTDLSTLRSWINIPWATKMEYEIYKAANSIYGYCK------------     346
tr|A0A3B6EB49|A0A3B6EB49_WHEAT      SV-SNATQTAKHDVMDWTSARGWLNLPIGLTLEHEIYKAAASLRS---------------     298
tr|A0A453E2E6|A0A453E2E6_AEGTS      SV-SNATQTAKHDVMDWTSARGWLNLPIGLTLEHEIYKAAVSLRS---------------     300
tr|A3BIJ2|A3BIJ2_ORYSJ              SI-SNATQTAKHDVMDWTSTRGWLNLPIGLTMEHEIYKAANSVRS---------------     299
tr|A2YK60|A2YK60_ORYSI              SI-SNATQTAKHDVMDWTSTRGWLNLPIGLTMEHEIYKAANSVRS---------------     299
tr|A0A0E0LJ79|A0A0E0LJ79_ORYPU      SI-SNATQTAKHDVMDWTSTRGWLNLPIGLTMEHEIYKAANSVRS---------------     299
tr|A0A2S3GXE8|A0A2S3GXE8_9POAL      SV-SNASQTAKHDVMDWTCARGWLNLPIGLTMEHEIYKAANTLMS---------------     300
tr|A0A3B6FGI8|A0A3B6FGI8_WHEAT      SV-SNATQTAKHDVMDWTSARGWLNLPIGLTLEHEIYKAAVSIRS---------------     300
tr|A0A3B6GNS6|A0A3B6GNS6_WHEAT      SV-SNATQTAKHDVMDWTSARGWLNLPIGLTLEHEIYKAAVSLRS---------------     300
tr|C5X3I0|C5X3I0_SORBI              SV-SNASQTAKHDVMDWTCARGWLNLPIGLTMEHEIYKAANTLSS---------------     300
tr|A0A2T7EN04|A0A2T7EN04_9POAL      SV-SNASQTAKHDVMDWTCARGWLNLPIGLTMEHEIYKAANTLMS---------------     300
tr|A0A453E2Q5|A0A453E2Q5_AEGTS      SV-SNATQTAKHDVMDWTSARGWLNLPIGLTLEHEIYKAAVSLRS---------------     300
tr|M8BMH1|M8BMH1_AEGTA              SV-SNATQTAKHDVMDWTSARGWLNLPIGLTLEHEIYKAAVSLRS---------------     300
tr|A0A0E0AHZ8|A0A0E0AHZ8_9ORYZ      SI-SNATQTAKHDVMDWTSTRGWLNLPIGLTMEHEIYKAANSVRS---------------     301
tr|A0A0E0EA20|A0A0E0EA20_9ORYZ      SI-SNATQTAKHDVMDWTSMRGWLNLPIGLTMEHEIYKAANSVRS---------------     301
tr|A0A0E0Q6I8|A0A0E0Q6I8_ORYRU      SI-SNATQTAKHDVMDWTSTRGWLNLPIGLTMEHEIYKAANSVRS---------------     301
tr|I1Q9P1|I1Q9P1_ORYGL              SI-SNATQTAKHDVMDWTSTRGWLNLPIGLTMEHEIYKAANSVRS---------------     301
tr|A0A0P0X4Z1|A0A0P0X4Z1_ORYSJ      SI-SNATQTAKHDVMDWTSTRGWLNLPIGLTMEHEIYKAANSVRS---------------     301
tr|Q6Z4J7|Q6Z4J7_ORYSJ              SI-SNATQTAKHDVMDWTSTRGWLNLPIGLTMEHEIYKAANSVRS---------------     301
tr|A0A0D3GPH8|A0A0D3GPH8_9ORYZ      SI-SNATQTAKHDVMDWTSTRGWLNLPIGLTMEHEIYKAANSVRS---------------     301
tr|A0A5J9VT31|A0A5J9VT31_9POAL      SV-SNASQTAKHDVMDWTCSRGWLNLPIGLTMEHEIYKAANSLRI---------------     302
```

```
tr|A0A5J9VVN9|A0A5J9VVN9_9POAL      SV-SNASQTAKHDVMDWTCSRGWLNLPIGLTMEHEIYKAANSLRI---------------      302
tr|A0A2S3HG76|A0A2S3HG76_9POAL      AA-NRP-IVHSHE-----DLAKKLQEAMDI------------------------------      443
tr|A0A2T7DU10|A0A2T7DU10_9POAL      AA-NRP-IVHSHE-----DLAKKLQEAMDI------------------------------      445
tr|T1L7X1|T1L7X1_TRIUA              SV-SNATQTAKHDVMDWTSARGWLNLPIGLTLEHEIYKAAVSLRS---------------      298
tr|A0A446N5P4|A0A446N5P4_TRITD      SV-SNATQTAKHDVMDWTSARGWLNLPIGLTLEHEIYKAAASLRS---------------      298
tr|I1GY35|I1GY35_BRADI              KI-SRACQLPTKDLTDLSTLRSWINIPWAHTMEYEIYKAANSIHGYCK------------      332
tr|A0A0R0FGY4|A0A0R0FGY4_SOYBN      TI-SNAAQGAKHDVMDWTCARGWINLPIGLSMEHEIYKASNTLRN---------------      288
```

Nonhuman mammals

```
sp|Q9H8W4|PKHF2_HUMAN               -----------------NDMSDDDDDDDS--SD---------------------------      249
tr|G3RXJ3|G3RXJ3_GORGO              -----------------NDMSDDDDDDDS--SD---------------------------      249
tr|A0A2R8ZRE3|A0A2R8ZRE3_PANPA      -----------------NDMSDDDDDDDS--SD---------------------------      249
tr|A0A6D2WS13|A0A6D2WS13_PANTR      -----------------NDMSDDDDDDDS--SD---------------------------      249
tr|H2QWG2|H2QWG2_PANTR              -----------------NDMSDDDDDDDS--SD---------------------------      249
tr|A0A2K6B0W2|A0A2K6B0W2_MACNE      -----------------NDMSDDDDDDDS--SD---------------------------      249
tr|A0A2K6JSQ3|A0A2K6JSQ3_RHIBE      -----------------NDMSDDDDDDDS--SD---------------------------      249
tr|A0A2K5UMP8|A0A2K5UMP8_MACFA      -----------------NDMSDDDDDDDS--SD---------------------------      249
tr|G7PC92|G7PC92_MACFA              -----------------NDMSDDDDDDDS--SD---------------------------      249
tr|A0A2I3GPW4|A0A2I3GPW4_NOMLE      -----------------NDMSDDDDDDDS--SD---------------------------      249
tr|A0A2K5XAN3|A0A2K5XAN3_MANLE      -----------------NDMSDDDDDDDS--SD---------------------------      249
tr|A0A2K5KIW2|A0A2K5KIW2_CERAT      -----------------NDMSDDDDDDDS--SD---------------------------      249
tr|A0A2J8UGL7|A0A2J8UGL7_PONAB      -----------------NDMSDDDDDDDS--SD---------------------------      249
tr|G7MZT0|G7MZT0_MACMU              -----------------NDMSDDDDDDDS--SD---------------------------      249
tr|A0A2K5PKC3|A0A2K5PKC3_CEBIM      -----------------NDMSDDDDDDDS--SD---------------------------      249
tr|A0A2K6NLA2|A0A2K6NLA2_RHIRO      -----------------NDMSDDDDDDDS--SD---------------------------      249
tr|F7ASZ1|F7ASZ1_MACMU              -----------------NDMSDDDDDDDS--SD---------------------------      249
tr|A0A6J3FGX3|A0A6J3FGX3_SAPAP      -----------------NDMSDDDDDDDS--SD---------------------------      249
tr|A0A6D2X014|A0A6D2X014_PONAB      -----------------NDMSDDDDDDDS--SD---------------------------      249
tr|A0A6I9ICZ8|A0A6I9ICZ8_VICPA      -----------------NDVSDDDDDDDS--SD---------------------------      249
tr|S9WGC9|S9WGC9_CAMFR              -----------------NDVSDDDDDDDS--SD---------------------------      249
tr|A0A5N4CHZ2|A0A5N4CHZ2_CAMDR      -----------------NDVSDDDDDDDS--SD---------------------------      249
tr|A0A2K5HBE8|A0A2K5HBE8_COLAP      -----------------NDMSDDDDDDDS--SD---------------------------      249
tr|A0A2K5C657|A0A2K5C657_AOTNA      -----------------NDMSDDDDDDDS--SD---------------------------      249
tr|A0A096MLM1|A0A096MLM1_PAPAN      -----------------NDMSDDDDDDDS--SD---------------------------      249
tr|F7FEC3|F7FEC3_CALJA              -----------------NDMSDDDEDDDS--SD---------------------------      249
tr|A0A0D9SB87|A0A0D9SB87_CHLSB      -----------------NDMSDDDDDDDS--SD---------------------------      249
tr|A0A2K6S793|A0A2K6S793_SAIBB      -----------------NDMSDDDDDDDS--SD---------------------------      249
tr|A0A3Q7TWS2|A0A3Q7TWS2_VULVU      -----------------NDVSDDDDDDDS--SD---------------------------      249
tr|A0A1S3AB18|A0A1S3AB18_ERIEU      -----------------NDVSDDDDDDDS--SD---------------------------      249
tr|A0A5E4BFX5|A0A5E4BFX5_MARMO      -----------------NDVSDDDDDDDS--SD---------------------------      249
tr|I3MAC4|I3MAC4_ICTTR              -----------------NDVSDDDDDDDS--SD---------------------------      249
tr|A0A1U7T0A4|A0A1U7T0A4_CARSF      -----------------NDVSDDDDDDDS--SD---------------------------      249
tr|A0A2K6ERG1|A0A2K6ERG1_PROCO      -----------------NDVSDDDDDDDS--SD---------------------------      249
```

```
tr|A0A6I9JBJ2|A0A6I9JBJ2_CHRAS      -----------------NDVSDDDDDDDS--SD---------------------------      249
tr|A0A3Q7P8U5|A0A3Q7P8U5_CALUR      -----------------NDVSDDDDDDDS--SD---------------------------      249
tr|A0A3P4LK23|A0A3P4LK23_GULGU      -----------------NDVSDDDDDDDS--SD---------------------------      249
tr|M3Z4K3|M3Z4K3_MUSPF              -----------------NDVSDDDDDDDS--SD---------------------------      249
tr|A0A485MJJ6|A0A485MJJ6_LYNPA      -----------------NDVSDDDDDDDS--SD---------------------------      249
tr|A0A667IBC1|A0A667IBC1_LYNCA      -----------------NDVSDDDDDDDS--SD---------------------------      249
tr|A0A4X1U327|A0A4X1U327_PIG        -----------------NDVSDDDDDDDS--SD---------------------------      249
tr|U6CRZ6|U6CRZ6_NEOVI              -----------------NDVSDDDDDDDS--SD---------------------------      249
tr|A0A2Y9EYL3|A0A2Y9EYL3_PHYMC      -----------------NDVSDDDDDDDS--SD---------------------------      249
tr|M3WVQ3|M3WVQ3_FELCA              -----------------NDVSDDDDDDDS--SD---------------------------      249
tr|A0A7J8DUD9|A0A7J8DUD9_MOLMO      -----------------NDVSDDDDDDDS--SD---------------------------      249
tr|A0A6P6HRR1|A0A6P6HRR1_PUMCO      -----------------NDVSDDDDDDDS--SD---------------------------      249
tr|A0A287ASA9|A0A287ASA9_PIG        -----------------NDVSDDDDDDDS--SD---------------------------      249
tr|A0A6J1ZRF1|A0A6J1ZRF1_ACIJB      -----------------NDVSDDDDDDDS--SD---------------------------      249
tr|A0A6P4WXG5|A0A6P4WXG5_PANPR      -----------------NDVSDDDDDDDS--SD---------------------------      249
tr|A0A5N3XBU7|A0A5N3XBU7_MUNRE      -----------------NDVSDDDDDDDS--SD---------------------------      249
tr|A0A5N3WEU8|A0A5N3WEU8_MUNMU      -----------------NDVSDDDDDDDS--SD---------------------------      249
tr|L8ILX8|L8ILX8_9CETA              -----------------NDVSDDDDDDDS--SD---------------------------      249
tr|A4IFN8|A4IFN8_BOVIN              -----------------NDVSDDDDDDDS--SD---------------------------      249
tr|A0A4W2EB56|A0A4W2EB56_BOBOX      -----------------NDVSDDDDDDDS--SD---------------------------      249
tr|A0A6P5CTL6|A0A6P5CTL6_BOSIN      -----------------NDVSDDDDDDDS--SD---------------------------      249
tr|A0A6J0WJ70|A0A6J0WJ70_ODOVR      -----------------NDVSDDDDDDDS--SD---------------------------      249
tr|A0A6P3GQJ4|A0A6P3GQJ4_BISBI      -----------------NDVSDDDDDDDS--SD---------------------------      249
tr|A0A2U3WPC7|A0A2U3WPC7_ODORO      -----------------NDVSDDDDDDDS--SD---------------------------      249
tr|A0A7J7VF05|A0A7J7VF05_RHIFE      -----------------NDVSDDDDDDDS--SD---------------------------      249
tr|L5KAU6|L5KAU6_PTEAL              -----------------NDVSDDDDDDDS--SD---------------------------      249
tr|A0A6P3QVM8|A0A6P3QVM8_PTEVA      -----------------NDVSDDDDDDDS--SD---------------------------      249
tr|A0A2Y9PAZ8|A0A2Y9PAZ8_DELLE      -----------------NDVSDDDDDDDS--SD---------------------------      249
tr|G1PK15|G1PK15_MYOLU              -----------------NDVSDDDDDDDS--SD---------------------------      249
tr|A0A7J7TK50|A0A7J7TK50_MYOMY      -----------------NDVSDDDDDDDS--SD---------------------------      249
tr|A0A2Y9GX76|A0A2Y9GX76_NEOSC      -----------------NDVSDDDDDDDS--SD---------------------------      249
tr|L5MHS6|L5MHS6_MYODS              -----------------NDVSDDDDDDDS--SD---------------------------      249
tr|A0A2U3XES2|A0A2U3XES2_LEPWE      -----------------NDVSDDDDDDDS--SD---------------------------      249
tr|A0A7J7VN67|A0A7J7VN67_PIPKU      -----------------NDVSDDDDDDDS--SD---------------------------      249
tr|A0A452QFV9|A0A452QFV9_URSAM      -----------------NDVSDDDDDDDS--SD---------------------------      249
tr|A0A6G1APQ5|A0A6G1APQ5_CROCR      -----------------NDVSDDDDDDDS--SD---------------------------      249
tr|A0A340WWB4|A0A340WWB4_LIPVE      -----------------NDVSDDDDDDDS--SD---------------------------      249
tr|A0A3Q7WAC4|A0A3Q7WAC4_URSAR      -----------------NDVSDDDDDDDS--SD---------------------------      249
tr|A0A250XXA1|A0A250XXA1_CASCN      -----------------NDVSDDDDDDDS--SD---------------------------      249
tr|G9KH59|G9KH59_MUSPF              -----------------NDVSDDDDDDDS--S----------------------------      248
tr|A0A1U7QHL5|A0A1U7QHL5_MESAU      -----------------NDVSDDDDDDDS--SD---------------------------      249
tr|A0A452CBQ1|A0A452CBQ1_BALAS      -----------------NDVSDDDDDDDS--SD---------------------------      249
tr|A0A2Y9QET1|A0A2Y9QET1_TRIMA      -----------------NDVSDDDDDDDS--SD---------------------------      249
tr|A0A6P5PKF8|A0A6P5PKF8_MUSCR      -----------------NDASDDDDDDDS--SD---------------------------      249
tr|A0A341CEL5|A0A341CEL5_NEOAA      -----------------NDVSDDDDDDDS--SD---------------------------      249
```

```
tr|A0A484GRA3|A0A484GRA3_SOUCH      -----------------NDVSDDDDDDDS--SD---------------------------      249
tr|A0A7J8C3Q9|A0A7J8C3Q9_ROUAE      -----------------NDVSDDDDDDDS--SD---------------------------      249
tr|A0A384BXS8|A0A384BXS8_URSMA      -----------------NDVSDDDDDDDS--SD---------------------------      249
tr|A0A2U4AI60|A0A2U4AI60_TURTR      -----------------NDVSDDDDDDDS--SD---------------------------      249
tr|A0A2Y9J9Y4|A0A2Y9J9Y4_ENHLU      -----------------NDVSDDDDDDDS--SD---------------------------      249
tr|L8YGB7|L8YGB7_TUPCH              -----------------NDVSDDDDDDDS--SD---------------------------      250
tr|G3UCQ4|G3UCQ4_LOXAF              -----------------NDVSDDDDDDDS--SD---------------------------      249
tr|D2HVB5|D2HVB5_AILME              -----------------NDVSDDDDDDDS--SD---------------------------      249
tr|A0A6I9L8U8|A0A6I9L8U8_PERMB      -----------------NDVSDDDDDDDS--SD---------------------------      249
tr|A0A1S3EVN9|A0A1S3EVN9_DIPOR      -----------------NDGSDDDDDDDS--SD---------------------------      249
tr|A0A2K5C233|A0A2K5C233_AOTNA      -----------------NDMSDDDDDDDS--SD---------------------------      249
tr|H0Y0B3|H0Y0B3_OTOGA              -----------------NDVSDDDDDDDS--SD---------------------------      249
tr|A0A6J2NCI4|A0A6J2NCI4_9CHIR      -----------------NDVSDDDDDDDS--SD---------------------------      249
sp|Q91WB4|PKHF2_MOUSE               -----------------NDASDDDDDDDS--SD---------------------------      249
tr|A0A7J7EHJ9|A0A7J7EHJ9_DICBM      -----------------NDVSDDDDDDDS--SD---------------------------      249
tr|K9IHW6|K9IHW6_DESRO              -----------------NDVSDDDDDDDS--SD---------------------------      249
tr|A0A452DYL3|A0A452DYL3_CAPHI      -----------------NDVSDDDDDDDS--SD---------------------------      249
tr|A0A3Q2HWW6|A0A3Q2HWW6_HORSE      -----------------NDVSDDDDDDDS--SD---------------------------      249
tr|W5PZ70|W5PZ70_SHEEP              -----------------NDVSDDDDDDDS--SD---------------------------      249
tr|A0A6P7ECY0|A0A6P7ECY0_SHEEP      -----------------NDVSDDDDDDDS--SD---------------------------      249
tr|B1WBV4|B1WBV4_RAT                -----------------NDASDDDDDDDS--SD---------------------------      249
tr|A0A6J2F8G3|A0A6J2F8G3_ZALCA      -----------------NDVSDDDDDDDS--SD---------------------------      248
tr|A0A7N4NQ24|A0A7N4NQ24_SARHA      -----------------NNVSDDEDDEDS--SD---------------------------      249
tr|A0A6P5IQU3|A0A6P5IQU3_PHACI      -----------------NNVSDDEDDEDS--SD---------------------------      249
tr|A0A286XYJ1|A0A286XYJ1_CAVPO      -----------------NNVSDDDDDDSS--D----------------------------      248
tr|A0A5E4BEY9|A0A5E4BEY9_MARMO      -----------------NDVLDDDDDDDS--SD---------------------------      249
tr|A0A091DW22|A0A091DW22_FUKDA      -----------------NDVSDDEDDDSS--D----------------------------      248
tr|A0A0P6K1S8|A0A0P6K1S8_HETGA      -----------------NNVSDDEDDDSS--D----------------------------      248
tr|F7A285|F7A285_ORNAN              -----------------NNTSDDDDDDSS--SD---------------------------      249
tr|F6Q9Z2|F6Q9Z2_MONDO              -----------------NNVSDDED------DEDSS----------D-------------      543
tr|A0A6P3FWN8|A0A6P3FWN8_OCTDE      -----------------NDVSDDDEEDSS--D----------------------------      248
tr|A0A6A1QJM0|A0A6A1QJM0_BALPH      -----------------NDVSDDDDDDDD--DDDDD----------SSD-----------      255
tr|A0A673V5I4|A0A673V5I4_SURSU      -----------------NQHYLKKTMKTE--KC---------------------------      249
tr|A0A6J0YZI7|A0A6J0YZI7_ODOVR      ------------------------------------------------------------      229
tr|F7F262|F7F262_MONDO              ------CSTL-SYW---LQ-----------------------------------------      238
tr|A0A5F8H7A3|A0A5F8H7A3_MONDO      ------CQSK-G-----RDVRTASPGTGS--QVQSA----------DSFPV---------      259
tr|A0A5F8GYD7|A0A5F8GYD7_MONDO      ISILAILAAI-SYL---FPPGTHTPHSGS--SE---------------------------      264
tr|A8Y5N8|A8Y5N8_MOUSE              ------------------------------------------------------------      197
tr|A0A5N3XKB2|A0A5N3XKB2_MUNRE      -----------------NDVSDDDD------DDSSD-------------------------      213
tr|A0A1A6FXD9|A0A1A6FXD9_NEOLE      -----------------NDVSDDDD------DDDSS----------D-------------      227
tr|A0A6B0RRU8|A0A6B0RRU8_9CETA      -----------------NDVSDDDD------DDDSS----------D-------------      465
tr|A0A5N3UY12|A0A5N3UY12_MUNMU      -----------------NDVSDDDD------DDDDD----------SSD-----------      204
tr|A0A0D9SB08|A0A0D9SB08_CHLSB      HLTGPICGASSG---DDDDSDEDKEGSRD--GDWPSRVEFY--GSGVAWSAFHS------      279
tr|A0A2J8KSM2|A0A2J8KSM2_PANTR      HLAGPICGASSG---DDDDSDEDKEGSRD--GDWPSRVEFY--ASGVAWSAFHS------      279
sp|Q96S99|PKHF1_HUMAN               HLARPICGASSG---DDDDSDEDKEGSRD--GDWPSSVEFY--ASGVAWSAFHS------      279
```

```
tr|A0A2K5JGW8|A0A2K5JGW8_COLAP      HLTRPICGASSG---DDDDSDEDKEGSRD--GDWPSRVEFY--GSGVAWSAFHS------      279
tr|A0A2K6CYZ9|A0A2K6CYZ9_MACNE      HLTRPICGASSG---DDDDSDEDKEGSRD--GDWPSRVEFY--GSGVAWSAFHS------      279
tr|A0A2K6JWD4|A0A2K6JWD4_RHIBE      HLTRPICGASSG---DDDDSDEDKEGSRD--GDWPSRVEFY--GSGVAWSAFHS------      279
tr|A0A2K5UDM0|A0A2K5UDM0_MACFA      HLTRPICGASSG---DDDDSDEDKEGSRD--GDWPSHVEFY--GSGVAWSAFHS------      279
tr|G3QH50|G3QH50_GORGO              HLARPICGASSG---DDDDSDEDKEGSRD--GDWPSRVEFY--ASGVAWSAFHS------      279
tr|A0A2I3G625|A0A2I3G625_NOMLE      HLARPICGASSG---DDDDSDEDKEGSRD--GDWPSRVEFY--ASGVAWSAFHS------      279
tr|A0A2K5XLS1|A0A2K5XLS1_MANLE      HLTRPICGASSG---DDDDSDEDKEGSRD--GDWPSRVEFY--GSGVAWSAFHS------      279
tr|A0A0A0MVG7|A0A0A0MVG7_PAPAN      HLTRPICGASSG---DDDDSDEDKEGSRD--GDWPSRVEFY--GSGVAWSAFHS------      279
tr|A0A2K5KX81|A0A2K5KX81_CERAT      HLTRPICGASSG---DDDDSDEDKEGSRD--GDWPSRVEFY--GSGVAWSAFHS------      279
tr|G7NLE7|G7NLE7_MACMU              HLTRPICGASSG---DDDDSDEDKEGSRD--GDWPSRVEFY--GSGVAWSAFHS------      279
tr|A0A2K6NPM4|A0A2K6NPM4_RHIRO      HLTRPICGASSG---DDDDSDEDKEGSRD--GDWPSRVEFY--GSGVAWSAFHS------      279
tr|F6WEV7|F6WEV7_MACMU              HLTRPICGASSG---DDDDSDEDKEGSRD--GDWPSRVEFY--GSGVAWSAFHS------      279
tr|H2QFY5|H2QFY5_PANTR              HLARPICGASSG---DDDDSDEDKEGSRD--GDWPSRVEFY--ASGVAWSAFHS------      279
tr|A0A2J8SAC5|A0A2J8SAC5_PONAB      HLARPICGASSG---DDDDSDEDKEGSRD--GDWPSRVEFY--ASGVAWSAFHS------      279
tr|A0A6D2Y8X4|A0A6D2Y8X4_PONAB      HLARPICGASSG---DDDDSDEDKEGSRD--GDWPSRVEFY--ASGVAWSAFHS------      279
tr|G7PX49|G7PX49_MACFA              HLTRPICGASSG---DDDDSDEDKEGSRD--GDWPSRVEFY--GSGVAWSAFHS------      279
tr|A0A2R9AA53|A0A2R9AA53_PANPA      HLAGPICGASSG---DDDDSDEDKEGSRD--GDWPSRVEFY--ASGVAWSAFHS------      364
tr|B4DWN9|B4DWN9_HUMAN              HLARPICGASSG---DDDDSDEDKEGSRD--GDWPSSVEFY--ASGVAWSAFHS------      364
tr|L8IB21|L8IB21_9CETA              HLAGAVCGASSG---DDDDSDEDREGSGD--GDWPSHVEFY--ASGVSWSSFHS------      279
tr|A2VE19|A2VE19_BOVIN              HLAGAVCGASSG---DDDDSDEDREGSGD--GDWPSHVEFY--ASGVSWSSFHS------      279
tr|A0A4W2H1W7|A0A4W2H1W7_BOBOX      HLAGAVCGASSG---DDDDSDEDREGSGD--GDWPSHVEFY--ASGVSWSSFHS------      279
tr|A0A6P5DDG5|A0A6P5DDG5_BOSIN      HLAGAVCGASSG---DDDDSDEDREGSGD--GDWPSHVEFY--ASGVSWSSFHS------      279
tr|A0A091D210|A0A091D210_FUKDA      HLGGAFYGASSGDDGDDDDSDEDKEGSRD--GDWPSRVQFY--ASGVSWSAFHS------      282
tr|A0A6B0R7Q5|A0A6B0R7Q5_9CETA      HLAGAVCGASSG---DDDDSDEDREGSGD--GDWPSHVEFY--ASGVSWSSFHS------      298
tr|A0A2K5PK00|A0A2K5PK00_CEBIM      HLTRAICGASSG---DDDDSDEDKEGSGD--GDWSSRVEFY--SSGVAWSAFHS------      279
tr|A0A1U7R682|A0A1U7R682_MESAU      LGG-TVSGASSG---DDDDSDEDREGSGD--GDWPSQVEFY--TSGVSWSAFHS------      279
tr|A0A2K6SFK7|A0A2K6SFK7_SAIBB      HLARAICGASSG---DDDDSDEDKEGSGD--GDWSSRVEFY--SSGVAWSAFHS------      279
tr|A0A2K5E2Y5|A0A2K5E2Y5_AOTNA      HLARAICGASSG---DDDDSDEDKEGSGD--GDWSSRVEFY--SSGVAWSAFHS------      279
tr|U3D2H9|U3D2H9_CALJA              HLARAICGASSG---DDDDSDEDKEGSGD--GDWSSRVEFY--SSGVAWSAFHS------      279
tr|A0A6J3FHK1|A0A6J3FHK1_SAPAP      HLTRAICGASSG---DDDDSDEDKEGSGD--GDWSSRVEFY--SSGVAWSAFHS------      279
tr|H0XH62|H0XH62_OTOGA              LAGAVCGGASSG---DDDDSEEDREGGGD--GDWPSSVEFY--ASDVSWSAFHS------      280
tr|A0A6J1Y5U6|A0A6J1Y5U6_ACIJB      YLAGAICGASSG---DDDDSDEDKEGSGD--GDWPSHVEFY--ASGVSWSSFHS------      279
tr|A0A7N4PT60|A0A7N4PT60_SARHA      HPPATTYGASSGED-ESDKSDEDKDGGSA--GSWPMGLEFY--ASGVKWSSFHS------      284
tr|A0A6P3YLF4|A0A6P3YLF4_SHEEP      YLAGAVCGASSG---DDDDSDEDREGSGD--GDWPSHVEFY--ASGVSWSSFHS------      279
tr|G5BL44|G5BL44_HETGA              HLGGAFYGASSGDDGDDDDSDEDKEGSRD--GDWPSRVQFY--ASGVSWSAFHS------      282
tr|A0A5K1U7A2|A0A5K1U7A2_CALJA      HLARAICGASSG---DDDDSDEDKEGSGD--GDWSSRVEFY--SSGNAFPFRKPQ-----      280
tr|A0A7J8CA60|A0A7J8CA60_MOLMO      SPARASCGASSGD--DDDDSDEDKEGSGD--GDWPGHVKFY--DSGVSWSSFHS------      275
sp|Q68FU1|PKHF1_RAT                 LGS-TMCGASSG---DDDDSDEDREGSGD--GDWPTQVEFY--ASGVSWSAFHS------      279
tr|A0A5J5MWL9|A0A5J5MWL9_MUNRE      YLAGAVCGASSG---DDDDSDEDREGSGD--ADWPSRVEFY--ASGVSWSSFHS------      279
tr|A0A5N3VFQ3|A0A5N3VFQ3_MUNMU      YLAGAVCGASSG---DDDDSDEDREGSGD--ADWPSRVEFY--ASGVSWSSFHS------      279
tr|A0A452DRQ5|A0A452DRQ5_CAPHI      YLAGAVCGASSG---DDDDSDEDREGSGD--GDWPSHVEFY--ASGVSWSSFHS------      279
tr|A0A6J0XTU7|A0A6J0XTU7_ODOVR      YLAGAVCGASSG---DDDDSDEDREGSGD--ADWPSRVEFY--ASGVSWSSFHS------      279
tr|A0A1S3AR32|A0A1S3AR32_ERIEU      ---AHLVGASSG---DDDDSDEDKEGGGD--GDWPGQVKFY--ASN-IWSSFHS------      272
tr|A0A7N4NQK3|A0A7N4NQK3_SARHA      HPPATTYGASSGED-ESDKSDEDKDGGSA--GSWPMGLEFY--ASGVKWSSFHS------      317
tr|A0A6P5K5Z3|A0A6P5K5Z3_PHACI      HSRGTTYGASSGED-ESDKSDEEKDGGSA--GSWPTGVEFY--ASGVKWSSFHS------      285
```

tr|A0A286XEI1|A0A286XEI1_CAVPO	HLGGALYGASSGDD----DSDEDKEGSRD--GDWPSRVQFY--ASSVSWSAFHS------	278
tr|A0A6P5K5R9|A0A6P5K5R9_PHACI	HSRGTTYGASSGED-ESDKSDEEKDGGSA--GSWPTGVEFY--ASGVKWSSFHS------	303
tr|A0A6J0XTS7|A0A6J0XTS7_ODOVR	YLAGAVCGASSG---DDDDSDEDREGSGD--ADWPSRVEFY--ASGVSWSSFHS------	320
tr|A0A485NDJ2|A0A485NDJ2_LYNPA	YLAGAICGASSG---DDDDSDEDKEGSGD--GDWPSHVEFY--ASGVSWSSFHS------	283
tr|A0A667J1S0|A0A667J1S0_LYNCA	YLAGAICGASSG---DDDDSDEDKEGSGD--GDWPSHVEFY--ASGVSWSSFHS------	283
tr|A0A4X2L5D8|A0A4X2L5D8_VOMUR	HSQGTTYGASSGED-ESDKSDEEKDGGSA--GSWPTGLEFY--ASGVKWSSFHS------	285
tr|A0A6P5K760|A0A6P5K760_PHACI	HSRGTTYGASSGED-ESDKSDEEKDGGSA--GSWPTGVEFY--ASGVKWSSFHS------	318
tr|M3WT21|M3WT21_FELCA	YLAGAICGASSG---DDDDSDEDKEGSGD--GDWPSHVEFY--ASGVSWSSFHS------	280
tr|A0A6P6H4E9|A0A6P6H4E9_PUMCO	YLAGAICGASSG---DDDDSDEDKEGSGD--GDWPSHVEFY--ASGVSWSSFHS------	281
tr|A0A6P4TXR5|A0A6P4TXR5_PANPR	YLAGAICGASSG---DDDDSDEDKEGSGD--GDWPSRVEFY--ASGVSWSSFHS------	281
tr|A0A2U3X5H7|A0A2U3X5H7_ODORO	-SAGAGCGASSG---DEEDSDEDKEGSGD--GDWPSRVEFY--ASGTSWSAFHS------	277
tr|A0A6G1B9W0|A0A6G1B9W0_CROCR	YLAGAVCGASSG---DDDDSDEDKEGSGD--GDWPSQVEFY--ASGVSWSSFHS------	280
tr|A0A6J0E1B0|A0A6J0E1B0_PERMB	LGG-AVCGASSG---DDDDSDEDREGSGD--GDWPSQVEFY--ASDVSWSAFHS------	279
tr|F7EZS4|F7EZS4_MONDO	HSPGTTYGVSSGED-ESDKSDEDKDGGSS--GSWPMGLEFY--ATGVKWSSFHS------	303
tr|A0A6J0DUG8|A0A6J0DUG8_PERMB	LGG-AVCGASSG---DDDDSDEDREGSGD--GDWPSQVEFY--ASDVSWSAFHS------	286
tr|A0A2Y9HYM5|A0A2Y9HYM5_NEOSC	-AAGAGCGASSG---DEEDSDEDKEGSGA--GDWPSRVEFY--ASGASWSAFHS------	277
tr|A0A2U3Y6L0|A0A2U3Y6L0_LEPWE	-AAGAGCGASSG---DEEDSDEDKEGSGA--GDWPSRVEFY--ASGASWSAFHS------	277
tr|A0A5E4BQ75|A0A5E4BQ75_MARMO	HLGGAFCGASSG---DDDDSDEDREGSRD--GDWPSHVEFY--TSGVSWSAFHS------	279
tr|I3N3T1|I3N3T1_ICTTR	HLGGAFCGASSG---DDDDSDEDREGSRD--GDWPSHVEFY--TSGVSWSAFHS------	279
tr|F7AL15|F7AL15_ORNAN	LR------LAVG--------------GTA--AYLNHALQ---------------------	243
tr|A0A6J0E1A5|A0A6J0E1A5_PERMB	LGG-AVCGASSG---DDDDSDEDREGSGD--GDWPSQVEFY--ASDVSWSAFHS------	296
tr|A0A3Q7MSJ7|A0A3Q7MSJ7_CALUR	-SAGAGCEASSG---DEEDSDEDKEGNGD--GDWPSSVEFY--ASGASWSAFHS------	277
tr|A0A6J2F2G5|A0A6J2F2G5_ZALCA	-SAGAGCGASSG---DEEDSDEDKEGNGD--GDWPSGVEFY--ASGASWSAFHS------	277
tr|A0A6P6EYH5|A0A6P6EYH5_OCTDE	HLGAAFYGASSGDD----DSDEDKEGSRD--GDWPSHVQFY--ASSVSWSAFHS------	278
sp|Q3TB82|PKHF1_MOUSE	LGG-TVCGASSG---DDDDSDEDREGNGD--GDWPTQVEFY--ASGVSWSAFHS------	279
tr|A0A061HTX9|A0A061HTX9_CRIGR	LGGGTVYGASSG---DDDDSDDDREGSGD--GDWPSQVEFY--TSGVSWSAFHS------	280
tr|A0A671FB93|A0A671FB93_RHIFE	AGSRALCGASSGD--EDEDSDEDREGSGD--GDWPSRVQFY--SSGVSWSSFHS------	274
tr|A0A341AK17|A0A341AK17_NEOAA	YLAGAVCGASSG---DDDDSDEDRESSGD--GDWPSRVEFY--ASGVTWSSFHS------	279
tr|A0A340YG10|A0A340YG10_LIPVE	YLAGAVCGASSG---DDDDSDEDREGSGD--GDWPSRVEFY--ASGVSWSSFHS------	279
tr|A0A5N4DR82|A0A5N4DR82_CAMDR	YLAGAVCGASSG---EDDDSDEDKEGSGD--GDWPSRVEFY--ASGVSWSSFHS------	279
tr|A0A4U1EUS3|A0A4U1EUS3_MONMO	YLAGAVCGASSG---DDDDSDEDREGSGD--GDWPSRVEFY--ASGVTWSSFHS------	279
tr|A0A2Y9P694|A0A2Y9P694_DELLE	YLAGAVCGASSG---DDDDSDEDREGSGD--GDWPSRVEFY--ASGVTWSSFHS------	279
tr|A0A2U3V2U0|A0A2U3V2U0_TURTR	YVAGAVCGASSG---DDDDSDEDREGSGD--GDWPSRVEFY--ASGVSWSSFHS------	279
tr|A0A3Q7TL14|A0A3Q7TL14_VULVU	YLGGAECGASSG---DDEDSDEDKEGGGD--GDWPSRVEFY--ASGVSWSAFHS------	283
tr|G1ML40|G1ML40_AILME	--	224
tr|A0A6P7R571|A0A6P7R571_MUSCR	LGG-TVCGASSG---DDDDSDEDREGNGD--GDWPTQVEFY--ASGVSWSAFHS------	279
tr|A0A3P4S7D1|A0A3P4S7D1_GULGU	YLPGAGAGASSG---DEEDSDEDKEGSGD--GDWPSHVEFY--ASGVSWSAFHS------	281
tr|A0A6P3V9N9|A0A6P3V9N9_OCTDE	HLGAAFYGASSGDD----DXDEDKEGSRD--GDWPSHVQFY--ASSVSWSAFHS------	278
tr|M3Z5G8|M3Z5G8_MUSPF	YLSGAGTGASSG---DEEDSDEDKEGSGD--SNWSSRVEFY--ASGVSWSAFHS------	285
tr|D2H395|D2H395_AILME	YLAGAVCGASS---	243
tr|A0A383YQU7|A0A383YQU7_BALAS	YLAGAVCGASSG---DDDDSDEDREGSGD--GDWPSRVEFY--ASGVSWSSFHS------	279
tr|A0A2Y9F4I2|A0A2Y9F4I2_PHYMC	YLAGAVCGASSG---DDDDSDEDREGSGD--GDWPSRVEFY--ASGVSWSSFHS------	279
tr|A0A2Y9LBN9|A0A2Y9LBN9_ENHLU	YLSGAGAGASSG---DEEDSDEDKEGSGD--GDWPSRVEFY--ASGVSWSAFHS------	281
tr|A0A3Q7UUY9|A0A3Q7UUY9_URSAR	YLTGAVCGASSG---DDEDSDEDREGSGD--GDWPSRVEFY--SSGVSWSAFHS------	279
tr|A0A2F0BD53|A0A2F0BD53_ESCRO	YLAGAVCGASSG---DDDDSDEDREGSGD--GDWPSRVEFY--ASGVSWSSFHS------	298

tr|G3UCB5|G3UCB5_LOXAF	YLAGAVLVASSG---DDDDSDEDKEGDGD--GDWPSRVEFY--ASGVSWSSFHS------	279
tr|A0A2Y9QH78|A0A2Y9QH78_TRIMA	YLAGAVFGASSG---DDDDSDEDKEGSGD--GDWPSRVEFY--ASGASWSSFHS------	279
tr|A0A250YE73|A0A250YE73_CASCN	HLGGPGGGTSSC---DDDDSDEDR----D--VSWPSRVEFY--TSGVSWSSFHS------	275
tr|A0A6I9K0J7|A0A6I9K0J7_CHRAS	HFAGA---ASSG---DDEDSDEDREGSGD--GDWSSRVHFY--TSNVSWSSFHS------	276
tr|A0A452QT72|A0A452QT72_URSAM	YLAGAVCGASSG---DDEDSDEDREGSGD--GDWPSRVEFY--SSGVSWSAFHS------	279
tr|A0A4X1TW95|A0A4X1TW95_PIG	YLAGAVCGASSG---DDDDSDEDREG-GD--GDWPSRVEFY--AGGVSWSSFHS------	283
tr|F1RNZ0|F1RNZ0_PIG	YLAGAVCGASSG---DDDDSDEDREG-GD--GDWPSRVEFY--AGGVSWSSFHS------	283
tr|A0A484GVY7|A0A484GVY7_SOUCH	YLAGAVCGASSG---DDDDSDEDREGSGD--GDWPSRVEFY--ASGVSWSSFHS------	348
tr|A0A7J8CKE8|A0A7J8CKE8_ROUAE	-PAGAGSGASSG-E-DEDDSEDDKEGSGD--SDWPSRVKFY--AAGVSWSSFHS------	275
tr|A0A5G2RAS5|A0A5G2RAS5_PIG	YLAGAVCGASSG---DDDDSDEDREG-GD--GDWPSRVEFY--AGGVSWSSFHS------	294
tr|A0A1A6G1C6|A0A1A6G1C6_NEOLE	LGG-AVCGASSG---DDDDSDEDREGSGD--GDWPSQVEFY--ASGVSWSAFHS------	297
tr|A0A6J2L2L8|A0A6J2L2L8_9CHIR	SPARAGCGASSGD--D-EDSDDDREGSGD--GDWPSRVRFY--DSGVSWSSFHS------	277
tr|A0A3Q2LSN2|A0A3Q2LSN2_HORSE	CLAGAICGASSG---DDYDSDEDKEGSGD--GEWPSHVEFY--TSGVSWSSFHS------	330
tr|E2R1B7|E2R1B7_CANLF	YLGGAECGASSG---DDEDSDEDKEGGGD--GDWPSHVEFY--ASGVSWSAFHG------	465
tr|K9IX34|K9IX34_DESRO	SLAGAGCGASSGD----EDSDEDREGSAD--GDWPSRVRFY--DSGVSWSAFHS------	279
tr|A0A7J7SDK0|A0A7J7SDK0_MYOMY	SEAGAGCGVSSGDE-DDDDSEEDKEGGGE--GDWPSRIRFY--DSGVSWSSFHS------	284
tr|A0A6P3IET2|A0A6P3IET2_BISBI	HLAGAVCGASSG---DDDDSDEDKEGSGD--GDWPSHVEFY--ASGVSWSSFHS------	265
tr|G3HI22|G3HI22_CRIGR	-----------------NDVSDDDD------DDDSS----------D-------------	122
tr|G5C4B3|G5C4B3_HETGA	-----------------NNVSDDED------DDSSD------------------------	127
tr|W5P1M9|W5P1M9_SHEEP	YLAGAVCGASSG---DDDDSDEDREGSGD--GDWPSHVEFY--ASGVSWSSFHS------	225
tr|S9WTG5|S9WTG5_CAMFR	YLAGAVCGASSGED---DDSDEDKEGSGD--GDWPSRVEFY--ASGVSWSSFHS------	303
tr|A0A6J0AU76|A0A6J0AU76_VICPA	YLAGAVCGASSG---EDDDSDEDKEGSGD--GDWPSRVEFY--ASGVSWSSFHS------	246
tr|L5KVX2|L5KVX2_PTEAL	-PAGAGSGASSG-E-DEDDSEEDKEGSGD--GDWPSRVKFY--GEGVSWSSFHS------	302
tr|A0A2J8KSN2|A0A2J8KSN2_PANTR	--	128
tr|A0A2J8SAE9|A0A2J8SAE9_PONAB	--	128
tr|K7ELB8|K7ELB8_HUMAN	--	128
tr|L8Y3Z0|L8Y3Z0_TUPCH	HLTGAVCGASSG---DDDDSDEDKEGYGD--GDWPSHVEFY--ASGVSWSAFHR------	640
tr|A0A2J8KSM7|A0A2J8KSM7_PANTR	--	82
tr|A0A2J8SAE1|A0A2J8SAE1_PONAB	--	82
tr|K7EIX0|K7EIX0_HUMAN	--	82
tr|A0A2J8KVM1|A0A2J8KVM1_PANTR	--	279
tr|A0A2J8XN15|A0A2J8XN15_PONAB	--	279
tr|F8VQX5|F8VQX5_HUMAN	--	279
tr|A0A4X2MDN9|A0A4X2MDN9_VOMUR	-LSSVLHSIPSGRKQKKIP-AALKEVSANTEDSTMSGYLYRSKGSKKPWKHFWFVIKNKV	1342
tr|A0A4X2M634|A0A4X2M634_VOMUR	-LSSVLHSIPSGRKQKKIP-AALKEVSANTEDSTMSGYLYRSKGSKKPWKHFWFVIKNKV	1375
tr|F6W6U6|F6W6U6_MONDO	-LSSVLHSIPSGRKQKKIP-AALKEVSANTEDSTMSGYLYRSKGSKKPWKHFWFVIKNKV	1369

Humans

sp|Q9H8W4|PKHF2_HUMAN	-DDDDD---------DSSD---	249
sp|Q96S99|PKHF1_HUMAN	-GDDDD---------SDEDKEG--------SRDGDWPSSVE------------------F	267
tr|B4DWN9|B4DWN9_HUMAN	-GDDDD---------SDEDKEG--------SRDGDWPSSVE------------------F	352
tr|K7ELB8|K7ELB8_HUMAN	--	128
tr|K7EIX0|K7EIX0_HUMAN	--	82

```
tr|F8VQX5|F8VQX5_HUMAN              ------------------------------------------------------------      279
tr|A4FVC4|A4FVC4_HUMAN              -PSSALSSVLH----SIP--SG--------RKQKKIPAALKEVSANT---EDSS-MSGYL      549
sp|Q6ZV73|FGD6_HUMAN                -PSSALSSVLH----SIP--SG--------RKQKKIPAALKEVSANT---EDSS-MSGYL      1340
tr|A0A2X0SFJ2|A0A2X0SFJ2_HUMAN      -PSSALSSVLH----SIP--SG--------RKQKKIPAALKEVSANT---EDST-MSGYL      1308
tr|Q49A55|Q49A55_HUMAN              GILE------------------------------------IESAEVSGNSVVCS-FLQ--      403
tr|F8W1R0|F8W1R0_HUMAN              GILE------------------------------------IESAEVSGNSVVCS-FLQ--      558
sp|Q96M96|FGD4_HUMAN                GILE------------------------------------IESAEVSGNSVVCS-FLQ--      651
tr|A0A2X0U2J9|A0A2X0U2J9_HUMAN      GILE------------------------------------IESAEVSGNSVVCS-FLQ--      651
tr|B7Z493|B7Z493_HUMAN              GILE------------------------------------IESAEVSGNSVVCS-FLQ--      736
tr|B7Z8F9|B7Z8F9_HUMAN              GILE------------------------------------IESAEVSGNSVVCS-FLQ--      763
tr|E9PJX4|E9PJX4_HUMAN              GILE------------------------------------IESAEVSGNSVVCS-FLQ--      763
tr|F8VWL3|F8VWL3_HUMAN              GILE------------------------------------IESAEVSGNSVVCS-FLQ--      788
sp|Q7Z6J4|FGD2_HUMAN                GILE------------------------------------KGSSATPDQSLMCS-FLQ--      552
tr|A8K304|A8K304_HUMAN              GILE------------------------------------KGSSATPDQSLMCS-FLQ--      552
tr|A0A384NQ05|A0A384NQ05_HUMAN      GILE------------------------------------KGSSATPDQSLMCS-FLQ--      552
tr|A0A2X0SYZ7|A0A2X0SYZ7_HUMAN      GILE------------------------------------KGSSATPDQSLMCS-FLQ--      552
sp|P98174|FGD1_HUMAN                SILE------------------------------------KQASVAAENSVICS-FLH--      829
tr|A0A024R9Y5|A0A024R9Y5_HUMAN      SILE------------------------------------KQASVAAENSVICS-FLH--      829
tr|A0A2X0SFE1|A0A2X0SFE1_HUMAN      SILE------------------------------------KQASVAAENSVICS-FLH--      829
tr|G3V2D8|G3V2D8_HUMAN              -ESPPYSFVVR-----V---PK--------ADEVEWILDLKEEE------NELV-RSEFY      1927
tr|A0A7I2YQV0|A0A7I2YQV0_HUMAN      -ESPPYSFVVR-----V---PK--------ADEVEWILDLKEEE------NELV-RSEFY      1901
sp|Q68DK2|ZFY26_HUMAN               -ESPPYSFVVR-----V---PK--------ADEVEWILDLKEEE------NELV-RSEFY      1927
tr|A0A7I2YQU3|A0A7I2YQU3_HUMAN      -ESPPYSFVVR-----V---PK--------ADEVEWILDLKEEE------NELV-RSEFY      1933
tr|A0A7I2V403|A0A7I2V403_HUMAN      -ESPPYSFVVR-----V---PK--------ADEVEWILDLKEEE------NELV-RSEFY      1942
tr|B7ZM68|B7ZM68_HUMAN              -SGSAFSSVFQ----SINPSTF--------KKQKKVPSALT-------------------      1114
tr|A3KMQ0|A3KMQ0_HUMAN              -SGSAFSSVFQ----SINPSTF--------KKQKKVPSALTEVAASG---EGSA-ISGYL      1129
sp|Q6ZNL6|FGD5_HUMAN                -SGSAFSSVFQ----SINPSTF--------KKQKKVPSALTEVAASG---EGSA-ISGYL      1370
tr|A0A2X0SFF2|A0A2X0SFF2_HUMAN      -SGSAFSSVFQ----SINPSTF--------KKQKKVPSALTEVAASG---EGSA-ISGYL      1370
sp|Q8WXA3|RUFY2_HUMAN               ------------------------------------------------------------      606
tr|H0YD93|H0YD93_HUMAN              ------------------------------------------------------------      620
tr|B3KSL1|B3KSL1_HUMAN              ------------------------------------------------------------      310
tr|A8K7B1|A8K7B1_HUMAN              ------------------------------------------------------------      600
sp|Q96T51|RUFY1_HUMAN               ------------------------------------------------------------      708
tr|Q59GI4|Q59GI4_HUMAN              ------------------------------------------------------------      1333
sp|O95405|ZFYV9_HUMAN               ------------------------------------------------------------      1425
sp|Q7Z3T8|ZFY16_HUMAN               ------------------------------------------------------------      1539
tr|A0A024RAL3|A0A024RAL3_HUMAN      ------------------------------------------------------------      1539
tr|B4DVN4|B4DVN4_HUMAN              GILE------------------------------------KGSSATPDQSLMCS-FLQ--      180
sp|Q15075|EEA1_HUMAN                ------------------------------------------------------------      1411
tr|Q49AA1|Q49AA1_HUMAN              ------------------------------------------------------------      192
tr|F8VY01|F8VY01_HUMAN              ------------------------------------------------------------      1206
sp|Q9HCC9|LST2_HUMAN                ------------------------------------------------------------      887
sp|Q9Y4F1|FARP1_HUMAN               -------------------------------------------------AVENQ-LSGNL      939
tr|A0A2X0TVY0|A0A2X0TVY0_HUMAN      -------------------------------------------------AVENQ-LSGNL      939
tr|B4DS99|B4DS99_HUMAN              ------------------------------------------------------------      525
```

```
tr|G5E953|G5E953_HUMAN              -PIAATSN----------------------------------------------------     1062
sp|Q13615|MTMR3_HUMAN               -PIAATSN----------------------------------------------------     1198
tr|A8K0L6|A8K0L6_HUMAN              -PIAATSN----------------------------------------------------     1198
tr|A7E2W2|A7E2W2_HUMAN              -PIAATSN----------------------------------------------------     1198
sp|Q5JSP0|FGD3_HUMAN                ---------------------------------------------DPQPSLLCG-PLR--     612
tr|A0A024R252|A0A024R252_HUMAN      ---------------------------------------------DPQPSLLCG-PLR--     612
tr|A0A2X0U4Q9|A0A2X0U4Q9_HUMAN      ---------------------------------------------DPQPSLLCG-PLR--     612
tr|Q9H8N9|Q9H8N9_HUMAN              -G-----------------LMVTCGTD---RIVKIWDMTP---------VVGC------S     359
sp|Q8IWB7|WDFY1_HUMAN               -G-----------------LMVTCGTD---RIVKIWDMTP---------VVGC------S     402
tr|A0A024R488|A0A024R488_HUMAN      -G-----------------LMVTCGTD---RIVKIWDMTP---------VVGC------S     402
tr|A0A7I2YQD1|A0A7I2YQD1_HUMAN      -----QSQLPPKRD-ETA-----------LQEEEELQLALALSQSEAEEKERLRQKSTYT     264
tr|I3L1P5|I3L1P5_HUMAN              -----QSQLPPKRD-ETA-----------LQEEEELQLALALSQSEAEEKERLRQKSTYT     287
tr|B4E1E2|B4E1E2_HUMAN              -----QSQLPPKRD-ETA-----------LQEEEELQLALALSQSEAEEKERLRQKSTYT     287
tr|A0A7I2V3Z1|A0A7I2V3Z1_HUMAN      -----QSQLPPKRD-ETA-----------LQEEEELQLALALSQSEAEEKERLRQKSTYT     220
tr|Q9NXY1|Q9NXY1_HUMAN              SCSP------------------------------------PGGAAEPPDTCSCA-PAA--     685
tr|A0A0S2Z4Q4|A0A0S2Z4Q4_HUMAN      -----QSQ----------------------------------------------------     247
tr|A0A7I2V6E0|A0A7I2V6E0_HUMAN      -----QSQLPPKRD-ETA-----------LQEEEELQLALALSQSEAEEKERLRQKSTYT     220
tr|A8K9G1|A8K9G1_HUMAN              ---------------------------------------------DPQPSLLCG-PLR--     611
tr|A0A7I2V2K2|A0A7I2V2K2_HUMAN      -----QSQLPPKRD-ETA-----------LQEEEELQLALALSQSEAEEKERLRQKSTYT     287
tr|A0A7I2V5A2|A0A7I2V5A2_HUMAN      -----QSQLPPKRD-ETA-----------LQEEEELQLALALSQSEAEEKERLRQKSTYT     287
tr|A0A7I2V3I8|A0A7I2V3I8_HUMAN      -----QSQLPPKRD-ETA-----------LQEEEELQLALALSQSEAEEKERLRQKSTYT     271
tr|A0A7I2V3F9|A0A7I2V3F9_HUMAN      -----QSQLPPKRD-ETA-----------LQEEEELQLALALSQSEAEEKERLRQKSTYT     287
tr|A0A7I2V2D8|A0A7I2V2D8_HUMAN      -----QSQLPPKRD-ETA-----------LQEEEELQLALALSQSEAEEKERLRQKSTYT     287
tr|A0A7I2V2W5|A0A7I2V2W5_HUMAN      -----QSQLPPKRD-ETA-----------LQEEEELQLALALSQSEAEEKERLRQKSTYT     278
tr|A0A7I2V2R9|A0A7I2V2R9_HUMAN      -----QSQLPPKRD-ETA-----------LQEEEELQLALALSQSEAEEKERLRQKSTYT     287
sp|O14964|HGS_HUMAN                 -----QSQLPPKRD-ETA-----------LQEEEELQLALALSQSEAEEKERLRQKSTYT     287
tr|A0A7I2V3J3|A0A7I2V3J3_HUMAN      -----QSQLPPKRD-ETA-----------LQEEEELQLALALSQSEAEEKERLRQKSTYT     287
tr|A0A0S2Z4R4|A0A0S2Z4R4_HUMAN      -----QSQLPPKRD-ETA-----------LQEEEELQLALALSQSEAEEKERLRQKSTYT     287
tr|A0A7I2V637|A0A7I2V637_HUMAN      -----QSQLPPKRD-ETA-----------LQEEEELQLALALSQSEAEEKERLRQKSTYT     305
tr|A0A7I2V5A3|A0A7I2V5A3_HUMAN      -----QSQLPPKRD-ETA-----------LQEEEELQLALALSQSEAEEKERLRQKSTYT     287
tr|B4DFP5|B4DFP5_HUMAN              ------------------------------------------------------------     221
sp|O94887|FARP2_HUMAN               -------------------------------------------------AVENQ-LSGYL     936
tr|A0A2X0SSL3|A0A2X0SSL3_HUMAN      -------------------------------------------------AVENQ-LSGYL     936
tr|J3QR65|J3QR65_HUMAN              -PIATASS----------------------------------------------------     1138
sp|Q9NYA4|MTMR4_HUMAN               -PIATASS----------------------------------------------------     1195
tr|Q5H9P4|Q5H9P4_HUMAN              ------------------------------------------------------------     924
sp|Q9P2R3|ANFY1_HUMAN               ------------------------------------------------------------     1169
tr|A0A7I2V307|A0A7I2V307_HUMAN      -----QSQLPPKRD-ETA-----------LQEEEELQLALALSQSEAEEKERLRQKSTYT     293
tr|A0A024R1I2|A0A024R1I2_HUMAN      -PIAATSN----------------------------------------------------     1161
sp|Q9HBF4|ZFYV1_HUMAN               ------------------------------------------------------------     777
tr|G3V5N8|G3V5N8_HUMAN              ------------------------------------------------------------     789
tr|C9JME2|C9JME2_HUMAN              -------------------------------------------------AVENQ-LSGNL     970
tr|C9JL08|C9JL08_HUMAN              ------------------------------------------------------------     241
sp|Q9BQ24|ZFY21_HUMAN               ------------------------------------------------------------     234
tr|E9PDH4|E9PDH4_HUMAN              -----IF-----------I-----------KRQAPLKVSLLQDLKDFFQK----------     1363
```

```
sp|Q9Y2I7|FYV1_HUMAN                 -----IF-----------I-----------KRQAPLKVSLLQDLKDFFQK----------      1419
tr|V9HWG4|V9HWG4_HUMAN               -----IF-----------I-----------KRQAPLKVSLLQDLKDFFQK----------      1419
tr|A0A7I2V5X2|A0A7I2V5X2_HUMAN       -----HSQRRRRRR-G--------------------------------------------      74
tr|A7E1Z6|A7E1Z6_HUMAN               ------------------------------------------------------------      281
tr|Q96LK4|Q96LK4_HUMAN               -G-----------------WLLTSRTD---KVIKLWDMTP---------VVS--------      297
sp|Q9BQS8|FYCO1_HUMAN                GPQAT-----G----G-----QGANTDYRPPDDAVFDIIT--------DEELCQ-IQESG      1295
sp|Q96P53|WDFY2_HUMAN                -G-----------------WLLTSGTD---KVIKLWDMTP---------VVS--------      400
tr|A7E293|A7E293_HUMAN               ------------------------------------------------------------      1563
sp|Q8IZQ1|WDFY3_HUMAN                ------------------------------------------------------------      3526
tr|A0A024RDC2|A0A024RDC2_HUMAN       ------------------------------------------------------------      3526
tr|B4DXH4|B4DXH4_HUMAN               ---------------------------------------------DPQPSLLCG-PLR--      215
tr|A0A7I2V5N3|A0A7I2V5N3_HUMAN       ------------------------------------------------------------      64
tr|A0A7I2V5W6|A0A7I2V5W6_HUMAN       ------------------------------------------------------------      64
tr|Q96P52|Q96P52_HUMAN               ------------------------------------------------------------      420
tr|B3KPJ0|B3KPJ0_HUMAN               ------------------------------EFSDDWTLSGKDNSGLIEQVGKCS-LLPYF      564
tr|H0Y696|H0Y696_HUMAN               ------------------------------EFSDDWTLSGKDNSGLIEQVGKCS-LLPYF      573
tr|A8K4R4|A8K4R4_HUMAN               ------------------------------------------------------------      1104
tr|Q6IQ41|Q6IQ41_HUMAN               ------------------------------EFSDDWTLSGKVGN----------------      1237
sp|Q96Q42|ALS2_HUMAN                 ------------------------------EFSDDWTLSGKGTLTMPNGD---Y-IEGYF      1249
tr|A0A2X0SFA0|A0A2X0SFA0_HUMAN       ------------------------------EFSDDWTLSGKGTLTMPNGD---Y-IEGYF      1249
sp|Q9H1K0|RBNS5_HUMAN                EYIRMAASLNA----GETTYSLEHASDLRVEVQKVYELID---------ALSKK-ILTLG      340
tr|A8K5H3|A8K5H3_HUMAN               EYIRMAASLNA----GETTYSLEHASDLRVEVQKVYELID---------ALSKK-ILTLG      340
tr|H3BRF9|H3BRF9_HUMAN               --------QSQ----GLT---------------RQDQMIA--------ERLARL-RQE--      164
sp|Q8TCU6|PREX1_HUMAN                GCLEHLFNQVD----SINALLKGPVMSRAFEETKHFPMN--HSLQEFKQKEECT-IRGRS      1272
tr|A0A2X0SFH1|A0A2X0SFH1_HUMAN       GCLEHLFNQVD----SINALLKGPVMSRAFEETKHFPMN--HSLQEFKQKEECT-IRGRS      1272
tr|M0QXT1|M0QXT1_HUMAN               ------------------------------------------------------------      248
tr|B3KPZ0|B3KPZ0_HUMAN               ------------------------------------------------------------      1093
tr|H3BRM1|H3BRM1_HUMAN               --------QSQ----GLT---------------RQDQMIA--------ERLARL-RQE--      158
tr|E9PJM7|E9PJM7_HUMAN               ------------------------------------------------------------      226
sp|Q96K21|ANCHR_HUMAN                --------QSQ----GLT---------------RQDQMIA--------ERLARL-RQE--      187
tr|B4E245|B4E245_HUMAN               ------------------------------------------------------------      516
tr|E7EMB1|E7EMB1_HUMAN               ------------------------------------------------------------      527
tr|B3KUB9|B3KUB9_HUMAN               ------------------------------------------------------------      527
sp|Q9UH65|SWP70_HUMAN                ------------------------------------------------------------      585
sp|Q70Z35|PREX2_HUMAN                SCLEHLFSQVD----SITNLLKGQAVVRAFDQTKYLTPG--RGLQEFQQEMEPK-LSCPK      1212
tr|A0A2X0SZ31|A0A2X0SZ31_HUMAN       SCLEHLFSQVD----SITNLLKGQAVVRAFDQTKYLTPG--RGLQEFQQEMEPK-LSCPK      1212
tr|H3BN64|H3BN64_HUMAN               --------QSQ----GLT---------------RQDQMIA--------ERLARL-RQE--      115
tr|H7C3C4|H7C3C4_HUMAN               -EQPKFLGIREQRVTGLMIFILMGLSVFMTSVLKFIPMPVLYGVFLYMGV--SS-LKGIQ      604
tr|C9JRP1|C9JRP1_HUMAN               -EQPKFLGIREQRVTGLMIFILMGLSVFMTSVLKFIPMPVLYGVFLYMGV--SS-LKGIQ      949
tr|A0A0A0MST8|A0A0A0MST8_HUMAN       -EQPKFLGIREQRVTGLMIFILMGLSVFMTSVLKFIPMPVLYGVFLYMGV--SS-LKGIQ      1038
tr|B5M450|B5M450_HUMAN               -EQPKFLGIREQRVTGLMIFILMGLSVFMTSVLKFIPMPVLYGVFLYMGV--SS-LKGIQ      1049
tr|E9PFN4|E9PFN4_HUMAN               -EQPKFLGIREQRVTGLMIFILMGLSVFMTSVLKFIPMPVLYGVFLYMGV--SS-LKGIQ      1049
sp|Q9Y6M7|S4A7_HUMAN                 -EQPKFLGIREQRVTGLMIFILMGLSVFMTSVLKFIPMPVLYGVFLYMGV--SS-LKGIQ      1053
tr|Q5JVY0|Q5JVY0_HUMAN               ------------------------------------------------------------      172
tr|E7EW28|E7EW28_HUMAN               -EQPKFLGIREQRVTGLMIFILMGSSVFMTSILKFIPMPVLYGVFLYMGA--SS-LKGIQ      954
```

```
tr|C9J240|C9J240_HUMAN              -EQPKFLGIREQRVTGLMIFILMGSSVFMTSILKFIPMPVLYGVFLYMGA--SS-LKGIQ      924
sp|Q6U841|S4A10_HUMAN               -EQPKFLGIREQRVTGLMIFILMGSSVFMTSILKFIPMPVLYGVFLYMGA--SS-LKGIQ      954
sp|Q13049|TRI32_HUMAN               ------------------------------------------------------------      653
tr|A0A024R843|A0A024R843_HUMAN      ------------------------------------------------------------      653
sp|P52735|VAV2_HUMAN                ------------------------------------------------------------      878
tr|B4DFS9|B4DFS9_HUMAN              ------------------------------------------------------------      328
tr|H0Y724|H0Y724_HUMAN              ------------------------------------------------------------      0
tr|B4E2V8|B4E2V8_HUMAN              ----RFVSMNR----GINN-------------GSDLPEDQLRNLFDSIK-----------      182
sp|Q9UIA0|CYH4_HUMAN                ----RFVSMNR----GINN-------------GSDLPEDQLRNLFDSIK-----------      239
tr|B2RCD2|B2RCD2_HUMAN              ----RFVSMNR----GINN-------------GSDLPEDQLRNLFDSIK-----------      239
tr|A7E2A5|A7E2A5_HUMAN              ---------------------------ATLDSDHKLPSRVIEELNVVLQRSRTL-PKELQ      1628
sp|Q8WZ64|ARAP2_HUMAN               ---------------------------ATLDSDHKLPSRVIEELNVVLQRSRTL-PKELQ      1698
tr|A0A2X0SFS3|A0A2X0SFS3_HUMAN      ---------------------------ATLDSDHKLPSRVIEELNVVLQRSRTL-PKELQ      1698
tr|B4DZ04|B4DZ04_HUMAN              ------------------------------------------------------------      415
tr|B4E1Y1|B4E1Y1_HUMAN              ------------------------------------------------------------      415
tr|Q5SQI7|Q5SQI7_HUMAN              ------------------------------------------------------------      542
tr|A0A2X0SFV4|A0A2X0SFV4_HUMAN      ------------------------------------------------------------      544
sp|Q7Z628|ARHG8_HUMAN               ------------------------------------------------------------      596
tr|Q5SQI5|Q5SQI5_HUMAN              ------------------------------------------------------------      596
tr|B4DS29|B4DS29_HUMAN              ------------------------------------------------------------      677
sp|Q2Y0W8|S4A8_HUMAN                -EQPKFLGIREQRVTGLMIFVLMGCSVFMTAILKFIPMPVLYGVFLYMGV--SS-LQGIQ      923
tr|Q9NUL6|Q9NUL6_HUMAN              ----RFVSMNR----GINN-------------GSDLPEDQLRNLFDSIK-----------      46
tr|A0A0D9SFG6|A0A0D9SFG6_HUMAN      ----RFVAMNR----GINE-------------GGDLPEELLRNLYDSIR-----------      239
sp|Q6ZNE9|RUFY4_HUMAN               ------------------------------------------------------------      571
tr|B2RBP7|B2RBP7_HUMAN              ------------------------------------------------------------      631
sp|Q8IVQ6|ZDH21_HUMAN               -RLAAFMG--------ITMLVG---------ITGLFYTQLIGIITD-----TTS-IE---      213
tr|Q15795|Q15795_HUMAN              ----RFVAMNR----GINE-------------GGDLPEELLRNLYDSIR-----------      105
tr|C9JKV5|C9JKV5_HUMAN              ------------------------------------------------------------      131
sp|Q9P2F6|RHG20_HUMAN               ---PHASGVPE------ANSLQ--------EEQKDLPLRAAEGLSPVQSAQRCS-SSPFQ      1116
tr|A0A2X0U2C5|A0A2X0U2C5_HUMAN      ---PHASGVPE------ANSLQ--------EEQKDLPLRAAEGLSPVQSAQRCS-SSPFQ      1116
tr|E9PNM3|E9PNM3_HUMAN              RPILQA-GLPA----N-KTVALGSNVEFM-----------------------CK-VYS--      281
```
