# Supplementary material for: The PH Domain and C-Terminal polyD Motif of Phafin2 Exhibit a Unique Concurrence in Animals
Source: Membranes (Basel). 2022 Jul 7;12(7):696. doi: 10.3390/membranes12070696 (PMC9324892; doi:10.3390/membranes12070696)
Supplement: Supplementary file 1 [file membranes-12-00696-s001.zip › Supplementary File S5.pdf]

Supplementary File S5: BLAST results and Multiple sequence alignment of human Phafin2 with each of top hit protozoan sequence search.

BLAST result

Description	*Scientific Name*	*Taxid*	*Max Score*	*Total Score*	*Query Cover*	*E value*	*Per. Ident*	*Acc. Len*	*Accession*
Rho guanine nucleotide exchange factor, putative [Entamoeba histolytica HM-1:IMSS]	Entamoeba histolytica HM-1:IMSS	294381	140	140	71%	1e-38	43.09%	455	XP_655361.1
Rho guanine nucleotide exchange factor, putative [Entamoeba histolytica HM-1:IMSS]	Entamoeba histolytica HM-1:IMSS	294381	129	129	71%	6e-34	38.46%	555	XP_649382.1
Rho guanine nucleotide exchange factor, putative [Entamoeba histolytica HM-1:IMSS]	Entamoeba histolytica HM-1:IMSS	294381	124	124	89%	1e-32	36.13%	441	XP_651719.1
Rho guanine nucleotide exchange factor, putative [Entamoeba histolytica HM-1:IMSS]	Entamoeba histolytica HM-1:IMSS	294381	124	124	97%	7e-32	32.16%	631	XP_651964.1
Rho guanine nucleotide exchange factor, putative [Entamoeba histolytica HM-1:IMSS-B]	Entamoeba histolytica HM-1:IMSS-B	885319	107	107	79%	2e-26	34.31%	460	EMH78141.1
Rho guanine nucleotide exchange factor, putative [Entamoeba histolytica HM-1:IMSS]	Entamoeba histolytica HM-1:IMSS	294381	107	107	79%	4e-26	34.31%	492	XP_655384.1
Rho guanine nucleotide exchange factor, putative [Entamoeba histolytica HM-1:IMSS]	Entamoeba histolytica HM-1:IMSS	294381	105	105	80%	1e-25	35.05%	495	XP_656365.1
Rho/RAC guanine nucleotide exchange factor, putative [Entamoeba histolytica HM-3:IMSS]	Entamoeba histolytica HM-3:IMSS	885315	104	104	79%	5e-25	33.99%	876	EMS15045.1
Rho guanine nucleotide exchange factor, putative [Entamoeba histolytica HM-1:IMSS]	Entamoeba histolytica HM-1:IMSS	294381	104	104	79%	5e-25	33.99%	876	XP_651436.1
rho/RAC guanine nucleotide exchange factor, putative [Entamoeba histolytica KU27]	Entamoeba histolytica KU27	885311	104	104	79%	5e-25	33.99%	745	EMD43911.1
Rho guanine nucleotide exchange factor, putative [Entamoeba histolytica HM-1:IMSS]	Entamoeba histolytica HM-1:IMSS	294381	103	103	81%	1e-24	32.21%	730	XP_656765.1
Rho guanine nucleotide exchange factor, putative [Entamoeba histolytica HM-1:IMSS]	Entamoeba histolytica HM-1:IMSS	294381	101	101	88%	4e-24	29.61%	696	XP_654401.1
Rho guanine nucleotide exchange factor, putative [Entamoeba histolytica HM-1:IMSS]	Entamoeba histolytica HM-1:IMSS	294381	85.9	85.9	70%	7e-19	31.49%	452	XP_651674.1
Rho guanine nucleotide exchange factor, putative [Entamoeba histolytica HM-1:IMSS]	Entamoeba histolytica HM-1:IMSS	294381	79.0	79.0	71%	3e-16	30.05%	725	XP_651711.2
Rho guanine nucleotide exchange factor, putative [Entamoeba histolytica HM-3:IMSS]	Entamoeba histolytica HM-3:IMSS	885315	77.4	77.4	38%	7e-16	41.67%	487	EMS16071.1
High-resolution solution structure of the LM5-1 FYVE domain from Leishmania major [Leishmania major]	Leishmania major	5664	64.3	64.3	32%	3e-13	39.51%	84	1Z2Q_A
conserved hypothetical protein [Leishmania major strain Friedlin]	Leishmania major strain Friedlin	347515	56.2	56.2	26%	9e-09	42.65%	377	XP_003722823.1

FYVE_zinc_finger_containing_protein_putative [Leishmania major strain Friedlin]	*Leishmania major* strain Friedlin	347515	51.2	51.2	28%	1e-07	36.99%	172	CAG9576238.1
conserved hypothetical protein [Leishmania major strain Friedlin]	*Leishmania major* strain Friedlin	347515	49.3	49.3	28%	2e-06	32.61%	2420	XP_001684146.1
zinc finger protein, putative [Entamoeba histolytica HM-1:IMSS]	*Entamoeba histolytica* HM-1:IMSS	294381	48.1	48.1	26%	4e-06	36.36%	395	XP_656065.1
conserved hypothetical protein [Leishmania major strain Friedlin]	*Leishmania major* strain Friedlin	347515	48.1	48.1	24%	5e-06	42.62%	636	XP_001682467.1
putative ras-like small GTPases [Leishmania major strain Friedlin]	*Leishmania major* strain Friedlin	347515	47.4	47.4	18%	1e-05	40.82%	766	

Top aligned sequence of Entamoeba histolytica

```
>XP_655361.1 Rho guanine nucleotide exchange factor, putative [Entamoeba histolytica HM-1:IMSS]
MSKRLYVVNEIVSTEESYARALQTVVDLYITPLKGMDLISEEDMKGLFCNLEELLDGANQLTKQLKDRLK
EFALNDNICDVFLLNKPLFNRFGPYIQNYEKASLLFDKLSKKKKFEEFCETQKENPLSNNGNLMSFLIFP
VQRIPRYILLMKEYIKNTEKDHPDAVIAEQVRRYLDSYAKAVNAIIANENNKQKMRDIIKMFTDLDPKTE
NDLKTLPRTLVREGILRKQCRKAVKERYFYLFNDATVIYGEQQGKKIQFHQLLELTSVKDIEDSDISQNG
FQLKGLQKSVVVYAETFDEKKEWVMDLNKAFTGERKYEFEEELSDNEAAPTWIPDDNVLDCMNCHSKFTL
LNRRHHCRKCGRVLCAECTKRRVVIPHISSKPVRVCENCATKFENKDFEGVDDNSEHVNANGVAPPSLPN
IPPPQRKQLIKISAMYPTLQPPSREPPELPQYESY
```

Top aligned sequence of Leishmania major

```
>XP_003722823.1 conserved hypothetical protein [Leishmania major strain Friedlin]
MSSSLSSPRPPQAEALPAPRGTIRFEDLGEPVQRLLRSLKGDVSEEDMALLEYVSVKDMAKIYSEMATDR
HAEEIWQELRSALQARKEKIARREAELAEKQRALEAEQEAEEQRLRQQLEEEERERISAEEARQQRREER
RRRKAEKAAAAAAAEEEAAEQAAAAAEEAAEEAAAAAEETERQRKAEKKRKRREARARELQEEQDALTAE
RARHETKKQQKSLNQQKDWSEFVQSHPLEYAQSETLDGAQQQLPAVIEQKELKHTLNAPPVVSEQLLSRT
YSPACGACGTRFDRPPPEWNCPMCERRYKKVVKVWQPDDDMENCMMCKEPIGRWSRHHCRNCGRLVCANC
SDKRAIIEDLGYDTPVRVCYDCFRLRS
```

There were no sequences found from Giardia lamblia

Multiple Protozoan Sequence Alignment with Human Phafin2

```
Homo_sapiens(Human)      -----------LKSPLNDM----SDDDDD---------D----DSSD-------------        249
XP_655361.1              -----------APPSLPNIPPPQRKQLIKI--------S--------AMYPTL-------        439
XP_649382.1              ENSEMSEAKGCTKEEFKIE----NTNDIIRDNEEAKAESTTLEESKDTEENNVM-----S        493
XP_651719.1              LDSISMESTSPLLK----------------------------------------------        441
XP_651964.1              QQKILSGGDINDSE--QND----DWQEF-----------------EDIEFDKQPSPRSLS        489
EMH78141.1               ------------------------------------------------------------        460
XP_655384.1              ------------------------------------------------------------        492
XP_656365.1              RSPRMSKSEKKDKQ--DK-------------K--KDKKKGKKSDSDDNNDNSQQT--KVV        464
EMS15045.1               QDQQLTTSSSNDDN--KKI----DVQPILQEE--SSNEQSTSVDVENMNNNEQP----LE        583
XP_651436.1              QDQQLTTSSSNDDN--KKI----DVQPILQEE--SNNEQSTSVDVENMNNNEQP----LE        583
EMD43911.1               QDQQLTTSSSNDDN--KKI----DVQPILQEE--SSNEQSTSVDVENMNNNEQP----LE        452
XP_656765.1              IRRDCKRSNSVGNPEEIFD----GLSVILNPDIQRT---QTANILNNVENSKSPLGRSIG        458
XP_654401.1              ----KDTTSSPALPEQSKQ----FNNPIH---------S----TNNTLSQEQTPSTIK--        483
XP_651674.1              --------------PLPSIPPPRRKTPFIQ--------N--------ATQFKKREPPR--        425
XP_651711.2              --TIRMKSSKEDEIVLRL--------------------------------EGVNLTRVRS        701
EMS16071.1               ENSEMSEAKGCTKEEFKIE----NTNDIIRDNEEAKAESTTLEESKDTEENNVM-----S        425
1Z2Q_A                   ------------------------------------------------------------        84
XP_003722823.1           ------------------------------------------------------------        377
CAG9576238.1             ----VLTTAGPGAPPVAAR----GQQDEGA--------GPTEPSTDDMNKPTSPLETP--        137
XP_001684146.1           ----VMDTYGHALLPLAGI----AKEPLKG--------G----ASADMKEPEKPSAAQ--        1896
XP_656065.1              ------------------------------------------------------------        395
XP_001682467.1           ------------------------------------------------------------        636
XP_001687717.1           QGLDVSSASSSALLPGPLP----VCS---D--------GNGHPPSNALTAPSAVTALP--        182
```
