# Supplementary material for: The PH Domain and C-Terminal polyD Motif of Phafin2 Exhibit a Unique Concurrence in Animals
Source: Membranes (Basel). 2022 Jul 7;12(7):696. doi: 10.3390/membranes12070696 (PMC9324892; doi:10.3390/membranes12070696)
Supplement: Supplementary file 1 [file membranes-12-00696-s001.zip › Supplementary File S6.pdf]

Supplementary File S6: Details of different modules found in model organisms

Domains Architecture of Arabidopsis thaliana

No	*Accession Number*	*Domains*
1	NP_001031110.1	Las17-binding protein actin regulator, FYVE zinc finger
2	NP_174273.3	Las17-binding protein actin regulator, FYVE zinc finger
3	NP_176362.3	FYVE zinc finger
4	NP_189909.1	Las17-binding protein actin regulator, FYVE zinc finge
5	NP_001320122.1	Phosphatidylinositol-4-phosphate 5-Kinase, TCP-1/cpn60 chaperonin family, FYVE zinc finger
6	NP_001328750.1	Phosphatidylinositol-4-phosphate 5-Kinase, TCP-1/cpn60 chaperonin family, FYVE zinc finger
7	NP_001190899.1	Phosphatidylinositol-4-phosphate 5-Kinase, TCP-1/cpn60 chaperonin family, FYVE zinc finger
8	NP_001078484.4	Phosphatidylinositol-4-phosphate 5-Kinase, TCP-1/cpn60 chaperonin family, FYVE zinc finger
9	NP_001328749.1	Phosphatidylinositol-4-phosphate 5-Kinase, TCP-1/cpn60 chaperonin family, FYVE zinc finger
10	NP_564103.1	FYVE zinc finger

11	NP_001319548.1	Phosphatidylinositol-4-phosphate 5-Kinase, TCP-1/cpn60 chaperonin family, FYVE zinc finger
12	NP_001328535.1	Regulator of chromosome condensation (RCC1) repeat, Transcription factor regulating root and shoot growth via Pin3, FYVE zinc finger, Transcription factor BRX N-terminal domain, PH
13	NP_001328536.1	Regulator of chromosome condensation (RCC1) repeat, Transcription factor regulating root and shoot growth via Pin3, FYVE zinc finger, Transcription factor BRX N-terminal domain, PH
14	NP_001154232.2	Regulator of chromosome condensation (RCC1) repeat, Transcription factor regulating root and shoot growth via Pin3, FYVE zinc finger, Transcription factor BRX N-terminal domain, PH
15	NP_001328534.1	Regulator of chromosome condensation (RCC1) repeat, Transcription factor regulating root and shoot growth via Pin3, FYVE zinc finger, Transcription factor BRX N-terminal domain, PH
16	NP_001327882.1	Regulator of chromosome condensation (RCC1) repeat, Transcription factor regulating root and shoot growth via Pin3, FYVE zinc finger, Transcription factor BRX N-terminal domain, PH
17	NP_188968.1	Regulator of chromosome condensation (RCC1) repeat, Transcription factor regulating root and shoot growth via Pin3, FYVE zinc finger, Transcription factor BRX N-terminal domain, PH
18	NP_001318728.1	Regulator of chromosome condensation (RCC1) repeat, Transcription factor regulating root and shoot growth via Pin3, FYVE zinc finger, Transcription factor BRX N-terminal domain, PH
19	NP_568268.3	Regulator of chromosome condensation (RCC1) repeat, Transcription factor regulating root and shoot growth via Pin3, FYVE zinc finger, Transcription factor BRX N-terminal domain, PH
20	NP_001322526.1	Regulator of chromosome condensation (RCC1) repeat, Transcription factor regulating root and shoot growth via Pin3, FYVE zinc finger, Transcription factor BRX N-terminal domain
21	NP_177129.2	Regulator of chromosome condensation (RCC1) repeat, Transcription factor regulating root and shoot growth via Pin3, FYVE zinc finger, Transcription factor BRX N-terminal domain
22	NP_001190338.1	Regulator of chromosome condensation (RCC1) repeat, Transcription factor regulating root and shoot growth via Pin3, FYVE zinc finger, Transcription factor BRX N-terminal domain, PH
23	NP_197443.3	Regulator of chromosome condensation (RCC1) repeat, Transcription factor regulating root and shoot growth via Pin3, FYVE zinc finger, Transcription factor BRX N-terminal domain, PH

24	NP_001330907.1	Regulator of chromosome condensation (RCC1) repeat, Transcription factor regulating root and shoot growth via Pin3, FYVE zinc finger, Transcription factor BRX N-terminal domain, PH
25	NP_565144.1	Regulator of chromosome condensation (RCC1) repeat, Transcription factor regulating root and shoot growth via Pin3, FYVE zinc finger, Transcription factor BRX N-terminal domain, PH
26	NP_001320309.1	Regulator of chromosome condensation (RCC1) repeat, Transcription factor regulating root and shoot growth via Pin3, FYVE zinc finger, Transcription factor BRX N-terminal domain, PH
27	NP_176767.1	Regulator of chromosome condensation (RCC1) repeat, Transcription factor regulating root and shoot growth via Pin3, FYVE zinc finger, Transcription factor BRX N-terminal domain
28	NP_196834.3	BAR domain of APPL family, Putative GTPase activating protein for Arf, Ankyrin repeats (many copies), Ankyrin repeat, PH domain, Ankyrin repeats

Retrieved sequences of Arabidopsis thaliana

>NP_001031110.1 RING/FYVE/PHD-type zinc finger family protein [Arabidopsis thaliana]
MDERDREIRASHGDSNAGNVVSQSIENTREEDSGSCEGFVEESKRLEPEQQKHGKYFFYDTPLSEETGVWIPVSVPPMLE
PDHEEWSRGLSFNGGYFPEGDMGWDQIFDEDKELTMWDVIVDMLLAARGKASALSSGNLERCGINFLSGHLLEQAWQDM
A
HTLTEANFGNAREILETEPPKWLPDSAASACMLCSVRFHPIMCSRHHCRYCGGIFCRDCSKGKSLVPVKFRVSDPQRVCD
VCFVRLESVQPYLMDQVSPAAQLPTHDLTDLSTLRSWVNFPWGQSMEYEIYKATNTLRGYITKVGSSRTERSIPDAILRQ
AKGLAVITVARVGVMVTYKIGTGLVVARRDDGSWSPPSAISSFGLGWGAQAGGEFIDFIIVLRTREAIQTFGSNTHLVVG
AGLSAAVGVTGRAVEADIRAGSGGYAACYTYSCSKGAFVGCSLEGSIFTTRTSENSRFYGSQSLAASDILLGSLPRPPAA
AALYRALGDLYQKMGR
>NP_174273.3 RING/FYVE/PHD-type zinc finger family protein [Arabidopsis thaliana]
MDERDREIRASHGDSNAGNVVSQSIENTREEDSGSCEGFVEESKRLEPEQQKHGKYFFYDTPLSEETGVWIPVSVPPMLE
PDHEEWSRGLSFNGGYFPEGDMGWDQIFDEDKELTMWDVIVDMLLAARGKASALSSGNLERCGINFLSGHLLEQAWQDM
A
HTLTEANFGNAREILETEPPKWLPDSAASACMLCSVRFHPIMCSRHHCRYCGGIFCRDCSKGKSLVPVKFRVSDPQRVCD
VCFVRLESVQPYLMDQVSPAAQLPTHDLTDLSTLRSWVNFPWGQSMEYEIYKATNTLRGYITKVGSSRTERSIPDAILRQ
AKGLAVITVARVGVMVTYKIGTGLVVARRDDGSWSPPSAISSFGLGWGAQAGGEFIDFIIVLRTREAIQTFGSNTHLVVG
AGLSAAVGVTGRAVEADIRAGSGGYAACYTYSCSKGAFVGCSLEGSIFTTRTSENSRFYGSQSLAASDILLGSLPRPPAA
AALYRALGDLYQKMGSETVRSPAISPLSED

>NP_176362.3 phosphoinositide binding protein [Arabidopsis thaliana]
MLEKIGLPPKPSLRGNSWVVDASHCQGCSSQFTFINRKHHCRRCGGLFCGTCTQQRLSLRGQGDSPVRICEPCKKIEEAA
RFELRHGYKNRAAKGGSSKRTVKNEDDVLSEILGSDVDVSSSSESVSSTDRNASKEMASSSSNKGMELDASPEELRKQAV
EAKNKYRILKGEGKSDEALKAFKRGRELEREADALEISLRRNRKRELSMRNVAETQNKAATKESSKSQKPLRQGGKGNDD
LAADLRELGWSDDEDKKPATISLEGEFSSLLREIPRSANPQKTGGIDKSQVIALKRKALTLKREGKLAEAKDELKKAKIL
ERELEEQELLGGADGSDDELSALINSMDDDKEDDLLAQYEGSHDFDISNLVGNLDDIGVHGEYDVTDEDMEDPAIAAALK
SLGWSEDPGHHENVHSRPSPKNRDESLAEIQTLKREALNLKRAGNVVEAMATLKKAKLLEKELEAADTSSETVDTTRAER
DTSLKPPPRSRLAIQKELLAVKKKALTLRREGKFNEAEEELKKGAVLQNQLDELDNSSKLAATGKATREKGNDLPDISSL
DDDGEVDVKDEELNDPNYLSMLKSLGWNDEDNNPAGPSSEKSDPLNSRPGKTAEAQGAYEVRVTKPRRTKAEIQRELLGL
KRKALTLRRQGNVDEAEEVLNQTQILEAQIMEIDSGKNLYADSDQPKKRSNDLATDSRLNGGDDSVTENDMKDPALLSTL
KNLGWEDEEPKKEEASFGSVQSSGPRIAAKSKGQIQRELLDLKRKALAFKRQGKTGDADELYSKASVLEAQLAELETPKM
EMKGSASAIKPENYMDVDLLVGSQMEDKAIKSASVSHAPQDSYDLLGDFISPAKSGSSGVVSQPGQQQPSMMDLLTGEHS
ERSQIHAEKGNAETMSGFRSGNNHGAEQRVAREESEPSHIQSASIQNTSPQNTLKQEILAHKKKALALKREGNISEAKKA
LQEAKLLERRLQEGENPSPEKLGRDDMVSTTEDPPAREKENSPSSSAPKAMSGRDRFKLQQESLSHKRQAMKLRREGKMQ
EAEAEFEIAKTLEAQLEDSTSSKPEPVDDVAVEDFLDPQLLSALKAIGLDNPVNPPPVSKTDTTQAAAKPNPVKESNRNT
NNQERSQLEERIKAEKVKAVTFKRAGKQAEALDALRRAKLYEKKLNALASS
>NP_189909.1 RING/FYVE/PHD-type zinc finger family protein [Arabidopsis thaliana]
MATLNGKASPYSEENIKFKYDDYDDDDDDDDGSGSGSGGGYEDGPKWSVQSIPTKKEVEYPIIDSGDYVDDGYDSSGELS
TTTTPPIQGNEKPEVNLKNVLTGLIAIVTGRNKNPLDQKNPSSNVSFLGSGTNGDTFVHSSVYIPSAPPLLEPSGINYSV
YKELLEAEPPEWLPDSLASTCMQCSTPFTAITCGRHHCRFCGGIFCRNCSKGRCLMPSRFRERNPQRVCDSCYERLDPLQ
CVLINSISNAVQVAKHDVVDWTCSRGWLNLPVGLSMEDEIYKAANTLRGYCQVARLDPEKSIPHAVLSRAKGLAIITVAK
AGALLSYKLGTGLVISRRPDGSWSAPSAILSVGLGWGAQIGGELMDFIIVLHDVKAVKTFCSRMHFSLGAGCSAAAGPIG
RVLEADLRAGDKGSGVCYTYSRSKGAFVGVSLEGNLVATRRDMNVKFYGDPYLSTSDILLGMVDQPKAAEPLYTALRELY
ARLRP
>NP_001320122.1 1-phosphatidylinositol-3-phosphate 5-kinase FAB1A [Arabidopsis thaliana]
MDSQDHKAPGFVDIVKSWIPRKSESSNMSRDFWMPDQSCPVCYECDAQFTVFNRRHHCRLCGRVFCAKCAANSIPSPSDE
TKDSHEEPERIRVCNYCYKQWEQGIVPPDNGASIISLHFSSSPSARSVASTTSNSSNCTIDSTAGPSPRPKMNPRASRRV
SSNMDSEKSEQQNAKSRRSSDHYGHVLDSSDNQVEFFVNSSGRSDGEADDDDDYQSDFAQSYAQGNDYYGAINLDEVDHI
YGSHEAHDVGVKIEPNISGFPPDQDLDSLNTETIDKTRQQENGWNDVKEGSPPCEESFEPEVVDFESDGLLWLPPEPENE
EDEREAVLSDDDGDEGDRGDWGYLRPSNSFNEKDFHSKDKSSGAMKNVVEGHFRALVAQLLEVDNLPMVNEGDEEGWLD
I
ITSLSWEAATLLKPDTSKSGGMDPGGYVKVKCIPCGRRSESMVVKGVVCKKNVAHRRMTSKIEKPRLLILGGALEYQRIS
NQLSSFDTLLQQEMDHLKMAVAKIDSHNPDILLVEKSVSRFAQEYLLAKDISLVLNIKRSLLERISRCTGAQIVPSIDQL

TSPKLGYCDLFHVEKFVETHVSPCQVAKKMAKTLMFFDGCPKPLGCTILLKGAHEDELKKVKHVIQYGVFAAYHLALETS
FLADEGASIHELPLQTPITVALPDKPSMVNRSISTIPGFTVSSAEKSPTTELRGEPHKANGDLTGNFTSSKTHFQGKLDG
NDRIDPSERLLHNLDTVYCKPPETITSKDDGLVPTLESRQLSFHVEEPSVQKDQWSVLSGATEQVTDGGYTNDSAVIGNQ
NFNRQEQMESSKGDFHPSASDHQSILVSLSTRCVWKGSVCERAHLLRIKYYGSFDKPLGRFLRDNLFDQDQCCPSCTMPA
EAHIHCYTHRQGSLTISVKKLPELLPGQREGKIWMWHRCLKCPRINGFPPATRRIVMSDAAWGLSFGKFLELSFSNHAAA
SRVANCGHSLHRDCLRFYGFGRMVACFRYASINIYAVTLPPAKLYFNYENQEWLQKESKEVIKKAEVLFNEVQEALSQIS
AKTMGAGSKGSTPNKIKLSLEELAGLLEQRKKEYKDSLQQMLNVVKDGQPTIDILLINKLRRLIIFDSYAWDECLAGAAN
MVRNNYLEAPKNSAPKVMGRNVSLEKLSDEKVKSIPTHVAICNDSLLQDADYETCLNQGKSFADTSGKFAIPEDVGSDRP
PDCRMEFDPSEGGKDNFVESSQVVKPAHTESQFQATDLSDTLDAAWIGEQTTSENGIFRPPSRAASTNGTQIPDLRLLGS
ESELNFKGGPTNDEHTTQVQLPSPSFYYSLNKNYSLNSRKHIMAEDRPVYVSSYRELEWRSGARLLLPLGCNDLVLPVYD
DEPTSIIAYALTSSEYKAQMSGSDKSRDRLDSGGSFSLFDSVNLLSLNSLSDLSVDMSRSLSSADEQVSQLLHSSLYLKD
LHARISFTDEGPPGKVKYSVTCYYAKEFEALRMICCPSETDFIRSLGRCRKWGAQGGKSNVFFAKSLDDRFIIKQVTKTE
LESFIKFGPAYFKYLTESISTKSPTSLAKILGIYQVSSKHLKGGKEFKMDVLVMENLLFKRNFTRLYDLKGSTRARYNPD
TSGSNTVLLDQNLVEAMPTSPIFVGSKAKRLLERAVWNDTSFLASIHVMDYSLLVGVDEERNELVLGIIDFMRQYTWDKH
LETWVKTSGLLGGPKNSTPTVISPQQYKKRFRKAMTAYFLMVPDQWSPAAVVPSNSSSAEVKEEEEKDNPQAVGNKS
>NP_001328750.1 1-phosphatidylinositol-3-phosphate 5-kinase FAB1A [Arabidopsis thaliana]
MDSQDHKAPGFVDIVKSWIPRKSESSNMSRDFWMPDQSCPVCYECDAQFTVFNRRHHCRLCGRVFCAKCAANSIPSPSDE
TKDSHEEPERIRVCNYCYKQWEQGIVPPDNGASIISLHFSSSPSARSVASTTSNSSNCTIDSTAGPSPRPKMNPRASRRV
SSNMDSEKSEQQNAKSRRSSDHYGHVLDSSDNQVEFFVNRSDGEADDDDDYQSDFAQSYAQGNDYYGAINLDEVDHIYGS
HEAHDVGVKIEPNISGFPPDQDLDSLNTETIDKTRQQENGWNDVKEGSPPCEESFEPEVVDFESDGLLWLPPEPENEEDE
REAVLSDDDGDEGDRGDWGYLRPSNSFNEKDFHSKDKSSGAMKNVVEGHFRALVAQLLEVDNLPMVNEGDEEGWLDIITS
LSWEAATLLKPDTSKSGGMDPGGYVKVKCIPCGRRSESMVVKGVVCKKNVAHRRMTSKIEKPRLLILGGALEYQRISNQL
SSFDTLLQQEMDHLKMAVAKIDSHNPDILLVEKSVSRFAQEYLLAKDISLVLNIKRSLLERISRCTGAQIVPSIDQLTSP
KLGYCDLFHVEKFVETHVSPCQVAKKMAKTLMFFDGCPKPLGCTILLKGAHEDELKKVKHVIQYGVFAAYHLALETSFLA
DEGASIHELPLQTPITVALPDKPSMVNRSISTIPGFTVSSAEKSPTTELRGEPHKANGDLTGNFTSSKTHFQGKLDGNDR
IDPSERLLHNLDTVYCKPPETITSKDDGLVPTLESRQLSFHVEEPSVQKDQWSVLSGATEQVTDGGYTNDSAVIGNQNFN
RQEQMESSKGDFHPSASDHQSILVSLSTRCVWKGSVCERAHLLRIKYYGSFDKPLGRFLRDNLFDQDQCCPSCTMPAEAH
IHCYTHRQGSLTISVKKLPELLPGQREGKIWMWHRCLKCPRINGFPPATRRIVMSDAAWGLSFGKFLELSFSNHAAASRV
ANCGHSLHRDCLRFYGFGRMVACFRYASINIYAVTLPPAKLYFNYENQEWLQKESKEVIKKAEVLFNEVQEALSQISAKT
MGAGSKGSTPNKIKLSLEELAGLLEQRKKEYKDSLQQMLNVVKDGQPTIDILLINKLRRLIIFDSYAWDECLAGAANMVR
NNYLEAPKNSAPKVMGRNVSLEKLSDEKVKSIPTHVAICNDSLLQDADYETCLNQGKSFADTSGKFAIPEDVGSDRPPDC
RMEFDPSEGGKDNFVESSQVVKPAHTESQFQATDLSDTLDAAWIGEQTTSENGIFRPPSRAASTNGTQIPDLRLLGSESE
LNFKGGPTNDEHTTQVQLPSPSFYYSLNKNYSLNSRKHIMAEDRPVYVSSYRELEWRSGARLLLPLGCNDLVLPVYDDEP

TSIIAYALTSSEYKAQMSGSDKSRDRLDSGGSFSLFDSVNLLSLNSLSDLSVDMSRSLSSADEQVSQLLHSSLYLKDLHA
RISFTDEGPPGKVKYSVTCYYAKEFEALRMICCPSETDFIRSLGRCRKWGAQGGKSNVFFAKSLDDRFIIKQVTKTELES
FIKFGPAYFKYLTESISTKSPTSLAKILGIYQVSSKHLKGGKEFKMDVLVMENLLFKRNFTRLYDLKGSTRARYNPDTSG
SNTVLLDQNLVEAMPTSPIFVGSKAKRLLERAVWNDTSFLASIHVMDYSLLVGVDEERNELVLGIIDFMRQYTWDKHLET
WVKTSGLLGGPKNSTPTVISPQQYKKRFRKAMTAYFLMVPDQWSPAAVVPSNSSSAEVKEEEEKDNPQAVGNKS
>NP_001190899.1 1-phosphatidylinositol-3-phosphate 5-kinase FAB1A [Arabidopsis thaliana]
MDSQDHKAPGFVDIVKSWIPRKSESSNMSRDFWMPDQSCPVCYECDAQFTVFNRRHHCRLCGRVFCAKCAANSIPSPSDE
TKDSHEEPERIRVCNYCYKQWEQGIVPPDNGASIISLHFSSSPSARSVASTTSNSSNCTIDSTAGPSPRPKMNPRASRRV
SSNMDSEKSEQQNAKSRRSSDHYGHVLDSSDNQVEFFVNSSGRSDGEADDDDDYQSDFAQSYAQGNDYYGAINLDEVDHI
YGSHEAHDVGVKIEPNISGFPPDQDLDSLNTETIDKTRQQENGWNDVKEGSPPCEESFEPEVVDFESDGLLWLPPEPENE
EDEREAVLSDDDGDEGDRGDWGYLRPSNSFNEKDFHSKDKSSGAMKNVVEGHFRALVAQLLEVDNLPMVNEGDEEGWLD
I
ITSLSWEAATLLKPDTSKSGGMDPGGYVKVKCIPCGRRSESMVVKGVVCKKNVAHRRMTSKIEKPRLLILGGALEYQRIS
NQLSSFDTLLQQEMDHLKMAVAKIDSHNPDILLVEKSVSRFAQEYLLAKDISLVLNIKRSLLERISRCTGAQIVPSIDQL
TSPKLGYCDLFHVEKFVETHVSPCQVAKKMAKTLMFFDGCPKPLGCTILLKGAHEDELKKVKHVIQYGVFAAYHLALETS
FLADEGASIHELPLQTPITVALPDKPSMVNRSISTIPGFTVSSAEKSPTTELRGEPHKANGDLTGNFTSSKTHFQGKLDG
NDRIDPSERLLHNLDTVYCKPPETITSKDDGLVPTLESRQLSFHVEEPSVQKDQWSVLSGATEQVTDGGYTNDSAVIGNQ
NFNRQEQMESSKGDFHPSASDHQSILVSLSTRCVWKGSVCERAHLLRIKYYGSFDKPLGRFLRDNLFDQDQCCPSCTMPA
EAHIHCYTHRQGSLTISVKKLPELLPGQREGKIWMWHRCLKCPRINGFPPATRRIVMSDAAWGLSFGKFLELSFSNHAAA
SRVANCGHSLHRDCLRFYGFGRMVACFRYASINIYAVTLPPAKLYFNYENQEWLQKESKEVIKKAEVLFNEVQEALSQIS
AKTMGAGSKGSTPNKIKLSLEELAGLLEQRKKEYKQMLNVVKDGQPTIDILLINKLRRLIIFDSYAWDECLAGAANMVRN
NYLEAPKNSAPKVMGRNVSLEKLSDEKVKSIPTHVAICNDSLLQDADYETCLNQGKSFADTSGKFAIPEDVGSDRPPDCR
MEFDPSEGGKDNFVESSQVVKPAHTESQFQATDLSDTLDAAWIGEQTTSENGIFRPPSRAASTNGTQIPDLRLLGSESEL
NFKGGPTNDEHTTQVQLPSPSFYYSLNKNYSLNSRKHIMAEDRPVYVSSYRELEWRSGARLLLPLGCNDLVLPVYDDEPT
SIIAYALTSSEYKAQMSGSDKSRDRLDSGGSFSLFDSVNLLSLNSLSDLSVDMSRSLSSADEQVSQLLHSSLYLKDLHAR
ISFTDEGPPGKVKYSVTCYYAKEFEALRMICCPSETDFIRSLGRCRKWGAQGGKSNVFFAKSLDDRFIIKQVTKTELESF
IKFGPAYFKYLTESISTKSPTSLAKILGIYQVSSKHLKGGKEFKMDVLVMENLLFKRNFTRLYDLKGSTRARYNPDTSGS
NTVLLDQNLVEAMPTSPIFVGSKAKRLLERAVWNDTSFLASIHVMDYSLLVGVDEERNELVLGIIDFMRQYTWDKHLETW
VKTSGLLGGPKNSTPTVISPQQYKKRFRKAMTAYFLMVPDQWSPAAVVPSNSSSAEVKEEEEKDNPQAVGNKS
>NP_001078484.4 1-phosphatidylinositol-3-phosphate 5-kinase FAB1A [Arabidopsis thaliana]
MDSQDHKAPGFVDIVKSWIPRKSESSNMSRDFWMPDQSCPVCYECDAQFTVFNRRHHCRLCGRVFCAKCAANSIPSPSDE
TKDSHEEPERIRVCNYCYKQWEQGIVPPDNGASIISLHFSSSPSARSVASTTSNSSNCTIDSTAGPSPRPKMNPRASRRV
SSNMDSEKSEQQNAKSRRSSDHYGHVLDSSDNQVEFFVNSGRSDGEADDDDDYQSDFAQSYAQGNDYYGAINLDEVDHIY

GSHEAHDVGVKIEPNISGFPPDQDLDSLNTETIDKTRQQENGWNDVKEGSPPCEESFEPEVVDFESDGLLWLPPEPENEE
DEREAVLSDDDGDEGDRGDWGYLRPSNSFNEKDFHSKDKSSGAMKNVVEGHFRALVAQLLEVDNLPMVNEGDEEGWLDII
TSLSWEAATLLKPDTSKSGGMDPGGYVKVKCIPCGRRSESMVVKGVVCKKNVAHRRMTSKIEKPRLLILGGALEYQRISN
QLSSFDTLLQQEMDHLKMAVAKIDSHNPDILLVEKSVSRFAQEYLLAKDISLVLNIKRSLLERISRCTGAQIVPSIDQLT
SPKLGYCDLFHVEKFVETHVSPCQVAKKMAKTLMFFDGCPKPLGCTILLKGAHEDELKKVKHVIQYGVFAAYHLALETSF
LADEGASIHELPLQTPITVALPDKPSMVNRSISTIPGFTVSSAEKSPTTELRGEPHKANGDLTGNFTSSKTHFQGKLDGN
DRIDPSERLLHNLDTVYCKPPETITSKDDGLVPTLESRQLSFHVEEPSVQKDQWSVLSGATEQVTDGGYTNDSAVIGNQN
FNRQEQMESSKGDFHPSASDHQSILVSLSTRCVWKGSVCERAHLLRIKYYGSFDKPLGRFLRDNLFDQDQCCPSCTMPAE
AHIHCYTHRQGSLTISVKKLPELLPGQREGKIWMWHRCLKCPRINGFPPATRRIVMSDAAWGLSFGKFLELSFSNHAAAS
RVANCGHSLHRDCLRFYGFGRMVACFRYASINIYAVTLPPAKLYFNYENQEWLQKESKEVIKKAEVLFNEVQEALSQISA
KTMGAGSKGSTPNKIKLSLEELAGLLEQRKKEYKDSLQQMLNVVKDGQPTIDILLINKLRRLIIFDSYAWDECLAGAANM
VRNNYLEAPKNSAPKVMGRNVSLEKLSDEKVKSIPTHVAICNDSLLQDADYETCLNQGKSFADTSGKFAIPEDVGSDRPP
DCRMEFDPSEGGKDNFVESSQVVKPAHTESQFQATDLSDTLDAAWIGEQTTSENGIFRPPSRAASTNGTQIPDLRLLGSE
SELNFKGGPTNDEHTTQVQLPSPSFYYSLNKNYSLNSRKHIMAEDRPVYVSSYRELEWRSGARLLLPLGCNDLVLPVYDD
EPTSIIAYALTSSEYKAQMSGSDKSRDRLDSGGSFSLFDSVNLLSLNSLSDLSVDMSRSLSSADEQVSQLLHSSLYLKDL
HARISFTDEGPPGKVKYSVTCYYAKEFEALRMICCPSETDFIRSLGRCRKWGAQGGKSNVFFAKSLDDRFIIKQVTKTEL
ESFIKFGPAYFKYLTESISTKSPTSLAKILGIYQVSSKHLKGGKEFKMDVLVMENLLFKRNFTRLYDLKGSTRARYNPDT
SGSNTVLLDQNLVEAMPTSPIFVGSKAKRLLERAVWNDTSFLASIHVMDYSLLVGVDEERNELVLGIIDFMRQYTWDKHL
ETWVKTSGLLGGPKNSTPTVISPQQYKKRFRKAMTAYFLMVPDQWSPAAVVPSNSSSAEVKEEEEKDNPQAVGNKS
>NP_001328749.1 1-phosphatidylinositol-3-phosphate 5-kinase FAB1A [Arabidopsis thaliana]
MDSQDHKAPGFVDIVKSWIPRKSESSNMSRDFWMPDQSCPVCYECDAQFTVFNRRHHCRLCGRVFCAKCAANSIPSPSDE
TKDSHEEPERIRVCNYCYKQWEQGIVPPDNGASIISLHFSSSPSARSVASTTSNSSNCTIDSTAGPSPRPKMNPRASRRV
SSNMDSEKSEQQNAKSRRSSDHYGHVLDSSDNQVEFFVNSSGRSDGEADDDDDYQSDFAQSYAQGNDYYGAINLDEVDHI
YGSHEAHDVGVKIEPNISGFPPDQDLDSLNTETIDKTRQQENGWNDVKEGSPPCEESFEPEVVDFESDGLLWLPPEPENE
EDEREAVLSDDDGDEGDRGDWGYLRPSNSFNEKDFHSKDKSSGAMKNVVEGHFRALVAQLLEVDNLPMVNEGDEEGWLD
I
ITSLSWEAATLLKPDTSKSGGMDPGGYVKVKCIPCGRRSESMVVKGVVCKKNVAHRRMTSKIEKPRLLILGGALEYQRIS
NQLSSFDTLLQQEMDHLKMAVAKIDSHNPDILLVEKSVSRFAQEYLLAKDISLVLNIKRSLLERISRCTGAQIVPSIDQL
TSPKLGYCDLFHVEKFVETHVSPCQVAKKMAKTLMFFDGCPKPLGCTILLKGAHEDELKKVKHVIQYGVFAAYHLALETS
FLADEGASIHELPLQTPITVALPDKPSMVNRSISTIPGFTVSSAEKSPTTELRGEPHKANGDLTGNFTSSKTHFQGKLDG
NDRIDPSERLLHNLDTVYCKPPETITSKDDGLVPTLESRQLSFHVEEPSVQKDQWSVLSGATEQVTDGGYTNDSAVIGNQ
NFNRQEQMESSKGDFHPSASDHQSILVSLSTRCVWKGSVCERAHLLRIKYYGSFDKPLGRFLRDNLFDQDQCCPSCTMPA
EAHIHCYTHRQGSLTISVKKLPELLPGQREGKIWMWHRCLKCPRINGFPPATRRIVMSDAAWGLSFGKFLELSFSNHAAA

SRVANCGHSLHRDCLRFYGFGRMVACFRYASINIYAVTLPPAKLYFNYENQEWLQKESKEVIKKAEVLFNEVQEALSQIS
AKTMGAGSKGSTPNKIKLSLEELAGLLEQRKKEYKDSLQQMLNVVKDGQPTIDILLINKLRRLIIFDSYAWDECLAGAAN
MVRNNYLEAPKNSAPKVMGRNVSLEKLSDEKVKSIPTHVAICNDSLLQDADYETCLNQESSQVVKPAHTESQFQATDLSD
TLDAAWIGEQTTSENGIFRPPSRAASTNGTQIPDLRLLGSESELNFKGGPTNDEHTTQVQLPSPSFYYSLNKNYSLNSRK
HIMAEDRPVYVSSYRELEWRSGARLLLPLGCNDLVLPVYDDEPTSIIAYALTSSEYKAQMSGSDKSRDRLDSGGSFSLFD
SVNLLSLNSLSDLSVDMSRSLSSADEQVSQLLHSSLYLKDLHARISFTDEGPPGKVKYSVTCYYAKEFEALRMICCPSET
DFIRSLGRCRKWGAQGGKSNVFFAKSLDDRFIIKQVTKTELESFIKFGPAYFKYLTESISTKSPTSLAKILGIYQVSSKH
LKGGKEFKMDVLVMENLLFKRNFTRLYDLKGSTRARYNPDTSGSNTVLLDQNLVEAMPTSPIFVGSKAKRLLERAVWNDT
SFLASIHVMDYSLLVGVDEERNELVLGIIDFMRQYTWDKHLETWVKTSGLLGGPKNSTPTVISPQQYKKRFRKAMTAYFL
MVPDQWSPAAVVPSNSSSAEVKEEEEKDNPQAVGNKS
>NP_564103.1 RING/FYVE/PHD zinc finger superfamily protein [Arabidopsis thaliana]
MQQGDYNSYYHHQYSQFQNPTPNPNPNPNPSPPAPATVAGPTDLTRNTYASAPPFTGGYGSADYSNYSQNYTPYGQNSEH
VPPSAPSFTSPSQPPPSPPATSLNPNSYSTFNQPPPPPTIHPQPLSSYGSFDSTAPYQQPTSQHMYYSPYDQHQTSGYSS
APPPSSAPAPNPNPAPYSSSLYSAPPYSSGGSSIPPSYEKPSVKFDQSGYDGYNRSRSDLGSDLYGKRSDSGEYPAFEDS
YGDGVYAYQGGKVEPYGSRGTAPKSSNSTLFDDYGRSISFSSSGRDSSVSSNSAKIVRAVPKADVQEDSTGGVQKFRVKL
LAETYGQTTTDVLCQIGLDGLRMLDPSTSRTLRIYPLENITRCEKLDSSILAFWSKTPVDIEAKRIRLQSNSYTTNTLLD
TVTAAMFQAKEIGGSSRPPTSGKLIEQTAEKKKGLGDWMNIIKPVNEEKDHWVPDEAVSKCTSCGSDFGAFIRRHHCRNC
GDVFCDKCTQGRIALTAEDNAPQVRVCDRCMAEVSQRLSNAKETTGRNVSLQSHEDLARKLQEEMERNRKSSSGLREGSG
RRMKEVACPTCTVHLQVQVPVSGSETIECGVCQNPFLVSAH
>NP_001319548.1 phosphatidylinositol-4-phosphate 5-kinase family protein [Arabidopsis thaliana]
MGTRDSNNRTFSEIVGLIKSWLPWRSEPATVSRDFWMPDQSCRVCYECDCQFTLINRRHHCRHCGRVFCGKCTANSIPFA
PSDLRTPREDWERIRVCNYCFRQWEQGDGGPHVSNITELSTSPSETSLLSSKTSTTANSSSFALGSMPGLIGLNQRVHHG
SDVSLHGVSSMETSVTKQGKETSRRSSFIATDVEDPSRFALNSIRSDDEYDEYGAYQTDIETSHSPRANEYYGPMEYNGM
GIDDVPCKHLGGETADQKSLSGSPLIHQCLESLIREGSEQFQKKSEHDGRDECEASSPADISDDQVVEPVDFENNGLLWV
PPEPENEEDERESALFDEEDNEGDASGEWGYLRPSTSFGSGEYRGEDRTTEEHKKAMKNVVDGHFRALLAQLLQVENISV
SDEEGKESWLEIITSLSWEAANLLKPDMSKSGGMDPGGYVKVKCLASGFRHDSMVVKGVVCKKNVVNRRMSTKIEKARLL
ILGGGLEYQRVSNQLSSFDTLLQQEKDHLKMAVAKIHAERPNILLVEKSVSRFAQEYLLAKDISLVLNIKRPLLDRIARC
TGAQIIPSVDHLSSQKLGYCENFRVDRYPEEHGSTGQVGKKVVKTLMYFEHCPKPLGFTILLRGANEDELKKVKHVVQYG
VFAAYHLALETSFLADEGASPELPLNSPITVALPDKSTSIERSISTVPGFTVSTYEKSPTMLSCAEPQRANSVPVSELLS
TTTNLSIQKDIPPIPYGSGWQAREINPSFVFSRHNISLNLPDRVIESRNSDLSGRSVPVDTPADKSNPIVVADETTNNSL
HLSGQGFVRKSSQIGTSIMVENQDNGSELTIAQQQNNEKPKETQSQKEEFPPSPSDHQSILVSLSSRSVWKGTVCERSHL
FRIKYYGSFDKPLGRFLRDHLFDQSYRCRSCEMPSEAHVHCYTHRQGSLTISVKKLQDYLLPGEKEGKIWMWHRCLRCPR
LNGFPPATLRVVMSDAAWGLSFGKFLELSFSNHAAASRVACCGHSLHRDCLRFYGFGNMVACFRYATIDVHSVYLPPSIL

SFNYENQDWIQRETDEVIERAELLFSEVLNAISQIAEKGFRRRIGELEEVLQKEKAEFEENMQKILHREVNEGQPLVDIL
ELYRIHRQLLFQSYMWDHRLINASTLHKLENSDDTKREENEKPPLAKSQTLPEMNAGTNSLLTGSEVNLNPDGDSTGDTG
SLNNVQKEADTNSDLYQEKDDGGEVSPSKTLPDTSYPLENKVDVRRTQSDGQIVMKNLSATLDAAWIGERQTSVEIPTNN
KVSLPPSTMSNSSTFPPISEGLMPIDLPEQQNEFKVAYPVSPALPSKNYENSEDSVSWLSVPFLNFYRSINKNFLLSSQK
LDTFGEHSPIYISSFREAELQGGPRLLLPVGLNDIVVPVYDDEPTSMIAYALMSPEYQRQTSAEGESLVSYPSELNIPRP
VDDTIFDPSRSNGSVDESILSISSSRSTSLLDPLSYTKALHARVSYGEDGTLGKVKYTVTCYYAKRFEALRGICLPSELE
YIRSLSRCKKWGAQGGKSNVFFAKTLDDRFIIKQVTKTELESFIKFAPAYFKYLSESISTKSPTCLAKILGIYQVATKQL
KSGKETKMDVLIMENLLFGRTVKRLYDLKGSSRARYNPDSSGSNKVLLDQNLIEAMPTSPIFVGNKAKRLLERAVWNDTA
FLALGDVMDYSLLVGVDEEKNELVLGIIDFLRQYTWDKHLESWVKFTGILGGPKNEAPTVISPKQYKRRFRKAMTTYFLM
VPDQWSPPNVVANNSKSDQPEETSQAGTQAE
>NP_001328535.1 Regulator of chromosome condensation (RCC1) family protein [Arabidopsis thaliana]
MKTDGMADPSSYVNHDRDIDQALVSLKKGTQLLKYSRKGRPKFRSFRLSPDETTLFWLSHGEEKGLKLATVSRILPGQRT
AVFRRYLRPEKDYLSFSLIYHNGDRSLDLICKDKAETEVWFAGLKSLIRQNRNKQAKSEIPEIHDSDCFSTGRPSTASID
FAPNNTRRGRTSIDLGIQNSPTKFGSSDVGYERGNMLRPSTDGFRISVSSTPSCSTGTSGPDDIESLGDVYVWGEVWSDG
ISPDGVVNSTTVKIDVLIPKPLESNVVLDVHQIACGVRHIALVTRQGEVFTWEEEAGGRLGHGIQVDVCRPKLVEFLALT
NIDFVACGEYHTCAVSTSGDLFTWGDGIHNVGLLGHGSDLSHWIPKRVSGPVEGLQVLSVACGTWHSALATANGKLFTFG
DGAFGVLGHGDRESVSYPKEVKMLSGLKTLKVACGVWHTVAIVEVMNQTGTSTSSRKLFTWGDGDKNRLGHGNKETYLL
P
TCVSSLIDYNFNQIACGHTFTVALTTSGHVFTMGGTSHGQLGSSNSDGKLPCLVQDRLVGEFVEEISCGDHHVAVLTSRS
EVFTWGKGSNGRLGHGDKDDRKTPTLVEALRERHVKSISCGSNFTSSICIHKWVSGADQSVCSGCRQAFGFTRKRHNCYN
CGLVHCHACSSKKALKAALAPTPGKPHRVCDACYTKLKAGESGYNSNVANRNSTTPTRSLDGTGRPDRDIRSSRILLSPK
TEPVKYSEVRSSRSESSIVRASQVPALQQLRDVAFPSSLSAIQNAFKPVASSSTSTLPSGTRSSRISSPPRSSGFSRGMI
DTLKKSNGVINKEMTKLQSQIKNLKEKCDNQGTEIQRLKKTAREASDLAVKHSSKHKAATEVMKSVAEHLRELKEKLPPE
VSRCEAFESMNSQAEAYLNASEASESCLPTTSLGMGQRDPTPSTNTQDQNIEEKQSSNGGNMRSQEPSGTTEASSSSKGG
GKELIEQFEPGVYVTYVLHKNGGKIFRRVRFSKRRFDEHQAEEWWNSKKDRLLKRYSHHASSSSPTASDSVPTPTQPQPP
ASDSVPTPTQPQPPASDSVPTPTQPQPPASPTQPNSDQAEDSKEVLDES
>NP_001328536.1 Regulator of chromosome condensation (RCC1) family protein [Arabidopsis thaliana]
MKTDGMADPSSYVNHDRDIDQALVSLKKGTQLLKYSRKGRPKFRSFRLSPDETTLFWLSHGEEKGLKLATVSRILPGQRT
AVFRRYLRPEKDYLSFSLIYHNGDRSLDLICKDKAETEVWFAGLKSLIRQNRNKQAKSEIPEVSIHDSDCFSTGRPSTAS
IDFAPNNTRRGRTSIDLGIQNSPTKFGSSDVGYERGNMLRPSTDGFRISVSSTPSCSTGTSGPDDIESLGDVYVWGEVWS
DGISPDGVVNSTTVKIDVLIPKPLESNVVLDVHQIACGVRHIALVTRQGEVFTWEEEAGGRLGHGIQVDVCRPKLVEFLA
LTNIDFVACGEYHTCAVSTSGDLFTWGDGIHNVGLLGHGSDLSHWIPKRVSGPVEGLQVLSVACGTWHSALATANGKLFT

FGDGAFGVLGHGDRESVSYPKEVKMLSGLKTLKVACGVWHTVAIVEVMNQTGTSTSSRKLFTWGDGDKNRLGHGNKETY
L
LPTCVSSLIDYNFNQIACGHTFTVALTTSGHVFTMGGTSHGQLGSSNSDGKLPCLVQDRLVGEFVEEISCGDHHVAVLTS
RSEVFTWGKGSNGRLGHGDKDDRKTPTLVEALRERHVKSISCGSNFTSSICIHKWVSGADQSVCSGCRQAFGFTRKRHNC
YNCGLVHCHACSSKKALKAALAPTPGKPHRVCDACYTKLKAGESGYNSNVANRNSTTPTRSLDGTGRPDRDIRSSRILLS
PKTEPVKYSEVRSSRSESSIVRASQVPALQQLRDVAFPSSLSAIQNAFKPVASSSTSTLPSGTRSSRISSPPRSSGFSRG
MIDTLKKSNGVINKEMTKLQSQIKNLKEKCDNQGTEIQRLKKTAREASDLAVKHSSKHKAATEVMKSVAEHLRELKEKLP
PEVSRCEAFESMNSQAEAYLNASEASESCLPTTSLGMGQRDPTPSTNTQDQNIEEKQSSNGGNMRSQEPSGTTEASSSSK
GGGKELIEQFEPGVYVTYVLHKNGGKIFRRVRFSKRRFDEHQAEEWWNSKKDRLLKRYSHHASSSSPTASDSVPTPTQPQ
PPASDSVPTPTQPQPPASDSVPTPTQPQPPASPTQPNSDQAEDSKEVLDES
>NP_001154232.2 Regulator of chromosome condensation (RCC1) family protein [Arabidopsis thaliana]
MKTDGMADPSSYVNHDRDIDQALVSLKKGTQLLKYSRKGRPKFRSFRLSPDETTLFWLSHGEEKGLKLATVSRILPGQRT
AVFRRYLRPEKDYLSFSLIYHNGDRSLDLICKDKAETEVWFAGLKSLIRQNRNKQAKSEIPEVSIHDSDCFSTGRPSTAS
IDFAPNNTRRGRTSIDLGIQNSPTKFGSSDVGYERGNMLRPSTDGFRISVSSTPSCSTGTSGPDDIESLGDVYVWGEVWS
DGISPDGVVNSTTVKIDVLIPKPLESNVVLDVHQIACGVRHIALVTRQGEVFTWEEEAGGRLGHGIQVDVCRPKLVEFLA
LTNIDFVACGEYHTCAVSTSGDLFTWGDGIHNVGLLGHGSDLSHWIPKRVSGPVEGLQVLSVACGTWHSALATANGKLFT
FGDGAFGVLGHGDRESVSYPKEVKMLSGLKTLKVACGVWHTVAIVEVMNQTGTSTSSRKLFTWGDGDKNRLGHGNKETY
L
LPTCVSSLIDYNFNQIACGHTFTVALTTSGHVFTMGGTSHGQLGSSNSDGKLPCLVQDRLVGEFVEEISCGDHHVAVLTS
RSEVFTWGKGSNGRLGHGDKDDRKTPTLVEALRERHVKSISCGSNFTSSICIHKWVSGADQSVCSGCRQAFGFTRKRHNC
YNCGLVHCHACSSKKALKAALAPTPGKPHRVCDACYTKLKAGESGYNSNVANRNSTTPTRSLDGTGRPDRDIRSSRILLS
PKTEPVKYSEVRSSRSESSIVRASQVPALQQLRDVAFPSSLSAIQNAFKPVASSSTSTLPSGTRSSRISSPPRSSGFSRG
MIDTLKKSNGVINKEMTKLQSQIKNLKEKCDNQGTEIQRLKKTAREASDLAVKHSSKHKAATEVMKSVAEHLRELKEKLP
PEVSRCEAFESMNSQAEAYLNASEASESCLPTTSLGMGQRDPTPSTNTQDQNIEEKQSSNGGNMRSQEPSGTTEASSSSK
GGGKELIEQFEPGVYVTYVLHKNGGKIFRRVRFSFFVANGDLMSIKQKNGGIAKKIGCLRGIVIMLHHRVQRLLIQFQHQ
LNLNHQLLIQFLHQLNLNHQLLIQFLHQLNLNHQLLLHNRILIKQRIQKKFLMKASHHVYNSSLAK
>NP_001328534.1 Regulator of chromosome condensation (RCC1) family protein [Arabidopsis thaliana]
MADPSSYVNHDRDIDQALVSLKKGTQLLKYSRKGRPKFRSFRLSPDETTLFWLSHGEEKGLKLATVSRILPGQRTAVFRR
YLRPEKDYLSFSLIYHNGDRSLDLICKDKAETEVWFAGLKSLIRQNRNKQAKSEIPEIHDSDCFSTGRPSTASIDFAPNN
TRRGRTSIDLGIQNSPTKFGSSDVGYERGNMLRPSTDGFRISVSSTPSCSTGTSGPDDIESLGDVYVWGEVWSDGISPDG
VVNSTTVKIDVLIPKPLESNVVLDVHQIACGVRHIALVTRQGEVFTWEEEAGGRLGHGIQVDVCRPKLVEFLALTNIDFV
ACGEYHTCAVSTSGDLFTWGDGIHNVGLLGHGSDLSHWIPKRVSGPVEGLQVLSVACGTWHSALATANGKLFTFGDGAFG
VLGHGDRESVSYPKEVKMLSGLKTLKVACGVWHTVAIVEVMNQTGTSTSSRKLFTWGDGDKNRLGHGNKETYLLPTCVSS

LIDYNFNQIACGHTFTVALTTSGHVFTMGGTSHGQLGSSNSDGKLPCLVQDRLVGEFVEEISCGDHHVAVLTSRSEVFTW
GKGSNGRLGHGDKDDRKTPTLVEALRERHVKSISCGSNFTSSICIHKWVSGADQSVCSGCRQAFGFTRKRHNCYNCGLVH
CHACSSKKALKAALAPTPGKPHRVCDACYTKLKAGESGYNSNVANRNSTTPTRSLDGTGRPDRDIRSSRILLSPKTEPVK
YSEVRSSRSESSIVRASQVPALQQLRDVAFPSSLSAIQNAFKPVASSSTSTLPSGTRSSRISSPPRSSGFSRGMIDTLKK
SNGVINKEMTKLQSQIKNLKEKCDNQGTEIQRLKKTAREASDLAVKHSSKHKAATEVMKSVAEHLRELKEKLPPEVSRCE
AFESMNSQAEAYLNASEASESCLPTTSLGMGQRDPTPSTNTQDQNIEEKQSSNGGNMRSQEPSGTTEASSSSKGGGKELI
EQFEPGVYVTYVLHKNGGKIFRRVRFSKRRFDEHQAEEWWNSKKDRLLKRYSHHASSSSPTASDSVPTPTQPQPPASDSV
PTPTQPQPPASDSVPTPTQPQPPASPTQPNSDQAEDSKEVLDES
>NP_001327882.1 Regulator of chromosome condensation (RCC1) family with FYVE zinc finger domain-containing protein [Arabidopsis thaliana]
MADPASCYIYHERDVDQALVVLKKGTQLLKYSRKGKPKFRAFRLSPDEKTLIWFSRGEEKGLKLFEVSRIVPGQRTAVFK
RFLRPEKDHLSFSLLYNNRERSLDLICKDKAETEVWFAALKFLIEKSRNRRARSEIPEIHDSDTFSVGRQSIDFVPSNIP
RGRTSIDLGYQNNSDVGYERGNMLRPSTDGFRISVSSTPSCSSGGSGPDDIESLGDVYVWGEVWTEGILPDGTASNETVK
TDVLTPRPLESNVVLDVHQIVCGVRHVALVTRQGEVFTWGEEVGGRLGHGIQVDISRPKLVEFLALTNIDFVACGEYHTC
VVSTSGDLFSWGDGIHNVGLLGHGSDISHWIPKRVSGPLEGLQVLSVACGTWHSALATANGKLFTFGDGAFGVLGHGNRE
SVSYPKEVQSLNGLKTVKVACSIWHTAAIVEVMGQTATSMSSRKLFTWGDGDKNRLGHGNKETYLLPTCVSSLIDYNFHK
IACGHTFTVALTTSGHVFTMGGTAHGQLGNSISDGKLPCLVQDRLVGEFVEEIACGAHHVAVLTSRSEVFTWGKGANGRL
GHGDTEDKRTPTLVEALRDRHVKSLSCGSNFTSSICIHKWVSGADQSICSGCRQAFGFTRKRHNCYNCGLVHCHACSSKK
ALKAALAPTPGKPHRVCDACYSKLKAAESGYSSNVNRNVATPGRSIDGSVRTDRETTRSSKVLLSANKNSVMSSSRPGFT
PESSNARASQVPSLQQLKDIAFPSSLSAIQNAFKPVVAPTTTPPRTLVIGPSSPSPPPPPRSSSPYARRPSPPRTSGFSR
SVIDSLRKTNEVMNQEMTKLHSQVKNLKQRCNNQGTEIERFQKAAKDASELAARQSSKHKAATEALKSVAEQLKELKEKL
PPEVSESEAFESINSQAEAYLNANKVSETSPLTTSGQEQETYQKTEEQVPSNSSITETSSSSRAPSTEASSSRISGKESK
EQFEPGVYVTYEVDMNGNKIFRRVRFSKKRFDEHQAEDWWTKNKDRLLKCYSSNSSSSSSSSNPTASDSPVAPQPPSDPS
VPEQSNEKEPDSET
>NP_188968.1 Regulator of chromosome condensation (RCC1) family with FYVE zinc finger domain-containing protein [Arabidopsis thaliana]
MADPASCYIYHERDVDQALVVLKKGTQLLKYSRKGKPKFRAFRLSPDEKTLIWFSRGEEKGLKLFEVSRIVPGQRTRFLR
PEKDHLSFSLLYNNRERSLDLICKDKAETEVWFAALKFLIEKSRNRRARSEIPEIHDSDTFSVGRQSIDFVPSNIPRGRT
SIDLGYQNNSDVGYERGNMLRPSTDGFRISVSSTPSCSSGGSGPDDIESLGDVYVWGEVWTEGILPDGTASNETVKTDVL
TPRPLESNVVLDVHQIVCGVRHVALVTRQGEVFTWGEEVGGRLGHGIQVDISRPKLVEFLALTNIDFVACGEYHTCVVST
SGDLFSWGDGIHNVGLLGHGSDISHWIPKRVSGPLEGLQVLSVACGTWHSALATANGKLFTFGDGAFGVLGHGNRESVSY
PKEVQSLNGLKTVKVACSIWHTAAIVEVMGQTATSMSSRKLFTWGDGDKNRLGHGNKETYLLPTCVSSLIDYNFHKIACG
HTFTVALTTSGHVFTMGGTAHGQLGNSISDGKLPCLVQDRLVGEFVEEIACGAHHVAVLTSRSEVFTWGKGANGRLGHGD

TEDKRTPTLVEALRDRHVKSLSCGSNFTSSICIHKWVSGADQSICSGCRQAFGFTRKRHNCYNCGLVHCHACSSKKALKA
ALAPTPGKPHRVCDACYSKLKAAESGYSSNVNRNVATPGRSIDGSVRTDRETTRSSKVLLSANKNSVMSSSRPGFTPESS
NARASQVPSLQQLKDIAFPSSLSAIQNAFKPVVAPTTTPPRTLVIGPSSPSPPPPPRSSSPYARRPSPPRTSGFSRSVID
SLRKTNEVMNQEMTKLHSQQRCNNQGTEIERFQKAAKDASELAARQSSKHKAATEALKSVAEQLKELKEKLPPEVSESEA
FESINSQAEAYLNANKVSETSPLTTSGQEQETYQKTEEQVPSNSSITETSSSSRAPSTEASSSRISGKESKEQFEPGVYV
TYEVDMNGNKIFRRVRFSKKRFDEHQAEDWWTKNKDRLLKCYSSNSSSSSSSSNPTASDSPVAPQPPSDPSVPEQSNEKE
PDSET
>NP_001318728.1 Regulator of chromosome condensation (RCC1) family with FYVE zinc finger domain-containing protein [Arabidopsis thaliana]
MADLVSYGNVVRDIEQALIALKKGAQLLKYGRKGKPKFCPFRLSNDETSLIWISNGGEKRLKLATVSKIVPGQRTAVFQR
YLRPDKDYLSFSLIYSNRKRTLDLICKDKVEAEVWIAGLKALISGQAGRSKIDGWSDGGLSIADSRDLTLSSPTNSSVCA
SRDFNIADSPYNSTNFPRTSRTENSVSSERSHVASDSPNMLVRGTGSDAFRVSVSSVQSSSSHGSAPDDCDALGDVYIWG
EVLCENVTKFGADKNIGYLGSRSDVLIPKPLESNVVLDVHHIACGVKHAALVSRQGEVFTWGEASGGRLGHGMGKDVTGP
QLIESLAATSIDFVACGEFHTCAVTMTGEIYTWGDGTHNAGLLGHGTDVSHWIPKRISGPLEGLQIASVSCGPWHTALIT
STGQLFTFGDGTFGVLGHGDKETVFYPREVESLSGLRTIAVACGVWHAAAIVEVIVTHSSSSVSSGKLFTWGDGDKSRLG
HGDKEPRLKPTCVSALIDHTFHRVACGHSLTVGLTTSGKVYTMGSTVYGQLGNPNADGKLPCLVEDKLTKDCVEEIACGA
YHVAVLTSRNEVFTWGKGANGRLGHGDVEDRKAPTLVDALKERHVKNIACGSNFTAAICLHKWVSGTEQSQCSACRQAFG
FTRKRHNCYNCGLVHCHSCSSKKSLKAALAPNPGKPYRVCDSCHSKLSKVSEANIDSRKNVMPRLSGENKDRLDKTEIRL
AKSGIPSNIDLIKQLDNRAARQGKKADTFSLVRTSQTPLTQLKDALTNVADLRRGPPKPAVTPSSSRPVSPFSRRSSPPR
SVTPIPLNVGLGFSTSIAESLKKTNELLNQEVVRLRAQAESLRHRCEVQEFEVQKSVKKVQEAMSLAAEESAKSEAAKEV
IKSLTAQVKDIAALLPPGAYEAETTRTANLLNGFEQNGFHFTNANGQRQSRSDSMSDTSLASPLAMPARSMNGLWRNSQS
PRNTDASMGELLSEGVRISNGFSEDGRNSRSSAASASNASQVEAEWIEQYEPGVYITLLALGDGTRDLKRVRFSRRRFRE
QQAETWWSENRERVYEKYNIRGTDRSSVATSPISQSQF
>NP_568268.3 Regulator of chromosome condensation (RCC1) family with FYVE zinc finger domain-containing protein [Arabidopsis thaliana]
MSRNGRMASDLSRAGPVERDIEQAIIALKKGAYLLKYGRRGKPKFCPFRLSNDETVLIWFSGNEEKHLKLSHVSRIISGQ
RTPIFQRYPRPEKEYQSFSLIYSERSLDVICKDKDEAEVWFTGLKALISHCHQRNRRTESRSDGTPSEANSPRTYTRRSS
PLHSPFSSNDSLQKDGSNHLRIHSPFESPPKNGLDKAFSDMALYAVPPKGFYPSDSATISVHSGGSDSMHGHMRGMGMDA
FRVSMSSAVSSSSHGSGHDDGDALGDVFIWGEGIGEGVLGGGNRRVGSSFDIKMDSLLPKALESTIVLDVQNIACGGQHA
VLVTKQGESFSWGEESEGRLGHGVDSNIQQPKLIDALNTTNIELVACGEFHSCAVTLSGDLYTWGKGDFGVLGHGNEVSH
WVPKRVNFLLEGIHVSSIACGPYHTAVVTSAGQLFTFGDGTFGVLGHGDKKSVFIPREVDSLKGLRTVRAACGVWHTAAV
VEVMVGSSSSSNCSSGKLFTWGDGDKGRLGHGNKEPKLVPTCVAALVEPNFCQVACGHSLTVALTTSGHVYTMGSPVYGQ
LGNSHADGKTPNRVEGKLHKSFVEEIACGAYHVAVLTSRTEVYTWGKGSNGRLGHGDVDDRNSPTLVESLKDKQVKSIAC

GTNFTAAVCIHRWASGMDQSMCSGCRQPFSFKRKRHNCYNCGLVFCHSCTSKKSLKACMAPNPNKPYRVCDKCFNKLKKT
METDPSSHSSLSRRGSINQGSDPIDKDDKFDSRSDGQLARFSLMESMRQVDSRHKKNKKYEFNSSRVSPIPSGSSQRGAL
NIAKSFNPVFGASKKFFSASVPGSRIVSRATSPISRRPSPPRSTTPTPTLSGLATPKFVVDDTKRTNDNLSQEVVKLRSQ
VESLTRKAQLQEVELERTTKQLKEALAITNEETTRCKAAKEVIKSLTAQLKDMAERLPVGSARTVKSPPSLNSFGSSPGR
IDPFNILNQANSQESEPNGITTPMFSNGTMTPAFGNGEATNEARNEKEWVEQDEPGVYITLTALAGGARDLKRVRFSRKR
FSEIQAEQWWADNRGRVYEQYNVRMVDKASEDLPR
>NP_001322526.1 Regulator of chromosome condensation (RCC1) family with FYVE zinc finger domain-containing protein [Arabidopsis thaliana]
MADSLRTSFSERDVEQAITSLKKGSYLLKYGRRGKPKFCPFQLTSDESALVWYSGKEEKQIKLSQVLRIVPGQRTPTFKR
YPRPEKEYQSFSLICPDRSLDLICKDKDEAEVWVVGLKSLITRVKVSKWKTTIKPEITSAECPTPHARRVSPFVTILDQV
IQPSNETSTQTRLGKVFSDIVAITAPPSNNNQTEASGNLFCPFSPTPANVENSNLRFSTNDPFRLSLSSAVSTSSHGSYH
EDFDALGDVFVWGESISDGVLSGTGNSLNSTTEDALLPKALESTIVLDAQNIACGKCHAVLVTKQGEIFSWGEGKGGKLG
HGLETDAQKPKFISSVRGLGFKSLACGDFHTCAITQSGDLYSWGDGTHNVDLLGHGNESSCWIPKRVTGDLQGLYVSDVA
CGPWHTAVVASSGQLFTFGDGTFGALGHGDRRSTSVPREVESLIGLIVTKVACGVWHTAAVVEVTNEASEAEVDSSRGQV
FTWGDGEKGQLGHGDNDTKLLPECVISLTNENICQVACGHSLTVSRTSRGHVYTMGSTAYGQLGNPTAKGNFPERVEGDI
VEASVEEIACGSYHVAVLTSKSEIYTWGKGLNGQLGHGNVENKREPAVVGFLREKQVKAITCGSNFTAVICVHKWVPGSE
HSLCAGCRNPFNFRRKRHNCYNCGLVFCKVCSSRKSLRAALAPDMNKPYRVCYGCFTKLKKSRESSPSTPTSRTRKLLNM
RKSTDVSERDSLTQKFLSVNARLSSADSSLHYSERRHHRRDLKPEVNNSNVFPSMNGSLQPVGSPFSKGSTALPKIPKNM
MVKIPGSGMSSRTTSPVSVKSTSPRRSYEVAAAESKQLKDSFNQDMAGLKEQVEQLASKAHQLEEELEKTKRQLKVVTAM
AADEAEENRSAKEVIRSLTTQLKEMAEKQSQKDSISTNSKHTDKEKSETVTQTSNQTHIRSMVSQDSQNENNLTSKSFAN
GHRKQNDKPEKVVQDEPGVYLTLLSLPGGGTELKRVRFSRKQFTEEQAEKWWGENGAKVCERHNILVS
>NP_177129.2 Regulator of chromosome condensation (RCC1) family with FYVE zinc finger domain-containing protein [Arabidopsis thaliana]
MCILELRLFAAASMADSLRTSFSERDVEQAITSLKKGSYLLKYGRRGKPKFCPFQLTSDESALVWYSGKEEKQIKLSQVL
RIVPGQRTPTFKRYPRPEKEYQSFSLICPDRSLDLICKDKDEAEVWVVGLKSLITRVKVSKWKTTIKPEITSAECPTPHA
RRVSPFVTILDQVIQPSNETSTQTRLGKVFSDIVAITAPPSNNNQTEASGNLFCPFSPTPANVENSNLRFSTNDPFRLSL
SSAVSTSSHGSYHEDFDALGDVFVWGESISDGVLSGTGNSLNSTTEDALLPKALESTIVLDAQNIACGKCHAVLVTKQGE
IFSWGEGKGGKLGHGLETDAQKPKFISSVRGLGFKSLACGDFHTCAITQSGDLYSWGDGTHNVDLLGHGNESSCWIPKRV
TGDLQGLYVSDVACGPWHTAVVASSGQLFTFGDGTFGALGHGDRRSTSVPREVESLIGLIVTKVACGVWHTAAVVEVTNE
ASEAEVDSSRGQVFTWGDGEKGQLGHGDNDTKLLPECVISLTNENICQVACGHSLTVSRTSRGHVYTMGSTAYGQLGNPT
AKGNFPERVEGDIVEASVEEIACGSYHVAVLTSKSEIYTWGKGLNGQLGHGNVENKREPAVVGFLREKQVKAITCGSNFT
AVICVHKWVPGSEHSLCAGCRNPFNFRRKRHNCYNCGLVFCKVCSSRKSLRAALAPDMNKPYRVCYGCFTKLKKSRESSP
STPTSRTRKLLNMRKSTDVSERDSLTQKFLSVNARLSSADSSLHYSERRHHRRDLKPEVNNSNVFPSMNGSLQPVGSPFS

KGSTALPKIPKNMMVKIPGSGMSSRTTSPVSVKSTSPRRSYEVAAAESKQLKDSFNQDMAGLKEQVEQLASKAHQLEEEL
EKTKRQLKVVTAMAADEAEENRSAKEVIRSLTTQLKEMAEKQSQKDSISTNSKHTDKEKSETVTQTSNQTHIRSMVSQDS
QNENNLTSKSFANGHRKQNDKPEKVVQDEPGVYLTLLSLPGGGTELKRVRFSRKQFTEEQAEKWWGENGAKVCERHNILV
S
>NP_001190338.1 Regulator of chromosome condensation (RCC1) family with FYVE zinc finger domain-containing protein [Arabidopsis thaliana]
MWSGFRDCTRNGRMTPSDLSRAGPVTRDIEQLKIELYSTFGVSKLDSSYILENKNALHAITALKKGAYLLKYGRRGKPKF
CPFRLSNDESVLIWFSGKEEKHLKLSHVSRIISGQRTPIFQRYPRPEKEYQSFSLIYDERSLDLICKDKDEAEVWFSGLK
ALISRCHQRKWRTESRSDGTPSEANSPRTYTRRSSPLHSPFSSNESFQKEGSNHLRLHSPYESPPKNGVDKAFSDMSLYA
VPPKGFFPPGSATMSVHSLSSGGSDTLHGHMKGMGMDAFRVSLSSAISSSSHGSGHDDGDTLGDVFMWGEGIGEGVLGGG
NHRVGSSLEIKMDSLLPKALESTIVLDVQNIACGGQHAVLVTKQGESFSWGEESEGRLGHGVDSNVQHPKLIDALNTTNI
ELVACGEYHSCAVTLSGDLYTWGKGDFGILGHGNEVSHWVPKRVNFLMEGIHVSSIACGPYHTAVVTSAGQLFTFGDGTF
GVLGHGDRKSVFIPREVDSLKGLRTVRAACGVWHTAAVVEVMVGSSSSSNCSSGKLFTWGDGDKSRLGHGDKEPKLVPTC
VAALVEPNFCQVACGHSLTVALTTSGHVYTMGSPVYGQLGNPHADGKVPTRVDGKLHKSFVEEIACGAYHVAVLTSRTEV
YTWGKGSNGRLGHGDADDRNSPTLVESLKDKQVKSIACGSNFTAAVCLHKWASGMDQSMCSGCRQPFNFKRKRHNCYNC
G
LVFCHSCSNKKSLKACMAPNPNKPYRVCDRCFNKLKKAMETDPSSHSSLSRRESVNQGSDAIDRDEKLDTRSDGQLARFS
LLEPMRQVDSRSKKNKKYEFNSSRVSPIPSGGSHRGSLNITKSFNPTFGSSKKFFSASVPGSRIASRATSPISRRPSPPR
STTPTPTLSGLTTPKIVVDDTKRSNDNLSQEVVMLRSQVENLTRKAQLQEVELERTTKQLKEALAIASEESARCKAAKEV
IKSLTAQLKDMAERLPVGSARTVKSPSLNSFGSSPDYAAPSSNTLNRPNSRETDSDSLTTVPMFSNGTSTPVFDSGSYRQ
QANHAAEAINRISTRSKESEPRNENEWVEQDEPGVYITLTALAGGARDLKRVRFSRKRFSEKQAEEWWAENRGRVYEQYN
VRIVVDKSSVGVGSEDLGH
>NP_197443.3 Regulator of chromosome condensation (RCC1) family with FYVE zinc finger domain-containing protein [Arabidopsis thaliana]
MSRNGRMTPSDLSRAGPVTRDIEQAITALKKGAYLLKYGRRGKPKFCPFRLSNDESVLIWFSGKEEKHLKLSHVSRIISG
QRTPIFQRYPRPEKEYQSFSLIYDERSLDLICKDKDEAEVWFSGLKALISRCHQRKWRTESRSDGTPSEANSPRTYTRRS
SPLHSPFSSNESFQKEGSNHLRLHSPYESPPKNGVDKAFSDMSLYAVPPKGFFPPGSATMSVHSLSSGGSDTLHGHMKGM
GMDAFRVSLSSAISSSSHGSGHDDGDTLGDVFMWGEGIGEGVLGGGNHRVGSSLEIKMDSLLPKALESTIVLDVQNIACG
GQHAVLVTKQGESFSWGEESEGRLGHGVDSNVQHPKLIDALNTTNIELVACGEYHSCAVTLSGDLYTWGKGDFGILGHGN
EVSHWVPKRVNFLMEGIHVSSIACGPYHTAVVTSAGQLFTFGDGTFGVLGHGDRKSVFIPREVDSLKGLRTVRAACGVWH
TAAVVEVMVGSSSSSNCSSGKLFTWGDGDKSRLGHGDKEPKLVPTCVAALVEPNFCQVACGHSLTVALTTSGHVYTMGSP
VYGQLGNPHADGKVPTRVDGKLHKSFVEEIACGAYHVAVLTSRTEVYTWGKGSNGRLGHGDADDRNSPTLVESLKDKQV
K

SIACGSNFTAAVCLHKWASGMDQSMCSGCRQPFNFKRKRHNCYNCGLVFCHSCSNKKSLKACMAPNPNKPYRVCDRCFNK
LKKAMETDPSSHSSLSRRESVNQGSDAIDRDEKLDTRSDGQLARFSLLEPMRQVDSRSKKNKKYEFNSSRVSPIPSGGSH
RGSLNITKSFNPTFGSSKKFFSASVPGSRIASRATSPISRRPSPPRSTTPTPTLSGLTTPKIVVDDTKRSNDNLSQEVVM
LRSQVENLTRKAQLQEVELERTTKQLKEALAIASEESARCKAAKEVIKSLTAQLKDMAERLPVGSARTVKSPSLNSFGSS
PDYAAPSSNTLNRPNSRETDSDSLTTVPMFSNGTSTPVFDSGSYRQQANHAAEAINRISTRSKESEPRNENEWVEQDEPG
VYITLTALAGGARDLKRVRFSRKRFSEKQAEEWWAENRGRVYEQYNVRIVVDKSSVGVGSEDLGH
>NP_001330907.1 Regulator of chromosome condensation (RCC1) family with FYVE zinc finger domain-containing protein [Arabidopsis thaliana]
MSRNGRMTPSDLSRAGPVTRDIEQAITALKKGAYLLKYGRRGKPKFCPFRLSNDESVLIWFSGKEEKHLKLSHVSRIISG
QRTPIFQRYPRPEKEYQSFSLIYDERSLDLICKDKDEAEVWFSGLKALISRCHQRKWRTESRSDGTPSEANSPRTYTRRS
SPLHSPFSSNESFQKEGSNHLRLHSPYESPPKNGVDKAFSDMSLYAVPPKGFFPPGSATMSVHSLSSGGSDTLHGHMKGM
GMDAFRVSLSSAISSSSHGSGHDDGDTLGDVFMWGEGIGEGVLGGGNHRVGSSLEIKMDSLLPKALESTIVLDVQNIACG
GQHAVLVTKQGESFSWGEESEGRLGHGVDSNVQHPKLIDALNTTNIELVACGEYHSCAVTLSGDLYTWGKGDFGILGHGN
EVSHWVPKRVNFLMEGIHVSSIACGPYHTAVVTSAGQLFTFGDGTFGVLGHGDRKSVFIPREVDSLKGLRTVRAACGVWH
TAAVVEVMVGSSSSSNCSSGKLFTWGDGDKSRLGHGDKEPKLVPTCVAALVEPNFCQVACGHSLTVALTTSGHVYTMGSP
VYGQLGNPHADGKVPTRVDGKLHKSFVEEIACGAYHVAVLTSRTEVYTWGKGSNGRLGHGDADDRNSPTLVESLKDKQV
K
SIACGSNFTAAVCLHKWASGMDQSMCSGCRQPFNFKRKRHNCYNCGLVFCHSCSNKKSLKACMAPNPNKPYRVCDRCFNK
LKKAMETDPSSHSSLSRRESVNQGSDAIDRDEKLDTRSDGQLARFSLLEPMRQVDSRSKKNKKYEFNSSRVSPIPSGGSH
RGSLNITKSFNPTFGSSKKFFSASVPGSRIASRATSPISRRPSPPRSTTPTPTLSGLTTPKIVVDDTKRSNDNLSQEVVM
LRSQVSCFPRFNSCSLLLCI
>NP_565144.1 Regulator of chromosome condensation (RCC1) family with FYVE zinc finger domain-containing protein [Arabidopsis thaliana]
MADLVTYSNADHNLEQALITLKKGTQLLKYGRKGKPKFYPFRLSSDEKSLIWISSSGEKRLKLASVSKIVPGQRTAVFQR
YLRPEKDYLSFSLLYNGKKKSLDLICKDKVEAEIWIGGLKTLISTGQGGRSKIDGWSGGGLSVDASRELTSSSPSSSSAS
ASRGHSSPGTPFNIDPITSPKSAEPEVPPTDSEKSHVALDNKNMQTKVSGSDGFRVSVSSAQSSSSHGSAADDSDALGDV
YIWGEVICDNVVKVGIDKNASYLTTRTDVLVPKPLESNIVLDVHQIACGVRHAAFVTRQGEIFTWGEESGGRLGHGIGKD
VFHPRLVESLTATSSVDFVACGEFHTCAVTLAGELYTWGDGTHNVGLLGHGSDISHWIPKRIAGSLEGLHVASVSCGPWH
TALITSYGRLFTFGDGTFGVLGHGDKETVQYPREVESLSGLRTIAVSCGVWHTAAVVEIIVTQSNSSSVSSGKLFTWGDG
DKNRLGHGDKDPRLKPTCVPALIDYNFHKIACGHSLTVGLTTSGQVFTMGSTVYGQLGNLQTDGKLPCLVEDKLASEFVE
EISCGAYHVAALTSRNEVYTWGKGANGRLGHGDLEDRKVPTIVEALKDRHVKYIACGSNYTAAICLHKWVSGAEQSQCST
CRLAFGFTRKRHNCYNCGLVHCHSCSSKKAFRAALAPSAGRLYRVCDSCYVKLSKVSEINDTNRRNSAVPRLSGENRDRL
DKSEIRLAKFGTSNMDLIKQLDSKAAKQGKKTDTFSLGRNSQLPSLLQLKDAVQSNIGDMRRATPKLAQAPSGISSRSVS

PFSRRSSPPRSATPMPSTSGLYFPVGIADNMKKTNEILNQEIVKLRTQVDSLTQKCEFQEVELQNSVKKTQEALALAEEE
SAKSRAAKEAIKSLIAQLKDVAEKLPPGESVKLACLQNGLDQNGFHFPEENGFHPSRSESMTSSISSVAPFDFAFANASW
SNLQSPKQTPRASERNSNAYPADPRLSSSGSVISERIEPFQFQNNSDNGSSQTGVNNTNQVEAEWIEQYEPGVYITLVAL
HDGTRDLRRVRFSRRRFGEHQAETWWSENREKVYEKYNVRVSEKSTASQTHRDRDEEEEDIPH
>NP_001320309.1 Regulator of chromosome condensation (RCC1) family with FYVE zinc finger domain-containing protein [Arabidopsis thaliana]
MDEKYLIWYSGEEERQLRLSSVITIVRGQITPNFQKQAQSDRKEQSFSLIYANGEHTLDLICKDKAQADSWFKGLRAVIT
KHHNIRNSVNHRSSRGAQSCINSPAGFMRRKQNLGLLEETPDVTQIRSLCGSPSTLLEERCLSNGLSCSSDSFAESDALG
PVSSYYETDYDFRNSDCDRSTGSELCRFSSQRFAASPPLSIITQPVTRSNVLKDIMIWGAITGLIDGSKNQNDALSPKLL
ESATMFDVQSISLGAKHAALVTRQGEVFCWGNGNSGKLGLKVNIDIDHPKRVESLEDVAVRSVACSDHQTCAVTESGELY
LWGIDGGTIEQSGSQFLTRKISDVLGGSLTVLSVACGAWHTAIVTSSGQLFTYGSGTFGVLGHGSLESVTKPKEVESLRR
MKVISVSCGPWHTAAIVETANDRKFYNAKSCGKLFTWGDGDKGRLGHADSKRKLVPTCVTELIDHDFIKVSCGWTLTVAL
SISGTVYTMGSSIHGQLGCPRAKDKSVNVVLGNLTRQFVKDIASGSHHVAVLTSFGNVYTWGKGMNGQLGLGDVRDRNSP
VLVEPLGDRLVESIACGLNLTAAICLHKEISLNDQTACSSCKSAFGFTRRKHNCYNCGLLFCNACSSKKAVNASLAPNKS
KLSRVCDSCFDHLWSITEFSRNVKMDNHTPRMQMVTRRVSEDLTEKQSENEMQNLPQANRSSDGQPRWGQVSGPSLFRFD
KISTSSSLNLSVSARRTSSTKISTSSESNKILTEEIERLKAVIKNLQRQCELGNEKMEECQQELDKTWEVAKEEAEKSKA
AKEIIKALASKLQANKEKPSNPLKTGIACNPSQVSPIFDDSMSIPYLTPITTARSQHETKQHVEKCVTKSSNRDSNIKLL
VDASPAITRTGYLQNETQDSSAEQVEQYEPGVYITFTALPCGQKTLKRVRFSRKRFSEKEAQRWWEEKQVLVYNKYDAEI
>NP_176767.1 Regulator of chromosome condensation (RCC1) family with FYVE zinc finger domain-containing protein [Arabidopsis thaliana]
MGEQQISVTVPRDRTDEQAILALKKGAQLLKCRRRGNPKFCPFKLSMDEKYLIWYSGEEERQLRLSSVITIVRGQITPNF
QKQAQSDRKEQSFSLIYANGEHTLDLICKDKAQADSWFKGLRAVITKHHNIRNSVNHRSSRGAQSCINSPAGFMRRKQNL
GLLEETPDVTQIRSLCGSPSTLLEERCLSNGLSCSSDSFAESDALGPVSSYYETDYDFRNSDCDRSTGSELCRFSSQRFA
ASPPLSIITQPVTRSNVLKDIMIWGAITGLIDGSKNQNDALSPKLLESATMFDVQSISLGAKHAALVTRQGEVFCWGNGN
SGKLGLKVNIDIDHPKRVESLEDVAVRSVACSDHQTCAVTESGELYLWGIDGGTIEQSGSQFLTRKISDVLGGSLTVLSV
ACGAWHTAIVTSSGQLFTYGSGTFGVLGHGSLESVTKPKEVESLRRMKVISVSCGPWHTAAIVETANDRKFYNAKSCGKL
FTWGDGDKGRLGHADSKRKLVPTCVTELIDHDFIKVSCGWTLTVALSISGTVYTMGSSIHGQLGCPRAKDKSVNVVLGNL
TRQFVKDIASGSHHVAVLTSFGNVYTWGKGMNGQLGLGDVRDRNSPVLVEPLGDRLVESIACGLNLTAAICLHKEISLND
QTACSSCKSAFGFTRRKHNCYNCGLLFCNACSSKKAVNASLAPNKSKLSRVCDSCFDHLWSITEFSRNVKMDNHTPRMQM
VTRRVSEDLTEKQSENEMQNLPQANRSSDGQPRWGQVSGPSLFRFDKISTSSSLNLSVSARRTSSTKISTSSESNKILTE
EIERLKAVIKNLQRQCELGNEKMEECQQELDKTWEVAKEEAEKSKAAKEIIKALASKLQANKEKPSNPLKTGIACNPSQV
SPIFDDSMSIPYLTPITTARSQHETKQHVEKCVTKSSNRDSNIKLLVDASPAITRTGYLQNETQDSSAEQVEQYEPGVYI
TFTALPCGQKTLKRVRFSRKRFSEKEAQRWWEEKQVLVYNKYDAEI

>NP_196834.3 ARF GTPase-activating protein [Arabidopsis thaliana]
MHFTKLDDSPMFRKQLQSMEESAEILRERSLKFYKGCRKYTEGLGEAYDGDIAFASALETFGGGHNDPISVAFGGPVMTK
FTIALREIGTYKEVLRSQVEHILNDRLLQFANMDLHEVKEARKRFDKASLTYDQAREKFLSLRKGTKSDVAAALEQELHT
SRSMFEQARFNLVTALSNVEAKKRFEFLEAVSGTMDAHLRYFKQGYELLHQMEPYINQVLTYAQQSRERSNYEQAALNEK
MQEYKRQVDRESRWGSNGSNGSPNGDGIQAIGRSSHKMIDAVMQSAARGKVQTIRQGYLSKRSSNLRGDWKRRFFVLDSR
GMLYYYRKQCSKPSGSGSQLSGQRNSSELGSGLLSRWLSSNNHGHGGVHDEKSVARHTVNLLTSTIKVDADQSDLRFCFR
IISPTKNYTLQAESALDQMDWIEKITGVIASLLSSQVPEQRLPGSPMGSGHHRSASESSSYESSEYDHPTTEEFVCERSF
LGYNERPSRSFQPQRSIRKGEKPIDALRKVCGNDKCADCGAPEPDWASLNLGVLVCIECSGVHRNLGVHISKVRSLTLDV
KVWEPSVISLFQALGNTFANTVWEELLHSRSAIHFDPGLTVSDKSRVMVTGKPSYADMISIKEKYIQAKYAEKLFVRRSR
DSDFPQSAAQQMWDAVSGNDKKAVYRLIVNGDADVNYVYDQTSSSSLTLSRVILVPERPKREDVLLRLRNELLDRTGSSS
NISPEGSGGSSLLHCACEKADLGMVELLLQYGANVNASDSSGQTPLHCCLLRGKVTIARLLLTRGADPEAMNREGKTALD
IAAESNFTDPEVLALLSDTNGYNHRQC

Domains Architecture of Drosophila melanogaster

No	***Accession Number***	***Domains***
1	NP_001284784.1	FYVE zinc finger, PH domain
2	NP_524729.2	Domain of unknown function (DUF3480), FYVE zinc finger, Smad anchor for receptor activation (SARA)
3	NP_001287560.1	RUN domain, FYVE zinc finger
4	NP_733203.1	RUN domain, FYVE zinc finger
5	NP_001287556.1	FYVE zinc finger

6	NP_001097943.1	FYVE zinc finger
7	NP_525099.3	Hepatocyte growth factor-regulated tyrosine kinase substrate, VHS domain , FYVE zinc finger
8	NP_722831.1	Hepatocyte growth factor-regulated tyrosine kinase substrate, FYVE zinc finger
9	NP_001096997.1	Myotubularin-like phosphatase domain, FYVE zinc finger
10	NP_001096996.1	Myotubularin-like phosphatase domain, FYVE zinc finger
11	NP_608968.2	Beige/BEACH domain, FYVE zinc finger , PH domain associated with Beige/BEACH WD domain, G-beta repeat
12	NP_001104451.1	Ankyrin repeats (3 copies), FYVE zinc finger, BTB/POZ domain
13	NP_001015359.1	Ankyrin repeats (3 copies), FYVE zinc finger, BTB/POZ domain
14	NP_001015360.1	Ankyrin repeats (3 copies), FYVE zinc finger, BTB/POZ domain
15	NP_730882.2	RhoGEF domain, FERM C-terminal PH-like domain, FERM N-terminal domain, FERM central domain, PH domain, FERM adjacent (FA)
16	NP_001036676.1	RhoGEF domain, FERM C-terminal PH-like domain, FERM N-terminal domain, FERM central domain, PH domain, FERM adjacent (FA)

17	NP_609186.1	FYVE zinc finger, Rabenosyn Rab binding domain
18	NP_611545.1	Rabaptin-like protein, FYVE zinc finger
19	NP_611269.1	Phosphatidylinositol-4-phosphate 5-Kinase, TCP-1/cpn60 chaperonin family, FYVE zinc finger
20	NP_731570.2	FYVE zinc finger
21	NP_001188779.1	WD domain, G-beta repeat, WD domain, G-beta repeat, FYVE zinc finger
22	NP_611791.2	Myotubularin-like phosphatase domain , FYVE zinc finger
23	NP_726322.1	Myotubularin-like phosphatase domain, FYVE zinc finger
24	NP_001097433.1	Myotubularin-like phosphatase domain, FYVE zinc finger
25	NP_647623.1	Zinc finger, C3HC4 type (RING finger)

Retrieved Sequences of Drosophila melanogaster

>NP_001284784.1 rush hour, isoform B [Drosophila melanogaster]
MVDRLVNSEANTRRIASVENCFGSSGVPLAMQGRVLVGEGVLTKMCRKRPKSRQFFLFNDILVYGNIVIGKKKYNKQHIM
PLEEVSLESIADNQTYRNGWYIRTTTKSFVVFAATSTEKQEWMAHINKCVEDLLRKSGKKPVENHAAVWVPDTDASVCMH
CKKTQFTFIQRRHHCRNCGAVVCAGCSAKKFLLPQQSTKALRVCDACYERLKHVPSSLGSGEDSAAATGAASGNKLNTTA

GDSSNDEDSDEETASPGGESHDEPRFYGDNSVLSAVEDSSTITSPSSATTGSLEAPQVTPSVQSSPAAVATTGSHC
>NP_524729.2 smad anchor for receptor activation [Drosophila melanogaster]
MDLVDIDQVLDNLELSIAADEQLSRNAAAAAAAGANPGRFLGDGASASISGVGAGAGVSGAAGVTPTPASGKSFVGVSQV
FNSLDDYRENVKSLQVDAQLPSYDWQTEAEHEDDIHKFSEQASKNNGQTIVSSSESLTTSSCSTFSSGPSPVSNGSHSEE
LATPPEDEKTSEVQVEPKEQDQQPEQVKHEDQHQDQAVPPPTQSKQTVEELAVGLSSISITASGTESQSLLPEEVTASSV
LGQVLEELSEESSPSREPETEMTNGIDVPLANLPASVAEVPVPKEMEQPVQTEEEDVELPQEQHPRRSQPLTFCSTMDEI
SDTELDSMLQEMDADDNQADDQQMPDDEKSCSASPVTEEESVRILSDEQETTSNDQMNVDNFSQASTVEFADLKQPESTP
VTAMGEDVASSFSSTESDSLSGRKLDGEVEVDREHEDASLATDALETQEQRPQRPQTLNLNGCQAESSTPSQDENPEIQQ
ETLQPTEVREGAAGVGVIPGEDDEESPIYEAVGYSDPHANLGKVPPIWVPDNMAGQCMQCQQKFTMIKRRHHCRACGKVL
CSVCCSQRFRLEFATEPESRVCVQCYMILSERQANGLNSESAPGSALPPTPIRSPNPNNPMEYCSTIPPHRQVANSPGAP
PPSVIVPVGVLKKTDGSSSNSSSSDGQKGQRKRKSVMFSDGIAPGSELASTMEQQWGESKHSRRGLSRSGSGAGQKPPTP
ATEEPPRSIDTSSTLGLVAQLFRGSIPPSAAAATLSATETSAGRSSSQSQAQTSPRRKAESSRNRKLPPNGDAQGCYLPP
EENGLPPICISSKEGGEVDYKVVRNDGSLLDRLQNETLKFIIKNNFFVLVKIVNMSCCMNRWVLNFTTSGLHHMGSDELI
ILLEIKQPKQEEYVKGEVAIPKDLFQHLYEIYVEAEHARSGTHELAFTSPRNANFLGSREHGGFIFIRPTYQCLQGVIVP
DNPYLVGVLIHRHEVPWAKVLPLRLILRLGAQYRYYPCPLISVRGRESVYGEIAQTIINFLVDFRNYSYSLPAIRGLYIH
MEDRQTTVIIPKYRQQDVIKAINNASDHILAFAGNFSKVADGHLVCMQNINDDRSEMYSYSTQAINIQGQPRKITGASFF
VLNEALKSSSGLSGKCSIVEDGLMVQIMPAKMEEVRQALRNQNDIDIVCGPIDATDDQSEIVSIKWVDNNRDINVGVKSP
IDDQPMDGISNMRVLKWASFNYSNTNYAIRLSDIYIIKCEDWYATNGGNCEEITRIAEQLARSASMALLPLLDLLAAAGI
NKLGLRATLDQDNVGYEAGARGSKLPPLYMNALDNHLIATLHGESSGIQEPIILELIFYILNA
>NP_001287560.1 uncharacterized protein Dmel_CG31064, isoform G [Drosophila melanogaster]
MRGLVEDTKRTKLSSANNNNLSSTSGSGRIKSSSLATGSSASGSSSKPLRSAVKTPTSGSATSLSSGTMAAKKAPQTGQQ
QVGKSAKSATAATPTGTTATAIGATATTRAQAAAAKSQKGQNPPSQQVQPKQQPPQPQQTQQQPLVLQQQQASNSSNTIA
LPKASHANTSEELAGGVQDTIYLCNFRVSVDGEWLCLKELQDIDVAGGTGNQAAQTSEKLAGGSSSQTGGFGSNYNVGSS
SSKRNSKRFSGLSTGSGSGGNAGGGGGLDITDNARDTAEIERSNLVNICKLVVKELLEQSLRYGRMLDSDHLPLQHFFIV
IEHVLGHGLRPKKGLLGPRKELWDLLQSVEHYCPEAQDITASVRDLPTVRTHIGRARAWLRIALMQKKLSDYLQALIEHR
EDSLFDYYEPHALMMSDEIVVIMGILVGLNVIDCNLCVKEEDLDSQQGVIDFSLYLRSSSRNADAAPEDSAPPALLDATG
QGNMIAVLDQKNYIEELNRHLNATVGNLQAKVESLTTTNALMKEDLAIARNSLLALQAENQAMRQSTSAGDNNSTGSGGS
SDKDKEKASEELVEERRKNSELEKELKLQVSLKAESDMAMKLLEKDIHEKQDTIVSLRRQLDDIKQINLEMYRKLQDCKA
SLTHKTELMTKLEVQKEDMASTIDQLEKKWSHDKSNLGEILKTTSQTLTTQVTASEERAARAEAESRIEREWRISLQEKE
LKLKEKIANLQGCLKELSEEKERNGKLKADLDKVRTQWSEAQTTLEELGIQLSVSKLKVSEMQDQERRQRQLLSGSAQSL
QAMPEAVGSPGIWAPDSIATHCTACEREFNLTRRKHHCRSCGEIFCKACSEHTLPLLNAQGQPGKPVRVCDNCYAAK
>NP_733203.1 uncharacterized protein Dmel_CG31064, isoform B [Drosophila melanogaster]
MSTDDLLVRRERSEDSQSSSVSIPVTPTTGGAAPAPSAPTSSLDRLRNTFRRTESISSQIFSHISTQFSNAAAAAASSEV

IDGTTTYPYPDEPAPPVVPVAPDTVASASASSPSPSKLKAKRLKRARDTAEIERSNLVNICKLVVKELLEQSLRYGRMLD
SDHLPLQHFFIVIEHVLGHGLRPKKGLLGPRKELWDLLQSVEHYCPEAQDITASVRDLPTVRTHIGRARAWLRIALMQKK
LSDYLQALIEHREDSLFDYYEPHALMMSDEIVVIMGILVGLNVIDCNLCVKEEDLDSQQGVIDFSLYLRSSSRNADAAPE
DSAPPALLDATGQGNMIAVLDQKNYIEELNRHLNATVGNLQAKVESLTTTNALMKEDLAIARNSLLALQAENQAMRQSTS
AGDNNSTGSGGSSDKDKEKASEELVEERRKNSELEKELKLQVSLKAESDMAMKLLEKDIHEKQDTIVSLRRQLDDIKQIN
LEMYRKLQDCKASLTHKTELMTKLEVQKEDMASTIDQLEKKWSHDKSNLGEILKTTSQTLTTQVTASEERAARAEAESRI
EREWRISLQEKELKLKEKIANLQGCLKELSEEKERNGKLKADLDKVRTQWSEAQTTLEELGIQLSVSKLKVSEMQDQERR
QRQLLSGSAQSLQAMPEAVGSPGIWAPDSIATHCTACEREFNLTRRKHHCRSCGEIFCKACSEHTLPLLNAQGQPGKPVR
VCDNCYAAK
>NP_001287556.1 uncharacterized protein Dmel_CG6051, isoform D [Drosophila melanogaster]
MDTFKRWLNKPKADDKSLLARFFHADRSLTAVASELDSFDGRAEPDRCTRLVSRLRQNQDKVLAITNLIMEELLGEDRDP
RAFRAKFPEEVLQENLAGQLWFGAECLAAGSSIMNRETESKEMRPLAQAVTKSLGNVRVLLRDQCLKNNVPNSKTLHLDL
NDSTTEQLYESLKIFDRLFAEFELSYVSAMVQVKSRHEYEMQQWIGVLFSETLQRALKIGLLDQEMVDAFDPGLMFSIPR
LAIVAGLVVYAKGPLNMDMPGDQLSEMFRPFRTILIKIRDLLRNLNNQELYQLEKLLCTNEDINTKVPLGSSSIEAPSPE
HSSHPTTSSSQNNNNSSNNNHSSSSTNTTSTTTTTAGTTNTHRTVERLVDQRNNNHNSNSNSSTNPTVEAATLRSPSMLS
LSATSTPTASPAPSPTPSHSIASTSSAATSSTNPPADWSDGDDEDEDDDDIDVDEEDIESSDDGTDEEQLLKDIVSADCA
SGYLIPNTNLGNLLQPQEVPLTDNFVASEDDEYGTAEQQGHQGLEEDEPSTSAAMLAATRTLQRLRLPSSDNEPLAEPTT
IKASEEHMQQPSGRHRDSHSHRHHQRHHHHHHHRHPHQHQHRQPHPHRTTRSGRKRCSLEAADPERIQPDREQNLASGDT
SAASSLSDDVSLAMRNTTARLKFNTENLLHRLFVCIAGVADQLQTNFASDLRQILRSVFLMNMSTAQEEIDIPEKTKESE
LFEFRASENDVIQESAGSNQSIYSAEEVNPELDNVFSAGGGNQATGQRHSAGASMQRNNTIDLASQPGEGSPSGATTATS
RSHVTRSRSLGDQEAASSATSSTAQLRQLEQQQQQQQLQIQLQRQRNNSVGSNTPSSASSTSSSSEQNSPVSARSGSRRR
LQSNNETQMPSSATSTSATLSPPAWIPDGKAPRCMACQTPFTAFRRRHHCRNCGGVFCGVCSNASAPLPKYGLTKAVRVC
RDCYVREVRSGMGVQGVQSVQSVQASAS
>NP_001097943.1 uncharacterized protein Dmel_CG6051, isoform B [Drosophila melanogaster]
MDTFKRWLNKPKADDKSLLARFFHADRSLTAVASELDSFDGRAEPDRCTRLVSRLRQNQDKVLAITNLIMEELLGEDRDP
RAFRAKFPEEVLQENLAGQLWFGAECLAAGSSIMNRETESKEMRPLAQAVTKSLGNVRVLLRDQCLKNNVPNSKTLHLDL
NDSTTEQLYESLKIFDRLFAEFELSYVSAMVQVKSRHEYEMQQWIGVLFSETLQRALKIGLLDQEMVDAFDPGLMFSIPR
LAIVAGLVVYAKGPLNMDMPGDQLSEMFRPFRTILIKIRDLLRNLNNQELYQLEKLLCTNEDINTKVPLGSSSIEAPSPE
HSSHPTTSSSQNNNNSSNNNHSSSSTNTTSTTTTTAGTTNTHRTVERLVDQRNNNHNSNSNSSTNPTVEAATLRSPSMLS
LSATSTPTASPAPSPTPSHSIASTSSAATSSTNPPADWSDGDDEDEDDDDIDVDEEDIESSDDGTDEEQLLKDIVSADCA
SGYLIPNTNLGNLLQPQEVPLTDNFVASEDDEYGTAEQQGHQGLEEDEPSTSAAMLAATRTLQRLRLPSSDNEPLAEPTT
IKASEEHMQQPSGRHRDSHSHRHHQRHHHHHHHRHPHQHQHRQPHPHRTTRSGRKRCSLEAADPERIQPDREQNLASGDT
SAASSLSDDVSLAMRNTTARLKFKSTENLLHRLFVCIAGVADQLQTNFASDLRQILRSVFLMNMSTAQEEIDIPEKTKES

ELFEFRASENDVIQESAGSNQSIYSAEEVNPELDNVFSAGGGNQATGQRHSAGASMQRNNTIDLASQPGEGSPSGATTAT
SRSHVTRSRSLGDQEAASSATSSTAQLRQLEQQQQQQLQIQLQRQRNNSVGSNTPSSASSTSSSSEQNSPVSARSGSRR
RLQSNNETQMPSSATSTSATLSPPAWIPDGKAPRCMACQTPFTAFRRRHHCRNCGGVFCGVCSNASAPLPKYGLTKAVRV
CRDCYVREVRSGMGVQGVQSVQSVQASAS
>NP_525099.3 hepatocyte growth factor regulated tyrosine kinase substrate, isoform B [Drosophila melanogaster]
MFRSSFDKNLENATSHLRLEPDWPSILLICDEINQKDVTPKNAFAAIKKKMNSPNPHSSCYSLLVLESIVKNCGAPVHEE
VFTKENCEMFSSFLESTPHENVRQKMLELVQTWAYAFRSSDKYQAIKDTMTILKAKGHTFPELREADAMFTADTAPNWAD
GRVCHRCRVEFTFTNRKHHCRNCGQVFCGQCTAKQCPLPKYGIEKEVRVCDGCFAALQRPTSGSGGAKSGPRPADSELPA
EYLNSTLAQQVQTPARKTEQELKEEEELQLALALSQSEAEQQKPKLQSLPPAAYRMQQRSPSPEAPPEPKEYHQQPEEAT
NPELAKYLNRSYWEQRKISESSSMASPSAPSPMPPTPQPQQIMPLQVKSADEVQIDEFAANMRTQVEIFVNRMKSNSSRG
RSISNDSSVQTLFMTLTSLHSQQLSYIKEMDDKRMWYEQLQDKLTQIKDSRAALDQLRQEHVEKLRRIAEEQERQRQMQM
AQKLDIMRKKKQEYLQYQRQLALQRIQEQEREMQLRQEQQKAQYLMGQSAPPFPYMPPSAVPQHGSPSHQLNNVYNPYAA
GVPGYLPQGPAPAPNGHGQFQAIPPGMYNPAIQQPMPPNLQPGGLMQQPAPPGNPQMMPPMPENQFANNPAAILQLPQQH
SIAQPPQIPFQPQPQQIPGQQPQQIPGQQPQQIPGQQPQQIPGQQPQQIPVQQPQPQPQMGHVMLQQHQAPPAAQAPPVT
EIANNQVQAVAAAPAPPQNEPGPAPVKAEEPATAELISFD
>NP_722831.1 hepatocyte growth factor regulated tyrosine kinase substrate, isoform A [Drosophila melanogaster]
MSSEEELSIEEESLDTMTILKAKGHTFPELREADAMFTADTAPNWADGRVCHRCRVEFTFTNRKHHCRNCGQVFCGQCTA
KQCPLPKYGIEKEVRVCDGCFAALQRPTSGSGGAKSGPRPADSELPAEYLNSTLAQQVQTPARKTEQELKEEEELQLALA
LSQSEAEQQKPKLQSLPPAAYRMQQRSPSPEAPPEPKEYHQQPEEATNPELAKYLNRSYWEQRKISESSSMASPSAPSPM
PPTPQPQQIMPLQVKSADEVQIDEFAANMRTQVEIFVNRMKSNSSRGRSISNDSSVQTLFMTLTSLHSQQLSYIKEMDDK
RMWYEQLQDKLTQIKDSRAALDQLRQEHVEKLRRIAEEQERQRQMQMAQKLDIMRKKKQEYLQYQRQLALQRIQEQERE
M
QLRQEQQKAQYLMGQSAPPFPYMPPSAVPQHGSPSHQLNNVYNPYAAGVPGYLPQGPAPAPNGHGQFQAIPPGMYNPAIQ
QPMPPNLQPGGLMQQPAPPGNPQMMPPMPENQFANNPAAILQLPQQHSIAQPPQIPFQPQPQQIPGQQPQQIPGQQPQQI
PGQQPQQIPGQQPQQIPVQQPQPQPQMGHVMLQQHQAPPAAQAPPVTEIANNQVQAVAAAPAPPQNEPGPAPVKAEEPAT
AELISFD
>NP_001096997.1 uncharacterized protein Dmel_CG3632, isoform F [Drosophila melanogaster]
MSDGSPPPSICFIRAAESYPKSQMEKEDSQLFVPFQELAGESIKYLGRTDDGILALSNYRIFLSKQSTGYETYVPLGLIE
SVQVRDLFQLIVNCKDASTVRCSFPSAEQCSDWQRRIHLMIGVPESLETLFAFPFYSWTCDVLGSGNGSGIVSAQANGNG
LIAKDRCLTLHNGLAKPTANVTASNPLPDPASETISAAMSNRLQRSVRYESDFKNEVARLGFDLKGSWRISTANADFKLC
PSYPPKLLVPCCITDEMLHNVANFRGSRRLPAVVWRHQKSGAILARCSQPEVGWLGWRNTRDEQLLKALADACAFDRGEH
ARHSTCANAKAQPTKGSKETNGQSGLSGKSSPSLDDSSHEELTLDEIKKILIVDARSYTSAVTNRARGGGCECIEYYPCA
EIEFMNLGNIHAIRKSFHAVRQLCASSPDDPNWYGQLEKTMWMQHLSGLLGATMTVVHTIEKNGRPVLVHCSDGWDRTPQ

IVATAQLCLDPYYRTVEGFRVLVEREWLNFGHKFADRSGNGPNSDEVNERCPVFLQWLDLVHQIHRQYPCSFEFSISYLI
KLAQHSLSCLFGTFLCNSLRERIENSVFDRTFSVWPFLAETMYRNPLYKHETEKVLWPAHSVRFLYFWSDVYLGSLGNKN
GTDLPLLSNERQSGHNGLMAKTRSSEDLTTNELGQSTISRRSSDPNLTVESIVTDCFNANSNSMFDIRSEANNSVSENVQ
ESVKDSKEVELDVTDATEVGPCGRSNNPIPDFHNDQSTASKRDILEVESQTQRRSSLESSQLIPGSVFIFGSSENDKNAV
PSESEESLATSQKLNSESGRPFFIFGSSENDQSPVPLKSEDTSDISRALWHGAIETSTDTLIPAEPVQKPNSDNSSRDHI
TSPTMELVSGDRKLESTSTVQASKISQSYNNLPKVHIRPNAVICPQRVDAFPHHLDLQVPRETTGNPDRCKPEKLAKDAN
ANANANGSLLASDGYNGEESLFISPDLQRFVGQSPFDAPSTGGRIRRNTFGSSYSRDMKFTAENGSAHQFPSSGIISLPP
TPFQERAQFTISCPDGLAHGLSEQNIRLHQIVQEHKLREEMLLREIHGMRLALLEKGCPSCNSIVSTNMEHENGSDIVEN
ASTCSWEAVEERSGPASYAPSSIQEKKASSVLWVPDHAVSRCSSCQTEFWLGRRKHHCRSCGEIFCADCSEFWAPLPNEK
LFNPVRLCGSCYTSVTTNVQEYAAVPAESQAKDDETSSAIS
>NP_001096996.1 uncharacterized protein Dmel_CG3632, isoform E [Drosophila melanogaster]
MDFMVPIHTMSDGSPPPSICFIRAAESYPKSQMEKEDSQLFVPFQELAGESIKYLGRTDDGILALSNYRIFLSKQSTGYE
TYVPLGLIESVQVRDLFQLIVNCKDASTVRCSFPSAEQCSDWQRRIHLMIGVPESLETLFAFPFYSWTCDVLGSGNGSGI
VSAQANGNGLIAKDRCLTLHNGLAKPTANVTASNPLPDPASETISAAMSNRLQRSVRYESDFKNEVARLGFDLKGSWRIS
TANADFKLCPSYPPKLLVPCCITDEMLHNVANFRGSRRLPAVVWRHQKSGAILARCSQPEVGWLGWRNTRDEQLLKALAD
ACAFDRGEHARHSTCANAKAQPTKGSKETNGQSGLSGKSSPSLDDSSHEELTLDEIKKILIVDARSYTSAVTNRARGGGC
ECIEYYPCAEIEFMNLGNIHAIRKSFHAVRQLCASSPDDPNWYGQLEKTMWMQHLSGLLGATMTVVHTIEKNGRPVLVHC
SDGWDRTPQIVATAQLCLDPYYRTVEGFRVLVEREWLNFGHKFADRSGNGPNSDEVNERCPVFLQWLDLVHQIHRQYPCS
FEFSISYLIKLAQHSLSCLFGTFLCNSLRERIENSVFDRTFSVWPFLAETMYRNPLYKHETEKVLWPAHSVRFLYFWSDV
YLGSLGNKNGTDLPLLSNERQSGHNGLMAKTRSSEDLTTNELGQSTISRRSSDPNLTVESIVTDCFNANSNSMFDIRSEA
NNSVSENVQESVKDSKEVELDVTDATEVGPCGRSNNPIPDFHNDQSTASKRDILEVESQTQRRSSLESSQLIPGSVFIFG
SSENDKNAVPSESEESLATSQKLNSESGRPFFIFGSSENDQSPVPLKSEDTSDISRALWHGAIETSTDTLIPAEPVQKPN
SDNSSRDHITSPTMELVSGDRKLESTSTVQASKISQSYNNLPKVHIRPNAVICPQRVDAFPHHLDLQVPRETTGNPDRCK
PEKLAKDANANANANGSLLASDGYNGEESLFISPDLQRFVGQSPFDAPSTGGRIRRNTFGSSYSRDMKFTAENGSAHQFP
SSGIISLPPTPFQERAQFTISCPDGLAHGLSEQNIRLHQIVQEHKLREEMLLREIHGMRLALLEKGCPSCNSIVSTNMEH
ENGSDIVENASTCSWEAVEERSGPASYAPSSIQEKKASSVLWVPDHAVSRCSSCQTEFWLGRRKHHCRSCGEIFCADCSE
FWAPLPNEKLFNPVRLCGSCYTSVTTNVQEYAAVPAESQAKDDETSSAIS
>NP_608968.2 blue cheese [Drosophila melanogaster]
MNVMRKLRGAASAGSVSGSSSGTSTANNSSPGSSRTDAGAPTAANGRNAEEALMDARVQVSLTTLKKLFNEYTHPREPLS
EQERDDKLYEMLPLFCKVFSSCPSNDMSEKFWDVVAFCQQVSRLMVSEIRKRASNQSTEAASIAIVKFLEVETTEETSSG
WMLLATLNLLANGDVSLIQVMTAAAVPSTLVKCLYLFFDLPIVEDDEPSADGGAVSEFNAHERRTLLQKVFVQLLVKLCS
YPYPAEELARMDDLTLLFSAITSPCPIHNIVWRKNAAEILTTISRNGLTDAVVSYIHSKGCMALCVDNMQRLTFGNPLEI
VEMFVTVFCFLKDSSQVSQILMDDFRASQGYVFLSDFLLKFDNNRSQSLEIQAAIRNLVLMISSLCMCGFYELRPPASQF

NTAFKLQNFQLPQATSRETCVRNVYAFQVLQNVFLKSTTPALCCTILDAISRVYHSENANYFILESEQTLSSFAERIHMK
SPQIQEKFYDLLEFIVFQLNFVPCKELISLSLLLKHNQSTSCSILCLKTLLNILRHNAVFKDVYREVGILEIFVGCLTRY
AAHVQKITKGDESVVVELENESEEELFDTLGKHVLEALTMLLGGGASNNAQLFREYGGAKCVHELVKFKHCRPQALGIVR
ELILSAGGDDDMLHILSLMHSVSPLQVEFKIQILNMLLGCLKDSHRTRTVFRKVGGFVYVTSVFVSLDGSMALPQPDIPQ
QDLILLLQIVFQTLATAMRFEPANAKFFHQEISSSSLCDTLRLLGCFGGSSALQDYTGTYEPQQSLLKYYHEIFSGDILS
VSFSEDVPYPLSYVCIVFRLLYSIALDNFEAPNLSGIITLFSDPPTTLRSPSKELAPPVHPSQLNLTQPSPEPRIVHPGV
VLCMLQLLPAVEYDMAPLQAVQLQVYLSEIIKSLVRSERNQQIMCDHGLAEKLLKLTRRALAEESHPLHVPMQYILERLA
AQALQPTELRQFLRLGEPLSCADIDLQQPYKLGGPVPLTRIKTLVSMTTPRDFRAHGSSTLPPFVELDMSAEGFGCLYLP
SLAPQATATAGGTIDANSIGGIGAGDRIFPPQTGLTYSTWFCVEKFSDPKTDPHCVRLLTLVRTIHNPREENLACLSILL
SARDKAIVVSTQETLVTPRKSIGDWEPEGSDDGIARIWCPDLLHEGQWHNLVVVLNRAVLKNSSLFLYLDGVPMHTQKLH
YIAQHPAAGNASLTSPTQIFGYIGTPPIWRRYSRLCWKQGVCHLIEDVLTQQTVQTIYQLGPHYMGSLQAPQLGKQSESL
APLVPEDRVLLGLNAKAVSKLTLVKIRKVYSRADNKSIAKQLNMNSHENATPIKILHNSAGHLAGAGRSLGGVVVGYLGV
RVFSPHPVSAMIDTVGGCNVLLGIIAMAQDVESLYAGVKALTCVVRSNRAAQAEMDRKRCYQTLGMFFKKKKHLLNSHIL
HLTFGLVGTVNSGQDMSAIPNVTAFQDLLCDLEIWHNAPNGLLRSLLEHLLELVVESSDKKQNVKIMRDLQLLVKLLHII
TQIQDHSTREILFSLLETLLGGQPRHTDLLLFGQYVAAKLPHAHSGGLERAVLLPSMKNPAEDQDGGVAQNIYLRNRCLS
LLHGLLFTPRNTVNYVICDDISKTLGMDWLLLFMQPHVHFTTVIIAVRILVVICANESFLVRFRDATHNGGYLRFTEMVS
QRKMLGLGAQQLNQRPTNGTGTVIVATPQNTIQHLPTQIAGEVRAAALNIPGFQLLEWLMNHHLDVPELYFLITALIMGQ
PVKVLATEHTKFDLDRVWSFLWGAPVSANTQLPKLNICPEGVCVLLAMVRGIVHGGECAPWLHSHPETIIQLLFSLYQNL
TDFAPVMMTGDVVTSLVAVLFPLASRPADSEPNSGASTPTDDTGSSFALPPPETLHGAPQPKLTAHPVCNCIIDFLRVIV
VDSLGLNMQGKPTPVIDLVLDAAPESAELPLQVQYQTQIIIALMDHLLAADVLVGEQAALPLVPLLQSQMQYIAPNVFYL
TARIVDKLWQGCLARNPHDIFDFVIKLIVQAKRRSSSLSLEHLHHSLNRSILFLLSRPTDDSRADQMSVLEALHKIIQHR
LLIFGAGNHELEFIGCLTYCLLQLTADMKIILEPATSRNTTWHVNPQTETAEPKDEDLNQLQGRNLIVGAAFRVWEELYV
CKKPAIEEVFKVSLTSPPPNSKAPDLQTTREQVMELASKLWFNYVEAERKATYRAPWELHTQIQSKIQKVTGGLSRLTSR
TKTKKEELVRTRSTLTREAAYESTGIHVQLIKDLLDLRAKQYQQMLQHTQRYVYQDWVQSEMELTRERGLWGPTGSCSLD
KWILDTTEGPHRMRKKTMRNDVFYLHYPYRPELELADNRQLKYKVASSLDSKTYALHGPQQPRILAEAGEHHAMQQQSSL
EAVQSHRLETSSSTSTPPPMVLPKLVGHGSTPCPQESVDGNAPEDDEEEEDTSMTSDNETFLRLLEEQEKISFMFRCARV
QGLDTFEGLLLFGKEHCYIVDGFTLLKNREIRDIDTLPPGAYEPIIPNSGGTSSTTSRAVSHKLRQCSKFAYEEIREVHK
RRYLLQPIALEVFSEDGRNYLLSFPRKVRNKVNQRFLALATALNDNAQQSVAGQKRTASVEQTAGIFSGLIGETSVTQRW
VRGEISNFQYLMHLNTLAGRSYNDLMQYPVFPWILADYDSEELDLTNPKTFRDFSRPMGAQAEERLEQFQKRFKEWDDPH
GETPPYHYGTHYSSAMIVCSYLVRLEPFSQPFLKLQGGHFDLADRMFHSIKEAWLSASKLNMADVKELIPEFFYLPEFLS
NFNNFDLGTKQNGETLNHVILPPWAKHDPREFIRLHRSALECDYVSQHLHLWIDLIFGCKQQGPAAVDAVNVFHHLFYEG
NVDIYNIDDPLKKNATIGFINNFGQIPKQLFKKAHPAKKMGGSRHSALIDPTSLIQGNSTVLQTDRLFFHNLDNLKPSLQ
PIKELKGPVGQILQPDKTVFAVEQNKVMMPPSYTKYIAWGFADHSLRIGLYDTDRASFVSEASAQNSGEILTCACPNAKM

IVTAGTSSVVTIWKFDANRKSLAVKHSLHGHTDAVTCLAASAAYNVIVSGSRDGTAIVWDMTRFTFVRQLRGHAGVVAAV
SINELTGDIATCSATWLHVWSINGDALAMVNTCVGSADRMQQILCVAFSQIREWDQQNVIITGSTDGVVRMWSLEHTQVP
IDRKLKRGTEVSSQDKPDTVSLEGKDNKDDKMSLMKQIKSLSQSEEEMEIVKSASESSISEASQHSHGSSKSAEAGPKAE
VQGERRKSSISGAKSLHEMKSATVEGQGSSYDPKSNEDEHAIRPSKSDTSLTDGFVVIDNDRHKGDQILRKGFRWQRQLM
FRSKLTMHTAYDRKDNAEPASITALTVSKDHKILYVGDARGRIFSWSVTEQPGRGVADHWLKDEGADQCVKCHVKFTLYE
RKHHCRNCGQVFCNKCSRFESEISRLRILKPVRVCQACYSQLRTNSLDN
>NP_001104451.1 uncharacterized protein Dmel_CG41099, isoform D [Drosophila melanogaster]
MGSNETFSVLKMEKILAVQSDEYTKLQRCYVELQRKYNENIASSENEESGLSSFLSRLSLTVASLFDKNTYADIYIRSQT
RVFPAHKIVLHARSEKWGNDLLSNIQQLDWSDLNEDVVLSLLRWIYTDLIDLQNDGLALDLLKAAHRFGLPSLLGICERA
LVTSVGVRSCIRFYCVAEEVGASTLLEYCSSIISTHWDDLTTDDFQHMSGPLLFEMLKTKTKHPLHAAVRLLREDVVSLC
IQKYGNSLVNTFSENGILPLEMALSSKNAKIAKSLVDNGMANINAVNMEGFSLLKSALKNGDAFSANFLLDQNCLLDLPS
KPSSDTALHIICNYGPDNTPEIMEVVKKILQRQLNINIQNIKGETPLHIAIARRNVEMVKLLLKVPNIDINLRTYDEKCA
LELSLSMGDHEFLIASILLSMGARTDRTNSKTGDSLLQVFALDRHRGEKSAIFLADFADLDHINFRGLTALHIAALNNMP
NLVKKLIVNGASSNLKHIDCGLKSALHIAVEANAIDALEAFVELKNKSHDIDFNCQDINGDSPLSLCLSLKRTTLVPTLI
RGGSDVNGKNKNNLSPLHQSIKNEDSDISLFLLEQGADITALTENLDSALDLSIKHDLSEVVDALCRRGIALSINKNGES
PLWSALEKGYEDVAKILVRHGIDTDCWDEGPEGCRQTLLHRAIDENKESVAIFLIQSQCDLDSSRQPGPNGEGGDEAQDK
ASPLHLCCHWGQTKVLQTLIDHGANVNLIDAESKSPLHVAIESQYDEIISILLCHPDIDLKLRDKSGNTPFATALDFRNH
NAAQRILDRFPTAAEQMDIRGRNFLHLAILKDDLESVLFLLAIQVDVNSRVHDANQSSPLHLAAASQNEMITRNLILAGA
RMNERDAVQKLPLHIAIERGNLPAVSALIQNNADYDATDADGNNALHIAVRCAQFFIVRELLTESRVNAEATNLKGRNPL
HELCRVVEDSTAGLICELFLESMPKYPINIPDMDGNTPLLLSFMRGQSPLCKILVKAGACLGTENKDGINIFNFKLATDQ
LLHNLLDQLPQESPWAESDYCQHCTNRFTITMRKHHCRHCGRVLCSKCSCNDVPILKFGINKPVRVCTVCFNVLQCGNGS
LS
>NP_001015359.1 uncharacterized protein Dmel_CG41099, isoform C [Drosophila melanogaster]
MKTGSNETFSVLKMEKILAVQSDEYTKLQRCYVELQRKYNENIASSENEESGLSSFLSRLSLTVASLFDKNTYADIYIRS
QTRVFPAHKIVLHARSEKWGNDLLSNIQQLDWSDLNEDVVLSLLRWIYTDLIDLQNDGLALDLLKAAHRFGLPSLLGICE
RALVTSVGVRSCIRFYCVAEEVGASTLLEYCSSIISTHWDDLTTDDFQHMSGPLLFEMLKTKTKHPLHAAVRLLREDVVS
LCIQKYGNSLVNTFSENGILPLEMALSSKNAKIAKSLVDNGMANINAVNMEGFSLLKSALKNGDAFSANFLLDQNCLLDL
PSKPSSDTALHIICNYGPDNTPEIMEVVKKILQRQLNINIQNIKGETPLHIAIARRNVEMVKLLLKVPNIDINLRTYDEK
CALELSLSMGDHEFLIASILLSMGARTDRTNSKTGDSLLQVFALDRHRGEKSAIFLADFADLDHINFRGLTALHIAALNN
MPNLVKKLIVNGASSNLKHIDCGLKSALHIAVEANAIDALEAFVELKNKSHDIDFNCQDINGDSPLSLCLSLKRTTLVPT
LIRGGSDVNGKNKNNLSPLHQSIKNEDSDISLFLLEQGADITALTENLDSALDLSIKHDLSEVVDALCRRGIALSINKNG
ESPLWSALEKGYEDVAKILVRHGIDTDCWDEGPEGCRQTLLHRAIDENKESVAIFLIQSQCDLDSSRQPGPNGEGGDEAQ
DKASPLHLCCHWGQTKVLQTLIDHGANVNLIDAESKSPLHVAIESQYDEIISILLCHPDIDLKLRDKSGNTPFATALDFR

NHNAAQRILDRFPTAAEQMDIRGRNFLHLAILKDDLESVLFLLAIQVDVNSRVHDANQSSPLHLAAASQNEMITRNLILA
GARMNERDAVQKLPLHIAIERGNLPAVSALIQNNADYDATDADGNNALHIAVRCAQFFIVRELLTESRVNAEATNLKGRN
PLHELCRVVEDSTAGLICELFLESMPKYPINIPDMDGNTPLLLSFMRGQSPLCKILVKAGACLGTENKDGINIFNFKLAT
DQLLHNLLDQLPQESPWAESDYCQHCTNRFTITMRKHHCRHCGRVLCSKCSCNDVPILKFGINKPVRVCTVCFNVLQCGN
GSLS
>NP_001015360.1 uncharacterized protein Dmel_CG41099, isoform B [Drosophila melanogaster]
MEKILAVQSDEYTKLQRCYVELQRKYNENIASSENEESGLSSFLSRLSLTVASLFDKNTYADIYIRSQTRVFPAHKIVLH
ARSEKWGNDLLSNIQQLDWSDLNEDVVLSLLRWIYTDLIDLQNDGLALDLLKAAHRFGLPSLLGICERALVTSVGVRSCI
RFYCVAEEVGASTLLEYCSSIISTHWDDLTTDDFQHMSGPLLFEMLKTKTKHPLHAAVRLLREDVVSLCIQKYGNSLVNT
FSENGILPLEMALSSKNAKIAKSLVDNGMANINAVNMEGFSLLKSALKNGDAFSANFLLDQNCLLDLPSKPSSDTALHII
CNYGPDNTPEIMEVVKKILQRQLNINIQNIKGETPLHIAIARRNVEMVKLLLKVPNIDINLRTYDEKCALELSLSMGDHE
FLIASILLSMGARTDRTNSKTGDSLLQVFALDRHRGEKSAIFLADFADLDHINFRGLTALHIAALNNMPNLVKKLIVNGA
SSNLKHIDCGLKSALHIAVEANAIDALEAFVELKNKSHDIDFNCQDINGDSPLSLCLSLKRTTLVPTLIRGGSDVNGKNK
NNLSPLHQSIKNEDSDISLFLLEQGADITALTENLDSALDLSIKHDLSEVVDALCRRGIALSINKNGESPLWSALEKGYE
DVAKILVRHGIDTDCWDEGPEGCRQTLLHRAIDENKESVAIFLIQSQCDLDSSRQPGPNGEGGDEAQDKASPLHLCCHWG
QTKVLQTLIDHGANVNLIDAESKSPLHVAIESQYDEIISILLCHPDIDLKLRDKSGNTPFATALDFRNHNAAQRILDRFP
TAAEQMDIRGRNFLHLAILKDDLESVLFLLAIQVDVNSRVHDANQSSPLHLAAASQNEMITRNLILAGARMNERDAVQKL
PLHIAIERGNLPAVSALIQNNADYDATDADGNNALHIAVRCAQFFIVRELLTESRVNAEATNLKGRNPLHELCRVVEDST
AGLICELFLESMPKYPINIPDMDGNTPLLLSFMRGQSPLCKILVKAGACLGTENKDGINIFNFKLATDQLLHNLLDQLPQ
ESPWAESDYCQHCTNRFTITMRKHHCRHCGRVLCSKCSCNDVPILKFGINKPVRVCTVCFNVLQCGNGSLS
>NP_730882.2 Chondrocyte-derived ezrin-like domain containing protein, isoform F [Drosophila melanogaster]
MSLADMGTASRSAGGEGGRHYDLATGGAGSGGHPVGGGLPGGRMTHSLSTPSGVDGTPSTPRHRGGKKLTVRIQMLDDSI
TMFQVQAKALGRVLFEQVCRQLNLLEADYFGLEYQEVSTHTKYWLDLEKPMNRQVGLSLIDPVLRFCIKFYTPDPAQLEE
EYTRYLFCLQIKRDLATGSLQCNDNTAALMASYIVQASCGDFVPEDYPDHTYLSSYRFVPNQDATMQRKIMENHKKHVGQ
SPAEADLNLLETARRCELYGMKMHPAKDVEGVPLNLAVAHMGITVFQNITRINTFSWAKIRKISFKRKRFLVKLHPEGYD
TVEFFFEGRNECKNFWKKCVENHGFFRCTAVQNTPRRKTRVLSRGSSFRYSGKTQKQIIEFVRENYVKRQNFQRSQSFRQ
GPLNASSRSQSHTYVNSSISANPLLPIDTAAWDYRNQCSDSMTPSLTKKAADTLDRRRDNPIGHMRSQVTAAQVEIYQTK
NYAADSPTSQEEAECSAAGAERQHHSAVAMDKLNSNRSLSPQGPQSWTSPSHSSHHQQRGPDPARVHPGDHNTDGYYGIN
GNMSLDRRGEITTPPTRYDLTLGSDKSSSLSRSEAGTYDVIQAEIQHAKRQELATGVATASHQNGNGNGNGHTLSTQHDI
EAEVKKRKWPTEPSYFLAKELLMTERTYKKDLDVLNTTFRQVLSLGDVEQLQPLFELLDSLAQHHNLFLRDIEHRMVQWE
GRGGHEAHRIGDVMMKHMAALPIYDEYVQTHLDILHCMNDMYEGDERFRQVYKEFEQQKVCYLPIGELLLKPLNRLLHYQ
LILERLCDYYGEEHIDYADAMAVHHLLVRSTKGIRSQLPDSANFVELCELQRDINFEQLVQPHRRLIRQGCLLKHSKRGL
QQRMFFLFSDLLLYGSKSPLDQSFRILGHVPVRSLLTENAEHNTFSIFGGQCAITVSAGTTAEKTLWLAELSKAAADIKN

RPPNMQLQLTTLKNCSSSEEGLDLFGLSNGNNSSLNSSVNGGGPLTTQQQKLQLQQQQQNRTQPSRSNTALHVCWHRGAT
VGLGDHLIAAEHQLSGYLLRKFKNSSGWQKLWVVFTSFCLYFYKSYQDEFALASLPLLGYTVGPPGHQDAVQKEFVFKLS
FKNHVYFFRAESAHTYNRWLEVLRSTTQTQDFKNVHSHAVLGN
>NP_001036676.1 Chondrocyte-derived ezrin-like domain containing protein, isoform E [Drosophila melanogaster]
MSLADMGTASRSAGGEGGRHYDLATGGAGSGGHPVGGGLPGGRMTHSLSTPSGVDGTPSTPRHRGGKKLTVRIQMLDDSI
TMFQVQAKALGRVLFEQVCRQLNLLEADYFGLEYQEVSTHTKYWLDLEKPMNRQVGLSLIDPVLRFCIKFYTPDPAQLEE
EYTRYLFCLQIKRDLATGSLQCNDNTAALMASYIVQASCGDFVPEDYPDHTYLSSYRFVPNQDATMQRKIMENHKKHVGQ
SPAEADLNLLETARRCELYGMKMHPAKDVEGVPLNLAVAHMGITVFQNITRINTFSWAKIRKISFKRKRFLVKLHPEGYG
YYKDTVEFFFEGRNECKNFWKKCVENHGFFRCTAVQNTPRRKTRVLSRGSSFRYSGKTQKQIIEFVRENYVKRQNFQRSQ
SFRQGPLNASSRSQSHTYVNSSISANPLLPIDTAAWDYRNQCSDSMTPSLTKKAADTLDRRRDNPIGHMRSQVTAAQVEI
YQTKNYAADSPTSQEEAECSAAGAERQHHSAVAMDKLNSNRSLSPQGPQSWTSPSHSSHHQQRGPDPARVHPGDHNTDGY
YGINGNMSLDRRGEITTPPTRYDLTLGSDKSSSLSRSEAGTYDVIQAEIQHAKRQELATGVATASHQNGNGNGNGHTLST
QHDIEAEVKKRKWPTEPSYFLAKELLMTERTYKKDLDVLNTTFRQVLSLGDVEQLQPLFELLDSLAQHHNLFLRDIEHRM
VQWEGRGGHEAHRIGDVMMKHMAALPIYDEYVQTHLDILHCMNDMYEGDERFRQVYKEFEQQKVCYLPIGELLLKPLNR
L
LHYQLILERLCDYYGEEHIDYADAMAVHHLLVRSTKGIRSQLPDSANFVELCELQRDINFEQLVQPHRRLIRQGCLLKHS
KRGLQQRMFFLFSDLLLYGSKSPLDQSFRILGHVPVRSLLTENAEHNTFSIFGGQCAITVSAGTTAEKTLWLAELSKAAA
DIKNRPPNMQLQLTTLKNCSSSEEGLDLFGLSNGNNSSLNSSVNGGGPLTTQQQKLQLQQQQQNRTQPSRSNTALHVCWH
RGATVGLGDHLIAAEHQLSGYLLRKFKNSSGWQKLWVVFTSFCLYFYKSYQDEFALASLPLLGYTVGPPGHQDAVQKEFV
FKLSFKNHVYFFRAESAHTYNRWLEVLRSTTQTQDFKNVHSHAVLGN
>NP_609186.1 Rabenosyn-5 [Drosophila melanogaster]
MSGNPFDSDEDQSSASGEILEGFLCPICRADLKSIDVLTDHFARQHAEEEDALKSFKDIFTKAKKKILNPFEDEKGASVS
SPAGSSSSAAGKNPNGNGDRNGPSNARSRLNVFNYMARQQPGAECSHFEYFQSIRNPRLERYASETNKLIIRLHRLLKDL
PADSVQRRQHEQQTVAWLDGSSVKLCPSCAKSFHIARRQHHCRLCGGIMCNDCSKFLPLEDAMQLASLSTTRSEPLQQLH
QHENAIRLCEHCLWLLDTRKDMHESRTCRPLLVQVYEQIRQLQKEVTPDLDMYLKIINSLNEGDTIFTLADAGALRGKIG
QVAEAMDMRSKRILAIFCEPGSREEALKKAIRLGCIQAIKERMLSLPPLPEESHIRQMQERRRMETEQRILTEQRMAMEA
YERNNMAANQSNVGVPGPESGSFAQGSDLQSLNNWSAPQAASKSSLDDPLIEQINIIKGYIKQARQDMNFEVVETLELNL
RELQREVYERQRQSQGSTAASPTEA
>NP_611545.1 Rabaptin-5 [Drosophila melanogaster]
MEENETRSEAREPTDLPNGEDDATSQHKLSHLQNEMRKMQNEFNTQRAKMRELYIQKEAEVSQSQQERRQLQAELDELKT
HLMVADLKSQNELQLRDLKAQEEISSLQQLVQDTIEETAHYKGEVERLRLELGKYQQIQQQTMAQQPQAESSGGIAPQVL
NQVKKTLGSVRKLGTDSLNSSFQQDEDTRASSKGNGKQYAPEEAEMMHSIVEQLQEEMKALKVKLREQDEQLQAKSASDE
SALHKSTSMDVAESACESCSLAEKKTEELGAHINKQQKQVDLLQKQLVESRETLVKEAALRKDLEDQWQEKREAHKSEVQ

SLRDQAKTNEQRLLDMQQKFLETKDEVIKQIQRVSDDRERVNKQLETLQADNDFLSGRYLATSEEIDNQYINLPNTVVEL
QELILRQQSELIQARVSSEYERQKCTSTEDEIQILRAQLEESNNERRAYKRKMQLDIKSLQDRVTEHLVTVQAYETTKTQ
LERKEAELNKQLSECRVEIIELQEANEKYAKTNADYKTKIKTLQEELSTMETVQKDFVKLSQTLQMSLEELRHADTEVRW
QDDDDVNNCPTCNAYFTVMVRKIHCRHCGHIYCDKCLTKTVPSGPRKRVARVCDICHTLLTPNTAPYFSQGQPPQQQQGQ
QQQQQSN
>NP_611269.1 fab1 kinase, isoform A [Drosophila melanogaster]
MTSNNQNNSSSHQHLHSPSKLTEFARNFEDKPESLFGRVVNKIQNVYNQSYNTVNDISSGSSSSSSTQPVQVVGKSQFFS
DSQTSTAEIADVETSSQSSVRPQPPTTLSIRTNSETRGTSTSSNTAAEDSETSDRVETLPLPTSEANQGRTVSNVLKHIS
NIVATKNNNDLRNYKDTELQRFWMPDSKAKECYDCSQKFSTFRRKHHCRLCGQIFCSKCCNQVVPGMIIRCDGDLKVCNY
CSKIVLTFLKSSSSEMGQDMQELQQHLSNKLEVQDSGSSLAKHPQMQRAPLPRKTSVGYQEERFSSHPTYTTLSIDDRKN
ILQQSNSLITLHEEMQRDLPAQNCGQRLIEFLNSNNKSANEVQAVAILNAMLAAGFLEPIVPDPEQMDFDSSLHYKFSKS
SSSDTSRTMSPQFEANPHAEPQPPKSMDQSAEEKEKELENELENDRCYTTATSKLLASYCEHEEQLLAQMLRAHNLDQEW
DKVLQMLCSTAANHFKPEHCSNDLMDIRNYVNFKKVPGGRRKDSKIVHGVAFSKNVAHKDMATHVPFPRILLLQCPIVYE
RIEGKFVTIETVLLQEKEYLRNVCARIMSFKPNVVLVHKNVAGIAQDLLRSYEVTLVLDVKLSVMERLSRTLQCDIVSSI
ESNITMPKLGYCNDFYIRNYNGKTLMFFEKLTNPRGYTCLLRGGSNAELTRVKRVASALLFARYNWRLEMSFLLNEFAQP
LSPKPSIFDSKETSPKTETEAELRSKRPIILERKSEDKITTIVSENVSDFTDPLRASQAEALSTSPCAPPVVEALAVEPR
YDNRFRTALSSTLLSVSPFLTFPLPYLETEQGRKCKLRKLFPAELYFSKQWSRTGLERPDSMGDGEAGKSEPGNKENQMQ
LLPAHDFVLMKITAPASSRDIQSKLAEFRSFGGRLPKGKAPMLRPKKKNAEVIQRPQKVSEEQLYKDALDPQNHQRLPVL
FCSFHYNPKGVSSFCKLPMLLDMKFYGQYDIMLEQFLQRYCCLFNSMCPSCNLPMLGHVRRYVHSLGCVHVYLTEDLTRS
DPTRIYFTSWCSICNATTPTIPLSDAAKCLSLAKYLEMRFHGHAYKRRPPSTDAEQGGTVCEHSLHRDYVHHFSFRGVGA
KFQYTPVEVWETDLPSLTVQLDLPQPFQSAQVQEEIKNFSIKGHEVYNRIHERIADLATEEENSPLVQHLKTMLTHDQFI
FKQKIEIVHTLLTDNRATAYDTSDALAMARRALAESIELWGPRLQEIEKLTAKQAHHIDSGTICTEELRPEQVQTADSSK
VTTSSLPKENDPLECPSEDTETGASNSQTVLDKNFSIDQMLASTVNVYSDKKSIRKILTQLLPSGNQVNPLQSPFPAQDH
LTLPLGSIPIHVRETDLSSVIAYSLTSMDYQKAIDEAEANSNAAHSSPQLKRKIPLAESVSDAEDSPSLSRTSSNTSAAP
NASVPSPATAASESEEKSKERIKQPPSPHITLAFQDHSCQFQCKIYFAREFDAMRSKSLKPPKLDKSLYRRLEKSKMREE
LRISQSRTGSEMELVRKPSDVGAPRTTEDDSNQEEDARIALARSLCKSVQWEARGGKSGSRFCKTLDDRFVLKEMNSRDM
TIFEPFAPKYFEYIDRCQQQQQPTLLAKIFGVFRVSVKKKDSFVERSVMVMENLFYGCNIENKFDLKGSERNRLVDPSNQ
QGEIVLLDENLVQMSWSKPLYVLSHSKTVLRDAIQRDSSFLEKNLVMDYSLLVGLDKKNGVLVLGIIDYIRTFTLDKRVE
SIIKGSGILGGKGKDPTVVNPERYKQRFIDAMDRYFLTVPDRWEGLSKV
>NP_731570.2 uncharacterized protein Dmel_CG5270 [Drosophila melanogaster]
MEDQQEENMQQLLNLLPKDQRQIFEIFIQWLNGKSSGPIQLHQNALLTVLQASPYPALQLLQLLHERSKLHIGQIISTLL
RQLLDGDVASPRSLCVLANFPASVLEASTLRQKLMTRLVVDSITSEHLMLTMLARSDGQLLDDLLHEQQLTMRAQCSEAA
PTKLLVLWLALNEREHFVASVCQHLKDFKCFQDVTLRNNLLILRLANEFALGSQTLEELGCLEKLLAEFDVSAVPEKLQE

VHGFFYADFQRLSTLLNFLRPREISKTLRVDALLRAPGVLPLIHENGIHFKHQELHRLIEDTYKWQQLTPALKCHHQEEV
EILSYYTALCHVYDVVLDQGEQTTKTKLVQLSVQLRQLHQLGALCSLLEDIFLLVFLRWEQLEPNSQRKGEDGENDEEDD
EQYVDDDVASPPRPTAVHSQRTRYGFICRSASLHALFTFLKAFVTKKIHSQDYKSAPEQQGRFQRLVDAISEALWKLGVL
QKIEQSLLKSAPSISCLLEPDQLLQLVQLHSTAKKKASSDDESRERSNHASSLNRRKARRQRRAVSFSGGVAGKASADGG
LAMEQLRARAQLLSGSGSRKNSSVTSPCERSIIPKMLSTPEQLAIMALALKNFNDVKKIIETFHLEHSQLNRELHFMEQQ
QLVKQKLSAIYANYQILEGQQANSGETTVEQIKGVAAKGFELSKIISVVDNFSQAHRLQQSPELKALIQRHNTSAQQGFL
QQFEERNLNALIICDLIVNLGFNREITSNLLLVIRRQQQQKKGGDEASNTPGSSELGAMNLLQNLCECMRLLERSGQQAA
LNELLSLKSYPLRPAVLALQLQREAAFQTLYQKEPSEYSHGHELRSNANQFQQLRSRHNYYARFCTYVQQLARLLQLRDP
NLEYHTTQLLRNDPYEVIGELIYECGITPLEIEASVAALHLNLVHVIALNICPQLSEEPTKRLPRVVAPQKQESIHNYIS
QHNQLLAHILLAIQLGSLPDGDADVDFSFLGHLVHLSEVNVLASVYDGNRVLAALNAYKLESARLDQLVTDQEQLLQILL
LGMSGQTDSPNRHKSRIDQLIGDLIEKDPRNIHLVVHMGNLGQRAQLLKEHFTRIPSSQQAKELIERTLHHRSAAKSIPT
ALRSELEHTLSDITIYARVSALLKFESWPQAYDFGRQTPNVIFEQLLQRRRFGLCLEWSRVVYLAGSAGQQRVCLLTLLD
ALLELRDGEELDESLLGIVEMFPPNPLVNFLDTHKDKFRSLPLLQWVIDYLEGHARDPRLYRNYQLSLEFLRQMDTNERS
PFWKLIRHPLLIVEQLVMNARFELLGKLLDAARSKLLKERPLGPCPYCFDKTGHVYDVQSSAGLGSGAGETPAKIRFQLG
QTTSEAFILLNFNSYQQDHFVGQDCLDLLLRIYASKALDYHVANVRAASEPSSLGTDVHNSLDSLCGAFVMPKQAPNRQQ
WTRDEEASHCMCCRRAAFTMLMRRHHCRRCGRVVCYACSTHRIRIPELYDELEVRICNDCAACSTPAKDQGDGTSSERSA
ISGQVSKSSGRSDSCKWQLSGIITHDKLLREEFSYEHAPSVALSLSILRNHVEQRSCVDLLLFHCRKLEKLIVPNPEVDY
GLVAKMINCLAFAAKVRGAPGELENIREHSEIIMAVVQQGCESLIPTGPLNNHNLRKLADALVEAEHWTLALEVHLKCGF
ATTGVMAAHGLACLRAGCYDAAREKFAHCMTRLSSEQLNSSICKNIFGVASTEAVLLPRKRPQRGPALLQEILQLIAAIP
QVQTQPETLHRASLIRSSNTSLASLFTRRREPYVQPRPLQEPALNIMNALAGLKNLAKGQYGGQMPTSEESRRQERGFEE
SLHYVLTYGSHADILTFLMRREELRAALRYWQHQQLDADLFIHHIFYPQLANGGLNVLMDELQQLDDAQFTAWRLPLLQT
CRHLEQQQQLSSLYQLQLLLKDPIRASMTCVKFYALQCENFQKLHANAQHLLSALRHLQGELDMAEWEHLQRQQARRNSV
SSTASVRGACFAMQMDARALNGHINTIRRQLEVAKFLDKCEREQPPDEPLRTIQTLKQIRLESSRGTLPTLFEGAADRIQ
ICILILMCGKNIDEGFGLAYGIMQDFKLAALKVFGATAKYLSRNQRLGEVERLLDCIGSNNGGDISTESDEILSIAINAA
VHSSAPETKQMLDRLIKRIGSVELRVSSYIYIGQLKSAYLLANKHDRLADIRRILRQAELTGQVHIKKLCDMKLQLSAAP
TPL
>NP_001188779.1 WD repeat and FYVE domain containing 2, isoform B [Drosophila melanogaster]
MAAEIKPAQRTNNDRFNTSKKPELLSKLEGSSDDVNAAILIPGENGVISVSDDKTVRVWLKRDSGQYWPSICQYMPSGCT
AIEYVSESRHLYVGQENGTVTQYALSEDCNRLSFLRDYLSHQARVMAVVFSKTHKWILSAGKDKQFAYHCTESGKRVGGY
NFETPCTALQFDALAKYAFVGDHAGQITMLRCDVQGVQLITTFNGHSAEIRCLKWVEGPQLLFSGACDQSVLVWDVGGKR
GTIYELQGHSNKVSALSYANHTQQLISCGEDSVVVFWEMNAMRKEVPGWVDTNNCQLCSRPFFWNFRSMMDQKQLGIRQ
H
HCRHCGKAVCDNCSTNRINIPIMGFEFDVRTCDPCYKQLQTVERPSLASFNDAKHSIVYMDLDEDRKRLLTVGQDRLIKI

WDLSNIWA
>NP_611791.2 uncharacterized protein Dmel_CG3530, isoform B [Drosophila melanogaster]
MDEIKLAKVENVRMIDRYNTKNPTVGTLYLTATHLIFVEPDSNKETWILHMHVASIEKLPLSTTGSPLLIRCKTFLSVTF
VIPKDSECHDVYTSLLKLFQPVSINKLYCFNYQPNKDDFPKNAGWDYFKLEAEFKHMLVPNEAWTLCSMNEKYELCDTYP
RQIYVPKEATTLMLISSSRFRSKGRLPVLTYLHNNKASICRCSQPLSGFSARCLEDEQMLEAIRKTNSNTDYMYVVDTRP
RINAMANRAAGKGYENEAFYENIKFHFLGIENIHVQRASLQKVLEACEQKSPTMSAFINALESSGWLKHIRSILDTSSFI
ANAVDKGVSVVVHCSDGWDRTAQVCSLAQLMLNPYYRTIKGFQALIEKDWLAFGHKFSERCGHIQTDAREVSPIFTQFLD
CTWQLMSQRSEAFEFNERFLLILHDHVHSCQFGTFVGNCEKDRLDLKLAERTFSLWGYMANHLNEYINPLYKPNVDEAIK
ANLAPQCIKFWRGMYSRFESGIHPREPLGDVLLDSKEHCNSLEDHVQHLTKRIASFKNYISKSAKKLQDATSAPGKVNKE
LSSNEINDNKYNYDKKLSELSAADDDHPLKASNMSFASLSLSAEQSSPPQALPEEINSVAVDWKPMRNVTTCSCSTPFDQ
FSKKTHCWRCGDIFCERCIDKNVALPGHDSGKPVPVCRGCFRQMQKQSP
>NP_726322.1 uncharacterized protein Dmel_CG3530, isoform A [Drosophila melanogaster]
MDITRFMNKFNEIERQLSPTSALRLSFDNISPETEEPPDGNKRLWGVPVPPFVSEKVVSWIEEEFNEAPVFYNGNSVLKK
VENVRMIDRYNTKNPTVGTLYLTATHLIFVEPDSNKETWILHMHVASIEKLPLSTTGSPLLIRCKTFLSVTFVIPKDSEC
HDVYTSLLKLFQPVSINKLYCFNYQPNKDDFPKNAGWDYFKLEAEFKHMLVPNEAWTLCSMNEKYELCDTYPRQIYVPKE
ATTLMLISSSRFRSKGRLPVLTYLHNNKASICRCSQPLSGFSARCLEDEQMLEAIRKTNSNTDYMYVVDTRPRINAMANR
AAGKGYENEAFYENIKFHFLGIENIHVQRASLQKVLEACEQKSPTMSAFINALESSGWLKHIRSILDTSSFIANAVDKGV
SVVVHCSDGWDRTAQVCSLAQLMLNPYYRTIKGFQALIEKDWLAFGHKFSERCGHIQTDAREVSPIFTQFLDCTWQLMSQ
RSEAFEFNERFLLILHDHVHSCQFGTFVGNCEKDRLDLKLAERTFSLWGYMANHLNEYINPLYKPNVDEAIKANLAPQCI
KFWRGMYSRFESGIHPREPLGDVLLDSKEHCNSLEDHVQHLTKRIASFKNYISKSAKKLQDATSAPGKVNKELSSNEIND
NKYNYDKKLSELSAADDDHPLKASNMSFASLSLSAEQSSPPQALPEEINSVAVDWKPMRNVTTCSCSTPFDQFSKKTHCW
RCGDIFCERCIDKNVALPGHDSGKPVPVCRGCFRQMQKQSP
>NP_001097433.1 uncharacterized protein Dmel_CG3530, isoform C [Drosophila melanogaster]
MHVASIEKLPLSTTGSPLLIRCKTFLSVTFVIPKDSECHDVYTSLLKLFQPVSINKLYCFNYQPNKDDFPKNAGWDYFKL
EAEFKHMLVPNEAWTLCSMNEKYELCDTYPRQIYVPKEATTLMLISSSRFRSKGRLPVLTYLHNNKASICRCSQPLSGFS
ARCLEDEQMLEAIRKTNSNTDYMYVVDTRPRINAMANRAAGKGYENEAFYENIKFHFLGIENIHVQRASLQKVLEACEQK
SPTMSAFINALESSGWLKHIRSILDTSSFIANAVDKGVSVVVHCSDGWDRTAQVCSLAQLMLNPYYRTIKGFQALIEKDW
LAFGHKFSERCGHIQTDAREVSPIFTQFLDCTWQLMSQRSEAFEFNERFLLILHDHVHSCQFGTFVGNCEKDRLDLKLAE
RTFSLWGYMANHLNEYINPLYKPNVDEAIKANLAPQCIKFWRGMYSRFESGIHPREPLGDVLLDSKEHCNSLEDHVQHLT
KRIASFKNYISKSAKKLQDATSAPGKVNKELSSNEINDNKYNYDKKLSELSAADDDHPLKASNMSFASLSLSAEQSSPPQ
ALPEEINSVAVDWKPMRNVTTCSCSTPFDQFSKKTHCWRCGDIFCERCIDKNVALPGHDSGKPVPVCRGCFRQMQKQSP
>NP_647623.1 uncharacterized protein Dmel_CG12099, isoform B [Drosophila melanogaster]
MDSNNSSNSKKQPQSSHHGKSSSAESQRKSSELHTNSNKQRHSRRRNQVPTPRNDQQQPQQPAKLRPNVDKRPKARGFG

GGYDFPSPRGQDGASGSSRLAYSATGYSRSGDFDHELNSVYTQGSKKQNLNHLLNFHCVPRELERGHHHQAQQHHGLSGR
KQRYNKEQFLQANFQFVIRSGAKAQVNGSPDALIDWSYIEQINIQTTEELQCPICLYPPVAAKLTRCGHAYCWPCLLHYL
SLSDKTWRKCPICYDAIHAGDLKSCTIEQLRDLQVGEKITFQLMRRRKGSMYIENHVAGLGETIERFPFVSAGEEAKRYS
KFLIAKRMDVAAIIERERNELLAESDVSCPEDVFIQQALVMLQERVEKLGVEKPDPKEDEEETAPVLCLETETKIDIEKS
DDASISSGEASSSLSYSSNHNKYYYFYQSNDGQNIYLHPLNVKMLQACYGTLDLGPLLIEAQIVQMEQHSMDEEHRRKFT
CLGHLPLTCQFSVVEVELQPPVVTGGILKLFKEDILHRKKERQRRDREERKREQHINEINDRQMGKLIASAANLDLSSSH
EFPTCGFEEALPAPSGSVPMNISNQDPGSRYSSVTLGSPKQEMWPTIGSNSPGGGGAPLDVQVGAWGRSAPPPPRAVMVP
RTSDEDFEQRSVGHWGLGELLVGALDQKKQKVGAAKSNGAAPAESKKQKKAKGKKMVPLFATGMNRAP

Domains Architecture of Danio rerio

No	***Accession Number***	***Domains***
1	NP_956538.1	FYVE zinc finger, PH domain
2	NP_956634.1	FYVE zinc finger, PH domain
3	XP_005164880.1	RhoGEF domain, PH domain, PH domain, FYVE zinc finger
4	XP_017210510.1	RhoGEF domain, PH domain, PH domain, FYVE zinc finger
5	XP_005164879.1	RhoGEF domain, PH domain, PH domain, FYVE zinc finger
6	NP_001093450.1	RhoGEF domain, PH domain, PH domain, FYVE zinc finger
7	XP_005155897.1	RhoGEF domain, FYVE zinc finger, PH domain, PH domain

8	XP_001919894.2	RhoGEF domain, PH domain, PH domain, FYVE zinc finger
9	XP_001332264.7	RhoGEF domain, FYVE zinc finger, PH domain
10	XP_021330870.1	RhoGEF domain, FYVE zinc finger, PH domain
11	NP_001171404.1	RhoGEF domain, PH domain, PH domain, FYVE zinc finger
12	XP_688794.4	RhoGEF domain, PH domain, PH domain, FYVE zinc finger
13	XP_017212899.1	RhoGEF domain, PH domain, PH domain, FYVE zinc finger
14	XP_017212897.1	RhoGEF domain, PH domain, PH domain, FYVE zinc finger
15	XP_017212898.1	RhoGEF domain, PH domain, PH domain, FYVE zinc finger
16	XP_002667361.2	RUN domain, FYVE zinc finger
17	NP_001099151.2	RUN domain, FYVE zinc finger
18	NP_001231848.1	RUN domain, FYVE zinc finger
19	XP_003200485.1	FYVE zinc finger
20	XP_001344504.2	Domain of unknown function (DUF3480), Smad anchor for receptor activation (SARA), FYVE zinc finger

21	XP_005171254.1	Domain of unknown function (DUF3480), Smad anchor for receptor activation (SARA), FYVE zinc finger
22	XP_021328582.1	FYVE zinc finger, FYVE zinc finger
23	XP_009304190.1	Domain of unknown function (DUF3480), FYVE zinc finger
24	NP_001071191.1	FYVE zinc finger
25	XP_005158933.1	FYVE zinc finger
26	XP_691596.6	FYVE zinc finger, FYVE zinc finger , Guanylate-binding protein, N-terminal domain
27	XP_009300450.1	Regulator of chromosome condensation (RCC1) repeats, MORN repeats, Regulator of chromosome condensation (RCC1) repeat, Vacuolar sorting protein 9 (VPS9) domain
28	XP_009300452.1	Regulator of chromosome condensation (RCC1) repeats, MORN repeats, Regulator of chromosome condensation (RCC1) repeat, Vacuolar sorting protein 9 (VPS9) domain
29	XP_009300451.1	Regulator of chromosome condensation (RCC1) repeats, MORN repeats, Regulator of chromosome condensation (RCC1) repeat, Vacuolar sorting protein 9 (VPS9) domain
30	XP_021333894.1	Myotubularin-like phosphatase domain, FYVE zinc finger
31	NP_957158.2	Myotubularin-like phosphatase domain, FYVE zinc finger
32	NP_956162.2	VHS domain, Hepatocyte growth factor-regulated tyrosine kinase substrate, FYVE zinc finger
33	XP_021325300.1	VHS domain, Hepatocyte growth factor-regulated tyrosine kinase substrate, FYVE zinc finger

34	XP_021325297.1	VHS domain, Hepatocyte growth factor-regulated tyrosine kinase substrate, FYVE zinc finger
35	XP_021325293.1	VHS domain, Hepatocyte growth factor-regulated tyrosine kinase substrate, FYVE zinc finger
36	XP_021325291.1	VHS domain, Hepatocyte growth factor-regulated tyrosine kinase substrate, FYVE zinc finger
37	XP_021332790.1	RhoGEF domain, PH domain, PH domain, FERM C-terminal PH-like domain, FERM central domain, FERM adjacent (FA),
38	XP_002667032.4	Myotubularin-like phosphatase domain, FYVE zinc finger
39	XP_021332402.1	Myotubularin-like phosphatase domain, FYVE zinc finger
40	XP_009299405.1	Myotubularin-like phosphatase domain, FYVE zinc finger
41	XP_005155593.1	Domain of unknown function (DUF3480), FYVE zinc finger
42	XP_021325289.1	VHS domain, Hepatocyte growth factor-regulated tyrosine kinase substrate, FYVE zinc finger
43	XP_005164430.1	VHS domain, Hepatocyte growth factor-regulated tyrosine kinase substrate, FYVE zinc finger
44	XP_021325286.1	VHS domain, Hepatocyte growth factor-regulated tyrosine kinase substrate, FYVE zinc finger
45	XP_021325285.1	VHS domain, Hepatocyte growth factor-regulated tyrosine kinase substrate, FYVE zinc finger
46	XP_005155592.1	Domain of unknown function (DUF3480), Smad anchor for receptor activation (SARA), FYVE zinc finger

47	XP_001920918.3	Domain of unknown function (DUF3480), Smad anchor for receptor activation (SARA), FYVE zinc finger
48	XP_005165797.1	Regulator of chromosome condensation (RCC1) repeats, MORN repeats, Regulator of chromosome condensation (RCC1) repeat, Vacuolar sorting protein 9 (VPS9) domain
49	NP_001096142.1	Ankyrin repeats (3 copies), BTB/POZ domain, FYVE zinc finger
50	XP_002662342.2	Regulator of chromosome condensation (RCC1) repeats, MORN repeats, Regulator of chromosome condensation (RCC1) repeat, Vacuolar sorting protein 9 (VPS9) domain
51	XP_005171962.1	Ankyrin repeats (3 copies), BTB/POZ domain, FYVE zinc finger
52	XP_005157097.1	Zinc finger FYVE domain-containing protein 21 C-terminus, FYVE zinc finger
53	NP_001153818.1	Zinc finger FYVE domain-containing protein 21 C-terminus, FYVE zinc finger
54	XP_021336159.1	FYVE zinc finger
55	NP_991308.1	WD domain, G-beta repeat, FYVE zinc finger
56	XP_009295365.1	FYVE zinc finger, RUN domain
57	XP_021332794.1	RhoGEF domain, FERM C-terminal PH-like domain, FERM central domain, FERM adjacent (FA), FERM N-terminal domain, PH
58	XP_021332793.1	RhoGEF domain, PH, FERM C-terminal PH-like domain, FERM central domain, FERM adjacent (FA), FERM N-terminal domain
59	XP_009300696.1	RhoGEF domain, PH, PH, FERM C-terminal PH-like domain, FERM central domain, FERM adjacent (FA), FERM N-terminal domain

60	XP_001344703.1	FYVE zinc finger, FYVE zinc finger
61	XP_021333893.1	Myotubularin-like phosphatase domain, FYVE zinc finger
62	XP_021332792.1	RhoGEF domain, PH, PH, FERM C-terminal PH-like domain, FERM central domain, FERM adjacent (FA), FERM N-terminal domain
63	XP_009302003.1	Myotubularin-like phosphatase domain, FYVE zinc finger
64	XP_017211771.1	Beige/BEACH domain, FYVE zinc finger, PH domain associated with Beige/BEACH, WD domain, G-beta repeat
65	XP_017211770.1	Beige/BEACH domain, FYVE zinc finger, PH domain associated with Beige/BEACH, WD domain, G-beta repeat
66	XP_017211768.1	Beige/BEACH domain, FYVE zinc finger, PH domain associated with Beige/BEACH, WD domain, G-beta repeat
67	XP_005165446.1	Beige/BEACH domain, FYVE zinc finger, PH domain associated with Beige/BEACH, WD domain, G-beta repeat
68	XP_017211769.1	Beige/BEACH domain, FYVE zinc finger, PH domain associated with Beige/BEACH, WD domain, G-beta repeat
69	XP_001921741.4	Beige/BEACH domain, FYVE zinc finger, PH domain associated with Beige/BEACH, WD domain, G-beta repeat
70	NP_001292489.1	FYVE zinc finger
71	XP_003199781.2	FYVE zinc finger
72	XP_021336195.1	FYVE zinc finger

73	XP_021336194.1	FYVE zinc finger
74	XP_005167727.1	Phosphatidylinositol-4-phosphate 5-Kinase, Phosphatidylinositol-4-phosphate 5-Kinase, TCP-1/cpn60 chaperonin family, Domain found in Dishevelled, Egl-10, and Pleckstrin (DEP), FYVE zinc finger
75	XP_005167726.1	Phosphatidylinositol-4-phosphate 5-Kinase, Phosphatidylinositol-4-phosphate 5-Kinase, TCP-1/cpn60 chaperonin family, Domain found in Dishevelled, Egl-10, and Pleckstrin (DEP), FYVE zinc finger
76	XP_005167724.1	Phosphatidylinositol-4-phosphate 5-Kinase, Phosphatidylinositol-4-phosphate 5-Kinase, TCP-1/cpn60 chaperonin family, Domain found in Dishevelled, Egl-10, and Pleckstrin (DEP), FYVE zinc finger
77	XP_009303000.1	Phosphatidylinositol-4-phosphate 5-Kinase, Phosphatidylinositol-4-phosphate 5-Kinase, TCP-1/cpn60 chaperonin family, Domain found in Dishevelled, Egl-10, and Pleckstrin (DEP), FYVE zinc finger
78	NP_001120777.1	Phosphatidylinositol-4-phosphate 5-Kinase, Phosphatidylinositol-4-phosphate 5-Kinase, TCP-1/cpn60 chaperonin family, Domain found in Dishevelled, Egl-10, and Pleckstrin (DEP), FYVE zinc finger
79	XP_005167722.1	Phosphatidylinositol-4-phosphate 5-Kinase, Phosphatidylinositol-4-phosphate 5-Kinase, TCP-1/cpn60 chaperonin family, Domain found in Dishevelled, Egl-10, and Pleckstrin (DEP), FYVE zinc finger
80	XP_009302997.1	Phosphatidylinositol-4-phosphate 5-Kinase, Phosphatidylinositol-4-phosphate 5-Kinase, TCP-1/cpn60 chaperonin family, Domain found in Dishevelled, Egl-10, and Pleckstrin (DEP), FYVE zinc finger
81	XP_009302999.1	Phosphatidylinositol-4-phosphate 5-Kinase, Phosphatidylinositol-4-phosphate 5-Kinase, TCP-1/cpn60 chaperonin family, Domain found in Dishevelled, Egl-10, and Pleckstrin (DEP), FYVE zinc finger
82	XP_009302996.1	Phosphatidylinositol-4-phosphate 5-Kinase, Phosphatidylinositol-4-phosphate 5-Kinase, TCP-1/cpn60 chaperonin family, Domain found in Dishevelled, Egl-10, and Pleckstrin (DEP), FYVE zinc finger

83	XP_009302998.1	Phosphatidylinositol-4-phosphate 5-Kinase, Phosphatidylinositol-4-phosphate 5-Kinase, TCP-1/cpn60 chaperonin family, Domain found in Dishevelled, Egl-10, and Pleckstrin (DEP), FYVE zinc finger
84	NP_001007058.2	WD domain, G-beta repeat, FYVE zinc finger
85	NP_571631.2	RhoGEF domain, PH, FYVE
86	XP_021332905.1	RhoGEF domain
87	XP_697706.2	Rabenosyn Rab binding domain, Rabenosyn Rab binding domain, FYVE zinc finger, Rabosyn-5 repeating NPF sequence-motif
88	XP_017211441.1	RhoGEF domain, FERM C-terminal PH-like domain, PH domain, PH domain, FERM N-terminal domain, FERM adjacent (FA), FERM central domain
89	XP_005171779.4	Myotubularin-like phosphatase domain, FYVE zinc finger

Retrieved Sequences of Danio rerio

>NP_956538.1 pleckstrin homology domain-containing family F member 2 [Danio rerio]
MVDRLANSEANSKRIGVVEACFGTAGQPLAIPGRVLIGEGVLTKLCRKRPKARQFFLFNDILVYGNIVIQKKKYNKQHII
PLESVTIDTVEDEGELRNGWLIKTPTKSFAVYAATATEKSEWMSHINKCVSDLLEKSGKSPTGEHAAVWVPDSEATVCMR
CQKMKFTPVNRRHHCRKCGFVVCGPCSEKKFLLPSQSSKPVRVCEFCYKQLSTGATLPPRSDSYSRQGSDFGSNNISDDD
DDDDSSD
>NP_956634.1 pleckstrin homology domain-containing family F member 1 [Danio rerio]
MAEHLAFTIQNRERIQAVESSFGRTGKMLQKPGRFLVGEGCLQKLCRRGPQPRVFYLFNDILVYGSIMLHGRWNNKQNII
PLEEVQQEDLEDGMAMANQWLIRTPRKSFYVSAESPEEKIAWMGHIEQYRTLHVKNKGLPAKKSGDDFATPWIPDVASAI
CMRCSKRFTVANRRHHCRRCGYIVCQACSKGRAVLPHISNRPVRVCRNCKNDMTDGMRQVQGKMRAKGNHWKKNSVED
TP

TMPEFENSSDEENENADECHQVPTKWFQSQEEDSFSAYCYIKPEHRNPPVGGH
>XP_005164880.1 FYVE, RhoGEF and PH domain-containing protein 6 isoform X2 [Danio rerio]
MSTGMKKPPVAPKPKLVPLHKPSPPPIAPKPEILLPSPSPTAHKRGKPAVAPKPCLLKAPQKPVQARQDHCKNQHPPVSK
NGGPALLSSNLSHYIIPPCNKVCHLANNKSNDSKEISREATLTTEVGVFKEGDSPKEHLFSNDTWEHTARSEHVQEQVPT
DSTQMSTEAQDETNSPSVEKDQTSTQVLTSDHIPRHCPTEPLQERSSEVKETGNVFSSCTSDSVGVPAPPSKPLPVPHPR
RPRRALLRQNVVEIAPSDTQADIPTETPENAQSDGLQETPKNTTCHSHTSTLQNACCPKEDIENTDTDSSTKSDPLHKVD
SFYNAQTEDMPSDLDSTHYSVPTNGATLASMDTAVEDGSQPPAPPPRQKSLPQMVDSLCKASTSVDNLLLHCQSDLQEVT
SNDEDAESDDEDDGAYGDFARYPITRSLPKQIKLSCGSQVIAITKPYIDGDEKSPKVMPKKPQRNSLPASVALRKQGTPP
PLPNSNPPVFGELPAPPQEKPSWRVALPSIPLFSRNQPTRSNSQPQAGVVGSVFVKQRAKSFSSADLQRVDNGSEASEQL
ARSDQTRRSLRKLLELRVCARLLPKLLRSGQSLDCTRTDSEYEEHKAVPANQVTSGDDEEAQGDSEADCGVEYENVPLYE
EIPEYMNLPWVYSNQDADTDVYEVQEPCEANRCSVSADLSEDGLSSNEEDGDSSDSSKEDMRHSKDKEEVERAKRNKVVH
IAMEIMSSEKVFVDVLKLLHIDFRDAVAKATRASGKPLVEEKVLNQILYYLPQLYELNKDLLKELEERVAHWSDHQRLAD
IFVQKGPYLKMYSTYIREFDRNVALLDEQCRKNPPFSSVVRQFETSPRCASLALKHYLLKPVQRIPQYQLLLTDYLKNLP
EDSSDYKDTQTALSVVKEVANHANDIMKQGDNFQKLMQVQYSLNGHHEIVQPGRVFLKEGTLMKLSRKVMQPRMFFLFN
D
ILLYTTPVQSGQYKVNSMLSLAGMKVSKPSQEAYQNELNIESVERSFILSANSATERDEWLEAIATAIDDYTRKKISFFS
SRSQELEGISDDGLPLGSKAPIWIPDLRTTMCMICTCEFTLTWRRHHCRACGKVVCQACSSNKFYLEYLKNQLARVCDHC
YIKLQHKGDQSNVTFSPSGRGSTFAFSRKQKKIPSALKEVSANTENSSMSGYLHRSKGHKKPWKRLWFVIKNKVLYTYAA
SEDVAALESQPLLGFFLREEKTGPAQKLQFKLYHKNTLYYIFRAEDIPTAQRWIEAFQEAMIL
>XP_017210510.1 FYVE, RhoGEF and PH domain-containing protein 6 isoform X3 [Danio rerio]
MKKPPVAPKPKLVPLHKPSPPPIAPKPEILLPSPSPTAHKRGKPAVAPKPCLLKAPQKPVQARQDHCKNQHPPVSKNGGP
ALLSSNLSHYIIPPCNKVCHLANNKSNDSKEISREATLTTEVGVFKEGDSPKEHLFSNDTWEHTARSEHVQEQVPTDSTQ
MSTEAQDETNSPSVEKDQTSTQVLTSDHIPRHCPTEPLQERSSEVKETGNVFSSCTSDSVGVPAPPSKPLPVPHPRRPRR
ALLRQNVVEIAPSDTQADIPTETPENAQSDGLQETPKNTTCHSHTSTLQNACCPKEDIENTDTDSSTKSDPLHKVDSFYN
AQTEDMPSDLDSTHYSVPTNGATLASMDTAVEDGSQPPAPPPRQKSLPQMVDSLCKASTSVDNLLLHCQSDLQEVTSNDE
DAESDDEDDGAYGDFARYPITRSLPKQIKLSCGSQVIAITKPYIDGDEKSPKVMPKKPQRNSLPASVALRKQGTPPPLPN
SNPPVFGELPAPPQEKPSWRVALPSIPLFSRNQPTRSNSQPQAGVVGSVFVKQRAKSFSSADLQRVDNGSEASEQLARSD
QTRRSLRKLLELRVCARLLPKLLRSGQSLDCTRTDSEYEEHKAVPANQVTSGDDEEAQGDSEADCGVEYENVPLYEEIPE
YMNLPWVYSNQDADTDVYEVQEPCEANSRCSVSADLSEDGLSSNEEDGDSSDSSKEDMRHSKDKEEVERAKRNKVVHIAM
EIMSSEKVFVDVLKLLHIDFRDAVAKATRASGKPLVEEKVLNQILYYLPQLYELNKDLLKELEERVAHWSDHQRLADIFV
QKGPYLKMYSTYIREFDRNVALLDEQCRKNPPFSSVVRQFETSPRCASLALKHYLLKPVQRIPQYQLLLTDYLKNLPEDS
SDYKDTQTALSVVKEVANHANDIMKQGDNFQKLMQVQYSLNGHHEIVQPGRVFLKEGTLMKLSRKVMQPRMFFLFNDILL
YTTPVQSGQYKVNSMLSLAGMKVSKPSQEAYQNELNIESVERSFILSANSATERDEWLEAIATAIDDYTRKKISFFSSRS

QELEGISDDGLPLGSKAPIWIPDLRTTMCMICTCEFTLTWRRHHCRACGKVVCQACSSNKFYLEYLKNQLARVCDHCYIK
LQHKGDQSNVTFSPSGRGSTFAFSRKQKKIPSALKEVSANTENSSMSGYLHRSKGHKKPWKRLWFVIKNKVLYTYAASED
VAALESQPLLGFFLREEKTGPAQKLQFKLYHKNTLYYIFRAEDIPTAQRWIEAFQEAMIL
>XP_005164879.1 FYVE, RhoGEF and PH domain-containing protein 6 isoform X1 [Danio rerio]
MSTGMKKPPVAPKPKLVPLHKPSPPPIAPKPEILLPSPSPTAHKRGKPAVAPKPCLLKAPQKPVQARQDHCKNQHPPVSK
NGGPALLSSNLSHYIIPPCNKVCHLANNKSNDSKEISREATLTTEVGVFKEGDSPKEHLFSNDTWEHTARSEHVQEQVPT
DSTQMSTEAQDETNSPSVEKDQTSTQVLTSDHIPRHCPTEPLQERSSEVKETGNVFSSCTSDSVGVPAPPSKPLPVPHPR
RPRRALLRQNVVEIAPSDTQADIPTETPENAQSDGLQETPKNTTCHSHTSTLQNACCPKEDIENTDTDSSTKSDPLHKVD
SFYNAQTEDMPSDLDSTHYSVPTNGATLASMDTAVEDGSQPPAPPPRQKSLPQMVDSLCKASTSVDNLLLHCQSDLQEVT
SNDEDAESDDEDDGAYGDFARYPITRSLPKQIKLSCGSQVIAITKPYIDGDEKSPKVMPKKPQRNSLPASVALRKQGTPP
PLPNSNPPVFGELPAPPQEKPSWRVALPSIPLFSRNQPTRSNSQPQAGVVGSVFVKQRAKSFSSADLQRVDNGSEASEQL
ARSDQTRRSLRKLLELRVCARLLPKLLRSGQSLDCTRTDSEYEEHKAVPANQVTSGDDEEAQGDSEADCGVEYENVPLYE
EIPEYMNLPWVYSNQDADTDVYEVQEPCEANSRCSVSADLSEDGLSSNEEDGDSSDSSKEDMRHSKDKEEVERAKRNKVV
HIAMEIMSSEKVFVDVLKLLHIDFRDAVAKATRASGKPLVEEKVLNQILYYLPQLYELNKDLLKELEERVAHWSDHQRLA
DIFVQKGPYLKMYSTYIREFDRNVALLDEQCRKNPPFSSVVRQFETSPRCASLALKHYLLKPVQRIPQYQLLLTDYLKNL
PEDSSDYKDTQTALSVVKEVANHANDIMKQGDNFQKLMQVQYSLNGHHEIVQPGRVFLKEGTLMKLSRKVMQPRMFFLFN
DILLYTTPVQSGQYKVNSMLSLAGMKVSKPSQEAYQNELNIESVERSFILSANSATERDEWLEAIATAIDDYTRKKISFF
SSRSQELEGISDDGLPLGSKAPIWIPDLRTTMCMICTCEFTLTWRRHHCRACGKVVCQACSSNKFYLEYLKNQLARVCDH
CYIKLQHKGDQSNVTFSPSGRGSTFAFSRKQKKIPSALKEVSANTENSSMSGYLHRSKGHKKPWKRLWFVIKNKVLYTYA
ASEDVAALESQPLLGFFLREEKTGPAQKLQFKLYHKNTLYYIFRAEDIPTAQRWIEAFQEAMIL
>NP_001093450.1 FYVE, RhoGEF and PH domain-containing protein 6 [Danio rerio]
MKKPPVAPKPKLVPLHKPSPPPIAPKPEILLPSPSPTAHKRGKPAVAPKPCLLKAPQKPVQARQDHCKNQHPPVSKNGGP
ALLSSNLSHYIIPPCNKVCHLANNKSNDSKEISREATLTTEVGVFKEGDSPKEHLFSNDTWEHTARSEHVQEQVPTDSTQ
MSTEAQDETNSPSVEKDQTSTQVLTSDHIPRHCPTEPLQERSSEVKETGNVFSSCTSDSVGVPAPPSKPLPVPHPRRPRR
ALLRQNVVEIAPSDTQADIPTETPENAQSDGLQETPKNTTCHSHTSTLQNACCPKEDIENTDTDSSTKSDPLHKVDSFYN
AQTEDMPSDLDSTHYSVPTNGATLASMDTAVEDGSQPPAPPPRQKSLPQMVDSLCKASTSVDNLLLHCQSDLQEVTSNDE
DAESDDEDDGAYGDFARYPITRSLPKQIKLSCGSQVIAITKPYIDGDEKSPKVMPKKPQRNSLPASVALRKQGTPPPLPN
SNPPVFGELPAPPQEKPSWRVALPSIPLFSRNQPTRSNSQPQAGVVGSVFVKQRAKSFSSADLQRVDNGSEASEQLARSD
QTRRSLRKLLELRVCARLLPKLLRSGQSLDCTRTDSEYEEHKAVPANQVTSGDDEEAQGDSEADCGVEYENVPLYEEIPE
YMNLPWVYSNQDADTDVYEVQEPSMTVEFVNAFLTFISHWLVCDELSVPAKQRCAELAVGFQMHRTLNQACGRVFGNVS
M
LPTNIYMSADLSEDGLSSNEEDGDSSDSSKEDMRHSKDKEEVERAKRNKVVHIAMEIMSSEKVFVDVLKLLHIDFRDAVA
KATRASGKPLVEEKVLNQILYYLPQLYELNKDLLKELEERVAHWSDHQRLADIFVQKGPYLKMYSTYIREFDRNVALLDE

QCRKNPPFSSVVRQFETSPRCASLALKHYLLKPVQRIPQYQLLLTDYLKNLPEDSSDYKDTQTALSVVKEVANHANDIMK
QGDNFQKLMQVQYSLNGHHEIVQPGRVFLKEGTLMKLSRKVMQPRMFFLFNDILLYTTPVQSGQYKVNSMLSLAGMKVSK
PSQEAYQNELNIESVERSFILSANSATERDEWLEAIATAIDDYTRKKISFFSSRSQELEGISDDGLPLGSKAPIWIPDLR
TTMCMICTCEFTLTWRRHHCRACGKVVCQACSSNKFYLEYLKNQLARVCDHCYIKLQHKGDQSNVTFSPSGRGSTFAFSR
KQKKIPSALKEVSANTENSSMSGYLHRSKGHKKPWKRLWFVIKNKVLYTYAASEDVAALESQPLLGFFLREEKTGPAQKL
QFKLYHKNTLYYIFRAEDIPTAQRWIEAFQEAMIL
>XP_005155897.1 FYVE, RhoGEF and PH domain-containing protein 5 isoform X2 [Danio rerio]
MSRPCPSTRGPKPPVAPKPRLASEVSHSIVGNGDITSSDRLELKQDNKSDVAQEESDKSRENCTDDQAENDLYDKEFAVN
YDTTCYDEIEDKLQMAVDETMGEDEDKNVTEIQHATTLSLSEAVDECLDSADEFHSKSADDIKPAESREVIYNVLEDTSA
CSENDTSTKACESQSPYEEIEEPPLNSFQCANGQYEEDIGAGANCVHHEAEEDGTGLSSKATNCSQPSLCEEYPYDIIGT
LDDNNTWQAERATKDPSGRFRFAEETEDAFEPYSVIEVVPSDLMCTADPETRCTDEESSNVKPMDSENTTEGTSNQEPYY
VSSEDVEDLEEEQAKLENGVSLPASEVQEMDKNEEVDDYADIEDHEGDDHQVECASSEDYVEIGDEDEPLNMERKTKGKS
VRERSARRRHDLLSQRNSCQPRLRLCNITVPADLDLGRTPELTNRVVFAHTTEAFEEDIEELDCHIVPFFEDSDTESDEH
IYEEAGFDSEGENFISLDRKSIVTRSRSLSGKVPGYVPETVPEETGTEYQSHDYYTVALDQNGDPHPKPSDEAEANRIIP
SLKPRRFLLSPRSLSVEGRDLQLSGHFEGEKSLREEYRLQRKDDTLSLPCVITSSGSFSQRSHQSSSGVSTPTSLVDIPP
PFELAYITKRPVTKSSPSLLIQHDSPDNPKKKKTSFKRFLALKFRKKSESKSRGDGSVRSSRSSSESSHHGPIRVLELDR
RSTSGSPKLQSHAGKPQRNSEIPTTFLLYKERQSRQGALKSYNSRGISRVESFEDRTRPPFMPLPLTKPRSISFPSADTS
DYENIPAMSSDYENIQIPHGRPTRAVTITEFFDDHNRTAAPANENDGYVDMNSFAGIENKSTTREQDTESAYTEPFPVCP
VSATVSNEEDHGRTSEEEDGCGDHCHDRQIDGRSRAYYVAKDLVDSEREHVKALKVLQEDFRAAVESGGDDNGPLIENGK
LDEIFSTLPQAYSLHSEILNELETRIKQWEDSPTVVDVILTRRAEFSSFATYISNYDRSMSVLDESCRKSQAFEDVVKQF
ETQNAEGVLVPLKHQLLRVIVRILQYRMLLTDYLNNLSPDSKEYEDTQAALVIVSEVADQVNDNLKHGENLLRLVHIDYS
VRGKRDLLQPGRVFVKEGTLMKVSRKSRQPRHLFLMNDVMLYTYPQQDGKYRLKNTLTLSGMKVSKPIIDNVLNTLRIEV
SDVTITLSASSLGEREDWFHTLSRAIADHAAGLNTFSSSSEAREKLWMSLGEAAPVLVPVSHVMMCMNCTSDFSLTLRRH
HCNACGKVVCRSCSRNRYPLKYLKDRMAKVCDHCYAELRKRGGDVPGSCGSVSPRTHRASRPLSAVFQSLQPPSLWRNRK
SSSPLTQVSVGAEGSTMSGSLQRRKKSKRRWKRLWFLLKDKDKVASESLSLQGFTVKLSDRSEGEDTDNVFQLYHKKTLY
YTFRADDQPTARRWVNAMEEATVL
>XP_001919894.2 FYVE, RhoGEF and PH domain-containing protein 5 isoform X1 [Danio rerio]
MSRPCPSTRGPKPPVAPKPRLASEVSHSIVGNGDITSSDRLELKQDNKSDVAQEESDKSRENCTDDQAENDLYDKEFAVN
YDTTCYDEIEDKLQMAVDETMGEDEDKNVTEIQHATTLSLSEAVDECLDSADEFHSKSADDIKPAESREVIYNVLEDTSA
CSENDTSTKACESQSPYEEIEEPPLNSFQCANGQYEEDIGAGANCVHHEAEEDGTGLSSKATNCSQPSLCEEYPYDIIGT
LDDNNTWQAERATKDPSGRFRFAEETEDAFEPYSVIEVVPSDLMCTADPETRCTDEESSNVKPMDSENTTEGTSNQEPYY
VSSEDVEDLEEEQAKLENGVSLPASEVQEMDKNEEVDDYADIEDHEGDDHQVECASSEDYVEIGDEDEPLNMERKTKGKS
VRERSARRRHDLLSQRNSCQPRLRLCNITVPADLDLGRTPELTNRVVFAHTTEAFEEDIEELDCHIVPFFEDSDTESDEH

IYEEAGFDSEGENFISLDRKSIVTRSRSLSGKVPGYVPETVPEETGTEYQSHDYYTVALDQNGDPHPKPSDEAEANRIIP
SLKPRRFLLSPRSLSVEGRDLQLSGHFEGEKSLREEYRLQRKDDTLSLPCVITSSGSFSQRSHQSSSGVSTPTSLVDIPP
PFELAYITKRPVTKSSPSLLIQHDSPDNPKKKKTSFKRFLALKFRKKSESKSRGDGSVRSSRSSSESSHHGPIRVLELDR
RSTSGSPKLQSHAGKPQRNSEIPTTFLLYKERQSRQGALKSYNSRGISRVESFEDRTRPPFMPLPLTKPRSISFPSADTS
DYENIPAMSSDYENIQIPHGRPTRAVTITEFFDDHNRTAAPANENDGYVDMNSFAGIENKSTTREQDTESAYTEPFPVCP
VSATVSNEEDHGRTSEEEDGCGDHCHDRQIDGRSRAYYVAKDLVDSEREHVKALKVLQEDFRAAVESGGDDNGPLIENGK
LDEIFSTLPQAYSLHSEILNELETRIKQWEDSPTVVDVILTRRAEFSSFATYISNYDRSMSVLDESCRKSQAFEDVVKQF
ETQNAEGVLVPLKHQLLRVIVRILQYRMLLTDYLNNLSPDSKEYEDTQAALVIVSEVADQVNDNLKHGENLLRLVHIDYS
VRGKRDLLQPGRVFVKEGTLMKVSRKSRQPRHLFLMNDVMLYTYPQQDGKYRLKNTLTLSGMKVSKPIIDNVLNTLRIEV
SDVTITLSASSLGEREDWFHTLSRAIADHAAGLNTFSSSSEAREKLWMSLGEAAPVLVPVSHVMMCMNCTSDFSLTLRRH
HCNACGKVVCRSCSRNRYPLKYLKDRMAKVCDHCYAELRKRGGDVPGSCGSVSPRTHRASRPLSAVFQSLQPPSLWRNRK
SSSPLTQVSVGAEGSTMSGSLQRRKKSKRRWKRLWFLLKDKVLYTFKAREDKVASESLSLQGFTVKLSDRSEGEDTDNVF
QLYHKKTLYYTFRADDQPTARRWVNAMEEATVL
>XP_001332264.7 FYVE, RhoGEF and PH domain-containing protein 4 isoform X1 [Danio rerio]
MSAVSRSKNVKHKSIDAEKKGNVLNSCSDLKHHVHWQKTRFRRAVGGRTSGSQGSAGAQVEDSASSFLKDASSVLRKSAV
YSVSAMPPPAVLPKPKFQCPPKISAANVIDGEQTDNDKTRERTTSDQSASSECAASSFSASDQSSGWDVWSGWWSEEVRG
QQEESGRGNGASVEKEEEQSRWKQDGLNEDMENDEDKPSVENDEDQQEQVNKLKLFKISSELLQTERAYVSRLHLLDQVF
CVRLTEEALKGSFPVDVVKGIFSNVGSIYTFHSQFLLPDLETRMSQWDSKPRVGDVLAQLAPFLRMYAEYVKNFDSAMEL
LKLWMERSTQFSAIIQDIQSQEVCGSLTLQHHMLEPVQRVPRYEMLLKDYLKKLPEDDEDRSQAQKSLNIISMAATHSNM
AIRKMENLKKLMEIYEMLGGEEDIVNPSNELIKEGQILKLAARNTSSMERYLFLFNNMLLYCVPKFSLVGQRFTVRTRVR
VEGMKVLETSNEDYPHTFQVSGKERTLELQASSQQDKEDWIKAFQETIEIFQQKNETFKSACKEATDEVSKEELGKRAPR
WIRDNEVTMCMKCKEPFNALTRRRHHCRACGYVVCYKCSDHKASLRYDSNKLNKVCKDCYHILTGRADAEEPVCGKKKG
I
LEIEAAQVSGNSFLCGFLQYSDRTKPSQRVWCVIPQHDALVLYLYGAPQDVKALCTIPLLGYQVEDVQRSVDHPPTGFRL
CQSKYIHCFTAETEEVKLRWLKVIRKAVIGEMPECQTPVDGDLVNGCHEGATDGT
>XP_021330870.1 FYVE, RhoGEF and PH domain-containing protein 4 isoform X2 [Danio rerio]
MFEKVRKSAYDVEEFRKTAFRRRSAGLNVEKKKKVRRGSVPGSQGSAGAQVEDSASSFLKDASSVLRKSAVYSVSAMPPP
AVLPKPKFQCPPKISAANVIDGEQTDNDKTRERTTSDQSASSECAASSFSASDQSSGWDVWSGWWSEEVRGQQEESGRGN
GASVEKEEEQSRWKQDGLNEDMENDEDKPSVENDEDQQEQVNKLKLFKISSELLQTERAYVSRLHLLDQVFCVRLTEEAL
KGSFPVDVVKGIFSNVGSIYTFHSQFLLPDLETRMSQWDSKPRVGDVLAQLAPFLRMYAEYVKNFDSAMELLKLWMERST
QFSAIIQDIQSQEVCGSLTLQHHMLEPVQRVPRYEMLLKDYLKKLPEDDEDRSQAQKSLNIISMAATHSNMAIRKMENLK
KLMEIYEMLGGEEDIVNPSNELIKEGQILKLAARNTSSMERYLFLFNNMLLYCVPKFSLVGQRFTVRTRVRVEGMKVLET
SNEDYPHTFQVSGKERTLELQASSQQDKEDWIKAFQETIEIFQQKNETFKSACKEATDEVSKEELGKRAPRWIRDNEVTM

CMKCKEPFNALTRRRHHCRACGYVVCYKCSDHKASLRYDSNKLNKVCKDCYHILTGRADAEEPVCGKKKGILEIEAAQVS
GNSFLCGFLQYSDRTKPSQRVWCVIPQHDALVLYLYGAPQDVKALCTIPLLGYQVEDVQRSVDHPPTGFRLCQSKYIHCF
TAETEEVKLRWLKVIRKAVIGEMPECQTPVDGDLVNGCHEGATDGT
>NP_001171404.1 FYVE, RhoGEF and PH domain-containing protein 4a [Danio rerio]
MQEGGLDGAIGKGRVSNLISRFEENRHTDGRREGTPPKQVSRSPNCSPPRKNYQRPREQTSDSSPHHALKPPNGVLAKMN
RDRDRERDMEGVTHAPVPIARINKQGLLNGDSVDLTKREITEEECVDSPDVHLNQAKEEEEQEKRKVSEQSDIVRMNSIP
EQKETNEQKLYKIANELLQTEKAYVARLNLLDKVFYTKLMEEARKDTFPVDVVKSIFSNITSINAFHSQFLLPDLEKRMG
EWTSTPRIGDILQKLTPFLKMYAEYVRNFDHAMDLLKQRIDRSPPFKAIILDIQSQEVCGNLTLQHHMLEPVQRVPRYEM
LLKDYLKKLPPEHMDRRDAEKSLEIIGMAATHSNTAIRKTENLKKLLEIYEMLGEEEDIVNPSNELIKEGHILKLAARNT
SAMDRYLFLFNNMLLYCVPKFSLVGQKFTVRTRIGIEGMNVKETFNEDYPHTFQVSGKERTLELQASSKLDKESWIKAFT
ETIDISLQKNETFKSAIKDTEEASDLNISELGKRAPRWIRDNEVTMCVKCKEGFNAITRRRHHCRACGYVVCWKCSDYKA
TLEYDGNKISKVCKHCYFILTGRTDSDDREGKKKGILEIEAAQFCGSSIMCGFLQYCERNKPWQKVWCVIPEKEALVLYL
YGAPQDVKAQSTIPLLGYHVEDNPRATDPPASFRLSQSKSIHSFAAESEELKQRWLKVIRMAVTGEVPESPEENSSAEIT
EELNSNGN
>XP_688794.4 FYVE, RhoGEF and PH domain-containing protein 1 isoform X1 [Danio rerio]
MQLNRPKSAHLISSSPHNSSSYSSQFKSMRFSYHISSGLPSPQPALRRTGSSSSSPRLLTKSLSLDPGPCLGAHETPQRL
CSDPGPLEPQHCNGTSENGPQDVPPPPPKTRPPLPGPKPQVPPKPPHLQQQNARRPRPPDKPLPPPPPSRPLPADPRQSR
ATMPRAEGSSSPTCVLSLIEKFEREQIIVVPDITGGVMCTPRVPDQQATPGSLSPQTSEFILPCTSLQEPLEAPLAEDEL
EKEEKEEEEEEDVDETEEDDSELGASCSDKRLSMESGYGASDKLLDNMEMTANQSEIPSDRLSLPPAQMDGKLANRDSGI
DSISSPSHSEELCFVGDEDRVGHEREICPCSPGPGISCSYVEGPDEEGAGGEEAGRDSEGDSDLEEGSGDESEMNPMALQ
PLPKAERQDSTEMSVQQRVFNIANELLHTEKAYVSRLHLLDQVFCAQLMEEARARSSFPCEMVMGIFSNICSIYCFHQQF
LLPALEKRMEEWDLNPRIGDILQKLAPFLKMYGEYVKNFDRAMELVNTWMQRSSQFKTIIHNIQKEEMCGNLTLQHHMLE
PVQRIPRYELLLKDYLHRLPEDADDFKDAQKSLELIATAAEHSNAAIRKMERMRKLLKVYELLGGEEDIVNPTNELIKEG
HILKLSAKNGTSQDRYLILFNDRLLYCVPKLRLIGQKFGVRARIDVDGMELKETSSMNVPRTFLVSGKQRSLELQARTEE
EKRDWIQAIQATIQRHEQTLETFRMLNCSFREEDLTPPNSPNCSELGKRAPTPIREKEVTLCMKCQEPFNSITKRRHHCK
ACGHVVCGKCSEFRARLLYDNNRANRVCIDCYTMLVGVPPSPASLSSSTQRRRSILEKQASVAAENSVLCSFLHHMEKGS
GRGWQKAWFVIPENEPLVLYIYGAPQDVKAQRSLPLIGFEVSLPESGDRLERRFAFKMSQSHLTVYFSADCEELQRRWME
VLSRAGRGEELQSNGPICEALEEEGEEPAPTGEST
>XP_017212899.1 FYVE, RhoGEF and PH domain-containing protein 1 isoform X4 [Danio rerio]
MTGFVFCCAMYMDKTSVLRGPCLGAHETPQRLCSDPGPLEPQHCNGTSENGPQDVPPPPPKTRPPLPGPKPQVPPKPPHL
QQQNARRPRPPDKPLPPPPPSRPLPADPRQSRATMPRAEGSSSPTCVLSLIEKFEREQIIVVPDITGGVMCTPRVPDQQA
TPGSLSPQTSEFILPCTSLQEPLEAPLAEDELEKEEKEEEEEEDVDETEEDDSELGASCSDKRLSMESGYGASDKLLDNM
EMTANQSEIPSDRLSLPPAQMDGKLANRDSGIDSISSPSHSEELCFVGDEDRVGHEREICPCSPGPGISCSYVEGPDEEG

AGGEEAGRDSEGDSDLEEGSGDESEMNPMALQPLPKAERQDSTEMSVQQRVFNIANELLHTEKAYVSRLHLLDQVFCAQL
MEEARARSSFPCEMVMGIFSNICSIYCFHQQFLLPALEKRMEEWDLNPRIGDILQKLAPFLKMYGEYVKNFDRAMELVNT
WMQRSSQFKTIIHNIQKEEMCGNLTLQHHMLEPVQRIPRYELLLKDYLHRLPEDADDFKDAQKSLELIATAAEHSNAAIR
KMERMRKLLKVYELLGGEEDIVNPTNELIKEGHILKLSAKNGTSQDRYLILFNDRLLYCVPKLRLIGQKFGVRARIDVDG
MELKETSSMNVPRTFLVSGKQRSLELQARTEEEKRDWIQAIQATIQRHEQTLETFRMLNCSFREEDLTPPNSPNCSELGK
RAPTPIREKEVTLCMKCQEPFNSITKRRHHCKACGHVVCGKCSEFRARLLYDNNRANRVCIDCYTMLVGVPPSPASLSSS
TQRRRSILEKQASVAAENSVLCSFLHHMEKGSGRGWQKAWFVIPENEPLVLYIYGAPQDVKAQRSLPLIGFEVSLPESGD
RLERRFAFKMSQSHLTVYFSADCEELQRRWMEVLSRAGRGEELQSNGPICEALEEEGEEPAPTGEST
>XP_017212897.1 FYVE, RhoGEF and PH domain-containing protein 1 isoform X2 [Danio rerio]
MQLNRPKSAHLISSSPHNSSSYSSQFKSMRFSYHISSGLPSPQPALRRTGPCLGAHETPQRLCSDPGPLEPQHCNGTSEN
GPQDVPPPPPKTRPPLPGPKPQVPPKPPHLQQQNARRPRPPDKPLPPPPPSRPLPADPRQSRATMPRAEGSSSPTCVLSL
IEKFEREQIIVVPDITGGVMCTPRVPDQQATPGSLSPQTSEFILPCTSLQEPLEAPLAEDELEKEEKEEEEEEDVDETEE
DDSELGASCSDKRLSMESGYGASDKLLDNMEMTANQSEIPSDRLSLPPAQMDGKLANRDSGIDSISSPSHSEELCFVGDE
DRVGHEREICPCSPGPGISCSYVEGPDEEGAGGEEAGRDSEGDSDLEEGSGDESEMNPMALQPLPKAERQDSTEMSVQQR
VFNIANELLHTEKAYVSRLHLLDQVFCAQLMEEARARSSFPCEMVMGIFSNICSIYCFHQQFLLPALEKRMEEWDLNPRI
GDILQKLAPFLKMYGEYVKNFDRAMELVNTWMQRSSQFKTIIHNIQKEEMCGNLTLQHHMLEPVQRIPRYELLLKDYLHR
LPEDADDFKDAQKSLELIATAAEHSNAAIRKMERMRKLLKVYELLGGEEDIVNPTNELIKEGHILKLSAKNGTSQDRYLI
LFNDRLLYCVPKLRLIGQKFGVRARIDVDGMELKETSSMNVPRTFLVSGKQRSLELQARTEEEKRDWIQAIQATIQRHEQ
TLETFRMLNCSFREEDLTPPNSPNCSELGKRAPTPIREKEVTLCMKCQEPFNSITKRRHHCKACGHVVCGKCSEFRARLL
YDNNRANRVCIDCYTMLVGVPPSPASLSSSTQRRRSILEKQASVAAENSVLCSFLHHMEKGSGRGWQKAWFVIPENEPLV
LYIYGAPQDVKAQRSLPLIGFEVSLPESGDRLERRFAFKMSQSHLTVYFSADCEELQRRWMEVLSRAGRGEELQSNGPIC
EALEEEGEEPAPTGEST
>XP_017212898.1 FYVE, RhoGEF and PH domain-containing protein 1 isoform X3 [Danio rerio]
MTGFVFCCAMYMDKTSVLRGSSSSSPRLLTKSLSLDPGPCLGAHETPQRLCSDPGPLEPQHCNGTSENGPQDVPPPPPKT
RPPLPGPKPQVPPKPPHLQQQNARRPRPPDKPLPPPPPSRPLPADPRQSRATMPRAEGSSSPTCVLSLIEKFEREQIIVV
PDITGGVMCTPRVPDQQATPGSLSPQTSEFILPCTSLQEPLEAPLAEDELEKEEKEEEEEEDVDETEEDDSELGASCSDK
RLSMESGYGASDKLLDNMEMTANQSEIPSDRLSLPPAQMDGKLANRDSGIDSISSPSHSEELCFVGDEDRVGHEREICPC
SPGPGISCSYVEGPDEEGAGGEEAGRDSEGDSDLEEGSGDESEMNPMALQPLPKAERQDSTEMSVQQRVFNIANELLHTE
KAYVSRLHLLDQVFCAQLMEEARARSSFPCEMVMGIFSNICSIYCFHQQFLLPALEKRMEEWDLNPRIGDILQKLAPFLK
MYGEYVKNFDRAMELVNTWMQRSSQFKTIIHNIQKEEMCGNLTLQHHMLEPVQRIPRYELLLKDYLHRLPEDADDFKDAQ
KSLELIATAAEHSNAAIRKMERMRKLLKVYELLGGEEDIVNPTNELIKEGHILKLSAKNGTSQDRYLILFNDRLLYCVPK
LRLIGQKFGVRARIDVDGMELKETSSMNVPRTFLVSGKQRSLELQARTEEEKRDWIQAIQATIQRHEQTLETFRMLNCSF
REEDLTPPNSPNCSELGKRAPTPIREKEVTLCMKCQEPFNSITKRRHHCKACGHVVCGKCSEFRARLLYDNNRANRVCID

CYTMLVGVPPSPASLSSSTQRRRSILEKQASVAAENSVLCSFLHHMEKGSGRGWQKAWFVIPENEPLVLYIYGAPQDVKA
QRSLPLIGFEVSLPESGDRLERRFAFKMSQSHLTVYFSADCEELQRRWMEVLSRAGRGEELQSNGPICEALEEEGEEPAP
TGEST
>XP_002667361.2 RUN and FYVE domain-containing protein 1 [Danio rerio]
MADEDPNTKVESGDVPAKEEEPEVTETGQEDVANNTDELQKDRPADSSWSAPILSFARKATETLSSGVNHYSAGLKTPDT
TQQHSSDPVKIAAVKDPVVVERSNLLSMMKLSIKVLIQSSLSLGRTLDSEYPPLQQFFVVLEHCLKHGLKVRKSFIGLQN
KSIWAPLEMIEKLCPESADIATSVRDMPGIKTGLGRARAWLHLALMQKKIADYMKVLINRRDLLGEFYEPGALVMEEEGA
VIVGMLVGLNVIDANLCVKGEDLDSQVCVIDFSMYLKDPQATESKQDDSKMTAILDQKHYIEELNRHLSCTVTDLQAKMD
SLEKTNSKLIEELTAATDRINALREEQEQLKQENANILQSSQRKEEVTLQDSQVELETYRQTRQGLDEMYSVVWKQYKEE
KRIRQELQKELELQIGLKQEMEVAMKLLEKDTHEKQDTLVALRQQLDQVKNLNLQMFNKAQASDRVAQKKEEDMVHLEK
K
MIQMEAAMKELEQRLQNSEDVQKNTDEQQNEIRTEMKGKVNALQKRLSDLDTLRAGLETELSAEKEQRQILQKELQREQD
NSAELRTQLQQLQGLQTELLDLRQEKQQLQQLCEEQEQALQEMGLHLSQSKLKMEDFKEVNKALKGHAWLKDDEATQCK
Q
CQKEFSISRRKHHCRNCGDIYCGNCSSNELALPSYPKPVRVCDVCHSLLLQRSSTGS
>NP_001099151.2 RUN and FYVE domain-containing protein 2 isoform 2 [Danio rerio]
MYSPQSLHRWGITHSESMERLAYSQAFRDPMAMERANLLNMAKLSIKGLIESALSFGRTLDSDYPPLQQFFVVMEHCLKH
GLKVKKSFLGYNKSLWGPLEMVEKLCPEAGEIAASVRDLPGLKTPLGRARAWLRLALMQKKLADYLRLLITRKDILSEFY
ESSAVMLEEEGAVIVGLLVGLNVIDANLCVKGEDLDTQVGVIDFSMYLKNDIDDYRSEERNGQIAAILDQKNYVEELNRQ
LNSTVQGLQGRVESLEKNNSKLIEELAIAKNNIIKLQEENHQLRTENSVILMKAQQRLAVTEGDLSCELDTYKQSRQGLD
EMYNEARRQLKEECQLRQDVENELVVQVSMKQEMEMAMKLLEKDIHEKQDTLIGLRHQLDEVKAINVEMYQKMQSSDDS
M
RQKNDMIARLEEKTNQITATMKQLEQRLQDAERERASAEDGVRKFKQDFTNKAESLQKQIVQREKQMTQLETDLKIERDW
RQTLQNELERERETITQLAAEAQQINGLKKEFHRLQDENTQLKGICEEQEHALEELGCKLSESKLKIEDIKEANKALQGG
QVWLKDKDATHCKLCEKEFSISRRKHHCRNCGEIFCNACSDNELPLPASPKPVRVCDTCHAMLLQRCSSNTT
>NP_001231848.1 RUN and FYVE domain-containing protein 2 isoform 1 [Danio rerio]
MATPAEQDLVLAEAEGNKERTQVFGILRLQEDKSAGEKASSPAMKSGDGRWQAPIFALARKASETFSGGIHVLPKVTEPK
TSPFTEEWGARAFRDPMAMERANLLNMAKLSIKGLIESALSFGRTLDSDYPPLQQFFVVMEHCLKHGLKVKKSFLGYNKS
LWGPLEMVEKLCPEAGEIAASVRDLPGLKTPLGRARAWLRLALMQKKLADYLRLLITRKDILSEFYESSAVMLEEEGAVI
VGLLVGLNVIDANLCVKGEDLDTQVGVIDFSMYLKNDIDDYRSEERNGQIAAILDQKNYVEELNRQLNSTVQGLQGRVES
LEKNNSKLIEELAIAKNNIIKLQEENHQLRTENSVILMKAQQRLAVTEGDLSCELDTYKQSRQGLDEMYNEARRQLKEEC
QLRQDVENELVVQVSMKQEMEMAMKLLEKDIHEKQDTLIGLRHQLDEVKAINVEMYQKMQSSDDSMRQKNDMIARLEEK
T

NQITATMKQLEQRLQDAERERASAEDGVRKFKQDFTNKAESLQKQIVQREKQMTQLETDLKIERDWRQTLQNELERERET
ITQLAAEAQQINGLKKEFHRLQDENTQLKGICEEQEHALEELGCKLSESKLKIEDIKEANKALQGGQVWLKDKDATHCKL
CEKEFSISRRKHHCRNCGEIFCNACSDNELPLPASPKPVRVCDTCHAMLLQRCSSNTT
>XP_003200485.1 early endosome antigen 1 [Danio rerio]
MLRRILQMTPGKGGSQSSESEQPSADMNNESSEGFICPQCMKSHNSAEELFKHYEVFHEPGDQPSPFSPGREDLTLLRQE
IQDLQASLKEERWFSEELKKELDKVQGRIATNKGLQSDGTSGEDAELEMRLNESETEKFNIKQMKDLFEQKAAQLATEIV
DIKSRYDEEKSMREALEQRLANLNQDMQKEKQEKEKLSAELLQRPGVEDVEVLKKELVQVQTLMDNMTREREEESERLKG
QYEQLQANFTTSEKTIAQLKAELEKGPQEAAVYTQQIHQLQSSLNNLQQQSQALSEKLSRKEKENQELEERLGHEQASKK
SLQANLHQKELELQESRARVSSGEAALSRAQAELTERGEEAARLRRELGELEKNQQELKAERKQLQQQREDKENQGLQQQ
SEISQLHAKLLEAERQVGELQGRLKEQRQLSGEKLKDREQQADLQLKLSRLEEELKESSTKSTDLQHQLDKSKQQHQEL
QTLQQSTNGKLREAQNDLEQVLRQIGDKDQKIQNLEALLQKSKESVSQLETEREDLCAKIQAGEGEAALLNQLQEKNHSL
QEQITQLTDKLKNQSESHKQAQDNLHEQVQEQKTHLRSAQDRCQGLETTVTELNTQLTESREKIAQLDTQLKAKTEMLLS
AEAAKNAQRADLESHLETAQNALQDKQQELSKVQAQLEEQARRLTEKQEQCSQLENGLKDSRDKLMTAEQRIETLQTQTK
KAEVELTELRSGREQAQKEQSSLKKQISELEMKTKELNRLLDSEKQGASTQQEELKKKSSALTETRQKLEKAEQERGALQ
ANLDKLSQEGQKQQAELDKKVQGLSSDLQKTQGEKETLVKEVATVKEALSKASKALKESQTQLDKEKKGSKATLEEKEKV
FQKVQQELQKNSEATSKELSNVKGLLEKSIEAEKSLQSQLTALNAQLKQCQDTLKEKEKSEQQLQAELKTRQGSFSQEVK
KLKTQVSELQASLAKKTEEEAKQKEQITALSKDLSSEKNRCAELQKTSDGTKESLTALQSDYYGKESELSAVRQDLKVCE
EKLVLAQEELVTNRNQLSAYETQIQELKTGHTAMEIDLSKRDEKIKQQVETLQKLQKQQGQTEEQLKKEKAQCEELRESQ
SALEKDKNKLSADLKTLAEKNEKELKDLQAAKQLLIQQKVEMQGKVEAAQASVEKEQREHQKTRDSIKQNEQQLKAETNK
IKQQLASEVKAKEEMVRQREEAEVKMSMQVTALNENVATLKREWQSSQRRVGELEKQTDELRGEIAVLEATVQNNQDERR
ALLERCVQGEGEMEKLQAKMLEMRRKLDDTTAAMQELGRENQSLQIKQSQSLTRKWTEDHEVQNCMACGKGFSVTVRKH
H
CRHCGNIFCAECSARNALTPSSKKPVRVCDNCFDELQG
>XP_001344504.2 zinc finger FYVE domain-containing protein 9 isoform X2 [Danio rerio]
MENYFQAEAFNLDKVLDEFEQNEDETDTPILSDAKWTQILAPPSHLLSLNPALTHSDLSPRDSPISFKPLLDTQTSDGPS
LVEVTRLNGKQGLGQQQEGKSEERPADVYSPPLPQPNIGKLVSTDGQLADLPSFNSGVLENGYPASPDHTVQSDIIRFND
DETNQPVIDKKCLKDTGPCELKLEQNGHISDAGRDFISTEEESEDKPSILKNGVKMETSKDESVSPSCKVGIRDNSNGLC
PEGSSSHKGVLHKSVNGGGELDCGRENDEGAFQNGVMVENENTEKDNSASNQEEKEKMLKTDHKGMHKESLEGERCLSD
G
LVEDLDGDAQNQLAVSSKEDSVTEEKEIEESKQETCDPVRQLESGRTGKLNNSRLQPVSIPYGGARPKQPVNLKLQIPQP
LSNQVQNEFGTNSKNKNMEPQSRTGGLDASSEATESGVVQINGEHGNGSSGLIIPSESPDNDLQAGQQGAFSKKPLSSLG
EVAPVWVPDSQAPICMKCEVKFTFTKRRHHCRACGKVFCAACCSLKSRLMYMDRKEARVCVTCHCALINAQTWENTGSG
C

NQSPNPNNPAEYCSTIPPLQQAQASGALSSPPPTVMVPVGVLKQPGSEGSLPREQRRVWFADGILPNGEATDSSKAPAAN
PNPSQPVATSQNSNKSSGANASEAVHGSGALTTSPVGSSFSLIPEDGLPPILISTGVKGDYAVEERPSEIALMQQLEEGC
PDPLVFVLNANLLAMVKLVNYVNRKCWYMTTKGMHAVGQAEVVVLLQCLPDEKTIPKDIFTHFVQLYREALAGNVLGHL
G
HSFFTQSFLGSKEHGGFLYVNPTFQTLQDLPLPNSPYLFGILVQKWETPWAKVFPIRLMLRLGAEYRFYPCPLFSVRFRK
PLFGETGHTIMNLLADFRNYQYTLPVVKGLVVDMEVRKTCIKIPSNRYNELMKAMNKSNEHVLAMGACFNDRADSHLVCV
QNDDGNYQTQAISIHHQPRKVTGASFFVFSGALKASSGYLAKTSIVEDGVMVQITAETMEALRQALREMKDFSIACGKAD
QEENQEHVQVQWVDDDHNVNKGVISPIDGKSMESVTSVKIFHGSEYKANGKVIKWTEVFFLRSEDQNNGLSDPADHSRLA
ENVARAFCMALCPHLKLLKEDGMAKLGLRVTLDSDQVGYLAGSNGQPLLPQYLSELDSALIPVIHSGACQLSEGPVVMEL
IFYILEIIS
>XP_005171254.1 zinc finger FYVE domain-containing protein 9 isoform X1 [Danio rerio]
MENYFQAEAFNLDKVLDEFEQNEDETDTPILSDAKWTQILAPPSHLLSLNPALTHSDLSPRDSPISFKPLLDTQTSDGPS
LVEVTRLNGKQGLGQQQEGKSEERPADVYSPPLPQPNIGKLVSTDGQLADLPSFNSGVLENGYPASPDHTVQSDIIRFND
DETNQPVIDKKCLKDTGPCELKLEQNGHISDAGRDFISTEEESEDKPSILKNGVKMETSKDESVSPSCKVGIRDNSNGLC
PEGSSSHKGVLHKSVNGGGELDCGRENDEGAFQNGVMVENENTEKDNSASNQEEKEKMLKTDHKGMHKESLEGERCLSD
G
LVEDLDGDAQNQLAVSSKEDSVTEEKEIEESKQETCDPVRQLESGRTGKLNNSRLQPVSIPYGGARPKQPVNLKLQIPQP
LSNQVQNEFGTNSKNKNMEPQSRTGGLDASSEATESGVVQINGEHGNGSSGLIIPSESPDNDLQAGQQGAFSKKPLSSLG
EVAPVWVPDSQAPICMKCEVKFTFTKRRHHCRACGKVFCAACCSLKSRLMYMDRKEARVCVTCHCALINAQTWENTGSG
C
NQSPNPNNPAEYCSTIPPLQQAQASGALSSPPPTVMVPVGVLKQPGSEGSLPREQRRVWFADGILPNGEATDSSKAPAAN
PNPSQPVATSQNSNKSSGANASEAVHGSGALTTSPVGSSFSLIPEDGLPPILISTGVKGGTGGHITDYAVEERPSEIALM
QQLEEGCPDPLVFVLNANLLAMVKLVNYVNRKCWYMTTKGMHAVGQAEVVVLLQCLPDEKTIPKDIFTHFVQLYREALA
G
NVLGHLGHSFFTQSFLGSKEHGGFLYVNPTFQTLQDLPLPNSPYLFGILVQKWETPWAKVFPIRLMLRLGAEYRFYPCPL
FSVRFRKPLFGETGHTIMNLLADFRNYQYTLPVVKGLVVDMEVRKTCIKIPSNRYNELMKAMNKSNEHVLAMGACFNDRA
DSHLVCVQNDDGNYQTQAISIHHQPRKVTGASFFVFSGALKASSGYLAKTSIVEDGVMVQITAETMEALRQALREMKDFS
IACGKADQEENQEHVQVQWVDDDHNVNKGVISPIDGKSMESVTSVKIFHGSEYKANGKVIKWTEVFFLRSEDQNNGLSDP
ADHSRLAENVARAFCMALCPHLKLLKEDGMAKLGLRVTLDSDQVGYLAGSNGQPLLPQYLSELDSALIPVIHSGACQLSE
GPVVMELIFYILEIIS
>XP_021328582.1 zinc finger FYVE domain-containing protein 1-like [Danio rerio]
MNHRKDGVPHMADGLCQYAHQYNNKVLICKKCYEGGREVIVIPKTSASTDNPWFGLAKYAWSGYVLECASCGIIYRSRQY

WMGNQDPESSVVRPEVKHVWQGTDAFQTDHQNAAQRVLDGMNYIIQSVTEYSSGPTKAVAAWLTDQVAPPYWKPNAEIS
E
CHGCKRVFEEVERKHHCRACGQGFCQACSTHSMPVPERGWGGTPVRVCDACYRQGGPQHCGQVSSTEPRTLVARKVTEV
A
QSTLDMVSTAVDYPLCFIKGVARPDYWVPDHEITQCHCCAKVFTPSMSKHHCRACGEGVCGNCSSKTRAVPSRGWDHPVR
VCDTCAEIRDSL
>XP_009304190.1 zinc finger FYVE domain-containing protein 9 [Danio rerio]
MRQAVTATLSVWGERRFGYSTLKDSSESLLDERKLRPGLKEDSVIEEKERAENEQEQLEAVLGPPSSPVEDTHQSGTTEP
SDAMLESCQDQKTSDGMEASLVSGPKNIDSGRKGSLGALPVQRCSSLGTTAPQWVPDSQAPACMKCGSKFSFTKRRHHCR
ACGKVFCVVCCDLRYKLTHLGGKEGRVCVTCHSTLINRTLRKDQKKVWFADNVLPQSESRSANSSPIHRSVSHTQSAGLA
QADVSDLRAADEVSGLSSNTKPTPPKDLPPILTSTGVKGDYTLEEKRTESSLLEELERGLAEPLVFVLNANLLAVVKIVS
YVNRRCWCVMSKGMHAVGQPEVVILLQCLPEEKRFPTDIFNHFIQIYWDAQTAGKLLNHLSHSLVSRGFLGNNEHAGFVY
VRPTFQSLEGLPLPAPPFLFGLLMLRAEVPWAKAFPLRLMLRLGAEYRFYPCPLFSVRFREPLFGPVNNSIMRLLVDFRF
YRYTLPTVPGLVVDLEARSTCIRIPKTHHPELLKALNKSNERVLALGACFNEQADSHLICVQTADGQYQTQAISIHSQPR
RVTGSCFFVFSGALKPSSGYLAKSSIVEDGLMVQITMETMAELRRCLRDMKDFTVTCGKLHPAERQEYVHIQWQDEEPRF
NKGIVSPIDGKSMESLTNIKTHQRLEFRANGKLIRWTEVYYLLKDQHPNGLNNHTSHNRLTERLARAFCLALCQSLCLLK
EDGMTKLALRVILDGQKMEFLAGSNGQRLPAPYQDCLNQSLMSVVQSNSQYQGLAACQLELIFYILEDTS
>NP_001071191.1 lateral signaling target protein 2 homolog [Danio rerio]
MMNRFRKWLYKPKRSDPQLLAQFYYADEELNQVATELDSLDGRKDPQRCTLLVNQFRSCQDNVLNIINQIMDECIPEERA
NRDFCVKFPEEIRHDNLAGQLWFGAECLAAGSIIMNREIESMAMRPLAKDLTRSLEEVRNITRDQALRDLNHYTERIKEA
LRHFDGLFAEFELSYVSAMVPVKSPKEYYIQQEVIVLFCETVERALKLEYLTQDMIDDYEPALMFTIPRLAIVCGLVIYS
EGPLNLDRKPEDMSELFRPFRTLLRKIRDLLQTLTEEELMTLERSLCISQDGEFPTSSTNDPSASTGPDSQTEELEKEKG
VEEVVDLTLFVTQEDSVWKEEEEKQVLPESSSESEEEEPIDADLACSMQYDEEEIEQLNMMVHQVGDEMSTLLSPPSQNQ
SPAHRPRPYNGSSLEGSSATSSTQASPRRAPGSYHDDDRVFFMDDLESGLSSELCRGQLPLPTVCLRSPEGSSCNGWLTV
CQSSDATNLGCQRKLSQSTESVGNSDRMVNGWEGLQDEDSVQTAEEIANRTGGMKLSATVIFNPHSPSLSDLAVVLPQSA
DAPEGGEGGALVATQCLLNSCVCCAGGCVDNHEDAMEPAGRSMALGFEKHKLTITSSVIQSAVAAGSPGKGNGHLPLTLP
PSQGHLTHSVPNCSVQNQAREDEGSQDGIHYPCCEKCSPGVLLAQDRGSGHEGGPSCTLQDTGCQTQHNASVKGRSECFG
KQSKDDNRKINSSSQESPLSSVPSSDIDGVSVTTCSLSSSYAPSPVSSLTTSSDMSEDLDHQEIQVALQAAKLAAHNKIR
SRFHSSSDLIHRLFVCISGVADQLQTNYASDLRSILKTLFEVMATKTDQGDNEKPKKGPCLGSAVLEDCALCQETISSSE
LAAKAREGQFEDPPEWVPDEACNSCIACKAPFTVIRRKHHCRSCGKIFCSRCSSHSAPLPRYGQMKPVRVCTHCYMFHVT
PFYSDRTGI
>XP_005158933.1 lateral signaling target protein 2 homolog isoform X1 [Danio rerio]
MMNRFRKWLYKPKRSDPQLLAQFYYADEELNQVATELDSLDGRKDPQRCTLLVNQFRSCQDNVLNIINQIMDECIPEERA

NRDFCVKFPEEIRHDNLAGQLWFGAECLAAGSIIMNREIESMAMRPLAKDLTRSLEEVRNITRDQALRDLNHYTERIKEA
LRHFDGLFAEFELSYVSAMVPVKSPKEYYIQQEVIVLFCETVERALKLEYLTQDMIDDYEPALMFTIPRLAIVCGLVIYS
EGPLNLDRKPEDMSELFRPFRTLLRKIRDLLQTLTEEELMTLERSLCISQDGEFPTSSTNDPSASTGPDSQTEELEKEKG
VEEVVDLTLFVTQEDSVWKEEEEKQVLPESSSESEEEEPIDADLACSMQYDEEEIEQLNMMVHQVGDEMSTLLSPPSQNQ
SPAHRPRPYNGSSLEGSSATSSTQASPRRAPGSYHDDDRVFFMDDLESGLSSELCRGQLPLPTVCLRSPEGSHCRPDASC
NGWLTVCQSSDATNLGCQRKLSQSTESVGNSDRMVNGWEGLQDEDSVQTAEEIANRTGGMKLSATVIFNPHSPSLSDLAV
VLPQSADAPEAGEGGALVATQCLLNSCVCCAGGCVDNHEDAMEPAGRSMALGFEKHKLTITSSVIQSAVAAGSPGKGNGH
LPLTLPPSQGHLTHSVPNCSVQNQAREDEGSQDGIHYPCCEKCSPGVLLAQDRGSGQEGGPSCTLQDTGCRTQHNASVKG
KSECFGKQSKDDNRKINSPVSSLTTSSDMSEDLDHQEIQVALQAAKLAAHNKIRSRFHSSSDLIHRLFVCISGVADQLQT
NYASDLRSILKTLFEVMATKTDQGDNEKPKKGPCLGSAVLEDCALCQETISSSELAAKAREGQFEDPPEWVPDEACNSCI
ACKAPFTVIRRKHHCRSCGKIFCSRCSSHSAPLPRYGQMKPVRVCTHCYMFHVTPFYSDRTGI
>XP_691596.6 zinc finger FYVE domain-containing protein 1 isoform X1 [Danio rerio]
MSEMLLKDMDEISLGPVKMDEQKENGRSFLLVDENESLQVKNEAHFLQKLGCSGAQGVKVVSIFGNTGDGKSHTLNHVLF
DREEVFATSPSPSSCTVGVWAAYDPQLNLIVLDTEGLLGAATQQNRRMRLLLKVLAVSDVVIYRTRAERLHNDMFQFLGS
ASAAYLKHFTPELRALSSRCGLDVPLSSLGPAIIVFQETSRTQLLGQDVSGLGQADTLLQKRFHDLGQSTDAFSSVQYVG
TQTITPPTDYSQLQHTVQQQVSNTSQRAPRQPAIVFSALQALSDRFCGEISDDKFSICSFFPDEYFTCPSVCLSCNVRCK
NGMNHRKDGVPHMADGLCQYAHQYNNKVLICKKCYEGGREVIVIPKTSASTDNPWFGLAKYAWSGYVLECASCGIIYRSR
QYWMGNQDPESSVVRPEVKHVWQGTDAFQTDHQNAAQRVLDGMNYIIQSVTEYSSGPTKAVAAWLTDQVAPPYWKPNA
EI
SECHGCKRVFEEVERKHHCRACGQGFCQACSTHSMPVPERGWGGTPVRVCDACYRQGGPQHCGQVSSTEPRTLVARKVTE
VAQSTLDMVSTAVDYPLCFIKGVARPDYWVPDHEITQCHCCAKVFTPSMSKHHCRACGEGVCGNCSSKTRAVPSRGWDHP
VRVCYTCAEIRDSL
>XP_009300450.1 alsin isoform X1 [Danio rerio]
MEKLISSGEGDSSGERGLLHTWKGYSYSTAPERVLLSRPVLQAALGARHGVLLVEGGQVYSFGELPWKQNQTSSAEPILE
AVLSEQRVVFVAAGSAHSGVVTEDGGVHMWGENVHGQCGLLGLSVIPNPTPVGVLDSEATPPQTVKILEVACGDQHTLAL
SAKHEVWAWGSGCQLGLNTSTFPVWKPQKVDHLSGRHVLQVACGSAHSVALVRCLPPPQEPRKPPQDKCGQCHQLLYTM
T
DKEDHVIISDGHHCPLGVEMDSTEIKSAVEGSPDPGKPGKSSPTEPVPCMSTQISKSPTSSNISESTRPPESTLPFELQN
QVPAYCQESIEAPVEDSAAHEAPAAAGEVCVEASENSIPEPDSCRSSGKSPYPDEQAVKAYLKRLSDHALAEHTIKTSST
IHSAQLSGDPANLFASEPLIPVAQSVTPMGSALNNLVVSCASAVGERVVSTYEALSLRKVIGYWVPGEGRERVEERLRLE
ESMQGKKSSSLGDIREEEAELSRRLSLPGLLSQDNAESEVKASSSNTTTEVSPRLLRRTSRPRMRAVPLASGGIPETDAH
LPSLQTEVWSWGQGQDGQLGHGDLLPRLQPDCVKSLNGKEVLKVAAGAHHSLALTAQSQVFSWGCNTSGQLGHMESPTT
F

PQLTKLSDGIRVWDIGAGLQHTLLLADGDCIQPILYYSGQQVKEVPEPTEEEEQTAEYTQQPVLLPFCMNLGYVSSVYAG
GRTCAALTDCNVMGFIASLHELAAAERKYYCKLSNIKSLLLQPLLKLDSLSSALGQSSAGLLQNLIGRMSRLSQLTGQNS
ASLTSFLQRSRDIRGLVILEDAHLFLDTYSEYSSAVGDLLVMGGFQALVKPCHDVFGKGAEVVQRLSECSEEGLTLADVL
AHLFYLPIKHLHEYGRLLLKLATCYEVSSVDYQKLQDACSKFESMALHLKRRRKEAEYTLHFWKSFPGKMTDSLRKPSRR
LICESSNKALTLQNAGRFSVNWFILFNDALVHAQGMVPYKSLFSTHHIFPLATLWVEPISEESTGVYGLKVTSPEESFAL
LASSPAEKAKWLRSINQAVEQALSGLGHDGIPPSTGMTQRADPPISRTASYTFYKDSRLKDAKYDGRWVSGKPHGRGVVK
WPDGRMYTGTFKNGFEDGFGDYVVSNKTLNSCDHYQGQWKDGKMHGFGTFRYASGEVYEGSFQDNMRHGHGMLRSGK
LNS
TSPSVFIGQWQYDKKSGYGVFDDITRGEKYMGMWLDDQRQGNGVVVTQFGLYYEGAFSNNKMMGTGVLLSEDDTTFEGE
F
LEDWTLNGKGVLTMPNGDYIEGSFCGVWGTGLKMSGSYYKPSLYDSDKEKGHALKLGRLAVHSEEKWKAVFLECWNRLG
C
DSPVQGQTTTAWDNIAIALTANLRQQRDSPELLSRSHHKTLESLECIPQHVGPVTLEVYHTIRRYLVKACDTPLHPLGRL
VETLVSVYRMTYVGVGANRRLLPQAVNEITSYLSRIFQLVRFLFPELPEEGGSLADPPKEKVGTDADGKLLESPKPGCVV
SSSALLLPVLLPRLYPPLFTLYALEKEKEDDVYWECVLRLNKQPDLALLAFLGVQQKFWPVTVTIHGEKQQVLSSTKDAC
FASAVETLQQISTTFTPSDKLQVIQLTFEEITQEVLALLKQDFLWSMDDLFPVFLFVVLRARIRNLGSEVSLIEDLMDPC
VQHGEHGIMFTTLKACYYQIQHEKVT
>XP_009300452.1 alsin isoform X4 [Danio rerio]
MEKLISSGEGDSSGERGLLHTWKGYSYSTAPERVLLSRPVLQAALGARHGVLLVEGGQVYSFGELPWKQNQTSSAEPILE
AVLSEQRVVFVAAGSAHSGVVTEDGGVHMWGENVHGQCGLLGLSVIPNPTPVGVLDSEATPPQTVKILEVACGDQHTLAL
SAKHEVWAWGSGCQLGLNTSTFPVWKPQKVDHLSGRHVLQVACGSAHSVALVRCLPPPQEPRKPPQDKCGQCHQLLYTM
T
DKEDHVIISDGHHCPLGVEMDSTEIKSAVEGSPDPGKPGKSSPTEPVPCMSTQISKSPTSSNISESTRPPESTLPFELQN
QVPAYCQESIEAPVEDSAAHEAPAAAGEVCVEASENSIPEPDSCRSSGKSPYPDEQAVKAYLKRLSDHALAEHTIKTSST
IHSAQLSGDPANLFASEPLIPVAQSVTPMGSALNNLVVSCASAVGERVVSTYEALSLRKVIGYWVPGEGRERVEERLRLE
ESMQGKKSSSLGDIREEEAELSRRLSLPGLLSQVSPRLLRRTSRPRMRAVPLASGGIPETDAHLPSLQTEVWSWGQGQDG
QLGHGDLLPRLQPDCVKSLNGKEVLKVAAGAHHSLALTAQSQVFSWGCNTSGQLGHMESPTTFPQLTKLSDGIRVWDIGA
GLQHTLLLADGDCIQPILYYSGQQVKEVPEPTEEEEQTAEYTQQPVLLPFCMNLGYVSSVYAGGRTCAALTDCNVMGFIA
SLHELAAAERKYYCKLSNIKSLLLQPLLKLDSLSSALGQSSAGLLQNLIGRMSRLSQLTGQNSASLTSFLQRSRDIRGLV
ILEDAHLFLDTYSEYSSAVGDLLVMGGFQALVKPCHDVFGKGAEVVQRLSECSEEGLTLADVLAHLFYLPIKHLHEYGRL
LLKLATCYEVSSVDYQKLQDACSKFESMALHLKRRRKEAEYTLHFWKSFPGKMTDSLRKPSRRLICESSNKALTLQNAGR
FSVNWFILFNDALVHAQGMVPYKSLFSTHHIFPLATLWVEPISEESTGVYGLKVTSPEESFALLASSPAEKAKWLRSINQ
AVEQALSGLGHDGIPPSTGMTQRADPPISRTASYTFYKDSRLKDAKYDGRWVSGKPHGRGVVKWPDGRMYTGTFKNGFED

GFGDYVVSNKTLNSCDHYQGQWKDGKMHGFGTFRYASGEVYEGSFQDNMRHGHGMLRSGKLNSTSPSVFIGQWQYDKK
SG
YGVFDDITRGEKYMGMWLDDQRQGNGVVVTQFGLYYEGAFSNNKMMGTGVLLSEDDTTFEGEFLEDWTLNGKGVLTMP
NG
DYIEGSFCGVWGTGLKMSGSYYKPSLYDSDKEKGHALKLGRLAVHSEEKWKAVFLECWNRLGCDSPVQGQTTTAWDNIAI
ALTANLRQQRDSPELLSRSHHKTLESLECIPQHVGPVTLEVYHTIRRYLVKACDTPLHPLGRLVETLVSVYRMTYVGVGA
NRRLLPQAVNEITSYLSRIFQLVRFLFPELPEEGGSLADPPKEKVGTDADGKLLESPKPGCVVSSSALLLPVLLPRLYPP
LFTLYALEKEKEDDVYWECVLRLNKQPDLALLAFLGVQQKFWPVTVTIHGEKQQVLSSTKDACFASAVETLQQISTTFTP
SDKLQVIQLTFEEITQEVLALLKQDFLWSMDDLFPVFLFVVLRARIRNLGSEVSLIEDLMDPCVQHGEHGIMFTTLKACY
YQIQHEKVT
>XP_009300451.1 alsin isoform X2 [Danio rerio]
MEKLISSGEGDSSGERGLLHTWKGYSYSTAPERVLLSRPVLQAALGARHGVLLVEGGQVYSFGELPWKQNQTSSAEPILE
AVLSEQRVVFVAAGSAHSGVVTEDGGVHMWGENVHGQCGLLGLSVIPNPTPVGVLDSEATPPQTVKILEVACGDQHTLAL
SAKHEVWAWGSGCQLGLNTSTFPVWKPQKVDHLSGRHVLQVACGSAHSVALVRCLPPPQEPRKPPQDKCGQCHQLLYTM
T
DKEDHVIISDGHHCPLGVEMDSTEIKSAVEGSPDPGKPGKSSPTEPVPCMSTQISKSPTSSNISESTRPPESTLPFELQN
QVPAYCQESIEAPVEDSAAHEAPAAAGEVCVEASENSIPEPDSCRSSGKSPYPDEQAVKAYLKRLSDHALAEHTIKTSST
IHSAQLSGDPANLFASEPLIPVAQSVTPMGSALNNLVVSCASAVGERVVSTYEALSLRKVIGYWVPGEGRERVEERLRLE
ESMQGKKSSSLGDIREEEAELSRRLSLPGLLSQGTHALLLLHPVSRFLSPRLLRRTSRPRMRAVPLASGGIPETDAHLPS
LQTEVWSWGQGQDGQLGHGDLLPRLQPDCVKSLNGKEVLKVAAGAHHSLALTAQSQVFSWGCNTSGQLGHMESPTTFPQ
L
TKLSDGIRVWDIGAGLQHTLLLADGDCIQPILYYSGQQVKEVPEPTEEEEQTAEYTQQPVLLPFCMNLGYVSSVYAGGRT
CAALTDCNVMGFIASLHELAAAERKYYCKLSNIKSLLLQPLLKLDSLSSALGQSSAGLLQNLIGRMSRLSQLTGQNSASL
TSFLQRSRDIRGLVILEDAHLFLDTYSEYSSAVGDLLVMGGFQALVKPCHDVFGKGAEVVQRLSECSEEGLTLADVLAHL
FYLPIKHLHEYGRLLLKLATCYEVSSVDYQKLQDACSKFESMALHLKRRRKEAEYTLHFWKSFPGKMTDSLRKPSRRLIC
ESSNKALTLQNAGRFSVNWFILFNDALVHAQGMVPYKSLFSTHHIFPLATLWVEPISEESTGVYGLKVTSPEESFALLAS
SPAEKAKWLRSINQAVEQALSGLGHDGIPPSTGMTQRADPPISRTASYTFYKDSRLKDAKYDGRWVSGKPHGRGVVKWPD
GRMYTGTFKNGFEDGFGDYVVSNKTLNSCDHYQGQWKDGKMHGFGTFRYASGEVYEGSFQDNMRHGHGMLRSGKLNST
SP
SVFIGQWQYDKKSGYGVFDDITRGEKYMGMWLDDQRQGNGVVVTQFGLYYEGAFSNNKMMGTGVLLSEDDTTFEGEFLE
D
WTLNGKGVLTMPNGDYIEGSFCGVWGTGLKMSGSYYKPSLYDSDKEKGHALKLGRLAVHSEEKWKAVFLECWNRLGCDS
P

VQGQTTTAWDNIAIALTANLRQQRDSPELLSRSHHKTLESLECIPQHVGPVTLEVYHTIRRYLVKACDTPLHPLGRLVET
LVSVYRMTYVGVGANRRLLPQAVNEITSYLSRIFQLVRFLFPELPEEGGSLADPPKEKVGTDADGKLLESPKPGCVVSSS
ALLLPVLLPRLYPPLFTLYALEKEKEDDVYWECVLRLNKQPDLALLAFLGVQQKFWPVTVTIHGEKQQVLSSTKDACFAS
AVETLQQISTTFTPSDKLQVIQLTFEEITQEVLALLKQDFLWSMDDLFPVFLFVVLRARIRNLGSEVSLIEDLMDPCVQH
GEHGIMFTTLKACYYQIQHEKVT
>XP_021333894.1 myotubularin-related protein 3 isoform X3 [Danio rerio]
MEEEGQQSLECIQANQIFPRKPPVLEEEDLQVPFPELHGEFTEYVGRAEDAVIAMSSYRLHIKFKESVVNNCSCEVSVPL
QLIECVECRDMFQLHITCKDCKIVRCQFSTFEQCQEWLKRLTAAVRPPSRIEELFSFAFHAWCVDLYTGEKEQHGDLCRP
GDHVMSRFHNEVERMGFDTQNAWRVSEINNKYKLCSSYPQLLLVPAWITDKELENVAAFRSWKRIPAVVYRHQSTGAVIA
RCGQPEVSWWGWRNADDEHLVQSIARACAVDSSSCKGVSNGSLSREYTNGADLSDVDFESSMTNSSEVETLAIQPRKLLI
LDARSYAAAVANRAKGGGCECPEYYPNCEVVFMGMANIHSIRKSFQSLRFLCTQMPDPANWLSALESTKWLQHLSLLLKA
SLLVSNAVDRDHRPVLVHCSDGWDRTPQIVALAKLLLDPYYRTIEGFQVLVETEWLDFGHKFADRCGHGENAEDLNERCP
VFLQWLDCVHQLQRQFPCSFEFNEAFLVKLVQHTYSCLFGTFLCNSGKEREDRRIQERTCSVWSLLRAANRSFGNMLYSS
HSETVLHPVCHVRNLMLWTAVYLPSSSPTTPSDDSCAPYPAPGANPEDQPLGRRPKTRSFDNLPSACEVGNPLTSNRRSS
DPSLNEKWQDHRRSLELNIGAGTDGAASPEPDERVNGQTMVGIMGTFSNGGTENGEGLKDVRLPMIDGVDEAELTVGVAV
GQMENILQEATKDESAPDTRSVAKPNETSAVNGKTDDIAQEGRTEDDLESKSLEDGNVNGHCSEDGNSEASSALSQDTSE
SQDSEEIEKCTTQEDDSSCNPTILTPNGLRTLTNGHGVEPSIEQAEKRLSLLESSTETLTEDLGVRPESLGPLSIPPPLK
ALAESSCLKQGLRTAARTLNNTPKRASLSAFPPASTDLLHPMCNGDSPDPEPSTPRTNGERATLSRQVSLASCGSLTLHA
RGTCSHHRCLHLGFLGRPSFSPPEPPSSSSRSHLDDDGLTLHTDAIQQRLRQIEAGHQLEVEALKRQVQELWSRLESHQA
AGMLRLNGDIGDEVTSITDSDFNLEPNCLSRCSTELFSEASWEQVDKQDTEVTRWYPDHLAAQCYGCERGFWLATRKHHC
RNCGNVFCGSCCDQKIPVPSQQLFEPSRVCRSCFSNLQPPAPPLDLELDKPITASSN
>NP_957158.2 myotubularin-related protein 3 [Danio rerio]
MEEEGQQSLECIQANQIFPRKPPVLEEEDLQVPFPELHGEFTEYVGRAEDAVIAMSSYRLHIKFKESVVNVPLQLIECVE
CRDMFQLHITCKDCKIVRCQFSTFEQCQEWLKRLTAAVRPPSRIEELFSFAFHAWCVDLYTGEKEQHGDLCRPGDHVMSR
FHNEVERMGFDTQNAWRVSEINNKYKLCSSYPQLLLVPAWITDKELENVAAFRSWKRIPAVVYRHQSTGAVIARCGQPEV
SWWGWRNADDEHLVQSIARACAVDSSSCKGVSNGSLSREYTNGADLSDVDFESSMTNSSEVETLAIQPRKLLILDARSYA
AAVANRAKGGGCECPEYYPNCEVVFMGMANIHSIRKSFQSLRFLCTQMPDPANWLSALESTKWLQHLSLLLKASLLVSNA
VDRDHRPVLVHCSDGWDRTPQIVALAKLLLDPYYRTIEGFQVLVETEWLDFGHKFADRCGHGENAEDLNERCPVFLQWLD
CVHQLQRQFPCSFEFNEAFLVKLVQHTYSCLFGTFLCNSGKEREDRRIQERTCSVWSLLRAANRSFGNMLYSSHSETVLH
PVCHVRNLMLWTAVYLPSSSPTTPSDDSCAPYPAPGANPEDQPLGRRPKTRSFDNLPSACEVGNPLTSNRRSSDPSLNEK
WQDHRRSLELNIGAGTDGAASPEPDERVNGQTMVGIMGTFSNGGTENGEGLKDVRLPMIDGVDEAELTVGVAVGQMENIL
QEATKDESAPDTRSVAKPNETSAVNGKTDDIAQEGRTEDDLESKSLEDGNVNGHCSEDGNSEASSALSQDTSESQDSEEI
EKCTTQEDDSSCNPTILTPNGLRTLTNGHGVEPSIEQAEKRLSLLESSTETLTEDLGVRPESLGPLSIPPPLKALAESSC

LKQGLRTAARTLNNTPKRASLSAFPPASTDLLHPMCNGDSPDPEPSTPRTNGERATLSRQVSLASCGSLTLHARGTCSHH
RCLHLGFLGRPSFSPPEPPSSSSRSHLDDDGLTLHTDAIQQRLRQIEAGHQLEVEALKRQVQELWSRLESHQAAGMLRLN
GDIGDEVTSITDSDFNLEPNCLSRCSTELFSEASWEQVDKQDTEVTRWYPDHLAAQCYGCERGFWLATRKHHCRNCGNVF
CGSCCDQKIPVPSQQLFEPSRVCRSCFSNLQPPAPPLDLELDKPITASSN
>NP_956162.2 hepatocyte growth factor-regulated tyrosine kinase substrate [Danio rerio]
MGKGGGTFERLLEKATSQLLLETDWESILQICDLIRQGDTQAKYAIGAIKKKLNDKNPHVALYALEVLESVVKNCGQTIH
DEVASKQTMEELKELFKKQPEPNVKNKILYLIQAWAHAFRNEPKYKVVQDTYQIMKVEGHVFPEFKESDAMFAAERAPDW
VDAEECHRCRVQFGVMTRKHHCRACGQIFCGKCSSKYSTIPKFGIEKEVRVCEPCFEILNKKAEGKAPSTGPSELPPEYL
TSPLSQQSQMPPKRDEAALQEEEELQLAIALSQSEAEEKERMRQKNPYSAYPKADPTPVTSSAPPVSTLYSSPVNSSAPS
AEEVDPELARYLNRTYWEKKQEEVRKSPTPSAPAPVSLAEPVPVSQPVESHVPVQPINIVEQYQNGESEENHEQFLKALQ
NSVTTFLNRMKSNHMRGRSITNDSAVLSLFQSINNMHPQLLEILNQLDEKRLYYEGLQDKLAQVRDARAALNALREEHRE
KLRRAAEEAERQRQIQLAQKLEIMRQKKQEYLEMQRQLAIQRLQEQEKERQMRLEQQKHTIQMRAQMPAFSLPYAQMQSL
PPNVAGGVVYPPAGPPSYPGTFSPSGSVEGSPMHGVYMNQAGQTAAGGPYQAMPPDPNMVNAYMYQTAGNSGQPAAPGQ
A
PPTTTPAYTNYQPTPTQGYQNVVSQAQSLPPMSQAAPTNGIAYMGYQQYNMQNMMSALPGQDPNMPPQQPYMPGQQPMY
Q
QMAPPGGPQQQSQQQQQPPPPPQAPPGSAEAQLISFD
>XP_021325300.1 hepatocyte growth factor-regulated tyrosine kinase substrate isoform X8 [Danio rerio]
MGKGGGTFERLLEKATSQLLLETDWESILQICDLIRQGDTQAKYAIGAIKKKLNDKNPHVALYALEVLESVVKNCGQTIH
DEVASKQTMEELKELFKKQPEPNVKNKILYLIQAWAHAFRNEPKYKVVQDTYQIMKVEGHVFPEFKESDAMFAAERAPDW
VDAEECHRCRVQFGVMTRKHHCRACGQIFCGKCSSKYSTIPKFGIEKEVRVCEPCFEILNKKAEGKAPSTGPSELPPEYL
TSPLSQQSQMPPKRDEAALQEEEELQLAIALSQSEAEEKERMRQKNPYSAYPKADPTPVTSSAPPVSTLYSSPVNSSAPS
AEEVDPELARYLNRTYWEKKQEEVRKSPTPSAPAPVSLAEPVPVSQPVESHVPVQPINIVEQYQNGESEENHEQFLKALQ
NSVTTFLNRMKSNHMRGRSITNDSAVLSLFQSINNMHPQLLEILNQLDEKRLYYEGLQDKLAQVRDARAALNALREEHRE
KLRRAAEEAERQRQIQLAQKLEIMRQKKQEYLEMQRQLAIQRLQEQEKERQMRLEQQKHTIQMRAQMPAFSLPYAQMQSL
PPNVAGGVVYPPAGPPSYPGTFSPSGSVEGSPMHGVYMNQAGQTAAGGPYQAMPPDPNMVNAYMYQTAGNSGQPAAPGQ
A
PPTTTPAYTNYQPTPTQGYQNVVSQAQSLPPMSQAAPTNGIAYMGYQQYNMQNMMSALPGQDPNMPPQQPYMPGQQPMY
Q
QVSNYMAPPGGPQQQSQQQQQPPPPPQAPPGSAEAQLISFD
>XP_021325297.1 hepatocyte growth factor-regulated tyrosine kinase substrate isoform X7 [Danio rerio]
MGKGGGTFERLLEKATSQLLLETDWESILQICDLIRQGDTQAKYAIGAIKKKLNDKNPHVALYALEVLESVVKNCGQTIH
DEVASKQTMEELKELFKKQPEPNVKNKILYLIQAWAHAFRNEPKYKVVQDTYQIMKVEGHVFPEFKESDAMFAAERAPDW

VDAEECHRCRVQFGVMTRKHHCRACGQIFCGKCSSKYSTIPKFGIEKEVRVCEPCFEILNKKAEGKAPSTGPSELPPEYL
TSPLSQQSQMPPKRDEAALQEEEELQLAIALSQSEAEEKERMRQKNPYSAYPKADPTPVTSSAPPVSTLYSSPVNSSAPS
AEEVDPELARYLNRTYWEKKQEEVRKSPTPSAPAPVSLAEPVPVSQPVESHVPVQPINIVEQQYQNGESEENHEQFLKAL
QNSVTTFLNRMKSNHMRGRSITNDSAVLSLFQSINNMHPQLLEILNQLDEKRLYYEGLQDKLAQVRDARAALNALREEHR
EKLRRAAEEAERQRQIQLAQKLEIMRQKKQEYLEMQRQLAIQRLQEQEKERQMRLEQQKHTIQMRAQMPAFSLPYAQMQS
LPPNVAGGVVYPPAGPPSYPGTFSPSGSVEGSPMHGVYMNQAGQTAAGGPYQAMPPDPNMVNAYMYQTAGNSGQPAAPG
Q
APPTTTPAYTNYQPTPTQGYQNVVSQAQSLPPMSQAAPTNGIAYMGYQQYNMQNMMSALPGQDPNMPPQQPYMPGQQPM
Y
QQVSNYMAPPGGPQQQSQQQQPPPPPQAPPGSAEAQLISFD
>XP_021325293.1 hepatocyte growth factor-regulated tyrosine kinase substrate isoform X6 [Danio rerio]
MGKGGGTFERLLEKATSQLLLETDWESILQICDLIRQGDTQAKYAIGAIKKKLNDKNPHVALYALEVLESVVKNCGQTIH
DEVASKQTMEELKELFKKQPEPNVKNKILYLIQAWAHAFRNEPKYKVVQDTYQIMKVEGHVFPEFKESDAMFAAERAPDW
VDAEECHRCRVQFGVMTRKHHCRACGQIFCGKCSSKYSTIPKFGIEKEVRVCEPCFEILNKKAEGKAPSTGPSELPPEYL
TSPLSQQSQVVPTESMPPKRDEAALQEEEELQLAIALSQSEAEEKERMRQKNPYSAYPKADPTPVTSSAPPVSTLYSSPV
NSSAPSAEEVDPELARYLNRTYWEKKQEEVRKSPTPSAPAPVSLAEPVPVSQPVESHVPVQPINIVEQYQNGESEENHEQ
FLKALQNSVTTFLNRMKSNHMRGRSITNDSAVLSLFQSINNMHPQLLEILNQLDEKRLYYEGLQDKLAQVRDARAALNAL
REEHREKLRRAAEEAERQRQIQLAQKLEIMRQKKQEYLEMQRQLAIQRLQEQEKERQMRLEQQKHTIQMRAQMPAFSLPY
AQMQSLPPNVAGGVVYPPAGPPSYPGTFSPSGSVEGSPMHGVYMNQAGQTAAGGPYQAMPPDPNMVNAYMYQTAGNSG
QP
AAPGQAPPTTTPAYTNYQPTPTQGYQNVVSQAQSLPPMSQAAPTNGIAYMGYQQYNMQNMMSALPGQDPNMPPQQPYMP
G
QQPMYQQVSNYMAPPGGPQQQSQQQQPPPPPQAPPGSAEAQLISFD
>XP_021325291.1 hepatocyte growth factor-regulated tyrosine kinase substrate isoform X5 [Danio rerio]
MGKGGGTFERLLEKATSQLLLETDWESILQICDLIRQGDTQAKYAIGAIKKKLNDKNPHVALYALEVLESVVKNCGQTIH
DEVASKQTMEELKELFKKQPEPNVKNKILYLIQAWAHAFRNEPKYKVVQDTYQIMKVEGHVFPEFKESDAMFAAERAPDW
VDAEECHRCRVQFGVMTRKHHCRACGQIFCGKCSSKYSTIPKFGIEKEVRVCEPCFEILNKKAEGKAPSTGPSELPPEYL
TSPLSQQSQVVPTESMPPKRDEAALQEEEELQLAIALSQSEAEEKERMRQKNPYSAYPKADPTPVTSSAPPVSTLYSSPV
NSSAPSAEEVDPELARYLNRTYWEKKQEEVRKSPTPSAPAPVSLAEPVPVSQPVESHVPVQPINIVEQQYQNGESEENHE
QFLKALQNSVTTFLNRMKSNHMRGRSITNDSAVLSLFQSINNMHPQLLEILNQLDEKRLYYEGLQDKLAQVRDARAALNA
LREEHREKLRRAAEEAERQRQIQLAQKLEIMRQKKQEYLEMQRQLAIQRLQEQEKERQMRLEQQKHTIQMRAQMPAFSLP
YAQMQSLPPNVAGGVVYPPAGPPSYPGTFSPSGSVEGSPMHGVYMNQAGQTAAGGPYQAMPPDPNMVNAYMYQTAGNS
GQ

PAAPGQAPPTTTPAYTNYQPTPTQGYQNVVSQAQSLPPMSQAAPTNGIAYMGYQQYNMQNMMSALPGQDPNMPPQQPYM
P
GQQPMYQQVSNYMAPPGGPQQQSQQQQQPPPPPQAPPGSAEAQLISFD
>XP_021332790.1 FERM, RhoGEF and pleckstrin domain-containing protein 2 isoform X2 [Danio rerio]
MGEIEGSYRSLQTPAGSRLGGQHNTGISTLEPGQSLSSAMGAGGKGHNKGLQIRVQGLDEAQEFYELESKADGQLLLSDV
FRRINLIESDYFGLEFQNLQMNWVWLDPSKLIVKQVRRPMNTLFRLSVKFFPPDPGQLQEEFTRYLFSLQIKRDLLDGRL
SCTENTAALLASHLVQSEIGDYDDLADREFLKMNKLLPCQEHVQEKIMELHRRHTGQTPAESDFQVLEIARKLEMFGVRF
HPAADREGTKINLAVAHMGLQVFQGHTKINTFNWSKIRKLSFKRKRFLIKLHPEVHGPHQDTLEFLMGSRDQCKIFWKNC
VEHHSFFRLLDQPQPKSKAIFFSRGSSFRYSGRTQKQLVEYVRDSGLRRTPYQRRNSKIRMSTRSLASDVPKQNLSFNDS
LRTHIPPSSITASFQSLHAPSVSMRAETPNQLLHLHQTQHQPLRAPSPMKQDPIKTNSQSTYAFPDSIAIDSPQLSPFND
KGPLCLSPSFQMSTLSLPGQAPSPLQSPILNEVGTARLDEDEEGRRKRYPTDKAYFIAKEILTTERTYLKDLEVITVWFR
SAVIKENAMPEGLMTLLFSNIDPIYEFHRGFLKEIDQRLALWEGRSNAHVKGDYQRIGDVMLRNMCSLKEFTGYLQKHDE
VLTELEKATKRVKKLETVYKEFELQKVCYLPLNTFLLKPIQRLMHYKLILERLCKHYSPQHRDYDDCKEALKEVAEIATQ
LQSSLIRLENFQKLTELQRDLIGIENLTAPGREFIREGCLYKLTKKGLQQRMFFLFSDMLLYTSKGVTATNQFKVHGQLP
LHGMIVEESENEWSVPHCFTIYSAQRTIVVAASSKVEMNKWIEDLNMAIEMSKKCQEKSDVLLDPSLCDRSNRSSDEVSL
EQESEDDMNSSRCSLDKQSHHRANTTMHVCWHRNTSVSMSDHSLAVENQLSGYLLRKFKNSNGWQKLWVVFTNFCLFFY
K
THQDDFPLASLPLLGYTVSTPGESDSIHKEYVFKLQFKSHVYFFRAESEYTFERWMEVIKSAASATGRMSLLVPKGPAEV
NVGN
>XP_002667032.4 myotubularin-related protein 4 isoform X1 [Danio rerio]
MSRSVSCSMLNCFGEEGPPSLEYIQARDLFPQKELLKEDDTLQVPFPVLQGEWVEFLGRADDAVIAISNYRLHIKFKDSI
INVPLRLIESVESRDMFQLHIICKDSKVVRCHFSTFKQCEEWLKRLQRAITHPARLEELFALAYHAWCLGANTDDEDQHL
HLCRPGDHVRHRLEVEVKRMGFDMQNAWRVSDINTNYKLCSSYPQKLLVPVWITDKELESVASFRSWKRIPVVVYRHQRN
GAVIARCSQPEISWWGWRNTEDEYLVTSIAKACQLDGGSRAGRSRGDGPDSDSDFDSSLTGCPAPDSSAAAQKLLILDAR
SYTAAVANRAKGGGCECEEYYPNCEVMFMGMANIHSIRNSFQSLRAVCSQIPDPGNWLSALESTRWLQHLSVMLKAASLV
CSALERDGRPVLVHCSDGWDRTPQIVALAKILLDPFYRTIEGFQVLVETEWLDFGHKFADRCGHQENGDDVSEQCPVFLQ
WLDCVHQLLKQFPCLFEFNEAFLVKLVQHTYSCLYGTFLCNNGKERETRNVYKRTCSIWSLLRTGNKNFQNFLYIPCHDM
VLQPVCHTRALQLWTAVYLPSSSPCTAAEDAVEVYLSPGAGDREEFSSRSLDRLPKTRSMDNLLSACENGVTLTRTSSDP
NLNKHCPEGRPSLEPNPPLILAPDAGALREELENHQSLPSEQLEDDQGVEPCLTTQPLPSIPRPLAIEQAPPPLISNQSP
PQITHSPLPPSDVHVRTAEITSTASLCNGFAHVENTRESSSDSVETLTEQSVCVEVKKPDEVRNGALVLLRERPHAVEYA
KTARRLISTSPLPDLSLLGSQWDSVQGMVQSACGGAIQHGSYHGRRLANKLLRAQAGSSVGGGPCCHRDPLRAPGSPIQS
GWFSAVKSSGYAALCSSSLGPSLRQLLPSHSCPSAGSSAYLDDDGLPVALDAVQQRLRQIEASYKQEVEVLRRQVRQLQL
KLESKQFCTPPSEPDVDYEDDITCLRESDGSEDSDSLSSRSEDRFSEGSWDRVERKDTEVTRWVPDHMASHCFNCDCEFW

IAKRRHHCRNCGNVFCKDCCHLKLPIPDQQLYDPVLVCNTCYDLLLESRTRELRSQQLKKAIATASS
>XP_021332402.1 myotubularin-related protein 4 isoform X3 [Danio rerio]
MFPGVDSEMSVPLRLIESVESRDMFQLHIICKDSKVVRCHFSTFKQCEEWLKRLQRAITHPARLEELFALAYHAWCLGAN
TDDEDQHLHLCRPGDHVRHRLEVEVKRMGFDMQNAWRVSDINTNYKLCSSYPQKLLVPVWITDKELESVASFRSWKRIPV
VVYRHQRNGAVIARCSQPEISWWGWRNTEDEYLVTSIAKACQLDGGSRAGRSRGDGPDSDSDFDSSLTGCPAPDSSAAAQ
KLLILDARSYTAAVANRAKGGGCECEEYYPNCEVMFMGMANIHSIRNSFQSLRAVCSQIPDPGNWLSALESTRWLQHLSV
MLKAASLVCSALERDGRPVLVHCSDGWDRTPQIVALAKILLDPFYRTIEGFQVLVETEWLDFGHKFADRCGHQENGDDVS
EQCPVFLQWLDCVHQLLKQFPCLFEFNEAFLVKLVQHTYSCLYGTFLCNNGKERETRNVYKRTCSIWSLLRTGNKNFQNF
LYIPCHDMVLQPVCHTRALQLWTAVYLPSSSPCTAAEDAVEVYLSPGAGDREEFSSRSLDRLPKTRSMDNLLSACENGVT
LTRTSSDPNLNKHCPEGRPSLEPNPPLILAPDAGALREELENHQSLPSEQLEDDQGVEPCLTTQPLPSIPRPLAIEQAPP
PLISNQSPPQITHSPLPPSDVHVRTAEITSTASLCNGFAHVENTRESSSDSVETLTEQSVCVEVKKPDEVRNGALVLLRE
RPHAVEYAKTARRLISTSPLPDLSLLGSQWDSVQGMVQSACGGAIQHGSYHGRRLANKLLRAQAGSSVGGGPCCHRDPLR
APGSPIQSGWFSAVKSSGYAALCSSSLGPSLRQLLPSHSCPSAGSSAYLDDDGLPVALDAVQQRLRQIEASYKQEVEVLR
RQVRQLQLKLESKQFCTPPSEPDVDYEDDITCLRESDGSEDSDSLSSRSEDRFSEGSWDRVERKDTEVTRWVPDHMASHC
FNCDCEFWIAKRRHHCRNCGNVFCKDCCHLKLPIPDQQLYDPVLVCNTCYDLLLESRTRELRSQQLKKAIATASS
>XP_009299405.1 myotubularin-related protein 4 isoform X2 [Danio rerio]
MGEEGPPSLEYIQARDLFPQKELLKEDDTLQVPFPVLQGEWVEFLGRADDAVIAISNYRLHIKFKDSIINVPLRLIESVE
SRDMFQLHIICKDSKVVRCHFSTFKQCEEWLKRLQRAITHPARLEELFALAYHAWCLGANTDDEDQHLHLCRPGDHVRHR
LEVEVKRMGFDMQNAWRVSDINTNYKLCSSYPQKLLVPVWITDKELESVASFRSWKRIPVVVYRHQRNGAVIARCSQPEI
SWWGWRNTEDEYLVTSIAKACQLDGGSRAGRSRGDGPDSDSDFDSSLTGCPAPDSSAAAQKLLILDARSYTAAVANRAKG
GGCECEEYYPNCEVMFMGMANIHSIRNSFQSLRAVCSQIPDPGNWLSALESTRWLQHLSVMLKAASLVCSALERDGRPVL
VHCSDGWDRTPQIVALAKILLDPFYRTIEGFQVLVETEWLDFGHKFADRCGHQENGDDVSEQCPVFLQWLDCVHQLLKQF
PCLFEFNEAFLVKLVQHTYSCLYGTFLCNNGKERETRNVYKRTCSIWSLLRTGNKNFQNFLYIPCHDMVLQPVCHTRALQ
LWTAVYLPSSSPCTAAEDAVEVYLSPGAGDREEFSSRSLDRLPKTRSMDNLLSACENGVTLTRTSSDPNLNKHCPEGRPS
LEPNPPLILAPDAGALREELENHQSLPSEQLEDDQGVEPCLTTQPLPSIPRPLAIEQAPPPLISNQSPPQITHSPLPPSD
VHVRTAEITSTASLCNGFAHVENTRESSSDSVETLTEQSVCVEVKKPDEVRNGALVLLRERPHAVEYAKTARRLISTSPL
PDLSLLGSQWDSVQGMVQSACGGAIQHGSYHGRRLANKLLRAQAGSSVGGGPCCHRDPLRAPGSPIQSGWFSAVKSSGYA
ALCSSSLGPSLRQLLPSHSCPSAGSSAYLDDDGLPVALDAVQQRLRQIEASYKQEVEVLRRQVRQLQLKLESKQFCTPPS
EPDVDYEDDITCLRESDGSEDSDSLSSRSEDRFSEGSWDRVERKDTEVTRWVPDHMASHCFNCDCEFWIAKRRHHCRNCG
NVFCKDCCHLKLPIPDQQLYDPVLVCNTCYDLLLESRTRELRSQQLKKAIATASS
>XP_005155593.1 zinc finger FYVE domain-containing protein 16 isoform X3 [Danio rerio]
MDSYFKAAVCDLDKLLDDFELSAEELESKPGTYVSPPCPDSTLIPGRFIPEQHTVPQTLPDVNALHYGLTSQSIDSNQKD
SYSNEWPLTGVDLLSSVDSRGIKSSAPPCPDRSLKPVCDLVNDTGSANLQQADSQEDFKELDIPDVQSTEGLLVDFESPE

DLSKPSHSEILKRDSEINCNSLSLLDVILPVVGEPYTSSSITLLETESSCPEASNSNGQNDRPWSQMDEDDVCNTCPSTT
EENISAFSEEHAVSLQPDSTSEDVRNPEDLTGSPTSEKESSLSCLPMAVSMCGSLVATLDPQKAAVVETKDEAELETKSA
IQEAEELTLPLEEPVPPFSSSRPDSSPEDEPEIVQIMSNPAAAGHYPERNFKRTLSPTDKLTCRDLGASNPSPSYSESPN
YSYEFGIANDYLPESDQTVMMVTDEELDAFLMGQGVKINPDAAVEKFSDDGFSEFNGNAERDGFLDEELQSCKVETFASP
ESDGSQYLEESEGSNVSSSSQDSSPLRADSTQTITHETVTSPVEIQSVCSPNQESTYGGARPKQLSNQTVRPPSERHRIS
HAEADVGGSANGSQVSPNQDEINCEPPSYQQYDSSYGCDELSEPPPYPGEMLEDGESSVDLSGHGAEGLGSKQPPWVPDS
EAPNCMNCMQKFTFTKRRHHCRACGKVYCASCCNRKCRLKYLDKEARVCVICHVTIQRGFPREQKHVWFADGILPNGEVA
DTTKLSVQTRRSSQESSPVTPDPPAPDDKFPDEREESVGGRSDSPVEASRPPVRGPWDYGLLCGVGNCVQRDASLVPEDD
DGLPPLLIITGEEEEGDLLVEERPAACQILLLLEEGGPQPLTFVLNANLLVNVKIVTYCGKRCWCLSSNGLQGVGQKELV
FIIECLPEENSLPRDVFNLYVSIYQDAQKGKFIEHLGNVTFTDSFLGSKDHGGVLFFTATFQPLEGLALPQVSFLCGVLI
QKLEVPWAKVFPLRLLLALGAKYSVYPCPLMSIRFRESVFKETGHTIMNLLTDLRNYQYSLPVVESLRVHMEMGNSYIEI
PKSRFNEMLKVVNSSNEHVISFGACFSTEADSHLVCIQNEDGSYQTSANSIPGKTRRVTGASFVVFNGALKASSGFIAKS
SIVEDGLMVQIPPETMEGLRQALRDQNDFQIPCGKTDSEETRENVNVRWVDWTAPVNAGVTSPVDGKSLEGVSSVKIQQD
SEFEMDGRTIRCTEVFYLLKSSDVSLPAILTSSAQFQREIATASCAALCPHLSVLMANGFNSLALRVSTDSDMVEYQAGS
SGRLLPQKYMNDLDGALIPVIHGGSSCVPHQAMDMEFFFYISLSI
>XP_021325289.1 hepatocyte growth factor-regulated tyrosine kinase substrate isoform X4 [Danio rerio]
MGKGGGTFERLLEKATSQLLLETDWESILQICDLIRQGDTQAKYAIGAIKKKLNDKNPHVALYALEVLESVVKNCGQTIH
DEVASKQTMEELKELFKKQPEPNVKNKILYLIQAWAHAFRNEPKYKVVQDTYQIMKVEGHVFPEFKESDAMFAAERAPDW
VDAEECHRCRVQFGVMTRKHHCRACGQIFCGKCSSKYSTIPKFGIEKEVRVCEPCFEILNNHPSLSPPPRKAEGKAPSTG
PSELPPEYLTSPLSQQSQMPPKRDEAALQEEEELQLAIALSQSEAEEKERMRQKNPYSAYPKADPTPVTSSAPPVSTLYS
SPVNSSAPSAEEVDPELARYLNRTYWEKKQEEVRKSPTPSAPAPVSLAEPVPVSQPVESHVPVQPINIVEQQYQNGESEE
NHEQFLKALQNSVTTFLNRMKSNHMRGRSITNDSAVLSLFQSINNMHPQLLEILNQLDEKRLYYEGLQDKLAQVRDARAA
LNALREEHREKLRRAAEEAERQRQIQLAQKLEIMRQKKQEYLEMQRQLAIQRLQEQEKERQMRLEQQKHTIQMRAQMPAF
SLPYAQMQSLPPNVAGGVVYPPAGPPSYPGTFSPSGSVEGSPMHGVYMNQAGQTAAGGPYQAMPPDPNMVNAYMYQTAG
N
SGQPAAPGQAPPTTTPAYTNYQPTPTQGYQNVVSQAQSLPPMSQAAPTNGIAYMGYQQYNMQNMMSALPGQDPNMPPQQ
P
YMPGQQPMYQQVSNYMAPPGGPQQQSQQQQQPPPPPQAPPGSAEAQLISFD
>XP_005164430.1 hepatocyte growth factor-regulated tyrosine kinase substrate isoform X3 [Danio rerio]
MGKGGGTFERLLEKATSQLLLETDWESILQICDLIRQGDTQAKYAIGAIKKKLNDKNPHVALYALEVLESVVKNCGQTIH
DEVASKQTMEELKELFKKQPEPNVKNKILYLIQAWAHAFRNEPKYKVVQDTYQIMKVEGHVFPEFKESDAMFAAERAPDW
VDAEECHRCRVQFGVMTRKHHCRACGQIFCGKCSSKYSTIPKFGIEKEVRVCEPCFEILNNHPSLSPPPRKAEGKAPSTG
PSELPPEYLTSPLSQQSQVVPTESMPPKRDEAALQEEEELQLAIALSQSEAEEKERMRQKNPYSAYPKADPTPVTSSAPP

VSTLYSSPVNSSAPSAEEVDPELARYLNRTYWEKKQEEVRKSPTPSAPAPVSLAEPVPVSQPVESHVPVQPINIVEQQYQ
NGESEENHEQFLKALQNSVTTFLNRMKSNHMRGRSITNDSAVLSLFQSINNMHPQLLEILNQLDEKRLYYEGLQDKLAQV
RDARAALNALREEHREKLRRAAEEAERQRQIQLAQKLEIMRQKKQEYLEMQRQLAIQRLQEQEKERQMRLEQQKHTIQMR
AQMPAFSLPYAQMQSLPPNVAGGVVYPPAGPPSYPGTFSPSGSVEGSPMHGVYMNQAGQTAAGGPYQAMPPDPNMVNAY
M
YQTAGNSGQPAAPGQAPPTTTPAYTNYQPTPTQGYQNVVSQAQSLPPMSQAAPTNGIAYMGYQQYNMQNMMSALPGQDP
N
MPPQQPYMPGQQPMYQQMAPPGGPQQQSQQQQQPPPPPQAPPGSAEAQLISFD
>XP_021325286.1 hepatocyte growth factor-regulated tyrosine kinase substrate isoform X2 [Danio rerio]
MGKGGGTFERLLEKATSQLLLETDWESILQICDLIRQGDTQAKYAIGAIKKKLNDKNPHVALYALEVLESVVKNCGQTIH
DEVASKQTMEELKELFKKQPEPNVKNKILYLIQAWAHAFRNEPKYKVVQDTYQIMKVEGHVFPEFKESDAMFAAERAPDW
VDAEECHRCRVQFGVMTRKHHCRACGQIFCGKCSSKYSTIPKFGIEKEVRVCEPCFEILNNHPSLSPPPRKAEGKAPSTG
PSELPPEYLTSPLSQQSQVVPTESMPPKRDEAALQEEEELQLAIALSQSEAEEKERMRQKNPYSAYPKADPTPVTSSAPP
VSTLYSSPVNSSAPSAEEVDPELARYLNRTYWEKKQEEVRKSPTPSAPAPVSLAEPVPVSQPVESHVPVQPINIVEQYQN
GESEENHEQFLKALQNSVTTFLNRMKSNHMRGRSITNDSAVLSLFQSINNMHPQLLEILNQLDEKRLYYEGLQDKLAQVR
DARAALNALREEHREKLRRAAEEAERQRQIQLAQKLEIMRQKKQEYLEMQRQLAIQRLQEQEKERQMRLEQQKHTIQMRA
QMPAFSLPYAQMQSLPPNVAGGVVYPPAGPPSYPGTFSPSGSVEGSPMHGVYMNQAGQTAAGGPYQAMPPDPNMVNAYM
Y
QTAGNSGQPAAPGQAPPTTTPAYTNYQPTPTQGYQNVVSQAQSLPPMSQAAPTNGIAYMGYQQYNMQNMMSALPGQDPN
M
PPQQPYMPGQQPMYQQVSNYMAPPGGPQQQSQQQQQPPPPPQAPPGSAEAQLISFD
>XP_021325285.1 hepatocyte growth factor-regulated tyrosine kinase substrate isoform X1 [Danio rerio]
MGKGGGTFERLLEKATSQLLLETDWESILQICDLIRQGDTQAKYAIGAIKKKLNDKNPHVALYALEVLESVVKNCGQTIH
DEVASKQTMEELKELFKKQPEPNVKNKILYLIQAWAHAFRNEPKYKVVQDTYQIMKVEGHVFPEFKESDAMFAAERAPDW
VDAEECHRCRVQFGVMTRKHHCRACGQIFCGKCSSKYSTIPKFGIEKEVRVCEPCFEILNNHPSLSPPPRKAEGKAPSTG
PSELPPEYLTSPLSQQSQVVPTESMPPKRDEAALQEEEELQLAIALSQSEAEEKERMRQKNPYSAYPKADPTPVTSSAPP
VSTLYSSPVNSSAPSAEEVDPELARYLNRTYWEKKQEEVRKSPTPSAPAPVSLAEPVPVSQPVESHVPVQPINIVEQQYQ
NGESEENHEQFLKALQNSVTTFLNRMKSNHMRGRSITNDSAVLSLFQSINNMHPQLLEILNQLDEKRLYYEGLQDKLAQV
RDARAALNALREEHREKLRRAAEEAERQRQIQLAQKLEIMRQKKQEYLEMQRQLAIQRLQEQEKERQMRLEQQKHTIQMR
AQMPAFSLPYAQMQSLPPNVAGGVVYPPAGPPSYPGTFSPSGSVEGSPMHGVYMNQAGQTAAGGPYQAMPPDPNMVNAY
M
YQTAGNSGQPAAPGQAPPTTTPAYTNYQPTPTQGYQNVVSQAQSLPPMSQAAPTNGIAYMGYQQYNMQNMMSALPGQDP
N

MPPQQPYMPGQQPMYQQVSNYMAPPGGPQQQSQQQQQPPPPPQAPPGSAEAQLISFD
>XP_005155592.1 zinc finger FYVE domain-containing protein 16 isoform X1 [Danio rerio]
MDSYFKAAVCDLDKLLDDFELSAEELESKPGTYVSPPCPDSTLIPGRFIPEQHTVPQTLPDVNALHYGLTSQSIDSNQKD
SYSNEWPLTGVDLLSSVDSRGIKSSAPPCPDRSLKPVCDLVNDTGSANLQQADSQEDFKELDIPDVQSTEGLLVDFESPE
DLSKPSHSEILKRDSEINCNSLSLLDVILPVVGEPYTSSSITLLETESSCPEASNSNGQNDRPWSQMDEDDVCNTCPSTT
EENISAFSEEHAVSLQPDSTSEDVRNPEDLTGSPTSEKESSLSCLPMAVSMCGSLVATLDPQKAAVVETKDEAELETKSA
IQEAEELTLPLEEPVPPFSSSRPDSSPEDEPEIVQIMSNPAAAGHYPERNFKRTLSPTDKLTCRDLGASNPSPSYSESPN
YSYEFGIANDYLPESDQTVMMVTDEELDAFLMGQGVKINPDAAVEKFSDDGFSEFNGNAERDGFLDEELQSCKVETFASP
ESDGSQYLEESEGSNVSSSSQDSSPLRADSTQTITHETVTSPVEIQSVCSPNQESTYGGARPKQLSNQTVRPPSERHRIS
HAEADVGGSANGSQVSPNQDEINCEPPSYQQYDSSYGCDELSEPPPYPGEMLEDGESSVDLSGHGAEGLGSKQPPWVPDS
EAPNCMNCMQKFTFTKRRHHCRACGKVYCASCCNRKCRLKYLDKEARVCVICHVTIQRAQAHERMMSPTGPSPNPNIPSE
YCSTIPPMQQARAAGTLNSPPPTVMVPVSVLKHPGNDGFPREQKHVWFADGILPNGEVADTTKLSVQTRRSSQESSPVTP
DPPAPDDKFPDEREESVGGRSDSPVEASRPPVRGPWDYGLLCGVGNCVQRDASLVPEDDDGLPPLLIITGEEEEGDLLVE
ERPAACQILLLLEEGGPQPLTFVLNANLLVNVKIVTYCGKRCWCLSSNGLQGVGQKELVFIIECLPEENSLPRDVFNLYV
SIYQDAQKGKFIEHLGNVTFTDSFLGSKDHGGVLFFTATFQPLEGLALPQVSFLCGVLIQKLEVPWAKVFPLRLLLALGA
KYSVYPCPLMSIRFRESVFKETGHTIMNLLTDLRNYQYSLPVVESLRVHMEMGNSYIEIPKSRFNEMLKVVNSSNEHVIS
FGACFSTEADSHLVCIQNEDGSYQTSANSIPGKTRRVTGASFVVFNGALKASSGFIAKSSIVEDGLMVQIPPETMEGLRQ
ALRDQNDFQIPCGKTDSEETRENVNVRWVDWTAPVNAGVTSPVDGKSLEGVSSVKIQQDSEFEMDGRTIRCTEVFYLLKS
SDVSLPAILTSSAQFQREIATASCAALCPHLSVLMANGFNSLALRVSTDSDMVEYQAGSSGRLLPQKYMNDLDGALIPVI
HGGSSCVPHQAMDMEFFFYISLSI
>XP_001920918.3 zinc finger FYVE domain-containing protein 16 isoform X2 [Danio rerio]
MDSYFKAAVCDLDKLLDDFELSAEELESKPGTYVSPPCPDSTLIPGRFIPEQHTVPQTLPDVNALHYGLTSQSIDSNQKD
SYSNEWPLTGVDLLSSVDSRGIKSSAPPCPDRSLKPVCDLVNDTGSANLQQADSQEDFKELDIPDVQSTEGLLVDFESPE
DLSKPSHSEILKRDSEINCNSLSLLDVILPVVGEPYTSSSITLLETESSCPEASNSNGQNDRPWSQMDEDDVCNTCPSTT
EENISAFSEEHAVSLQPDSTSEDVRNPEDLTGSPTSEKESSLSCLPMAVSMCGSLVATLDPQKAAVVETKDEAELETKSA
IQEAEELTLPLEEPVPPFSSSRPDSSPEDEPEIVQIMSNPAAAGHYPERNFKRTLSPTDKLTCRDLGASNPSPSYSESPN
YSYEFGIANDYLPESDQTVMMVTDEELDAFLMGQGVKINPDAAVEKFSDDGFSEFNGNAERDGFLDEELQSCKVETFASP
ESDGSQYLEESEGSNVSSSSQDSSPLRADSTQTITHETVTSPVEIQSVCSPNQESTYGGARPKQLSNQTVRPPSERHRIS
HAEADVGGSANGSQVSPNQDEINCEPPSYQQYDSSYGCDELSEPPPYPGEMLEDGESSVDLSGHGAEGLGSKQPPWVPDS
EAPNCMNCMQKFTFTKRRHHCRACGKVYCASCCNRKCRLKYLDKEARVCVICHVTIQRAQAHERMMSPTGPSPNPNIPSE
YCSTIPPMQQARAAGTLNSPPPTVMVPVSVLKHPGFPREQKHVWFADGILPNGEVADTTKLSVQTRRSSQESSPVTPDPP
APDDKFPDEREESVGGRSDSPVEASRPPVRGPWDYGLLCGVGNCVQRDASLVPEDDDGLPPLLIITGEEEEGDLLVEERP
AACQILLLLEEGGPQPLTFVLNANLLVNVKIVTYCGKRCWCLSSNGLQGVGQKELVFIIECLPEENSLPRDVFNLYVSIY

QDAQKGKFIEHLGNVTFTDSFLGSKDHGGVLFFTATFQPLEGLALPQVSFLCGVLIQKLEVPWAKVFPLRLLLALGAKYS
VYPCPLMSIRFRESVFKETGHTIMNLLTDLRNYQYSLPVVESLRVHMEMGNSYIEIPKSRFNEMLKVVNSSNEHVISFGA
CFSTEADSHLVCIQNEDGSYQTSANSIPGKTRRVTGASFVVFNGALKASSGFIAKSSIVEDGLMVQIPPETMEGLRQALR
DQNDFQIPCGKTDSEETRENVNVRWVDWTAPVNAGVTSPVDGKSLEGVSSVKIQQDSEFEMDGRTIRCTEVFYLLKSSDV
SLPAILTSSAQFQREIATASCAALCPHLSVLMANGFNSLALRVSTDSDMVEYQAGSSGRLLPQKYMNDLDGALIPVIHGG
SSCVPHQAMDMEFFFYISLSI
>XP_005165797.1 alsin isoform X3 [Danio rerio]
MEKLISSGEGDSSGERGLLHTWKGYSYSTAPERVLLSRPVLQAALGARHGVLLVEGGQVYSFGELPWKQNQTSSAEPILE
AVLSEQRVVFVAAGSAHSGVVTEDGGVHMWGENVHGQCGLLGLSVIPNPTPVGVLDSEATPPQTVKILEVACGDQHTLAL
SAKHEVWAWGSGCQLGLNTSTFPVWKPQKVDHLSGRHVLQVACGSAHSVALVRCLPPPQEPRKPPQDKCGQCHQLLYTM
T
DKEDHVIISDGHHCPLGVEMDSTEIKSAVEGSPDPGKPGKSSPTEPVPCMSTQISKSPTSSNISESTRPPESTLPFELQN
QVPAYCQESIEAPVEDSAAHEAPAAAGEVCVEASENSIPEPDSCRSSGKSPYPDEQAVKAYLKRLSDHALAEHTIKTSST
IHSAQLSGDPANLFASEPLIPVAQSVTPMGSALNNLVVSCASAVGERVVSTYEALSLRKVIGYWVPGEGRERVEERLRLE
ESMQGKKSSSLGDIREEEAELSRRLSLPGLLSQDNAESEVKASSSNTTTEVSPRLLRRTSRPRMRAVPLASGGIPETDAH
LPSLQTEVWSWGQGQDGQLGHGDLLPRLQPDCVKSLNGKEVLKVAAGAHHSLALTAQSQVFSWGCNTSGQLGHMESPTT
F
PQLTKLSDGIRVWDIGAGLQHTLLLADGDCIQPILYYSGQQVKEVPEPTEEEEQTAEYTQQPVLLPFCMNLGYVSSVYAG
GRTCAALTDCNVMGFIASLHELAAAERKYYCKLSNIKSLLLQPLLKLDSLSSALGQSSAGLLQNLIGRMSRLSQLTGQNS
ASLTSFLQRSRDIRGLVILEDAHLFLDTYSEYSSAVGDLLVMGGFQALVKPCHDVFGKGAEVVQRLSECSEEGLTLADVL
AHLFYLPIKHLHEYGRLLLKLATCYEVSSVDYQKLQDACSKFESMALHLKRRRKEAEYTLHFWKSFPGKMTDSLRKPSRR
LICESSNKALTLQNAGRFSVNWFILFNDALVHAQFSTHHIFPLATLWVEPISEESTGVYGLKVTSPEESFALLASSPAEK
AKWLRSINQAVEQALSGLGHDGIPPSTGMTQRADPPISRTASYTFYKDSRLKDAKYDGRWVSGKPHGRGVVKWPDGRMYT
GTFKNGFEDGFGDYVVSNKTLNSCDHYQGQWKDGKMHGFGTFRYASGEVYEGSFQDNMRHGHGMLRSGKLNSTSPSVFI
G
QWQYDKKSGYGVFDDITRGEKYMGMWLDDQRQGNGVVVTQFGLYYEGAFSNNKMMGTGVLLSEDDTTFEGEFLEDWTL
NG
KGVLTMPNGDYIEGSFCGVWGTGLKMSGSYYKPSLYDSDKEKGHALKLGRLAVHSEEKWKAVFLECWNRLGCDSPVQGQ
T
TTAWDNIAIALTANLRQQRDSPELLSRSHHKTLESLECIPQHVGPVTLEVYHTIRRYLVKACDTPLHPLGRLVETLVSVY
RMTYVGVGANRRLLPQAVNEITSYLSRIFQLVRFLFPELPEEGGSLADPPKEKVGTDADGKLLESPKPGCVVSSSALLLP
VLLPRLYPPLFTLYALEKEKEDDVYWECVLRLNKQPDLALLAFLGVQQKFWPVTVTIHGEKQQVLSSTKDACFASAVETL
QQISTTFTPSDKLQVIQLTFEEITQEVLALLKQDFLWSMDDLFPVFLFVVLRARIRNLGSEVSLIEDLMDPCVQHGEHGI

MFTTLKACYYQIQHEKVT
>NP_001096142.1 rabankyrin-5 [Danio rerio]
MRTEEVQKLQKHLALLRQEYVKMQQKLVETERRCSVLAAQASVPGSSSQASDSFISRLLAIVSDLYQQEQYSDLKVKIGD
QRLSAHKFVLAARSEIWSLANLASTSELDLSDAKPEVAMAMLRWTYTDDLELNGNDGFLIDLMKLANRFQLHLLRERCEK
GVMSSVNVRNCIRFYQTAEELDAATLMNYCGEIIASHWDDLRKEDFSTMDAQLLYKMIKSKTEYPLHKAIKVEREDVVFL
YLIEMDAQLPGKLNELDNNGDLALDLALSKKLESIATTLVNNKADVDMVDQSGWSLLHKAIQRGDEFASTFLIRHSAQVN
AATVGAVETPLHLVCSFSPKKHSGEVMAGMARIAEALLKAGANPNMQNSKGRTPLHEAVVSGNEPVFNQLLQCKQLDLEL
KDHEGSTALWLALQYITVASDPSVNPFEDEAPVVNGTSFDENSFAARLIQRGSNPDAPDTNGNCLMQRAAVAGSEAAAIF
LATHGAKVNHTNKWGETPLHTACRCGLAGLTVELLQQGANPNLQTDIALPGGVEKSQGVSQQSPLHLAIVHNHPDVVSVI
LEQKANALHATNNLQIIPDFSLKDSMDQTVLGLALWTGMHTIAAQLLGSGAAINDTMSDGQTLLHMAIKRQDSKSALFLL
EHQADINVRTQEGQTALQLAISNQLPLVVDAICTRGADMSVVNEKGDPPLWLALENGLEDIASTLVRHGCDATCWSSGPG
GCQQTLLHRAIDENNEVTACFLIRSGCDVNSSRRPGPNGEGDEEARDGQCPLHLAASWGLEEVAQCLLEFGANVNAQDSE
GRAPIHVAISNQQSVIIQLLISHPEIRLNIRDRQGMTPFACAMTHKNNKAAEAILKREPGAAEQVDNKGRNFLHVAVQNS
DIESVLFLISVQANVNSRVQDSAKLSPLHLAVQAGSEIIVRNLLLAGAKVNELTKHRQTALHLAAQQDLSTICSVLIENG
VDFAAVDENGNNALHLAVMQGRLNNVRALLTESNVDAEAYNLSQSPMHVLGQYGKENAAAIFELFLECMPEYPLDKPDNE
GNTVLLLAYMKGNANLCRAIVRAGARLGVTNNQGINIFNYQVATKQLLFRLLDMLSKEPPWCDGSNCYECITKFGVTTRK
HHCRHCGRLLCHKCSIKEIPIIKFDLNKPVRVCDICFDVLTLGGVS
>XP_002662342.2 alsin isoform X5 [Danio rerio]
MEKLISSGEGDSSGERGLLHTWKGYSYSTAPERVLLSRPVLQAALGARHGVLLVEGGQVYSFGELPWKQNQTSSAEPILE
AVLSEQRVVFVAAGSAHSGVVTEDGGVHMWGENVHGQCGLLGLSVIPNPTPVGVLDSEATPPQTVKILEVACGDQHTLAL
SAKHEVWAWGSGCQLGLNTSTFPVWKPQKVDHLSGRHVLQVACGSAHSVALVRCLPPPQEPRKPPQDKCGQCHQLLYTM
T
DKEDHVIISDGHHCPLGVEMDSTEIKSAVEGSPDPGKPGKSSPTEPVPCMSTQISKSPTSSNISESTRPPESTLPFELQN
QVPAYCQESIEAPVEDSAAHEAPAAAGEVCVEASENSIPEPDSCRSSGKSPYPDEQAVKAYLKRLSDHALAEHTIKTSST
IHSAQLSGDPANLFASEPLIPVAQSVTPMGSALNNLVVSCASAVGERVVSTYEALSLRKVIGYWVPGEGRERVEERLRLE
ESMQGKKSSSLGDIREEEAELSRRLSLPGLLSQVSPRLLRRTSRPRMRAVPLASGGIPETDAHLPSLQTEVWSWGQGQDG
QLGHGDLLPRLQPDCVKSLNGKEVLKVAAGAHHSLALTAQSQVFSWGCNTSGQLGHMESPTTFPQLTKLSDGIRVWDIGA
GLQHTLLLADGDCIQPILYYSGQQVKEVPEPTEEEEQTAEYTQQPVLLPFCMNLGYVSSVYAGGRTCAALTDCNVMGFIA
SLHELAAAERKYYCKLSNIKSLLLQPLLKLDSLSSALGQSSAGLLQNLIGRMSRLSQLTGQNSASLTSFLQRSRDIRGLV
ILEDAHLFLDTYSEYSSAVGDLLVMGGFQALVKPCHDVFGKGAEVVQRLSECSEEGLTLADVLAHLFYLPIKHLHEYGRL
LLKLATCYEVSSVDYQKLQDACSKFESMALHLKRRRKEAEYTLHFWKSFPGKMTDSLRKPSRRLICESSNKALTLQNAGR
FSVNWFILFNDALVHAQFSTHHIFPLATLWVEPISEESTGVYGLKVTSPEESFALLASSPAEKAKWLRSINQAVEQALSG
LGHDGIPPSTGMTQRADPPISRTASYTFYKDSRLKDAKYDGRWVSGKPHGRGVVKWPDGRMYTGTFKNGFEDGFGDYVVS

NKTLNSCDHYQGQWKDGKMHGFGTFRYASGEVYEGSFQDNMRHGHGMLRSGKLNSTSPSVFIGQWQYDKKSGYGVFDDI
T
RGEKYMGMWLDDQRQGNGVVVTQFGLYYEGAFSNNKMMGTGVLLSEDDTTFEGEFLEDWTLNGKGVLTMPNGDYIEGS
FC
GVWGTGLKMSGSYYKPSLYDSDKEKGHALKLGRLAVHSEEKWKAVFLECWNRLGCDSPVQGQTTTAWDNIAIALTANLR
Q
QRDSPELLSRSHHKTLESLECIPQHVGPVTLEVYHTIRRYLVKACDTPLHPLGRLVETLVSVYRMTYVGVGANRRLLPQA
VNEITSYLSRIFQLVRFLFPELPEEGGSLADPPKEKVGTDADGKLLESPKPGCVVSSSALLLPVLLPRLYPPLFTLYALE
KEKEDDVYWECVLRLNKQPDLALLAFLGVQQKFWPVTVTIHGEKQQVLSSTKDACFASAVETLQQISTTFTPSDKLQVIQ
LTFEEITQEVLALLKQDFLWSMDDLFPVFLFVVLRARIRNLGSEVSLIEDLMDPCVQHGEHGIMFTTLKACYYQIQHEKV
T
>XP_005171962.1 rabankyrin-5 isoform X1 [Danio rerio]
MAEEEVQKLQKHLALLRQEYVKMQQKLVETERRCSVLAAQASVPGSSSQASDSFISRLLAIVSDLYQQEQYSDLKVKIGD
QRLSAHKFVLAARSEIWSLANLASTSELDLSDAKPEVAMAMLRWTYTDDLELNGNDGFLIDLMKLANRFQLHLLRERCEK
GVMSSVNVRNCIRFYQTAEELDAATLMNYCGEIIASHWDDLRKEDFSTMDAQLLYKMIKSKTEYPLHKAIKVEREDVVFL
YLIEMDAQLPGKLNELDNNGDLALDLALSKKLESIATTLVNNKADVDMVDQSGWSLLHKAIQRGDEFASTFLIRHSAQVN
AATVGAVETPLHLVCSFSPKKHSGEVMAGMARIAEALLKAGANPNMQNSKGRTPLHEAVVSGNEPVFNQLLQCKQLDLEL
KDHEGSTALWLALQYITVASDPSVNPFEDEAPVVNGTSFDENSFAARLIQRGSNPDAPDTNGNCLMQRAAVAGSEAAAIF
LATHGAKVNHTNKWGETPLHTACRCGLAGLTVELLQQGANPNLQTDIALPGGVEKSQGVSQQSPLHLAIVHNHPDVVSVI
LEQKANALHATNNLQIIPDFSLKDSMDQTVLGLALWTGMHTIAAQLLGSGAAINDTMSDGQTLLHMAIKRQDSKSALFLL
EHQADINVRTQEGQTALQLAISNQLPLVVDAICTRGADMSVVNEKGDPPLWLALENGLEDIASTLVRHGCDATCWSSGPG
GCQQTLLHRAIDENNEVTACFLIRSGCDVNSSRRPGPNGEGDEEARDGQCPLHLAASWGLEEVAQCLLEFGANVNAQDSE
GRAPIHVAISNQQSVIIQLLISHPEIRLNIRDRQGMTPFACAMTHKNNKAAEAILKREPGAAEQVDNKGRNFLHVAVQNS
DIESVLFLISVQANVNSRVQDSAKLSPLHLAVQAGSEIIVRNLLLAGAKVNELTKHRQTALHLAAQQDLSTICSVLIENG
VDFAAVDENGNNALHLAVMQGRLNNVRALLTESNVDAEAYNLRGQSPMHVLGQYGKENAAAIFELFLECMPEYPLDKPD
N
EGNTVLLLAYMKGNANLCRAIVRAGARLGVTNNQGINIFNYQVATKQLLFRLLDMLSKEPPWCDGSNCYECITKFGVTTR
KHHCRHCGRLLCHKCSIKEIPIIKFDLNKPVRVCDICFDVLTLGGVS
>XP_005157097.1 zinc finger FYVE domain-containing protein 21 isoform X1 [Danio rerio]
MSAVPDGKKLVRSPSGLRMVPENGAFNSPFTLDEPQWVPDKECPRCMQCDTKFDFITRKHHCRRCGRCFCDKCCSQKVAL
PRMCFVDPVRQCAECSLISQKEVEFYDKQLKVLTAGGTFVVKVGSSEKSETMVCRLSNNHRYLFLDGNSHFEVELSRISS
MQVLTEGSTPGEKDICSYTSLLDNQISEGGTLRASGMVLQYKPPGSLDLQQLNMDTADDKRIASAWLAAMHKAAKLLYES
KDQ

>NP_001153818.1 zinc finger FYVE domain-containing protein 21 [Danio rerio]
MSAVPDGKKLVRSPSGLRMVPENGAFNSPFTLDEPQWVPDKECPRCMQCDTKFDFITRKHHCRRCGRCFCDKCCSQKVAL
PRMCFVDPVRQCAECSLISQKEVEFYDKQLKVLTAGGTFVVKVGSSEKSETMVCRLSNNHRYLFLDGNSHFEVELSRISS
MQVLTEGSTPGGGTLRASGMVLQYKPPGSLDLQQLNMDTADDKRIASAWLAAMHKAAKLLYESKDQ
>XP_021336159.1 zinc finger FYVE domain-containing protein 21 isoform X2 [Danio rerio]
MSAVPDGKKLVRSPSGLRMVPENGAFNSPFTLDEPQWVPDKECPRCMQCDTKFDFITRKHHCRRCGRCFCDKCCSQKVAL
PRMCFVDPVRQCAECSLISQKEVEFYDKQLKVLTAERRSSDRFGTNEG
>NP_991308.1 WD repeat and FYVE domain-containing protein 2 [Danio rerio]
MAAEIHPRPQTRKPVLLSKIEGFQDVVNTAVIIPKEDGVISVSQDRTIRVWLKRDSGQYWPSVYHTMPVACSCMSFNPET
RRISVGLENGTVSEFVLSEDYNQMTPARTYQAHQGGVTVVLFVLEMEWVLSTGQDKSFTWHCSESGQQLGTYRTTAWVSG
LQFDVETRHAFVGDHSGQVTILKLEQDNCSLVTTFKGHTGNVTALCWDPVQRVLFSGSSDHSIIMWDIGGRKGTAIELQG
HNDKVQGLCYAPHTRQLISCGSDGGIVIWNMDVTRQETPEWLDSDSCQKCEQPFFWNFKQMWDSKKIGLRQHHCRKCGQA
VCGKCSSKRSTIPLMGFEFEVRVCDSCHDLITDEERAPTATFHDSKHSIVHMQYEPTRGWLLTSGTDKVIKLWDMTPVVS
>XP_009295365.1 FYVE and coiled-coil domain-containing protein 1-like isoform X1 [Danio rerio]
MATGAAVGDSQIQRIIRDLRDAVSELTKEYKESGEPITDDSSNLHKFSYKLEYLLQFDQKEKTTFLGSRKDYWDYFSDCL
AKIKGANDGIRFVKSISELKTSLGKGRAFIRYSLVHQRLADTLQQCLMNSRVTSDWYNPRSPFLKPHLSVDIISYLYELN
DVQFDVASRGHDLDAEWPTFARRTLGMTSSPSHLWKAPSRSSSINSLASTYSQQHHEFPGSPDFGPGLLSDMSMQNSSIL
NDTSMSAIDELRLELDQSELKQRELIDRIQQLGDEGSELRGVVVELQRQLDVSLAAQGNHQELQRNLEVLIESEHALSRE
VEVLRDRETRREVSHKDLQDMLAAAERKNEELMTRLDGVLDEKGQRAASDFNSAQKIHELLNELKEAEKKRMDALAEGEE
KRRHAEHLAEEVKVKDEALKEAEVKMAAWMEKGEQLQTRAVEQRNFMEKLQGALAVREKETSNLQRQLRDLQNSLENM
EK
QANVEKKRMQDDKEELEMKMNGLEGLLQSLRTQLKVKESDLLSSTKRVHFLERESEKLRSENQKLEYELENSTKKEAKKI
DEYKDSCAKLIEQNTKLLQTVNKNEESKKELLENKSSLESELAGLRASEKQLRAQIDDAKVTVDEREQRLREENRNLDES
LQKANMQLEESESSIRQKEQENKDLMEVQVTLKSALAAMQKEIRDINNQIGELEKNLGVARCNEANLNAQLKDKATQLED
REKLCEELQGRVEELESRQRDLEVEKTKAERAFVKQTEMIQSLEAQRNLAEKTQLEKSTCQAKETKEMALKLTLLEDQLG
LSAKEVSKLQEEVVNLRAKLHSAVEEKDKTQAKLEVTEASCAELRILTEHLKKQAEEQNRLHVSELLQSSEHVDKLTSQL
NQETSAHEKTTAALASAKEDLVALKAQNERMVLENAETRESLHRVNTEMAELGMTICKLTAEREEARERWAAEAVRIQEL
QQHGVKETERLNASLVALHQENSSLQEELQQTDKLSETMLELKQLLDKTEGERDAAREEITAVKFQMSTESMSLKHQMKS
LQEEIDGLKDQLDTERKKKSELEAKLSELEGANVEYSRLIEEKDSHITYCETLLRESESETQQLQERASRSKEALSDVEK
EREELKQKLDQVLMETQNQHLRMSAELEDLGQTKVNLEERLIELIRDKDALWQKSDALEFEQKLRAEEQWWLVDKEATHC
LGCQGQFTWWLRRHHCRLCGRIFCYYCSNNYVMTKNSKKERCCRECYTQHGAVVERFTKAELNSSSDNQPSNTAAQLPPP
PYIPTPRVTVTDPGERADDGAFDIITDEEVNQIYDSDSRTTNGSHEEEGGQTGDNSVSSDVTSEEQEDMISVQDAEITLL
KSGELSASVPLDIDEIPHFGDGSRELFVKSSCYSTIPITVGDRGPIISWVFSSEPKSISFTVVYRETLDTPVEQAKVLIP

LTRCNSHKETIQGQLKVRNAGVYTLIFDNSFSRFLSKKVNYHLTIEKPVIYDGSDFP
>XP_021332794.1 FERM, RhoGEF and pleckstrin domain-containing protein 2 isoform X5 [Danio rerio]
MGEIEGSYRSLQTPAGSRLGGQHNTGISTLEPGQSLSSAMGAGGKGHNKGLQIRVQGLDEAQEFYELESKADGQLLLSDV
FRRINLIESDYFGLEFQNLQMNWVWLDPSKLIVKQVRRPMNTLFRLSVKFFPPDPGQLQEEFTRYLFSLQIKRDLLDGRL
SCTENTAALLASHLVQSEIGDYDDLADREFLKMNKLLPCQEHVQEKIMELHRRHTGQTPAESDFQVLEIARKLEMFGVRF
HPAADREGTKINLAVAHMGLQVFQGHTKINTFNWSKIRKLSFKRKRFLIKLHPEVHGPHQDTLEFLMGSRDQCKIFWKNC
VEHHSFFRLLDQPQPKSKAIFFSRGSSFRYSGRTQKQLVEYVRDSGLRRTPYQRRNSKIRMSTRSLASDVPKQNLSFNDS
LRTHIPPSSITASFQSLHAPSVSMRAETPNQLLHLHQTQHQPLRAPSPMKQDPIKTNSQSTYAFPDSIAIDSPQLSPFND
KGPLCLSPSFQMSTLSLPGQAPSPLQSPILNEVGTARLDEDEEGRRKRYPTDKAYFIAKEILTTERTYLKDLEVITVWFR
SAVIKENAMPEGLMTLLFSNIDPIYEFHRGFLKEIDQRLALWEGRSNAHVKGDYQRIGDVMLRNMCSLKEFTGYLQKHDE
VLTELEKATKRVKKLETVYKEFELQKVCYLPLNTFLLKPIQRLMHYKLILERLCKHYSPQHRDYDDCKEALKEVAEIATQ
LQSSLIRLENFQKLTELQRDLIGIENLTAPGREFIREGCLYKLTKKGLQQRMFFLFSDMLLYTSKGVTATNQFKVHGQLP
LHGMILIVLDAPVEESENEWSVPHCFTIYSAQRTIVVAASSKVEMNKWIEDLNMAIEMSKKCQEKSDVLLDPSLCDRSNR
SSDEVSLEQESEDDMNSSRCSLDKQSHHRANTTMHVCWHRNTSVSMSDHSLAVEYSVLQPCLQSFPLAICSVRARDQTPS
HMCAGTATRTCLSVTICA
>XP_021332793.1 FERM, RhoGEF and pleckstrin domain-containing protein 2 isoform X4 [Danio rerio]
MGEIEGSYRSLQTPAGSRLGGQHNTGISTLEPGQSLSSAMGAGGKGHNKGLQIRVQGLDEAQEFYELESKADGQLLLSDV
FRRINLIESDYFGLEFQNLQMNWVWLDPSKLIVKQVRRPMNTLFRLSVKFFPPDPGQLQEEFTRYLFSLQIKRDLLDGRL
SCTENTAALLASHLVQSEIGDYDDLADREFLKMNKLLPCQEHVQEKIMELHRRHTGQTPAESDFQVLEIARKLEMFGVRF
HPAADREGTKINLAVAHMGLQVFQGHTKINTFNWSKIRKLSFKRKRFLIKLHPEVHGPHQDTLEFLMGSRDQCKIFWKNC
VEHHSFFRLLDQPQPKSKAIFFSRGSSFRYSGRTQKQLVEYVRDSGLRRTPYQRRNSKIRMSTRSLASDVPKQNLSFNDS
LRTHIPPSSITASFQSLHAPSVSMRAETPNQLLHLHQTQHQPLRAPSPMKQDPIKTNSQSTYAFPDSIAIDSPQLSPFND
KGPLCLSPSFQMSTLSLPGQAPSPLQSPILNERYPTDKAYFIAKEILTTERTYLKDLEVITVWFRSAVIKENAMPEGLMT
LLFSNIDPIYEFHRGFLKEIDQRLALWEGRSNAHVKGDYQRIGDVMLRNMCSLKEFTGYLQKHDEVLTELEKATKRVKKL
ETVYKEFELQKVCYLPLNTFLLKPIQRLMHYKLILERLCKHYSPQHRDYDDCKEALKEVAEIATQLQSSLIRLENFQKLT
ELQRDLIGIENLTAPGREFIREGCLYKLTKKGLQQRMFFLFSDMLLYTSKGVTATNQFKVHGQLPLHGMILIVLDAPVEE
SENEWSVPHCFTIYSAQRTIVVAASSKVEMNKWIEDLNMAIEMSKKCQEKSDVLLDPSLCDRSNRSSDEVSLEQESEDDM
NSSRCSLDKQSHHRANTTMHVCWHRNTSVSMSDHSLAVENQLSGYLLRKFKNSNGWQKLWVVFTNFCLFFYKTHQDDFP
L
ASLPLLGYTVSTPGESDSIHKEYVFKLQFKSHVYFFRAESEYTFERWMEVIKSAASATGRMSLLVPKGPAEVNVGN
>XP_009300696.1 FERM, RhoGEF and pleckstrin domain-containing protein 2 isoform X1 [Danio rerio]
MGEIEGSYRSLQTPAGSRLGGQHNTGISTLEPGQSLSSAMGAGGKGHNKGLQIRVQGLDEAQEFYELESKADGQLLLSDV
FRRINLIESDYFGLEFQNLQMNWVWLDPSKLIVKQVRRPMNTLFRLSVKFFPPDPGQLQEEFTRYLFSLQIKRDLLDGRL

SCTENTAALLASHLVQSEIGDYDDLADREFLKMNKLLPCQEHVQEKIMELHRRHTGQTPAESDFQVLEIARKLEMFGVRF
HPAADREGTKINLAVAHMGLQVFQGHTKINTFNWSKIRKLSFKRKRFLIKLHPEVHGPHQDTLEFLMGSRDQCKIFWKNC
VEHHSFFRLLDQPQPKSKAIFFSRGSSFRYSGRTQKQLVEYVRDSGLRRTPYQRRNSKIRMSTRSLASDVPKQNLSFNDS
LRTHIPPSSITASFQSLHAPSVSMRAETPNQLLHLHQTQHQPLRAPSPMKQDPIKTNSQSTYAFPDSIAIDSPQLSPFND
KGPLCLSPSFQMSTLSLPGQAPSPLQSPILNEVGTARLDEDEEGRRKRYPTDKAYFIAKEILTTERTYLKDLEVITVWFR
SAVIKENAMPEGLMTLLFSNIDPIYEFHRGFLKEIDQRLALWEGRSNAHVKGDYQRIGDVMLRNMCSLKEFTGYLQKHDE
VLTELEKATKRVKKLETVYKEFELQKVCYLPLNTFLLKPIQRLMHYKLILERLCKHYSPQHRDYDDCKEALKEVAEIATQ
LQSSLIRLENFQKLTELQRDLIGIENLTAPGREFIREGCLYKLTKKGLQQRMFFLFSDMLLYTSKGVTATNQFKVHGQLP
LHGMILIVLDAPVEESENEWSVPHCFTIYSAQRTIVVAASSKVEMNKWIEDLNMAIEMSKKCQEKSDVLLDPSLCDRSNR
SSDEVSLEQESEDDMNSSRCSLDKQSHHRANTTMHVCWHRNTSVSMSDHSLAVENQLSGYLLRKFKNSNGWQKLWVVFT
N
FCLFFYKTHQDDFPLASLPLLGYTVSTPGESDSIHKEYVFKLQFKSHVYFFRAESEYTFERWMEVIKSAASATGRMSLLV
PKGPAEVNVGN
>XP_001344703.1 zinc finger FYVE domain-containing protein 1 [Danio rerio]
MSGQGSSAEKGLNGVLVCQESYACGGTDEAAFECDECGSLQCIRCELELHRQDRMRNHDRIRIAPGHVPYCDSCKGDGGV
PNGGRLRAVVRCQGCKINLCLDCQKRTHSGVSKRKHPLTAYPPPSKPPENNCSTGDEQLDELKAKLEKVTSFLLVDENED
MQVKNEEDFVRKLGCRPEELLKVVSIFGNTGEGKSHTLNHTFFLGREVFKTSPTQESCTVGVWAAMDPVHKVVVVDTEGL
LGAGTNQGQRTRLLLKVLAVSDLVIYRTHADRLHDDLFKFLGDASDAYLKHFTKELKATTARCGLDVPLSTLGPAVIIFH
ETVHTKLLGSDKPSESAERLLQERFRKLGLFPEAFSSIQYRGTRTHNPPTDFSGLLRSVEQQLDNNSTRSPRSAGVIHKA
LQALSECFSGEIPDEHLASNSFFPDEYFTCSSICLSCGSGCKNSMNHLREGVVHEAKHRCRYSAHYDNRIYTCKACYESG
KEVIVVPKTTASSDSPWFGLAIYAWSGYVIECPNCSVIYRSRQYWYGNQDPVDTVVRTEIQHVWPGSDKFLKDNNNAAQR
LLDGVNFMAQSVSELSVKPAKAVTAWLTDQIAPAYWKPNSLILRCYKCGAGFEDNDTKHHCRACGEGFCDGCSSKTRPVP
ERGWGLAPVRVCDACFQNRGIPEELLDAALEEEEGGTLIARKVGEAVQNTLGAVVTAIDIPLGLVKDAARPAYWVPDQDI
RCCNQCQREFNARLSIHHCRACGQGVCNDCSPDRRAVPSRGWDHPVRVCTTCNQKSGEL
>XP_021333893.1 myotubularin-related protein 3 isoform X1 [Danio rerio]
MEEEGQQSLECIQANQIFPRKPPVLEEEDLQVPFPELHGEFTEYVGRAEDAVIAMSSYRLHIKFKESVVNNCSCEVSVPL
QLIECVECRDMFQLHITCKDCKIVRCQFSTFEQCQEWLKRLTAAVRPPSRIEELFSFAFHAWCVDLYTGEKEQHGDLCRP
GDHVMSRFHNEVERMGFDTQNAWRVSEINNKYKLCSSYPQLLLVPAWITDKELENVAAFRSWKRIPAVVYRHQSTGAVIA
RCGQPEVSWWGWRNADDEHLVQSIARACAVDSSSCKGVSNGSLSREYTNGADLSDVDFESSMTNSSEVETLAIQPRKLLI
LDARSYAAAVANRAKGGGCECPEYYPNCEVVFMGMANIHSIRKSFQSLRFLCTQMPDPANWLSALESTKWLQHLSLLLKA
SLLVSNAVDRDHRPVLVHCSDGWDRTPQIVALAKLLLDPYYRTIEGFQVLVETEWLDFGHKFADRCGHGENAEDLNERCP
VFLQWLDCVHQLQRQFPCSFEFNEAFLVKLVQHTYSCLFGTFLCNSGKEREDRRIQERTCSVWSLLRAANRSFGNMLYSS
HSETVLHPVCHVRNLMLWTAVYLPSSSPTTPSDDSCAPYPAPGANPEDQPLGRRPKTRSFDNLPSACEVGNPLTSNRRSS

DPSLNEKWQDHRRSLELNIGAGTDGAASPEPDERVNGQTMVGIMGTFSNGGTENGEGLKDVRLPMIDGVDEAELTVGVAV
GQMENILQEATKDESAPDTRSVAKPNETSAVNGKTDDIAQEGRTEDDLESKSLEDGNVNGHCSEDGNSEASSALSQDTSE
SQDSEEIEKCTTQEDDSSCNPTILTPNGLRTLTNGHGVEPSIEQAEKRLSLLESSTETLTEDLGVRPESLGPLSIPPPLK
ALAESSCLKQGLRTAARTLNNTPKRASLSAFPPASTDLLHPMCNGDSPDPEPSTPRTNGERATLSRQVSLASCGSLTLHA
RGTCSHHRCLHLGFLGRPSFSPPEPPSSSSRSHLDDDGLTLHTDAIQQRLRQIEAGHQLEVEALKRQVQELWSRLESHQA
AGMLRLNGDIGDEVTSITDSDFNLEPNCLSRCSTELFSEASWEQVDKQDTEVTRWYPDHLAAQCYGCERGFWLATRKHHC
RGKERMEEVWNCGNVFCGSCCDQKIPVPSQQLFEPSRVCRSCFSNLQPPAPPLDLELDKPITASSN
>XP_021332792.1 FERM, RhoGEF and pleckstrin domain-containing protein 2 isoform X3 [Danio rerio]
MGEIEGSYRSLQTPAGSRLGGQHNTGISTLEPGQSLSSAMGAGGKGHNKGLQIRVQGLDEAQEFYELESKADGQLLLSDV
FRRINLIESDYFGLEFQNLQMNWVWLDPSKLIVKQVRRPMNTLFRLSVKFFPPDPGQLQEEFTRYLFSLQIKRDLLDGRL
SCTENTAALLASHLVQSEIGDYDDLADREFLKMNKLLPCQEHVQEKIMELHRRHTGQTPAESDFQVLEIARKLEMFGVRF
HPAADREGTKINLAVAHMGLQVFQGHTKINTFNWSKIRKLSFKRKRFLIKLHPEVHGPHQDTLEFLMGSRDQCKIFWKNC
VEHHSFFRLLDQPQPKSKAIFFSRGSSFRYSGRTQKQLVEYVRDSGLRRTPYQRRNSKIRMSTRSLASDVPKQNLSFNDS
LRTHIPPSSITASFQSLHAPSVSMRAETPNQLLHLHQTQHQPLRAPSPMKQDPIKTNSQSTYAFPDSIAIDSPQLSPFND
KGPLCLSPSFQMSTLSLPGQAPSPLQSPILNEVGTARLDEDEEGRRKRYPTDKAYFIAKEILTTERTYLKDLEVITVWFR
SAVIKENAMPEGLMTLLFSNIDPIYEFHRGFLKEIDQRLALWEGRSNAHVKGDYQRIGDVMLRNMCSLKEFTGYLQKHDE
VLTELEKATKRVKKLETVYKEFELQKVCYLPLNTFLLKPIQRLMHYKLILERLCKHYSPQHRDYDDCKEALKEVAEIATQ
LQSSLIRLENFQKLTELQRDLIGIENLTAPGREFIREGCLYKLTKKGLQQRMFFLFSDMLLYTSKGVTATNQFKVHGQLP
LHGMILIVLDAPVEESENEWSVPHCFTIYSAQRTIVVAASSKVEMNKWIEDLNMAIEMSKKCQEKSDVLLDPSLCDRSNI
FSSPAVSPELPPRYLLGQGQRPNTITHVCWYRNQNLSLSDYLRMNQNQLSGYLLRKFKNSNGWQKLWVVFTNFCLFFYKT
HQDDFPLASLPLLGYTVSTPGESDSIHKEYVFKLQFKSHVYFFRAESEYTFERWMEVIKSAASATGRMSLLVPKGPAEVN
VGN
>XP_009302003.1 myotubularin-related protein 3 isoform X2 [Danio rerio]
MEEEGQQSLECIQANQIFPRKPPVLEEEDLQVPFPELHGEFTEYVGRAEDAVIAMSSYRLHIKFKESVVNVPLQLIECVE
CRDMFQLHITCKDCKIVRCQFSTFEQCQEWLKRLTAAVRPPSRIEELFSFAFHAWCVDLYTGEKEQHGDLCRPGDHVMSR
FHNEVERMGFDTQNAWRVSEINNKYKLCSSYPQLLLVPAWITDKELENVAAFRSWKRIPAVVYRHQSTGAVIARCGQPEV
SWWGWRNADDEHLVQSIARACAVDSSSCKGVSNGSLSREYTNGADLSDVDFESSMTNSSEVETLAIQPRKLLILDARSYA
AAVANRAKGGGCECPEYYPNCEVVFMGMANIHSIRKSFQSLRFLCTQMPDPANWLSALESTKWLQHLSLLLKASLLVSNA
VDRDHRPVLVHCSDGWDRTPQIVALAKLLLDPYYRTIEGFQVLVETEWLDFGHKFADRCGHGENAEDLNERCPVFLQWLD
CVHQLQRQFPCSFEFNEAFLVKLVQHTYSCLFGTFLCNSGKEREDRRIQERTCSVWSLLRAANRSFGNMLYSSHSETVLH
PVCHVRNLMLWTAVYLPSSSPTTPSDDSCAPYPAPGANPEDQPLGRRPKTRSFDNLPSACEVGNPLTSNRRSSDPSLNEK
WQDHRRSLELNIGAGTDGAASPEPDERVNGQTMVGIMGTFSNGGTENGEGLKDVRLPMIDGVDEAELTVGVAVGQMENIL
QEATKDESAPDTRSVAKPNETSAVNGKTDDIAQEGRTEDDLESKSLEDGNVNGHCSEDGNSEASSALSQDTSESQDSEEI

EKCTTQEDDSSCNPTILTPNGLRTLTNGHGVEPSIEQAEKRLSLLESSTETLTEDLGVRPESLGPLSIPPPLKALAESSC
LKQGLRTAARTLNNTPKRASLSAFPPASTDLLHPMCNGDSPDPEPSTPRTNGERATLSRQVSLASCGSLTLHARGTCSHH
RCLHLGFLGRPSFSPPEPPSSSSRSHLDDDGLTLHTDAIQQRLRQIEAGHQLEVEALKRQVQELWSRLESHQAAGMLRLN
GDIGDEVTSITDSDFNLEPNCLSRCSTELFSEASWEQVDKQDTEVTRWYPDHLAAQCYGCERGFWLATRKHHCRGKERME
EVWNCGNVFCGSCCDQKIPVPSQQLFEPSRVCRSCFSNLQPPAPPLDLELDKPITASSN
>XP_017211771.1 WD repeat and FYVE domain-containing protein 3 isoform X6 [Danio rerio]
MLAEHRENSRAGFGLKKPLKMNMVKRIMGRPRQEECSPQDNALGLMHLRRLFSELCHPPRHMTQKEQEEKLYMMLPVFN
R
VFGNAPPSTMNEKFSDLLQFTTQVSRLMVTEIRRRASNKSTEAASRAIVQFLEVNQSEETSRGWMLLTTINLLASSGQKT
VDCMTTMSVPSTLVKCLYLFFDLPHMPEVPAATQTELPLADRRALLQKVFVQILVKLCSFVSPAEELAQKDDLQLLFSAI
TSWCPPHNLPWRKSAGEVLTTISRHGLSVNVVKYIHEKECLSTCIQNMQQSDDLSPLEIVEMFAGLSCFLKDSSDVSQTL
LDDFRMCQGYTFLIDLMIRLEQAKEDESKDALKDLVNLVTALTTYGVSELKPAGLTTGAPFLLPGFVVPQPSGKGHTVRN
IQAFSVLQNAFLKAKSGRLARMILEAIANIYAADYANYFILEAQHTLSQFAERVTKLPEVQSKYFELLEFVVFGLNYVPC
KELFSVSVLLKSSTSYGCSITATRTLLKLGRHHPVFSDVFREVGLLEVLVNLLHKYAALLKDPTQTHSDQGDSKNNVAEE
QKQLAWLVMETLTVLLQGSNTNAGLFREFGGARCVHNIVKYRQCREHALMIIQQLVLSPSGDDDMGTLLGLMHSAPTAEL
QLKTDILRALLAVLRESHRTRTVFRKVGGFVYVTSLLLAMERSLCSPPVIGWEKVNQNKVFELLHTIFCTLTAAMRYEPA
NSHFFRTEIQYEKLADAVRLLGCFSDAKKVGPATVFPSNAQPFQRLLEDEIIPAENVCPTLKHCSKLFIYMYKMATDSFD
RHGYSSSSNSASGKALTDLKLHLGNSSQHSSDPVVIHPGAVLAMLDLLPSVSSDTQPEHALDLQLAVANILQLLVHSERN
QQILCEAGLHSRLLQRCSCALGDEDHPLHPPLQRMFERLASQALEPMVLREFLRLGNPLNCGAWDKKLLKQYRVHKPSSL
SYEAEMRNSMTLSMEGFGPDSVFTINEDNSQYRISRSLVRSAEGSTVPLTRVKCLVSMTTPHDIRLHGSSVTPAFVELDT
SLEGFGCLFLPSLAPHNAPSSNANTSGVSDGAVVSGMGNGERFFPPPSGLSYSTWFCVERFSTPPQSHPVRLLTVVRRAT
SSEQHYVCLAIVLSAKDRSLIVSTKEELLQSYVDDFSEESSFYEILPCCARFRCADLITEGQWHHLLLVMSKGMLKNSMA
TLYIDGQMVSTVKLHYVHNSPGGSASANPPVVSTVYSYIGTPPAQRQLSTLVWRLGPTHFLEEVLPAASVAAIYELGPNY
VGSFQAVYLPCKDSKTEAVPPCPVGLVPEEKVSFSLFAHSVSALTVAKIRKVYNKLDSKAIAKQLGVSSHESATPVKLVH
NAVGHLNGSARTVGAAVIGYLGVRAFVTKPVATNLQYIGGAAAILGLVAMASDVEGLYAAVKALVCVVKSNPLASKEMER
IKGYQLLAMLLKKKRPLLNSHILHLTFSLVGTVDSGHETSIIPNSTAFQDLLCDFEVWLHAPYELHLSLFEHFIELLTES
SEAAKNAKLLREFQLIPKLLLTLRDQSLSQPTITAIGNVLSLLLQGFPNTYDLLRFGQFISSTLPTFAVCEKFVIMENTN
EEKIDRGADEEFGGLLSSNLILLRNRLLDILLKQLFSCKEKGTVNAQACEELVRTLGFDWLLMFMEEHIHSSTVTAALLI
LVVLLSNQSILSRFKEGLSGGGWLDHTDSVLTNKIGTVLGFNVGRSAGGRSTLREINRDACHFPGFPVLQTLLPKHTNVP
EIYFMLMALFFQQPITELPESLQVHFDLDSIWTFIFGVPASSATVVGSLHNMCTETAFLLLAMLRSMLNLPWQSEEEGSW
LREYPITLMQFFRYLYHNIPDLAPMWHSPDFLCTLAATVFPFNIRPYSEMVSDLDDEASSPTEEFKAFVTDTGMNRSQSE
YCNVGNKTYLTNHPAKKYVFDFMRLLIMDNLCMTPASKQTPIIDLLLEASPERSTRTQQKEFQSYILDSVMEQLLAADVL
MGEEASLPITTGGSYQVLVNNVFYFTQRVVDKLWQGMFNKESKLVVDFIVQLITQSKRRSQGLSLDSVYHCLNRTILYQL

SRPHKTVPQQVTLLDSLRVLTVNRNLVLGPGNHDQEFVACLAHCLINLHTGSSVEGFGLEAEARMTTWHVAVPSENESDS
VQSQDVSEGRQLLLKAVNRVWTELMHSKRQMLEDIFKVSLPVNDRGHVDIGTARPALEEPAAKSWQNHLVHEKKCISRGE
TVAPANQSKLSRVSSGFGLSKLTGSRRSKKESGMNKNNLSAQETFQWMFTHIAVVRDLVAMQYKEHQERQQNALKYVMD
E
WAGIEYELLRERGLWGPPIGSHLDKFQLDMTEGPCRMRKKMVRNDMFYIHYPYVPDPEPNASTQKPARYRRAISYDSKEY
YMRLLSGNPGMYQHSIEQNTEGETTQQEPEHGEDTIARVKGLVKAPLKRSRSTADGADEESQDQLQELLESGAMDEEQKT
DNTTLLRLLEEGEKIQHMYRCARVQGLDTSEGLLLFGKEHFYVIDGFTMTVTREIRDIETLPPNMHEPIIPRGARQSKSQ
LKRTCSIFAYEDIREVHKRRYLLQPMAAEVFSGDGRNYLLAFQKGVRNKVYQRFLAVVPSLADSSESVSGQRPNTSVEQG
SGLLSTLVGEKSVTQRWERGEISNFQYLMHLNTLAGRSYNDLMQYPVFPWILADFDSEELDLNNPKTFRNLSKPMGAQTD
DRLIQYKKRFKDWEDPNGETPAYHYGTHYSSAMIVASYLVRMEPFTQIFLRLQGGHFDLADRMFHSVREAWLSASKHNMA
DVKELIPEFFYLPEFLLNSNNFDLGSKQNGTKLGDVILPPWAKGDPREFIRVHREALECDYVSAHLHEWIDLIFGYKQQG
PPAVEAVNVFHHLFYEGQVDIYNINDPLKETATIGFINNFGQIPKQLFKKPHPPKRVRSKSNGEAPGGPVTLNSTLDKIF
FHHLDNLRPSLTPVKELKEPVGQIVCTDKGILAVEQNKVLVPPAWNKTFAWGYADLSCRLSNYESDKAVVVYECLSEWGQ
ILCAICPNPKLVITGGFSTAICVWETGSSKEKAKSLTLKQALLGHTDAVTCLTASSAYHIVVSGSRDRTCIIWDLNKLSF
VTQLRGHRAPVSALCINELTGDIVSCAGTYIHVWSINGSPVASANTFTGRSQQILCCCVSEMNEWDTQNVIVTGHSDGVV
RFWRIEFLQVPETPAPEPIEPPDVPDCCGEIEGRDAPDEDSSESEGEEASLSQEPKPRSSNSQPSSAVHRPKPRTGASWS
VDSSSDDSRRWSDTLSIDEKEGFVFVNYSEGQVRPGVPVQPHSQQPSGPSHGPVPQPLQPSTMEARSYNKLKPGYRWERQ
LVFRSKLTMHTAFDRKDNAHPAEITALAISKDHSKILVGDGRGRVFSWSVSDQPGRSTADHWVKDEQVDSCSGCTVRFSL
TERRHHCRNCGQVFCQKCSRFQSEIKRLKISSPVRVCQNCYYNLQHERSSEENPRN
>XP_017211770.1 WD repeat and FYVE domain-containing protein 3 isoform X5 [Danio rerio]
MLAEHRENSRAGFGLKKPLKMNMVKRIMGRPRQEECSPQDNALGLMHLRRLFSELCHPPRHMTQKEQEEKLYMMLPVFN
R
VFGNAPPSTMNEKFSDLLQFTTQVSRLMVTEIRRRASNKSTEAASRAIVQFLEVNQSEETSRGWMLLTTINLLASSGQKT
VDCMTTMSVPSTLVKCLYLFFDLPHMPEVPAATQTELPLADRRALLQKVFVQILVKLCSFVSPAEELAQKDDLQLLFSAI
TSWCPPHNLPWRKSAGEVLTTISRHGLSVNVVKYIHEKECLSTCIQNMQQSDDLSPLEIVEMFAGLSCFLKDSSDVSQTL
LDDFRMCQGYTFLIDLMIRLEQAKEDESKDALKDLVNLVTALTTYGVSELKPAGLTTGAPFLLPGFVVPQPSGKGHTVRN
IQAFSVLQNAFLKAKSGRLARMILEAIANIYAADYANYFILEAQHTLSQFAERVTKLPEVQSKYFELLEFVVFGLNYVPC
KELFSVSVLLKSSTSYGCSITATRTLLKLGRHHPVFSDVFREVGLLEVLVNLLHKYAALLKDPTQTHSDQGDSKNNVAEE
QKQLAWLVMETLTVLLQGSNTNAGLFREFGGARCVHNIVKYRQCREHALMIIQQLVLSPSGDDDMGTLLGLMHSAPTAEL
QLKTDILRALLAVLRESHRTRTVFRKVGGFVYVTSLLLAMERSLCSPPVIGWEKVNQNKVFELLHTIFCTLTAAMRYEPA
NSHFFRTEIQYEKLADAVRLLGCFSDAKKVGPATVFPSNAQPFQRLLEDEIIPAENVCPTLKHCSKLFIYMYKMATDSFD
SRHGYSSSSNSASGKALTDLKLHLGNSSQHSSDPVVIHPGAVLAMLDLLPSVSSDTQPEHALDLQLAVANILQLLVHSER
NQQILCEAGLHSRLLQRCSCALGDEDHPLHPPLQRMFERLASQALEPMVLREFLRLGNPLNCGAWDKKLLKQYRVHKPSS

LSYEAEMRNSMTLSMEGFGPDSVFTINEDNSQYRISRSLVRSAEGSTVPLTRVKCLVSMTTPHDIRLHGSSVTPAFVELD
TSLEGFGCLFLPSLAPHNAPSSNANTSGVSDGAVVSGMGNGERFFPPPSGLSYSTWFCVERFSTPPQSHPVRLLTVVRRA
TSSEQHYVCLAIVLSAKDRSLIVSTKEELLQSYVDDFSEESSFYEILPCCARFRCADLITEGQWHHLLLVMSKGMLKNSM
ATLYIDGQMVSTVKLHYVHNSPGGSASANPPVVSTVYSYIGTPPAQRQLSTLVWRLGPTHFLEEVLPAASVAAIYELGPN
YVGSFQAVYLPCKDSKTEAVPPCPVGLVPEEKVSFSLFAHSVSALTVAKIRKVYNKLDSKAIAKQLGVSSHESATPVKLV
HNAVGHLNGSARTVGAAVIGYLGVRAFVTKPVATNLQYIGGAAAILGLVAMASDVEGLYAAVKALVCVVKSNPLASKEM
E
RIKGYQLLAMLLKKKRPLLNSHILHLTFSLVGTVDSGHETSIIPNSTAFQDLLCDFEVWLHAPYELHLSLFEHFIELLTE
SSEAAKNAKLLREFQLIPKLLLTLRDQSLSQPTITAIGNVLSLLLQGFPNTYDLLRFGQFISSTLPTFAVCEKFVIMENT
NEEKIDRGADEEFGGLLSSNLILLRNRLLDILLKQLFSCKEKGTVNAQACEELVRTLGFDWLLMFMEEHIHSSTVTAALL
ILVVLLSNQSILSRFKEGLSGGGWLDHTDSVLTNKIGTVLGFNVGRSAGGRSTLREINRDACHFPGFPVLQTLLPKHTNV
PEIYFMLMALFFQQPITELPESLQVHFDLDSIWTFIFGVPASSATVVGSLHNMCTETAFLLLAMLRSMLNLPWQSEEEGS
WLREYPITLMQFFRYLYHNIPDLAPMWHSPDFLCTLAATVFPFNIRPYSEMVSDLDDEASSPTEEFKAFVTDTGMNRSQS
EYCNVGNKTYLTNHPAKKYVFDFMRLLIMDNLCMTPASKQTPIIDLLLEASPERSTRTQQKEFQSYILDSVMEQLLAADV
LMGEEASLPITTGGSYQVLVNNVFYFTQRVVDKLWQGMFNKESKLVVDFIVQLITQSKRRSQGLSLDSVYHCLNRTILYQ
LSRPHKTVPQQVTLLDSLRVLTVNRNLVLGPGNHDQEFVACLAHCLINLHTGSSVEGFGLEAEARMTTWHVAVPSENESD
SVQSQDVSEGRQLLLKAVNRVWTELMHSKRQMLEDIFKVSLPVNDRGHVDIGTARPALEEPAAKSWQNHLVHEKKCISRG
ETVAPANQSKLSRVSSGFGLSKLTGSRRSKKESGMNKNNLSAQETFQWMFTHIAVVRDLVAMQYKEHQERQQNALKYVM
D
EWAGIEYELLRERGLWGPPIGSHLDKFQLDMTEGPCRMRKKMVRNDMFYIHYPYVPDPEPNASTQKPARYRRAISYDSKE
YYMRLLSGNPGMYQHSIEQNTEGETTQQEPEHGEDTIARVKGLVKAPLKRSRSTADGADEESQDQLQELLESGAMDEEQK
TDNTTLLRLLEEGEKIQHMYRCARVQGLDTSEGLLLFGKEHFYVIDGFTMTVTREIRDIETLPPNMHEPIIPRGARQSKS
QLKRTCSIFAYEDIREVHKRRYLLQPMAAEVFSGDGRNYLLAFQKGVRNKVYQRFLAVVPSLADSSESVSGQRPNTSVEQ
GSGLLSTLVGEKSVTQRWERGEISNFQYLMHLNTLAGRSYNDLMQYPVFPWILADFDSEELDLNNPKTFRNLSKPMGAQT
DDRLIQYKKRFKDWEDPNGETPAYHYGTHYSSAMIVASYLVRMEPFTQIFLRLQGGHFDLADRMFHSVREAWLSASKHNM
ADVKELIPEFFYLPEFLLNSNNFDLGSKQNGTKLGDVILPPWAKGDPREFIRVHREALECDYVSAHLHEWIDLIFGYKQQ
GPPAVEAVNVFHHLFYEGQVDIYNINDPLKETATIGFINNFGQIPKQLFKKPHPPKRVRSKSNGEAPGGPVTLNSTLDKI
FFHHLDNLRPSLTPVKELKEPVGQIVCTDKGILAVEQNKVLVPPAWNKTFAWGYADLSCRLSNYESDKAVVVYECLSEWG
QILCAICPNPKLVITGGFSTAICVWETGSSKEKAKSLTLKQALLGHTDAVTCLTASSAYHIVVSGSRDRTCIIWDLNKLS
FVTQLRGHRAPVSALCINELTGDIVSCAGTYIHVWSINGSPVASANTFTGRSQQILCCCVSEMNEWDTQNVIVTGHSDGV
VRFWRIEFLQVPETPAPEPIEPPDVPDCCGEIEGRDAPDEDSSESEGEEASLSQEPKPRSSNSQPSSAVHRPKPRTGASW
SVDSSSDDSRRWSDTLSIDEKEGFVFVNYSEGQVRPGVPVQPHSQQPSGPSHGPVPQPLQPSTMEARSYNKLKPGYRWER
QLVFRSKLTMHTAFDRKDNAHPAEITALAISKDHSKILVGDGRGRVFSWSVSDQPGRSTADHWVKDEQVDSCSGCTVRFS

LTERRHHCRNCGQVFCQKCSRFQSEIKRLKISSPVRVCQNCYYNLQHERSSEENPRN
>XP_017211768.1 WD repeat and FYVE domain-containing protein 3 isoform X2 [Danio rerio]
MLAEHRENSRAGFGLKKPLKMNMVKRIMGRPRQEECSPQDNALGLMHLRRLFSELCHPPRHMTQKEQEEKLYMMLPVFN
R
VFGNAPPSTMNEKFSDLLQFTTQVSRLMVTEIRRRASNKSTEAASRAIVQFLEVNQSEETSRGWMLLTTINLLASSGQKT
VDCMTTMSVPSTLVKCLYLFFDLPHMPEVPAATQTELPLADRRALLQKVFVQILVKLCSFVSPAEELAQKDDLQLLFSAI
TSWCPPHNLPWRKSAGEVLTTISRHGLSVNVVKYIHEKECLSTCIQNMQQSDDLSPLEIVEMFAGLSCFLKDSSDVSQTL
LDDFRMCQGYTFLIDLMIRLEQAKEDESKDALKDLVNLVTALTTYGVSELKPAGLTTGAPFLLPGFVVPQPSGKGHTVRN
IQAFSVLQNAFLKAKSGRLARMILEAIANIYAADYANYFILEAQHTLSQFAERVTKLPEVQSKYFELLEFVVFGLNYVPC
KELFSVSVLLKSSTSYGCSITATRTLLKLGRHHPVFSDVFREVGLLEVLVNLLHKYAALLKDPTQTHSDQGDSKNNVAEE
QKQLAWLVMETLTVLLQGSNTNAGLFREFGGARCVHNIVKYRQCREHALMIIQQLVLSPSGDDDMGTLLGLMHSAPTAEL
QLKTDILRALLAVLRESHRTRTVFRKVGGFVYVTSLLLAMERSLCSPPVIGWEKVNQNKVFELLHTIFCTLTAAMRYEPA
NSHFFRTEIQYEKLADAVRLLGCFSDAKKVGPATVFPSNAQPFQRLLEDEIIPAENVCPTLKHCSKLFIYMYKMATDSFD
SRAEQVPPCLTHETSLPSPWGTPAITRKRHGYSSSSNSASGKALTDLKLHLGNSSQHSSDPVVIHPGAVLAMLDLLPSVS
SDTQPEHALDLQLAVANILQLLVHSERNQQILCEAGLHSRLLQRCSCALGDEDHPLHPPLQRMFERLASQALEPMVLREF
LRLGNPLNCGAWDKKLLKQYRVHKPSSLSYEAEMRNSMTLSMEGFGPDSVFTINEDNSQYRISRSLVRSAEGSTVPLTRV
KCLVSMTTPHDIRLHGSSVTPAFVELDTSLEGFGCLFLPSLAPHNAPSSNANTSGVSDGAVVSGMGNGERFFPPPSGLSY
STWFCVERFSTPPQSHPVRLLTVVRRATSSEQHYVCLAIVLSAKDRSLIVSTKEELLQSYVDDFSEESSFYEILPCCARF
RCADLITEGQWHHLLLVMSKGMLKNSMATLYIDGQMVSTVKLHYVHNSPGGSASANPPVVSTVYSYIGTPPAQRQLSTLV
WRLGPTHFLEEVLPAASVAAIYELGPNYVGSFQAVYLPCKDSKTEAVPPCPVGLVPEEKVSFSLFAHSVSALTVAKIRKV
YNKLDSKAIAKQLGVSSHESATPVKLVHNAVGHLNGSARTVGAAVIGYLGVRAFVTKPVATNLQYIGGAAAILGLVAMAS
DVEGLYAAVKALVCVVKSNPLASKEMERIKGYQLLAMLLKKKRPLLNSHILHLTFSLVGTVDSGHETSIIPNSTAFQDLL
CDFEVWLHAPYELHLSLFEHFIELLTESSEAAKNAKLLREFQLIPKLLLTLRDQSLSQPTITAIGNVLSLLLQGFPNTYD
LLRFGQFISSTLPTFAVCEKFVIMENTNEEKIDRGADEEFGGLLSSNLILLRNRLLDILLKQLFSCKEKGTVNAQACEEL
VRTLGFDWLLMFMEEHIHSSTVTAALLILVVLLSNQSILSRFKEGLSGGGWLDHTDSVLTNKIGTVLGFNVGRSAGGRST
LREINRDACHFPGFPVLQTLLPKHTNVPEIYFMLMALFFQQPITELPESLQVHFDLDSIWTFIFGVPASSATVVGSLHNM
CTETAFLLLAMLRSMLNLPWQSEEEGSWLREYPITLMQFFRYLYHNIPDLAPMWHSPDFLCTLAATVFPFNIRPYSEMVS
DLDDEASSPTEEFKAFVTDTGMNRSQSEYCNVGNKTYLTNHPAKKYVFDFMRLLIMDNLCMTPASKQTPIIDLLLEASPE
RSTRTQQKEFQSYILDSVMEQLLAADVLMGEEASLPITTGGSYQVLVNNVFYFTQRVVDKLWQGMFNKESKLVVDFIVQL
ITQSKRRSQGLSLDSVYHCLNRTILYQLSRPHKTVPQQVTLLDSLRVLTVNRNLVLGPGNHDQEFVACLAHCLINLHTGS
SVEGFGLEAEARMTTWHVAVPSENESDSVQSQDVSEGRQLLLKAVNRVWTELMHSKRQMLEDIFKVSLPVNDRGHVDIGT
ARPALEEPAAKSWQNHLVHEKKCISRGETVAPANQSKLSRVSSGFGLSKLTGSRRSKKESGMNKNNLSAQETFQWMFTHI

AVVRDLVAMQYKEHQERQQNALKYVMDEWAGIEYELLRERGLWGPPIGSHLDKFQLDMTEGPCRMRKKMVRNDMFYIH
YP
YVPDPEPNASTQKPARYRRAISYDSKEYYMRLLSGNPGMYQHSIEQNTEGETTQQEPEHGEDTIARVKGLVKAPLKRSRS
TADGADEESQDQLQELLESGAMDEEQKTDNTTLLRLLEEGEKIQHMYRCARVQGLDTSEGLLLFGKEHFYVIDGFTMTVT
REIRDIETLPPNMHEPIIPRGARQSKSQLKRTCSIFAYEDIREVHKRRYLLQPMAAEVFSGDGRNYLLAFQKGVRNKVYQ
RFLAVVPSLADSSESVSGQRPNTSVEQGSGLLSTLVGEKSVTQRWERGEISNFQYLMHLNTLAGRSYNDLMQYPVFPWIL
ADFDSEELDLNNPKTFRNLSKPMGAQTDDRLIQYKKRFKDWEDPNGETPAYHYGTHYSSAMIVASYLVRMEPFTQIFLRL
QGGHFDLADRMFHSVREAWLSASKHNMADVKELIPEFFYLPEFLLNSNNFDLGSKQNGTKLGDVILPPWAKGDPREFIRV
HREALECDYVSAHLHEWIDLIFGYKQQGPPAVEAVNVFHHLFYEGQVDIYNINDPLKETATIGFINNFGQIPKQLFKKPH
PPKRVRSKSNGEAPGGPVTLNSTLDKIFFHHLDNLRPSLTPVKELKEPVGQIVCTDKGILAVEQNKVLVPPAWNKTFAWG
YADLSCRLSNYESDKAVVVYECLSEWGQILCAICPNPKLVITGGFSTAICVWETGSSKEKAKSLTLKQALLGHTDAVTCL
TASSAYHIVVSGSRDRTCIIWDLNKLSFVTQLRGHRAPVSALCINELTGDIVSCAGTYIHVWSINGSPVASANTFTGRSQ
QILCCCVSEMNEWDTQNVIVTGHSDGVVRFWRIEFLQVPETPAPEPIEPPDVPDCCGEIEGRDAPDEDSSESEGEEASLS
QEPKPRSSNSQPSSAVHRPKPRTGASWSVDSSSDDSRRWSDTLSIDEKEGFVFVNYSEGQVRPGVPVQPHSQQPSGPSHG
PVPQPLQPSTMEARSYNKLKPGYRWERQLVFRSKLTMHTAFDRKDNAHPAEITALAISKDHSKILVGDGRGRVFSWSVSD
QPGRSTADHWVKDEQVDSCSGCTVRFSLTERRHHCRNCGQVFCQKCSRFQSEIKRLKISSPVRVCQNCYYNLQHERSSEE
NPRN
>XP_005165446.1 WD repeat and FYVE domain-containing protein 3 isoform X1 [Danio rerio]
MLAEHRENSRAGFGLKKPLKMNMVKRIMGRPRQEECSPQDNALGLMHLRRLFSELCHPPRHMTQKEQEEKLYMMLPVFN
R
VFGNAPPSTMNEKFSDLLQFTTQVSRLMVTEIRRRASNKSTEAASRAIVQFLEVNQSEETSRGWMLLTTINLLASSGQKT
VDCMTTMSVPSTLVKCLYLFFDLPHMPEVPAATQTELPLADRRALLQKVFVQILVKLCSFVSPAEELAQKDDLQLLFSAI
TSWCPPHNLPWRKSAGEVLTTISRHGLSVNVVKYIHEKECLSTCIQNMQQSDDLSPLEIVEMFAGLSCFLKDSSDVSQTL
LDDFRMCQGYTFLIDLMIRLEQAKEDESKDALKDLVNLVTALTTYGVSELKPAGLTTGAPFLLPGFVVPQPSGKGHTVRN
IQAFSVLQNAFLKAKSGRLARMILEAIANIYAADYANYFILEAQHTLSQFAERVTKLPEVQSKYFELLEFVVFGLNYVPC
KELFSVSVLLKSSTSYGCSITATRTLLKLGRHHPVFSDVFREVGLLEVLVNLLHKYAALLKDPTQTHSDQGDSKNNVAEE
QKQLAWLVMETLTVLLQGSNTNAGLFREFGGARCVHNIVKYRQCREHALMIIQQLVLSPSGDDDMGTLLGLMHSAPTAEL
QLKTDILRALLAVLRESHRTRTVFRKVGGFVYVTSLLLAMERSLCSPPVIGWEKVNQNKVFELLHTIFCTLTAAMRYEPA
NSHFFRTEIQYEKLADAVRLLGCFSDAKKVGPATVFPSNAQPFQRLLEDEIIPAENVCPTLKHCSKLFIYMYKMATDSFD
SRAEQVPPCLTHETSLPSPWGTPAITRKSRHGYSSSSNSASGKALTDLKLHLGNSSQHSSDPVVIHPGAVLAMLDLLPSV
SSDTQPEHALDLQLAVANILQLLVHSERNQQILCEAGLHSRLLQRCSCALGDEDHPLHPPLQRMFERLASQALEPMVLRE
FLRLGNPLNCGAWDKKLLKQYRVHKPSSLSYEAEMRNSMTLSMEGFGPDSVFTINEDNSQYRISRSLVRSAEGSTVPLTR
VKCLVSMTTPHDIRLHGSSVTPAFVELDTSLEGFGCLFLPSLAPHNAPSSNANTSGVSDGAVVSGMGNGERFFPPPSGLS

YSTWFCVERFSTPPQSHPVRLLTVVRRATSSEQHYVCLAIVLSAKDRSLIVSTKEELLQSYVDDFSEESSFYEILPCCAR
FRCADLITEGQWHHLLLVMSKGMLKNSMATLYIDGQMVSTVKLHYVHNSPGGSASANPPVVSTVYSYIGTPPAQRQLSTL
VWRLGPTHFLEEVLPAASVAAIYELGPNYVGSFQAVYLPCKDSKTEAVPPCPVGLVPEEKVSFSLFAHSVSALTVAKIRK
VYNKLDSKAIAKQLGVSSHESATPVKLVHNAVGHLNGSARTVGAAVIGYLGVRAFVTKPVATNLQYIGGAAAILGLVAMA
SDVEGLYAAVKALVCVVKSNPLASKEMERIKGYQLLAMLLKKKRPLLNSHILHLTFSLVGTVDSGHETSIIPNSTAFQDL
LCDFEVWLHAPYELHLSLFEHFIELLTESSEAAKNAKLLREFQLIPKLLLTLRDQSLSQPTITAIGNVLSLLLQGFPNTY
DLLRFGQFISSTLPTFAVCEKFVIMENTNEEKIDRGADEEFGGLLSSNLILLRNRLLDILLKQLFSCKEKGTVNAQACEE
LVRTLGFDWLLMFMEEHIHSSTVTAALLILVVLLSNQSILSRFKEGLSGGGWLDHTDSVLTNKIGTVLGFNVGRSAGGRS
TLREINRDACHFPGFPVLQTLLPKHTNVPEIYFMLMALFFQQPITELPESLQVHFDLDSIWTFIFGVPASSATVVGSLHN
MCTETAFLLLAMLRSMLNLPWQSEEEGSWLREYPITLMQFFRYLYHNIPDLAPMWHSPDFLCTLAATVFPFNIRPYSEMV
SDLDDEASSPTEEFKAFVTDTGMNRSQSEYCNVGNKTYLTNHPAKKYVFDFMRLLIMDNLCMTPASKQTPIIDLLLEASP
ERSTRTQQKEFQSYILDSVMEQLLAADVLMGEEASLPITTGGSYQVLVNNVFYFTQRVVDKLWQGMFNKESKLVVDFIVQ
LITQSKRRSQGLSLDSVYHCLNRTILYQLSRPHKTVPQQVTLLDSLRVLTVNRNLVLGPGNHDQEFVACLAHCLINLHTG
SSVEGFGLEAEARMTTWHVAVPSENESDSVQSQDVSEGRQLLLKAVNRVWTELMHSKRQMLEDIFKVSLPVNDRGHVDIG
TARPALEEPAAKSWQNHLVHEKKCISRGETVAPANQSKLSRVSSGFGLSKLTGSRRSKKESGMNKNNLSAQETFQWMFTH
IAVVRDLVAMQYKEHQERQQNALKYVMDEWAGIEYELLRERGLWGPPIGSHLDKFQLDMTEGPCRMRKKMVRNDMFYIH
Y
PYVPDPEPNASTQKPARYRRAISYDSKEYYMRLLSGNPGMYQHSIEQNTEGETTQQEPEHGEDTIARVKGLVKAPLKRSR
STADGADEESQDQLQELLESGAMDEEQKTDNTTLLRLLEEGEKIQHMYRCARVQGLDTSEGLLLFGKEHFYVIDGFTMTV
TREIRDIETLPPNMHEPIIPRGARQSKSQLKRTCSIFAYEDIREVHKRRYLLQPMAAEVFSGDGRNYLLAFQKGVRNKVY
QRFLAVVPSLADSSESVSGQRPNTSVEQGSGLLSTLVGEKSVTQRWERGEISNFQYLMHLNTLAGRSYNDLMQYPVFPWI
LADFDSEELDLNNPKTFRNLSKPMGAQTDDRLIQYKKRFKDWEDPNGETPAYHYGTHYSSAMIVASYLVRMEPFTQIFLR
LQGGHFDLADRMFHSVREAWLSASKHNMADVKELIPEFFYLPEFLLNSNNFDLGSKQNGTKLGDVILPPWAKGDPREFIR
VHREALECDYVSAHLHEWIDLIFGYKQQGPPAVEAVNVFHHLFYEGQVDIYNINDPLKETATIGFINNFGQIPKQLFKKP
HPPKRVRSKSNGEAPGGPVTLNSTLDKIFFHHLDNLRPSLTPVKELKEPVGQIVCTDKGILAVEQNKVLVPPAWNKTFAW
GYADLSCRLSNYESDKAVVVYECLSEWGQILCAICPNPKLVITGGFSTAICVWETGSSKEKAKSLTLKQALLGHTDAVTC
LTASSAYHIVVSGSRDRTCIIWDLNKLSFVTQLRGHRAPVSALCINELTGDIVSCAGTYIHVWSINGSPVASANTFTGRS
QQILCCCVSEMNEWDTQNVIVTGHSDGVVRFWRIEFLQVPETPAPEPIEPPDVPDCCGEIEGRDAPDEDSSESEGEEASL
SQEPKPRSSNSQPSSAVHRPKPRTGASWSVDSSSDDSRRWSDTLSIDEKEGFVFVNYSEGQVRPGVPVQPHSQQPSGPSH
GPVPQPLQPSTMEARSYNKLKPGYRWERQLVFRSKLTMHTAFDRKDNAHPAEITALAISKDHSKILVGDGRGRVFSWSVS
DQPGRSTADHWVKDEQVDSCSGCTVRFSLTERRHHCRNCGQVFCQKCSRFQSEIKRLKISSPVRVCQNCYYNLQHERSSE
ENPRN
>XP_017211769.1 WD repeat and FYVE domain-containing protein 3 isoform X3 [Danio rerio]

MLAEHRENSRAGFGLKKPLKMNMVKRIMGRPRQEECSPQDNALGLMHLRRLFSELCHPPRHMTQKEQEEKLYMMLPVFN
R
VFGNAPPSTMNEKFSDLLQFTTQVSRLMVTEIRRRASNKSTEAASRAIVQFLEVNQSEETSRGWMLLTTINLLASSGQKT
VDCMTTMSVPSTLVKCLYLFFDLPHMPEVPAATQTELPLADRRALLQKVFVQILVKLCSFVSPAEELAQKDDLQLLFSAI
TSWCPPHNLPWRKSAGEVLTTISRHGLSVNVVKYIHEKECLSTCIQNMQQSDDLSPLEIVEMFAGLSCFLKDSSDVSQTL
LDDFRMCQGYTFLIDLMIRLEQAKEDESKDALKDLVNLVTALTTYGVSELKPAGLTTGAPFLLPGFVVPQPSGKGHTVRN
IQAFSVLQNAFLKAKSGRLARMILEAIANIYAADYANYFILEAQHTLSQFAERVTKLPEVQSKYFELLEFVVFGLNYVPC
KELFSVSVLLKSSTSYGCSITATRTLLKLGRHHPVFSDVFREVGLLEVLVNLLHKYAALLKDPTQTHSDQGDSKNNVAEE
QKQLAWLVMETLTVLLQGSNTNAGLFREFGGARCVHNIVKYRQCREHALMIIQQLVLSPSGDDDMGTLLGLMHSAPTAEL
QLKTDILRALLAVLRESHRTRTVFRKVGGFVYVTSLLLAMERSLCSPPVIGWEKVNQNKVFELLHTIFCTLTAAMRYEPA
NSHFFRTEIQYEKLADAVRLLGCFSDAKKVGPATVFPSNAQPFQRLLEDEIIPAENVCPTLKHCSKLFIYMYKMATDSFD
SRAEQVPPCLTHETSLPSPWGTPAITRKSRHGYSSSSNSASGKALTDLKLHLGNSSQHSSDPVVIHPGAVLAMLDLLPSV
SSDTQPEHALDLQLAVANILQLLVHSERNQQILCEAGLHSRLLQRCSCALGDEDHPLHPPLQRMFERLASQALEPMVLRE
FLRLGNPLNCGAWDKKLLKQYRVHKPSSLSYEAEMRNSMTLSMEGFGPDSVFTINEDNSQYRISRSLVRSAEGSTVPLTR
VKCLVSMTTPHDIRLHGSSVTPAFVELDTSLEGFGCLFLPSLAPHNAPSSNANTSGVSDGAVVSGMGNGERFFPPPSGLS
YSTWFCVERFSTPPQSHPVRLLTVVRRATSSEQHYVCLAIVLSAKDRSLIVSTKEELLQSYVDDFSEESSFYEILPCCAR
FRCADLITEGQWHHLLLVMSKGMLKNSMATLYIDGQMVSTVKLHYVHNSPGGSASANPPVVSTVYSYIGTPPAQRQLSTL
VWRLGPTHFLEEVLPAASVAAIYELGPNYVGSFQAVYLPCKDSKTEAVPPCPVGLVPEEKVSFSLFAHSVSALTVAKIRK
VYNKLDSKAIAKQLGVSSHESATPVKLVHNAVGHLNGSARTVGAAVIGYLGVRAFVTKPVATNLQYIGGAAAILGLVAMA
SDVEGLYAAVKALVCVVKSNPLASKEMERIKGYQLLAMLLKKKRPLLNSHILHLTFSLVGTVDSGHETSIIPNSTAFQDL
LCDFEVWLHAPYELHLSLFEHFIELLTESSEAAKNAKLLREFQLIPKLLLTLRDQSLSQPTITAIGNVLSLLLQGFPNTY
DLLRFGQFISSTLPTFAVCEKFVIMENTNEEKIDRGADEEFGGLLSSNLILLRNRLLDILLKQLFSCKEKGTVNAQACEE
LVRTLGFDWLLMFMEEHIHSSTVTAALLILVVLLSNQSILSRFKEGLSGGGWLDHTDSVLTNKIGTVLGFNVGRSAGGRS
TLREINRDACHFPGFPVLQTLLPKHTNVPEIYFMLMALFFQQPITELPESLQFDLDSIWTFIFGVPASSATVVGSLHNMC
TETAFLLLAMLRSMLNLPWQSEEEGSWLREYPITLMQFFRYLYHNIPDLAPMWHSPDFLCTLAATVFPFNIRPYSEMVSD
LDDEASSPTEEFKAFVTDTGMNRSQSEYCNVGNKTYLTNHPAKKYVFDFMRLLIMDNLCMTPASKQTPIIDLLLEASPER
STRTQQKEFQSYILDSVMEQLLAADVLMGEEASLPITTGGSYQVLVNNVFYFTQRVVDKLWQGMFNKESKLVVDFIVQLI
TQSKRRSQGLSLDSVYHCLNRTILYQLSRPHKTVPQQVTLLDSLRVLTVNRNLVLGPGNHDQEFVACLAHCLINLHTGSS
VEGFGLEAEARMTTWHVAVPSENESDSVQSQDVSEGRQLLLKAVNRVWTELMHSKRQMLEDIFKVSLPVNDRGHVDIGTA
RPALEEPAAKSWQNHLVHEKKCISRGETVAPANQSKLSRVSSGFGLSKLTGSRRSKKESGMNKNNLSAQETFQWMFTHIA
VVRDLVAMQYKEHQERQQNALKYVMDEWAGIEYELLRERGLWGPPIGSHLDKFQLDMTEGPCRMRKKMVRNDMFYIHY
PY
VPDPEPNASTQKPARYRRAISYDSKEYYMRLLSGNPGMYQHSIEQNTEGETTQQEPEHGEDTIARVKGLVKAPLKRSRST

ADGADEESQDQLQELLESGAMDEEQKTDNTTLLRLLEEGEKIQHMYRCARVQGLDTSEGLLLFGKEHFYVIDGFTMTVTR
EIRDIETLPPNMHEPIIPRGARQSKSQLKRTCSIFAYEDIREVHKRRYLLQPMAAEVFSGDGRNYLLAFQKGVRNKVYQR
FLAVVPSLADSSESVSGQRPNTSVEQGSGLLSTLVGEKSVTQRWERGEISNFQYLMHLNTLAGRSYNDLMQYPVFPWILA
DFDSEELDLNNPKTFRNLSKPMGAQTDDRLIQYKKRFKDWEDPNGETPAYHYGTHYSSAMIVASYLVRMEPFTQIFLRLQ
GGHFDLADRMFHSVREAWLSASKHNMADVKELIPEFFYLPEFLLNSNNFDLGSKQNGTKLGDVILPPWAKGDPREFIRVH
REALECDYVSAHLHEWIDLIFGYKQQGPPAVEAVNVFHHLFYEGQVDIYNINDPLKETATIGFINNFGQIPKQLFKKPHP
PKRVRSKSNGEAPGGPVTLNSTLDKIFFHHLDNLRPSLTPVKELKEPVGQIVCTDKGILAVEQNKVLVPPAWNKTFAWGY
ADLSCRLSNYESDKAVVVYECLSEWGQILCAICPNPKLVITGGFSTAICVWETGSSKEKAKSLTLKQALLGHTDAVTCLT
ASSAYHIVVSGSRDRTCIIWDLNKLSFVTQLRGHRAPVSALCINELTGDIVSCAGTYIHVWSINGSPVASANTFTGRSQQ
ILCCCVSEMNEWDTQNVIVTGHSDGVVRFWRIEFLQVPETPAPEPIEPPDVPDCCGEIEGRDAPDEDSSESEGEEASLSQ
EPKPRSSNSQPSSAVHRPKPRTGASWSVDSSSDDSRRWSDTLSIDEKEGFVFVNYSEGQVRPGVPVQPHSQQPSGPSHGP
VPQPLQPSTMEARSYNKLKPGYRWERQLVFRSKLTMHTAFDRKDNAHPAEITALAISKDHSKILVGDGRGRVFSWSVSDQ
PGRSTADHWVKDEQVDSCSGCTVRFSLTERRHHCRNCGQVFCQKCSRFQSEIKRLKISSPVRVCQNCYYNLQHERSSEEN
PRN
>XP_001921741.4 WD repeat and FYVE domain-containing protein 3 isoform X4 [Danio rerio]
MNMVKRIMGRPRQEECSPQDNALGLMHLRRLFSELCHPPRHMTQKEQEEKLYMMLPVFNRVFGNAPPSTMNEKFSDLLQF
TTQVSRLMVTEIRRRASNKSTEAASRAIVQFLEVNQSEETSRGWMLLTTINLLASSGQKTVDCMTTMSVPSTLVKCLYLF
FDLPHMPEVPAATQTELPLADRRALLQKVFVQILVKLCSFVSPAEELAQKDDLQLLFSAITSWCPPHNLPWRKSAGEVLT
TISRHGLSVNVVKYIHEKECLSTCIQNMQQSDDLSPLEIVEMFAGLSCFLKDSSDVSQTLLDDFRMCQGYTFLIDLMIRL
EQAKEDESKDALKDLVNLVTALTTYGVSELKPAGLTTGAPFLLPGFVVPQPSGKGHTVRNIQAFSVLQNAFLKAKSGRLA
RMILEAIANIYAADYANYFILEAQHTLSQFAERVTKLPEVQSKYFELLEFVVFGLNYVPCKELFSVSVLLKSSTSYGCSI
TATRTLLKLGRHHPVFSDVFREVGLLEVLVNLLHKYAALLKDPTQTHSDQGDSKNNVAEEQKQLAWLVMETLTVLLQGSN
TNAGLFREFGGARCVHNIVKYRQCREHALMIIQQLVLSPSGDDDMGTLLGLMHSAPTAELQLKTDILRALLAVLRESHRT
RTVFRKVGGFVYVTSLLLAMERSLCSPPVIGWEKVNQNKVFELLHTIFCTLTAAMRYEPANSHFFRTEIQYEKLADAVRL
LGCFSDAKKVGPATVFPSNAQPFQRLLEDEIIPAENVCPTLKHCSKLFIYMYKMATDSFDSRAEQVPPCLTHETSLPSPW
GTPAITRKSRHGYSSSSNSASGKALTDLKLHLGNSSQHSSDPVVIHPGAVLAMLDLLPSVSSDTQPEHALDLQLAVANIL
QLLVHSERNQQILCEAGLHSRLLQRCSCALGDEDHPLHPPLQRMFERLASQALEPMVLREFLRLGNPLNCGAWDKKLLKQ
YRVHKPSSLSYEAEMRNSMTLSMEGFGPDSVFTINEDNSQYRISRSLVRSAEGSTVPLTRVKCLVSMTTPHDIRLHGSSV
TPAFVELDTSLEGFGCLFLPSLAPHNAPSSNANTSGVSDGAVVSGMGNGERFFPPPSGLSYSTWFCVERFSTPPQSHPVR
LLTVVRRATSSEQHYVCLAIVLSAKDRSLIVSTKEELLQSYVDDFSEESSFYEILPCCARFRCADLITEGQWHHLLLVMS
KGMLKNSMATLYIDGQMVSTVKLHYVHNSPGGSASANPPVVSTVYSYIGTPPAQRQLSTLVWRLGPTHFLEEVLPAASVA
AIYELGPNYVGSFQAVYLPCKDSKTEAVPPCPVGLVPEEKVSFSLFAHSVSALTVAKIRKVYNKLDSKAIAKQLGVSSHE
SATPVKLVHNAVGHLNGSARTVGAAVIGYLGVRAFVTKPVATNLQYIGGAAILGLVAMASDVEGLYAAVKALVCVVKSN

PLASKEMERIKGYQLLAMLLKKKRPLLNSHILHLTFSLVGTVDSGHETSIIPNSTAFQDLLCDFEVWLHAPYELHLSLFE
HFIELLTESSEAAKNAKLLREFQLIPKLLLTLRDQSLSQPTITAIGNVLSLLLQGFPNTYDLLRFGQFISSTLPTFAVCE
KFVIMENTNEEKIDRGADEEFGGLLSSNLILLRNRLLDILLKQLFSCKEKGTVNAQACEELVRTLGFDWLLMFMEEHIHS
STVTAALLILVVLLSNQSILSRFKEGLSGGGWLDHTDSVLTNKIGTVLGFNVGRSAGGRSTLREINRDACHFPGFPVLQT
LLPKHTNVPEIYFMLMALFFQQPITELPESLQVHFDLDSIWTFIFGVPASSATVVGSLHNMCTETAFLLLAMLRSMLNLP
WQSEEEGSWLREYPITLMQFFRYLYHNIPDLAPMWHSPDFLCTLAATVFPFNIRPYSEMVSDLDDEASSPTEEFKAFVTD
TGMNRSQSEYCNVGNKTYLTNHPAKKYVFDFMRLLIMDNLCMTPASKQTPIIDLLLEASPERSTRTQQKEFQSYILDSVM
EQLLAADVLMGEEASLPITTGGSYQVLVNNVFYFTQRVVDKLWQGMFNKESKLVVDFIVQLITQSKRRSQGLSLDSVYHC
LNRTILYQLSRPHKTVPQQVTLLDSLRVLTVNRNLVLGPGNHDQEFVACLAHCLINLHTGSSVEGFGLEAEARMTTWHVA
VPSENESDSVQSQDVSEGRQLLLKAVNRVWTELMHSKRQMLEDIFKVSLPVNDRGHVDIGTARPALEEPAAKSWQNHLVH
EKKCISRGETVAPANQSKLSRVSSGFGLSKLTGSRRSKKESGMNKNNLSAQETFQWMFTHIAVVRDLVAMQYKEHQERQQ
NALKYVMDEWAGIEYELLRERGLWGPPIGSHLDKFQLDMTEGPCRMRKKMVRNDMFYIHYPYVPDPEPNASTQKPARYRR
AISYDSKEYYMRLLSGNPGMYQHSIEQNTEGETTQQEPEHGEDTIARVKGLVKAPLKRSRSTADGADEESQDQLQELLES
GAMDEEQKTDNTTLLRLLEEGEKIQHMYRCARVQGLDTSEGLLLFGKEHFYVIDGFTMTVTREIRDIETLPPNMHEPIIP
RGARQSKSQLKRTCSIFAYEDIREVHKRRYLLQPMAAEVFSGDGRNYLLAFQKGVRNKVYQRFLAVVPSLADSSESVSGQ
RPNTSVEQGSGLLSTLVGEKSVTQRWERGEISNFQYLMHLNTLAGRSYNDLMQYPVFPWILADFDSEELDLNNPKTFRNL
SKPMGAQTDDRLIQYKKRFKDWEDPNGETPAYHYGTHYSSAMIVASYLVRMEPFTQIFLRLQGGHFDLADRMFHSVREAW
LSASKHNMADVKELIPEFFYLPEFLLNSNNFDLGSKQNGTKLGDVILPPWAKGDPREFIRVHREALECDYVSAHLHEWID
LIFGYKQQGPPAVEAVNVFHHLFYEGQVDIYNINDPLKETATIGFINNFGQIPKQLFKKPHPPKRVRSKSNGEAPGGPVT
LNSTLDKIFFHHLDNLRPSLTPVKELKEPVGQIVCTDKGILAVEQNKVLVPPAWNKTFAWGYADLSCRLSNYESDKAVVV
YECLSEWGQILCAICPNPKLVITGGFSTAICVWETGSSKEKAKSLTLKQALLGHTDAVTCLTASSAYHIVVSGSRDRTCI
IWDLNKLSFVTQLRGHRAPVSALCINELTGDIVSCAGTYIHVWSINGSPVASANTFTGRSQQILCCCVSEMNEWDTQNVI
VTGHSDGVVRFWRIEFLQVPETPAPEPIEPPDVPDCCGEIEGRDAPDEDSSESEGEEASLSQEPKPRSSNSQPSSAVHRP
KPRTGASWSVDSSSDDSRRWSDTLSIDEKEGFVFVNYSEGQVRPGVPVQPHSQQPSGPSHGPVPQPLQPSTMEARSYNKL
KPGYRWERQLVFRSKLTMHTAFDRKDNAHPAEITALAISKDHSKILVGDGRGRVFSWSVSDQPGRSTADHWVKDEQVDSC
SGCTVRFSLTERRHHCRNCGQVFCQKCSRFQSEIKRLKISSPVRVCQNCYYNLQHERSSEENPRN
>NP_001292489.1 zinc finger FYVE domain-containing protein 26 [Danio rerio]
MHPFGREEETSRRELFGFFRRCLQRGEWELAAACVSQLGEARAEDPHSPRDIVKAIVTHPYPLRWETVGSPHRLAWYWLQ
VLERPTEIKVSVSVKRELEFLLLLEELEDIPQSVLKELHEAFLYSEEVKGKAPGDSASPLSAALLSCLRSLLTRQQPRLC
HSLISFLSNGDPSRDHTLQDAFIQHLTEQVKVPAKDRNREWVEQVCSVLALAPWGSDRSGAQLEPLWEGLWAAREGLMTE
ERILGCLLRPQSQALLMAYSSTALRLLKDKLLAEAPHTQVDLPDLERVMLGLCCHKDKRLAWKAIYFECLSSGKHFLEQV
LLTGLDLIKREEFSKLETLLHSEFQPLSRLLLLLGWMHCQSLESAKTLLTVLHHRQPQSNDSALSDLACTLSCQLGVLEW
CTQNNPGISHDALLSHLHMLDHHSALYVLHCLTPLAKFEEHKVLELLQRTGDPSQVDSPSSSVQRNITLFRGFCAMKYAL

YAISVNARTHAHTGSLDCEPLHELQTEAAQEDETAEGCSNLFQHYLSECQLYLEAVPALFRLELLENIFSLLFLSDSDFT
QQTSTATETQPYTNTETSKPKSTKEVNTAEHFLKAENEGERMDELNQINPQLGHLTSGCRGFLVDLNVMEGILRLVREGL
EGVCGVGQEDGRALGADVELAESLGCSVTAETFSARLQRLSKYTAEAQWRLQIVTSNHGNGNDGLYLPSPLPSPALPLKR
QGSGSSGTSSRRRRKHHRHRSERHVSSERQNGEVSTSTSDGGVCVSVVCRGTETCPCGGSHSWLVPAMLSPPESLLIACI
RRGNYIEAQQVIAMYGLENSECAGELVFMDRYREVLIELAQVEQKIESQSLSTSSSSSEGLGTLGATPAGRTRMGSSSRL
TLKSIGSAAAAGMAFYSISDVADRLMSTPSRPIPCLEDSYWLSQCTIDSPDFVRPLVEELSPAAMAAFDLSCCQCQLWKT
SRQLLETAERRLSSFLEARGVRVDPKVPHANGIRGFPSVLQQISKILNKTSTNKGMSKTDCNGEENAVCSPFGCSAQEVL
LSCHSTLTEESITIQQNLNQRLEVTLQTLSSATNTSVDSLVGSNLLTALAEQAALKQSELDCHPVRTAMKQLLQYLDQLC
PLVPDGAPDRPDYIRSFFDYVNVLASVLVRSLGSEDQSTVVKLGNPLLLLLQSPAQLLSHLLFDRQVSPDRVLSLLQQER
LRLSVQQVVVQRCCETLPLWDSRAVNFSQRVSGLNGGAFCPASIASILQQYAQDCSPSLDLSDPTTTSDPSSESEVSVED
VSAASTSLSTSPPSPSSSSPSSSFLLTPSALSFLKSRSPLVAALACLSASRGGVTRVTSSGWSGLPSYFRGSGRKEAVLD
GEQISREGEALLKNFPILRMYLRTMAEPVLGVSLEADEGLGVALCGKPVVGMLFSGLQGNAAQAMAAEAFQQALNNGDLN
RALDLLELYAQPCSQEGALRDKLLAFTALQESSSAEQLFRVRDWELRGRVVLQGLDRWPLQQCLDLLHFCLSDQNTKDPL
REQLQQRKQELDMYQRMLRLQPSLPWETWQELREESTKNPESVFSQMLEAREFELCAQWVQLYSVSDQSGMQLQTEHLLY
LLEHNHTEEAFQLLEALSDSMGLEVSERALDRRPGLAACHFLSDYLTLHFQSQMTPARRRHIHALHLGSKVLLTLPEASR
QDYFSLLSDPLLMLEQMLMNLKVDWAAVAITTLRSLLLAQDAGITTQHIDTLLADYARKALDFPYAPREWSRSDSVISLQ
DAFLQCPAQESCPSSPSHTPPPSTGGTPMQTPSSERRPSGKKIRPLATPFTPPEKTPDRKDWIPDHKQHICMVCQRERFT
MFNRRHHCRRCGRLVCHSCSSKKMAVAGFDEPVRVCDQCYNFFHTDSDEELEQGEAGSPSSIDEVLNGVLSLPEVSRKQY
RLSPNPAENQQLKSEFYYEQAPSASLCVAILTLHSDHAACGQQLIDHCRSLSRKLTNPEVDARLLTDVMRQLLFSAKLMF
VNAGCTQEPALCDSYISKVDVLKILVTANYKYIPSLDDIQETAAVTRLRNQLLELEYYQLAVEVSTKSALDPNGVWQAWA
MASLKAGNLSGAREKFVRCLKAPVDRNQLNHGPRLLQEIIQHLESTVKLTLSQTMDEDILASLRELEEALSDSAPPEGTE
SKVQRCNFHQECLFYLFTYGTHLSLISFYLRHDCLKDALTYLQNKGCSDDVFLEGIFQPCLERGRLGALQGLLENLDPTL
ETWGRYLLSACQLLQRRGHYHSLYQLQQFMMDHIRAAMTCIRFFSHGAQSYLQLGEQQRWLIRAKEHLRTYLQEQQSRRK
SHSSSFRKKMSSTDVSRHIHTIELQLEVTRFLHRCESSPSKTAVGSTYTGNSAPPTLFGNSPMKVDVACKVMLGGKNIEE
GFGIAYRVIQDFQLEALAVYIRVGQRLVRQRQYSSVRQLHKCVGESGTASKHDCDAIVLSCVSVADKSPADAKELETLIL
ECKNPENKIKAYLQCSKLRAAYLQAVKLKLVKATPLIQEVMRAAESSGDSVMHDICRQWLSEHQEPSSKQR
>XP_003199781.2 zinc finger FYVE domain-containing protein 26 isoform X3 [Danio rerio]
MHPFGREEETSRRELFGFFRRCLQRGEWELAAACVSQLGEARAEDPHSPRDIVKAIVTHPYPLRWETVGSPHRLAWYWLQ
VLERPTEIKVSVSVKRELEFLLLLEELEDIPQSVLKELHEAFLYSEEVKGKAPGDSASPLSAALLSCLRSLLTRQQPRLC
HSLISFLSNGDPSRDHTLQDAFIQHLTEQVKVPAKDRNREWVEQVCSVLALAPWGSDRSGAQLEPLWEGLWAAREGLMTE
ERILGCLLRPQSQALLMAYSSTALRLLKDKLLAEAPHTQVDLPDLERVMLGLCCHKDKRLAWKAIYFECLSSGKHFLEQV
LLTGLDLIKREEFSKLETLLHSEFQPLSRLLLLLGWMHCQSLESAKTLLTVLHHRQPQSNDSALSDLACTLSCQLGVLEW
CTQNNPGISHDALLSHLHMLDHHSALYVLHCLTPLAKFEEHKVLELLQRTGDPSQVDSPSSSVQRNITLFRGFCAMKYAL

YAISVNARTHAHTGSLDCEPLHELQTEAAQEDETAEGCSNLFQHYLSECQLYLEAVPALFRLELLENIFSLLFLSDSDFT
QQTSTATETQPYTNTETSKPKSTKEVNTAEHFLKAENEGERMDELNQINPQLGHLTSGCRGFLVDLNVMEGILRLVREGL
EGVCGVGQEDGRALGADVELAESLGCSVTAETFSARLQRLSKYTAEAQWRLQIVTSNHGNGNDGLYLPSPLPSPALPLKR
QGSGSSGTSSRRRRKHHRHRSERHVSSERQNGEVSTSTSDGGVCVSVVCRGTETCPCGGSHSWLVPAMLSPPESLLIACI
RRGNYIEAQQVIAMYGLENSECAGELVFMDRYREVLIELAQVEQKIESQSLSTSSSSSEGLGTLGATPAGRTRMGSSSRL
TLKSIGSAAAAGMAFYSISDVADRLMSTPSRPIPCLEDSYWLSQCTIDSPDFVRPLVEELSPAAMAAFDLSCCQCQLWKT
SRQLLETAERRLSSFLEARGVRVDPKVPHANGIRGFPSVLQQISKILNKTSTNKGMSKTDCNGEENAVCSPFGCSAQEVL
LSCHSTLTEESITIQQNLNQRLEVTLQTLSSATNTSVDSLVGSNLLTALAEQAALKQSELDCHPVRTAMKQLLQYLDQLC
PLVPDGAPDRPDYIRSFFDYVNVLASVLVRSLGSEDQSTVVKLGNPLLLLLQSPAQLLSHLLFDRQVSPDRVLSLLQQER
LRLSVQQVVVQRCCETLPLWDSRAVNFSQRVSGLNGGAFCPASIASILQQYAQDCSPSLDLSDPTTTSDPSSESEVSVED
VSAASTSLSTSPPSPSSSSPSSSFLLTPSALSFLKSRSPLVAALACLSASRGGVTRVTSSGWSGLPSYFRGSGRKEAVLD
GEQISREGEALLKNFPILRMYLRTMAEPVLGVSLEADEGLGVALCGKPVVGMLFSGLQGNAAQAMAAEAFQQALNNGDLN
RALDLLELYAQPCSQEGALRDKLLAFTALQESSSAEQLFRVRDWELRGRVVLQGLDRWPLQQCLDLLHFCLSDQNTKDPL
REQLQQRKQELDMYQRMLRLQPSLPWETWQELREESTKNPESVFSQMLEAREFELCAQWVQLYSVSDQSGMQLQTEHLLY
LLEHNHTEEAFQLLEALSDSMGLEVSERALDRRPGLAACHFLSDYLTLHFQSQMTPARRRHIHALHLGSKVLLTLPEASR
QDYFSLLSDPLLMLEQMLMNLKVDWAAVAITTLRSLLLAQDAGITTQHIDTLLADYARKALDFPYAPREWSRSDSVISLQ
DAFLQCPAQESCPSSPSHTPPPSTGGTPMQTPSSERRPSGKKIRPLATPFTPPEKTPDRKDWIPDHKQHICMVCQRERFT
MFNRRHHCRRCGRLVCHSCSSKKMAVAGFDEPVRVCDQCYNFFHTDSDEELEQGEVAGSPSSIDEVLNGVLSLPEVSRKQ
YRLSPNPAENQQLKSEFYYEQAPSASLCVAILTLHSDHAACGQQLIDHCRSLSRKLTNPEVDARLLTDVMRQLLFSAKLM
FVNAGCTQEPALCDSYISKVDVLKILVTANYKYIPSLDDIQETAAVTRLRNQLLELEYYQLAVEVSTKSALDPNGVWQAW
AMASLKAGNLSGAREKFVRCLKAPVDRNQLNHGPRLLQEIIQHLESTVKLTLSQTMDEDILASLRELEEALSDSAPPEGT
ESKVQRCNFHQECLFYLFTYGTHLSLISFYLRHDCLKDALTYLQNKGCSDDVFLEGIFQPCLERGRLGALQGLLENLDPT
LETWGRYLLSACQLLQRRGHYHSLYQLQQFMMDHIRAAMTCIRFFSHGAQSYLQLGEQQRWLIRAKEHLRTYLQEQQSRR
KSHSSSFRKKMSSTDVSRHIHTIELQLEVTRFLHRCESSPSKTAVGSTYTGNSAPPTLFGNSPMKVDVACKVMLGGKNIE
EGFGIAYRVIQDFQLEALAVYIRVGQRLVRQRQYSSVRQLHKCVGESGTASKHDCDAIVLSCVSVADKSPADAKELETLI
LECKNPENKIKAYLQCSKLRAAYLQAVKLKLVKATPLIQEVMRAAESSGDSVMHDICRQWLSEHQEPSSKQR
>XP_021336195.1 zinc finger FYVE domain-containing protein 26 isoform X2 [Danio rerio]
MHPFGREEETSRRELFGFFRRCLQRGEWELAAACVSQLGEARAEDPHSPRDIVKAIVTHPYPLRWETVGSPHRLAWYWLQ
VLERPTEIKVSVSVKRELEFLLLLEELEDIPQSVLKELHEAFLYSEEVKGKAPGDSASPLSAALLSCLRSLLTRQQPRLC
HSLISFLSNGDPSRDHTLQDAFIQHLTEQVKVPAKDRNREWVEQVCSVLALAPWGSDRSGAQLEPLWEGLWAAREGLMTE
ERILGCLLRPQSQALLMAYSSTALRLLKDKLLAEAPHTQVDLPDLERVMLGLCCHKDKRLAWKAIYFECLSSGKHFLEQV
LLTGLDLIKREEFSKLETLLHSEFQPLSRLLLLLGWMHCQSLESAKTLLTVLHHRQPQSNDSALSDLACTLSCQLGVLEW
CTQNNPGISHDALLSHLHMLDHHSALYVLHCLTPLAKFEEHKVLELLQRTGDPSQVDSPSSSVQRNITLFRGFCAMKYAL

YAISVNARTHAHTGSLDCEPLHELQTEAAQEDETAEGCSNLFQHYLSECQLYLEAVPALFRLELLENIFSLLFLSDSDFT
QQTSTATETQPYTNTETSKPKSTKEVNTAEHFLKAENEGERMDELNQINPQLGHLTSGCRGFLVDLNVMEGILRLVREGL
EGVCGVGQEDGRALGADVELAESLGCSVTAETFSARLQRLSKYTAEAQWRLQIVTSNHGNGNDGLYLPSPLPSPALPLKR
QGSGSSGTSSRRRRKHHRHRSERHVSSERQNGEVSTSTSDGGVCVSVVCRGTETCPCGGSHSWLVPAMLSPPESLLIACI
RRGNYIEAQQVIAMYGLENSECAGELVFMDRYREVLIELAQVEQKIESQSLSTSSSSSEGLGTLGATPAGRTRMGSSSRL
TLKSIGSAAAAGMAFYSISDVADRLMSTPSRPIPCLEDSYWLSQCTIDSPDFVRPLVEELSPAAMAAFDLSCCQCQLWKT
SRQLLETAERRLSSFLEARGVRVDPKVPHANGIRGFPSVLQQISKILNKTSTNKGMSKTDCNGEENAVCSPFGCSAQEVL
LSCHSTLTEESITIQQNLNQRLEVTLQTLSSATNTSVDSLVGSNLLTALAEQAALKQSELDCHPVRTAMKQLLQYLDQLC
PLVPDGAPDRPDYIRSFFDYVNVLASVLVRSLGSEDQSTVVKLGNPLLLLLQSPAQLLSHLLFDRQVSPDRVLSLLQQER
LRLSVQQVVVQRCCETLPLWDSRAVNFSQRVSGLNGGAFCPASIASILQQYAQDCSPSLDLSDPTTTSDPSSESEVSVED
VSAASTSLSTSPPSPSSSSPSSSFLLTPSALSFLKSRSPLVAALACLSASRGGVTRVTSSGWSGLPSYFRGSGRKEAVLD
GEQISREGEALLKNFPILRMYLRTMAEPVLGVSLEADEGLGVALCGKPVVGMLFSGLQGNAAQAMAAEAFQQALNNGDLN
RALDLLELYAQPCSQEGALRDKLLAFTALQESSSAEQLFRVRDWELRGRVVLQGLDRWPLQQCLDLLHFCLSDQNTKDPL
REQLQQRKQELDMYQRMLRLQPSLPWETWQELREESTKNPESVFSQMLEAREFELCAQWVQLYSVSDQSGMQLQTEHLLY
LLEHNHTEEAFQLLEALSDSMGLEVSERALDRRPGLAACHFLSDYLTLHFQSQMTPARRRHIHALHLGSKVLLTLPEASR
QDYFSLLSDPLLMLEQMLMNLKVDWAAVAITTLRSLLLAQDAGITTQHIDTLLADYARKALDFPYAPREWSRSDSVISLQ
DAFLQCPAQESCPSSPSHTPPPSTGGTPMQTPSSERRPSGKKIRPLATPFTPPEKTPDRKDWIPDHKQHICMVCQRERFT
MFNRRHHCRRCGRLVCHSCSSKKMAVAGFDEPVRVCDQCYNFFHTDSDEELEQGEAGSPSSIDEVLNGVLSLPEVSRKQY
RLSPNPAENQQLKSEFYYEQAPSASLCVAILTLHSDHAACGQQLIDHCRSLSRKLTNPEVDARLLTDVMRQLLFSAKLMF
VNAGCTQEPALCDSYISKVDVLKILVTANYKYIPSLDDIQETAAVTRLRNQLLELEYYQLAVEVSTKSALDPNGVWQAWA
MASLKAGNLSGAREKFVRCLKAPVDRNQLNHGPRLLQEIIQHLESTVKLTLSQTMDEDILASLRELEEALSDSAPPEGTE
SKVQRCNFHQECLFYLFTYGTHLSLISFYLRHDCLKDALTYLQNKGCSDDVFLEGIFQPCLERGRLGALQGLLENLDPTL
ETWGRYLLSACQLLQRRGHYHSLYQLQQFMMDHIRAAMTCIRFFSHGAQSYLQLGEQQRWLIRAKEHLRTYLQEQQSRRK
SHSSSFRKKMSSTDVSRHIHTIELQLEVTRFLHRCESSPSKTAVGSTYTGNSAPPTLFGNSPMKVDVACKCLTVMLGGKN
IEEGFGIAYRVIQDFQLEALAVYIRVGQRLVRQRQYSSVRQLHKCVGESGTASKHDCDAIVLSCVSVADKSPADAKELET
LILECKNPENKIKAYLQCSKLRAAYLQAVKLKLVKATPLIQEVMRAAESSGDSVMHDICRQWLSEHQEPSSKQR
>XP_021336194.1 zinc finger FYVE domain-containing protein 26 isoform X1 [Danio rerio]
MHPFGREEETSRRELFGFFRRCLQRGEWELAAACVSQLGEARAEDPHSPRDIVKAIVTHPYPLRWETVGSPHRLAWYWLQ
VLERPTEIKVSVSVKRELEFLLLLEELEDIPQSVLKELHEAFLYSEEVKGKAPGDSASPLSAALLSCLRSLLTRQQPRLC
HSLISFLSNGDPSRDHTLQDAFIQHLTEQVKVPAKDRNREWVEQVCSVLALAPWGSDRSGAQLEPLWEGLWAAREGLMTE
ERILGCLLRPQSQALLMAYSSTALRLLKDKLLAEAPHTQVDLPDLERVMLGLCCHKDKRLAWKAIYFECLSSGKHFLEQV
LLTGLDLIKREEFSKLETLLHSEFQPLSRLLLLLGWMHCQSLESAKTLLTVLHHRQPQSNDSALSDLACTLSCQLGVLEW
CTQNNPGISHDALLSHLHMLDHHSALYVLHCLTPLAKFEEHKVLELLQRTGDPSQVDSPSSSVQRNITLFRGFCAMKYAL

YAISVNARTHAHTGSLDCEPLHELQTEAAQEDETAEGCSNLFQHYLSECQLYLEAVPALFRLELLENIFSLLFLSDSDFT
QQTSTATETQPYTNTETSKPKSTKEVNTAEHFLKAENEGERMDELNQINPQLGHLTSGCRGFLVDLNVMEGILRLVREGL
EGVCGVGQEDGRALGADVELAESLGCSVTAETFSARLQRLSKYTAEAQWRLQIVTSNHGNGNDGLYLPSPLPSPALPLKR
QGSGSSGTSSRRRRKHHRHRSERHVSSERQNGEVSTSTSDGGVCVSVVCRGTETCPCGGSHSWLVPAMLSPPESLLIACI
RRGNYIEAQQVIAMYGLENSECAGELVFMDRYREVLIELAQVEQKIESQSLSTSSSSSEGLGTLGATPAGRTRMGSSSRL
TLKSIGSAAAAGMAFYSISDVADRLMSTPSRPIPCLEDSYWLSQCTIDSPDFVRPLVEELSPAAMAAFDLSCCQCQLWKT
SRQLLETAERRLSSFLEARGVRVDPKVPHANGIRGFPSVLQQISKILNKTSTNKGMSKTDCNGEENAVCSPFGCSAQEVL
LSCHSTLTEESITIQQNLNQRLEVTLQTLSSATNTSVDSLVGSNLLTALAEQAALKQSELDCHPVRTAMKQLLQYLDQLC
PLVPDGAPDRPDYIRSFFDYVNVLASVLVRSLGSEDQSTVVKLGNPLLLLLQSPAQLLSHLLFDRQVSPDRVLSLLQQER
LRLSVQQVVVQRCCETLPLWDSRAVNFSQRVSGLNGGAFCPASIASILQQYAQDCSPSLDLSDPTTTSDPSSESEVSVED
VSAASTSLSTSPPSPSSSSPSSSFLLTPSALSFLKSRSPLVAALACLSASRGGVTRVTSSGWSGLPSYFRGSGRKEAVLD
GEQISREGEALLKNFPILRMYLRTMAEPVLGVSLEADEGLGVALCGKPVVGMLFSGLQGNAAQAMAAEAFQQALNNGDLN
RALDLLELYAQPCSQEGALRDKLLAFTALQESSSAEQLFRVRDWELRGRVVLQGLDRWPLQQCLDLLHFCLSDQNTKDPL
REQLQQRKQELDMYQRMLRLQPSLPWETWQELREESTKNPESVFSQMLEAREFELCAQWVQLYSVSDQSGMQLQTEHLLY
LLEHNHTEEAFQLLEALSDSMGLEVSERALDRRPGLAACHFLSDYLTLHFQSQMTPARRRHIHALHLGSKVLLTLPEASR
QDYFSLLSDPLLMLEQMLMNLKVDWAAVAITTLRSLLLAQDAGITTQHIDTLLADYARKALDFPYAPREWSRSDSVISLQ
DAFLQCPAQESCPSSPSHTPPPSTGGTPMQTPSSERRPSGKKIRPLATPFTPPEKTPDRKDWIPDHKQHICMVCQRERFT
MFNRRHHCRRCGRLVCHSCSSKKMAVAGFDEPVRVCDQCYNFFHTDSDEELEQGEVAGSPSSIDEVLNGVLSLPEVSRKQ
YRLSPNPAENQQLKSEFYYEQAPSASLCVAILTLHSDHAACGQQLIDHCRSLSRKLTNPEVDARLLTDVMRQLLFSAKLM
FVNAGCTQEPALCDSYISKVDVLKILVTANYKYIPSLDDIQETAAVTRLRNQLLELEYYQLAVEVSTKSALDPNGVWQAW
AMASLKAGNLSGAREKFVRCLKAPVDRNQLNHGPRLLQEIIQHLESTVKLTLSQTMDEDILASLRELEEALSDSAPPEGT
ESKVQRCNFHQECLFYLFTYGTHLSLISFYLRHDCLKDALTYLQNKGCSDDVFLEGIFQPCLERGRLGALQGLLENLDPT
LETWGRYLLSACQLLQRRGHYHSLYQLQQFMMDHIRAAMTCIRFFSHGAQSYLQLGEQQRWLIRAKEHLRTYLQEQQSRR
KSHSSSFRKKMSSTDVSRHIHTIELQLEVTRFLHRCESSPSKTAVGSTYTGNSAPPTLFGNSPMKVDVACKCLTVMLGGK
NIEEGFGIAYRVIQDFQLEALAVYIRVGQRLVRQRQYSSVRQLHKCVGESGTASKHDCDAIVLSCVSVADKSPADAKELE
TLILECKNPENKIKAYLQCSKLRAAYLQAVKLKLVKATPLIQEVMRAAESSGDSVMHDICRQWLSEHQEPSSKQR
>XP_005167727.1 1-phosphatidylinositol 3-phosphate 5-kinase isoform X10 [Danio rerio]
MAADDKASSSSSVMDWSSEPPPSPTSPSHLTHFKPLTPEQDEPPLRSAYSSFVSLFRFNKEEGRPPSVTEKSQSASSSPQ
GPRRNWSSPSHSIHGSETHRKHPELLRRTSAASEGRRKSEAPLGSHDPRTAVQLRTALKRLKEIMEGKSQDSDLKQYWMP
DSQCKECYDCNEKFTTFRRRHHCRLCGQIFCSRCCNQEIPGKFMGYTGDLRACTYCRKIALSYAHSTDSSSIGEDLSALS
DSPCSVCVLEPTEPRTPVGGRKASRNIFLEEDLAWQSHQESQNSGLNSRLTVVQEDIGKSPARKRSASVTNLSLDRSGSS
MVPAYESSVSPQNSRALPKTDHSEEERKILLDSFQLKDIWKKICHNTTGMEFQDHRYWLRTYPNCIVGKELVNWLLRNGT
ISTRAQAIAIGQALVDGRWLDCVTHHDQIFRDEYALYRPLQSTEFSETPSPDSDSVNSLEGHSEPSWFKDIKFDDSDTEQ

LADENDYVTPSSSSPSKRTSVSSFHSAVDSDSAASISLNMEQDNVNFHIKKQAKYPHVPPYPAEQKMEVLLSEDGGQHIS
ISDAFIKESLFNRRVEEKAKEMLFTPLGWHHSSLDQLREENGEKKAMERLLSANHSHMMALLQQLLYSESLSLSWRDIIV
PVVRQVVQTVRPDVRSCDDDMDIRQFVHIKKIPGGKKFDSAVVNGFVCTKNIAHKKMNSYIKNPKILLLKCSIEYLYREE
TKFTCIDPIVLQEREFLKNYVQRIVDVRPNLVLVEKTVSRIAQDMLLEHGIAIVINVKPQVLDRVSRMTQGDLVISMDQL
LTKPRLGTCHKFYLHSFQLPNSEVKTLMFFEGCPPQLGCTIKLRGASEYELARVKEIIIFMVCVAYHSQLEISFLMDEFA
MPPSLAESSSFPCLLESTTLREEDETSGERESQDNDESTLGDDNLTLVPGDDFETGFTGTIKAHSREPSTSESSHKDGES
PRINKNASITSFSGGEEENIKTSTPLSSFSSTFPQPVTPPFLISDVKEMSQEVVKGSGEGEKNKYLESTLVHQDSTSSET
SLPPTRLFRDPLQDDTGLFVTEHVASSDDRLKSISALFKQELKDIILCISPFITFREPFLLTPAGLRCPSRDYFPEQVYL
SPLLNKDSKELDGRRKRQLLKESSPGSGSLTNGSVSHQRAIQILPCHKLTSARIAEQVGSSQQLARMLADYRAQGGRIRQ
REGTQFREAPPPKAPIKADSEEDKGAGVIDMTWSTKLDCLNPINHQRLCVLFSSSSAQSNNAPNPCVSPWIVTMEFYGKN
DLTLGVFLERYCFRPSYQCPSMYCETPMVHHIRRFVHGKGCVQIVLKELDSPVPGYQHTILNYSWCRICKQVTPVVPLSN
DSWSMSFAKYLELRFYGHQYTRRANAEPCGHSIHKDYHQYFSYNQMVASFSYIPVRLLEICLPPPKIIIRNQGPSKSSLQ
QDLKDFSQKVAQVYLAIDDRLTSLKTDTFSKTREEKMEDMFAQKDMEESELRGWIEKLQVRLQTSVIDSSQQLQAVLESV
VVKKQGLCETLQSWNNRLQELFQQEKGRKRLSVPPSPGRHRQPTTDESKTSALESSPRNPSPVVPNGDKEDRHLNTFPSS
SGSSSLLQLPSPAEQPSDVITSGPSFPDQDSVSNPEDMFDGHLLGSNDSQVKEKSTMKTILANLLPGNSYNPIPFPFDPD
KHYLMYEHERVPIAVCEREPSSIIAFALSCKEYKTALEELTKTTAKTGGDDTSQTISAGESRVKNSPAKPSDNSMSQLSR
SSVDADPLKEPESADKQKKQTGNPHIELQFSDANAKFYCRIYYAEEFHKMREEIMESTEDDFVRSLSHCVNWQARGGKSG
AVFYATEDDRFILKQMPRLEVQSFLDFAPHYFTYITGAVHLKRPTALAKILGVYRIGYKNSQNNTEKKLDLLVMENLFYG
RKMAQVFDLKGSLRNRNVKTDQGKESCEVVLLDENLLKLVHDNPLYIRSHCKAILRAAIHSDALFLSSHLIIDYSLLVGR
DDSTDELVVGIIDYIRTFTWDKKLEMVVKSTGILGGQGKMPTVVSPELYRTRFCEAMDKYFLMVPDHWSGLGLNC
>XP_005167726.1 1-phosphatidylinositol 3-phosphate 5-kinase isoform X9 [Danio rerio]
MAADDKASSSSSVMDWSSEPPPSPTSPSHLTHFKPLTPEQDEPPLRSAYSSFVSLFRFNKEEGRPPSVTEKSQSASSSPQ
GPRRNWSSPSHSIHGSETHRKHPELLRRTSAASEGRRKSEAPLGSHDPRTAVQLRTALKRLKEIMEGKSQDSDLKQYWMP
DSQCKECYDCNEKFTTFRRRHHCRLCGQIFCSRCCNQEIPGKFMGYTGDLRACTYCRKIALSYAHSTDSSSIGEDLSALS
DSPCSVCVLEPTEPRTPVGGRKASRNIFLEEDLAWQRKNSIGMRKNHQESQNSGLNSRLTVVQEDIGKSPARKRSASVTN
LSLDRSGSSMVPAYESSVSPQNSRALPKTDHSEEERKILLDSFQLKDIWKKICHNTTGMEFQDHRYWLRTYPNCIVGKEL
VNWLLRNGTISTRAQAIAIGQALVDGRWLDCVTHHDQIFRDEYALYRPLQSTEFSETPSPDSDSVNSLEGHSEPSWFKDI
KFDDSDTEQLADENDYVTPSSSSPSKRTSVSSFHSAVDSDSAASISLNMEQDNVNFHIKKQAKYPHVPPYPAEQKMEVLL
SEDGGQHISISDAFIKESLFNRRVEEKAKEMLFTPLGWHHSSLDQLREENGEKKAMERLLSANHSHMMALLQQLLYSESL
SLSWRDIIVPVVRQVVQTVRPDVRSCDDDMDIRQFVHIKKIPGGKKFDSAVVNGFVCTKNIAHKKMNSYIKNPKILLLKC
SIEYLYREETKFTCIDPIVLQEREFLKNYVQRIVDVRPNLVLVEKTVSRIAQDMLLEHGIAIVINVKPQVLDRVSRMTQG
DLVISMDQLLTKPRLGTCHKFYLHSFQLPNSEVKTLMFFEGCPPQLGCTIKLRGASEYELARVKEIIIFMVCVAYHSQLE
ISFLMDEFAMPPSLAESSSFPCLLESTTLREEDETSGERESQDNDESTLGDDNLTLVPGDDFETGFTGTIKAHSREPSTS

ESSHKDGESPRINKNASITSFSGGEEENIKTSTPLSSFSSTFPQPVTPPFLISDVKEMSQEVVKGSGEGEKNKYLESTLV
HQDSTSSETSLPPTRLFRDPLQDDTGLFVTEHVASSDDRLKSISALFKQELKDIILCISPFITFREPFLLTPAGLRCPSR
DYFPEQVYLSPLLNKDSKELDGRRKRQLLKESSPGSGSLTNGSVSHQRAIQILPCHKLTSARIAEQVGSSQQLARMLADY
RAQGGRIRQREGTQFREAPPPKAPIKADSEEDKGAGVIDMTWSTKLDCLNPINHQRLCVLFSSSSAQSNNAPNPCVSPWI
VTMEFYGKNDLTLGVFLERYCFRPSYQCPSMYCETPMVHHIRRFVHGKGCVQIVLKELDSPVPGYQHTILNYSWCRICKQ
VTPVVPLSNDSWSMSFAKYLELRFYGHQYTRRANAEPCGHSIHKDYHQYFSYNQMVASFSYIPVRLLEICLPPPKIIIRN
QGPSKSSLQQDLKDFSQKVAQVYLAIDDRLTSLKTDTFSKTREEKMEDMFAQKDMEESELRGWIEKLQVRLQTSVIDSSQ
QLQAVLESVVVKKQGLCETLQSWNNRLQELFQQEKGRKRLSVPPSPGRHRQPTTDESKTSALESSPRNPSPVVPNGDKED
RHLNTFPSSSGSSSLLQLPSPAEQPSDVITSGPSFPDQDSVSNPEDMFDGHLLGSNDSQVKEKSTMKTILANLLPGNSYN
PIPFPFDPDKHYLMYEHERVPIAVCEREPSSIIAFALSCKEYKTALEELTKTTAKTGGDDTSQTISAGESRVKNSPAKPS
DNSMSQLSRSSVDADPLKEPESADKQKKQTGNPHIELQFSDANAKFYCRIYYAEEFHKMREEIMESTEDDFVRSLSHCVN
WQARGGKSGAVFYATEDDRFILKQMPRLEVQSFLDFAPHYFTYITGAVHLKRPTALAKILGVYRIGYKNSQNNTEKKLDL
LVMENLFYGRKMAQVFDLKGSLRNRNVKTDQGKESCEVVLLDENLLKLVHDNPLYIRSHCKAILRAAIHSDALFLSSHLI
IDYSLLVGRDDSTDELVVGIIDYIRTFTWDKKLEMVVKSTGILGGQGKMPTVVSPELYRTRFCEAMDKYFLMVPDHWSGL
GLNC
>XP_005167724.1 1-phosphatidylinositol 3-phosphate 5-kinase isoform X8 [Danio rerio]
MAADDKASSSSSVMDWSSEPPPSPTSPSHLTHFKPLTPEQDEPPLRSAYSSFVSLFRFNKEKTGANIAPAKKLAKLEEGR
PPSVTEKSQSASSSPQGPRRNWSSPSHSIHGSETHRKHPELLRRTSAASEGRRKSEAPLGSHDPRTAVQLRTALKRLKEI
MEGKSQDSDLKQYWMPDSQCKECYDCNEKFTTFRRRHHCRLCGQIFCSRCCNQEIPGKFMGYTGDLRACTYCRKIALSYA
HSTDSSSIGEDLSALSDSPCSVCVLEPTEPRTPVGGRKASRNIFLEEDLAWQSHQESQNSGLNSRLTVVQEDIGKSPARK
RSASVTNLSLDRSGSSMVPAYESSVSPQNSRALPKTDHSEEERKILLDSFQLKDIWKKICHNTTGMEFQDHRYWLRTYPN
CIVGKELVNWLLRNGTISTRAQAIAIGQALVDGRWLDCVTHHDQIFRDEYALYRPLQSTEFSETPSPDSDSVNSLEGHSE
PSWFKDIKFDDSDTEQLADENDYVTPSSSSPSKRTSVSSFHSAVDSDSAASISLNMEQDNVNFHIKKQAKYPHVPPYPAE
QKMEVLLSEDGGQHISISDAFIKESLFNRRVEEKAKEMLFTPLGWHHSSLDQLREENGEKKAMERLLSANHSHMMALLQQ
LLYSESLSLSWRDIIVPVVRQVVQTVRPDVRSCDDDMDIRQFVHIKKIPGGKKFDSAVVNGFVCTKNIAHKKMNSYIKNP
KILLLKCSIEYLYREETKFTCIDPIVLQEREFLKNYVQRIVDVRPNLVLVEKTVSRIAQDMLLEHGIAIVINVKPQVLDR
VSRMTQGDLVISMDQLLTKPRLGTCHKFYLHSFQLPNSEVKTLMFFEGCPPQLGCTIKLRGASEYELARVKEIIIFMVCV
AYHSQLEISFLMDEFAMPPSLAESSSFPCLLESTTLREEDETSGERESQDNDESTLGDDNLTLVPGDDFETGFTGTIKAH
SREPSTSESSHKDGESPRINKNASITSFSGGEEENIKTSTPLSSFSSTFPQPVTPPFLISDVKEMSQEVVKGSGEGEKNK
YLESTLVHQDSTSSETSLPPTRLFRDPLQDDTGLFVTEHVASSDDRLKSISALFKQELKDIILCISPFITFREPFLLTPA
GLRCPSRDYFPEQVYLSPLLNKDSKELDGRRKRQLLKESSPGSGSLTNGSVSHQRAIQILPCHKLTSARIAEQVGSSQQL
ARMLADYRAQGGRIRQREGTQFREAPPPKAPIKADSEEDKGAGVIDMTWSTKLDCLNPINHQRLCVLFSSSSAQSNNAPN
PCVSPWIVTMEFYGKNDLTLGVFLERYCFRPSYQCPSMYCETPMVHHIRRFVHGKGCVQIVLKELDSPVPGYQHTILNYS

WCRICKQVTPVVPLSNDSWSMSFAKYLELRFYGHQYTRRANAEPCGHSIHKDYHQYFSYNQMVASFSYIPVRLLEICLPP
PKIIIRNQGPSKSSLQQDLKDFSQKVAQVYLAIDDRLTSLKTDTFSKTREEKMEDMFAQKDMEESELRGWIEKLQVRLQT
SVIDSSQQLQAVLESVVVKKQGLCETLQSWNNRLQELFQQEKGRKRLSVPPSPGRHRQPTTDESKTSALESSPRNPSPVV
PNGDKEDRHLNTFPSSSGSSSLLQLPSPAEQPSDVITSGPSFPDQDSVSNPEDMFDGHLLGSNDSQVKEKSTMKTILANL
LPGNSYNPIPFPFDPDKHYLMYEHERVPIAVCEREPSSIIAFALSCKEYKTALEELTKTTAKTGGDDTSQTISAGESRVK
NSPAKPSDNSMSQLSRSSVDADPLKEPESADKQKKQTGNPHIELQFSDANAKFYCRIYYAEEFHKMREEIMESTEDDFVR
SLSHCVNWQARGGKSGAVFYATEDDRFILKQMPRLEVQSFLDFAPHYFTYITGAVHLKRPTALAKILGVYRIGYKNSQNN
TEKKLDLLVMENLFYGRKMAQVFDLKGSLRNRNVKTDQGKESCEVVLLDENLLKLVHDNPLYIRSHCKAILRAAIHSDAL
FLSSHLIIDYSLLVGRDDSTDELVVGIIDYIRTFTWDKKLEMVVKSTGILGGQGKMPTVVSPELYRTRFCEAMDKYFLMV
PDHWSGLGLNC
>XP_009303000.1 1-phosphatidylinositol 3-phosphate 5-kinase isoform X7 [Danio rerio]
MAADDKASSSSSVMDWSSEPPPSPTSPSHLTHFKPLTPEQDEPPLRSAYSSFVSLFRFNKEKTGANIAPAKKLAKLEEGR
PPSVTEKSQSASSSPQGPRRNWSSPSHSIHGSETHRKHPELLRRTSAASAEWEEGRRKSEAPLGSHDPRTAVQLRTALKR
LKEIMEGKSQDSDLKQYWMPDSQCKECYDCNEKFTTFRRRHHCRLCGQIFCSRCCNQEIPGKFMGYTGDLRACTYCRKIA
LSYAHSTDSSSIGEDLSALSDSPCSVCVLEPTEPRTPVGGRKASRNIFLEEDLAWQSHQESQNSGLNSRLTVVQEDIGKS
PARKRSASVTNLSLDRSGSSMVPAYESSVSPQNSRALPKTDHSEEERKILLDSFQLKDIWKKICHNTTGMEFQDHRYWLR
TYPNCIVGKELVNWLLRNGTISTRAQAIAIGQALVDGRWLDCVTHHDQIFRDEYALYRPLQSTEFSETPSPDSDSVNSLE
GHSEPSWFKDIKFDDSDTEQLADENDYVTPSSSSPSKRTSVSSFHSAVDSDSAASISLNMEQDNVNFHIKKQAKYPHVPP
YPAEQKMEVLLSEDGGQHISISDAFIKESLFNRRVEEKAKEMLFTPLGWHHSSLDQLREENGEKKAMERLLSANHSHMMA
LLQQLLYSESLSLSWRDIIVPVVRQVVQTVRPDVRSCDDDMDIRQFVHIKKIPGGKKFDSAVVNGFVCTKNIAHKKMNSY
IKNPKILLLKCSIEYLYREETKFTCIDPIVLQEREFLKNYVQRIVDVRPNLVLVEKTVSRIAQDMLLEHGIAIVINVKPQ
VLDRVSRMTQGDLVISMDQLLTKPRLGTCHKFYLHSFQLPNSEVKTLMFFEGCPPQLGCTIKLRGASEYELARVKEIIIF
MVCVAYHSQLEISFLMDEFAMPPSLAESSSFPCLLESTTLREEDETSGERESQDNDESTLGDDNLTLVPGDDFETGFTGT
IKAHSREPSTSESSHKDGESPRINKNASITSFSGGEEENIKTSTPLSSFSSTFPQPVTPPFLISDVKEMSQEVVKGSGEG
EKNKYLESTLVHQDSTSSETSLPPTRLFRDPLQDDTGLFVTEHVASSDDRLKSISALFKQELKDIILCISPFITFREPFL
LTPAGLRCPSRDYFPEQVYLSPLLNKDSKELDGRRKRQLLKESSPGSGSLTNGSVSHQRAIQILPCHKLTSARIAEQVGS
SQQLARMLADYRAQGGRIRQREGTQFREAPPPKAPIKADSEEDKGAGVIDMTWSTKLDCLNPINHQRLCVLFSSSSAQSN
NAPNPCVSPWIVTMEFYGKNDLTLGVFLERYCFRPSYQCPSMYCETPMVHHIRRFVHGKGCVQIVLKELDSPVPGYQHTI
LNYSWCRICKQVTPVVPLSNDSWSMSFAKYLELRFYGHQYTRRANAEPCGHSIHKDYHQYFSYNQMVASFSYIPVRLLEI
CLPPPKIIIRNQGPSKSSLQQDLKDFSQKVAQVYLAIDDRLTSLKTDTFSKTREEKMEDMFAQKDMEESELRGWIEKLQV
RLQTSVIDSSQQLQAVLESVVVKKQGLCETLQSWNNRLQELFQQEKGRKRLSVPPSPGRHRQPTTDESKTSALESSPRNP
SPVVPNGDKEDRHLNTFPSSSGSSSLLQLPSPAEQPSDVITSGPSFPDQDSVSNPEDMFDGHLLGSNDSQVKEKSTMKTI
LANLLPGNSYNPIPFPFDPDKHYLMYEHERVPIAVCEREPSSIIAFALSCKEYKTALEELTKTTAKTGGDDTSQTISAGE

SRVKNSPAKPSDNSMSQLSRSSVDADPLKEPESADKQKKQTGNPHIELQFSDANAKFYCRIYYAEEFHKMREEIMESTED
DFVRSLSHCVNWQARGGKSGAVFYATEDDRFILKQMPRLEVQSFLDFAPHYFTYITGAVHLKRPTALAKILGVYRIGYKN
SQNNTEKKLDLLVMENLFYGRKMAQVFDLKGSLRNRNVKTDQGKESCEVVLLDENLLKLVHDNPLYIRSHCKAILRAAIH
SDALFLSSHLIIDYSLLVGRDDSTDELVVGIIDYIRTFTWDKKLEMVVKSTGILGGQGKMPTVVSPELYRTRFCEAMDKY
FLMVPDHWSGLGLNC
>NP_001120777.1 1-phosphatidylinositol 3-phosphate 5-kinase [Danio rerio]
MAADDKASSSSSVMDWSSEPPPSPTSPSHLTHFKPLTPEQDEPPLRSAYSSFVSLFRFNKEKTGANIAPAKKLAKLEEGR
PPSVTEKSQSASSSPQGPRRNWSSPSHSIHGSETHRKHPELLRRTSAASEGRRKSEAPLGSHDPRTAVQLRTALKRLKEI
MEGKSQDSDLKQYWMPDSQCKECYDCNEKFTTFRRRHHCRLCGQIFCSRCCNQEIPGKFMGYTGDLRACTYCRKIALSYA
HSTDSSSIGEDLSALSDSPCSVCVLEPTEPRTPVGGRKASRNIFLEEDLAWQRKNSIGMRKNHQESQNSGLNSRLTVVQE
DIGKSPARKRSASVTNLSLDRSGSSMVPAYESSVSPQNSRALPKTDHSEEERKILLDSFQLKDIWKKICHNTTGMEFQDH
RYWLRTYPNCIVGKELVNWLLRNGTISTRAQAIAIGQALVDGRWLDCVTHHDQIFRDEYALYRPLQSTEFSETPSPDSDS
VNSLEGHSEPSWFKDIKFDDSDTEQLADENDYVTPSSSSPSKRTSVSSFHSAVDSDSAASISLNMEQDNVNFHIKKQAKY
PHVPPYPAEQKMEVLLSEDGGQHISISDAFIKESLFNRRVEEKAKEMLFTPLGWHHSSLDQLREENGEKKAMERLLSANH
SHMMALLQQLLYSESLSLSWRDIIVPVVRQVVQTVRPDVRSCDDDMDIRQFVHIKKIPGGKKFDSAVVNGFVCTKNIAHK
KMNSYIKNPKILLLKCSIEYLYREETKFTCIDPIVLQEREFLKNYVQRIVDVRPNLVLVEKTVSRIAQDMLLEHGIAIVI
NVKPQVLDRVSRMTQGDLVISMDQLLTKPRLGTCHKFYLHSFQLPNSEVKTLMFFEGCPPQLGCTIKLRGASEYELARVK
EIIIFMVCVAYHSQLEISFLMDEFAMPPSLAESSSFPCLLESTTLREEDETSGERESQDNDESTLGDDNLTLVPGDDFET
GFTGTIKAHSREPSTSESSHKDGESPRINKNASITSFSGGEEENIKTSTPLSSFSSTFPQPVTPPFLISDVKEMSQEVVK
GSGEGEKNKYLESTLVHQDSTSSETSLPPTRLFRDPLQDDTGLFVTEHVASSDDRLKSISALFKQELKDIILCISPFITF
REPFLLTPAGLRCPSRDYFPEQVYLSPLLNKDSKELDGRRKRQLLKESSPGSGSLTNGSVSHQRAIQILPCHKLTSARIA
EQVGSSQQLARMLADYRAQGGRIRQREGTQFREAPPPKAPIKADSEEDKGAGVIDMTWSTKLDCLNPINHQRLCVLFSSS
SAQSNNAPNPCVSPWIVTMEFYGKNDLTLGVFLERYCFRPSYQCPSMYCETPMVHHIRRFVHGKGCVQIVLKELDSPVPG
YQHTILNYSWCRICKQVTPVVPLSNDSWSMSFAKYLELRFYGHQYTRRANAEPCGHSIHKDYHQYFSYNQMVASFSYIPV
RLLEICLPPPKIIIRNQGPSKSSLQQDLKDFSQKVAQVYLAIDDRLTSLKTDTFSKTREEKMEDMFAQKDMEESELRGWI
EKLQVRLQTSVIDSSQQLQAVLESVVVKKQGLCETLQSWNNRLQELFQQEKGRKRLSVPPSPGRHRQPTTDESKTSALES
SPRNPSPVVPNGDKEDRHLNTFPSSSGSSSLLQLPSPAEQPSDVITSGPSFPDQDSVSNPEDMFDGHLLGSNDSQVKEKS
TMKTILANLLPGNSYNPIPFPFDPDKHYLMYEHERVPIAVCEREPSSIIAFALSCKEYKTALEELTKTTAKTGGDDTSQT
ISAGESRVKNSPAKPSDNSMSQLSRSSVDADPLKEPESADKQKKQTGNPHIELQFSDANAKFYCRIYYAEEFHKMREEIM
ESTEDDFVRSLSHCVNWQARGGKSGAVFYATEDDRFILKQMPRLEVQSFLDFAPHYFTYITGAVHLKRPTALAKILGVYR
IGYKNSQNNTEKKLDLLVMENLFYGRKMAQVFDLKGSLRNRNVKTDQGKESCEVVLLDENLLKLVHDNPLYIRSHCKAIL
RAAIHSDALFLSSHLIIDYSLLVGRDDSTDELVVGIIDYIRTFTWDKKLEMVVKSTGILGGQGKMPTVVSPELYRTRFCE
AMDKYFLMVPDHWSGLGLNC

>XP_005167722.1 1-phosphatidylinositol 3-phosphate 5-kinase isoform X5 [Danio rerio]
MAADDKASSSSSVMDWSSEPPPSPTSPSHLTHFKPLTPEQDEPPLRSAYSSFVSLFRFNKEKTGANIAPAKKLAKLEEGR
PPSVTEKSQSASSSPQGPRRNWSSPSHSIHGSETHRKHPELLRRTSAASAEWEEGRRKSEAPLGSHDPRTAVQLRTALKR
LKEIMEGKSQDSDLKQYWMPDSQCKECYDCNEKFTTFRRRHHCRLCGQIFCSRCCNQEIPGKFMGYTGDLRACTYCRKIA
LSYAHSTDSSSIGEDLSALSDSPCSVCVLEPTEPRTPVGGRKASRNIFLEEDLAWQRKNSIGMRKNHQESQNSGLNSRLT
VVQEDIGKSPARKRSASVTNLSLDRSGSSMVPAYESSVSPQNSRALPKTDHSEEERKILLDSFQLKDIWKKICHNTTGME
FQDHRYWLRTYPNCIVGKELVNWLLRNGTISTRAQAIAIGQALVDGRWLDCVTHHDQIFRDEYALYRPLQSTEFSETPSP
DSDSVNSLEGHSEPSWFKDIKFDDSDTEQLADENDYVTPSSSSPSKRTSVSSFHSAVDSDSAASISLNMEQDNVNFHIKK
QAKYPHVPPYPAEQKMEVLLSEDGGQHISISDAFIKESLFNRRVEEKAKEMLFTPLGWHHSSLDQLREENGEKKAMERLL
SANHSHMMALLQQLLYSESLSLSWRDIIVPVVRQVVQTVRPDVRSCDDDMDIRQFVHIKKIPGGKKFDSAVVNGFVCTKN
IAHKKMNSYIKNPKILLLKCSIEYLYREETKFTCIDPIVLQEREFLKNYVQRIVDVRPNLVLVEKTVSRIAQDMLLEHGI
AIVINVKPQVLDRVSRMTQGDLVISMDQLLTKPRLGTCHKFYLHSFQLPNSEVKTLMFFEGCPPQLGCTIKLRGASEYEL
ARVKEIIIFMVCVAYHSQLEISFLMDEFAMPPSLAESSSFPCLLESTTLREEDETSGERESQDNDESTLGDDNLTLVPGD
DFETGFTGTIKAHSREPSTSESSHKDGESPRINKNASITSFSGGEEENIKTSTPLSSFSSTFPQPVTPPFLISDVKEMSQ
EVVKGSGEGEKNKYLESTLVHQDSTSSETSLPPTRLFRDPLQDDTGLFVTEHVASSDDRLKSISALFKQELKDIILCISP
FITFREPFLLTPAGLRCPSRDYFPEQVYLSPLLNKDSKELDGRRKRQLLKESSPGSGSLTNGSVSHQRAIQILPCHKLTS
ARIAEQVGSSQQLARMLADYRAQGGRIRQREGTQFREAPPPKAPIKADSEEDKGAGVIDMTWSTKLDCLNPINHQRLCVL
FSSSSAQSNNAPNPCVSPWIVTMEFYGKNDLTLGVFLERYCFRPSYQCPSMYCETPMVHHIRRFVHGKGCVQIVLKELDS
PVPGYQHTILNYSWCRICKQVTPVVPLSNDSWSMSFAKYLELRFYGHQYTRRANAEPCGHSIHKDYHQYFSYNQMVASFS
YIPVRLLEICLPPPKIIIRNQGPSKSSLQQDLKDFSQKVAQVYLAIDDRLTSLKTDTFSKTREEKMEDMFAQKDMEESEL
RGWIEKLQVRLQTSVIDSSQQLQAVLESVVVKKQGLCETLQSWNNRLQELFQQEKGRKRLSVPPSPGRHRQPTTDESKTS
ALESSPRNPSPVVPNGDKEDRHLNTFPSSSGSSSLLQLPSPAEQPSDVITSGPSFPDQDSVSNPEDMFDGHLLGSNDSQV
KEKSTMKTILANLLPGNSYNPIPFPFDPDKHYLMYEHERVPIAVCEREPSSIIAFALSCKEYKTALEELTKTTAKTGGDD
TSQTISAGESRVKNSPAKPSDNSMSQLSRSSVDADPLKEPESADKQKKQTGNPHIELQFSDANAKFYCRIYYAEEFHKMR
EEIMESTEDDFVRSLSHCVNWQARGGKSGAVFYATEDDRFILKQMPRLEVQSFLDFAPHYFTYITGAVHLKRPTALAKIL
GVYRIGYKNSQNNTEKKLDLLVMENLFYGRKMAQVFDLKGSLRNRNVKTDQGKESCEVVLLDENLLKLVHDNPLYIRSHC
KAILRAAIHSDALFLSSHLIIDYSLLVGRDDSTDELVVGIIDYIRTFTWDKKLEMVVKSTGILGGQGKMPTVVSPELYRT
RFCEAMDKYFLMVPDHWSGLGLNC
>XP_009302997.1 1-phosphatidylinositol 3-phosphate 5-kinase isoform X2 [Danio rerio]
MAADDKASSSSSVMDWSSEPPPSPTSPSHLTHFKPLTPEQDEPPLRSAYSSFVSLFRFNKEKTGANIAPAKKLAKLEEGR
PPSVTEKSQSASSSPQGPRRNWSSPSHSIHGSETHRKHPELLRRTSAASEGRRKSEAPLGSHDPRTAVQLRTALKRLKEI
MEGKSQDSDLKQYWMPDSQCKECYDCNEKFTTFRRRHHCRLCGQIFCSRCCNQEIPGKFMGYTGDLRACTYCRKIALSYA
HSTDSSSIGEDLSALSDSPCSVCVLEPTEPRTPVGGRKASRNIFLEEDLAWQRKNSIGMRKNHQESQNSGLNSRLTVVQE

DIGKSPARKRSASVTNLSLDRSGSSMVPAYESSVSPQNSRALPKTDHSEEERKILLDSFQLKDIWKKICHNTTGMEFQDH
RYWLRTYPNCIVGKELVNWLLRNGTISTRAQAIAIGQALVDGRWLDCVTHHDQIFRDEYALYRPLQSTEFSETPSPDSDS
VNSLEGHSEPSWFKDIKFDDSDTEQLADENDYVTPSSSSPSKRTSVSSFHSAVDSDSAASISLNMEQDNVNFHIKKQAKY
PHVPPYPAEQKSMEPHDPFTPETDIHAPMEVLLSEDGGQHISISDAFIKESLFNRRVEEKAKEMLFTPLGWHHSSLDQLR
EENGEKKAMERLLSANHSHMMALLQQLLYSESLSLSWRDIIVPVVRQVVQTVRPDVRSCDDDMDIRQFVHIKKIPGGKKF
DSAVVNGFVCTKNIAHKKMNSYIKNPKILLLKCSIEYLYREETKFTCIDPIVLQEREFLKNYVQRIVDVRPNLVLVEKTV
SRIAQDMLLEHGIAIVINVKPQVLDRVSRMTQGDLVISMDQLLTKPRLGTCHKFYLHSFQLPNSEVKTLMFFEGCPPQLG
CTIKLRGASEYELARVKEIIIFMVCVAYHSQLEISFLMDEFAMPPSLAESSSFPCLLESTTLREEDETSGERESQDNDES
TLGDDNLTLVPGDDFETGFTGTIKAHSREPSTSESSHKDGESPRINKNASITSFSGGEEENIKTSTPLSSFSSTFPQPVT
PPFLISDVKEMSQEVVKGSGEGEKNKYLESTLVHQDSTSSETSLPPTRLFRDPLQDDTGLFVTEHVASSDDRLKSISALF
KQELKDIILCISPFITFREPFLLTPAGLRCPSRDYFPEQVYLSPLLNKDSKELDGRRKRQLLKESSPGSGSLTNGSVSHQ
RAIQILPCHKLTSARIAEQVGSSQQLARMLADYRAQGGRIRQREGTQFREAPPPKAPIKADSEEDKGAGVIDMTWSTKLD
CLNPINHQRLCVLFSSSSAQSNNAPNPCVSPWIVTMEFYGKNDLTLGVFLERYCFRPSYQCPSMYCETPMVHHIRRFVHG
KGCVQIVLKELDSPVPGYQHTILNYSWCRICKQVTPVVPLSNDSWSMSFAKYLELRFYGHQYTRRANAEPCGHSIHKDYH
QYFSYNQMVASFSYIPVRLLEICLPPPKIIIRNQGPSKSSLQQDLKDFSQKVAQVYLAIDDRLTSLKTDTFSKTREEKME
DMFAQKDMEESELRGWIEKLQVRLQTSVIDSSQQLQAVLESVVVKKQGLCETLQSWNNRLQELFQQEKGRKRLSVPPSPG
RHRQPTTDESKTSALESSPRNPSPVVPNGDKEDRHLNTFPSSSGSSSLLQLPSPAEQPSDVITSGPSFPDQDSVSNPEDM
FDGHLLGSNDSQVKEKSTMKTILANLLPGNSYNPIPFPFDPDKHYLMYEHERVPIAVCEREPSSIIAFALSCKEYKTALE
ELTKTTAKTGGDDTSQTISAGESRVKNSPAKPSDNSMSQLSRSSVDADPLKEPESADKQKKQTGNPHIELQFSDANAKFY
CRIYYAEEFHKMREEIMESTEDDFVRSLSHCVNWQARGGKSGAVFYATEDDRFILKQMPRLEVQSFLDFAPHYFTYITGA
VHLKRPTALAKILGVYRIGYKNSQNNTEKKLDLLVMENLFYGRKMAQVFDLKGSLRNRNVKTDQGKESCEVVLLDENLLK
LVHDNPLYIRSHCKAILRAAIHSDALFLSSHLIIDYSLLVGRDDSTDELVVGIIDYIRTFTWDKKLEMVVKSTGILGGQG
KMPTVVSPELYRTRFCEAMDKYFLMVPDHWSGLGLNC
>XP_009302999.1 1-phosphatidylinositol 3-phosphate 5-kinase isoform X4 [Danio rerio]
MAADDKASSSSSVMDWSSEPPPSPTSPSHLTHFKPLTPEQDEPPLRSAYSSFVSLFRFNKEEGRPPSVTEKSQSASSSPQ
GPRRNWSSPSHSIHGSETHRKHPELLRRTSAASAEWEEGRRKSEAPLGSHDPRTAVQLRTALKRLKEIMEGKSQDSDLKQ
YWMPDSQCKECYDCNEKFTTFRRRHHCRLCGQIFCSRCCNQEIPGKFMGYTGDLRACTYCRKIALSYAHSTDSSSIGEDL
SALSDSPCSVCVLEPTEPRTPVGGRKASRNIFLEEDLAWQRKNSIGMRKNHQESQNSGLNSRLTVVQEDIGKSPARKRSA
SVTNLSLDRSGSSMVPAYESSVSPQNSRALPKTDHSEEERKILLDSFQLKDIWKKICHNTTGMEFQDHRYWLRTYPNCIV
GKELVNWLLRNGTISTRAQAIAIGQALVDGRWLDCVTHHDQIFRDEYALYRPLQSTEFSETPSPDSDSVNSLEGHSEPSW
FKDIKFDDSDTEQLADENDYVTPSSSSPSKRTSVSSFHSAVDSDSAASISLNMEQDNVNFHIKKQAKYPHVPPYPAEQKS
MEPHDPFTPETDIHAPMEVLLSEDGGQHISISDAFIKESLFNRRVEEKAKEMLFTPLGWHHSSLDQLREENGEKKAMERL
LSANHSHMMALLQQLLYSESLSLSWRDIIVPVVRQVVQTVRPDVRSCDDDMDIRQFVHIKKIPGGKKFDSAVVNGFVCTK

NIAHKKMNSYIKNPKILLLKCSIEYLYREETKFTCIDPIVLQEREFLKNYVQRIVDVRPNLVLVEKTVSRIAQDMLLEHG
IAIVINVKPQVLDRVSRMTQGDLVISMDQLLTKPRLGTCHKFYLHSFQLPNSEVKTLMFFEGCPPQLGCTIKLRGASEYE
LARVKEIIIFMVCVAYHSQLEISFLMDEFAMPPSLAESSSFPCLLESTTLREEDETSGERESQDNDESTLGDDNLTLVPG
DDFETGFTGTIKAHSREPSTSESSHKDGESPRINKNASITSFSGGEEENIKTSTPLSSFSSTFPQPVTPPFLISDVKEMS
QEVVKGSGEGEKNKYLESTLVHQDSTSSETSLPPTRLFRDPLQDDTGLFVTEHVASSDDRLKSISALFKQELKDIILCIS
PFITFREPFLLTPAGLRCPSRDYFPEQVYLSPLLNKDSKELDGRRKRQLLKESSPGSGSLTNGSVSHQRAIQILPCHKLT
SARIAEQVGSSQQLARMLADYRAQGGRIRQREGTQFREAPPPKAPIKADSEEDKGAGVIDMTWSTKLDCLNPINHQRLCV
LFSSSSAQSNNAPNPCVSPWIVTMEFYGKNDLTLGVFLERYCFRPSYQCPSMYCETPMVHHIRRFVHGKGCVQIVLKELD
SPVPGYQHTILNYSWCRICKQVTPVVPLSNDSWSMSFAKYLELRFYGHQYTRRANAEPCGHSIHKDYHQYFSYNQMVASF
SYIPVRLLEICLPPPKIIIRNQGPSKSSLQQDLKDFSQKVAQVYLAIDDRLTSLKTDTFSKTREEKMEDMFAQKDMEESE
LRGWIEKLQVRLQTSVIDSSQQLQAVLESVVVKKQGLCETLQSWNNRLQELFQQEKGRKRLSVPPSPGRHRQPTTDESKT
SALESSPRNPSPVVPNGDKEDRHLNTFPSSSGSSSLLQLPSPAEQPSDVITSGPSFPDQDSVSNPEDMFDGHLLGSNDSQ
VKEKSTMKTILANLLPGNSYNPIPFPFDPDKHYLMYEHERVPIAVCEREPSSIIAFALSCKEYKTALEELTKTTAKTGGD
DTSQTISAGESRVKNSPAKPSDNSMSQLSRSSVDADPLKEPESADKQKKQTGNPHIELQFSDANAKFYCRIYYAEEFHKM
REEIMESTEDDFVRSLSHCVNWQARGGKSGAVFYATEDDRFILKQMPRLEVQSFLDFAPHYFTYITGAVHLKRPTALAKI
LGVYRIGYKNSQNNTEKKLDLLVMENLFYGRKMAQVFDLKGSLRNRNVKTDQGKESCEVVLLDENLLKLVHDNPLYIRSH
CKAILRAAIHSDALFLSSHLIIDYSLLVGRDDSTDELVVGIIDYIRTFTWDKKLEMVVKSTGILGGQGKMPTVVSPELYR
TRFCEAMDKYFLMVPDHWSGLGLNC
>XP_009302996.1 1-phosphatidylinositol 3-phosphate 5-kinase isoform X1 [Danio rerio]
MAADDKASSSSSVMDWSSEPPPSPTSPSHLTHFKPLTPEQDEPPLRSAYSSFVSLFRFNKEKTGANIAPAKKLAKLEEGR
PPSVTEKSQSASSSPQGPRRNWSSPSHSIHGSETHRKHPELLRRTSAASAEWEEGRRKSEAPLGSHDPRTAVQLRTALKR
LKEIMEGKSQDSDLKQYWMPDSQCKECYDCNEKFTTFRRRHHCRLCGQIFCSRCCNQEIPGKFMGYTGDLRACTYCRKIA
LSYAHSTDSSSIGEDLSALSDSPCSVCVLEPTEPRTPVGGRKASRNIFLEEDLAWQRKNSIGMRKNHQESQNSGLNSRLT
VVQEDIGKSPARKRSASVTNLSLDRSGSSMVPAYESSVSPQNSRALPKTDHSEEERKILLDSFQLKDIWKKICHNTTGME
FQDHRYWLRTYPNCIVGKELVNWLLRNGTISTRAQAIAIGQALVDGRWLDCVTHHDQIFRDEYALYRPLQSTEFSETPSP
DSDSVNSLEGHSEPSWFKDIKFDDSDTEQLADENDYVTPSSSSPSKRTSVSSFHSAVDSDSAASISLNMEQDNVNFHIKK
QAKYPHVPPYPAEQKSMEPHDPFTPETDIHAPMEVLLSEDGGQHISISDAFIKESLFNRRVEEKAKEMLFTPLGWHHSSL
DQLREENGEKKAMERLLSANHSHMMALLQQLLYSESLSLSWRDIIVPVVRQVVQTVRPDVRSCDDDMDIRQFVHIKKIPG
GKKFDSAVVNGFVCTKNIAHKKMNSYIKNPKILLLKCSIEYLYREETKFTCIDPIVLQEREFLKNYVQRIVDVRPNLVLV
EKTVSRIAQDMLLEHGIAIVINVKPQVLDRVSRMTQGDLVISMDQLLTKPRLGTCHKFYLHSFQLPNSEVKTLMFFEGCP
PQLGCTIKLRGASEYELARVKEIIIFMVCVAYHSQLEISFLMDEFAMPPSLAESSSFPCLLESTTLREEDETSGERESQD
NDESTLGDDNLTLVPGDDFETGFTGTIKAHSREPSTSESSHKDGESPRINKNASITSFSGGEEENIKTSTPLSSFSSTFP
QPVTPPFLISDVKEMSQEVVKGSGEGEKNKYLESTLVHQDSTSSETSLPPTRLFRDPLQDDTGLFVTEHVASSDDRLKSI

SALFKQELKDIILCISPFITFREPFLLTPAGLRCPSRDYFPEQVYLSPLLNKDSKELDGRRKRQLLKESSPGSGSLTNGS
VSHQRAIQILPCHKLTSARIAEQVGSSQQLARMLADYRAQGGRIRQREGTQFREAPPPKAPIKADSEEDKGAGVIDMTWS
TKLDCLNPINHQRLCVLFSSSSAQSNNAPNPCVSPWIVTMEFYGKNDLTLGVFLERYCFRPSYQCPSMYCETPMVHHIRR
FVHGKGCVQIVLKELDSPVPGYQHTILNYSWCRICKQVTPVVPLSNDSWSMSFAKYLELRFYGHQYTRRANAEPCGHSIH
KDYHQYFSYNQMVASFSYIPVRLLEICLPPPKIIIRNQGPSKSSLQQDLKDFSQKVAQVYLAIDDRLTSLKTDTFSKTRE
EKMEDMFAQKDMEESELRGWIEKLQVRLQTSVIDSSQQLQAVLESVVVKKQGLCETLQSWNNRLQELFQQEKGRKRLSVP
PSPGRHRQPTTDESKTSALESSPRNPSPVVPNGDKEDRHLNTFPSSSGSSSLLQLPSPAEQPSDVITSGPSFPDQDSVSN
PEDMFDGHLLGSNDSQVKEKSTMKTILANLLPGNSYNPIPFPFDPDKHYLMYEHERVPIAVCEREPSSIIAFALSCKEYK
TALEELTKTTAKTGGDDTSQTISAGESRVKNSPAKPSDNSMSQLSRSSVDADPLKEPESADKQKKQTGNPHIELQFSDAN
AKFYCRIYYAEEFHKMREEIMESTEDDFVRSLSHCVNWQARGGKSGAVFYATEDDRFILKQMPRLEVQSFLDFAPHYFTY
ITGAVHLKRPTALAKILGVYRIGYKNSQNNTEKKLDLLVMENLFYGRKMAQVFDLKGSLRNRNVKTDQGKESCEVVLLDE
NLLKLVHDNPLYIRSHCKAILRAAIHSDALFLSSHLIIDYSLLVGRDDSTDELVVGIIDYIRTFTWDKKLEMVVKSTGIL
GGQGKMPTVVSPELYRTRFCEAMDKYFLMVPDHWSGLGLNC
>XP_009302998.1 1-phosphatidylinositol 3-phosphate 5-kinase isoform X3 [Danio rerio]
MAADDKASSSSSVMDWSSEPPPSPTSPSHLTHFKPLTPEQDEPPLRSAYSSFVSLFRFNKEKTGANIAPAKKLAKLEEGR
PPSVTEKSQSASSSPQGPRRNWSSPSHSIHGSETHRKHPELLRRTSAASAEWEEGRRKSEAPLGSHDPRTAVQLRTALKR
LKEIMEGKSQDSDLKQYWMPDSQCKECYDCNEKFTTFRRRHHCRLCGQIFCSRCCNQEIPGKFMGYTGDLRACTYCRKIA
LSYAHSTDSSSIGEDLSALSDSPCSVCVLEPTEPRTPVGGRKASRNIFLEEDLAWQSHQESQNSGLNSRLTVVQEDIGKS
PARKRSASVTNLSLDRSGSSMVPAYESSVSPQNSRALPKTDHSEEERKILLDSFQLKDIWKKICHNTTGMEFQDHRYWLR
TYPNCIVGKELVNWLLRNGTISTRAQAIAIGQALVDGRWLDCVTHHDQIFRDEYALYRPLQSTEFSETPSPDSDSVNSLE
GHSEPSWFKDIKFDDSDTEQLADENDYVTPSSSSPSKRTSVSSFHSAVDSDSAASISLNMEQDNVNFHIKKQAKYPHVPP
YPAEQKSMEPHDPFTPETDIHAPMEVLLSEDGGQHISISDAFIKESLFNRRVEEKAKEMLFTPLGWHHSSLDQLREENGE
KKAMERLLSANHSHMMALLQQLLYSESLSLSWRDIIVPVVRQVVQTVRPDVRSCDDDMDIRQFVHIKKIPGGKKFDSAVV
NGFVCTKNIAHKKMNSYIKNPKILLLKCSIEYLYREETKFTCIDPIVLQEREFLKNYVQRIVDVRPNLVLVEKTVSRIAQ
DMLLEHGIAIVINVKPQVLDRVSRMTQGDLVISMDQLLTKPRLGTCHKFYLHSFQLPNSEVKTLMFFEGCPPQLGCTIKL
RGASEYELARVKEIIIFMVCVAYHSQLEISFLMDEFAMPPSLAESSSFPCLLESTTLREEDETSGERESQDNDESTLGDD
NLTLVPGDDFETGFTGTIKAHSREPSTSESSHKDGESPRINKNASITSFSGGEEENIKTSTPLSSFSSTFPQPVTPPFLI
SDVKEMSQEVVKGSGEGEKNKYLESTLVHQDSTSSETSLPPTRLFRDPLQDDTGLFVTEHVASSDDRLKSISALFKQELK
DIILCISPFITFREPFLLTPAGLRCPSRDYFPEQVYLSPLLNKDSKELDGRRKRQLLKESSPGSGSLTNGSVSHQRAIQI
LPCHKLTSARIAEQVGSSQQLARMLADYRAQGGRIRQREGTQFREAPPPKAPIKADSEEDKGAGVIDMTWSTKLDCLNPI
NHQRLCVLFSSSSAQSNNAPNPCVSPWIVTMEFYGKNDLTLGVFLERYCFRPSYQCPSMYCETPMVHHIRRFVHGKGCVQ
IVLKELDSPVPGYQHTILNYSWCRICKQVTPVVPLSNDSWSMSFAKYLELRFYGHQYTRRANAEPCGHSIHKDYHQYFSY
NQMVASFSYIPVRLLEICLPPPKIIIRNQGPSKSSLQQDLKDFSQKVAQVYLAIDDRLTSLKTDTFSKTREEKMEDMFAQ

KDMEESELRGWIEKLQVRLQTSVIDSSQQLQAVLESVVVKKQGLCETLQSWNNRLQELFQQEKGRKRLSVPPSPGRHRQP
TTDESKTSALESSPRNPSPVVPNGDKEDRHLNTFPSSSGSSSLLQLPSPAEQPSDVITSGPSFPDQDSVSNPEDMFDGHL
LGSNDSQVKEKSTMKTILANLLPGNSYNPIPFPFDPDKHYLMYEHERVPIAVCEREPSSIIAFALSCKEYKTALEELTKT
TAKTGGDDTSQTISAGESRVKNSPAKPSDNSMSQLSRSSVDADPLKEPESADKQKKQTGNPHIELQFSDANAKFYCRIYY
AEEFHKMREEIMESTEDDFVRSLSHCVNWQARGGKSGAVFYATEDDRFILKQMPRLEVQSFLDFAPHYFTYITGAVHLKR
PTALAKILGVYRIGYKNSQNNTEKKLDLLVMENLFYGRKMAQVFDLKGSLRNRNVKTDQGKESCEVVLLDENLLKLVHDN
PLYIRSHCKAILRAAIHSDALFLSSHLIIDYSLLVGRDDSTDELVVGIIDYIRTFTWDKKLEMVVKSTGILGGQGKMPTV
VSPELYRTRFCEAMDKYFLMVPDHWSGLGLNC
>NP_001007058.2 WD repeat and FYVE domain-containing protein 1 [Danio rerio]
MAAEIHSRPQSSRPVLLNKIEGHSDGVNAAVLIPKEDGVITVSEDRTIRVWLKRDSGQYWPSIYHTVSSPCSCVSYHHES
RRIFIGQDNGAVVEFLISEDLNKMNHVKTYPAHQNRVSDLLFSLESQWLVSTGHDKSISWMSTQSGSMLGRHSFGSWASC
LQYDNDTQHAFVGDYSGQITLLKLDEHSCSVITTLKGHEGSVGALYWDPVHRWLFSGASDHSVIMWDIGGRQGRTLLLQG
HHEKVQALRYLPLTRQLVSCSADGGITVWNMDTTRQEAPQWLDSDSCQKCEQPFFWNIKQMWDTKTLGLRQHHCRKCGK
A
ICGKCSSKRSTYPIMGFEFQVRMCDDCFNTIKEDDRTPLATFHEGKHSIAHMDMDPNRGLMVTCGSDRVVKIWDMTQVVG
SSVAAGFSHR
>NP_571631.2 faciogenital dysplasia [Danio rerio]
MMDSPDILHSDAQLSLSDRLSCTPAEISGLSDAVQCSKHTQESSEHTPMLQDVPPEADGETSGKIPNRDSGIDSPSCGGE
GECFPNEEHKQEYTHEEDKREHTHDVDKQEHTHEERKREYTHDEDKREYTHEEDSDLEEEEQEEEEEDQNTGIKASVCDP
QKLLNIAKELLQTEEAYVKRLNLLDQVFCARLTEAGIPADVITGIFSNISCIHRFHQQFLLPELHTRITQEWSCNPRIGD
ILQKLAPFLKMYGEYVKNFDRSMELVSLWTQRSGTFKSTVQSIQKQEVCGNLTLQHHMLEPVQRIPRYELLLKDYLKKLP
EGAADHRDAQNALELISTAANHSNAAIRKMEKMHKLLEVYERLGGEEDIVNPANELIKEGHIKKMSAKNGSAQDRYLYLF
NNMVLYCVPKLRLMGQKFSVRERIDIAGMEVHENVKQNVPHTFTITGKQRSLELQARTAEERDDWIKVILGTIEKHKQNS
ETFKAFNSSFSREDEHTPDSPLGPCSNTSICETDGAQHERKSSKKREKERETCKGCSEAFNFTKRKHHCKSCGAAVCGKC
LKVCDSRNIRMCRPCAEAEGAAGGATEHKKRVEKQLSVSQLSSSQRSGKGEEVEQEQILHR
>XP_021332905.1 FYVE, RhoGEF and PH domain-containing protein 5-like [Danio rerio]
MPDDVRQEGQSFRSIRRASEDTSRLSHLSLKIVGIIKNPKHKQHSNISVQKKMYFNMLHNSKSLSSPNSREQTPKVHRKI
VVEHKGSQERRTNKIVYPERRLQVDETEVEIILEQGDELGCGDELAHGVDLDLAEGLEKDKPLESEDDEVSVDLHSDLYS
KKEVDYNIVAEELQVKDMGKAAWGLTKNGKNKSSRNVQLASNAVYESLAHADRLLFDKMCTGKTLIESFYPSGIVADRMF
SSEEDIDVMFPDICDAGEALADKDGLSECDLFEQTEPLQLTETKDNINGDIMMLTIEEGLNSSDLSSVEEEYDEMQMEKE
LMEEQINKSVAGNKAAIPSNSEESDHPSNSSSSQTCVLSGSDTTLDEITNNNYNSKNMTESDEAISAILNLVLVQQQPE
TQDQNSQQYQDEESNGEDKEEQVVDNLSESEGKELMVDNVYEETEPKIQAKDFLQNRKYFMSRSISVETPGKRADLLSGL
PAGNGRLLLHPQAFYTDQCLFGDSRPALTGSLGCLSQGTRTSKVTRVDIPPPFELASITKRPIRKSSPSIPNEISASCKK

PDFGFKRYLLPLRFLRKSDRKSMMDTRSISSRSSSESSPQGSCRRMDFIRHNMGSPELQRSPDCTPPVSPSSFLSHRNRR
KEGLNFSLNNDLACSSISIEPGFLSEPLPLSKPRSFSSPNTDSSEYENVQNVASHYENVQIRLLNPVIQGQRNQSSPSDT
DGYVDMSSLPGFQGKSQSSEQETDSLYTFCSPNVRADGTVGVFVGVACAQEKKTTAPDKLTVSCSRAFYSAKELLDSEAQ
HVKTLQLLYESVNADERLMTVWTEVPDICTLHQDIHTLLETRLKEWDQNEGIAEIILAKKTEFSIFSSYISHYDDKLNYL
EHIQSTLSDSVTLKKQLLQVIVRILQYRMLLTDYLNNLSPDSREFENTQDALVVVSDVAYHASDSLKNGADLMRLVHIEH
SVLGLTDLIQPGRVFVKEGTLMKVSRKCKQPRHLFLMNDIMLYTYPQQDGKYRLINRLPLAGMKVSKPPMESAQSAIKIE
VKDISITLSARQETNTSHVLYCVCSVCLMLVFVRAAPALNETAGLSQ
>XP_697706.2 rabenosyn-5 [Danio rerio]
MASIYSPSLEDSAEVKEGFLCPLCFKDLQSFYQLQDHYEEEHAGDSRHVGGQLKNLVQKAKKAKDKLLKRDGDERTDSSY
ESFYYGGVDPYMWEPQELGATRSHLDYFKKQRAARIDHYVIEVNKCIIRLEKLTSFDRTNMDAAQIRSLEKSVVSWVNDQ
DVPFCPDCGGKFNLRNRRHHCRLCGSIMCRKCTEFVPLPMAYKLTSGTREALWAPGSGGSPVSTGQAVVQQTSRRGSISS
ISSVTSVLEEKDEDRVRCCHHCMDALLRHQRKLEEKDHVPDIVKLYERLRTCMDKVEAKAPEYTRMAESLNAGEKTYSLE
STAALRMEIQKYYELIDVLSKKILTLGLKEELQPHPKALQLQRMVRYTATLFIQEKLLGLTSLPTKEKYEELKEKRRVEQ
EKRAQQERQVSLESHKRRLENEKNRPTVSVNGDVAAPRVTKAGGWLPQSDTRSEQDDPLLQQIENIESFLRQARSANRSD
EVCMLEENLRQLQDEFDAQQTSRAIEISQRLADETDLQQKQIQHLQQREMEHRALAKTQERPLRGETDRTDEHSVRGDLE
KTDHTRRGGADQAEEQPFRANKAQYQSLRANKAEDPSLRANKTQYQSLRANKAEDPTLRASKTQEHSLRGDTSKTQEHSP
PSLENKSPSKNPFDEEPFSPVEEEPTNSQTANGKKEYNPFEEENDDPNAAKNPFEDDDDDDDDRSNPFKEVSDETPASST
NPFDEDDEDDTAIEEELLLQQIDNIRAYIFDAKLSGRSDEVELLSENLKELQRTLREQKRKK
>XP_017211441.1 FERM, RhoGEF and pleckstrin domain-containing protein 1 [Danio rerio]
MVEPEERPSVAGQRLGAPESLGISTLEPGQRPPTMPPGRQISIRVQMLDDTQEVFEVSQRAPGKALFDLVCSHLNLVEGD
YFGLEFQDQRKMIVWLDLLKPILKQIRRPKNIILRFVVKFFPPDHTQLLEELTRYLFALQIKHDLACGRLTCNESSAALL
VAHIVQSEIGDFDEVQCKQHLLNNKYIPEQDTLMDKIIGYHRKHVGQTPAESDYQLLEIARRLEMYGVRLHPAKDREGTR
LSLAVAHSGVLVFQGHTKINAFNWSKVRKLSFKRKRFLIKLRPDLNQSNCQDTLEFMMGSRDCCKVFWKICVEYHAFFRL
FEEPKPKPKPVLFTRGSSFRFSGRTQRQVIDYVKDTEFKKVPFERKHSKIQSNSNLSPFPSRQSAEGQGEMLQRNASASG
HQADSPLGRTQVTVENPALQRYTGSLNSQTNGCRNGTSDEADARLKPSPSKQQAEHLSTGLASHSAKGPVSRSPQHSESP
SAGQMMVNGQKQSLSVGQSPDGRQPSPLTSPLLYDAGFIRADDEDEVRRKRFPTDKAYFIAKELLTTERTYLKDLEVITV
SFQKAVLKDEAMPDSLRNLIFANFEPVYKFHETFLKDVEQRLAQWEGRSNAHIKGDYQRVGDVMLKNIQGLKLLSSHLQK
HSESLLELEQSCRSSRRLEVLCRDFELQKVCYLPLNIFFLRPLHRLLHYKQILERLCKHYPPTHDDFRDSRAALADVSEL
VLQLHTTLMKMENFQKLLELKKDLVGIDSLAAPGREFIRLGCLSKLSGKGLQQRMFFLFSDVVMYTSRGLTASNQFKVHG
QLPLYGMTIRESEDEWGVPYSFTLFGQRQSVVVAASSASEMEKWIEDIRMAIELADKSNGPTTEILSSSFSDSKSPEDCS
ADQESEDDLSSSRSSLERQTPHRGNTTVHVCWHRSTSVSMVDFSVALENQLSGNLLRKFKNSNGWQKLWVVFTNFSLFFY
KTHQDEYPLASLPLLGYSITIPSESENIHKDYVFKLHFKSHVYYFRSESEYTFERWMEVIRSATCSSSVLRSSSRKDPLV
Y

>XP_005171779.4 myotubularin-related protein 3-like [Danio rerio]
MVEEVQQSLEAHGSSGLQVEQVPFALLLGECVDHVEKTQECVLALTSYRLHIKLQENSISVPLQLIESVESHEPTQIQLT
CKDCKVIRCNFLSVDQCQDWLRRISAASGPPARLQELFAFSFLSCSNSVTAEDREVHDGICRAGGHKQRFKRELQRMGFD
THSAWRISNINSNYRVCSSYPEQILVPAAVTDQQLENVAAFRSWKRIPAVVYRHQSSGAVIGRCGQPEVSWWGWRNADDE
HLVQAIAKACATDPAANTALANGSCVHNHANGTISGSDPHMANGKVEAEPSATPSQKLLIMDARSYAAAVANRAKGGGCE
CPEYYPNCEVMFMGMANIHSIRKSFQCLRTLCAQVPDPLNWLSALEGTKWLQHLSLLLKASVLVVNAVDRDHRPVLVHCS
DGWDRTPQILALSKLMLDPFYRTIEGFEVLVETEWLDFGHKFSERCGHGERADDVNERCPVFLQWLDCVHQLQRQFPCSF
QFNEAFLVKLVQHTYSCLFGTFLCNSVKEREEQRVQEKTCSVWSLLRPANQSFHNILYSPRSETVLRPVCHVRNLMLWSA
VYLHNSAPSTPSDESCVPQSTPSASPIDTPVSRLPKTRSYENLSTSCENLTPNRRSSDPSVKEKWQDHLLALKINGENTH
TSPLQHTLALNTTAAHTQKTLELSVTDSQIENTEQEVTEELNRKLELLCSTSVSQDSSLNMKNNDQIFPEDISHIDTTSA
DGLHHKITHNPSQSSSQSSMESDSSSNHTAQKNPFHSPSQMSAAGDVSPPSGVWRCSAAFGPLERDGLSVHSDAVQVRLQ
QMETGHQLQVETLKKQVQELWSRIHANGKLSSLPDGDNNTESECLQRSGATDLTESSAEHIQQKDTEVSQWFSDQSSRCY
GCERRFWQAARRHHCRNCGNVFCTSCCDQKVASQQLREHKHMCQVCLSHLRPSPVPPALPPADLELEKPITASSN

<u>Domains architecture of Caenorhabditis elegans</u>

No	***Accession Number***	***Domains***
1	NP_499183.1	FYVE zinc finger, PH domain
2	NP_495565.2	Domain of unknown function (DUF3480), FYVE zinc finger,
3	NP_001379841.1	FYVE zinc finger
4	NP_001024127.1	FYVE zinc finger
5	NP_001024128.1	FYVE zinc finger
6	NP_001022045.1	Domain of unknown function (DUF3480), FYVE zinc finger,

7	NP_508756.2	FYVE zinc finger
8	NP_501375.2	VHS domain, Hepatocyte growth factor-regulated tyrosine kinase substrate, FYVE zinc finger
9	NP_001368458.1	FYVE zinc finger, RhoGEF domain
10	NP_001368457.1	FYVE zinc finger, RhoGEF domain
11	NP_001294046.1	FYVE zinc finger, RhoGEF domain
12	NP_001294045.1	FYVE zinc finger, RhoGEF domain
13	NP_501455.2	FYVE zinc finger, RhoGEF domain
14	NP_001294044.2	FYVE zinc finger, RhoGEF domain

15	NP_510155.3	Phosphatidylinositol-4-phosphate 5-Kinase, TCP-1/cpn60 chaperonin family, FYVE zinc finger
16	NP_001382742.1	Phosphatidylinositol-4-phosphate 5-Kinase, TCP-1/cpn60 chaperonin family, FYVE zinc finger
17	NP_001255656.1	Beige/BEACH domain, FYVE zinc finger, PH domain associated with Beige/BEACH
18	NP_001255659.1	Beige/BEACH domain, FYVE zinc finger, PH domain associated with Beige/BEACH
19	NP_001255657.1	Beige/BEACH domain, FYVE zinc finger, PH domain associated with Beige/BEACH
20	NP_001255658.1	Beige/BEACH domain, FYVE zinc finger, PH domain associated with Beige/BEACH
21	NP_001252086.1	Ankyrin repeats (3 copies), FYVE zinc finger
22	NP_001252087.1	Ankyrin repeats (3 copies), FYVE zinc finger

23	NP_001252085.1	Ankyrin repeats (3 copies), FYVE zinc finger, BTB/POZ domain
24	NP_001022794.2	Myotubularin-like phosphatase domain, FYVE zinc finger
25	NP_497766.3	Myotubularin-like phosphatase domain, FYVE zinc finger
26	NP_501352.1	FYVE zinc finger, Rabenosyn Rab binding domain
27	NP_001294172.1	FYVE zinc finger, Rabenosyn Rab binding domain
28	NP_495983.1	WD domain, G-beta repeat, FYVE zinc finger

Retrieved sequences of Caenorhabditis elegans

>NP_499183.1 Uncharacterized protein CELE_ZK632.12 [Caenorhabditis elegans]
MVDRLVNSEVNSRRMANVEQCFGKMGEQLSVFGRVLVGEGVLVKMCRKKPKQRQFFLFNDILVYGNIVISKKRYNKQRIL
RLEGVQVEDLEDDGIEKHGWIIKTPAKSFAVYAATETEKREWMLHIERCVTDLLERGNKQAATAHAAVWVPDGEAVKCMV
CGKTQFNLVQRRHHCRNCGRVVCGACSSRTFRIDNVHKKPVRVCDHCFDSLSSATPGQEESEPKTGNRLHHEDSSSDSED
EVNGSGRSSNESRPTFYREDVQQPAT
>NP_495565.2 FYVE-type domain-containing protein [Caenorhabditis elegans]

MEEVPDMDDLLDEMESDLVKKCCSVQSKPLLPPTSIGMQPRGIKHNPAPRTVVTVSKFVISEDMNEQMEGNINIDTSQLP
NTFQTKSSFPPNSDERNIGKENEVSARQEIAHKLKSVHNYSDATNFDPSKKVITQKFSQESEPESEPEPEPEEVSEVEED
TLVPETQIISNLNATARDTEPTATISKPNLDLDTEMEDMLAYLNDESVDKSSLKDKPAREEGGRDSIEESANESALYQFA
DRFSELVISEALNHRKMHYDPAVKEFYEANDSVPDVFAMSLDSTHSSLNSFNADNHNYLSEPENSSKKERMQSVAEENEA
SEEPTQSSSETEKNLQNEKIDVEFIENSEAIVQNESEDVPESNSTSGTVLENIEDHLESDVVIENGSESSFVNEKMNEAS
TAHLVAEQSVVDDSEIHLETEDQETLSPETADSSTNKNSDEFPDEGSQSEASVNNEEVLENVEHVEDECTVVDAVNENTD
SSIEENDEENTENRPRFDSSIATIHVLHESDSDESATPRRERRLTESELQLGKTSPYWIPDSECPNCMLCNTRFTIITRR
HHCRACGRVLCGSCCNEKAFLEYLQEEGKKLQAVRVCKPCSAMLARIETHEQDEQRRRESIASENGLSGDETISSIPAAS
TSIPRGVLKTKPTVHVNEEDGASTSSQAHSTSGNVSDSSRRSVMFRDGVRPGAPADEGNPEEERSTALKPKKKSRKRTSV
VRRIAELRMEDELACALPKEGITKLLIVKPDCEHPKFEDASTVLETLCNFSVVTVVLKKNLNCTVQIFNNPNFGLVWAVS
TQGFAQIGLDELMFSWTLNDQEKQKVDAEEPELDEKSALVVLPLPVLHRISLIYTQSTEHEYAGVRKVENRLMRVHSVTD
PTYPLTKHIMFFRPTVQVGLKNMRIPTNPFLIACFLHDDELNWATALPNRLLYKLGEKFNVFPTPFVNSIGRESTYSTDV
SGTVLKVFTDFRSWSYRMRHISGCTVSLTNDKTVIRIPKSAIKELKDVLNFSRSMVAWSCDLHCEDDSVLVCEETDPGLY
STQVFAKNIGQRESTSASFVILDGGSKVSSLQVNVVEDGVAVRLQSERLETILNAINDGNDAVESSKDMEFRVEFVEDGN
WICTDSDYYPKSQIDGLYLINKFQYGLSLERALTQVLQVQGINDYGIRMSHVYNLGDGRLQPEEEPKIYGMIEIAARECV
AMLEPHIQHLICAGIGSVSIRLFVSPYEFEYDVSKWLGLEAENDKYKQSLDQLIPMLYNMVEYVPNGFEVEFVLSIVSTR
ALPI
>NP_001379841.1 FYVE-type domain-containing protein [Caenorhabditis elegans]
MEEVPDMDDLLDEMESDLVKKCCSVQSKPLLPPTSIGMQPRGIKHNPAPRTVVTVSKFVISEDMNEQMEGNINIDTSQLP
NTFQTKSSFPPNSDERNIGKENEVSARQEIAHKLKSVHNYSDATNFDPSKKVITQKFSQESEPESEPEPEPEEVSEVEED
TLVPETQIISNLNATARDTEPTATISKPNLDLDTEMEDMLAYLNDESVDKSSLKDKPAREEGGRDSIEESANESALYQFA
DRFSELVISEALNHRKMHYDPAVKEFYEANDSVPDVFAMSLDSTHSSLNSFNADNHNYLSEPENSSKKERMQSVAEENEA
SEEPTQSSSETEKNLQNEKIDVEFIENSEAIVQNESEDVPESNSTSGTVLENIEDHLESDVVIENGSESSFVNEKMNEAS
TAHLVAEQSVVDDSEIHLETEDQETLSPETADSSTNKNSDEFPDEGSQSEASVNNEEVLENVEHVEDECTVVDAVNENTD
SSIEENDEENTENRPRFDSSIATIHVLHESDSDESATPRRERRLTESELQLGKTSPYWIPDSECPNCMLCNTRFTIITRR
HHCRACGRVLCGSCCNEKAFLEYLQEEGKKLQAVRLLPPQFLAVY
>NP_001024127.1 FYVE-type domain-containing protein [Caenorhabditis elegans]
MLRRLKEQVSQVANDIDGRLNNSRPSISSQGSQSQSQKSENDQEIEGFLCPMCMVELGGPDELTVHFEKEHSDFQTSANR
NHPDYTPPQQAPLAFATKDQEIEELRIRVNEEKRFAERIKEELDNIKSVMAIASEVTEDEIPYMAQQIQVLTADKGMVTR
QFLELEKESGQQSRELQQVKQERGDLMAKLKQMSVTMREITDESESGKVEMEDLKRELKVVKSDVVRYEIEVSRLEKMLD
QRPSEDDVNVLRTELVNAQKLMDAISQEKDIEIKEHLNSIRNLSMEREKQHIVNENLEKKIGEGEETVKQLQISYDAQSE
ELKQRNERVVQLEARIEENVFELSENKQNVKRLEDKVQESQDALQMLSNINGSNEEQMISLNSKFERNTAERKRIEAVFE
EKVTVQGERLKTLEMANLDLTNELASMGSLLDKERSLLEEKNKEISERDSSINDLKEKLAESEKKATKYKNELKEHADLV

ENLTLQLNKLQENSKDLMEKISAGEGGAKMAIEQLEQEKVKLTNELQTSSEKTKKASGELEAKISELEKKLRDAEASRTD
KEQKWKQEKESFERKLAEAEDEIKRKGERFVEMEKEMEEERQKATDRTLKLKDALVNSEKNLETIKKESEDREKIVREKD
AHLEENKKRIEDAVQKLEEAEKRARELEASVSSRDTTVSTKESELSELKGKLTESNSFIEELKVQVEKVSNEISEKQQEV
ENLMAEMRDKEAHWKTKRDEFEAQMLRNQEDNEEASSTLKSVQEQLMKEKETSGEEKNQLISVKSQLEELKTEVERLIRS
EEEKTQEIEKLKSAVTATTQERDELTATSESLRTECENLNSKIQSIEESRRHAEEKGSENLERMITEKSRLEKDIEERES
TIQSIQEALETKDNEIESLKTTQRVVEDELVSKISHIESFNSRIEEFEKEMASGKRTIERLEAEKAEETEKLVVFTGTQS
QKQEELEKLQKEIQEKETTIARMTSSKTQFEAMFADVQQTLSKEINDKTEEIERLMERIDSLEKVNHSRIEELESRLTQR
ERVVESLEADLAAVRNIEQEKLDELQKLKEEFDELKKAETMWQAEKDMLIERCLGSESDIEYEKERSQENKRRFDDALSA
MHELGRANQSLQIDADRYSSRKWLDDAEAINCTECGKVFSLTVRKHHCRVCGKIYCNPCSSKSVRIASAKNPVRACNTCF
TDSQK
>NP_001024128.1 FYVE-type domain-containing protein [Caenorhabditis elegans]
MLRRLKEQVSQVANDIDGRLNNSRPSISSQGSQSQSQKSENDQEIEGFLCPMCMVELGGPDELTVHFEKEHSDTSANRNH
PDYTPPQQAPLAFATKDQEIEELRIRVNEEKRFAERIKEELDNIKSVMAIASEVTEDEIPYMAQQIQVLTADKGMVTRQF
LELEKESGQQSRELQQVKQERGDLMAKLKQMSVTMREITDESESGKVEMEDLKRELKVVKSDVVRYEIEVSRLEKMLDQR
PSEDDVNVLRTELVNAQKLMDAISQEKDIEIKEHLNSIRNLSMEREKQHIVNENLEKKIGEGEETVKQLQISYDAQSEEL
KQRNERVVQLEARIEENVFELSENKQNVKRLEDKVQESQDALQMLSNINGSNEEQMISLNSKFERNTAERKRIEAVFEEK
VTVQGERLKTLEMANLDLTNELASMGSLLDKERSLLEEKNKEISERDSSINDLKEKLAESEKKATKYKNELKEHADLVEN
LTLQLNKLQENSKDLMEKISAGEGGAKMAIEQLEQEKVKLTNELQTSSEKTKKASGELEAKISELEKKLRDAEASRTDKE
QKWKQEKESFERKLAEAEDEIKRKGERFVEMEKEMEEERQKATDRTLKLKDALVNSEKNLETIKKESEDREKIVREKDAH
LEENKKRIEDAVQKLEEAEKRARELEASVSSRDTTVSTKESELSELKGKLTESNSFIEELKVQVEKVSNEISEKQQEVEN
LMAEMRDKEAHWKTKRDEFEAQMLRNQEDNEEASSTLKSVQEQLMKEKETSGEEKNQLISVKSQLEELKTEVERLIRSEE
EKTQEIEKLKSAVTATTQERDELTATSESLRTECENLNSKIQSIEESRRHAEEKGSENLERMITEKSRLEKDIEERESTI
QSIQEALETKDNEIESLKTTQRVVEDELVSKISHIESFNSRIEEFEKEMASGKRTIERLEAEKAEETEKLVVFTGTQSQK
QEELEKLQKEIQEKETTIARMTSSKTQFEAMFADVQQTLSKEINDKTEEIERLMERIDSLEKVNHSRIEELESRLTQRER
VVESLEADLAAVRNIEQEKLDELQKLKEEFDELKKAETMWQAEKDMLIERCLGSESDIEYEKERSQENKRRFDDALSAMH
ELGRANQSLQIDADRYSSRKWLDDAEAINCTECGKVFSLTVRKHHCRVCGKIYCNPCSSKSVRIASAKNPVRACNTCFTD
SQK
>NP_001022045.1 FYVE-type domain-containing protein [Caenorhabditis elegans]
MEEVPDMDDLLDEMESDLVKKCCSVQSKPLLPPTSIGMQPRGIKHNPAPRTVVTVSKFVISEDMNEQMEGNINIDTSQLP
NTFQTKSSFPPNSDERNIGKENEVSARQEIAHKLKSVHNYSDATNFDPSKKVITQKFSQESEPESEPEPEPEEVSEVEED
TLVPETQIISNLNATARDTEPTATISKPNLDLDTEMEDMLAYLNDESVDKSSLKDKPAREEGGRDSIEESANESALYQFA
DRFSELVISEALNHRKMHYDPAVKEFYEANDSVPDVFAMSLDSTHSSLNSFNADNHNYLSEPENSSKKERMQSVAEENEA
SEEPTQSSSETEKNLQNEKIDVEFIENSEAIVQNESEDVPESNSTSGTVLENIEDHLESDVVIENGSESSFVNEKMNEAS

TAHLVAEQSVVDDSEIHLETEDQETLSPETADSSTNKNSDEFPDEGSQSEASVNNEEVLENVEHVEDECTVVDAVNENTD
SSIEENDEENTENRPRFDSSIATIHVLHESDSDESATPRRERRLTESELQLGKTSPYWIPDSECPNCMLFTIITRRHHCR
ACGRVLCGSCCNEKAFLEYLQEEGKKLQAVRVCKPCSAMLARIETHEQDEQRRRESIASENGLSGDETISSIPAASTSIP
RGVLKTKPTVHVNEEDGASTSSQAHSTSGNVSDSSRRSVMFRDGVRPGAPADEGNPEEERSTALKPKKKSRKRTSVVRRI
AELRMEDELACALPKEGITKLLIVKPDCEHPKFEDASTVLETLCNFSVVTVVLKKNLNCTVQIFNNPNFGLVWAVSTQGF
AQIGLDELMFSWTLNDQEKQKVDAEEPELDEKSALVVLPLPVLHRISLIYTQSTEHEYAGVRKVENRLMRVHSVTDPTYP
LTKHIMFFRPTVQVGLKNMRIPTNPFLIACFLHDDELNWATALPNRLLYKLGEKFNVFPTPFVNSIGRESTYSTDVSGTV
LKVFTDFRSWSYRMRHISGCTVSLTNDKTVIRIPKSAIKELKDVLNFSRSMVAWSCDLHCEDDSVLVCEETDPGLYSTQV
FAKNIGQRESTSASFVILDGGSKVSSLQVNVVEDGVAVRLQSERLETILNAINDGNDAVESSKDMEFRVEFVEDGNWICT
DSDYYPKSQIDGLYLINKFQYGLSLERALTQVLQVQGINDYGIRMSHVYNLGDGRLQPEEEPKIYGMIEIAARECVAMLE
PHIQHLICAGIGSVSIRLFVSPYEFEYDVSKWLGLEAENDKYKQSLDQLIPMLYNMVEYVPNGFEVEFVLSIVSTRALPI
>NP_508756.2 Lateral signaling target protein 2 [Caenorhabditis elegans]
MQSFRKIWNKPRPDDWMPLARFYYADSALNDIASELDSFDGRRDPDRCNALVTRLRVAQDRVLHIITEMLIHLYPREQDR
ACRDFRVKFPDEILHDTLPGQLWFGAECLSAGSNIIDHETESDLIRPLAKDVTKQLDFLRDLLKNQSLRDPSAYNPVIKE
NLLKFDKLFAEFEYQYVSAMVPVKSVKEHDSQLDVAVLFSEVLSLALVKDLITQDLIDYCDPSVMIAIPRLGIVWGLLVY
SNGALNVDVPAENLSEMFRPFYSLLVKIRNLLRILTPTELTKLETVLCKGESAVPEDTSSTLTMSDFRTNATDEEKAKNN
QRVWMCDMPSDSTSSLDSSVQDSSSETTSLASSALASPHSGSEENVSQIENEEGDDEAIGTNSNSSNEVTESPETIEEPD
NVDMEESSESEVDTHIDETRNESDDEITDDVQASDVLQVETKKCKSSRLLEQKKFDKSVKTIIPMQTDPRSQIDPKNLRS
RFRSSEDLVHRLFVCIAGVADQLQTNYSSEIRKVLKLILQPSEIIPVYEVVNAQVANSQTEGEETGVEAQETLPLPAFMG
VRWVPDEDCEQCTACSMPFNFVRRRHHCRNCGRIFCHKCSCNTISIPEHGYDRKVRVCNLCYVHRLNSFGCNEPMSQVNE
NGATVPSVTEQQSAQTASASS
>NP_501375.2 Hepatocyte growth factor-regulated tyrosine kinase substrate [Caenorhabditis elegans]
MATKFQRVLDQATDSTLVEPNWEGIILCTDMIRSGEVPAKPSLQAIRKRMQHENPHVVNHTLLVLDACVKNCGHKVHAEV
ATREFMEDFKNLVTENKYDEVKNKSLEMLQCWATAFANKPEYKMVVDTHNLMKLAGFDFPSLKEADAMFMAQVAPEWA
DG
PECYRCRSVFSVFTRKHHCRACGQIFCDKCSSRELALPQFGIEKEVRVCETCYEKKVAEVKERYPAVKKQLDAAHGRKGD
SEADKAAKEKLLREKEEEDLALAIAISQSEAEAKEKEKQNNLYSMYNGIKPEVDGYKGAAEPVTAPPPDDSSSDPLARYL
NRDYWQQKKEGKVEDWSSRSSALSATAPPPSEPSIAPSICSTLMGPDDNSLNADIAAMSLGGTNGLNGTVSDDAKLQADD
TMKWCQSIREQVSVMDNRIRSNLARGRPVFNDSAIQDLFTKLTELHSHVLSRMHTLDEQRGYYESLQDHLANIGEARQAI
DEMREEHERKRQERMAEEQRLRQAQMQQTLEMMRMKKHAMLMEQREQALQRFQQQQQEMAMRRHQQQAYYNPQQM
GYGAP
PPSGPQQPPQQSYYGYQQGPQSQAPPPSSQYQQSHASTQQQQQQYYQHYQANQTVTQPVQQAQQQYQQQYQGYYQQQQP
Y

QQQAGYQNPQQHQNYQNGTTNENGQYQQQQSSEVKQEHQNHGYQQQQAQHPPASVASNGHNGYGNVDQNAHHHQQQQ
PQI
AEQPLISFD
>NP_001368458.1 Uncharacterized protein CELE_C28C12.10 [Caenorhabditis elegans]
MGFVPRMQNGGTGTPSERPISQVSTLSQVSDEFDEGDTSASDEESMNSEKHLRRHRHEDDFDELPLPKNERKTTTVAATH
SEIMNEMEHLFVRGGKKNNGVHKQQRRQSNIDEIPSDVGKLRDNRKGRHNSLFVSPTSGMSSSSTDDFSRITSMTSDRSS
ILTSHSGGEDSTDGASPVPDYETGDEKDDQRLKKLHYAAVEFLKVQNNYVQYLKEMAVLYPEYMERFGKRVGRDLLASHN
GHENVVLQIKKIMVQILPIHEMLLKEIDKVCSNWDSRYPNMSKTIGTFADFLKCCQPFLDNKADFLNKLLQLRNEDKEFD
EATYMFETEVFKRGKKGAVIQQLDQVHQNFMRYKLLMLRYSEYLIDDCDEKEKAQEAIQKLENVTQAVNQKMGLPTTEEL
TKLYYRFQCQFNVLEPGRVLIRQSEVMKQTRKELQPRYLVLFTDYLWICRVSSSGQFDINRSYRIPLEYMKFERMEEDER
IRCLQIRSRVKSALIIFSSEKERNQWTDDLTKAQYDRKSYKRRQSTAVQRHDENKKKMNKLLMLTPEVPDASDLERPQLI
DSRSRSMSCESQDELSSVPVTPLDNGEIDETLGFGEVTRNGSGKKPPSEMIKPVWLPDNISNECLMEGCSTEFNIINRRH
HCRDCGWLICKFCKGQAPLSKYDFTKQNVCSECFDRHYKAYKDGVLFPSKNMIVQSDETILVKIGKRNDKEIVDPRKLFK
APVNYGFRHRNVEEKRAQSIVFGRVYLRSRKTETVRHALLRRDDLKLVFYKAELDSKSVLELLIYGYFYRETPLDDGWLF
ELVHRNQIRTDDTKDDVISFRVDNSASAKKWSAAFADKLELDPTRG
>NP_001368457.1 Uncharacterized protein CELE_C28C12.10 [Caenorhabditis elegans]
MGFVPRMQNGGTGTPSERPISQVSTLSQVSDEFDEGDTSASDEESMNSEKHLRRHRHEDDFDELPLPKNERKTTTVAATH
SEIMNEMEHLFVRGGKKNNGVHKQQRRQSNIDEIPSDVGKLRDNRKGRHNSLFVSPTSGMSSSSTDDFSRITSMTSDRSS
ILTSHSGGEDSTDGASPVPDYETGDEKDDQRLKKLHYAAVEFLKVQNNYVQYLKEMAVLYPEYMERFGKRVGRDLLASHN
GHENVVLQIKKIMVQILPIHEMLLKEIDKVCSNWDSRYPNMSKTIGTFADFLKCCQPFLDNKADFLNKLLQLRNEDKEFD
EATYMFETEVFKRGKKGAVIQQLDQVHQNFMRYKLLMLRYSEYLIDDCDEKEKAQEAIQKLENVTQAVNQKMGLPTTEEL
TKLYYRFQCQFNVLEPGRVLIRQSEVMKQTRKELQPRYLVLFTDYLWICRVSSSGQFDINRSYRIPLEYMKFERMEEDER
IRCLQIRSRVKSALIIFSSEKERNQWTDDLTKAQYDRKSYKRRQSTAVQRHDENKKKMNKLLMLTPEVPDASDLERPQLI
DSRSRSMSCESQDELSSVPVTPLDNGEIDETLGFGEVTRNGSGKKPPSEMIKPVWLPDNISNECLMEGCSTEFNIINRRH
HCRDCGWLICKFCKGQAPLSKYDFTKQNVCSECFDRHYKAYKDGVLFPSKNMIVQSDETILVKIGKRNDKEIVDPRKLFK
APVNYGFRHRNVEEKRAQSIVFGRVYLSFRSRKTETVRHALLRRDDLKLVFYKAELDSKSVLELLIYGYFYRETPLDDGW
LFELVHRNQIRTDDTKDDVISFRVDNSASAKKWSAAFADKLELDPTRG
>NP_001294046.1 Uncharacterized protein CELE_C28C12.10 [Caenorhabditis elegans]
MNFELPSSSTSDVSSQNSPKCDPHPSCTTPTILVSPATSEYSNGVFFGANETNGSGVKDRARQLVNMGFVPRMQNGGTGT
PSERPISQVSTLSQVSDEFDEGDTSASDEESMNSEKHLRRHRHEDDFDELPLPKNERKTTTVAATHSEIMNEMEHLFVRG
GKKNNGVHKQQRRQSNIDEIPSDVGKLRDNRKGRHNSLFVSPTSGMSSSSTDDFSRITSMTSDRSSILTSHSGGEDSTDG
ASPVPDYETGDEKDDQRLKKLHYAAVEFLKVQNNYVQYLKEMAVLYPEYMERFGKRVGRDLLASHNGHENVVLQIKKIM
V

QILPIHEMLLKEIDKVCSNWDSRYPNMSKTIGTFADFLKCCQPFLDNKADFLNKLLQLRNEDKEFDEATYMFETEVFKRG
KKGAVIQQLDQVHQNFMRYKLLMLRYSEYLIDDCDEKEKAQEAIQKLENVTQAVNQKMGLPTTEELTKLYYRFQCQFNVL
EPGRVLIRQSEVMKQTRKELQPRYLVLFTDYLWICRVSSSGQFDINRSYRIPLEYMKFERMEEDERIRCLQIRSRVKSAL
IIFSSEKERNQWTDDLTKAQYDRKSYKRRQSTAVQRHDENKKKMNKLLMLTPEVPDASDLERPQLIDSRSRSMSCESQDE
LSSVPVTPLDNGEIDETLGFGEVTRNGSGKKPPSEMIKPVWLPDNISNECLMEGCSTEFNIINRRHHCRDCGWLICKFCK
GQAPLSKYDFTKQNVCSECFDRHYKAYKDGVLFPSKNMIVQSDETILVKIGKRNDKEIVDPRKLFKAPVNYGFRHRNVEE
KRAQSIVFGRVYLSFRSRKTETVRHALLRRDDLKLVFYKAELDSKSVLELLIYGYFYRETPLDDGWLFELVHRNQIRTDD
TKDDVISFRVDNSASAKKWSAAFADKLELDPTRG
>NP_001294045.1 Uncharacterized protein CELE_C28C12.10 [Caenorhabditis elegans]
MNFELPSSSTSDVSSQNSPKCDPHPSCTTPTILVSPATSEYSNGVFFGANETNGSGVKDRARQLVNMGFVPRMQNGGTGT
PSERPISQVSTLSQVSDEFDEGDTSASDEESMNSEKHLRRHRHEDDFDELPLPKNERKTTTVAATHSEIMNEMEHLFVRG
GKKNNGVHKQQRRQSNIDEIPSDVGKLRDNRKGRHNSLFVSPTSGMSSSSTDDFSRITSMTSDRSSILTSHSGGEDSTDG
ASPVPDYETGDEKDDQRLKKLHYAAVEFLKVQNNYVQYLKEMAVLYPEYMERFGKRVGRDLLASHNGHENVVLQIKKIM
V
QILPIHEMLLKEIDKVCSNWDSRYPNMSKTIGTFADFLKCCQPFLDNKADFLNKLLQLRNEDKEFDEATYMFETEVFKRG
KKGAVIQQLDQVHQNFMRYKLLMLRYSEYLIDDCDEKEKAQEAIQKLENVTQAVNQKMGLPTTEELTKLYYRFQCQFNVL
EPGRVLIRQSEVMKQTRKELQPRYLVLFTDYLWICRVSSSGQFDINRSYRIPLEYMKFERMEEDERIRCLQIRSRVKSAL
IIFSSEKERNQWTDDLTKAQYDRKSYKRRQSTAVQRHDENKKKMNKLLMLTPEVPDASDLERPQLIDSRSRSMSCESQDE
LSSVPVTPLDNGEIDETLGFGEVTRNGSGKKPPSEMIKPVWLPDNISNECLMEGCSTEFNIINRRHHCRDCGWLICKFCK
GQAPLSKYDFTKQNVCSECFDRHYKAYKDGVLFPSKNMIVQSDETILVKIGKRNDKEIVDPRKLFKAPVNYGFRHRNVEE
KRAQSIVFGRVYLRSRKTETVRHALLRRDDLKLVFYKAELDSKSVLELLIYGYFYRETPLDDGWLFELVHRNQIRTDDTK
DDVISFRVDNSASAKKWSAAFADKLELDPTRG
>NP_501455.2 Uncharacterized protein CELE_C28C12.10 [Caenorhabditis elegans]
MKYDMNHRKNSDDTPPASRTVKEMMAEFQNKLDDGDNRFRKQPPPPPSPRRAPPPPPHRKPASLSPPPDDENTIRPPSSS
ESSENIPEEPQELITPNTTRRSLGPKPTVAPKPLFLNGLLPSSSTSDVSSQNSPKCDPHPSCTTPTILVSPATSEYSNGV
FFGANETNGSGVKDRARQLVNMGFVPRMQNGGTGTPSERPISQVSTLSQVSDEFDEGDTSASDEESMNSEKHLRRHRHED
DFDELPLPKNERKTTTVAATHSEIMNEMEHLFVRGGKKNNGVHKQQRRQSNIDEIPSDVGKLRDNRKGRHNSLFVSPTSG
MSSSSTDDFSRITSMTSDRSSILTSHSGGEDSTDGASPVPDYETGDEKDDQRLKKLHYAAVEFLKVQNNYVQYLKEMAVL
YPEYMERFGKRVGRDLLASHNGHENVVLQIKKIMVQILPIHEMLLKEIDKVCSNWDSRYPNMSKTIGTFADFLKCCQPFL
DNKADFLNKLLQLRNEDKEFDEATYMFETEVFKRGKKGAVIQQLDQVHQNFMRYKLLMLRYSEYLIDDCDEKEKAQEAIQ
KLENVTQAVNQKMGLPTTEELTKLYYRFQCQFNVLEPGRVLIRQSEVMKQTRKELQPRYLVLFTDYLWICRVSSSGQFDI
NRSYRIPLEYMKFERMEEDERIRCLQIRSRVKSALIIFSSEKERNQWTDDLTKAQYDRKSYKRRQSTAVQRHDENKKKMN
KLLMLTPEVPDASDLERPQLIDSRSRSMSCESQDELSSVPVTPLDNGEIDETLGFGEVTRNGSGKKPPSEMIKPVWLPDN

ISNECLMEGCSTEFNIINRRHHCRDCGWLICKFCKGQAPLSKYDFTKQNVCSECFDRHYKAYKDGVLFPSKNMIVQSDET
ILVKIGKRNDKEIVDPRKLFKAPVNYGFRHRNVEEKRAQSIVFGRVYLRSRKTETVRHALLRRDDLKLVFYKAELDSKSV
LELLIYGYFYRETPLDDGWLFELVHRNQIRTDDTKDDVISFRVDNSASAKKWSAAFADKLELDPTRG
>NP_001294044.2 Uncharacterized protein CELE_C28C12.10 [Caenorhabditis elegans]
MKYDMNHRKNSDDTPPASRTVKEMMAEFQNKLDDGDNRFRKQPPPPPSPRRAPPPPPHRKPASLSPPPDDENTIRPPSSS
ESSENIPEEPQELITPNTTRRSLGPKPTVAPKPLFLNGLLPSSSTSDVSSQNSPKCDPHPSCTTPTILVSPATSEYSNGV
FFGANETNGSGVKDRARQLVNMGFVPRMQNGGTGTPSERPISQVSTLSQVSDEFDEGDTSASDEESMNSEKHLRRHRHED
DFDELPLPKNERKTTTVAATHSEIMNEMEHLFVRGGKKNNGVHKQQRRQSNIDEIPSDVGKLRDNRKGRHNSLFVSPTSG
MSSSSTDDFSRITSMTSDRSSILTSHSGGEDSTDGASPVPDYETGDEKDDQRLKKLHYAAVEFLKVQNNYVQYLKEMAVL
YPEYMERFGKRVGRDLLASHNGHENVVLQIKKIMVQILPIHEMLLKEIDKVCSNWDSRYPNMSKTIGTFADFLKCCQPFL
DNKADFLNKLLQLRNEDKEFDEATYMFETEVFKRGKKGAVIQQLDQVHQNFMRYKLLMLRYSEYLIDDCDEKEKAQEAIQ
KLENVTQAVNQKMGLPTTEELTKLYYRFQCQFNVLEPGRVLIRQSEVMKQTRKELQPRYLVLFTDYLWICRVSSSGQFDI
NRSYRIPLEYMKFERMEEDERIRCLQIRSRVKSALIIFSSEKERNQWTDDLTKAQYDRKSYKRRQSTAVQRHDENKKKMN
KLLMLTPEVPDASDLERPQLIDSRSRSMSCESQDELSSVPVTPLDNGEIDETLGFGEVTRNGSGKKPPSEMIKPVWLPDN
ISNECLMEGCSTEFNIINRRHHCRDCGWLICKFCKGQAPLSKYDFTKQNVCSECFDRHYKAYKDGVLFPSKNMIVQSDET
ILVKIGKRNDKEIVDPRKLFKAPVNYGFRHRNVEEKRAQSIVFGRVYLSFRSRKTETVRHALLRRDDLKLVFYKAELDSK
SVLELLIYGYFYRETPLDDGWLFELVHRNQIRTDDTKDDVISFRVDNSASAKKWSAAFADKLELDPTRG
>NP_510155.3 1-phosphatidylinositol-3-phosphate 5-kinase [Caenorhabditis elegans]
MGDASELQFFEKLEPPPETEEPTPVSGGYFSNIIGQIFRSDGSPQPNGVVPPTEERQNLERTESGESRTSEESGFQRSLT
DKFTGLLKSKVNPQLKDYNDSNFKQYWMPDSTGRECYQCEERFTTFRRRHHCRLCGQIFCAKCCSSHIDGAALGYMGELR
LCDYCARKVQRLAEEGKQTPTTSTTRSQTPVNSRKISFDRNTAHKNSDTVRTVSNGAIWSLCPPESSMPPEITSPPQLGS
RRNSLAQSSNGPGVPTILSVADLCASNSAMLTNSHSHPMITEEEESGPDWFRTMHPGMDGVINNETSDSADVFAYANLAG
AITNEFTEMMDARAAEPTSATTDRKITFPSLSLDESVMNAAKEEQRDNLEELFRRNTERILDEVMKREYIREDKWRDLIL
KSVYEVVENVTVNVPSGDTMNIADYVHVKKVHKKEGKVDSEIIWGVACSRSLVYKSLSEEDESSHTTESIMIVSGSIEYE
RVSNKLSSIEPIIVQEEKFLEKQIDRIATKRASLILVEGGVSHIAAQLLHKRGIKVAVNVKMSILQRISRATGADIVSNS
DSQLVEQNLGCCPEFQQRNMQQEDGRIKTLMIFADCQKETGCTVLLHGDDLKELVAVKRVVQFLVTIVYSNYLEQSYLNA
FNTTIARRQSDCVVCEKRRAIVYSQGEKTEFEKNLYATMLSSSPVIEFEPPLLETATGRECPLIAYFKQPLYKLLKPGDV
ELIKQGYEEDIVPIPKKEPLLVDRRHAFAQCNRGAILDNVARSFRLFGGINFRRRTAQIVKHRKIVETEKQPFRAKDVLD
PRVHQTLAVLFGSFSRKSPNAPYFCVRPWVVSMQYYKDHDMTIGEFLVKFCFNRSYECPSSNCEVPMLDHSRKLVYGKVC
VEISTQTVNEAENAIESEQQKSIMTWRNCGKCNCSSQMVKFDKAIWHLSFAKFLEYIGNSCFTTDTIYPITNQNQCSHCF
FHEKLYFFAMDNLVTTFKVIAIRPYSVVFSPIICSVKVLKVSRKELSDDVSRIATLALTACEDTNKQLAELDEEVQITPI
VVKLAGAIRNTMALASESRMFAKNILSGDEDLIRSNDRLYREVTGTFMKVREVTYNLIALWNENCAAIKYPKRTPEDIQE
IATLQKLENPFPSHLHLAIKLQPRLGVVVRDIQDTRGNFKPDIGSIIAYALSAVDYNKIPEAADTVSMDSASSSLKFSQM

DDGENLASSQHLEVEFEDESASYYVKMLYAEKFRKLRELLIAEGEETFIRSLSNSTFWTPQGGKSGSFFYRTQDDRFVVK
QMSRFEIQSFVKFAPNYFDYLTTSATESKLTTLCKVYGVFRIGYKSKTTTLKVDILVMEYLFYNHNVSQVWDLKGSLRNR
LASTGKSANEMVLLDENFVKDLWNQQLYVLPHSKAAMNQAISNDSHFLSSQYIMDYSLLVGVDDDNGELILGIVDYMRTY
TLDKKLESWVKIVAIPGAHLPTILSPEMYCARFSEAIDSYFPVVPDQWTGLGSIRSY
>NP_001382742.1 PIP Kinase [Caenorhabditis elegans]
MGDASELQFFEKLEPPPETEEPTPVSVSSGGYFSNIIGQIFRSDGSPQPNGVVPPTEERQNLERTESGESRTSEESGFQR
SLTDKFTGLLKSKVNPQLKDYNDSNFKQYWMPDSTGRECYQCEERFTTFRRRHHCRLCGQIFCAKCCSSHIDGAALGYMG
ELRLCDYCARKVQRLAEEGKQTPTTSTTRSQTPVNSRKISFDRNTAHKNSDTVRTVSNGAIWSLCPPESSMPPEITSPPQ
LGSRRNSLAQSSNGPGVPTILSVADLCASNSAMLTNSHSHPMITEEEESGPDWFRTMHPGMDGVINNETSDSADVFAYAN
LAGAITNEFTEMMDARAAEPTSATTDRKITFPSLSLDESVMNAAKEEQRDNLEELFRRNTERILDEVMKREYIREDKWRD
LILKSVYEVVENVTVNVPSGDTMNIADYVHVKKVHKKEGKVDSEIIWGVACSRSLVYKSLSEEDESSHTTESIMIVSGSI
EYERVSNKLSSIEPIIVQEEKFLEKQIDRIATKRASLILVEGGVSHIAAQLLHKRGIKVAVNVKMSILQRISRATGADIV
SNSDSQLVEQNLGCCPEFQQRNMQQEDGRIKTLMIFADCQKETGCTVLLHGDDLKELVAVKRVVQFLVTIVYSNYLEQSY
LNAFNTTIARRQSDCVVCEKRRAIVYSQGEKTEFEKNLYATMLSSSPVIEFEPPLLETATGRECPLIAYFKQPLYKLLKP
GDVELIKQGYEEDIVPIPKKEPLLVDRRHAFAQCNRGAILDNVARSFRLFGGINFRRRTAQIVKHRKIVETEKQPFRAKD
VLDPRVHQTLAVLFGSFSRKSPNAPYFCVRPWVVSMQYYKDHDMTIGEFLVKFCFNRSYECPSSNCEVPMLDHSRKLVYG
KVCVEISTQTVNEAENAIESEQQKSIMTWRNCGKCNCSSQMVKFDKAIWHLSFAKFLEYIGNSCFTTDTIYPITNQNQCS
HCFFHEKLYFFAMDNLVTTFKVIAIRPYSVVFSPIICSVKVLKVSRKELSDDVSRIATLALTACEDTNKQLAELDEEVQI
TPIVVKLAGAIRNTMALASESRMFAKNILSGDEDLIRSNDRLYREVTGTFMKVREVTYNLIALWNENCAAIKYPKRTPED
IQEIATLQKLENPFPSHLHLAIKLQPRLGVVVRDIQDTRGNFKPDIGSIIAYALSAVDYNKIPEAADTVSMDSASSSLKF
SQMDDGENLASSQHLEVEFEDESASYYVKMLYAEKFRKLRELLIAEGEETFIRSLSNSTFWTPQGGKSGSFFYRTQDDRF
VVKQMSRFEIQSFVKFAPNYFDYLTTSATESKLTTLCKVYGVFRIGYKSKTTTLKVDILVMEYLFYNHNVSQVWDLKGSL
RNRLASTGKSANEMVLLDENFVKDLWNQQLYVLPHSKAAMNQAISNDSHFLSSQYIMDYSLLVGVDDDNGELILGIVDYM
RTYTLDKKLESWVKIVAIPGAHLPTILSPEMYCARFSEAIDSYFPVVPDQWTGLGSIRSY
>NP_001255656.1 WD40 and FYVE domain protein [Caenorhabditis elegans]
METRDERTLSLLHLRKTFSEYLKIPVSGSRSNDPSRLLPLFHKVMSMYTPQQLNAEFKEVVHFATFLCSVLVKEVRQRAA
STGTIEAAQSIAEFLRPGTELKGYTILEAIRFLLSSEDEIMIDAACKVSLPSTLVKTIYLFFDLPAAATTVTVTDLTENA
TETEDEMLKNNEKLHDMIGQIMEGLCRFKCVSEELVRKDDLLLLFVGTTSQVAEANNCWRKLCARLLEVIADKSITNAVI
KYVIVKKCVRIFVKNLSSAPSNSHSDAQKTAESIICLSCFLKNSAYLTDQLLDEFYEADGYTVIKDFLLKSETDNEIVRN
ILLMAISLVNSGKFEITPQYTSGLVQLPAFQLPIPAGNGLSVRNLNAFALLYHVFLESTSESTSCTVIDILHSIYTCDPA
NYFILDKEYPLSIFIDQLERKPHAVRVKLLELIEFAVFQLSHIPCRELISLCVLLKTEISAGKTAMCTIIVQMCFKLITV
DAIIKDAFREVGLLDALCFIIRRMFEMYEKNIQNASFTVTSTSSRSQDQLKLSLLTTDLLTIIIKNNTENGKLFTECFGA
KLLIEIVCEVNEEWRSSLLQLVKQLLIVAPTDQYIMHLVNTLQDRIPIENLEVMFSLLKALLGVVRESHKVRIQFRKSGG

FLALSALLLGLEKRFTDLHPAGDGRIPREQCQMLEFVHIIFKVFTLSMRFEPSNAKYFSTEITWDSITSLLRLTSIFNES
TVISIEETEWKSLTHTDLASEIAACHEVFRLDDNIEAGNVPKGMPFNIYFGCYVCRLIFNMALDNYEKMTSDIRWNDDGA
SLEESIVSWTSSLLVHPGAIISMLCLLPSISASSMKWTIAAQYYVSLLLKAILKSERNQQIMCQVDMPKHLLRIAEKLFL
TENHILLQPFYYLLERLSYQSLTPNQLRSFLRLDSPLCCRSLDDDEDDDDDSKIEIADNEGGPVPLQRVKALVSMMTPRD
QYIGTAPSFVEFDMSVEGFAALYLPSLAPMFSTTKAERIFPPLNGFSFCTWIYLDALSDKKADAHPIRLLTVTRAVAPPN
SDPPSKKPSQTAHLACFQCQFSAFDRSLLISTEESDQPGADLEKTANFQTDKLIRITLADQVRCGEWFHLAVVFNRSVLK
SSQVSLYLNGRHISTQKLMYVAQNAGGAATQLAQTYSVNAVVGTLPALRRPSRLRFRLASIFLAEEPMTAETVRAVVQLQ
PHYIGNYQTASTAPLFHEEKIVFSLSAAATQELTLAKIRTMYGKMDAEILSQHLGISPNDHSTPLRVLCNTISHAPGAGR
TFGGVIVGYLGMRTFTPRPVPSLLDSIGGFASVYGLIAMAVDSEGLYASLKSLVSAIRSQPRLLATWNNNRSYQILAVLL
EDKAKMLNSHIMHMVFNVTGTADTSREHAPTISNPSAFEDLLCDLKVWKGAPSELHKMLLEHFYELITDHQLNNLQVVRK
SSLLSRILLIIHDEPTMIKNTDEIIFNLISAIMQPQCDSRSILKIGQSIAATLPTSESECYEDSHLPFHISEIQKLFIES
SDPPPEALHQVYIRNRLLNIIANFLANSNTQVQQQMCDQLVRTLGFDWLFALMSPGVHSGTIYLALRILLLILNQPSLLA
RFKEGSANGGWLSEADSVVRNRAAVVLGFSVSAHGGAVGSKIDINPELSNCGGFAALEHLMAAHADKPYPYYAMLSLLVG
QPISALRFCDQFNMELVWTHVFGLNSTSSVFEAINSANFCFDAIIPLFAMIRTSIYHQSSIHQPWTVTNPSTVVQMITFM
YQNSPAFFNIAHTDEFILALFSTLIEDTNAMGVKSEVANRRSQDGGGSPDAEYFQAFLAQPNVRIVMDLLKKICCDNLQV
NTSKNDTIIDAILDNISESGNTRKTQIACLTSLLHSVLEHTVGTDLLSSSALPPNTQAQNIVQIVANISLLSSRAVDAFW
NGLVFGSESMRMLSTLYHLQVIASKKVNKAVNAEPITGCIMRMTLFILSRPIDSVPVQLSVLDALSTLVSKRYLFLSSNE
AWFFASLTHLIFMLSVTPDVLFQDNSSSSDLDRTSAQVAMCACRVWSDVICAKQALIEETFKKQSVTDINAARALLSHSA
GVYWQQFVDSQLRAIQTGGSSAQGSAVTAKDIIQQQISSKFNRVASGITRFAAKRSMSTTTALPSSGSVAAWKSTSTDKQ
VIFMWLRVHVSLIKELVRAQSTRYTEWHAHVRKWCLHDWHQWEAELTRERGIWGPERASKLEKFKLDLTEGPTTRMRRKL
IPNRNFYHIYPFRPHLEAPSAKAQRAKVAISFDSKPYYETCLRHRRRTLDTRIIDSSNVSTASPEDGSGSSNLLGYSFTD
LSQINSSLIRRLSTTAPATSNVSGTSLELNEEECETSERKEDDESISSISEKTDSNGVQSSGPSTSSNTTKEAKKEEKKE
EKKAGPDNQTLLRLLEQGEQLHSMFRCARIQGLETAEGLLLFGRDHFYVVDGFTLLKTKEIRDLDFLSQEMHDPIVPYPA
TGATQPPKSSRLCSKFSYNMIREVHKRRYLLQPIALEVFSSDGRNYLLAFPKKIRDRVFDKLTSMAINLSSGGSDSLGGQ
KANVAIETTGRGASLLSSLIGQQSVTQRWLSGNISNFQYLMHLNTLAGRCYNDLSQYPIFPWVLADYTSSQLDFNNISTF
RDFSKPMGAQSPDRLEQFLKRFREWDDPSGETPPYMYGTHYSSAMIVVSYLVRLEPFTQQFLSLQGGHFDLADRMFHSVG
DAWTSASRNNMADVKELIPEFFTLPEMFTNTNHFDLGVKQNGIHVNDVLLPAWCHGDPREFIRLHRQALESDYVSSHLHE
WIDLIFGYKQNGEEAVKNSNLFHHLFYEGSVDFERIDDPLTRNATIGFVNNFGQIPTQLFKKPHPQKKVNILEGFSNTPG
VTTSRLFYHAIHNMSAPQTPFKELRSAIGSIHQNDKIGVVALEQNKVFIGTNRYITWGLPDRSVRMGQIDNDKSVCVHEM
CEVDEMTCAAAGDETTLFCGNTSGCITVWKVNNKPLSMKKLSVLNGHSDAITCLVSCQSHAVLVSASRDLTVLVWHLSEM
FLIRQLPKHPHAVLAVAVNDATGDIATACSTLLHVWTLNGELLAVLNTCDVAPAIDPQQMIISLAFSTMNEWDNDNVIMC
GTSDGIVKIYSCVVFENDGSVSEHPALENPNSNSNENHDRSAAIAARLEKQRKRLKNSSTTATSSSVSGSGSASLTSPGP
ASPSAGGSRATNIDVGGAQFVRVLVQRTALTMHTAFNRPDNVHPAPITAIAPSRDHRSLYVGDGIGRVWCWQSAEGGGRA

DHWVQDVTRQRCDDCEHKFTLADRKHHCRNCGQIFCSTCSRFESHITRMNISRPVRVCRKCFQRLQQSSNLHNL
>NP_001255659.1 WD40 and FYVE domain protein [Caenorhabditis elegans]
METRDERTLSLLHLRKTFSEYLKIPVSGSRSNDPSRLLPLFHKVMSMYTPQQLNAEFKEVVHFATFLCSVLVKEVRQRAA
STGTIEAAQSIAEFLRPGTELKGYTILEAIRFLLSSEDEIMIDAACKVSLPSTLVKTIYLFFDLPAAATTVTVTDLTENA
TETEDEMLKNNEKLHDMIGQIMEGLCRFKCVSEELVRKDDLLLLFVGTTSQVAEANNCWRKLCARLLEVIADKSITNAVI
KYVIVKKCVRIFVKNLSSAPSNSHSDAQKTAESIICLSCFLKNSAYLTDQLLDEFYEADGYTVIKDFLLKSETDNEIVRN
ILLMAISLVNSGKFEITPQYTSGLVQLPAFQLPIPAGNGLSVRNLNAFALLYHVFLESTSESTSCTVIDILHSIYTCDPA
NYFILDKEYPLSIFIDQLERKPHAVRVKLLELIEFAVFQLSHIPCRELISLCVLLKTEISAGKTAMCTIIVQMCFKLITV
DAIIKDAFREVGLLDALCFIIRRMFEMYEKNIQNASFTVTSTSSRSQDQLKLSLLTTDLLTIIIKNNTENGKLFTECFGA
KLLIEIVCEVNEEWRSSLLQLVKQLLIVAPTDQYIMHLVNTLQDRIPIENLEVMFSLLKALLGVVRESHKVRIQFRKSGG
FLALSALLLGLEKRFTDLHPAGDGRIPREQCQMLEFVHIIFKVFTLSMRFEPSNAKYFSTEITWDSITSLLRLTSIFNES
TVISIEETEWKSLTHTDLASEIAACHEVFRLDDNIEAGNVPKGMPFNIYFGCYVCRLIFNMALDNYEKMTSDIRWNDDGA
SLEESIVSWTSSLLVHPGAIISMLCLLPSISASSMKWTIAAQYYVSLLLKAILKSERNQQIMCQVDMPKHLLRIAEKLFL
TENHILLQPFYYLLERLSYQSLTPNQLRSFLRLDSPLCCRSLDDDEDDDDSKIEIADNEGGPVPLQRVKALVSMMTPRD
QYIGTAPSFVEFDMSVEGFAALYLPSLAPMFSTTKAERIFPPLNGFSFCTWIYLDALSDKKADAHPIRLLTVTRAVAPPN
SDPPSKKPSQTAHLACFQCQFSAFDRSLLISTEESDQPGADLEKTANFQTDKLIRITLADQVRCGEWFHLAVVFNRSVLK
SSQVSLYLNGRHISTQKLMYVAQNAGGAATQLAQTYSVNAVVGTLPALRRPSRLRFRLASIFLAEEPMTAETVRAVVQLQ
PHYIGNYQTASTAPLFHEEKIVFSLSAAATQELTLAKIRTMYGKMDAEILSQHLGISPNDHSTPLRVLCNTISHAPGAGR
TFGGVIVGYLGMRTFTPRPVPSLLDSIGGFASVYGLIAMAVDSEGLYASLKSLVSAIRSQPRLLATWNNNRSYQILAVLL
EDKAKMLNSHIMHMVFNVTGTADTSREHAPTISNPSAFEDLLCDLKVWKGAPSELHKMLLEHFYELITDHQLNNLQVVRK
SSLLSRILLIIHDEPTMIKNTDEIIFNLISAIMQPQCDSRSILKIGQSIAATLPTSESECYEDSHLPFHISEIQKLFIES
SDPPPEALHQVYIRNRLLNIIANFLANSNTQVQQQMCDQLVRTLGFDWLFALMSPGVHSGTIYLALRILLLILNQPSLLA
RFKEGSANGGWLSEADSVVRNRAAVVLGFSVSAHGGAVGSKIDINPELSNCGGFAALEHLMAAHADKPYPYYAMLSLLVG
QPISALRFCDQFNMELVWTHVFGLNSTSSVFEAINSANFCFDAIIPLFAMIRTSIYHQSSIHQPWTVTNPSTVVQMITFM
YQNSPAFFNIAHTDEFILALFSTLIEDTNAMGVKSEVANRRSQDGGGSPDAEYFQAFLAQPNVRIVMDLLKKICCDNLQV
NTSKNDTIIDAILDNISESGNTRKTQIACLTSLLHSVLEHTVGTDLLSSSALPPNTQAQNIVQIVANISLLSSRAVDAFW
NGLVFGSESMRMLSTLYHLQVIASKKVNKAVNAEPITGCIMRMTLFILSRPIDSVPVQLSVLDALSTLVSKRYLFLSSNE
AWFFASLTHLIFMLSVTPDVLFQDNSSSSDLDRTSAQVAMCACRVWSDVICAKQALIEETFKKQSVTDINAARALLSHSA
GVYWQQFVDSQLRAIQTGGSSAQGSAVTAKDIIQQQISSKFNRVASGITRFAAKRSMSTTTALPSSGSVAAWKSTSTDKQ
VIFMWLRVHVSLIKELVRAQSTRYTEWHAHVRKWCLHDWHQWEAELTRERGIWGPERASKLEKFKLDLTEGPTTRMRRKL
IPNRNFYHIYPFRPHLEAPSAKAQRAKVAISFDSKPYYETCLRHRRRTLDTRIIDSSNVSTASPEDGSGSSNLLGYSFTD
LSLSEINSSLIRRLSTTAPATSNVSGTSLELNEEECETSERKEDDESISSISEKTDSNGVQSSGPSTSSNTTKEAKKEEK
KEEKKAGPDNQTLLRLLEQGEQLHSMFRCARIQGLETAEGLLLFGRDHFYVVDGFTLLKTKEIRDLDFLSQEMHDPIVPY

PATGATQPPKSSRLCSKFSYNMIREVHKRRYLLQPIALEVFSSDGRNYLLAFPKKIRDRVFDKLTSMAINLSSGGSDSLG
GQKANVAIETTGRGASLLSSLIGQQSVTQRWLSGNISNFQYLMHLNTLAGRCYNDLSQYPIFPWVLADYTSSQLDFNNIS
TFRDFSKPMGAQSPDRLEQFLKRFREWDDPSGETPPYMYGTHYSSAMIVVSYLVRLEPFTQQFLSLQGGHFDLADRMFHS
VGDAWTSASRNNMADVKELIPEFFTLPEMFTNTNHFDLGVKQNGIHVNDVLLPAWCHGDPREFIRLHRQALESDYVSSHL
HEWIDLIFGYKQNGEEAVKNSNLFHHLFYEGSVDFERIDDPLTRNATIGFVNNFGQIPTQLFKKPHPQKKVNILEGFSNT
PGVTTSRLFYHAIHNMSAPQTPFKELRSAIGSIHQNDKIGVVALEQNKVFIGTNRYITWGLPDRSVRMGQIDNDKSVCVH
EMCEVDEMTCAAAGDETTLFCGNTSGCITVWKVNNKPLSMKKLSVLNGHSDAITCLVSCQSHAVLVSASRDLTVLVWHLS
EMFLIRQLPKHPHAVLAVAVNDATGDIATACSTLLHVWTLNGELLAVLNTCDVAPAIDPQQMIISLAFSTMNEWDNDNVI
MCGTSDGIVKIYSCVVFENDGSVSEHPALENPNSNSNENHDRSAAIAARLEKQRKRLKNSSTTATSSSVSGSGSASLTSP
GPASPSAGGSRATNIDVGGAQFVRVLVQRTALTMHTAFNRPDNVHPAPITAIAPSRDHRSLYVGDGIGRVWCWQSAEGGG
RADHWVQDVTRQRCDDCEHKFTLADRKHHCRNCGQIFCSTCSRFESHITRMNISRPVRVCRKCFQRLQQSSNLHNL
>NP_001255657.1 WD40 and FYVE domain protein [Caenorhabditis elegans]
METRDERTLSLLHLRKTFSEYLKIPVSGSRSNDPSRLLPLFHKVMSMYTPQQLNAEFKEVVHFATFLCSVLVKEVRQRAA
STGTIEAAQSIAEFLRPGTELKGYTILEAIRFLLSSEDEIMIDAACKVSLPSTLVKTIYLFFDLPAAATTVTVTDLTENA
TETEDEMLKNNEKLHDMIGQIMEGLCRFKCVSEELVRKDDLLLLFVGTTSQVAEANNCWRKLCARLLEVIADKSITNAVI
KYVIVKKCVRIFVKNLSSAPSNSHSDAQKTAESIICLSCFLKNSAYLTDQLLDEFYEADGYTVIKDFLLKSETDNEIVRN
ILLMAISLVNSGKFEITPQYTSGLVQLPAFQLPIPAGNGLSVRNLNAFALLYHVFLESTSESTSCTVIDILHSIYTCDPA
NYFILDKEYPLSIFIDQLERKPHAVRVKLLELIEFAVFQLSHIPCRELISLCVLLKTEISAGKTAMCTIIVQMCFKLITV
DAIIKDAFREVGLLDALCFIIRRMFEMYEKNIQNASFTVTSTSSRSQDQLKLSLLTTDLLTIIIKNNTENGKLFTECFGA
KLLIEIVCEVNEEWRSSLLQLVKQLLIVAPTDQYIMHLVNTLQDRIPIENLEVMFSLLKALLGVVRESHKVRIQFRKSGG
FLALSALLLGLEKRFTDLHPAGDGRIPREQCQMLEFVHIIFKVFTLSMRFEPSNAKYFSTEITWDSITSLLRLTSIFNES
TVISIEETEWKSLTHTDLASEIAACHEVFRLDDNIEAGNVPKGMPFNIYFGCYVCRLIFNMALDNYEKMTSDIRWNDDGA
SLEESIVSWTSSLLVHPGAIISMLCLLPSISASSMKWTIAAQYYVSLLLKAILKSERNQQIMCQVDMPKHLLRIAEKLFL
TENHILLQPFYYLLERLSYQSLTPNQLRSFLRLDSPLCCRSLDDDEDDDDDSKIEIADNEGGPVPLQRVKALVSMMTPRD
QYIGTAPSFVEFDMSVEGFAALYLPSLAPMFSTTKAERIFPPLNGFSFCTWIYLDALSDKKADAHPIRLLTVTRAVAPPN
SDPPSKKPSQTAHLACFQCQFSAFDRSLLISTEESDQPGADLEKTANFQTDKLIRITLADQVRCGEWFHLAVVFNRSVLK
SSQVSLYLNGRHISTQKLMYVAQNAGGAATQLAQTYSVNAVVGTLPALRRPSRLRFRLASIFLAEEPMTAETVRAVVQLQ
PHYIGNYQTASTAPLFHEEKIVFSLSAAATQELTLAKIRTMYGKMDAEILSQHLGISPNDHSTPLRVLCNTISHAPGAGR
TFGGVIVGYLGMRTFTPRPVPSLLDSIGGFASVYGLIAMAVDSEGLYASLKSLVSAIRSQPRLLATWNNNRSYQILAVLL
EDKAKMLNSHIMHMVFNVTGTADTSREHAPTISNPSAFEDLLCDLKVWKGAPSELHKMLLEHFYELITDHQLNNLQVVRK
SSLLSRILLIIHDEPTMIKNTDEIIFNLISAIMQPQCDSRSILKIGQSIAATLPTSESECYEDSHLPFHISEIQKLFIES
SDPPPEALHQVYIRNRLLNIIANFLANSNTQVQQQMCDQLVRTLGFDWLFALMSPGVHSGTIYLALRILLLILNQPSLLA
RFKEGSANGGWLSEADSVVRNRAAVVLGFSVSAHGGAVGSKIDINPELSNCGGFAALEHLMAAHADKPYPYYAMLSLLVG

QPISALRFCDQFNMELVWTHVFGLNSTSSVFEAINSANFCFDAIIPLFAMIRTSIYHQSSIHQPWTVTNPSTVVQMITFM
YQNSPAFFNIAHTDEFILALFSTLIEDTNAMGVKSEVANRRSQDGGGSPDAEYFQAFLAQPNVRIVMDLLKKICCDNLQV
NTSKNDTIIDAILDNISESGNTRKTQIACLTSLLHSVLEHTVGTDLLSSSALPPNTQAQNIVQIVANISLLSSRAVDAFW
NGLVFGSESMRMLSTLYHLQVIASKKVNKAVNAEPITGCIMRMTLFILSRPIDSVPVQLSVLDALSTLVSKRYLFLSSNE
AWFFASLTHLIFMLSVTPDVLFQFQDNSSSSDLDRTSAQVAMCACRVWSDVICAKQALIEETFKKQSVTDINAARALLSH
SAGVYWQQFVDSQLRAIQTGGSSAQGSAVTAKDIIQQQISSKFNRVASGITRFAAKRSMSTTTALPSSGSVAAWKSTSTD
KQVIFMWLRVHVSLIKELVRAQSTRYTEWHAHVRKWCLHDWHQWEAELTRERGIWGPERASKLEKFKLDLTEGPTTRMR
R
KLIPNRNFYHIYPFRPHLEAPSAKAQRAKVAISFDSKPYYETCLRHRRRTLDTRIIDSSNVSTASPEDGSGSSNLLGYSF
TDLSQINSSLIRRLSTTAPATSNVSGTSLELNEEECETSERKEDDESISSISEKTDSNGVQSSGPSTSSNTTKEAKKEEK
KEEKKAGPDNQTLLRLLEQGEQLHSMFRCARIQGLETAEGLLLFGRDHFYVVDGFTLLKTKEIRDLDFLSQEMHDPIVPY
PATGATQPPKSSRLCSKFSYNMIREVHKRRYLLQPIALEVFSSDGRNYLLAFPKKIRDRVFDKLTSMAINLSSGGSDSLG
GQKANVAIETTGRGASLLSSLIGQQSVTQRWLSGNISNFQYLMHLNTLAGRCYNDLSQYPIFPWVLADYTSSQLDFNNIS
TFRDFSKPMGAQSPDRLEQFLKRFREWDDPSGETPPYMYGTHYSSAMIVVSYLVRLEPFTQQFLSLQGGHFDLADRMFHS
VGDAWTSASRNNMADVKELIPEFFTLPEMFTNTNHFDLGVKQNGIHVNDVLLPAWCHGDPREFIRLHRQALESDYVSSHL
HEWIDLIFGYKQNGEEAVKNSNLFHHLFYEGSVDFERIDDPLTRNATIGFVNNFGQIPTQLFKKPHPQKKVNILEGFSNT
PGVTTSRLFYHAIHNMSAPQTPFKELRSAIGSIHQNDKIGVVALEQNKVFIGTNRYITWGLPDRSVRMGQIDNDKSVCVH
EMCEVDEMTCAAAGDETTLFCGNTSGCITVWKVNNKPLSMKKLSVLNGHSDAITCLVSCQSHAVLVSASRDLTVLVWHLS
EMFLIRQLPKHPHAVLAVAVNDATGDIATACSTLLHVWTLNGELLAVLNTCDVAPAIDPQQMIISLAFSTMNEWDNDNVI
MCGTSDGIVKIYSCVVFENDGSVSEHPALENPNSNSNENHDRSAAIAARLEKQRKRLKNSSTTATSSSVSGSGSASLTSP
GPASPSAGGSRATNIDVGGAQFVRVLVQRTALTMHTAFNRPDNVHPAPITAIAPSRDHRSLYVGDGIGRVWCWQSAEGGG
RADHWVQDVTRQRCDDCEHKFTLADRKHHCRNCGQIFCSTCSRFESHITRMNISRPVRVCRKCFQRLQQSSNLHNL
>NP_001255658.1 WD40 and FYVE domain protein [Caenorhabditis elegans]
METRDERTLSLLHLRKTFSEYLKIPVSGSRSNDPSRLLPLFHKVMSMYTPQQLNAEFKEVVHFATFLCSVLVKEVRQRAA
STGTIEAAQSIAEFLRPGTELKGYTILEAIRFLLSSEDEIMIDAACKVSLPSTLVKTIYLFFDLPAAATTVTVTDLTENA
TETEDEMLKNNEKLHDMIGQIMEGLCRFKCVSEELVRKDDLLLLFVGTTSQVAEANNCWRKLCARLLEVIADKSITNAVI
KYVIVKKCVRIFVKNLSSAPSNSHSDAQKTAESIICLSCFLKNSAYLTDQLLDEFYEADGYTVIKDFLLKSETDNEIVRN
ILLMAISLVNSGKFEITPQYTSGLVQLPAFQLPIPAGNGLSVRNLNAFALLYHVFLESTSESTSCTVIDILHSIYTCDPA
NYFILDKEYPLSIFIDQLERKPHAVRVKLLELIEFAVFQLSHIPCRELISLCVLLKTEISAGKTAMCTIIVQMCFKLITV
DAIIKDAFREVGLLDALCFIIRRMFEMYEKNIQNASFTVTSTSSRSQDQLKLSLLTTDLLTIIIKNNTENGKLFTECFGA
KLLIEIVCEVNEEWRSSLLQLVKQLLIVAPTDQYIMHLVNTLQDRIPIENLEVMFSLLKALLGVVRESHKVRIQFRKSGG
FLALSALLLGLEKRFTDLHPAGDGRIPREQCQMLEFVHIIFKVFTLSMRFEPSNAKYFSTEITWDSITSLLRLTSIFNES
TVISIEETEWKSLTHTDLASEIAACHEVFRLDDNIEAGNVPKGMPFNIYFGCYVCRLIFNMALDNYEKMTSDIRWNDDGA

SLEESIVSWTSSLLVHPGAIISMLCLLPSISASSMKWTIAAQYYVSLLLKAILKSERNQQIMCQVDMPKHLLRIAEKLFL
TENHILLQPFYYLLERLSYQSLTPNQLRSFLRLDSPLCCRSLDDDEDDDDDSKIEIADNEGGPVPLQRVKALVSMMTPRD
QYIGTAPSFVEFDMSVEGFAALYLPSLAPMFSTTKAERIFPPLNGFSFCTWIYLDALSDKKADAHPIRLLTVTRAVAPPN
SDPPSKKPSQTAHLACFQCQFSAFDRSLLISTEESDQPGADLEKTANFQTDKLIRITLADQVRCGEWFHLAVVFNRSVLK
SSQVSLYLNGRHISTQKLMYVAQNAGGAATQLAQTYSVNAVVGTLPALRRPSRLRFRLASIFLAEEPMTAETVRAVVQLQ
PHYIGNYQTASTAPLFHEEKIVFSLSAAATQELTLAKIRTMYGKMDAEILSQHLGISPNDHSTPLRVLCNTISHAPGAGR
TFGGVIVGYLGMRTFTPRPVPSLLDSIGGFASVYGLIAMAVDSEGLYASLKSLVSAIRSQPRLLATWNNNRSYQILAVLL
EDKAKMLNSHIMHMVFNVTGTADTSREHAPTISNPSAFEDLLCDLKVWKGAPSELHKMLLEHFYELITDHQLNNLQVVRK
SSLLSRILLIIHDEPTMIKNTDEIIFNLISAIMQPQCDSRSILKIGQSIAATLPTSESECYEDSHLPFHISEIQKLFIES
SDPPPEALHQVYIRNRLLNIIANFLANSNTQVQQQMCDQLVRTLGFDWLFALMSPGVHSGTIYLALRILLLILNQPSLLA
RFKEGSANGGWLSEADSVVRNRAAVVLGFSVSAHGGAVGSKIDINPELSNCGGFAALEHLMAAHADKPYPYYAMLSLLVG
QPISALRFCDQFNMELVWTHVFGLNSTSSVFEAINSANFCFDAIIPLFAMIRTSIYHQSSIHQPWTVTNPSTVVQMITFM
YQNSPAFFNIAHTDEFILALFSTLIEDTNAMGVKSEVANRRSQDGGGSPDAEYFQAFLAQPNVRIVMDLLKKICCDNLQV
NTSKNDTIIDAILDNISESGNTRKTQIACLTSLLHSVLEHTVGTDLLSSSALPPNTQAQNIVQIVANISLLSSRAVDAFW
NGLVFGSESMRMLSTLYHLQVIASKKVNKAVNAEPITGCIMRMTLFILSRPIDSVPVQLSVLDALSTLVSKRYLFLSSNE
AWFFASLTHLIFMLSVTPDVLFQFQDNSSSSDLDRTSAQVAMCACRVWSDVICAKQALIEETFKKQSVTDINAARALLSH
SAGVYWQQFVDSQLRAIQTGGSSAQGSAVTAKDIIQQQISSKFNRVASGITRFAAKRSMSTTTALPSSGSVAAWKSTSTD
KQVIFMWLRVHVSLIKELVRAQSTRYTEWHAHVRKWCLHDWHQWEAELTRERGIWGPERASKLEKFKLDLTEGPTTRMR
R
KLIPNRNFYHIYPFRPHLEAPSAKAQRAKVAISFDSKPYYETCLRHRRRTLDTRIIDSSNVSTASPEDGSGSSNLLGYSF
TDLSLSEINSSLIRRLSTTAPATSNVSGTSLELNEEECETSERKEDDESISSISEKTDSNGVQSSGPSTSSNTTKEAKKE
EKKEEKKAGPDNQTLLRLLEQGEQLHSMFRCARIQGLETAEGLLLFGRDHFYVVDGFTLLKTKEIRDLDFLSQEMHDPIV
PYPATGATQPPKSSRLCSKFSYNMIREVHKRRYLLQPIALEVFSSDGRNYLLAFPKKIRDRVFDKLTSMAINLSSGGSDS
LGGQKANVAIETTGRGASLLSSLIGQQSVTQRWLSGNISNFQYLMHLNTLAGRCYNDLSQYPIFPWVLADYTSSQLDFNN
ISTFRDFSKPMGAQSPDRLEQFLKRFREWDDPSGETPPYMYGTHYSSAMIVVSYLVRLEPFTQQFLSLQGGHFDLADRMF
HSVGDAWTSASRNNMADVKELIPEFFTLPEMFTNTNHFDLGVKQNGIHVNDVLLPAWCHGDPREFIRLHRQALESDYVSS
HLHEWIDLIFGYKQNGEEAVKNSNLFHHLFYEGSVDFERIDDPLTRNATIGFVNNFGQIPTQLFKKPHPQKKVNILEGFS
NTPGVTTSRLFYHAIHNMSAPQTPFKELRSAIGSIHQNDKIGVVALEQNKVFIGTNRYITWGLPDRSVRMGQIDNDKSVC
VHEMCEVDEMTCAAAGDETTLFCGNTSGCITVWKVNNKPLSMKKLSVLNGHSDAITCLVSCQSHAVLVSASRDLTVLVWH
LSEMFLIRQLPKHPHAVLAVAVNDATGDIATACSTLLHVWTLNGELLAVLNTCDVAPAIDPQQMIISLAFSTMNEWDNDN
VIMCGTSDGIVKIYSCVVFENDGSVSEHPALENPNSNSNENHDRSAAIAARLEKQRKRLKNSSTTATSSSVSGSGSASLT
SPGPASPSAGGSRATNIDVGGAQFVRVLVQRTALTMHTAFNRPDNVHPAPITAIAPSRDHRSLYVGDGIGRVWCWQSAEG
GGRADHWVQDVTRQRCDDCEHKFTLADRKHHCRNCGQIFCSTCSRFESHITRMNISRPVRVCRKCFQRLQQSSNLHNL

>NP_001252086.1 Uncharacterized protein CELE_F22G12.4 [Caenorhabditis elegans]
MTAALLYRLIDGNTKNVLHSIVSIGREDVLFLYFMQNSAKIPKILNDLDPQGASALEIALCSEHEKSRQIAAQLVEKGAD
VNVKDAERGETILMRMCRKENLSAMDFLLGNGADGRDSQSPGDYNVVHVASRIPSPALATWISENKEKLDLDKVDAEERT
PLICAVMANNHIMCETLIRSGVNCDVTTSEGHTALSTCLLMSDAPNRRIAELLMLNGAGVNFAICDSKTPFFNEIVSRKD
VASVEALLAANVDCHVADGRGQTACHVAAEAGAPEILSKIVEARRGLKWPRDADDRTALDIAVEKRDLKSARICIKGGAD
VNARDANGQTLLVKAILANDDEMGVFLIEHDAKAKNDERISSKTYVEAACERGLLNTVRSFISNGCKLNSRCSTGYSLLH
SALSHQKLDVASLLVNFGCDVESRVALGAGGEVLDDGDESWITKQTLLHRLIDDGDQQSAVFLIESGADVNARKEYRNPT
DDDQFTPAHMAVSWAQNDVLRALRDHSANLCDVDSDGRTPAHIGVREQNVDGVKILLDAENVEFIPIRDKFGQTILSQAM
AMKDHQIASLIVARQPHAAVQTNGNGENLLHQAIRQNDIESVLFLLAVAKADPCRPITDGSLKTPLHLAAIAKDEMILRN
LILVNDDVNVTSADGTTPLLEALKARNDKHAAILMENGAEPNVKDEYGENAMLCAVRSGSLDCIRAVADSSRTNRYARNK
IGYTSLHICALLTIDKLPKRTSSSDVIELVLIYAEEEAQAQNEKQFAGFIDARDADGNTALMIAYSQGNAGVCRSLLKRR
ACMGQRNNGDVNVFTYETATKQLLLGLLESLEAEPRWSDGDTCDCGARFSLTSRKHHCRHCGRHVCSKCSETTMPIAKYG
EEKRVRVCDVCAHVISTGTAPRR
>NP_001252087.1 Uncharacterized protein CELE_F22G12.4 [Caenorhabditis elegans]
MRMCRKENLSAMDFLLGNGADGRDSQSPGDYNVVHVASRIPSPALATWISENKEKLDLDKVDAEERTPLICAVMANNHIM
CETLIRSGVNCDVTTSEGHTALSTCLLMSDAPNRRIAELLMLNGAGVNFAICDSKTPFFNEIVSRKDVASVEALLAANVD
CHVADGRGQTACHVAAEAGAPEILSKIVEARRGLKWPRDADDRTALDIAVEKRDLKSARICIKGGADVNARDANGQTLLV
KAILANDDEMGVFLIEHDAKAKNDERISSKTYVEAACERGLLNTVRSFISNGCKLNSRCSTGYSLLHSALSHQKLDVASL
LVNFGCDVESRVALGAGGEVLDDGDESWITKQTLLHRLIDDGDQQSAVFLIESGADVNARKEYRNPTDDDQFTPAHMAVS
WAQNDVLRALRDHSANLCDVDSDGRTPAHIGVREQNVDGVKILLDAENVEFIPIRDKFGQTILSQAMAMKDHQIASLIVA
RQPHAAVQTNGNGENLLHQAIRQNDIESVLFLLAVAKADPCRPITDGSLKTPLHLAAIAKDEMILRNLILVNDDVNVTSA
DGTTPLLEALKARNDKHAAILMENGAEPNVKDEYGENAMLCAVRSGSLDCIRAVADSSRTNRYARNKIGYTSLHICALLT
IDKLPKRTSSSDVIELVLIYAEEEAQAQNEKQFAGFIDARDADGNTALMIAYSQGNAGVCRSLLKRRACMGQRNNGDVNV
FTYETATKQLLLGLLESLEAEPRWSDGDTCDCGARFSLTSRKHHCRHCGRHVCSKCSETTMPIAKYGEEKRVRVCDVCAH
VISTGTAPRR
>NP_001252085.1 Uncharacterized protein CELE_F22G12.4 [Caenorhabditis elegans]
MGSNKEPKIDYLDILKEQFNALQSEHLELRRKYELECSSSTPDSFPSRLLSLTSSLLEKPRFSDVTFKFAGNSLKSVPAH
KYVLAARTDFWKFENGGDEKSEIQISDDVDFEAFHVAIRWIYTDEIDLKMEDEKLLKVCETATSFRLEQLKNVCVQQLGA
RLHVDNCIKIYEFAEKQSLRQLSTVAGSMIAGAWPKLGPAHFAQMTAALLYRLIDGNTKNVLHSIVSIGREDVLFLYFMQ
NSAKIPKILNDLDPQGASALEIALCSEHEKSRQIAAQLVEKGADVNVKDAERGETILMRMCRKENLSAMDFLLGNGADGR
DSQSPGDYNVVHVASRIPSPALATWISENKEKLDLDKVDAEERTPLICAVMANNHIMCETLIRSGVNCDVTTSEGHTALS
TCLLMSDAPNRRIAELLMLNGAGVNFAICDSKTPFFNEIVSRKDVASVEALLAANVDCHVADGRGQTACHVAAEAGAPEI
LSKIVEARRGLKWPRDADDRTALDIAVEKRDLKSARICIKGGADVNARDANGQTLLVKAILANDDEMGVFLIEHDAKAKN

DERISSKTYVEAACERGLLNTVRSFISNGCKLNSRCSTGYSLLHSALSHQKLDVASLLVNFGCDVESRVALGAGGEVLDD
GDESWITKQTLLHRLIDDGDQQSAVFLIESGADVNARKEYRNPTDDDQFTPAHMAVSWAQNDVLRALRDHSANLCDVDSD
GRTPAHIGVREQNVDGVKILLDAENVEFIPIRDKFGQTILSQAMAMKDHQIASLIVARQPHAAVQTNGNGENLLHQAIRQ
NDIESVLFLLAVAKADPCRPITDGSLKTPLHLAAIAKDEMILRNLILVNDDVNVTSADGTTPLLEALKARNDKHAAILME
NGAEPNVKDEYGENAMLCAVRSGSLDCIRAVADSSRTNRYARNKIGYTSLHICALLTIDKLPKRTSSSDVIELVLIYAEE
EAQAQNEKQFAGFIDARDADGNTALMIAYSQGNAGVCRSLLKRRACMGQRNNGDVNVFTYETATKQLLLGLLESLEAEPR
WSDGDTCDCGARFSLTSRKHHCRHCGRHVCSKCSETTMPIAKYGEEKRVRVCDVCAHVISTGTAPRR
>NP_001022794.2 Myotubularin-related protein 3 [Caenorhabditis elegans]
MTVTSSAAIDIGGGGGGRRSDRLDSDRTSEDMSFIASPANESFQIGASFVDVQNESSGSIDTATATLHELNYTFGMPPVT
EESENMPQNYETVVELLPGEERAPINKLTEFPIEGGSLFVTNFRIVVILKDKEVEEALRFLVFPLQDIEQIDLAIPAFIH
LSLKIGRMFTICFKTAEDAALVHKILYTAFQRLNRPISSIYTSRPQDWTSKNTDNPMQSLNAFAWKFSEAVDELDRDGKL
PSWLLRADSVAQEITHIDFNRLGMSEHFQISSVNENFEVCPTYPEKIIVPKGITDDDIRKGAPYRSIGRFPAVIWRCRKT
RAVLMRSSQPQVGILSWRNPTDEKIIEEAVKASRIEGEEKKQFIIMDARGYTSAFANRARSGGFENTEYYQQAKLEFLGL
PNIHAVRGSFNNVRTMLHNLGPNEQLLTSLQTTGWLLNLSNLLVNAANCADHLSKGHSVLVHCSDGWDRTTQVTTLAKIM
LDEYYRTVKGFEELIRRDWIAFGHKLYDRQLVAFGNWGTSDERSPVFLQFLEAVRHLQREQPTLFQFTHAYLIKLAKHAY
SGLFGSFLFNSHKERREAMEKCKGTLVDIWRFIGPHNEEYVNQSFDEHYTGAVKPVNVSVINLRVWHEVFADEEEHYTQI
FSPKEERPLSGCTTPMNTSTSTNLVKSKSSESINSLNVDGSAKESSQQHPTCSTTPSDNTNSLPMSTSFIQQSLYQPKVR
GVAAIDRDGVIRFEDDEQAMLRKKNKLRAEEIRRKDEKIEELRRRAVLDTNKVSPGQRQSYSESDVETTGTLERVMSDVS
MVDPVNELPHFKPNTTWEGESGHCAYCKKEFNKLSVYVEDRQHHCRNCGRVVCEDCSKNRFSVIEEGKSVQKRACDSCYD
SMHETDLKLSSSSTTTTSSSTKIENDSNVPGLDNNSDNVSENVSENAIPDIIVEEKEAEDPIKEAESPSKETKCPKTLRN
FISFSPKSSMRKNKVHSRDPLKSIDEGSSSQQAESDDVLDVNEQPL
>NP_497766.3 Myotubularin-related protein 3 [Caenorhabditis elegans]
MTVTSSAAIDIGGGGGGRRSDRLDSDRTSEDMSFIASPANIGASFVDVQNESSGSIDTATATLHELNYTFGMPPVTEESE
NMPQNYETVVELLPGEERAPINKLTEFPIEGGSLFVTNFRIVVILKDKEVEEALRFLVFPLQDIEQIDLAIPAFIHLSLK
IGRMFTICFKTAEDAALVHKILYTAFQRLNRPISSIYTSRPQDWTSKNTDNPMQSLNAFAWKFSEAVDELDRDGKLPSWL
LRADSVAQEITHIDFNRLGMSEHFQISSVNENFEVCPTYPEKIIVPKGITDDDIRKGAPYRSIGRFPAVIWRCRKTRAVL
MRSSQPQVGILSWRNPTDEKIIEEAVKASRIEGEEKKQFIIMDARGYTSAFANRARSGGFENTEYYQQAKLEFLGLPNIH
AVRGSFNNVRTMLHNLGPNEQLLTSLQTTGWLLNLSNLLVNAANCADHLSKGHSVLVHCSDGWDRTTQVTTLAKIMLDEY
YRTVKGFEELIRRDWIAFGHKLYDRQLVAFGNWGTSDERSPVFLQFLEAVRHLQREQPTLFQFTHAYLIKLAKHAYSGLF
GSFLFNSHKERREAMEKCKGTLVDIWRFIGPHNEEYVNQSFDEHYTGAVKPVNVSVINLRVWHEVFADEEEHYTQIFSPK
EERPLSGCTTPMNTSTSTNLVKSKSSESINSLNVDGSAKESSQQHPTCSTTPSDNTNSLPMSTSFIQQSLYQPKVRGVAA
IDRDGVIRFEDDEQAMLRKKNKLRAEEIRRKDEKIEELRRRAVLDTNKVSPGQRQSYSESDVETTGTLERVMSDVSMVDP
VNELPHFKPNTTWEGESGHCAYCKKEFNKLSVYVEDRQHHCRNCGRVVCEDCSKNRFSVIEEGKSVQKRACDSCYDSMHE

TDLKLSRATSLSEMSSFDSFGPSSPPASSTSSSSLNMLASMSPTRPQPIPISCKSSSNSISSPRPSPGEFSRHSSTQAVK
G
>NP_501352.1 FYVE-type domain-containing protein [Caenorhabditis elegans]
MIGATGSGGTGGHNDVVRQGFICPFCMEDFGEYERLICHVENDHPEEDSSDLAGMFVSNVKGFFDKAKRGIQKLDAKKSI
ADLASNVETEFTETTEKLVERAPLPRIPKPLPPDEILPGTRRSKTREFLSTRELNINEVAVRTNMLIIRLDRLINGGPKD
KDPVKRKEFEREVVPWLDDAEAVCCPLCASRFGLTRRRHHCRLCGRVLCHSCSKFLSFDTAKTLTSSSNPLGDTHIIVEE
PNTVENEQELLQPEENGRRSFFSLSQKSMDKMKRAMAGAVQKVHSAATGEEIIAGALNEQDLSEHMRVCNLCLRDLHFRE
SQMDKKEPPEFVQQYDLMKVMIEEVKNLVPTYVRVVTSLKNGESVYSHKHGEELRSKCLEIQKSIDYVSKKIAEGANDKP
NSAKEQQLRKNIRLYAVQALQGLIGQMESLPTAEECSNLIIKRKERRAKDFARVNRTVMKMSSSMPQLQEQHSHLNSTSS
VSSFGGPSSIDDGWSAEDNDLVFEPSSIEDDTIQTDHPLYEQREQLRKFLFQASATGKLDEMDILERNLKEIEEEMMRLG
LSC
>NP_001294172.1 FYVE-type domain-containing protein [Caenorhabditis elegans]
MIGATGSGGTGGHNDVVRQGFICPFCMEDFGEYERLICHVENDHPEEDSSDLAGMFVSNVKGFFDKAKRGIQKLDAKKSI
ADLASNVETEFTETTEKLVERAPLPRIPKPLPPDEILPGTRRSKTREFLSTRELNINEVAVRTNMLIIRLDRLINGGPKD
KDPVKRKEFEREVVPWLDDAEAVCCPLCASRFGLTRRRHHCRLCGRVLCHSCSKFLSFDTAKTLTSSSNPLGDTHIIVEE
PNTVENEQELLQPEENGRRSFFSLSQKSMDKMKRAMAGAVQKVHSAATGEEIIAGALNEQDLSEHMRVCNLCLRDLHFRE
SQMDKKEPPEFVQQYDLMKVMIEEVKNLVPTYVRVVTSLKNGESVYSHKHGEELRSKCLEIQKSIDYVSKKIAEGANDKP
NSAKEQQLRKNIRLYAVQALQGLIGQMESLPTAEECSNLIIKRKERRAKDFARVNRTVMKMSSSMPQLQEQHSHLNSTSS
VSSFGGPSSIDDGWSAEDNDLSRVFEPSSIEDDTIQTDHPLYEQREQLRKFLFQASATGKLDEMDILERNLKEIEEEMMR
LGLSC
>NP_495983.1 WD repeat and FYVE domain-containing protein 2 [Caenorhabditis elegans]
MAAIINQRVEQGESSMGGAKPALLHRITGHVARINDVILLSKDEGVWTASDDRSVRLYLKRDNDQFWPSIHHFMPVAPTS
LYYSEETYKLLVGLINGNVYEFSVADDFNSMTESRKWTCHAGPISGLGFALSSELIFSCSRDKSIVWHCSENSNKMGSYL
LENSCTAMVIDLPFVFVGDHGGHVTVLRIVDNQPNLVSKLSAHTNAITSLTWDGNKKVLYSGSSDHLIIMWDIGGGRGEA
YELNGHNGKVTTLCAAPAAKRLFSADEHGKLMCWDMNCKRVETPEWKTSDCCQKCNQPFFWNLQAMWQRKVVGLRQH
HCRTCGSAVCGSCCDNWTTYPPMGYETKIRICNDCNARMKDNPQNFNLTPLAIPHEIHTGITAMHLQETLGLLVTSGQNRVI
MIWDVRSVCSAPSGSQ

Domain Architecture of Saccharomyces cerevisiae

No	***Accession Number***	***Domains***
1	NP_014403.3	VHS domain,FYVE zinc finger<br>Ubiquitin interaction motif<br>Ubiquitin interaction motif
2	NP_010599.1	FYVE zinc finger,Ring finger domain
3	NP_011492.3	FYVE zinc finger
4	NP_116674.2	Phosphatidylinositol-4-phosphate 5-Kinase, TCP-1/cpn60 chaperonin family, FYVE zinc finger
5	NP_010610.3	FYVE zinc finger, Rabenosyn Rab binding domain

Retrieved Sequences of Saccharomyces cerevisiae

>NP_014403.3 ESCRT-0 subunit protein VPS27 [Saccharomyces cerevisiae S288C]
MSVSTPSELDALIEQATSESIPNGDLDLPIALEISDVLRSRRVNPKDSMRCIKKRILNTADNPNTQLSSWKLTNICVKNG
GTPFIKEICSREFMDTMEHVILREDSNEELSELVKTILYELYVAFKNDSQLNYVAKVYDKLISRGIKFPEKLTLSNSPTA

MFDSKTPADWIDSDACMICSKKFSLLNRKHHCRSCGGVFCQEHSSNSIPLPDLGIYEPVRVCDSCFEDYDLKRHDDSKKS
KKHRHKRKKDRDYSTPEDEEELIRKAIELSLKESRNSASSEPIVPVVESKNEVKRQEIEEEEDPDLKAAIQESLREAEEA
KLRSERQKASRQMQPQQPSPQPQPIHSVDLSDEEKDSIYMFASLVEKMKSRPLNEILEDSKLQNLAQRVFASKARLNYAL
NDKAQKYNTLIEMNGKISEIMNIYDRLLEQQLQSINLSQQYTLPQVPSDPYNYLTENVQNPAESYQTPPLQQLSSHQYKP
QQDVSRQQSVKANSSPTTNIDHLKTIDVTPHAQQKPQSHVELAPSDPPYPKEEAEDEGTQAVQDEESSTQESRERPYPVE
TENGETSINKRPQGITRYDFPTVPARKFVQPESTVPLPASSSEIPIKEERPPSPQEELLIEL
>NP_010599.1 phosphatidylinositol-3-phosphate-binding ubiquitin-protein ligase [Saccharomyces cerevisiae S288C]
MVIKEDCINNLARWQADEEAHSCFQCKTNFSFLVRRHHCRCCGRIFCSSCTENFVNYNKKRVHALQKKNSDVESPPYRTC
NECYDNLLHLNLLVSSTNRDVRLSQTSVPPNALALSAPDSNTDEDAEILEDSVDQSGTACRSEESSQNEEDHFCPICNSD
LTQFPDEEETRKHVEDCIQRAENAQQHTNTSDAADDSVKESPAFQNRMLVYKISPNTTDNAIKECPICFENMEPGEKVGR
LECLCVFHYKCIKNWFHKRAQMTAAQKGNGHAFVKRNFCPFHDAVF
>NP_011492.3 Pib2p [Saccharomyces cerevisiae S288C]
MTALHSVSKTPAIKEEEEDGDERDGRGVPLGPRNHDYRGRKGDEESGADTVTSPITFEKKKIAPRASTHSEQSILSSISL
KSMVNQHRQQQLQQESSTGAGTGFVDRKQQIQSPAMVSILRKNSAEENVRSSHSSKLGEGQIDGRKASASKEIGKTLPFT
DDQRSNPELDPTNSVVDVSRGKNTKSKTVFNELEDDADDDDEVRQKNLTTQALRKLSSFKMNASSNLRLSKENKAKESSS
SSTSSVSSSSTSKVENIVDKLTTTNSSSMSQLRFGNTNVIIDSVNHAAKPPHQQMLRKPSLEFLPQPASSTNLNFNSNKH
KSNVRQISNPKKPLYIPAVLRKVSETNITNDDLLNATLSSYYKKASNLEHGFNPSKSQSASVQNANNLRIISSQSSVQSN
TSSILESYKNKISSYLFPNSIPNSDRINLIPTISNRNSARVNPPTKDHWIPDSKRNSCRYCHKPFTLWERKHHCRHCGDI
FCQDHLRHWLYLDSQANFIMINELNNGGINGGGTLCKICDDCLVEYENLSTTNHNANTNEDNINVEEGEDDDNDNRKKLR
NYYKNRQMNALFRPKKGGSSQEHATVDRDTTTPIQVKSNDEEADNENTGGEQEEGNDVLGSVIGSVPANWNWSSF
>NP_116674.2 1-phosphatidylinositol-3-phosphate 5-kinase [Saccharomyces cerevisiae S288C]
MSSEEPHASISFPDGSHVRSSSTGTSSVNTIDATLSRPNYIKKPSLHIMSTSTTSTTTDLVTNPILSNISVPKISPPTSS
SIATATSTSHVTGTASHSNIKANANTSTSVNKKNLPPTTSGRIPSSTIKRYPSRYKPSHSLQLPIKNDSNFKRSSIYASK
STVTAIPIRNNRPISMQNSYARTPDSDHDDVGDEVSSIKSASSSLTASLSKSFLFAFYNNRKKDKTSNNGVLSKEYWMKD
ESSKECFSCGKTFNTFRRKHHCRICGQIFCSSCTLLIDGDRFGCHAKMRVCYNCYEHADTYEDSSDEENDSTMQLNEPRS
RSRSRSSNTNPYSHSHSHLHLISQDNHNGTDLHDPVAATDNPQQQNEVYLLNDDDVQSIMTSGEDSKLFISTPPPPPKMA
IPATKQGGSLEISFDSENDRALHYQDDNPGRHHHLDSVPTRYTIRDMDNISHYDTNSNSTLRPHYNTNNSTITINNLNNT
TSNNSNYNNTNSNSNINNPAHSLRRSIFHYVSSNSVNKDSNNSSATPASSAQSSSILDPANRIIGNYAHRNYKFKFNYNS
KGPSQQNDTANGNNDNNNNNNNNNNNNNNNNSASGIADNNNIPSNDNGTTFTLDKKKRNPLTKSKSTSAYLEYPLNEEDSS
EDEGSMSIYSVLNDDHKTDNPIRSMRNSTKSFQRAQASLQRMRFRRKSKSKHFPNNSKSSIYRDLNFLTNSTPNLLSVVS
DDNLYDDSSPLQDKASSSAASRLTDRKFSNSSGSNNNSNSNSNINTDPWKRIASISGFKLKKEKKRELNEVSLLHMHALL
KQLLNDQEISNLQEWITLLDGALRKVLRTILNARDLNTLDFRQTYVKIKRISGGSPQNSEYIDGVVFSKALPSKTMPRHL
KNPRILLIMFPLEYQKNNNHFLSIESVFRQEREYLDKLVSRLKSLHPDIIYVGANVSGYALELLNDSGIVVQFNMKPQVI

ERIAKLTEADIAISVDKLATNIKMGECETFEVKSYIYGNISKTYTFLRGCNPELGGTILLRGDSLENLRKIKQVSEFMVY
AIFSLKLESSFFNDNFIQLSTDVYLKRAESKKLQVFEGYFADFLIKFNNRILTVSPTVDFPIPFLLEKARGLEKKLIERI
NQYESESDLDRQTQLNMLQGLESTITKKHLGNLIKFLHEMEIENLELEFQKRSRQWEVSYSSSQNLLGTGSHQSITVLYS
MVSTKTATPCVGPQIVTIDYFWDSDISIGQFIENVVGTARYPCQQGCNGLYLDHYRSYVHGSGKVDVLIEKFQTRLPKLK
DIILTWSYCKKCGTSTPILQISEKTWNHSFGKYLEVMFWSYKDSVTGIGKCPHDFTKDHVKYFGYNDLVVRLEYSDLEVH
ELITPPRKIKWKPHIDIKLKVELYYKILEKINNFYGSVLSRLERIKLDSMTKDKVLSGQAKIIELKSNATEEQKLMLQDL
DTFYADSPCDQHLPLNLVIKSLYDKAVNWNSTFAIFAKSYLPSETDISRITAKQLKKLFYDSSRKDSEDKKSLHDEKAKT
RKPEKNELPLEGLKDVEKPKIDSKNTTENRDRTNEPQNAVTITTFKDDTPIIPTSGTSHLTVTPSASSVSSSLTPQTEER
PPISRSGTGISMTHDKSTRPNIRKMSSDSSLCGLASLANEYSKNNKVSKLATFFDQMHFDALSKEFELERERERLQLNKD
KYQAIRLQTSTPIVEIYKNVKDAVDEPLHSRSSGNNLSSANVKTLEAPVGEHSRANNCNPPNLDQNLETELENSISQWGE
NILNPSGKTTASTHLNSKPVVKETSENPKSIVRESDNSKSEPLPPVITTTTVNKVESTPQPEKSLLMKTLSNFWADRSAY
LWKPLVYPTCPSEHIFTDSDVIIREDEPSSLIAFCLSTSDYRNKMMNLNVQQQQQQTAEAAPAKTGGNSGGTTQTGDPS
VNISPSVSTTSHNKGRDSEISSLVTTKEGLLNTPPIEGARDRTPQESQTHSQANLDTLQELEKIMTKKTATHLRYQFEEG
LTVMSCKIFFTEHFDVFRKICDCQENFIQSLSRCVKWDSNGGKSGSGFLKTLDDRFIIKELSHAELEAFIKFAPSYFEYM
AQAMFHDLPTTLAKVFGFYQIQVKSSISSSKSYKMDVIIMENLFYEKKTTRIFDLKGSMRNRHVEQTGKANEVLLDENMV
EYIYESPIHVREYDKKLLRASVWNDTLFLAKMNVMDYSLVIGIDNEGYTLTVGIIDFIRTFTWDKKLESWVKEKGLVGGA
SVIKQPTVVTPRQYKKRFREAMERYILMVPDPWYREGN
>NP_010610.3 Pep7p [Saccharomyces cerevisiae S288C]
MDLENVSCPICLRKFDNLQALNAHLDVEHGFNDNEDSLGSNDSRLVNGKQKKARSVDSSAQKLKRSHWEKFKKGKSCCHT
CGRTLNNNIGAINCRKCGKLYCRRHLPNMIKLNLSAQYDPRNGKWYNCCHDCFVTKPGYNDYGEVIDLTPEFFKVRNIKR
EDKNLRLLQLENRFVRLVDGLITLYNTYSRSIIHNLKMNSEMSKLERTVTPWRDDRSVLFCNICSEPFGLLLRKHHCRLC
GMVVCDDANRNCSNEISIGYLMSAASDLPFEYNIQKDDLLHIPISIRLCSHCIDMLFIGRKFNKDVRMPLSGIFAKYDSM
QNISKVIDSLLPIFEDSLNSLKVETAKDSENTLDPKNLNDLARLRHKLLNSFNLYNTLTRQLLSVEPQSHLERQLQNSIK
IASAAYINEKILPLKSLPAILNPEGHKTNEDGQKAEPEVKKLSQLMIENLTIKEVKELREELMVLKEQSYLIESTIQDYK
KQRRLEEIVTLNKNLEELHSRIHTVQSKLGDHGFN
